# Enantioselective synthesis of hydantoins by chiral acid-catalysed condensation of glyoxals and ureas†

**DOI:** 10.1039/d3sc01656k

**Published:** 2023-06-13

**Authors:** Sushant Aryal, Christopher A. Hone, Matthew I. J. Polson, Daniel J. Foley

**Affiliations:** a School of Physical and Chemical Sciences, University of Canterbury Christchurch New Zealand daniel.foley@canterbury.ac.nz; b Center for Continuous Flow Synthesis and Processing, Research Center Pharmaceutical Engineering Graz Austria; c Biomolecular Interaction Centre, University of Canterbury Christchurch New Zealand

## Abstract

Hydantoins are important scaffolds in natural products and pharmaceuticals, with only a few synthetic strategies available for their asymmetric preparation. We herein describe a single-step enantioselective synthesis of 5-monosubstituted hydantoins *via* condensation of glyoxals and ureas in the presence of a chiral phosphoric acid at room temperature. Products were formed in up to 99% yield and 98 : 2 e.r. Using mechanistic and kinetic studies, including time course ^1^H NMR monitoring, we revealed that the reaction likely proceeds *via* face-selective protonation of an enol-type intermediate.

## Abstract 2

The paper describes the enantioselective synthesis of 5-monosubstituted hydantoins *via* chiral phosphoric acid-catalysed condensation of glyoxals and ureas at room temperature.

## Graphical abstract

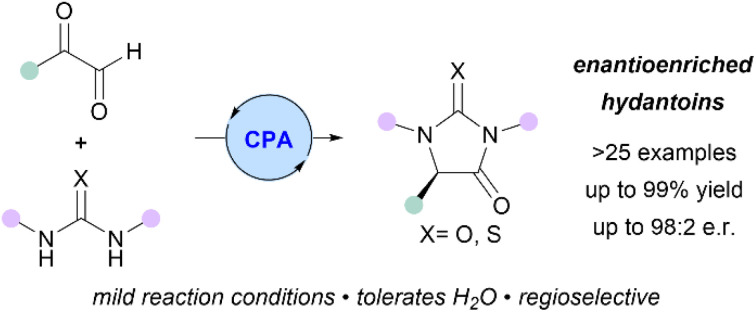

## Introduction

**Fig. 1:**
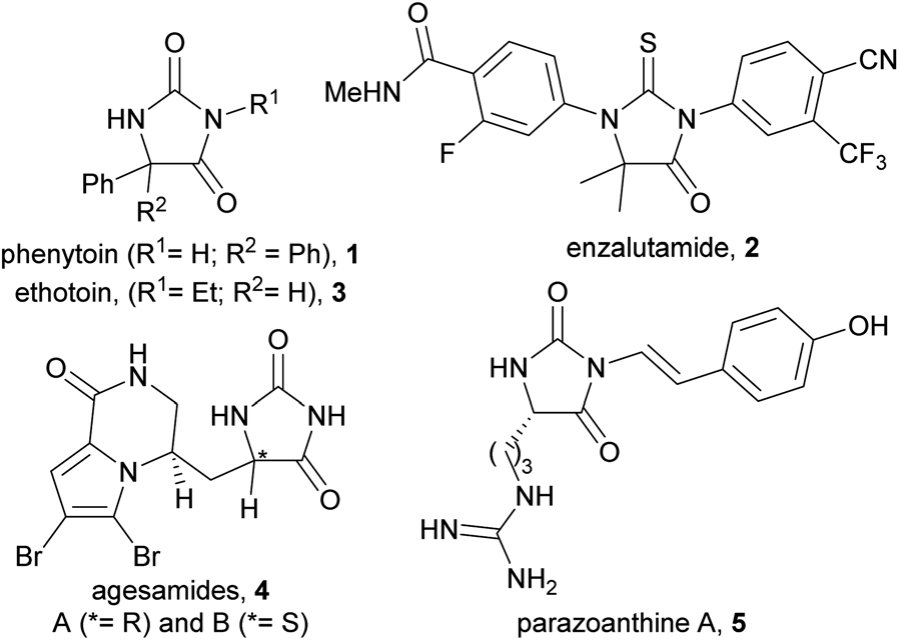
Examples of bioactive hydantoins.

**Fig. 2:**
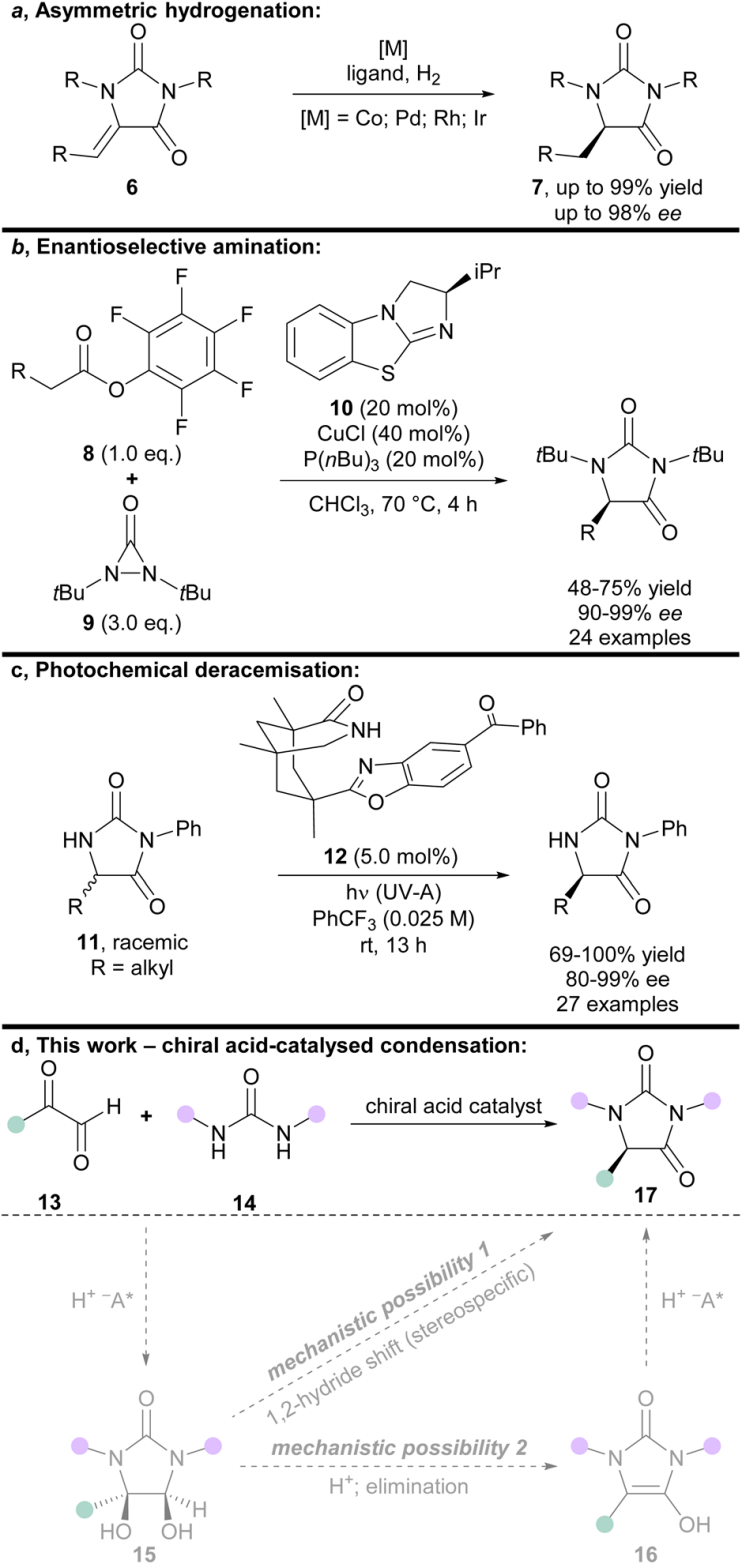
(a–c) State-of-the-art methods for the asymmetric synthesis of hydantoins; (d) chiral acid-catalysed condensation of glyoxals and ureas (this work), including possible mechanisms for enantioinduction (grey arrows/structures).

The hydantoin scaffold exhibits a diverse array of bioactivities, for instance, phenytoin 1 is a well-known anti-seizure treatment, while enzalutamide 2 is a nonsteroidal antiandrogen used in the treatment of prostate cancer (Fig. 1).^1^ Bioactive 5-monosubstituted hydantoins include, amongst others, the phenytoin analogue, ethotoin 3, and the marine natural products agesamides A and B 4^2^ and parazoanthine A 5.^3^ Furthermore, enantioenriched 5-monosubstituted hydantoins serve as useful chiral auxiliaries in a variety of diastereoselective reactions.^4–6^ The presence of various vectors for functionalisation of the hydantoin scaffold, along with the sp^3^-hybridised stereocentre, renders them useful for investigation as “3D” fragments in early-stage drug discovery.^7^ Despite their importance, however, there are relatively few methods available for the asymmetric synthesis of 5-monosubstituted hydantoins from achiral precursors (Fig. 2a–c).

One effective strategy for enantioselective synthesis of 5-monosubstituted hydantoins is the asymmetric hydrogenation of prochiral hydantoins 6 bearing exocyclic alkenes at the 5-position (Fig. 2a). This type of approach was first reported by Takeuchi in 1987,^8^ who used a Co catalyst in the presence of an amine ligand to prepare enantioenriched hydantoins 7 in up to 82% *ee*. More recently, precious metal catalysts have been used to effect the same overall transformation in the presence of chiral phosphine ligands, including Pd^9^ (up to: 96% yield; 90% *ee*), Rh^10^ (up to: 99% yield; 97% *ee*), and Ir^11^ (up to: 99% yield; 98% *ee*). A limitation of this strategy is that it can only deliver hydantoins bearing aliphatic substituents at the 5-position.

Building upon an earlier protocol by Shi,^12^ in 2018 Gong reported the enantioselective α-amination of pentafluorophenyl esters 8 using diaziridinone 9, mediated by cooperative catalysis between Cu(i) and the chiral benzotetramisole catalyst 10 (Fig. 2b).^13^ The enantiodetermining step was postulated to proceed *via* face-selective attack of a urea-derived *N*-centred radical onto a chiral benzotetramisole-derived enamine intermediate. Some limitations of this approach are the need for pentafluorophenyl esters 8 to achieve high *ee*'s, high catalyst (and ligand) loadings, and the use of superheated solvent.

In 2021, Bach introduced an elegant photochemical deracemisation of hydantoins 11 mediated by hydrogen atom transfer in the presence of a chiral diarylketone 12 (Fig. 2c).^14,15^ Some constraints of the approach are that it appears limited to hydantoins that bear an irremovable *N*-phenyl group at the 3-position, and aliphatic groups at the 5-position. It also requires a fluorinated solvent, and low reactant concentrations.

Given the demand for enantiopure hydantoins, the development of new methods for their asymmetric synthesis from easily accessible achiral precursors remains an important area of research. We envisioned that a new enantioselective synthesis of hydantoins could be achieved *via* chiral acid-catalysed condensation of glyoxals and ureas (Fig. 2d).

To the best of our knowledge, the acid-mediated condensation of arylglyoxals and ureas was first reported by Arnold and Möbius in the patent literature in 1970.^16^ Prior to this, Ekeley and Ronzio had reported that the reaction was only successful under base-mediated conditions.^17^ Despite being known for >80 years, both the acid- and base-mediated condensations of glyoxals and ureas have received relatively little attention, with <150 reports in SciFinder to date. Significantly, none of the reported condensations of substituted glyoxals and ureas in the literature are enantioselective.

The acid-mediated condensation of substituted glyoxals 13 and ureas 14 has been suggested^18,19^ to occur *via* a reaction mechanism related to that established for the Biltz hydantoin synthesis from 1,2-diketones and ureas (Fig. 2d, mechanistic possibility 1).^20^ In this mechanism, glyoxals 13 and ureas 14 would first react to form vicinal diol intermediates 15, which would then undergo 1,2-hydride migration (presumably in a stereospecific manner).^18,19^ Vicinal diol intermediates 15 have been isolated and fully characterised previously.^21,22^ However, we envisioned that an alternative mechanism may be possible (Fig. 2d, mechanistic possibility 2); elimination of the vicinal diol intermediate 15 would afford planar enol-type intermediate 16, which could then undergo protonation to provide the hydantoin product 17.

Regardless of whether the reaction proceeds *via* mechanistic possibility 1 or 2 (Fig. 2d), we envisioned that use of an appropriate chiral acid could enable an asymmetric condensation to give hydantoins 17, either by (i) controlling face-selective addition of ureas 14 to glyoxals 13 to give enantioenriched diols 15 (followed by stereospecific 1,2-hydride migration); or (ii) by face-selective protonation of enols 16.

Chiral phosphoric acids (CPAs) have proved immensely powerful for effecting asymmetric reactions in recent years, and are conveniently tuned because of their structural modularity and ease-of-synthesis.^23–27^ We herein describe the first asymmetric synthesis of hydantoins from glyoxals and ureas, using chiral phosphoric acid catalysis.

## Results and discussion

Our studies began by investigating the chiral phosphoric acid-catalysed condensation of phenylglyoxal monohydrate 13a with 1,3-dibenzylurea 14a in CH_3_CN at rt. The first reactions (entries 1–3, Table 1) were performed under air atmosphere and “ordinary” laboratory lighting (overhead 9 W white LEDs inside a fumehood). Initial investigations using (*R*)-BINOL and (*R*)-SPINOL-derived CPAs, 18a and 19a, resulted in complete conversion of the reactants to the hydantoin 17a, but no enantiomeric enrichment of the product was observed (entries 1–2). Use of (*R*)-H_8_-BINOL 20a (5.0 mol%), bearing 9-anthracenyl substituents at the 3 and 3′ positions, afforded the corresponding hydantoin 17a in 40% isolated yield and 85 : 15 e.r., however, a major side product, 5-hydroxyhydantoin 21a (32%) was also formed under the reaction conditions,* as well as several other unidentified products (entry 3).

**Table 1:** Preliminary investigations and initial optimisations

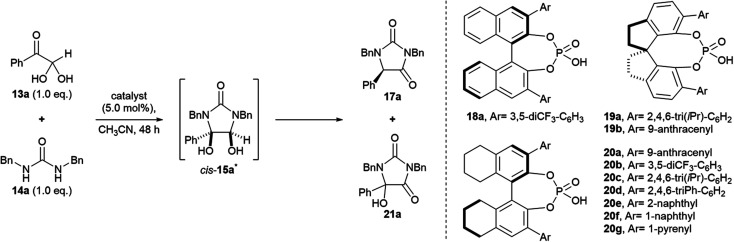					
Entry^a^	Catalyst	Conditions	Ratio 17a : 21a^b^	Isolated yield 17a/%	e.r.^c^17a
1^d^	18a	Air atmosphere, light^h^	100 : 0	98	52 : 48
2^e^	19a	Air atmosphere, light	100 : 0	84	52 : 48
3	20a	Air atmosphere, light	55 : 45^f^	40	85 : 15
4	20a	Ar atmosphere, dark	>99 : trace	98	85 : 15
5	20b	Ar atmosphere, light	100 : 0	99	62 : 38
6	20c	Ar atmosphere, light	100 : 0	nd^g^	50 : 49
7	20d	Ar atmosphere, light	100 : 0	98	70 : 30
8	20e	Ar atmosphere, light	100 : 0	98	72 : 28
9	20f	Ar atmosphere, light	100 : 0	98	81 : 19
10	20g	Ar atmosphere, light	100 : 0	98	80 : 20
11	19b	Ar atmosphere, dark	>99 : trace	98	29 : 71

a Unless indicated, the reaction conditions were: phenylglyoxal monohydrate 13a (0.1 mmol), 1,3-dibenzylurea 14a (0.1 mmol), catalyst (5.0 mol%), CH_3_CN (0.1 M), rt, no stirring, 48 h.

b Ratio determined by analysis of the ^1^H NMR spectra (600 MHz, CD_3_CN) before purification.

c Determined by chiral HPLC (see ESI).

d 24 h.

e 36 h.

f 21a isolated in 32% yield.

g 90 : 10 ratio of 15a to 17a by analysis of the ^1^H NMR spectra (600 MHz, CD_3_CN). Nd = not determined. *Relative stereochemistry shown; major enantiomer unknown (see later).

h Normal overhead fumehood lighting.

Carrying out the reaction using (*R*)-H_8_-BINOL 20a in the absence of oxygen, and in the dark, provided the targeted hydantoin 17a in 98% isolated yield, and 85 : 15 e.r. (entry 4). Presumably, exposure to light (entry 3) leads to singlet oxygen formation *via* photosensitisation of triplet oxygen by the excited 9-anthracenyl rings on (*R*)-H_8_-BINOL 20a. Singlet oxygen subsequently undergoes [2 + 2] cycloaddition with an enol intermediate of type 16, resulting in the formation of 5-hydroxyhydantoin 21a (Fig. S2†).^28,29^ A ^1^H NMR time course experiment over 48 h revealed >99% conversion to the target hydantoin 17a when the reaction was carried out in the absence of oxygen and light; only a marginal trace of 5-hydroxyhydantoin 21a formed (Fig. S1†). Interestingly, in the presence of oxygen, but the absence of light, the ^1^H NMR ratio of 17a:21a after 48 h was 94 : 6, suggesting that a slow background reaction between triplet oxygen and enol 16a (Table S1, entry 6†).^28,29^

Our initial results (entries 1–4) suggested that (i) a H_8_-BINOL backbone, or (ii) large bulky substituents at the 3 and 3′ positions, or (iii) both, were needed in order to prepare the hydantoin products in high e.r. To investigate these hypotheses, first the use of alternate CPAs based on H_8_-BINOL were explored in the reaction (entries 5–11). Notably, H_8_-BINOL catalysts bearing large fused aromatic rings at the 3 and 3′ positions, *e.g.* 1-naphthyl and 1-pyrenyl rings (entries 9 and 10), provided the product in similar yields to (*R*)-H_8_-BINOL 20a (entry 4) but did not improve upon the e.r. achieved (81 : 19 and 80 : 20 e.r., respectively, *vs.* 85 : 15 for 20a). Secondly, the (*R*)-SPINOL CPA 19b, bearing a 9-anthracenyl substituent at the 6 and 6′ positions was explored in the reaction (entry 11). This showed preference for the formation of the (*S*)-enantiomer of hydantoin 17a, which was formed in 98% yield and 29 : 71 e.r. Since our investigations of the CPA structure did not furnish further improvements to the isolated yield and e.r. of the hydantoin product 17a, we chose to proceed and investigate optimisation of other reaction parameters using the anthracenyl-substituted (*R*)-H_8_-BINOL 20a catalyst.

We next chose to investigate the reaction solvent, which can markedly influence the solubility and relative acidity (p*K*_a_) of chiral phosphoric acids.^30^ Conversion to hydantoin 17a was highest in aprotic solvents (Table 2, entries 1–6), with complete conversion and the highest e.r.’s achieved in chlorinated solvents (entries 5–6), in which the catalyst was fully solubilised (*cf*. CH_3_CN). Most notably, when the reaction was run in CHCl_3_ it proceeded to give hydantoin 17a in 99% yield and 96 : 4 e.r. (entry 6). In contrast, in polar protic EtOH, conversion to hydantoin 17a was sluggish, with *cis*-diol 15a being the major component of the reaction mixture at 48 h (entry 7).

**Table 2:** Further optimisations

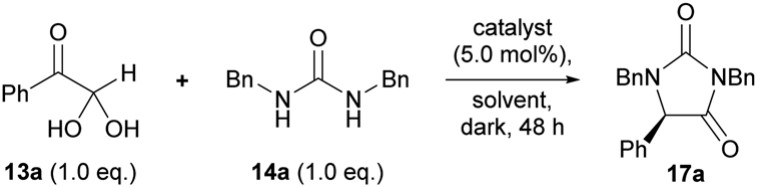					
Entry^a^	Solvent	Catalyst (loading/mol%)	Ratio 15a : 17a^b^	Isolated yield 17a/%	e.r. 17a^c^
1	CH_3_CN	20a	0 : 100^d^	98	85 : 15
2	Et_2_O	20a	28 : 72^d^	71	81 : 19
3	THF	20a	40 : 60^d^	60	76 : 26
4	PhMe	20a	16 : 84^d^	84	86 : 14
5	CH_2_Cl_2_	20a	0 : 100	99	94 : 6
6	CHCl_3_	20a	0 : 100	99	96 : 4
7^e^	EtOH	20a	55 : 29^d^^f^	29	69 : 31
8	CHCl_3_	20f	0 : 100	99	85 : 15
9	CHCl_3_	20g	0 : 100	99	84 : 16
10^g^	CHCl_3_	20a (2.0)	0 : 100	99	95 : 5
11	CHCl_3_	20a (1.0)	0 : 100	98	87 : 13
12	CHCl_3_	20a (0.5)	trace:100	97	85 : 15
13	CHCl_3_	20a (0.1)	10 : 90	90	66 : 34
14^h^	CHCl_3_	20a (2.0)	0 : 100	99	91 : 9
15^i^	CHCl_3_	20a (2.0)	4 : 96	96	97 : 3

a Unless indicated, the reaction conditions were: phenylglyoxal monohydrate 13a (0.1 mmol), 1,3-dibenzylurea 14a (0.1 mmol), catalyst (5.0 mol%), solvent (0.1 M), dark, Ar, rt, no stirring, 48 h.

b Unless indicated, ratio determined by analysis of the ^1^H NMR spectra (600 MHz, K_2_CO_3_-neutralised CDCl_3_) before purification.

c Determined by chiral HPLC (see ESI).

d Ratio determined by analysis of the ^1^H NMR spectra (600 MHz, CD_3_CN) before purification.

e 40 °C.

f 16 : 55 : 29 ratio of 14a : 15a : 17a.

g 20 h.

h 60 °C, 2 h.

i 0 °C, 72 h.

Investigation of the reaction in CHCl_3_ using the other front-running catalysts, 20f and 20g, did not lead to improvements in the e.r. of the hydantoin 17a, although the isolated yield essentially remained the same (entries 8–9). Further investigations therefore focused on optimising the protocol using (*R*)-H_8_-BINOL 20a as the catalyst. Lowering the catalyst 20a loading to 2.0 mol% led to only slight erosion in e.r. (96 : 4 → 95 : 5), with 99% isolated yield, and the reaction was found to be complete at 20 h (entry 10). Further lowering of the catalyst 20a loading led to unacceptable erosion of the e.r. and conversion in 48 h (entries 11–13). Heating the reaction to 60 °C reduced the reaction time to two hours, expediently providing hydantoin 17a in 99% isolated yield and with slightly diminished e.r. (91 : 9, entry 14). To improve the e.r. of the product 17a, the method in entry 14 may be coupled with recrystallisation (see below). When the reaction temperature was lowered to 0 °C (entry 15), the e.r. of hydantoin product 17a was improved to 97 : 3, but at the expense of both reaction time (incomplete at 72 h) and isolated yield (96%). The presence of drying agents did not alter the enantioselectivity of the process (Table S2, entries 1–6; 8†). Addition of activated molecular sieves slowed the reaction down (entries 3–6; 8), however, addition of 10 eq. H_2_O had no effect on the yield, e.r., or reaction time (entry 7).

Based on the high yield and e.r. of hydantoin 17a produced when 2.0 mol% (*R*)-H_8_-BINOL 20a was used at room temperature for 20 h (entry 10), these conditions were chosen to explore the substrate scope.

**Scheme 1:**
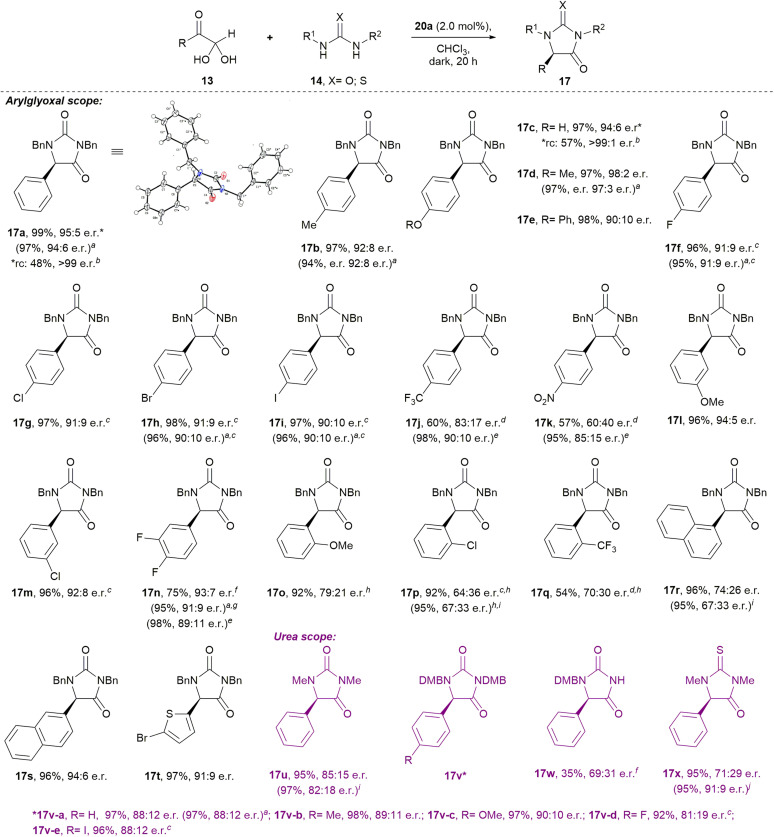
Phosphoric acid-catalysed condensation of arylglyoxals with ureas to give enantioenriched hydantoins. Standard reaction conditions: 13 (0.1 mmol), 14 (0.1 mmol), catalyst 20a (2.0 mol%), CHCl_3_, dark, Ar, rt, no stirring, NMR tube. (a) Scaled-up reaction conditions: 13 (0.5 mmol), 14 (0.5 mmol), catalyst 20a (2.0 mol%), CHCl_3_, dark, Ar, rt, stirring, glass vial; (b) yield and e.r. after recrystallisation; (c) 40 h; (d) 7 days; (e) 60 °C, 4 h; (f) 72 h; (g) 5 days; (h) 13 (0.15 mmol) used; (i) 60 °C, 2 h; (j) 0 °C, 72 h; (k) 60 °C, 0.5 h. rc = recrystallisation (unoptimised) of the sample marked with an asterisk. DMB = 2,4-dimethoxybenzyl.

Our optimised conditions (Table 2, entry 10) were directly applicable to a range of glyoxal (and glyoxal hydrate) starting materials to prepare enantioenriched hydantoins (Scheme 1). Glyoxals bearing electron-rich (4-Me; 4-OH; 4-OMe; 4-OPh) and moderately electron deficient (4-F; 4-Cl; 4-Br; 4-I) aryl rings were well tolerated in the protocol, with all yields ≥96% and e.r.’s ≥90 : 10. Electron deficient 4-CF_3_- and 4-NO_2_-phenyglyoxal monohydrates, performed sluggishly in the reaction (→17j–k), with incomplete conversion after seven days at rt. Analysis by ^1^H NMR at 600 MHz revealed the ratio of *cis*-diol 15j to hydantoin 17j to be 38 : 62, while the ratio of 15k to hydantoin 17k was 35 : 65. Interestingly, heating these reactions to 60 °C gave complete conversion to hydantoins 17j–k in just 4 h. Hydantoin 17j was isolated in 98% yield and 90 : 10 e.r., while compound 17k was isolated in 95% yield and 85 : 15 e.r.

Substituents at the aryl 3-position were well tolerated in the reaction. 3-OMe-phenylglyoxal hemihydrate gave hydantoin 17l in 96% yield and 94 : 5 e.r., while 3-Cl-phenylglyoxal hemihydrate gave 17m in 96% yield and 92 : 8 e.r. Electron deficient 3,4-(difluoro)phenylglyoxal monohydrate reacted to give hydantoin 17n in 75% isolated yield and 93 : 7 e.r. at rt, although the reaction was incomplete at 72 h (the ratio of 15n to 17n was 23 : 77 when the crude reaction mixture was analysed by ^1^H NMR at 600 MHz). At rt, the reaction only went to completion after five days, giving compound 17n in 95% yield and 91 : 9 e.r. However, heating the reaction to 60 °C provided compound 17n in 98% yield and 89 : 11 e.r. after 4 hours.

The reaction of sterically hindered 2-substituted arylglyoxals produced hydantoins with lower e.r.’s (→17o–q; 64 : 36–79 : 21 e.r.), which was also observed in the case of 1-naphthylglyoxal hemihydrate (→17r; 74 : 26 e.r.). However, reaction of the sterically less hindered 2-naphthylglyoxal monohydrate yielded 17s in 96% yield and 94 : 6 e.r. Heteroaromatic 5-bromo-2-thiopheneglyoxal monohydrate gave 17t in 97% yield and 91 : 9 e.r.

It is noteworthy that recrystallisation of the hydantoin products 17 could improve their e.r., for instance for 17a and 17c, in each case recrystallisation improved the e.r. to >99 : trace. Additionally, the absolute configurations of hydantoins 17a, 17f, and 17h were determined through single-crystal X-ray diffraction (see ESI†).

Excitingly, preliminary studies with alkylglyoxals demonstrated that the corresponding hydantoins S17y-ac can be prepared enantioselectively, albeit that further optimisation is required in future (see Scheme S1†).

Brief investigation of the urea component revealed that high yields and reasonable e.r.’s are maintained when 1,3-dimethylurea (→17u) and 1,3-*di*DMB-urea were used in the protocol (→17v). Interestingly, 1-DMB-urea performed sluggishly in the reaction, but regioselectively provided the 1-protected hydantoin 17w in 35% yield and 69 : 31 e.r. after 72 h (incomplete conversion). When 1,3-dimethylthiourea was reacted with phenylglyoxal monohydrate at rt, thiohydantoin 17x was isolated in 95% yield and 71 : 29 e.r. When the reaction temperature was dropped to 0 °C, conversion to the thiohydantoin 17x was incomplete after 72 h (the ratio of *cis*-diol 15x to thiohydantoin 17x was 11 : 89), and thiohydantoin 17x was isolated in 86% yield and 62 : 38 e.r. Curiously, however, heating the reaction to 60 °C for 0.5 h gave thiohydantoin 17x in 95% yield and 91 : 9 e.r.

**Fig. 3:**
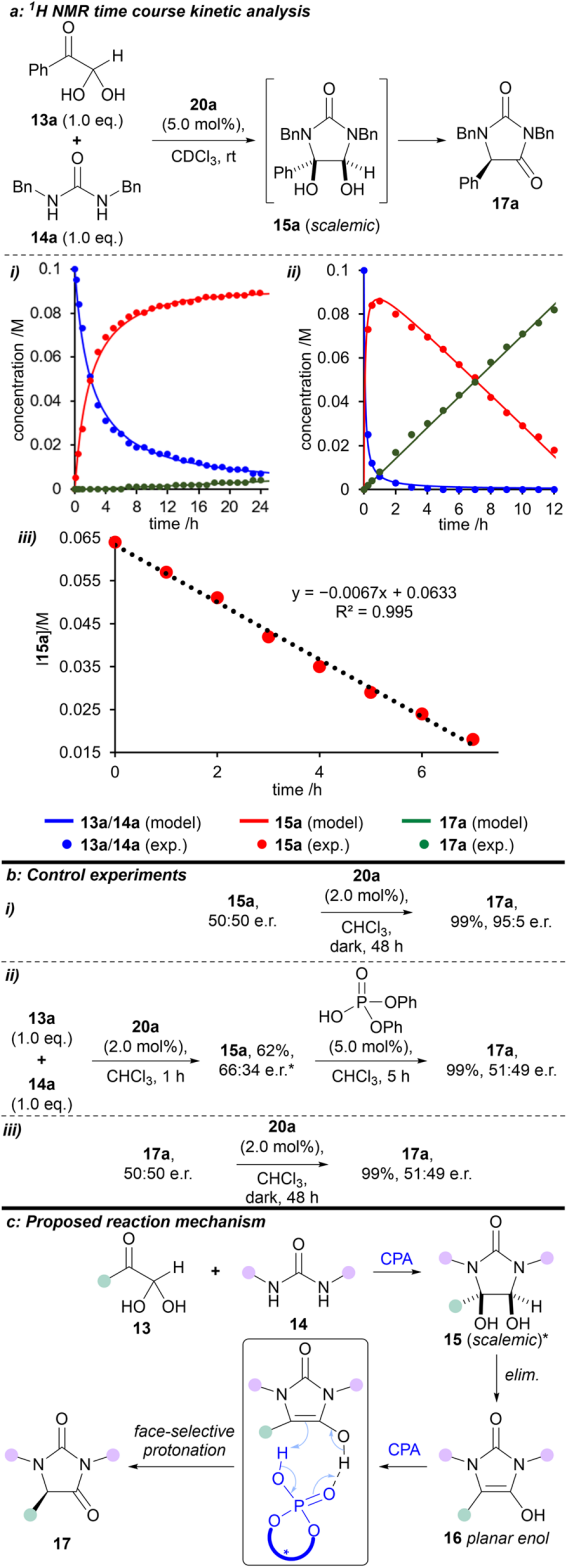
Kinetic and mechanistic studies. (a) ^1^H NMR-derived time course kinetic analysis of the reaction between phenylglyoxal monohydrate 13a and 1,3-dibenzylurea 14a in CDCl_3_ at rt (600 MHz): (i) Reaction progress without catalyst; (ii) Reaction progress with catalyst 20a; (iii) Variable time normalisation analysis (VTNA) concentration results against time with catalyst 20a. (b) Control experiments. (c) Proposed reaction mechanism. *Relative stereochemistry shown; major enantiomer unknown. exp. = experimental. elim. = elimination.

Time course ^1^H NMR studies were used to investigate the kinetics of the condensation reaction in CDCl_3_ over 24 hours, both in the presence and absence of catalyst 20a. A global optimisation algorithm was used to determine the two rate constants within the model by maximising the convergence of the model-predicted reaction outcomes to the experimental data (Fig. 3a, i-iii). In the absence of catalyst 20a, the reaction between phenylglyoxal monohydrate and 1,3-dibenzylurea proceeded to make *cis*-diol 15a, which barely reacted further (Fig. 3a–i). The first step was fitted as a second-order process, and the intramolecular cyclisation as a first-order process, to obtain rate constants of 4.9 M^−1^ h^−1^ and 0.0020 h^−1^, respectively. However, with the addition of 2.0 mol% (*R*)-H_8_-BINOL 20a, both of these steps were considerably faster (Fig. 3a-ii). Second-order formation of the *cis*-diol 15a proceeded with a rate of 150 M^−1^ h^−1^. Interestingly, the subsequent formation of hydantoin 17a in the rate-determining step displayed linear (zero-order) behaviour with a fitted rate constant of 0.0070 M h^−1^. By using the variable time normalisation analysis (VTNA) technique,^31^ we graphically fitted the kinetic plot for the (*R*)-H_8_-BINOL 20a-catalysed reaction with an ‘artificial zero’ after full conversion of the starting materials (from 5 h), and at a known concentration for intermediate 15a. Subsequently, reaction of intermediate 15a to form the corresponding product 17a in the rate-determining step could be fitted with increased accuracy (rate constant = 0.0067 M h^−1^; and *R*^2^ = 0.995, Fig. 3a-iii). This behaviour switched to first-order when 15a was nearly consumed (see Fig. S13†). Future studies will endeavour to gain a deeper kinetic insight into the reaction system using approaches outlined by others.^32–35^

To gain a deeper understanding of the reaction mechanism, a series of experiments were carried out (Fig. 3b). First, we prepared and isolated racemic *cis*-diol 15a (see ESI†) and then exposed it to the optimised reaction conditions (Fig. 3b–i). We obtained the corresponding hydantoin 17a in 95 : 5 e.r., which is consistent with the e.r. obtained for the reaction between phenylglyoxal monohydrate and 1,3-dibenzylurea under the same conditions (*cf*. Scheme 1).

Secondly, we reacted phenylglyoxal monohydrate with 1,3-dibenzylurea in the presence of (*R*)-H_8_-BINOL 20a for 1 hour at rt to afford *cis*-diol 15a in 62% yield and 66 : 34 e.r. (see ESI†). When this scalemic *cis*-diol 15a was treated with (achiral) diphenylphosphoric acid, hydantoin 17a was isolated as a racemate (Fig. 3b-ii).

Finally, exposing racemic hydantoin 17a to (*R*)-H_8_-BINOL 20a for 48 hours resulted in recovery of racemic 17a (Fig. 3b-iii).

Based on the above kinetic and experimental observations, we can explain the origin of enantioselectivity in the reaction (*R*)-H_8_-BINOL 20a catalyses formation of (scalemic) *cis*-diol 15 then, in the enantiodetermining step, converts *cis*-diol 15 to enantioenriched hydantoin 17, presumably *via* face-selective protonation of a transient, planar enol-type intermediate 16 (Fig. 3c). Further support for the reaction mechanism proceeding through a planar enol intermediate 16 is provided by the observed formation of 5-hydroxyhydantoin 21a when the reaction is performed in the presence of singlet (and to a lesser extent triplet) oxygen.

**Scheme 2:**
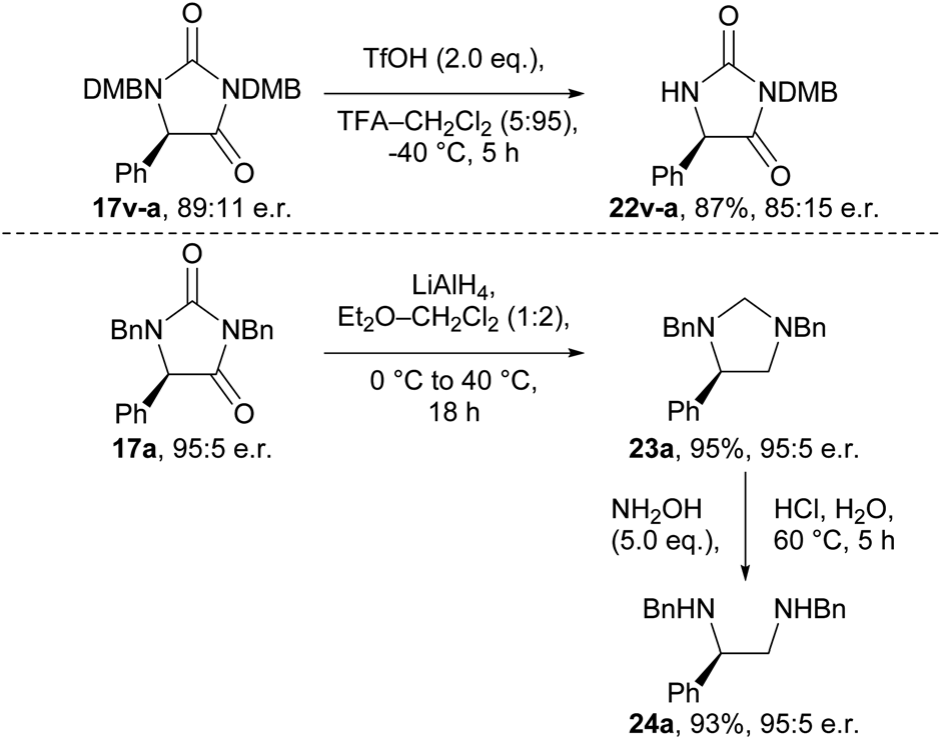
Synthetic modification of the enantioenriched hydantoin.

To demonstrate the utility of the hydantoin products formed, we briefly investigated their synthetic modification to provide various chiral building blocks (Scheme 2). Our attempts to remove the *N*-benzyl groups *via* hydrogenation and other conditions were unfortunately unsuccessful (Tables S17 and S18†). However, we were able to remove the 2,4-dimethoxybenzyl (DMB) group from *N*-1 of hydantoin 17v-a using TfOH at −40 °C to give compound 22v-a, albeit with some erosion in e.r. (89 : 11 → 85 : 15). Additionally, we were able to obtain imidazolidine 23a in 95% yield, and with complete retention of e.r., by reduction of enantioenriched hydantoin 17a using LiAlH_4_.^36^ Subsequent treatment of imidazolidine 23a with hydroxylamine revealed vicinal diamine 24a, a ligand scaffold used in enantioselective metal-catalysed reactions.^37,38^

## Conclusions

In summary, we have established a new asymmetric synthesis of hydantoins *via* the chiral phosphoric acid-catalysed condensation of glyoxals and ureas at room temperature. The reaction proceeds in high yields and enantioselectivities using a variety of substituted aryl glyoxals. Mechanistic investigations revealed that the enantiodetermining step likely arises from the face-selective protonation of a transient enol-type intermediate. Further development of this approach, for instance by modular structural variation of the chiral phosphoric acid catalyst, holds great promise for the broad application of this strategy in the enantioselective synthesis of hydantoins.

## Data availability

The relevant data is detailed in the ESI.†

## Author contributions

DJF conceptualised the idea and supervised the experimental work. SA completed all of the experimental work (except XRD). CH analysed the ^1^H NMR time course studies carried out by SA, and derived the reaction rates. MIJP determined the crystal structures and absolute configurations of 17a, 17f, and 17h by XRD. All authors contributed to writing the paper and ESI.†

## Conflicts of interest

There are no conflicts to declare.

## Supplementary Material

SC-014-D3SC01656K-s001

SC-014-D3SC01656K-s002
