# Supplementary material for: Enantioselective synthesis of hydantoins by chiral acid-catalysed condensation of glyoxals and ureas
Source: Chem Sci. 2023 Jun 13;14(29):7905–12. doi: 10.1039/d3sc01656k (PMC10370572; doi:10.1039/d3sc01656k)
Supplement: SC-014-D3SC01656K-s001 [file SC-014-D3SC01656K-s001.pdf]

Electronic Supplementary Material (ESI) for Chemical Science.
This journal is © The Royal Society of Chemistry 2023

Enantioselective synthesis of hydantoins by chiral acid-catalysed condensation of glyoxals and ureas

Sushant Aryal,[1] Christopher A. Hone,[2] Matthew I. J. Polson,[1] and Daniel J. Foley*[1,3]

[1]*School of Physical and Chemical Sciences, University of Canterbury, Christchurch, New Zealand.*

[2]*Center for Continuous Flow Synthesis and Processing, Research Center Pharmaceutical Engineering, Graz, Austria.*

[3]*Biomolecular Interaction Centre, University of Canterbury, Christchurch, New Zealand.*

Supporting Information

1. Contents

1. Contents 1
2. General experimental 3
2. Reaction development in CH_3CN with catalyst 20a 5
3. Mechanistic suggestion for the formation 5-hydroxyhydantoin 21a 7
4. Investigation of reaction additives 8
5. Investigation of reaction concentration 9
6. Investigation of reactant stoichiometry 9
7. Time course ^{1}H NMR monitoring in CD_3CN 10
8. Time course ^{1}H NMR monitoring in $CDCl_3$ 13
   8.1 Without catalyst 13
   8.2 With catalyst 20a 16
9. Kinetic fitting 19
   9.1 In the absence of catalyst 19
   9.2 In the presence of catalyst 20a 20
   9.3 Graphical fitting of 15a conversion in the presence of catalyst 20a 20
   9.4 Variable time normalization analysis (VTNA) 30
10. Arylglyoxal substrate scope for asymmetric hydantoin synthesis 35
11. Alkylglyoxal substrate scope for asymmetric hydantoin synthesis 71

12. Reactions of enantioenriched hydantoins 77
12.1 Attempted deprotection of 1,3-*di*DMB hydantoin 77
12.2 Synthesis of an enantioenriched vicinal diamine 79
13. Synthesis of *cis*-diol 15a and 5-hydroxyhydantoin 21a 82
14. Limitations of the phosphoric acid-catalysed hydantoin synthesis 85
14.1 Phenylglyoxal diethyl acetal 85
14.2 Unsubstituted urea 86
14.3 Attempted glycouril synthesis 86
15. Attempted *N*-benzyl deprotection 87
15.1 Catalytic hydrogenation 87
15.2 NBS-facilitated hydrolysis 87
16. Synthesis of substituted ureas 88
17. Synthesis of aryl / alkyl glyoxals 91
18. Crystal data 112
18.1 Crystal data for 17a 112
18.2 Crystal data for 17f 113
18.3 Crystal data for 17h 114
19. References 115
20. Processed NMR spectra 116
21. Aryl / alkyl glyoxal ^{1}H NMR and IR spectra 205
22. HPLC traces 240

2. General experimental

Unless stated, all reactions in non-aqueous solvents were carried out under an atmosphere of nitrogen in oven-dried glassware. Anhydrous DCM, DMF, Et_2O, hexane, MeCN, MeOH, PhMe, and THF were obtained from the departmental solvent delivery system: each solvent was degassed with nitrogen and dried by passage over an activated Al_2O_3 column. All other anhydrous solvents were obtained in SureSeal bottles from Sigma-Aldrich. Petrol refers to petroleum ether (b.p. 40-60 °C). De-ionised water was purified by reverse osmosis using a Millipore Milli-Q Gradient purification system equipped with Rephile RR400Q101 and RRQTUM0EX purification cartridges, and a RAFFC0250 filter. All chiral phosphoric acids used were purchased from Strem and Sigma-Aldrich. As well as purchasing catalyst **20a**, we also prepared it by the route described by Bernardi.[1] All other commercial starting materials were sourced from Sigma-Aldrich, AKSci and Alfa Aesar.

Normal laboratory lighting levels refers to the reaction being performed under standard overhead lighting inside a fumehood (9 W white LEDs).

Thin Layer chromatography was carried out on aluminium-backed silica plates (silica gel 60 F_{254}). The plates were visualised by the quenching of fluorescence under an ultraviolet lamp (λ_{max} = 254 nm) and by staining with $KMnO_4$. Flash chromatography was performed using silica gel 60 (230-400 mesh) supplied by Chem-Supply.

Melting points were recorded on a Mettler Toledo MP90 Melting Point System, and are uncorrected.

Optical rotation values were recorded on a Rudolph Research Analytical Autopol IV Automatic Polarimeter at the sodium D-line (589 nm). Specific rotations were calculated, and are given in deg $dm^{-1}cm^3\ g^{-1}$ (units are omitted). Samples were measured at room temperature; concentrations are in mg/mL.

Infrared spectra were recorded on a Bruker Alpha II FTIR, and the resulting spectra were processed using Opus.

High resolution accurate mass samples were analysed by Dr Amanda Inglis and Dr Marie Squire on a maXis 3G UHR-Qq-TOF mass spectrometer (Bruker Daltonik GmbH, Bremen, Germany) coupled to a Dionex Ultimate 3000 LC system (ThermoFisher). 5 µL sample was injected into a flow of 50:50 H_2O (0.5% HCO_2H):MeCN at 0.2 mL/min, 5 µL ESI-L Low Concentration Tuning Mix (Agilent Technologies) was injected after each sample as a calibrant. The data was processed using Compass software (Bruker Daltonik GmbH, Bremen, Germany).

Proton (^1H) and carbon (^{13}C) NMR spectra were recorded using either a JEOL JNM-ECZ400S spectrometer (at 400 and 100 MHz, respectively) or a JEOL JNM-ECZ600R spectrometer (at 600 and 150 MHz, respectively). Fluorine (^{19}F) NMR spectra were recorded using JEOL JNM-ECZ600R spectrometer (at 565 MHz). Chemical shifts (δ) are reported in ppm. Coupling constants (*J*) are quoted in Hertz (Hz) and splitting

patterns reported in an abbreviated manner: app. (apparent), s (singlet), d (doublet), t (triplet), q (quartet), m (multiplet). Fully assigned compounds were assigned with COSY, DEPT-135, TOCSY, HMBC and HSQC (or HMQC) experiments. Compounds are numbered with respect to their IUPAC names, which were generated using MarvinSketch. All spectra were processed, interpreted, and assigned using MestreNova.

High-performance liquid chromatography (HPLC) was performed on a Dionex Ultimate 3000 LC system (ThermoFisher) using Astec® Cellulose DMP or CHIRALPAK® AS-3R as the chiral stationary phase. HPLC-grade solvents were used, which were purchased from ThermoFisher and Sigma-Aldrich. For each experiment, the solvent mixture used is reported. HPLC chromatograms were processed using Chromeleon™ Dionex Version 7.2.

X-ray crystallography studies were performed by Dr Matthew Polson. Single crystals were selected and mounted on a nylon loop in perfluorinated oil on a SuperNova, Dual, Cu at home/near, HyPix diffractometer. The crystal was kept at 120.0 (2) K during data collection. Using Olex2,[2] the structure was solved with the SHELXT [3] structure solution program using Intrinsic Phasing and refined with the SHELXL [4] refinement package using Least Squares minimisation. Hydrogen atoms on carbon atoms were placed in calculated positions and treated as riding atoms during refinement with a fixed Uiso 1.2 times the carbon atom.

2. Reaction development in CH_3CN with catalyst 20a

Table S1: Preliminary reaction condition development with catalyst **20a** in CH_3CN.

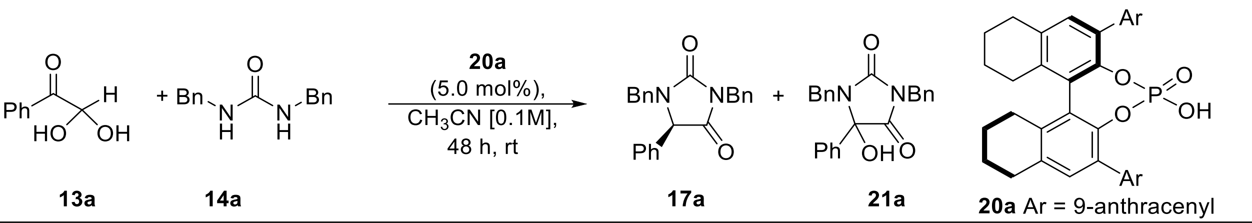

Entry[a]	Reaction set-up	Stirring / no stirring	Conditions	Ratio **17a:21a**[b]	Isolated yield **17a** /%[c]	e.r. **17a** [d]
1[e]	glass vial*	stirring	air atmosphere, light†	55:45	40	85:15
2	glass vial	stirring	Ar atmosphere (balloon), **dark**	84:16	nd	85:15
3	glass vial	stirring	Ar atmosphere, **dark**	98:2	nd	nd
4	glass vial	no stirring	Ar atmosphere, **dark**	99:1	nd	nd
5[e]	NMR tube‡	no stirring	Air atmosphere, light	90:10	nd	nd
6[e]	NMR tube	no stirring	air atmosphere, **dark**	94:6	nd	nd
7	NMR tube	no stirring	Ar atmosphere, light	96:4	nd	nd
8	NMR tube	no stirring	Ar atmosphere, **dark**	>99:trace	98	85:15
9[f]	NMR tube	no stirring	Ar atmosphere, **dark**	>99:trace	98	85:15

[a] Unless otherwise stated reaction condition were: phenylglyoxal monohydrate **13a** (0.1 mmol), 1,3-dibenzylurea **14a** (0.1 mmol), catalyst **20a** (5.0 mol%), Ar-degassed CH_3CN (1.0 mL, 0.1 M), the reaction set up was purged with Ar for 1-2 min then sealed with 5-7 layers of parafilm. [b] Ratio determined by analysis and integration of the crude ^1H NMR spectra (600 MHz, CD_3CN). [c] Isolated yield after flash chromatography. [d] Determined by chiral HPLC (see General Experimental). [e] Non-degassed CH_3CN. [f] CD_3CN instead of CH_3CN (for time course ^1H NMR monitoring). nd = not determined. *Supelco® 27151 7 mL glass vial from Sigma Aldrich, †Normal overhead fumehood lighting (see General Experimental). ‡Wilmad® Z271993 5 mL borosilicate NMR tube from Sigma Aldrich.

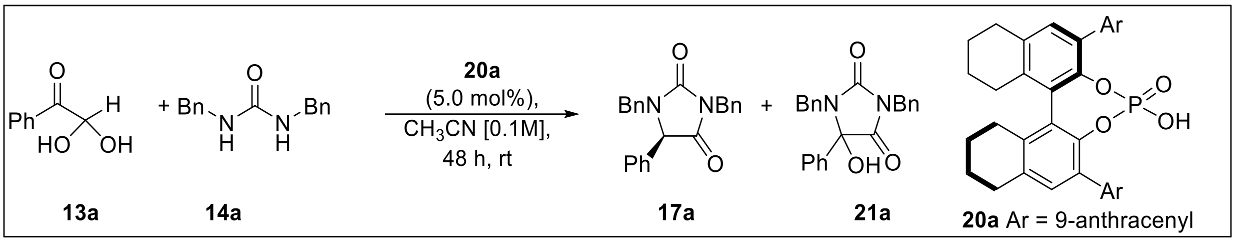

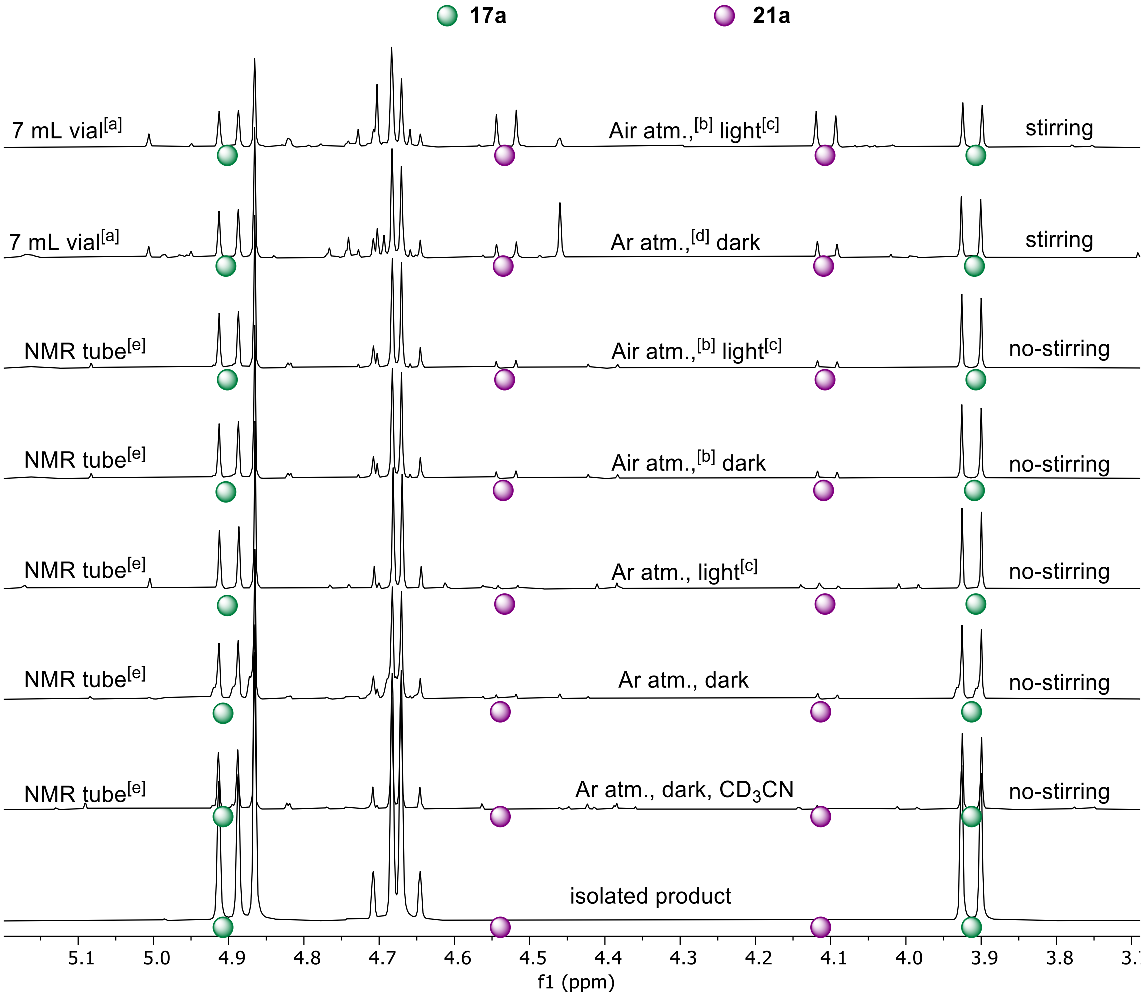

Figure S1: Stacked crude ^{1}H NMR spectrum showing the reaction progress at 48 h using catalyst **20a**. Conditions: phenylglyoxal monohydrate **13a** (0.1 mmol), 1,3-dibenzylurea **14a** (0.1 mmol), catalyst **20a** (5.0 mol%), Ar-degassed CH_3CN (1.0 mL, 0.1 M), reaction set up purged with Ar for 1-2 min and sealed with 5-7 layers of parafilm. [a] 7 mL glass vial (Supelco® 27151). [b] Air atmosphere, non-degassed CH_3CN. [c] Normal overhead fumehood lighting (see General Experimental). [d] Under Argon (balloon). [e] 5 mL borosilicate NMR tube (Wilmad® Z271993).

3. Mechanistic suggestion for the formation 5-hydroxyhydantoin 21a

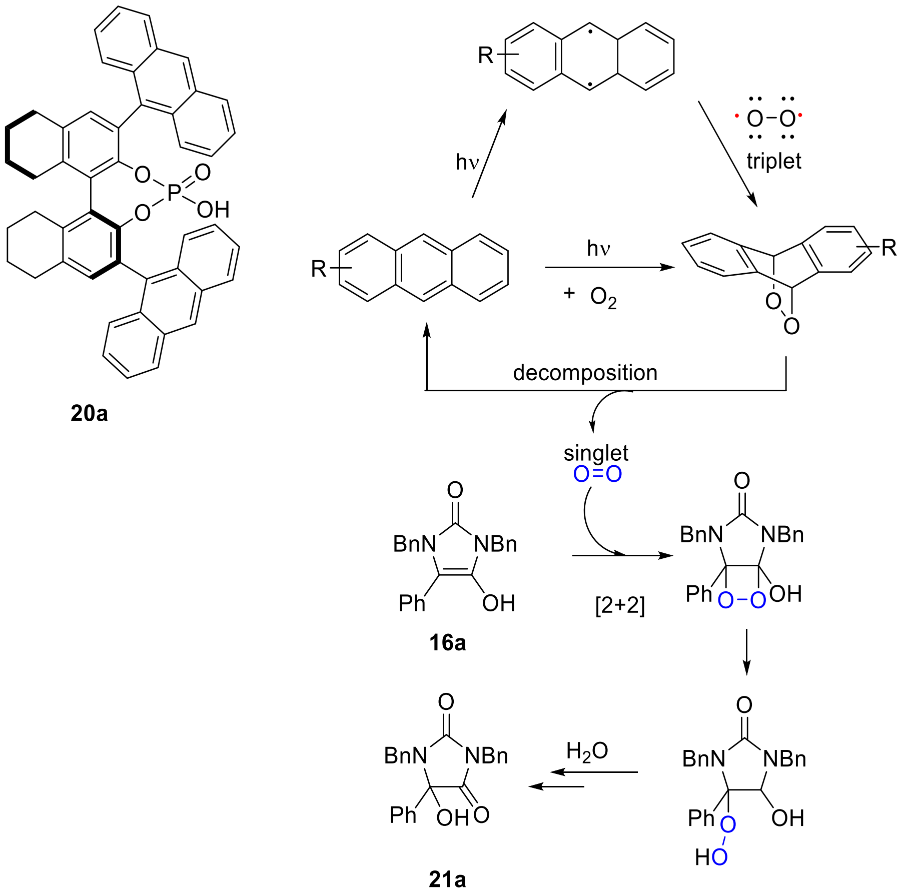

Figure S2: Mechanistic suggestion for the formation of 5-hydroxyhydantoin product **21a**.[5,6]

4. Investigation of reaction additives

Table S2: Investigation of reaction additives.

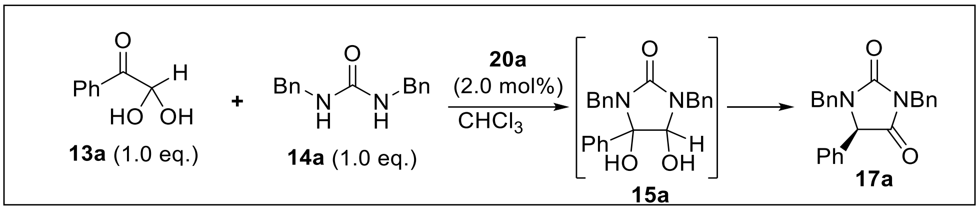

Entry[a]	Additive	Time\h	Ratio **15a:17a**[b]	Isolated yield **17a** /%[c]	e.r. **17a** [d]
1	$MgSO_4$ (2.0 eq.)	20	0:100	99	95:5
2	Na_2SO_4 (2.0 eq.)	20	0:100	99	95:5
3	3 Å MS (40 mg)[e]	20	35:65	nd	nd
4	3 Å MS (40 mg)[e]	48	0:100	99	95:5
5	4 Å MS (40 mg)[e]	20	20:80	nd	nd
6	4 Å MS (40 mg)[e]	48	0:100	99	95:5
7	H_2O (10.0 eq.)	20	0:100	99	95:5
8[f]	4 Å MS (40 mg)[e]	2	0:100	99	92:8
9[f]	H_2O (10.0 eq.)	2	0:100	99	92:8

[a] Unless otherwise stated reaction condition were: phenylglyoxal monohydrate **13a** (0.1 mmol), 1,3-dibenzylurea **14a** (0.1 mmol), catalyst **20a** (2.0 mol%), Ar-degassed $CHCl_3$ (1.0 mL, 0.1 M), the reaction set up was purged with Ar for 1-2 min then sealed with 5-7 layers of parafilm at rt. [b] Ratio determined by analysis and integration of the crude ^{1}H NMR spectra (600 MHz, K_2CO_3-neutralised $CDCl_3$). [c] Isolated yield after flash chromatography. [d] Determined by chiral HPLC (see General Experimental). [e] ~265 wt% relative to **13a**. [f] reaction at 60 °C. MS = Molecular sieves (4-8 mesh) activated using high vacuum at 200 °C for 20 h followed by slow cooling in a N_2 environment. nd = not determined.

5. Investigation of reaction concentration

Table S3: Investigation of reaction concentration.

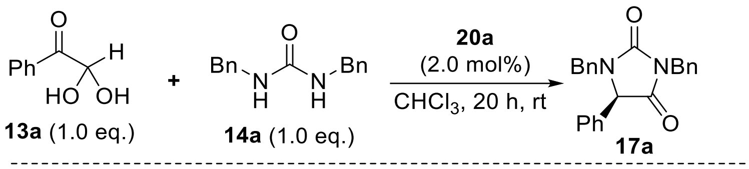

Entry[a]	Concentration/ [M]	Isolated yield **17a** /%[b]	e.r. **17a** [c]
1	0.05	98	94:6
2	0.1	99	95:5
3	0.2	99	95:5

[a] *Reaction condition*: phenylglyoxal monohydrate **13a** (0.1 mmol), 1,3-dibenzylurea **14a** (0.1 mmol), catalyst **20a** (2.0 mol%), Ar-degassed $CHCl_3$ (x mL), the reaction set up was purged with Ar for 1-2 min then sealed with 5-7 layers of parafilm at rt. [b] Isolated yield after flash chromatography. [c] Determined by chiral HPLC (see General Experimental)

6. Investigation of reactant stoichiometry

Table S4: Investigation of **13a** and **14a** stoichiometry.

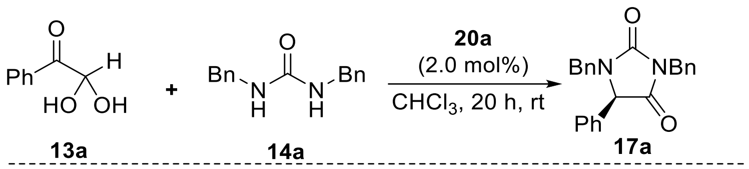

Entry[a]	**13a** /mmol	**14a** /mmol	Ratio **13a:14a** /eq.	Isolated yield **17a** /%[b]	e.r. **17a** [c]
1	0.10	0.10	1.0:1.0	98	95:5
2	0.11	0.10	1.0:1.1	99	95:5
3	0.11	0.10	1.1:1.0	99	95:5

Reaction condition: phenylglyoxal monohydrate **13a** (mmol provided), 1,3-dibenzylurea **14a** (mmol provided), catalyst **20a** (2.0 mol%), Ar-degassed $CHCl_3$ (1.0 mL, 0.1 M), the reaction set up was purged with Ar for 1-2 min then sealed with 5-7 layers of parafilm at rt. [b] Isolated yield after flash chromatography. [c] Determined by chiral HPLC (see General Experimental)

7. Time course ^{1}H NMR monitoring in CD_3CN

For images of the reaction set up, see **Figures S3-4**.

To an oven dried NMR tube* charged with 1,3-dibenzyl urea **14a** (24 mg, 101 µmol, 1.0 eq.) and catalyst **20a** (3.6 mg, 5.0 µmol, 5.0 mol%) was added *via* cannula phenylglyoxal monohydrate **13a**† (15 mg, 99 µmol, 1.0 eq.) and mesitylene (*internal standard*, 3.5 µL, 0.33 eq.) in CD_3CN (1.0 mL, 0.1 M). The reaction tube headspace was briefly purged using an Ar balloon before attaching the tube cap. The reaction sample was loaded into the NMR spectrometer (Bruker AV600). After optimising the tuning and shimming, the ^{1}H NMR measurements were taken at approximately t = ~15 min, 30 min, then at every hour for 48 h. During this process, the NMR tube was not withdrawn from the NMR spectrometer. The crude conversion of phenylglyoxal monohydrate **13a** and 1,3-dibenzylurea **14a** to intermediate diol **15a** and product **17a** was monitored by integrating known the product peaks versus the internal standard (mesitylene).

* Wilmad® NMR tubes 5 mm diam., precision frequency 300 MHz, L 7 in., purchased from Sigma-Aldrich (Wilmad® Z271993)
† Vendor: Alfa Aesar.

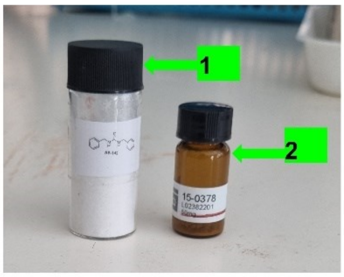

1. 1,3 dibenzylurea **14a**
2. Catalyst **20a**

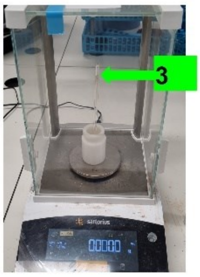

3. Tare the Wilmad® Z271993 NMR tube on weighing scale

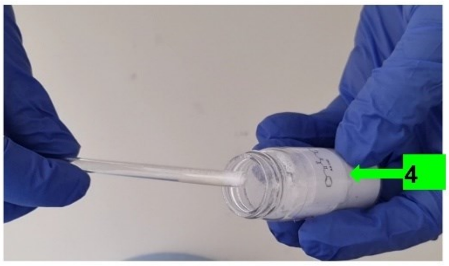

4. Charge the Wilmad® Z271993 NMR tube with 1,3 dibenzylurea **14a**

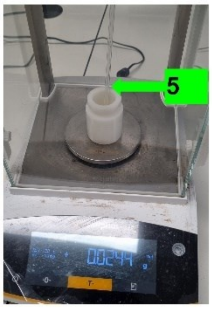

5. weigh out 1,3 dibenzylurea 14a

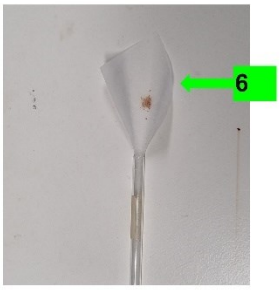

6. Weigh out catalyst in butter paper **20a**

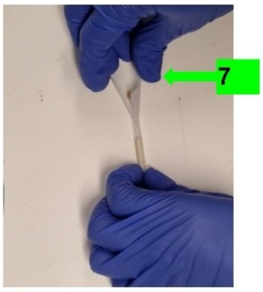

7. Charge the Wilmad® Z271993 NMR tube with catalyst **20a**

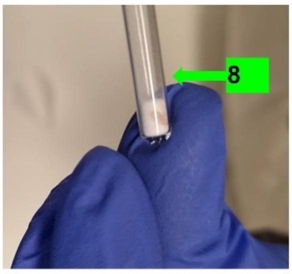

8. Tap the tube a make 1,3 dibenzylurea **14a** and catalyst **20a** at bottom

Figure S3: Charging an NMR tube with 1,3-benzylurea **14a** and catalyst **20a**.

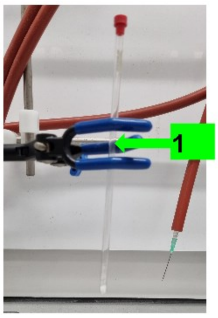

1. NMR tube charged with 1,3 dibenzylurea **14a** and catalyst **20a**

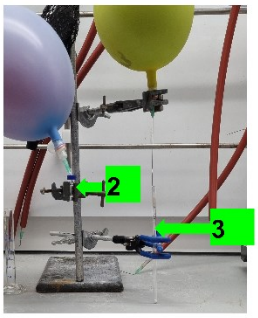

2. GC vial charged with phenylglyoxal monohydrate **13a** and mesitylene in reaction solvent

3. NMR tube charged with 1,3 dibenzylurea **14a** and catalyst **20a** under Argon balloon.

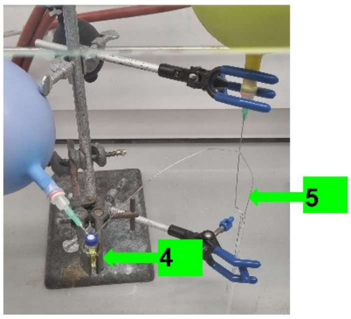

4. Cannulation of GC Vial to NMR tube

5. NMR tube wrapped with Aluminium foil under Argon.

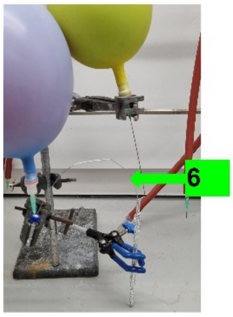

6. Phenylglyoxal monohydrate **13a** and mesitylene transfer to NMR tube via cannulation of GC Vial.

Figure S4: Transferring phenylglyoxal monohydrate **13a** and mesitylene dissolved in the reaction solvent into an NMR tube.

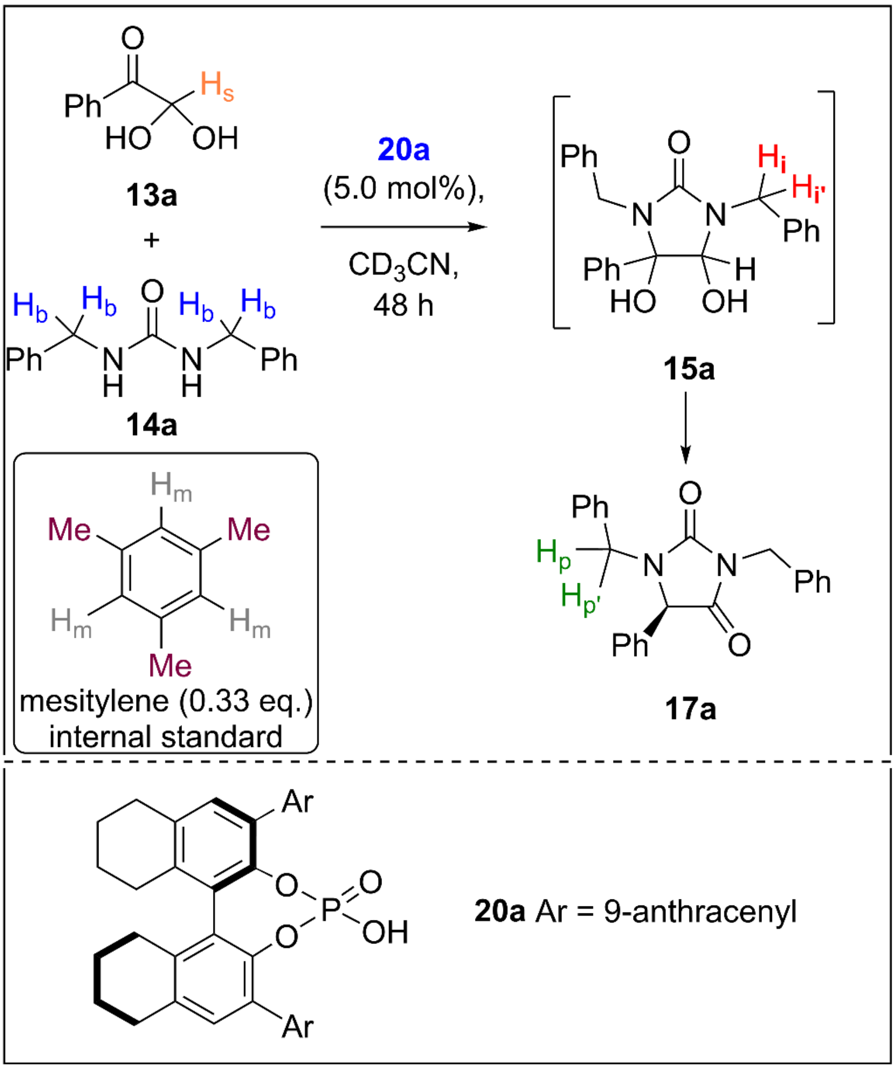

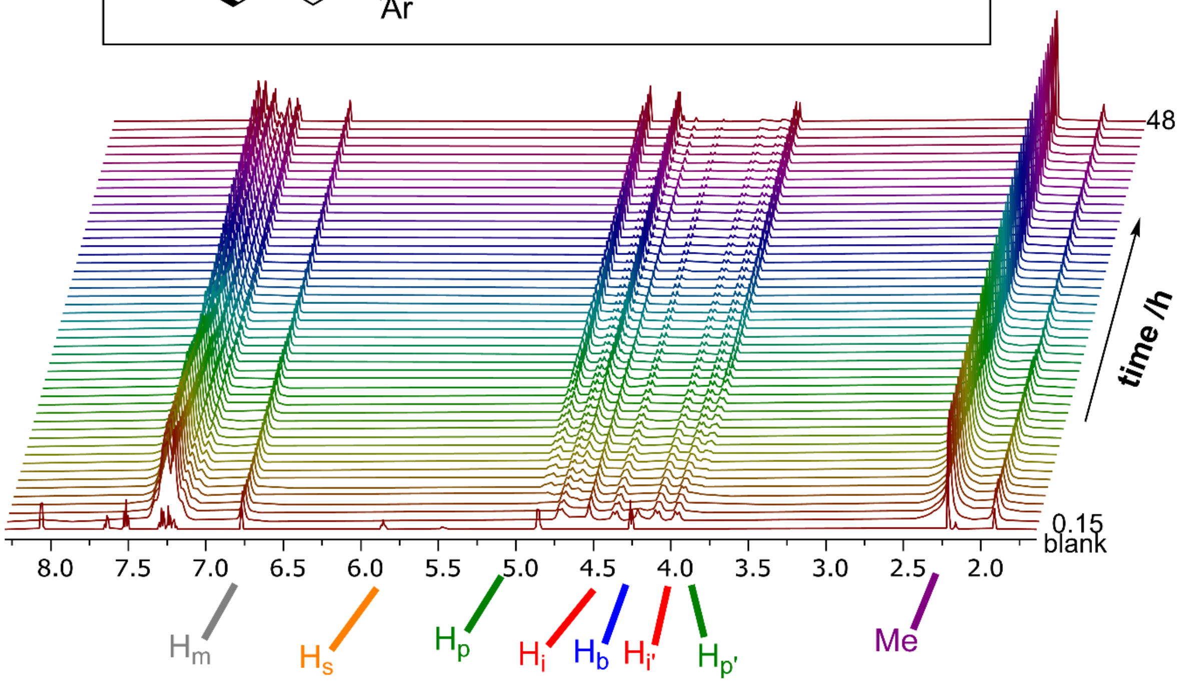

Figure S5: Time course ^{1}H NMR monitoring for chiral phosphoric acid **20a**-catalysed reaction of phenylglyoxal monohydrate **13a** (0.1 mmol) and 1,3-dibenzylurea **14a** (0.1 mmol) in CD_3CN (1.0 mL, 0.1 M) analysed at 600 MHz.

8. Time course ^{1}H NMR monitoring in $CDCl_3$

8.1 Without catalyst

For images of the following reaction set up, see **Figures S3-4**.

To an oven dried NMR tube* charged with 1,3-dibenzyl urea **14a** (24 mg, 101 µmol, 1.0 eq.) was added *via* cannula phenylglyoxal monohydrate **13a**† (15 mg, 99 µmol, 1.0 eq.) and mesitylene (*internal standard*, 3.5 µL, 0.33 eq.) in $CDCl_3$ (1.0 mL, 0.1 M). The reaction tube headspace was briefly purged using an Ar balloon before attaching the tube cap. The reaction sample was loaded into the NMR spectrometer (Bruker AV600). After optimising the tuning and shimming, the ^{1}H NMR measurements were taken at approximately t = ~15 min, 30 min, then at every hour for 24 h. During this process, the NMR tube was not withdrawn from the NMR spectrometer. The crude conversion of phenylglyoxal monohydrate **13a** and 1,3-dibenzylurea **14a** to intermediate diol **15a** and product **17a** was monitored by integrating known the product peaks versus the internal standard (mesitylene).

* Wilmad® NMR tubes 5 mm diam., precision frequency 300 MHz, L 7 in., purchased from Sigma-Aldrich (Wilmad® Z271993)
† Vendor: Alfa Aesar.

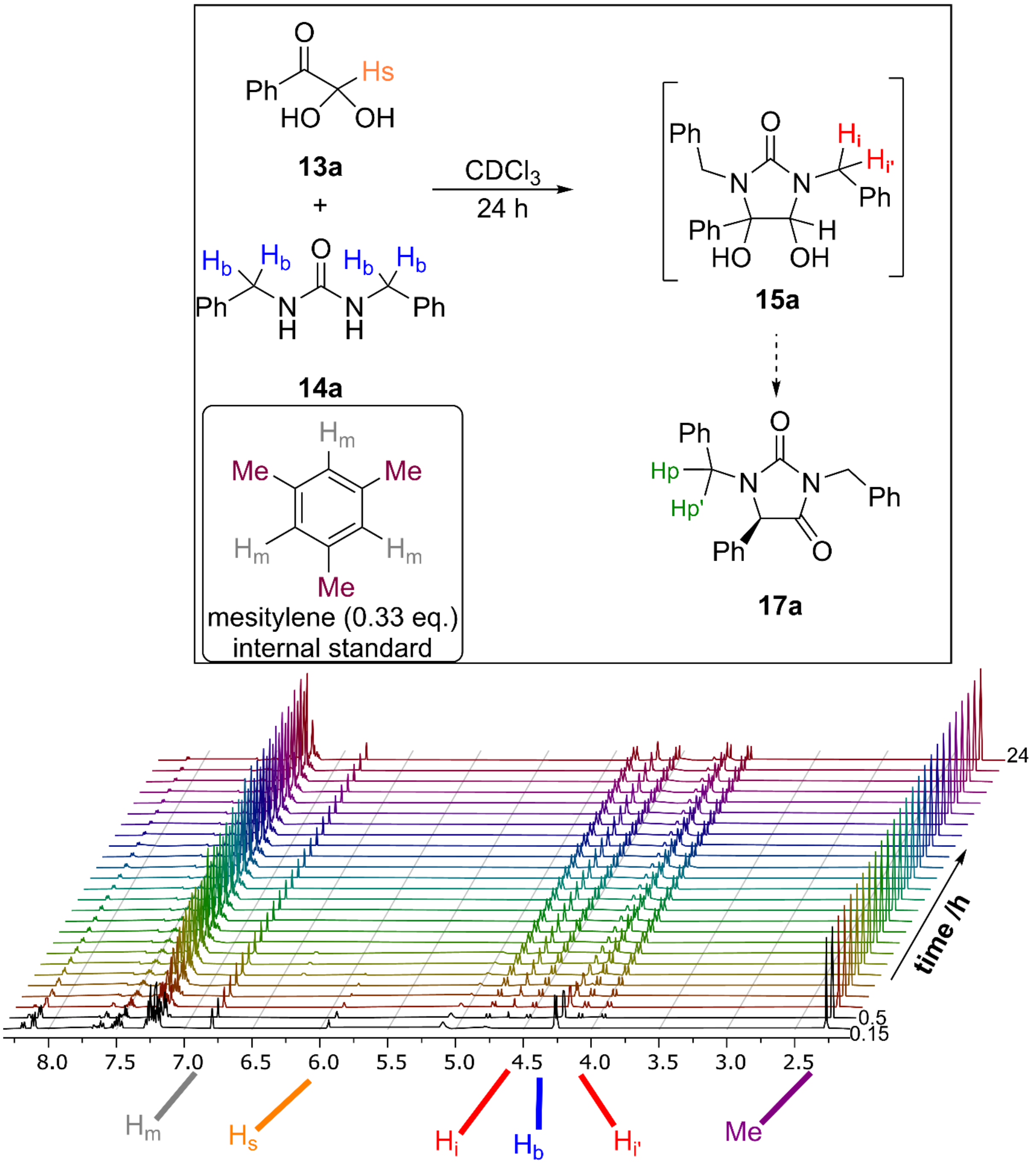

Figure S6: Time course ^{1}H NMR monitoring for the reaction of phenylglyoxal monohydrate **13a** (0.1 mmol) and 1,3-dibenzylurea **14a** (0.1 mmol) in the absence of a catalyst in CD_3CN (1.0 mL, 0.1 M) analysed at 600 MHz.

Table S5: ^{1}H NMR conversion (%) vs time (h) in the absence of a catalyst in $CDCl_3$.

Time /h	**Conversion %**			
	**13a**	**14a**	**15a**	**17a**
0	100	100	0	0
0.25	95	95	5	0
0.5	84	84	16	0
1	73	73	27	0
2	51	51	49	0
3	38	38	62	0
4	31	31	69	0
5	27	27	73	0
6	25	25	75	0
7	21	21	78	1
8	19	19	80	1
9	19	19	80	1
10	17	17	82	1
11	16	16	83	1
12	16	16	83	1
13	14	14	84	2
14	13	13	85	2
15	13	13	85	2
16	12	12	86	2
17	11	11	87	2
18	10	10	87	3
19	10	10	87	3
20	9	9	88	3
21	9	9	88	3
22	9	9	88	3
23	7	7	89	4
24	7	7	89	4

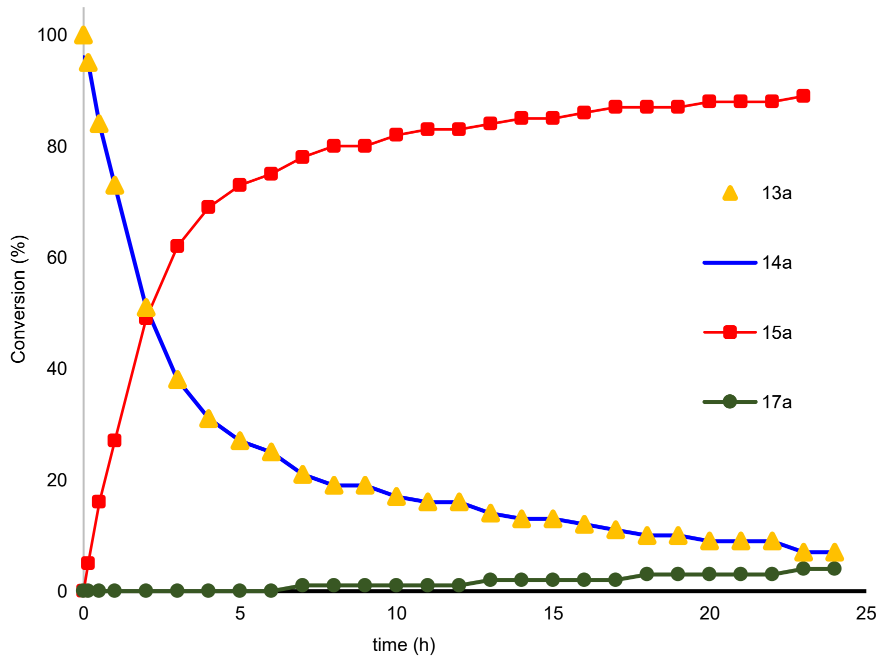

Figure S7: Conversion (%) vs time (h) in the absence of a catalyst in $CDCl_3$.

8.2 With catalyst 20a

For images of the following reaction set up, see **Figures S3-4**:

To an oven dried NMR tube* charged with 1,3-dibenzyl urea **14a** (24 mg, 101 μmol, 1.0 eq.) and catalyst **20a** (1.5 mg, 2.0 μmol, 2.0 mol%) was added *via* cannula phenylglyoxal monohydrate **13a**† (15 mg, 99 μmol, 1.0 eq.) and mesitylene (*internal standard*, 3.5 μL, 0.33 eq.) in $CDCl_3$ (1.0 mL, 0.1 M). The reaction tube headspace was briefly purged using an Ar balloon before attaching the tube cap. The reaction sample was loaded into the NMR spectrometer (Bruker AV600). After optimising the tuning and shimming, the ^1H NMR measurements were taken at approximately t = ~15 min, 30 min, then at every hour for 24 h. During this process, the NMR tube was not withdrawn from the NMR spectrometer. The crude conversion of phenylglyoxal monohydrate **13a** and 1,3-dibenzylurea **14a** to intermediate diol **15a** and product **17a** was monitored by integrating known the product peaks versus the internal standard (mesitylene).

* Wilmad® NMR tubes 5 mm diam., precision frequency 300 MHz, L 7 in., purchased from Sigma-Aldrich (Wilmad® Z271993)
† Vendor: Alfa Aesar.

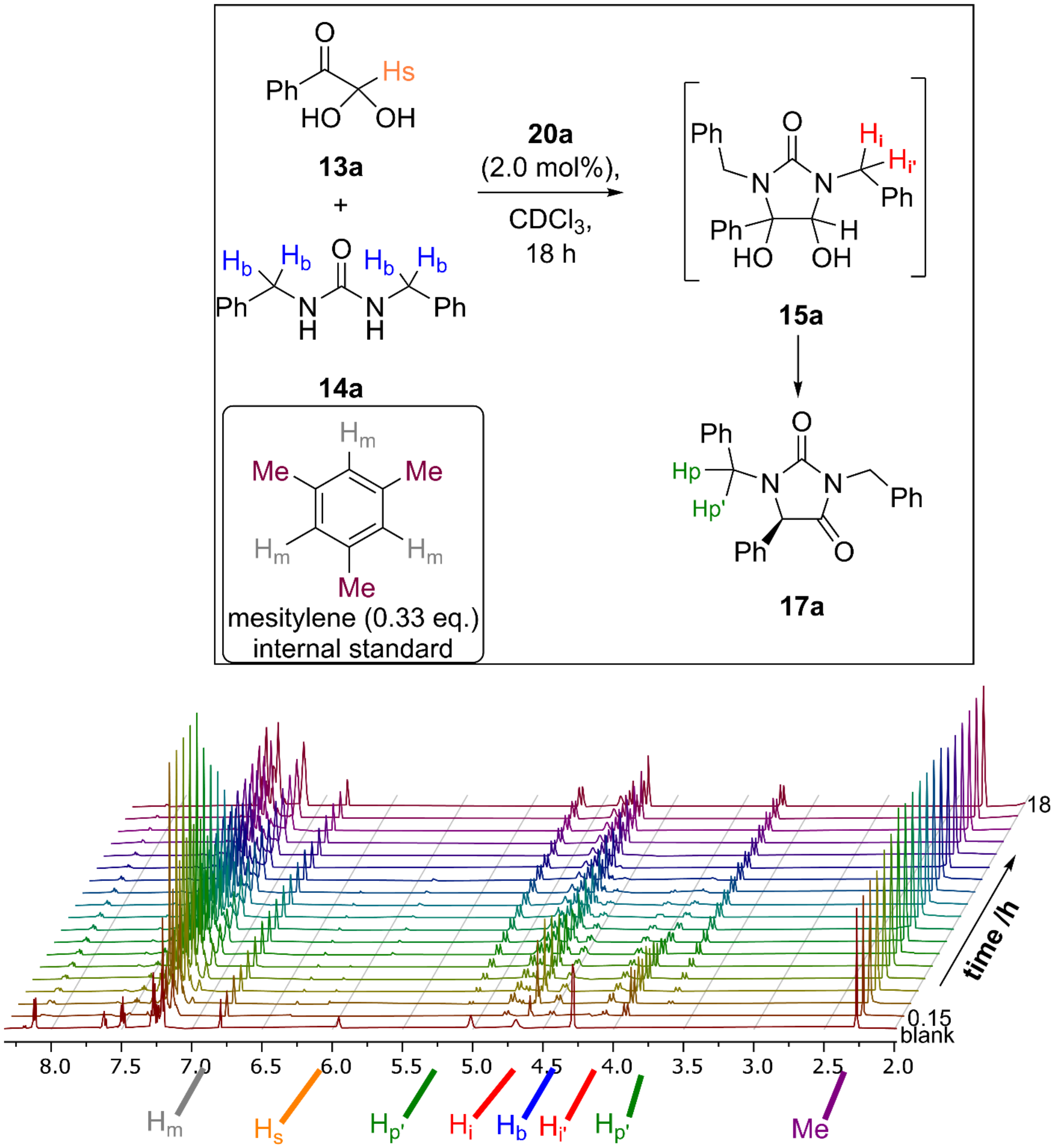

Figure S8: Time course ^{1}H NMR monitoring for the chiral phosphoric acid 2**0a**-catalysed reaction of phenylglyoxal monohydrate **13a** (0.1 mmol) and 1,3-dibenzylurea **14a** (0.1 mmol) in $CDCl_3$ (1.0 mL, 0.1 M) analysed at 600 MHz.

Table S6: ^{1}H NMR conversion percentage against time for the reaction with catalyst **20a** in $CDCl_3$.

**Time /h**	**Conversion (%)**			
	**13a**	**14a**	**15a**	**17a**
0	100	100	0	0
0.25	25	25	73	2
0.5	12	12	84	4
1	6	6	86	8
2	3	3	80	17
3	1	1	74	25
4	0.5	0.5	69.5	30
5	0	0	64	36
6	0	0	57	43
7	0	0	51	49
8	0	0	42	58
9	0	0	35	65
10	0	0	29	71
11	0	0	24	76
12	0	0	18	82
13	0	0	14	86
14	0	0	10	90
15	0	0	7	93
16	0	0	5	95
17	0	0	3	97
18	0	0	0	99

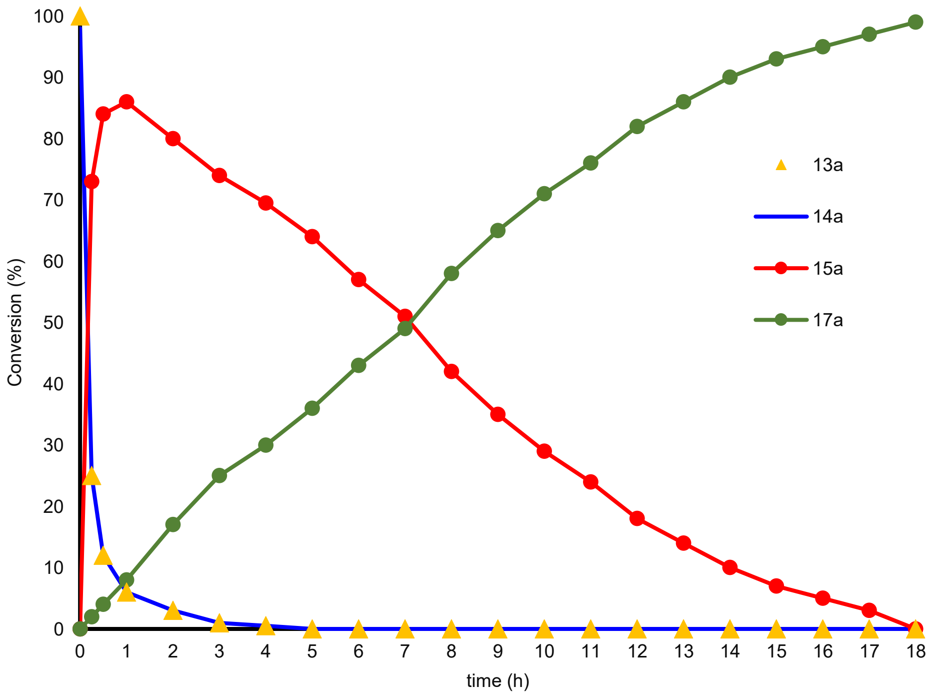

Figure S9: Conversion % vs time plots obtained from the ^{1}H NMR measurements.

9. Kinetic fitting

The rate constants were estimated for the reaction profiles using a software package called Compunetics (v3.1.1) (https://compunetics.net/). The software automatically adjusted the rate constant values based to improve the candidate model fit to the experimental data. The sum of squared error (SSE) difference between the ordinary differential equation (ODE) curves and the experimental data were minimised to arrive at the final estimated rate constant values. A global optimisation algorithm, pattern search, was used for the fitting. The errors on fitted rate constant values are absolute errors, whereby this is the maximum possible error in the ODE based on fittings of each permutation of the ±2.5% relative error specified concentration for each of the species fitted. Moreover, the maximum variance was determined from the original fitting, and reported as the absolute error in the parameter fit. The statistical analysis was used to determine the most likely reaction model based on model simplicity and agreement with experimental data.

9.1 In the absence of catalyst

Two rate limiting steps were simultaneously fitted for the profiling data in the absence of catalyst: (i) k_1 - the bimolecular (second order) reaction of **13a** and **14a** to form the intermediate **15a**; and (ii) k_2 the (first order) reaction of intermediate **15a** to the corresponding product **17a**.

Table S7: Rate constant (k_1 and k_2) estimates with error for fitting of reaction profiles for no catalyst. SSE = 1.67E-04 M.

Parameter	Value ± error
k_1	4.88 ± 0.32 M^{-1} h^{-1}
k_2	0.00196 ± 0.00083 h^{-1}

9.2 In the presence of catalyst 20a

The same model structure as described above was attempted for the reaction system in the presence of catalyst, but this displayed a significant lack-of-fit for the conversion of intermediate **15a** to the corresponding product **17a** (see Figure S11). Thus, two rate limiting steps were simultaneously fitted for the profiling data in the presence of catalyst: (i) k_1 - the bimolecular (second order) reaction of **13a** and **14a** to form the intermediate **15a**; and (ii) k_2 the (zero order) reaction of intermediate **15a** to the corresponding product **17a**.

Table S8: Rate constant (k_1 and k_2) estimates with error for fitting of reaction profiles for catalyst **20a**. SSE = 1.80E-04 M.

Parameter	Value ±error
k_1	150 ± 15 M^{-1} h^{-1}
k_2	0.00703 ± 0.00015 M h^{-1}

9.3 Graphical fitting of 15a conversion in the presence of catalyst 20a

Due to the rapid reaction between **13a** and **14a** to form the intermediate **15a**, the kinetic plot for the experiment containing catalyst has also been fitted with an 'artificial zero', whereby at the 'artificial zero' there is already full conversion of the starting material. This technique of fitting kinetics with an artificial zero is akin to variable time normalisation analysis (VTNA) analysis, whereby the time axis is shifted, asserting an experimental 'start time' at 'artificial zero', when concentrations are known for the intermediate and product.[7] This can fix fitting discrepancies that may occur, as the early measured time points due to the relatively fast conversion of starting material may not be accurate. First order and zero order fits were considered, with a zero-order fit showing a better fit to the experimental data (see Figure S15 and S16)

Table S9: Rate constant (k_2) estimates with error for fitting of reaction profiles with catalyst added.

	Value	R^2
First order - k_2	0.180 h^{-1}	0.984
Zero order - k_2	0.0067 M h^{-1}	0.995

Table S10: Experimental concentration results against time for the reaction in the absence of a catalyst in $CDCl_3$.

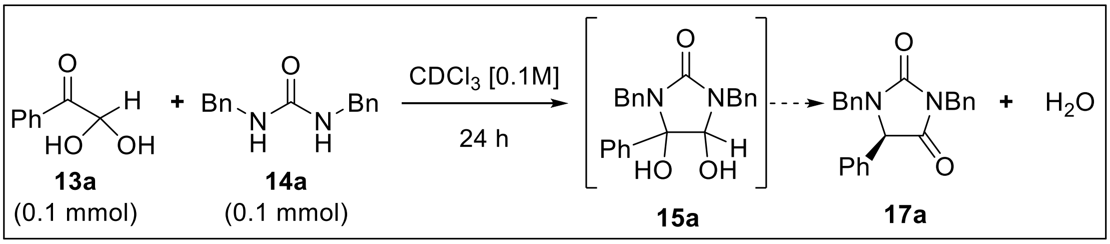

Time /h	Concentration [M]				
	**13a**	**14a**	**15a**	H_2O	**17a**
0	0.1	0.1	0	-	0
0.15	0.095	0.095	0.005	-	0
0.5	0.084	0.084	0.016	-	0
1	0.073	0.073	0.027	-	0
2	0.051	0.051	0.049	-	0
3	0.038	0.038	0.062	-	0
4	0.031	0.031	0.069	-	0
5	0.027	0.027	0.073	-	0
6	0.025	0.025	0.075	-	0
7	0.021	0.021	0.078	-	0.001
8	0.019	0.019	0.08	-	0.001
9	0.019	0.019	0.08	-	0.001
10	0.017	0.017	0.082	-	0.001
11	0.016	0.016	0.083	-	0.001
12	0.016	0.016	0.083	-	0.001
13	0.014	0.014	0.084	-	0.002
14	0.013	0.013	0.085	-	0.002
15	0.013	0.013	0.085	-	0.002
16	0.012	0.012	0.086	-	0.002
17	0.011	0.011	0.087	-	0.002
18	0.01	0.01	0.087	-	0.003
19	0.01	0.01	0.087	-	0.003
20	0.009	0.009	0.088	-	0.003
21	0.009	0.009	0.088	-	0.003
22	0.009	0.009	0.088	-	0.003
23	0.007	0.007	0.089	-	0.004
24	0.007	0.007	0.089	-	0.004

		value	± error
**13a+14a→15a+H_2O**	k_1	4.88	0.32 M^{-1} h^{-1}
**15a→17a+H_2O**	k_2	0.00196	0.00083 h^{-1}
	SSE	1.67E-04	M

Table S11: Model based concentration results against time for the reaction in the absence of a catalyst in $CDCl_3$.

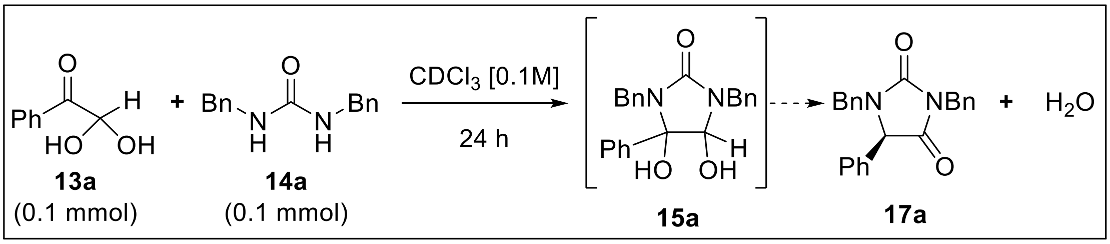

Time /h	Concentration (M)				
	**13a**	**14a**	**15a**	**H_2O**	**17a**
0	0.1	0.1	0	0	0
0.005178	0.099748	0.099748	0.000251667	0.000251671	2.1415E-09
0.010356	0.099498	0.099498	0.0005021	0.000502113	6.7483E-09
0.015534	0.099249	0.099249	0.000751281	0.000751309	1.386E-08
0.041681	0.098008	0.098008	0.001992019	0.001992189	8.5226E-08
0.067827	0.096797	0.096797	0.003202673	0.003203109	2.1772E-07
0.093974	0.095616	0.095616	0.004383878	0.0043847	4.1077E-07
0.120121	0.094463	0.094463	0.00553655	0.005537876	6.6319E-07
0.192233	0.09142	0.09142	0.008578474	0.008581778	1.6521E-06
0.264345	0.088567	0.088567	0.011430195	0.011436303	3.0544E-06
0.336458	0.085887	0.085887	0.014108451	0.014118142	4.8454E-06
0.40857	0.083364	0.083364	0.016628615	0.016642619	7.0018E-06
0.579983	0.077931	0.077931	0.022055713	0.022082673	1.348E-05
0.751397	0.073164	0.073164	0.026813955	0.026857274	2.166E-05
0.922811	0.068946	0.068946	0.031023009	0.031085673	3.1332E-05
1.094225	0.065185	0.065185	0.034772637	0.034857294	4.2328E-05
1.265638	0.061813	0.061813	0.038132627	0.038241644	5.4507E-05
1.564911	0.056701	0.056701	0.043220439	0.043376987	7.8273E-05
1.864183	0.052372	0.052372	0.047523009	0.047732543	0.00010477
2.163455	0.048654	0.048654	0.051212105	0.051479254	0.00013357
2.462727	0.045427	0.045427	0.054408846	0.054737603	0.00016438
2.762	0.042598	0.042598	0.057205131	0.057598978	0.00019692
3.258579	0.038618	0.038618	0.061127879	0.061636342	0.00025423
3.755159	0.035319	0.035319	0.064366445	0.064996416	0.00031498
4.251739	0.032535	0.032535	0.067086526	0.067843722	0.00037859
4.748319	0.030155	0.030155	0.069399947	0.0702892	0.00044462
5.244899	0.0281	0.0281	0.071387185	0.072412638	0.00051272
5.975875	0.025543	0.025543	0.073841188	0.07507354	0.00061617
6.706851	0.023411	0.023411	0.075866673	0.077312271	0.00072279
7.437827	0.021604	0.021604	0.077564316	0.079228418	0.00083205
8.168803	0.020054	0.020054	0.079002176	0.080889218	0.00094351
8.89978	0.018713	0.018713	0.080230219	0.082343979	0.00105687
10.01496	0.016983	0.016983	0.081783798	0.084249582	0.00123289
11.13014	0.015544	0.015544	0.083044155	0.085868041	0.00141194
12.24532	0.014325	0.014325	0.084081038	0.08726797	0.00159346
13.3605	0.013283	0.013283	0.084939893	0.088493956	0.00177702
14.47568	0.012383	0.012383	0.085654211	0.089578807	0.00196229
16.17923	0.011225	0.011225	0.086526724	0.091022718	0.00224799

Time /h	Concentration (M)				
	**13a**	**14a**	**15a**	**H_2O**	**17a**
17.88278	0.010262	0.010262	0.087201614	0.092274105	0.00253623
21.28989	0.008755	0.008755	0.088126399	0.094362764	0.00311817
22.99344	0.008157	0.008157	0.08843194	0.095254091	0.00341106
25.2	0.007494	0.007494	0.08871383	0.096297299	0.00379172

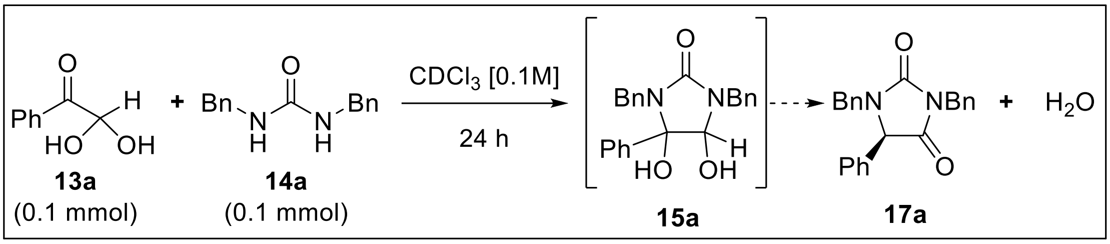

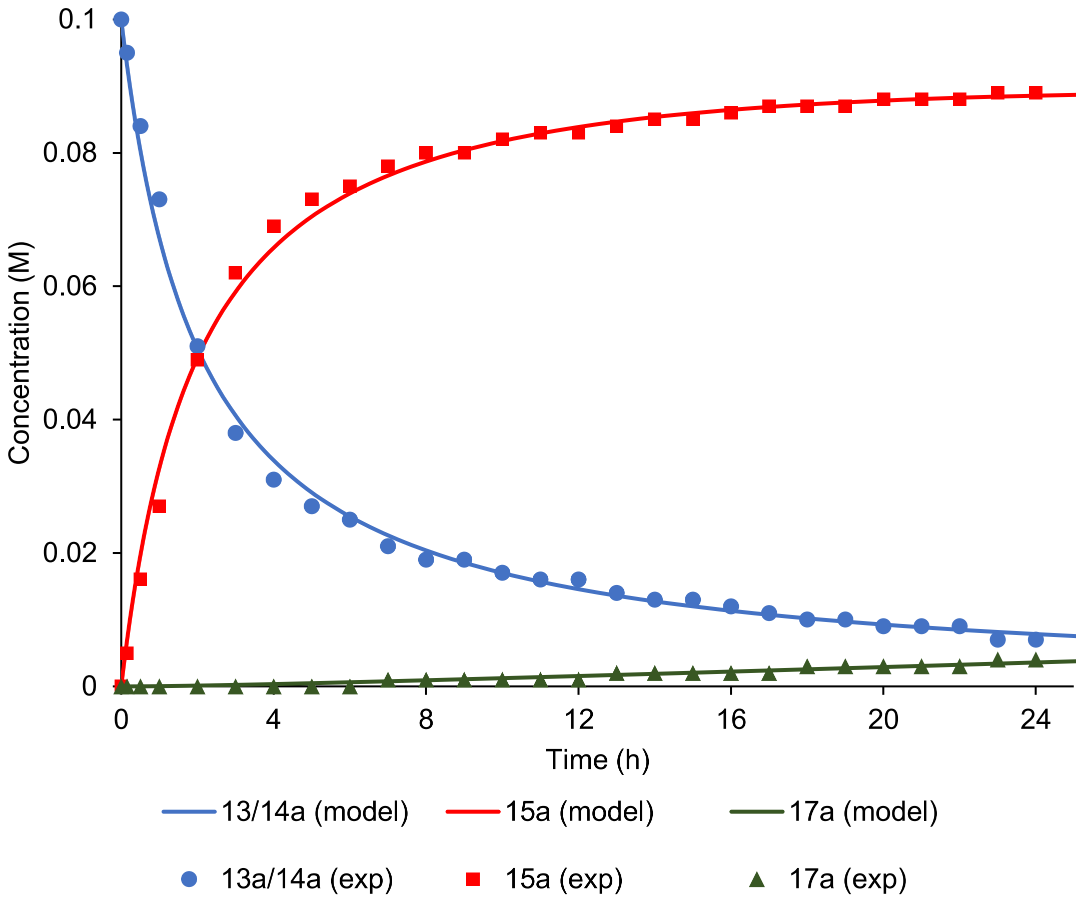

Figure S10: Experimental vs model concentration plot against time for the reaction in the absence of catalyst in $CDCl_3$.

Table S12: Experimental concentration results against time for **20a**-catalysed reaction in $CDCl_3$.

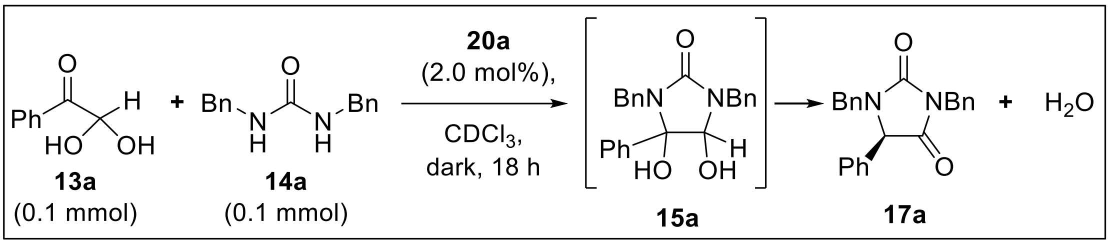

Time /h	Concentration [M]				
	**13a**	**14a**	**15a**	**H_2O**	**17a**
0	0.1	0.1	0		0
0.25	0.025	0.025	0.073		0.002
0.5	0.012	0.012	0.084		0.004
1	0.006	0.006	0.086		0.008
2	0.003	0.003	0.08		0.017
3	0.001	0.001	0.074		0.025
4	0.0005	0.0005	0.0695		0.03
5	0	0	0.064		0.036
6	0	0	0.057		0.043
7	0	0	0.051		0.049
8	0	0	0.042		0.058
9	0	0	0.035		0.065
10	0	0	0.029		0.071
11	0	0	0.024		0.076
12	0	0	0.018		0.082
13	0	0	0.014		0.086
14	0	0	0.01		0.09
15	0	0	0.007		0.093
16	0	0	0.005		0.095
17	0	0	0.003		0.097
18	0	0	0		0.099

		**value**	**± error**
**13+14a→15a+ H_2O**	k_1	150	15 M^{-1} h^{-1}
**15a^0→17a+H_2O**	k_2	0.00703	0.00015 h^{-1}
	SSE	1.80E-04	M

Table S13: Model-based concentration results against time for **20a**-catalysed reaction in $CDCl_3$.

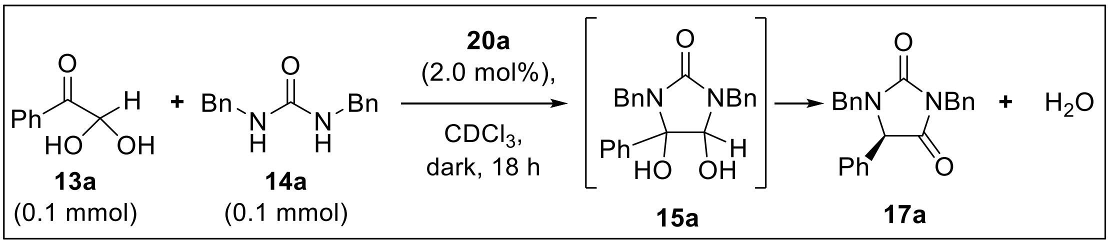

Time /h	Concentration				
	**13a**	**14a**	**15a**	**H_2O**	**17a**
0	0.1	0.1	0	0	0
0.000169	0.099748	0.099748	0.000250908	0.000252908	1.00007E-06
0.000337	0.099497	0.099497	0.000500426	0.000504738	2.15627E-06
0.000506	0.099248	0.099248	0.000748668	0.000755342	3.33684E-06
0.000919	0.098642	0.098642	0.001352147	0.001364613	6.23296E-06
0.001332	0.098042	0.098042	0.001948542	0.001966818	9.13807E-06
0.001745	0.09745	0.09745	0.002537784	0.00256187	1.2043E-05
0.002159	0.096865	0.096865	0.003119929	0.003149823	1.49468E-05
0.004524	0.093644	0.093644	0.006324893	0.006388034	3.15707E-05
0.006889	0.090629	0.090629	0.009322967	0.009419349	4.8191E-05
0.009255	0.087803	0.087803	0.012132579	0.012262201	6.4811E-05
0.01162	0.085148	0.085148	0.014770889	0.014933751	8.14311E-05
0.017181	0.079503	0.079503	0.020376409	0.020617423	0.000120507
0.022742	0.074563	0.074563	0.025277591	0.025596757	0.000159583
0.028303	0.070199	0.070199	0.029602606	0.029999925	0.000198659
0.033864	0.066315	0.066315	0.033447084	0.033922554	0.000237735
0.039425	0.062838	0.062838	0.03688503	0.037438653	0.000276812
0.048866	0.057711	0.057711	0.041946088	0.042632382	0.000343147
0.058306	0.053359	0.053359	0.046231897	0.047050861	0.000409482
0.067747	0.049614	0.049614	0.049910126	0.05086176	0.000475817
0.077187	0.046358	0.046358	0.053099628	0.054183933	0.000542152
0.086628	0.043501	0.043501	0.055890812	0.057107787	0.000608488
0.10247	0.039433	0.039433	0.059847653	0.061287256	0.000719802
0.118311	0.036061	0.036061	0.063108114	0.064770346	0.000831116
0.134153	0.033216	0.033216	0.065841293	0.067726153	0.00094243
0.149995	0.030785	0.030785	0.068161145	0.070268634	0.001053745
0.165836	0.028685	0.028685	0.070149629	0.072479747	0.001165059
0.189138	0.026075	0.026075	0.07259653	0.075254114	0.001328792
0.21244	0.023898	0.023898	0.074609165	0.077594217	0.001492526
0.235741	0.022054	0.022054	0.076289796	0.079602315	0.001656259
0.259043	0.020472	0.020472	0.077707554	0.081347539	0.001819993
0.282345	0.019103	0.019103	0.078913132	0.082880585	0.001983726
0.317871	0.017339	0.017339	0.080428056	0.08489477	0.002233357
0.353397	0.01587	0.01587	0.081646997	0.086612973	0.002482988
0.388923	0.014627	0.014627	0.082640472	0.08810571	0.002732619
0.42445	0.013563	0.013563	0.083454491	0.08941899	0.00298225
0.514231	0.011463	0.011463	0.084923611	0.092149846	0.003613117
0.459976	0.012645	0.012645	0.084122929	0.09058669	0.00323188

Time /h	Concentration				
	13a	14a	15a	H_2O	17a
0.568487	0.01048	0.01048	0.085525283	0.093513991	0.003994354
0.622743	0.009651	0.009651	0.085973456	0.094724637	0.004375591
0.676998	0.008943	0.008943	0.086300492	0.095814146	0.004756827
0.731254	0.008332	0.008332	0.086530102	0.09680623	0.005138064
0.812506	0.007559	0.007559	0.086731889	0.098149884	0.005708997
0.893759	0.006916	0.006916	0.086803673	0.099363535	0.006279931
0.975011	0.006373	0.006373	0.08677574	0.100477469	0.006850864
1.056263	0.005908	0.005908	0.08666989	0.101513485	0.007421798
1.137516	0.005506	0.005506	0.086501186	0.102486648	0.007992731
1.25432	0.005017	0.005017	0.08616997	0.10379692	0.008813475
1.371124	0.004607	0.004607	0.085758627	0.105027065	0.009634219
1.487928	0.00426	0.00426	0.085285329	0.106195254	0.010454963
1.604732	0.003961	0.003961	0.084763309	0.107314722	0.011275706
1.721536	0.003701	0.003701	0.084202759	0.108395659	0.01209645
1.906856	0.003354	0.003354	0.083247276	0.11004454	0.013398632
2.092175	0.003068	0.003068	0.082230893	0.111632519	0.014700813
2.277495	0.002828	0.002828	0.081169489	0.113175479	0.016002995
2.462815	0.002621	0.002621	0.0800734	0.114683752	0.017305176
2.648135	0.002443	0.002443	0.078949509	0.116164225	0.018607358
2.905103	0.002233	0.002233	0.077353994	0.11817997	0.020412988
3.162071	0.002056	0.002056	0.075725053	0.120162288	0.022218617
3.419039	0.001905	0.001905	0.074070271	0.122118765	0.024024247
3.676007	0.001775	0.001775	0.072394961	0.124054715	0.025829877
3.932975	0.001661	0.001661	0.070703001	0.125974015	0.027635507
4.335436	0.00151	0.00151	0.068026177	0.128953111	0.030463467
4.737896	0.001384	0.001384	0.065324168	0.131907022	0.033291427
5.140357	0.001278	0.001278	0.062602923	0.134841697	0.036119387
5.542818	0.001186	0.001186	0.059866493	0.137761187	0.038947347
5.945279	0.001107	0.001107	0.057117831	0.140668445	0.041775307
6.554549	0.001005	0.001005	0.05293821	0.145051116	0.046056453
7.16382	0.000921	0.000921	0.048741577	0.149416775	0.050337599
7.773091	0.000849	0.000849	0.044531972	0.153769462	0.054618745
8.382362	0.000788	0.000788	0.040312112	0.158111894	0.058899891
8.991632	0.000735	0.000735	0.036083984	0.162446058	0.063181037
9.970175	0.000663	0.000663	0.029279612	0.169393486	0.070056937
10.94872	0.000604	0.000604	0.022462677	0.176328351	0.076932837
11.92726	0.000555	0.000555	0.015636314	0.183253787	0.083808737
12.6	0.000525	0.000525	0.010938833	0.18801054	0.088535853

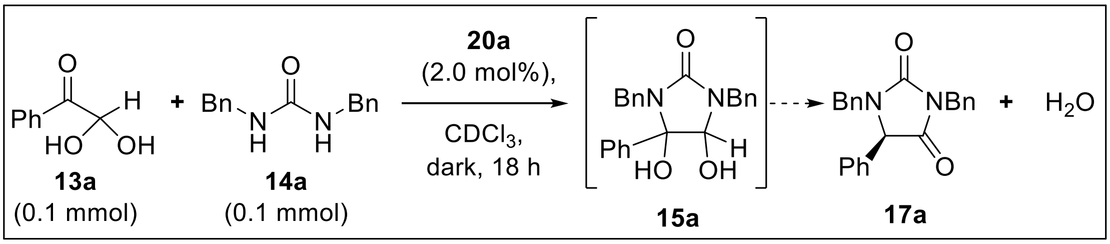

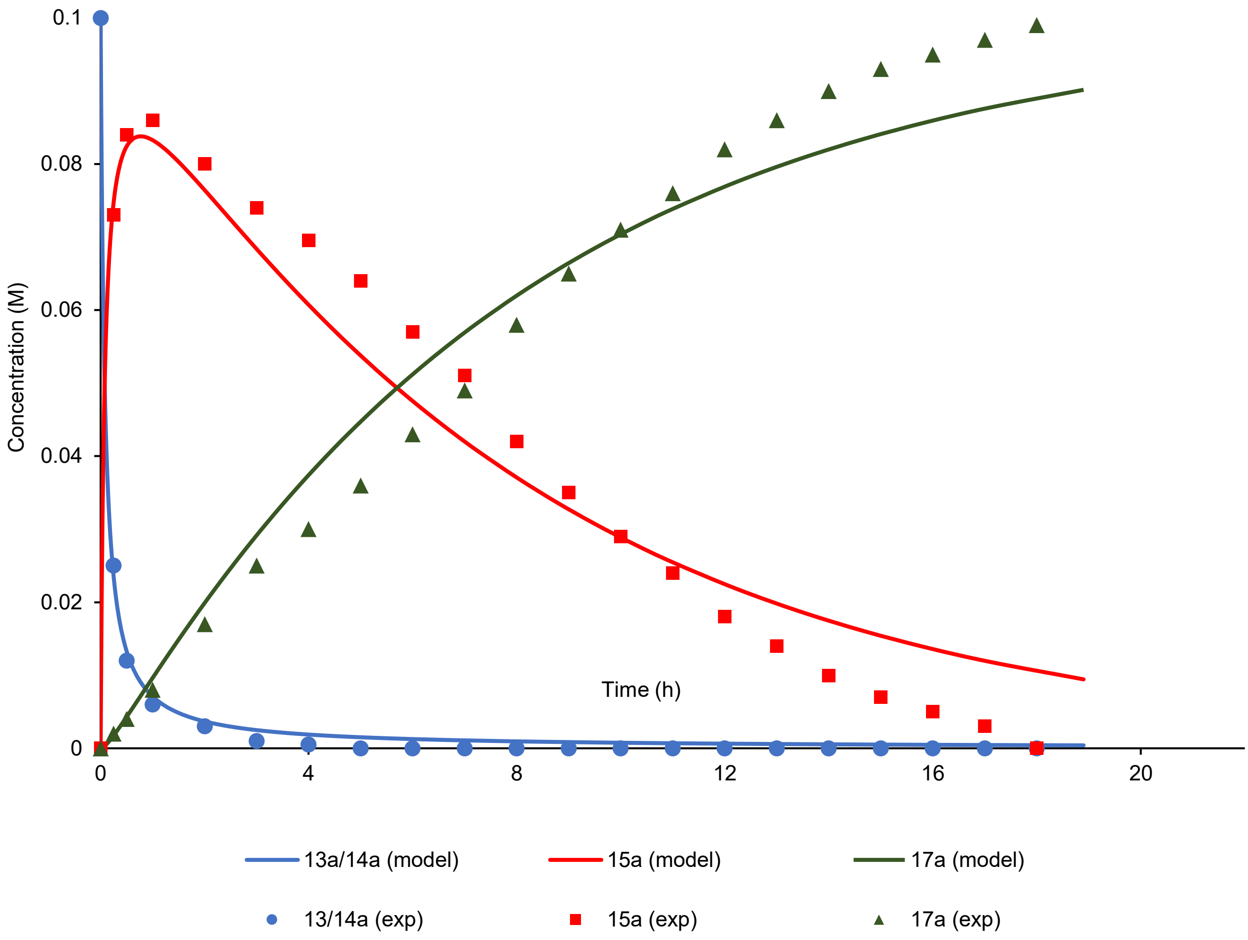

Figure S11: Experimental vs model-based concentration plot against time for **20a**-catalysed reaction in $CDCl_3$. The experimental data shows significant lack-of-fit to the model-based data.

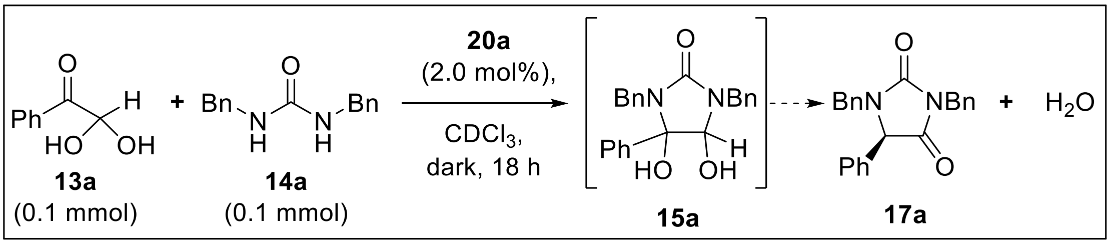

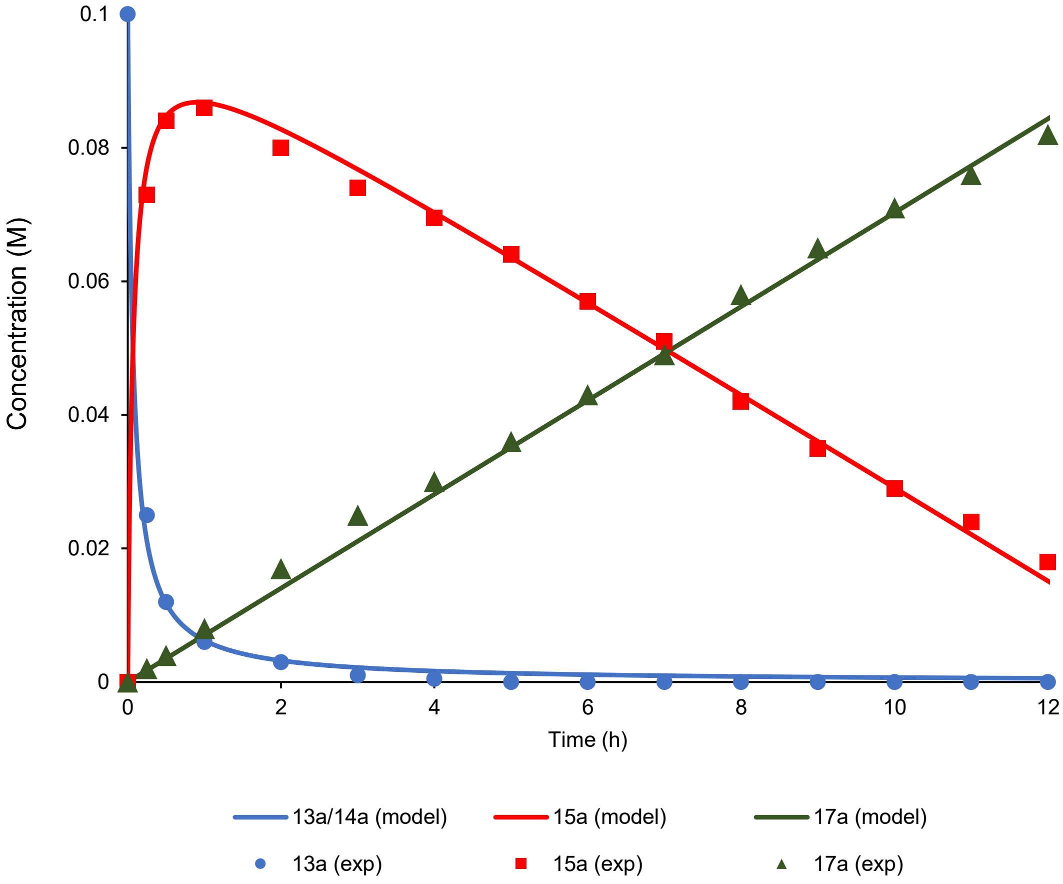

Figure S12: Experimental vs model-based concentration plot against time for **20a**-catalysed reaction in $CDCl_3$. Fitting kinetics between 0-12 h.

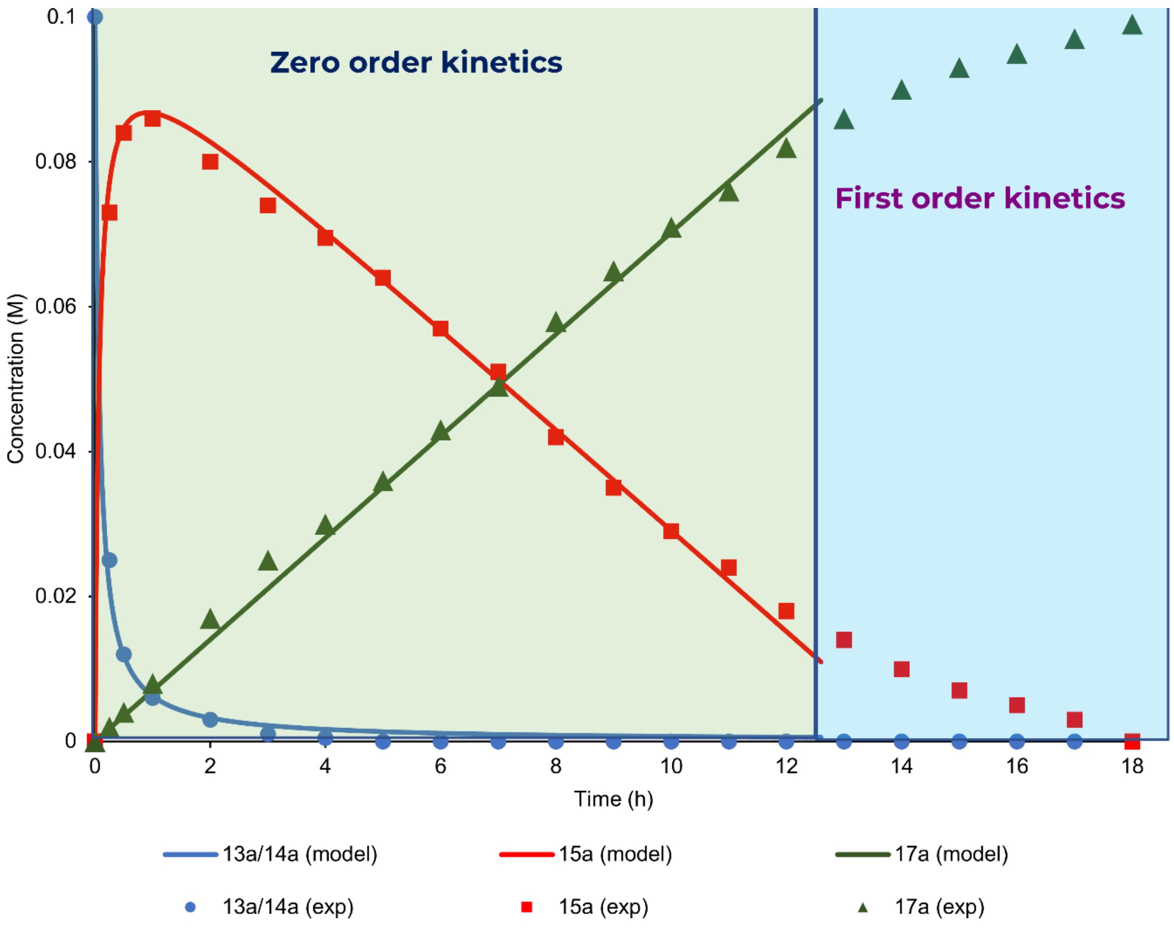

Figure S13: Extended experimental vs model-based concentration plot against time for 20a-catalysed reaction in $CDCl_3$. Near to the reaction completion, values are not fitted to zero order kinetics. The system approaches first order near to full conversion.

9.4 Variable time normalization analysis (VTNA)

Artificial starting time point of 0 h for when concentration of 15a is known.

Table S14: VTNA experimental concentration results against time for **20a**-catalysed reaction in $CDCl_3$.

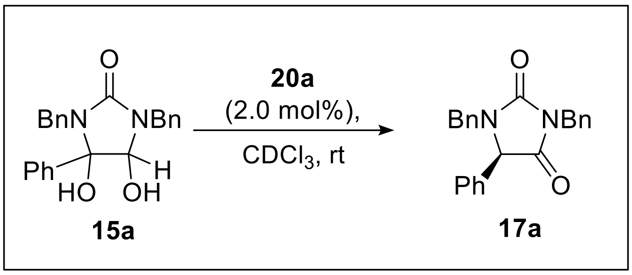

Actual experimental time /h	Artificial starting time /h	Concentration (M)		
		**15a**	**H_2O**	**17a**
5	0	0.064		0.036
6	1	0.057		0.043
7	2	0.051		0.049
8	3	0.042		0.058
9	4	0.035		0.065
10	5	0.029		0.071
11	6	0.024		0.076
12	7	0.018		0.082
13	8	0.014		0.086
14	9	0.01		0.09
15	10	0.007		0.093
16	11	0.005		0.095
17	12	0.003		0.097

		value	± error	
**15a^0→15a+ H_2O**	k_1	0.0068	0.0002 M h^{-1}	**zero order**
**15a→15a+ H_2O**	k_1	0.330	0.067 h^{-1}	**first order**

Table S15: VTNA model-based concentration results against time for **20a**-catalysed reaction in $CDCl_3$.

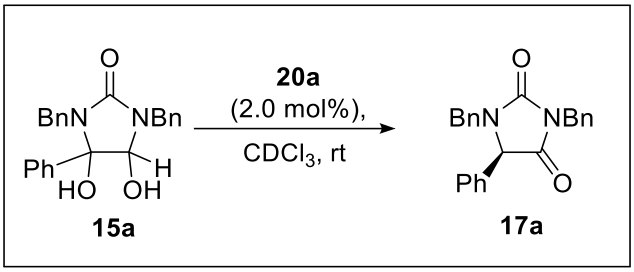

time /h	Concentration [M]		
	15a	H_2O	17a
0	0.064		0.036
0.735	0.059002923		0.0409971
1.47	0.054005846		0.0459942
2.205	0.049008769		0.0509912
2.94	0.044011692		0.0559883
3.675	0.039014615		0.0609854
4.41	0.034017538		0.0659825
5.145	0.029020461		0.0709795
5.88	0.024023384		0.0759766
6.615	0.019026307		0.0809737
7.108472	0.017374886		0.0826251
7.216944	0.016770952		0.083229
7.325416	0.016187926		0.0838121
7.882225	0.013472621		0.0865274
8.439034	0.011202064		0.0887979
8.995843	0.009312197		0.0906878
9.444527	0.008026941		0.0919731
9.893211	0.00691924		0.0930808
10.3419	0.005964324		0.0940357
10.79058	0.005141138		0.0948589
11.42058	0.004175624		0.0958244
12.05058	0.003392874		0.0966071
12.68058	0.002757151		0.0972428

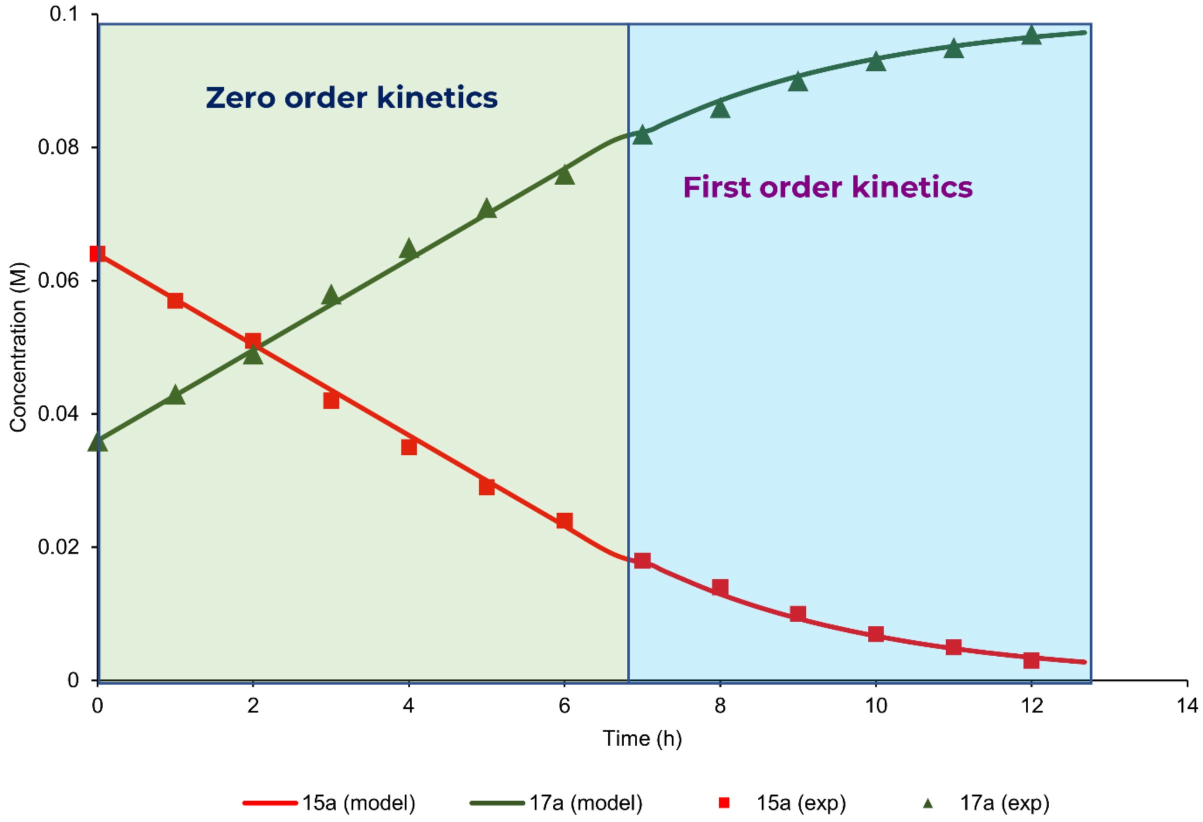

Figure S14: VTNA experimental vs model-based concentration results against time for **20a**-catalysed reaction in $CDCl_3$.

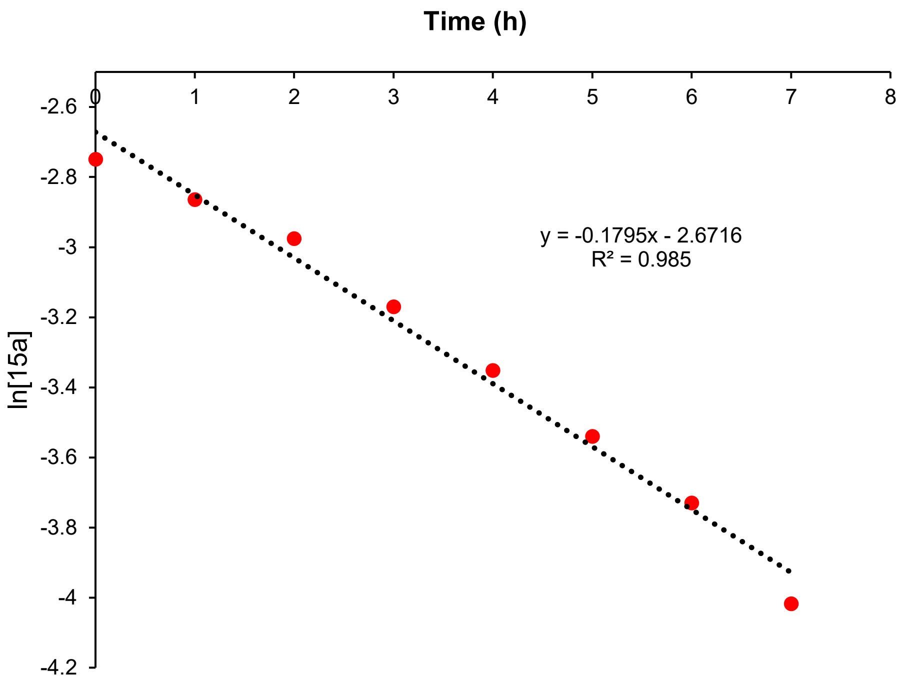

Figure S15: VTNA ln[**15a**] against time showing fit with first order kinetics and a rate constant k_2 = 0.180 h^{-1} with a coefficient of determination R^2= 0.985.

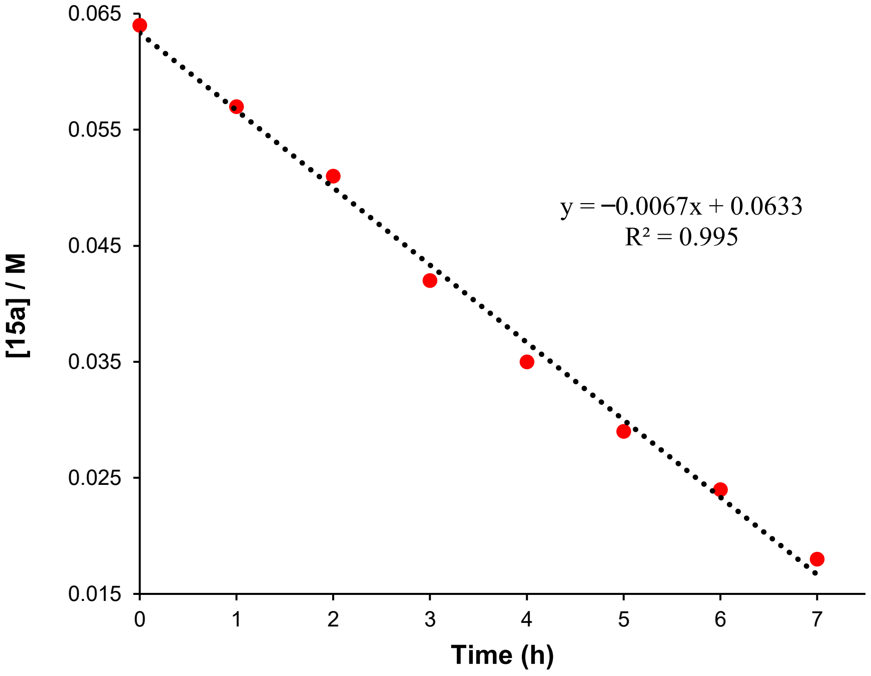

Figure S16: VTNA [**15a**] against time showing improved fit with zero order kinetics and a rate constant k_2 = 0.0067 M $h^{-1}$with a coefficient of determination R^2= 0.995.

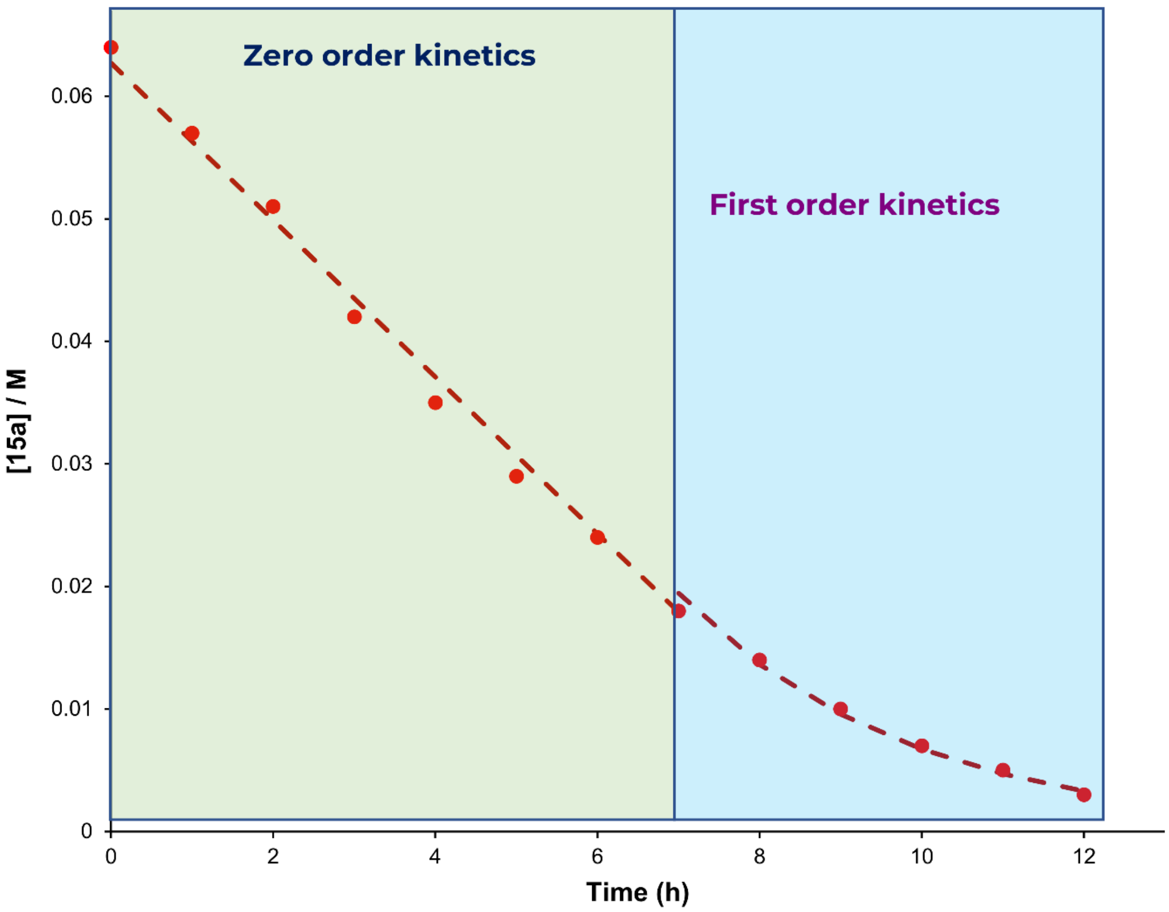

Figure S17: VTNA [**15a**] against time showing initial fit with zero order kinetics with a rate constant of k_2 = 0.0067 M h^{-1} and then switch to first order kinetics.

10. Arylglyoxal substrate scope for asymmetric hydantoin synthesis

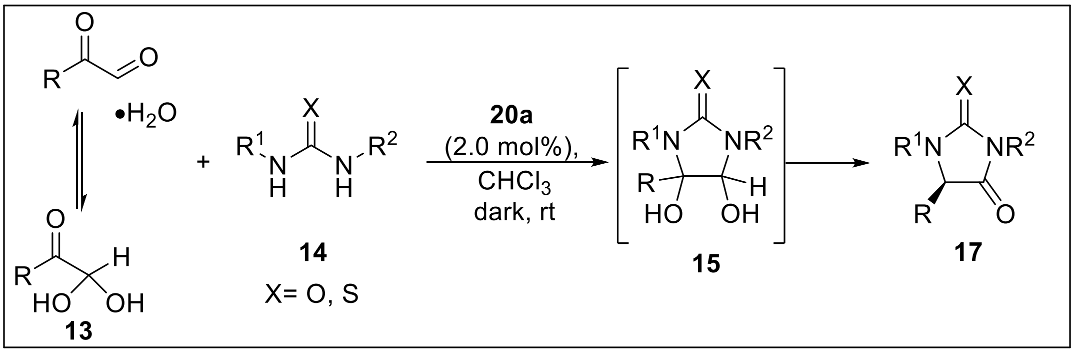

Note: *For glyoxals bearing electron-withdrawing aromatic substituents (e.g. 4-Cl; 4-Br etc.), careful TLC monitoring (sample acquired through a needle, under Ar) still introduced enough oxygen to form traces of the unwanted 5-hydroxyhydantoin* ***21****. To achieve full conversion to the target hydantoin* **17***, in the absence of the 5-hydroxyhydantoin* ***21****, TLC monitoring must be avoided, and reactions should be run for the times we have indicated for the specific substrates.*

General procedure A:

To an oven dried NMR tube* charged with urea **14** (100 µmol, 1.0 eq.) and catalyst **20a** (1.4 mg, 2.0 µmol, 2.0 mol%) was added aryl / alkylglyoxal† **13** (100 µmol, 1.0 eq.) dissolved in $CHCl_3$ (0.1 M, 1.0 mL)‡. The reaction tube headspace was briefly purged with Ar before inserting a cap. The cap was additionally sealed with a parafilm, the tube was wrapped with aluminium foil, and stood at rt without stirring. Every 20 h, under an Ar balloon, a crude sample (1-2 drops) was withdrawn, concentrated *in vacuo* at rt, and the crude conversion of the starting materials **13-14**, to the intermediate diol **15** and product **17** was monitored by ^1H NMR spectroscopy at 600 MHz using an NMR capillary tube§. Following complete disappearance of starting materials **13-14**, and the intermediate diol **15**, the reaction mixture was concentrated *in vacuo* at rt and purified by flash chromatography using the eluent specified. The isolated product was diluted in the solvent specified (MeCN for CHIRALPAK® AS-3R, or EtOH for Astec® Cellulose DMP) before eluting on the chiral HPLC.

* Wilmad® NMR tubes 5 mm diam., precision frequency 300 MHz, L 7 in., purchased from Sigma-Aldrich (Wilmad® Z271993).
† mmol estimated based on the molecular weight of the monohydrate or hemihydrate form or glyoxal form (as described).
‡ $CHCl_3$ was neutralised over K_2CO_3 and degassed under a stream of Ar at 0 °C prior to setting up the reaction.
§ Wilmad® NMR capillary tubes short, O.D. × L 2.5 mm × 100 mm.

General procedure B – scaled up reactions:

An oven dried 7 mL glass vial (Supelco® 27151) fitted with a Teflon coated stirred bar was charged with arylglyoxal* **13** (0.50 mol, 1.0 eq.), 1,3-dibenzyl urea **14a** (0.50 mol, 1.0 eq.), and catalyst **20a** (7.1 mg, 10 µmol, 2.0 mol%). The reactants were dissolved in $CHCl_3$ (5.0 mL, 0.1 M)† and the reaction headspace was purged with Ar for 2-3 min. The lid was additionally sealed with 5-7 layers parafilm, wrapped with aluminium foil and stirred for the time specified at rt. The reaction mixture was concentrated *in vacuo* at rt and purified by flash chromatography using the eluents specified. The isolated product was diluted in the solvent specified (MeCN for CHIRALPAK® AS-3R, or EtOH for Astec® Cellulose DMP) before eluting on the chiral HPLC.

* mmol estimated based on the molecular weight of the monohydrate or hemihydrate form or glyoxal form (as described).

† $CHCl_3$ was neutralised over K_2CO_3 and degassed under a stream of Ar at 0 °C prior to setting up the reaction.

1,3-Dibenzyl-5-phenylimidazolidine-2,4-dione 17a

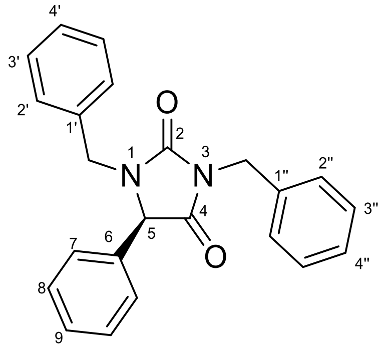

General procedure **A** was followed using phenylglyoxal monohydrate **13a*** (15 mg, 99 µmol, 1.0 eq.) and 1,3-dibenzylurea **14a** (24 mg, 101 µmol, 1.0 eq.) for 20 h. Flash chromatography eluting with 90:10 hexane–EtOAc gave the title compound **17a** (35 mg, 99 µmol, 99%, 95:5 e.r.) as a colourless amorphous solid. The product was recrystallised in 50:50 hexane–EtOH (1.0 mL) to give compound **17a** (17 mg, 48 µmol, 48%, >99: trace e.r.) as colourless needles.

General procedure **B** was followed using phenylglyoxal monohydrate **13a** (76 mg, 0.50 mmol, 1.0 eq.) 1,3-dibenzyl urea **14a** and (120 mg 0.50 mmol, 1.0 eq.) for 20 h. Flash chromatography eluting with 90:10 petrol–EtOAc gave the title compound **17a** (175 mg, 0.49 mmol, 98%, 94:6 e.r.) as a colourless amorphous solid.

R*f* 0.21 (90:10 hexane–EtOAc).

^{1}H NMR (600 MHz, $CDCl_3$): δ 7.42-7.39 (2H, m, 2"-H) 7.39-7.37 (3H, m, 8-H and 9-H), 7.35-7.32 (2H, m, 4'-H and 4"-H) 7.31-7.26 (4H, m, 3-H' and 3"-H), 7.17-7.05 (4H, m, 7-H and 2'-H), 5.14 (1H, d, *J* 15.0, 1-NCH_AH$_B$Ph), 4.78 (1H, d, *J* 14.5, 3-NCH_AH$_B$Ph), 4.68 (1H, d, *J* 14.5, 3-NCH$_A$$H_B$Ph), 4.66 (1H, s, 5-H) 3.71 (1H, d, *J* 15.0, 1-NCH$_A$$H_B$Ph).

^{13}C NMR (100 MHz, $CDCl_3$, 1×ArC-H not observed): δ 171.1 (4-C), 156.4 (2-C), 136.2 (1"-C), 135.4 (1'-C), 132.5 (6-C), 129.4 (8-C), 129.0 (3'-C or 3"-C), 128.8 (3'-C or 3"-C), 128.6 (2'-C or 2"-C), 128.5 (2'-C or 2"-C), 128.2 (4'-C), 128.0 (4"-C or 9-C), 127.7 (7-C), 63.1 (5-C), 44.6 (1-N*C*H_2Ph), 42.9 (3-N*C*H_2Ph).

IR ν_{max}(neat)/cm^{-1} 3025, 1758 (C=O), 1693 (C=O), 1432, 1334, 1250, 1129.

HRMS (ESI): $C_{23}H_{21}N_2O_2$ $[M+H]^+$; calculated 357.1598, found 357.1595.

$[\alpha]_D^{21}$ = −34 (c. 0.1, $CHCl_3$, 95:5 e.r.).

HPLC (Astec® Cellulose DMP, 94:6 hexane–EtOH, 1 mL/min flow rate, 254 nm): t_r(e_1, major) = 14.8 min; t_r(e_2, minor) = 20.8 min.

* Vendor: Alfa Aesar.

M.p: 93-95 °C.

The spectral data are consistent with the literature values.[8]

X-ray crystallography: **Crystal Data** for $C_{23}H_{20}N_2O_2$ (M =356.41 g/mol): monoclinic, space group $P2_1$ (no. 4), a = 12.0926(2) Å, b = 5.4361(2) Å, c = 13.6728(3) Å, β = 95.641(2)°, V = 894.45(4) $Å^3$, Z = 2, T = 120.0(2) K, μ(Cu Kα) = 0.679 mm^{-1}, $Dcalc$ = 1.323 g/cm^3, 15907 reflections measured (7.346° ≤ 2Θ ≤ 148.93°), 3500 unique (R_{int} = 0.0890, R_{sigma} = 0.0566) which were used in all calculations. The final R_1 was 0.0550 (I > 2σ(I)) and wR_2 was 0.1526 (all data). CCDC 2235706 contains the supplementary crystallographic data for this compound. Crystals were grown by slow evaporation from hexane–EtOH.

1,3-Dibenzyl-5-(4-methylphenyl)imidazolidine-2,4-dione 17b

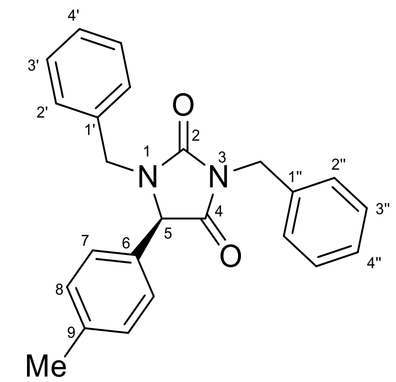

General procedure **A** was followed starting with 4-methylphenylglyoxal monohydrate **13b** (17 mg, 102 µmol,* 1.0 eq.) and 1,3-dibenzylurea **14a** (24 mg, 101 µmol, 1.0 eq.) for 20 h. Flash chromatography eluting with 90:10 hexane–EtOAc gave the *title compound* **17b** (36 mg, 97 µmol, 97%, 92:8 e.r.) as a colourless amorphous solid.

General procedure **B** was followed using phenylglyoxal monohydrate **13b** (83 mg, 0.50 mmol, 1.0 eq.) 1,3-dibenzyl urea **14a** and (120 mg 0.50 mmol, 1.0 eq.) for 20 h. Flash chromatography eluting with 90:10 petrol–EtOAc gave the *title compound* **17b** (174 mg, 0.47 mmol, 94%, 92:8 e.r.) as a colourless amorphous solid.

R*f* 0.27 (90:10 hexane–EtOAc).

^{1}H NMR (600 MHz, $CDCl_3$): δ 7.41 (2H, app. dd, *J* 8.1, 1.1, 2''-H), 7.35-7.32 (2H, m, 4'-H and 4''-H), 7.31-7.28 (4H, m, 3'-H and 3''-H), 7.19 (2H, d, *J* 8.0, 8-H), 7.12 (2H, app. dd, *J* 7.4, 2.1, 2'-H), 7.01 (2H, d, *J* 8.0, 7-H), 5.14 (1H, d, *J* 15.0, 1-NCH_AH$_B$Ph), 4.78 (1H, d, *J* 14.5, 3-NCH_AH$_B$Ph), 4.68 (1H, d, *J* 14.5, 3-NCH$_A$$H_B$Ph), 4.63 (1H, s, 5-H), 3.69 (1H, d, *J* 15.0, 1-NCH$_A$$H_B$Ph), 2.36 (3H, s, PhC$H_3$).

^{13}C NMR (150 MHz, $CDCl_3$): δ 171.4 (4-C), 156.5 (2-C), 139.5 (9-C), 136.3 (1''-C), 135.5 (1'-C) 130.2 (8-C), 129.5 (6-C), 129.1 (3''-C), 128.8 (3'-C), 128.6 (2'-C or 2''-C), 128.6 (2'-C or 2''-C), 128.2 (4'-C), 128.0 (4''-C), 127.6 (7-C), 62.9 (5-C), 44.5 (1-N*C*H_2Ph), 42.9 (3-N*C*H_2Ph), 21.4 (Ph*C*H_3)

IR ν_{max}(film from $CDCl_3$)/cm^{-1} 3031, 1769 (C=O), 1706 (C=O), 1441, 1417, 1336, 1078.

HRMS (ESI): $C_{24}H_{23}N_2O_2$ [M+H]$^+$; calculated 371.1754, found 371.1757.

$[\alpha]_D^{21}$ = −42 (c. 0.1, $CHCl_3$, 92:8 e.r.).

HPLC (CHIRALPAK® AS-3R, 60:40 MeCN–H_2O, 0.5 mL/min flow rate, 220 nm); t_r(e_1, major) = 12.4 min; t_r(e_2, minor) = 13.5 min.

* mmol estimated based on the molecular weight of the monohydrate form of the glyoxal.

1,3-Dibenzyl-5-(4-hydroxyphenyl)imidazolidine-2,4-dione 17c

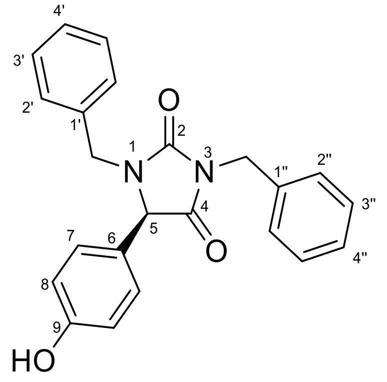

General procedure **A** was followed starting with 4-hydroxyphenylglyoxal monohydrate **13c** (17 mg, 101 µmol,* 1.0 eq.) and 1,3-dibenzylurea **14a** (24 mg, 101 µmol, 1.0 eq.) for 20 h. Flash chromatography eluting with 80:20 hexane–EtOAc gave the *title compound* **17c** (36 mg, 97 µmol, 97%, 94:6 e.r.) as a colourless amorphous solid. The product was recrystallised in 50:50 hexane–EtOH (1.0 mL) to give compound **17c** (21 mg, 57 µmol, 57%, >99:trace e.r.) as colourless needles.

R*f* 0.23 (80:20 hexane–EtOAc)

^{1}H NMR (600 MHz, $CDCl_3$, O*H* not observed): δ 7.44-7.40 (2H, m, 2''-H), 7.37-7.33 (2H, m, 4'-H and 4''-H), 7.32-7.28 (4H, m, 3'-H and 3''-H), 7.14-7.06 (2H, m, 2'-H), 6.88-6.82 (2H, m, 7-H), 6.65-6.59 (2H, m, 8-H), 5.09 (1H, d, *J* 15.0, 1-NCH_AH$_B$Ph), 4.78 (1H, d, *J* 14.5, 3-NCH_AH$_B$Ph), 4.68 (1H, d, *J* 14.5, 3-NCH$_A$$H_B$Ph), 4.59 (1H, s, 5-H), 3.67 (1H, d, *J* 15.0, 1-NCH$_A$$H_B$Ph).

^{13}C NMR (150 MHz, $CDCl_3$): δ 172.2 (4-C), 156.9 (9-C), 156.2 (2-C), 136.1 (1''-C), 135.4 (1'-C), 129.2 (7-C), 129.0 (3'-C or 3''-C), 128.9 (3'-C or 3''-C), 128.7 (2'-C or 2''-C), 128.6 (2'-C or 2''-C), 128.2 (4'-C), 128.1 (4''-C), 123.8 (6-C), 116.4 (8-C), 62.7 (5-C), 44.5 (1-N*C*H_2Ph), 42.9 (3-N*C*H_2Ph).

IR ν_{max}(neat)/cm^{-1} 3364 (OH), 1764 (C=O), 1694 (C=O), 1517, 1448, 1414, 1225.

HRMS (ESI): $C_{23}H_{21}N_2O_3$ $[M+H]^+$; calculated 373.1547, found 373.1554.

$[\alpha]_D^{21}$ = −32 (c. 0.1, $CHCl_3$, 94:6 e.r.).

HPLC (Astec® Cellulose DMP, 80:20 hexane–EtOH, 1 mL/min flow rate, 254 nm): t_r(e_1, major) = 12.5 min; t_r(e_2, minor) = 16.8 min.

* mmol estimated based on the molecular weight of the monohydrate form of the glyoxal.

1,3-Dibenzyl-5-(4-methoxyphenyl)imidazolidine-2,4-dione 17d

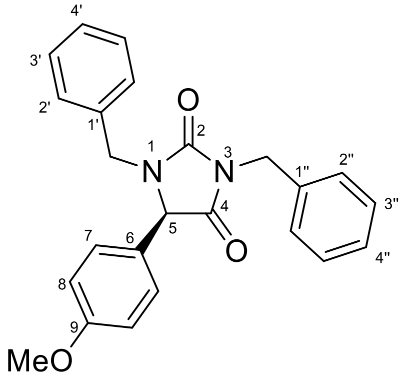

General procedure **A** was followed starting with 4-methoxyphenylglyoxal monohydrate **13d*** (18 mg, 99 μmol, 1.0 eq.) and 1,3-dibenzylurea **14a** (24 mg, 100 μmol, 1.0 eq.) for 20 h. Flash chromatography eluting with 90:10 hexane–EtOAc gave the *title compound* **17d** (38 mg, 97 μmol, 97%, 98:2 e.r.) as a colourless amorphous solid.

General procedure **B** was followed using 4-methoxyphenylglyoxal monohydrate **13d** (93 mg, 0.50 mmol, 1.0 eq.) 1,3-dibenzyl urea **14a** and (120 mg 0.50 mmol, 1.0 eq.) for 20 h. Flash chromatography eluting with 90:10 petrol–EtOAc gave the *title compound* **17d** (187 mg, 0.48 mmol, 97%, 97:3 e.r.) as a colourless amorphous solid.

$\mathbf{R}_f$ 0.20 (90:10 hexane–EtOAc).

^{1}H NMR (600 MHz, $CDCl_3$): δ 7.43-7.38 (2H, m, 2"-H), 7.36-7.32 (2H, m, 4'-H and 4"-H), 7.32-7.26 (4H, m, 3'-H and 3"-H), 7.14-7.07 (2H, m, 2'-H), 7.06-6.99 (2H, m, 7-H), 6.94-6.86 (2H, m, 8-H), 5.12 (1H, d, *J* 15.0, 1-NCH_AH$_B$Ph), 4.79 (1H, d, *J* 14.5, 3-NCH_AH$_B$Ph), 4.68 (1H, d, *J* 14.5, 3-NCH$_A$$H_B$Ph), 4.61 (1H, s, 5-H), 3.81 (3H, s, OCH_3), 3.69 (1H, d, *J* 15.0, 1-NCH$_A$$H_B$Ph).

^{13}C NMR (150 MHz, $CDCl_3$, 2×Ar-C not observed): δ 171.8 (4-C), 160.7 (9-C), 156.6 (2-C), 136.5 (1"-C), 135.8 (1'-C), 129.1 (7-C), 128.8 (3'-C or 3"-C), 128.6 (2'-C or 2"-C), 128.3 (4'-C), 128.0 (4"-C), 124.4 (6-C), 115.0 (8-C), 62.9 (5-C), 55.7 (OCH_3), 44.7 (1-N*C*H_2Ph), 43.1 (3-N*C*H_2Ph).

IR ν_{max}(film from $CDCl_3$)/cm^{-1} 2932, 1769 (C=O), 1707 (C=O), 1512, 1442, 1418, 1304.

HRMS (ESI): $C_{24}H_{23}N_2O_2$ $[M+H]^+$; calculated 387.1703, found 387.1723.

$[\alpha]_D^{21}$ = −41 (c. 0.2, $CHCl_3$, 98:2 e.r.).

HPLC (Astec® Cellulose DMP, 80:20 hexane–*n*-PrOH, 1 mL/min flow rate, 220 nm): t_r(e_1, major) = 12.5 min; t_r(e_2, minor) = 21.6 min.

* Vendor: Alfa Aesar.

1,3-Dibenzyl-5-(4-phenoxyphenyl)imidazolidine-2,4-dione 17e

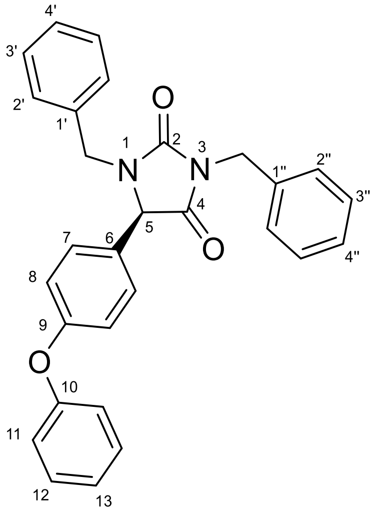

General procedure **A** was followed starting with 4-phenoxyphenylglyoxal monohydrate **13e*** (25 mg, 102 µmol, 1.0 eq.) and 1,3-dibenzylurea **14a** (24 mg, 100 µmol, 1.0 eq.) for 20 h. Flash chromatography eluting with 90:10 hexane–EtOAc gave the *title compound* **17e** (43 mg, 98 µmol, 98%, 90:10 e.r.) as a viscous colourless oil.

$\mathbf{R}_f$ 0.18 (90:10 hexane–EtOAc).

^{1}H NMR (600 MHz, $CDCl_3$): δ 7.42-7.39 (2H, m, 2"-H), 7.38-7.35 (2H, m, 11-H), 7.34-7.27 (6H, m, 4'-H, 4"-H, 3'-H and 3"-H), 7.15 (1H, t, *J* 7.4, 13-H), 7.13-7.11 (2H, m, 2'-H), 7.05 (2H, d, *J* 8.6, 7-H), 7.03 (2H, dd, *J* 8.7, 1.1, 12-H), 6.97 (2H, d, *J* 8.6, 8-H), 5.11 (1H, d, *J* 15.0, 1-NCH_AH$_B$Ph), 4.78 (1H, d, *J* 14.5, 3-NCH_AH$_B$Ph), 4.68 (1H, d, *J* 14.5, 3-NCH$_A$$H_B$Ph), 4.64 (1H, s, 5-H), 3.75 (1H, d, *J* 15.0, 1-NCH$_A$$H_B$Ph).

^{13}C NMR (150 MHz, $CDCl_3$, 1×Ar-C_q not observed): δ 171.4 (4-C), 158.6 (10-C or 9-C), 156.4 (2-C, 10-C or 9-C), 136.2 (1"-C), 135.5 (1'-C), 130.1 (11-C), 129.3 (7-C), 129.1 (2'-C or 2"-C), 128.9 (2'-C or 2"-C), 128.7 (3'-C or 3"-C), 128.6 (3'-C or 3"-C), 128.3 (4'-C), 128.1 (4"-C), 126.9 (6-C), 124.1 (13-C), 119.7 (12-C), 119.1 (8-C), 62.6 (5-C), 44.7 (1-NCH$_2$Ph), 43.0 (3-NCH$_2$Ph).

IR ν_{max}(film from $CDCl_3$)/cm^{-1} 3031, 1769 (C=O), 1707 (C=O), 1587, 1486, 1417, 1236.

HRMS (ESI): $C_{29}H_{25}N_2O_3$ $[M+H]^+$; calculated 449.1860, found 449.1865.

$[\alpha]_D^{21}$ = −44 (c. 0.2, $CHCl_3$, 90:10 e.r.).

HPLC (Astec® Cellulose DMP, 80:20 hexane– *n*-PrOH, 1 mL/min flow rate, 254 nm): t_r(e_1, major) = 18.6 min; t_r(e_2, minor) = 20.9 min.

* Vendor: AK Scientific.

1,3-Dibenzyl-5-(4-fluorophenyl)imidazolidine-2,4-dione 17f

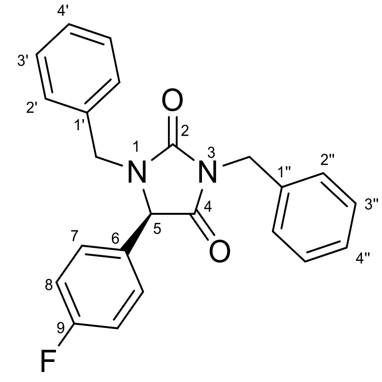

General procedure **A** was followed using 4-fluorophenylglyoxal monohydrate **13f*** (17 mg, 99 µmol, 1.0 eq.) and 1,3-dibenzylurea **14a** (24 mg, 100 µmol, 1.0 eq.) for 40 h. Flash chromatography eluting with 90:10 hexane–EtOAc gave the *title compound* **17f** (36 mg, 96 µmol, 96%, 91:9 e.r.) as a colourless amorphous solid.

General procedure **B** was followed using 4-fluorophenylphenylglyoxal monohydrate **13f** (85 mg, 0.50 mmol, 1.0 eq.) 1,3-dibenzyl urea **14a** and (120 mg, 0.50 mmol, 1.0 eq.) for 40 h. Flash chromatography eluting with 90:10 petrol–EtOAc gave the *title compound* **17f** (178 mg, 0.47 mmol, 95%, 91:9 e.r.) as a colourless amorphous solid.

$\mathbf{R}_f$ 0.17 (90:10 hexane–EtOAc).

^{1}H NMR (600 MHz, $CDCl_3$): δ 7.41-7.38 (2H, m, 2''-H), 7.35-7.32 (2H, m 4'-H and 4''-H), 7.31-7.28 (4H, m, 3'-H and 3''-H), 7.13-7.05 (6H, m, 7-H, 8-H and 2'-H), 5.13 (1H, d, *J* 15.0, 1-NCH_AH$_B$Ph), 4.77 (1H, d, *J* 14.5, 3-NCH_AH$_B$Ph), 4.68 (1H, d, *J* 14.5, 3-NCH$_A$$H_B$Ph), 4.64 (1H, s, 5-H), 3.71 (1H, d, *J* 15.0, 1-NCH$_A$$H_B$Ph).

^{13}C NMR (150 MHz, $CDCl_3$, 1×Ar-C not observed): δ 171.0 (4-C), 163.4 (d, $J_{C\text{-}F}$ 248.5, 9-C), 156.4 (2-C), 136.2 (1''-C), 135.3 (1'-C), 129.5 (d, $^3J_{C\text{-}F}$ 8.1, 7-C), 129.1 (Ar-C), 128.9 (Ar-C), 128.6 (Ar-C), 128.5 (6-C), 128.3 (4'-C), 128.1 (4''-C), 116.59 (d, $^2J_{C\text{-}F}$ 21.7, 8-C), 62.4 (5-C), 44.8 (1-NCH$_2$Ph), 43.0 (3-NCH$_2$Ph).

^{19}F NMR (565 MHz, $CDCl_3$): δ −111.6.

IR ν_{max}(film from $CDCl_3$)/cm^{-1} 3031, 1769 (C=O), 1706 (C=O), 1490, 1441, 1417, 1336.

HRMS (ESI): $C_{23}H_{20}FN_2O_2$ $[M+H]^+$; calculated 375.1503, found 375.1511.

$[\alpha]_D^{21}$ = −42 (c. 0.3, $CHCl_3$, 91:9 e.r.).

HPLC (Astec® Cellulose DMP, 80:20 hexane–*n*-PrOH, 1 mL/min flow rate, 254 nm): t_r(e_1, major) = 10.1 min; t_r(e_2, minor) = 12.3 min.

* Vendor: AK Scientific.

X-ray crystallography: **Crystal Data** for $C_{23}H_{19}N_2O_2F$ (M =374.40 g/mol): monoclinic, space group $P2_1$ (no. 4), a = 12.3101(2) Å, b = 5.45170(10) Å, c = 14.3867(2) Å, β = 106.621(2)°, V = 925.16(3) Å^3, Z = 2, T = 119.9(3) K, μ(Cu Kα) = 0.764 mm^{-1}, $Dcalc$ = 1.344 g/cm^3, 11231 reflections measured (7.494° ≤ 2Θ ≤ 134.126°), 3114 unique (R_{int} = 0.0762, R_{sigma} = 0.0570) which were used in all calculations. The final R_1 was 0.0731 (I > 2σ(I)) and wR_2 was 0.1834 (all data). CCDC 2235705 contains the supplementary crystallographic data for this compound. Crystals were grown by slow evaporation from hexane–EtOH

1,3-Dibenzyl-5-(4-chlorophenyl)imidazolidine-2,4-dione 17g

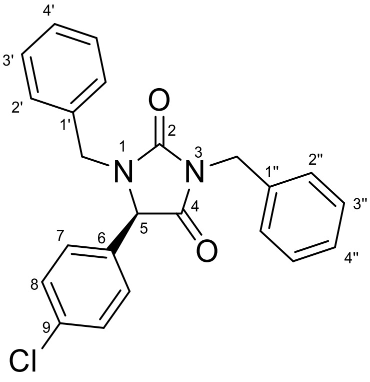

General procedure **A** was followed starting with 4-chlorophenylglyoxal monohydrate **13g** (19 mg, 100 µmol,* 1.0 eq.) and 1,3-dibenzylurea **14a** (24 mg, 100 µmol, 1.0 eq.) for 40 h. Flash chromatography eluting with 90:10 hexane–EtOAc gave the *title compound* **17g** (38 mg, 97 µmol, 97%, 91:9 e.r.) as a colourless amorphous solid.

$\mathbf{R}_f$ 0.24 (90:10 hexane–EtOAc).

^{1}**H NMR** (600 MHz, $CDCl_3$): δ 7.40-7.38 (2H, m, 2"-H), 7.36 (2H, d *J* 8.4, 8-H), 7.35-7.32 (2H, m, 4' and 4"-H), 7.32-7.28 (4H, m, 3' and 3"-H), 7.11-7.08 (2H, m, 2'-H), 7.06 (2H, d, *J* 8.4, 7-H), 5.15 (1H, d, *J* 15.0, 1-NCH_AH$_B$Ph), 4.77 (1H, d, *J* 14.5, 3-NCH_AH$_B$Ph), 4.67 (1H, d, *J* 14.5, 3-NCH$_A$$H_B$Ph), 4.62 (1H, s, 5-H), 3.71 (1H, d, *J* 15.0, 1-NCH$_A$$H_B$Ph).

^{13}**C NMR** (150 MHz, $CDCl_3$, 1×Ar-C not observed): δ 170.8 (4-C), 156.4 (2-C), 136.1 (1"-C), 135.5 (9-C), 135.2 (1'-C) 131.2 (6-C), 129.7 (8-C), 129.1 (3'-C or 3"-C), 129.0 (7-C), 128.9 (3'-C or 3"-C), 128.6 (2'-C or 2"-C), 128.4 (4'-C), 128.2 (4"-C), 62.4 (5-C), 44.8 (1-N*C*H_2Ph), 43.0 (3-N*C*H_2Ph).

IR ν_{max}(film from $CDCl_3$)/cm^{-1} 1771 (C=O), 1706 (C=O), 1509, 1442, 1416, 1226, 1097.

HRMS (ESI): $C_{23}H_{20}{}^{35}ClN_2O_2$ $[M+H]^+$; calculated 391.1208, found 391.1222.

$[\alpha]_D^{21}$ = −61 (c. 0.1, $CHCl_3$, 91:9 e.r.).

HPLC (CHIRALPAK® AS-3R, 60:40 MeCN–H_2O, 0.5 mL/min flow rate, 238 nm); t_r(e_1, major) = 14.3 min; t_r(e_2, minor) = 16.0 min.

* mmol estimated based on the molecular weight of the monohydrate form of the glyoxal.

1,3-Dibenzyl-5-(4-bromophenyl)imidazolidine-2,4-dione 17h

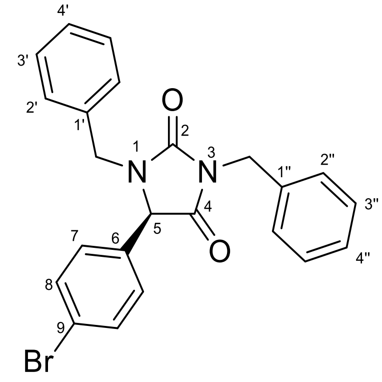

General procedure **A** was followed starting with 4-bromophenylglyoxal monohydrate **13h**[*] (23 mg, 100 µmol, 1.0 eq.) and 1,3-dibenzylurea **14a** (24 mg, 100 µmol, 1.0 eq.) for 40 h. Flash chromatography eluting with 90:10 hexane–EtOAc gave the *title compound* **17h** (42 mg, 98 µmol, 98%, 91:9 e.r.) as a colourless amorphous solid.

General procedure **B** was followed using 4-bromophenylglyoxal monohydrate **13h** (115 mg, 0.50 mmol, 1.0 eq.) 1,3-dibenzyl urea **14a** and (120 mg, 0.50 mmol, 1.0 eq.) for 40 h. Flash chromatography eluting with 90:10 petrol–EtOAc gave the *title compound* **17h** (209 mg, 0.48 mmol, 96%, 90:10 e.r.) as a colourless amorphous solid.

$\mathbf{R}_f$ 0.24 (90:10 hexane–EtOAc)

^{1}H NMR (600 MHz, $CDCl_3$): δ 7.52 (2H, d *J* 8.4, 8-H), 7.40-7.38 (2H, m, 2''-H), 7.35-7.32 (2H, m, 4'-H and 4''-H), 7.32-7.28 (4H, m, 3'-H and 3''-H), 7.11-7.08 (2H, m, 2'-H), 7.00 (2H, d, *J* 8.4, 7-H), 5.15 (1H, d, *J* 15.0, 1-NCH_AH$_B$Ph), 4.77 (1H, d, *J* 14.5, 3-NCH_AH$_B$Ph), 4.67 (1H, d, *J* 14.5, 3-NCH$_A$$H_B$Ph), 4.61 (1H, s, 5-H), 3.71 (1H, d, *J* 15.0, 1-NCH$_A$$H_B$Ph).

^{13}C NMR (150 MHz, $CDCl_3$ 1×Ar-C not observed): δ 170.7 (4-C), 156.4 (2-C), 136.1 (1''-C), 135.2 (1'-C), 132.7 (8-C), 131.7 (6-C), 129.3 (7-C), 129.2 (3'-C or 3''-C), 128.9 (3'-C or 3''-C), 128.6 (2'-C or 2''-C), 128.4 (4'-C), 128.2 (4''-C), 123.7 (9-C), 62.5 (5-C), 44.8 (1-N*C*H$_2$Ph), 43.0 (3-N*C*H$_2$Ph).

IR ν_{max}(film from $CDCl_3$)/cm^{-1} 1771 (C=O), 1706 (C=O), 1509, 1442, 1416, 1226, 1097.

HRMS (ESI): $C_{23}H_{20}{}^{79}BrN_2O_2$ $[M+H]^+$; calculated 435.0703, found 435.0700.

$[\alpha]_D^{21} = -42$ (c. 0.2, $CHCl_3$, 91:9 e.r.).

HPLC (CHIRALPAK® AS-3R, 50:50 MeCN–H_2O, 0.5 mL/min flow rate, 210 nm); t_r(e_1, major) = 42.5 min; t_r(e_2, minor) = 49.4 min.

[*] Vendor: AK Scientific, Inc.

X-ray crystallography: **X-ray crystallography**: **Crystal Data** for $C_{23}H_{19}N_2O_2Br$ (M =435.31 g/mol): monoclinic, space group $P2_1$ (no. 4), a = 12.5962(6) Å, b = 5.2216(2) Å, c = 15.4025(6) Å, β = 109.764(5)°, V = 953.38(7) Å^3, Z = 2, T = 120.0(2) K, μ(Cu Kα) = 3.112 mm^{-1}, $Dcalc$ = 1.516 g/cm^3, 8019 reflections measured (6.098° ≤ 2Θ ≤ 133.94°), 3014 unique (R_{int} = 0.0824, R_{sigma} = 0.0773) which were used in all calculations. The final R_1 was 0.0616 (I > 2σ(I)) and wR_2 was 0.1612 (all data). CCDC 2241398 contains the supplementary crystallographic data for this compound. Crystals were grown by slow evaporation from hexane–$CHCl_3$

1,3-Dibenzyl-5-(4-iodophenyl)imidazolidine-2,4-dione 17i

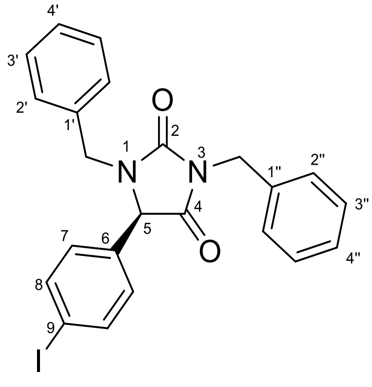

General procedure **A** was followed starting with 4-iodophenylglyoxal monohydrate **13i** (28 mg, 100 µmol,* 1.0 eq.) and 1,3-dibenzylurea **14a** (24 mg, 100 µmol, 1.0 eq.) for 40 h. Flash chromatography eluting with 90:10 hexane–EtOAc gave the *title compound* **17i** (47 mg, 97 µmol, 97%, 90:10 e.r.) as a colourless amorphous solid.

General procedure **B** was followed using 4-bromophenylglyoxal monohydrate **13i** (139 mg, 0.50 mmol, 1.0 eq.) 1,3-dibenzyl urea **14a** and (120 mg, 0.50 mmol, 1.0 eq.) for 40 h. Flash chromatography eluting with 90:10 petrol–EtOAc gave the *title compound* **17i** (463 mg, 0.48 mmol, 96%, 90:10 e.r.) as a colourless amorphous solid.

R*f* 0.25 (90:10 hexane–EtOAc).

^{1}H NMR (600 MHz, $CDCl_3$): δ 7.72 (2H, d, *J* 8.4, 8-H), 7.39-7.37 (2H, m, 2"-H), 7.35-7.32 (2H, m, 4' and 4"-H), 7.31-7.28 (4H, m, 3'-H and 3"-H), 7.14-7.03 (2H, m, 2'-H), 6.86 (2H, d, *J* 8.4, 7-H), 5.14 (1H, d, *J* 15.0, 1-NC$H_A$$H_B$Ph), 4.77 (1H, d, *J* 14.5, 3-NC$H_A$$H_B$Ph), 4.66 (1H, d, *J* 14.5, 3-NC$H_A$$H_B$Ph), 4.59 (1H, s, 5-H), 3.71 (1H, d, *J* 15.0, 1-NC$H_A$$H_B$Ph).

^{13}C NMR (150 MHz, $CDCl_3$): δ 170.6 (4-C), 156.4 (2-C), 138.6 (8-C), 136.1 (1"-C), 135.2 (1'-C), 132.4 (6-C), 129.5 (7-C), 129.2 (3'-C or 3"-C), 128.9 (3'-C or 3"-C), 128.6 (2 peaks 2'-C and 2"-C), 128.4 (4'-C), 128.2 (4"-C), 95.5 (9-C), 62.6 (5-C), 44.8 (1-N*C*H_2Ph), 43.0 (3-N*C*H_2Ph).

IR ν_{max}(film from $CDCl_3$)/cm^{-1} 3031, 1768 (C=O), 1707 (C=O), 1490, 1445, 1413, 1337.

HRMS (ESI): $C_{23}H_{20}IN_2O_2$ $[M+H]^+$; calculated 483.0564, found 483.0570.

$[\alpha]_D^{21}$ = −66 (c. 0.1, $CHCl_3$, 90:10 e.r.).

HPLC (CHIRALPAK® AS-3R, 70:30 MeCN–H_2O, 0.5 mL/min flow rate, 254 nm); t_r(e_1, major) = 9.7 min; t_r(e_2, minor) = 11.2 min.

* mmol estimated based on the molecular weight of the monohydrate form of the glyoxal.

1,3-Dibenzyl-5-(4-(trifluoromethyl)phenyl)imidazolidine-2,4-dione 17j

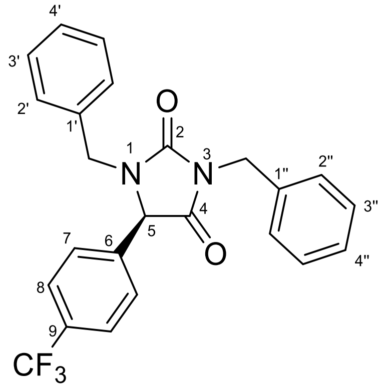

General procedure A was followed starting with 4-(trifluoromethyl)phenylglyoxal monohydrate **13j** (22 mg, 100 µmol,* 1.0 eq.) and 1,3-dibenzylurea **14a** (24 mg, 100 µmol, 1.0 eq.) at 60 °C for 4 h. Flash chromatography eluting with 90:10 petrol–EtOAc gave the *title compound* **17j** (42 mg, 98 µmol, 98%, 90:10 e.r.) as a colourless amorphous solid.

R*f* 0.23 (90:10 petrol–EtOAc).

^{1}H NMR (600 MHz, $CDCl_3$): δ 7.64 (2H, d, *J* 8.2, 8-H), 7.41-7.38 (2H, m, 2''-H), 7.35-7.32 (2H, m, 4'-H and 4''-H), 7.31-7.28 (4H, m, 3'-H and 3''-H), 7.26 (2H, d, *J* 8.2, 7-H), 7.11-7.06 (2H, m, 2'-H), 5.14 (1H, d, *J* 15.0, 1-NCH_AH_BPh), 4.77 (1H, d, *J* 14.5, 3-NCH_AH_BPh), 4.71 (1H, s, 5-H), 4.68 (1H, d, *J* 14.5, 3-NCH_AH_BPh), 3.75 (1H, d, *J* 15.0, 1-NCH_AH_BPh).

^{13}C NMR (150 MHz, $CDCl_3$, 1×Ar-C not observed): δ 170.4 (4-C), 156.5 (2-C), 138.7 (6-C), 136.0 (1''-C), 135.0 (1'-C), 131.7 (q, $^2J_{C\text{-}F}$ 32.6, 9-C), 129.2 (3'-C or 3''-C), 128.9 (3'-C or 3''-C), 128.6 (2'-C or 2''-C), 128.5 (4'-C), 128.2 (4''-C), 128.1 (7-C), 126.5 (q, $^3J_{C\text{-}F}$ 3.8, 8-C), 123.9 (q, $J_{C\text{-}F}$ 272.2, CF_3), 62.6 (5-C), 45.1 (1-NCH_2Ph), 43.1 (3-NCH_2Ph).

^{19}F NMR (376 MHz, $CDCl_3$): δ = −62.7.

IR ν_{max}(film from $CDCl_3$)/cm^{-1} 3031, 1769 (C=O), 1706 (C=O), 1490, 1441, 1417, 1336.

HRMS (ESI): $C_{24}H_{20}F_3N_2O_2$ $[M+H]^+$; calculated 425.1471, found 425.1485.

$[\alpha]_D^{22}$ = −41 (c. 0.1, $CHCl_3$, 90:10 e.r.).

HPLC (CHIRALPAK® AS-3R, 70:30 MeCN–H_2O, 0.5 mL/min flow rate, 254 nm); $t_r(e_1$, major) = 6.4 min; $t_r(e_2$, minor) = 7.2 min.

* mmol estimated based on the molecular weight of the monohydrate form of the glyoxal.

1,3-Dibenzyl-5-(4-nitrophenyl)imidazolidine-2,4-dione 17k

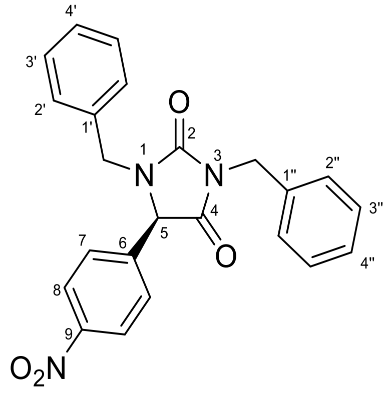

General procedure A was followed starting with 4-nitrophenylglyoxal monohydrate **13k** (20 mg, 101 µmol,[*] 1.0 eq.) and 1,3-dibenzylurea **14a** (24 mg, 100 µmol, 1.0 eq.) at 60 ºC for 4 h. Flash chromatography eluting with 90:10 petrol–EtOAc gave the *title compound* **17k** (38 mg, 95 µmol, 95%, 85:15 e.r.) as a colourless amorphous solid.

R*f* 0.18 (90:10 petrol–EtOAc).

^{1}H NMR (600 MHz, $CDCl_3$): δ 8.26 – 8.17 (2H, m, 8-H), 7.41-7.36 (2H, m, 2''-H), 7.36-7.27 (8H, m, 3'-H, 3''-H, 4'-H, 4''-H and 7-H), 7.12-7.05 (2H, m, 2'-H), 5.16 (1H, d, *J* 15.0, 1-NC$H_A$$H_B$Ph), 4.79-4.76 (2H, m, includes at δ 4.77 (1H, d, *J* 14.5: 3-NC$H_A$$H_B$Ph; and at δ 4.77: 1H, s, 5-H), 4.68 (1H, d, *J* 14.5, 3-NC$H_A$$H_B$Ph), 3.81 (1H, d, *J* 15.0, 1-NC$H_A$$H_B$Ph).

^{13}C NMR (150 MHz, $CDCl_3$, 2×Ar-C not observed): δ 169.7 (4-C), 156.4 (2-C), 148.6 (9-C) 139.7 (6-C), 135.8 (1''-C), 134.7 (1'-C), 129.2 (3'-C or 3''-C), 128.9 (3'-C, 3''-C or 7-C), 128.6 (2'-C, 2''-C or 7-C), 128.5 (4'-C), 128.3 (4''-C), 124.6 (8-C), 62.3 (5-C), 45.3 (1-N*C*H_2Ph), 43.1 (3-N*C*H_2Ph).

IR ν_{max}(film from $CDCl_3$)/cm^{-1} 2926, 1773 (C=O), 1709 (C=O), 1606, 1522, 1443, 1416.

HRMS (ESI): $C_{23}H_{20}N_3O_4$ $[M+H]^+$; calculated 402.1448, found 402.1457.

$[\alpha]_D^{22}$ = −54 (c. 0.1, $CHCl_3$, 85:15 e.r.).

HPLC (CHIRALPAK® AS-3R, 70:30 MeCN–H_2O, 0.5 mL/min flow rate, 254 nm); t_r(e_1, major) = 6.7 min; t_r(e_2, minor) = 7.1 min.

[*] mmol estimated based on the molecular weight of the monohydrate form of the glyoxal.

1,3-Dibenzyl-5-(3-methoxyphenyl)imidazolidine-2,4-dione 17l

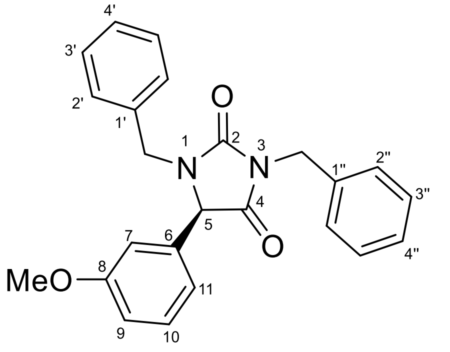

General procedure **A** was followed starting with 3-methoxyphenylglyoxal hemihydrate **13l** (17 mg, 99 µmol,* 1.0 eq.) and 1,3-dibenzylurea **14a** (24 mg, 100 µmol, 1.0 eq.) for 20 h. Flash chromatography eluting with 90:10 hexane–EtOAc gave the *title compound* **17l** (37 mg, 96 µmol, 96%, 94:6 e.r.) as a colourless amorphous solid.

R*f* 0.20 (90:10 hexane–EtOAc).

^{1}H NMR (600 MHz, $CDCl_3$): δ 7.44-7.38 (2H, m, 2''-H), 7.36-7.29 (6H, m Ar-H), 7.29-7.27 (1H, m, 10-H), 7.15-7.10 (2H, m, 2'-H), 6.91 (1H, ddd, *J* 8.3, 2.6, 0.9, 9-H), 6.72 (1H, app. dt, *J* 7.6, 1.3, 11-H), 6.60-6.56 (1H, m, 7-H), 5.13 (1H, d, *J* 15.0, 1-NCH_AH_BPh), 4.78 (1H, d, *J* 14.5, 3-NCH_AH_BPh), 4.68 (1H, d, *J* 14.5, 3-NCH_AH_BPh), 4.62 (1H, s, 5-C), 3.73 (1H, d, *J* 15.0, 1-NCH_AH_BPh), 3.72 (3H, s, OCH_3).

^{13}C NMR (150 MHz, $CDCl_3$): δ 171.1 (4-C), 160.4 (8-C), 156.5 (2-C), 136.3 (1''-C), 135.5 (1'-C), 134.1 (6-C), 130.5 (10-C) 129.1 (3'-C or 3''-C), 128.9 (3'-C or 3''-C), 128.7 (2'-C or 2''-C), 128.6 (2'-C or 2''-C), 128.3 (4'-C), 128.1 (4''-C), 120.1 (11-C), 115.3 (9-C), 112.8 (7-C), 63.1 (5-C), 55.4 (OCH_3), 44.7 (1-NCH_2Ph), 42.9 (3-NCH_2Ph).

IR ν_{max}(film from $CDCl_3$)/cm^{-1} 2928, 1769 (C=O), 1710 (C=O), 1602, 1492, 1443, 1419.

HRMS (ESI): $C_{24}H_{23}N_2O_3$ $[M+H]^+$; calculated 387.1703, found 387.1712.

$[\alpha]_D^{21}$ = −42 (c. 0.2, $CHCl_3$, 94:6 e.r.).

HPLC (Astec® Cellulose DMP column, 80:20 hexane–EtOH, 1 mL/min flow rate, 280 nm); t_r(e_1, major) = 9.7 min; t_r(e_2, minor) = 14.0 min.

* mmol estimated based on the molecular weight of the hemihydrate form of the glyoxal.

1,3-Dibenzyl-5-(3-chlorophenyl)imidazolidine-2,4-dione 17m

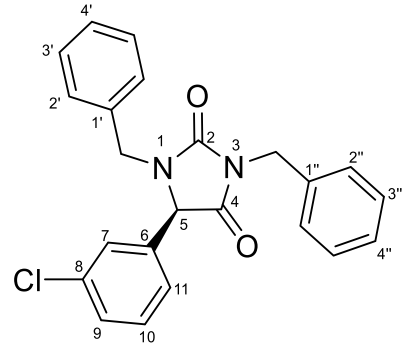

General procedure **A** was followed starting with 3-chlorophenylglyoxal hemihydrate **13m** (18 mg, 101 µmol,* 1.0 eq.) and 1,3-dibenzylurea **14a** (24 mg, 100 µmol, 1.0 eq.) for 40 h. Flash chromatography eluting with 90:10 hexane–EtOAc gave the *title compound* **17m** (37 mg, 100 µmol, 96%, 92:8 e.r.) as a colourless amorphous solid.

$\mathbf{R}_f$ 0.25 (90:10 hexane–EtOAc).

^{1}**H NMR** (600 MHz, $CDCl_3$): δ 7.41-7.38 (2H, m, 2''-H), 7.37-7.35 (2H, m, 10-H and 9-H) 7.35-7.32 (2H, m, 4'-H and 4''-H) 7.31-7.28 (4H, m, 3'-H and 3''-H), 7.14 -7.06 (3H, m, 7-H and 2'-H), 7.02 (1H, app. dt, *J* 7.6, 1.5, 1.5, 11-H), 5.15 (1H, d, *J* 15.0, 1-NC$H_A$$H_B$Ph), 4.78 (1H, d, *J* 14.5, 3-NC$H_A$$H_B$Ph), 4.68 (1H, d, *J* 14.5, 3-NC$H_A$$H_B$Ph), 4.68 (1H, s, 5-H), 3.75 (1H, d, *J* 15.0, 1-NC$H_A$$H_B$Ph).

^{13}**C NMR** (150 MHz, $CDCl_3$,1×Ar-C not observed) δ 170.5 (4-C), 156.4 (2-C), 136.1 (1''-C), 135.5 (8-C), 135.1 (1'-C) 134.7 (6-C), 130.7 (Ar-C), 129.8 (Ar-C), 129.2 (Ar-C), 128.9 (Ar-C), 128.6 (Ar-C), 128.6 (Ar-C), 128.5 (4'-C), 128.2 (4''-C), 127.8 (7-C), 125.9 (11-C), 62.5 (5-C), 45.0 (1-N*C*H_2Ph), 43.1 (3-N*C*H_2Ph).

IR ν_{max}(film from $CDCl_3$)/cm^{-1} 2923, 1765 (C=O), 1702 (C=O), 1493, 1449, 1417, 1336.

HRMS (ESI): $C_{23}H_{20}{}^{35}ClN_2O_2$ $[M+H]^+$; calculated 391.1208, found 391.1215.

$[\alpha]_D^{21}$ = −40 (c. 0.2, $CHCl_3$, 92:8 e.r.).

HPLC (Astec® Cellulose DMP column, 80:20 hexane–EtOH, 1.0 mL/min flow rate, 254 nm); $t_r(e_1$, major) = 10.0 min; $t_r(e_2$, minor) = 11.2 min.

* mmol estimated based on the molecular weight of the hemihydrate form of the glyoxal.

1,3-Dibenzyl-5-(3,4-difluorophenyl)imidazolidine-2,4-dione 17n

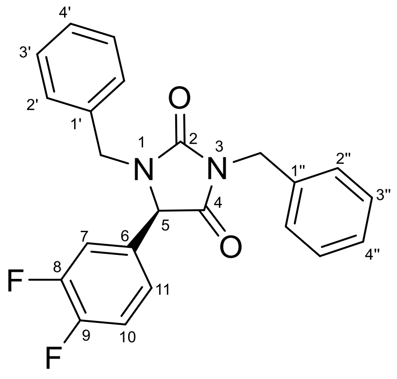

General procedure **A** was followed starting with 3,4-difluorophenylglyoxal monohydrate **13n**[*] (19 mg, 100 µmol, 1.0 eq.) and 1,3-dibenzylurea **14a** (24 mg, 100 µmol, 1.0 eq.) for 72 h. Flash chromatography eluting with 90:10 hexane–EtOAc gave the *title compound* **17n** (29 mg, 75 µmol, 75%, 93:7 e.r.) as a colourless amorphous solid.

General procedure **B** was followed using 3,4-difluorophenylglyoxal monohydrate **13n** (95 mg, 0.50 mmol, 1.0 eq.) 1,3-dibenzyl urea **14a** and (120 mg 0.50 mmol, 1.0 eq.) for 120 h. Flash chromatography eluting with 90:10 petrol–EtOAc gave the *title compound* **17n** (188 mg, 0.48 mmol, 96%, 91:9 e.r.) as a colourless amorphous solid.

R*f* 0.22 (90:10 hexane–EtOAc).

^{1}H NMR (600 MHz, $CDCl_3$): δ 7.42-7.37 (2H, m, 2"-H), 7.36-7.32 (2H, m, 4'-H and 4"-H) 7.31-7.28 (4H, m, 3'-H and 3"-H), 7.18 (1H, app. dt, *J* 9.9, 8.2, 10-H), 7.11-7.08 (2H, m, 2'-H), 6.94 (1H, ddd, *J* 10.3, 7.2, 2.2, 7-H), 6.90-6.85(1H, m, 11-H), 5.14 (1H, d, *J* 14.9, 1-NCH_AH$_B$Ph), 4.60 (1H, s, 5-H), 4.77 (1H, d, *J* 14.5, 3-NCH_AH$_B$Ph), 4.67 (1H, d, *J* 14.5, 3-NCH$_A$$H_B$Ph), 3.88 (1H, d, *J* 14.9, 1-NCH$_A$$H_B$Ph).

^{13}C NMR (150 MHz, $CDCl_3$, 1×Ar-C not observed) δ 170.4 (4-C), 156.4 (2-C), 151.8 (dd, $J_{C\text{-}F}$ 253.8, 15.3, 8-C or 9-C), 150.2 (dd, $J_{C\text{-}F}$ 253.8, 15.3, 8-C or 9-C), 136.0 (1"-C), 135.0 (1'-C), 129.6 (app. t, $^{3,4}J_{C\text{-}F}$ 4.3, 6-C), 129.2 (3'-C or 3"-C), 128.9 (3'-C or 3"-C), 128.6 (2'-C or 2"-C), 128.5 (4'-C), 128.2 (4"-C), 123.9 (dd, $^{3,4}J_{C\text{-}F}$ 6.6, 3.8, 11-C), 118.5 (d, $^{2}J_{C\text{-}F}$ 17.6, 10-C), 116.8 (d, $^{2}J_{C\text{-}F}$ 18.4, 7-C), 62.1 (5-C), 45.0 (1-N*C*H$_2$Ph), 43.1 (3-N*C*H$_2$Ph).

^{19}F NMR (565 MHz, $CDCl_3$): δ = −135.1, −135.8.

IR ν_{max}(film from $CDCl_3$)/cm^{-1} 3032, 1773 (C=O), 1710 (C=O), 1518, 1442, 1418, 1289.

HRMS (ESI): $C_{23}H_{19}F_2N_2O_2$ [M+H]$^+$; calculated 393.1409, found 393.1427.

$[\alpha]_D^{21}$ = −25 (c. 0.3, $CHCl_3$, 93:7 e.r.).

[*] Vendor: AK Scientific.

HPLC (CHIRALPAK® AS-3R, 50:50 MeCN–H_2O, 0.5 mL/min flow rate, 254 nm); t_r(e_1, major) = 23.8 min; t_r(e_2, minor) = 27.9 min..

1,3-Dibenzyl-5-(2-methoxy)phenyl)imidazolidine-2,4-dione 17o

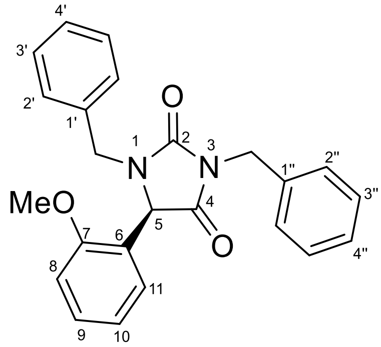

General procedure **A** was followed starting with 2-methoxyphenylglyoxal **13o*** (27 mg, 150 µmol, 1.5 eq.) and 1,3-dibenzylurea **2a** (24 mg, 100 µmol, 1.0 eq.) for 20 h. Flash chromatography eluting with 90:10 hexane–EtOAc gave the *title compound* **4o** (36 mg, 95 µmol, 95%, 79:21 e.r.) as a colourless amorphous solid.

R*f* 0.20 (90:10 hexane–EtOAc).

^{1}H NMR (600 MHz, CD_3CN, 345 K): δ 7.45 (2H, dd, *J* 8.4, 1.3, 2''-H), 7.41-7.37 (2H, m, 3''-H), 7.37-7.30 (2H, m, 9-H and 4''-H), 7.28-7.20 (3H, m, 3'-H and 4'-H), 7.12-7.06 (3H, m, 2'-H and 11-H), 6.93 (1H, td, *J* 7.5, 7.5, 1.2, 10-H), 6.90 (1H, d, *J* 8.3, 8-H), 4.98 (1H, s, 5-H), 4.74 (1H, d, *J* 14.8, 3-NCH_AH_BPh), 4.69 (1H, d, *J* 14.8, 3-NCH_AH_BPh), 4.66 (1H, d, *J* 15.4, 1-NCH_AH_BPh), 3.97 (1H, d, *J* 15.4, 1-NCH_AH_BPh), 3.46 (3H, s, OCH_3).

^{13}C NMR (150 MHz, CD_3CN, 345 K, 1×Ar-C not observed): δ 172.8 (4-C), 159.0 (7-C), 157.5 (2-C), 138.1 (1''-C), 137.7 (1'-C), 132.9 (11-C), 132.0 (9-C), 129.6 (3''-C), 129.3 (2''-C or 3'-C), 128.9 (2'-C), 128.6 (4''-C), 128.3 (4'-C), 121.9 (6-C), 121.5 (10-C), 112.4 (8-C), 62.9 (5-C), 55.9 (OCH_3), 45.5 (1-NCH_2Ph), 43.2 (3-NCH_2Ph).

IR ν_{max}(film from $CDCl_3$)/cm^{-1} 2931, 1771 (C=O), 1708 (C=O), 1585, 1490, 1445, 1417, 1331.

HRMS (ESI): $C_{24}H_{23}N_2O_3$ $[M+H]^+$; calculated 387.1703, found 387.1711.

$[\alpha]_D^{21}$ = −48 (c. 0.1, $CHCl_3$, 79:21 e.r.).

HPLC (CHIRALPAK® AS-3R, 50:50 MeCN–H_2O, 0.5 Ml/min flow rate, 280 nm); t_r(e_1, minor) = 23.2 min; t_r(e_2, major) = 24.1 min.

* mmol estimated based on the molecular weight of the glyoxal form.

1,3-Dibenzyl-5-(2-chlorophenyl)imidazolidine-2,4-dione 17p

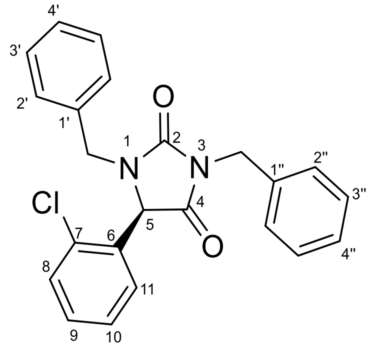

General procedure **A** was followed starting with 2-chlorophenylglyoxal **13p** (30 mg, 150 µmol,* 1.5 eq.) and 1,3-dibenzylurea **14a** (24 mg, 100 µmol, 1.0 eq.) for 40 h. Flash chromatography eluting with 90:10 hexane–EtOAc gave the *title compound* **17p** (36 mg, 92 µmol, 92%, 64:34 e.r.) as a colourless amorphous solid.

$\mathbf{R}_f$ 0.22 (90:10 hexane–EtOAc).

^{1}H NMR (600 MHz, CD_3CN, 345 K): δ 7.43-7.39 (2H, m 2''-H), 7.39-7.35 (4H, m, 3''-H, 8-H and 9-H), 7.34-7.29 (2H, m, 4''-H and 11-H), 7.29-7.23 (3H, m, 3' and 4'-H), 7.17 (1H, dd, *J* 7.7, 1.7, 10-H), 7.15-7.10 (2H, m, 2'-H), 5.28 (1H, br. s, 5-H), 4.79 (1H, d, *J* 15.3, 1-NC$H_A$$H_B$Ph), 4.74 (1H, d, *J* 15.0, 3-NC$H_A$$H_B$Ph), 4.71 (1H, d, *J* 15.0, 3-NC$H_A$$H_B$Ph), 4.02 (1H, d, *J* 15.3, 1-NC$H_A$$H_B$Ph).

^{13}C NMR (150 MHz, CD_3CN, 345 K, 1×Ar-C_q, and 5×Ar-C not observed): δ 171.5 (4-C), 157.5 (2-C), 137.5 (1''-C), 136.9 (1'-C), 134.8 (7-C), 131.8 (Ar-C), 131.4 (Ar-C), 129.5 (Ar-C), 129.2 (Ar-C), 128.7 (Ar-C), 60.5 (5-C), 45.9 (1-N*C*H_2Ph), 43.4 (3-N*C*H_2Ph).

IR ν_{max}(film from $CDCl_3$)/cm^{-1} 3030, 1767 (C=O), 1703 (C=O), 1491, 1441, 1417, 1336.

HRMS (ESI): $C_{23}H_{20}{}^{35}ClN_2O_2$ $[M+H]^+$; calculated 391.1208, found 391.1213.

$[\alpha]_D^{21}$ = −28 (c. 0.1, $CHCl_3$, 64:36 e.r.).

HPLC (CHIRALPAK® AS-3R, 60:40 MeCN–H_2O, 0.5 mL/min flow rate, 220 nm); t_r(e_1, major) = 12.0 min; t_r(e_2, minor) = 12.7 min.

* mmol estimated based on the molecular weight of glyoxal form.

1,3-Dibenzyl-5-(2-(trifluoromethyl)phenyl)imidazolidine-2,4-dione 17q

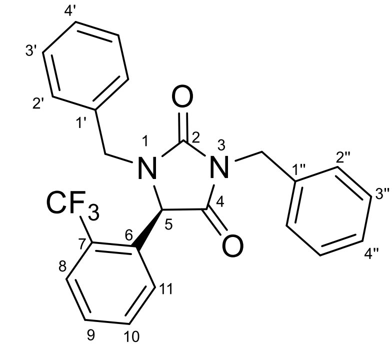

General procedure **A** was followed starting with the 2-(trifluoromethyl)phenylglyoxal **13q** (30 mg, estimated 150 µmol*, 1.5 eq.) and 1,3-dibenzylurea **14a** (24 mg, 100 µmol, 1.0 eq.) for 7 days. Flash chromatography eluting with 90:10 hexane–EtOAc gave the *title compound* **17q** (22 mg, 54 µmol, 54%, 71:29 e.r.) as a colourless amorphous solid.

R*f* 0.23 (90:10 hexane–EtOAc).

^{1}H NMR (600 MHz, $CDCl_3$): δ 7.72 (1H, dd, *J* 7.1, 2.1, 8-H), 7.48-7.43 (2H, m, 9-H and 10-H), 7.43-7.40 (2H, m, 2''-H), 7.36-7.28 (3H, m, Ar-H), 7.25-23 (3H, m, Ar-H), 7.12-7.06 (3H, m, 11-H and 2'-H), 5.25 (1H, s, 5-H), 4.95 (1H, d, *J* 15.2, 1-NCH_AH_BPh), 4.78 (1H, d, *J* 14.4, 3-NCH_AH_BPh), 4.69 (1H, d, *J* 14.4, 3-NCH_AH_BPh), 3.84 (1H, d, *J* 15.2, 1-NCH_AH_BPh).

^{13}C NMR (150 MHz, $CDCl_3$, 1×Ar-C not observed): δ 170.0 (4-C), 156.8 (2-C), 136.0 (1''-C), 135.3 (1'-C), 132.8 (9-C), 131.8 (6-C) 130.3 (q, $^2J_{C-F}$ 32.6, 7-C), 129.4 (10-C), 128.9 (Ar-C), 128.8 (Ar-C), 128.6 (Ar-C), 128.2 (Ar-C), 128.2 (Ar-C), 127.6 (11-C), 126.8 (q, $^3J_{C-F}$, 5.9, 8-C), 123.7 (q, J_{C-F} 274.2, CF_3), 59.2 (5-C), 45.1 (NCH_2Ph), 43.1 (NCH_2Ph).

^{19}F NMR (376 MHz, $CDCl_3$): δ = −57.1.

IR ν_{max}(film from $CDCl_3$)/cm^{-1} 3031, 1767 (C=O), 1706 (C=O), 1490, 1441, 1417, 1336.

HRMS (ESI): $C_{24}H_{20}F_3N_2O_2$ $[M+H]^+$; calculated 425.1471, found 425.1486.

$[\alpha]_D^{21}$ = −22 (c. 0.2, $CHCl_3$, 70:30 e.r.).

HPLC (Astec® Cellulose DMP column, 80:20 hexane–EtOH, 1 mL/min flow rate, 254 nm), t_r(e_1, major) = 8.0 min; t_r(e_2, minor) = 9.2 min.

* mmol estimated based on the molecular weight of the glyoxal from.

1,3-Dibenzyl-5-(naphthalen-1-yl)imidazolidine-2,4-dione 17r

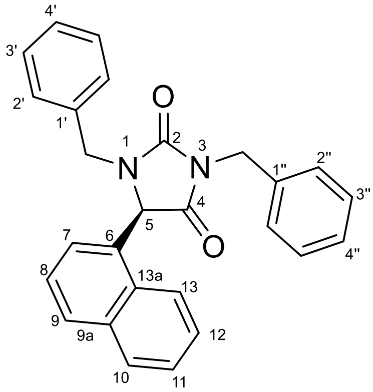

General procedure **A** was followed starting with 1-naphthylglyoxal hemihydrate **13r** (20 mg, 103 µmol,* 1.0 eq.) and 1,3-dibenzylurea **24a** (24 mg, 100 µmol, 1.0 eq.). Flash chromatography eluting with 90:10 hexane–EtOAc gave the *title compound* **17r** (39 mg, 96 µmol, 96%, 74:26 e.r.) as a colourless amorphous solid.

R*f* 0.22 (90:10 hexane–EtOAc).

^{1}H NMR (600 MHz, $CDCl_3$, 223 K, 50:50 mixture of rotamers): δ 7.97-7.90 (2H, m, 10 and 13-H), 7.90-7.86 (0.5H, m, Ar-H), 7.63-7.59 (1H, m, 2''-H), 7.58-7.55 (1H, m, 11-H and 12-H), 7.55-7.51 (1H, m, Ar-H), 7.49-7.41 (3.5H, m, Ar-H and 2''-H). 7.40-7.28 (5.5H, m, Ar-H), 7.21-7.16 (1H, m, 2'-H), 7.11-7.05 (1H, m, 2'-H), 6.93 (0.5H, d, *J* 8.4, Ar-H), 6.92-6.86 (0.5H, m, Ar-H), 5.63 (0.5H, s, 5-H), 5.32 (0.5H, d, *J* 15.1, 1-NCH_AH$_B$Ph), 5.08 (0.5H, d, *J* 15.1, 1-NCH_AH$_B$Ph), 5.04 (0.5H, s, 5-H), 4.98 (1H, d, *J* 13.9, 3-NCH_AH$_B$Ph), 4.86-4.79 (1H, m, includes at δ 4.83: 0.5H, d, *J* 13.9, 3-NCH$_A$$H_B$Ph; and at δ 4.82: 0.5H, d, *J* 14.6, 3-NCH_AH$_B$Ph), 4.65 (0.5H, d, *J* 14.6, 3-NCH$_A$$H_B$Ph), 3.80 (0.5H, d, *J* 15.1, 1-NCH$_A$$H_B$Ph), 3.40 (0.5H, d, *J* 15.1, 1-NCH$_A$$H_B$Ph).

^{13}C NMR (150 MHz, $CDCl_3$, mixture of two rotamers): δ 171.4 (4-C), 171.1 (4-C), 157.0 (2-C), 155.9 (2-C), 136.2 (1''-C), 135.9 (1''-C), 135.5 (Ar-C_q), 134.4 (Ar-C_q), 132.1 (6-C), 132.0 (6-C), 131.1 (Ar-C_q), 129.8 (Ar-C), 129.6 (Ar-C), 129.3 (Ar-C), 129.0 (Ar-C), 129.0 (Ar-C), 128.8 (Ar-C), 128.7 (Ar-C), 128.6 (Ar-C), 128.5 (Ar-C), 128.3 (Ar-C), 128.1 (Ar-C), 127.0 (Ar-C), 126.4 (Ar-C), 125.5 (Ar-C), 125.1 (Ar-C), 123.6 (Ar-C), 123.2 (Ar-C), 122.5 (Ar-C), 65.3 (5-C), 58.3 (5-C), 44.8 (1-N*C*H_2Ph), 43.2 (3-N*C*H_2Ph), 43.0 (3-N*C*H_2Ph) [34 of 46 expected peaks observed].

IR ν_{max}(neat)/cm^{-1} 2927, 1769 (C=O), 1699 (C=O), 1492, 1441, 1416, 1074.

HRMS (ESI): $C_{27}H_{23}N_2O_2$ $[M+H]^+$; calculated 407.1754, found 407.1764.

$[\alpha]_D^{21}$ = −29 (c. 0.1, $CHCl_3$, 74:26 e.r.).

* mmol estimated based on the molecular weight of the hemihydrate form of the glyoxal.

HPLC (Astec® Cellulose DMP column, 25 cm, 80:20 hexane–EtOH, 1 mL/min flow rate, 254 nm); t_r(e_1, major) = 12.0 min; t_r(e_2, minor) = 23.0 min.

1,3-Dibenzyl-5-(naphthalen-2-yl)imidazolidine-2,4-dione 17s

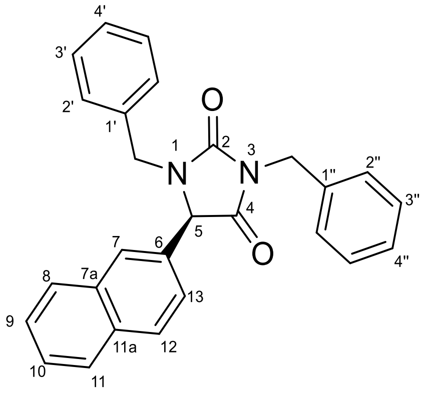

General procedure **A** was followed starting with 2-naphthylglyoxal monohydrate **13s** (20 mg, 99 µmol,* 1.0 eq.) and 1,3-dibenzylurea **14a** (24 mg, 100 µmol, 1.0 eq.) for 20 h. Flash chromatography eluting with 90:10 hexane–EtOAc gave *title compound* **17s** (39 mg, 96 µmol, 96%, 94:6 e.r.) as a colourless amorphous solid.

R*f* 0.22 (90:10 hexane–EtOAc).

^{1}H NMR (600 MHz, $CDCl_3$): δ 7.90-7.84 (2H, m, 8-H and 12-H), 7.83-7.79 (1H, m, 11-H), 7.64 (1H, s, 7-H), 7.57-7.50 (2H, m, 9-H and 10-H), 7.48-7.42 (2H, m, 2''-H), 7.37-7.34 (2H, 4'-H and 4''-H), 7.32 (4H, m, 3'-H and 3''-H), 7.17 (1H, dd, *J* 8.5, 1.9, 13-H), 7.15-7.10 (2H, m, 2'-H), 5.20 (1H, d, *J* 15.1, 1-NCH_AH$_B$Ph), 4.84 (1H, s, 5-H), 4.84 (1H, d, *J* 14.5, 3-NCH_AH$_B$Ph), 4.72 (1H, d, *J* 14.5, 3-NCH$_A$$H_B$Ph), 3.75 (1H, d, *J* 15.1, 1-NCH$_A$$H_B$Ph).

^{13}C NMR (150 MHz, $CDCl_3$, 1×Ar-C not observed): δ 171.2 (4-C), 156.5 (2-C), 136.3 (1''-C), 135.5 (1'-C), 133.8 (Ar-C$_q$), 133.4 (Ar-C$_q$), 129.9 (6-C), 129.7 (Ar-C) 129.1 (3'-C or 3''-C), 128.9 (3'-C or 3''-C), 128.6 (2'-C or 2''-C), 128.3 (9-C), 128.3 (4'-C), 128.1 (2 peaks, Ar-C), 128.0 (2 peaks, Ar-C), 127.0 (Ar-C), 126.9 (Ar-C), 124.0 (13-C), 63.3 (5-C), 44.8 (1-N*C*H$_2$Ph), 43.0 (3-N*C*H$_2$Ph).

IR ν_{max}(neat)/cm^{-1} 3031, 1769 (C=O), 1706 (C=O), 1495, 1418, 1338, 1137.

HRMS (ESI): $C_{27}H_{23}N_2O_2$ [M+H]$^+$; calculated 407.1754, found 407. 1765.

$[\alpha]_D^{21}$ = −49 (c. 0.1, $CHCl_3$, 94:6 e.r.).

HPLC (Astec® Cellulose DMP column, 25 cm, 80:20 hexane–EtOH, 1 mL/min flow rate, 254 nm); t_r(e_1, major) = 11.5 min; t_r(e_2, minor) = 19.1 min.

* mmol estimated based on the molecular weight of the monohydrate form of the glyoxal.

1,3-Dibenzyl-5-(5-bromothiophen-2-yl)imidazolidine-2,4-dione 17t

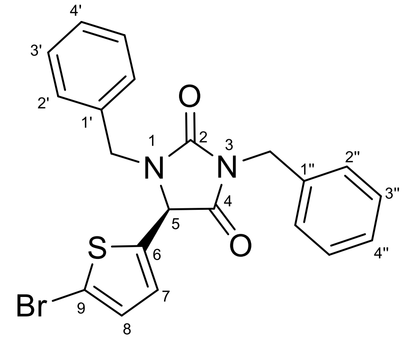

General procedure **A** was followed starting with 5-bromo-2-thiopheneglyoxal monohydrate **17t**[*] (23 mg, 99 µmol, 1.0 eq,) and 1,3-dibenzylurea **14a** (24.0 mg, 101 µmol, 1.0 eq.). Flash chromatography eluting with 90:10 hexane–EtOAc gave the *title compound* **17t** (43 mg, 97 µmol, 97%, 91:9 e.r.) as a colourless amorphous solid.

R*f* 0.23 (90:10 hexane–EtOAc).

^{1}H NMR (600 MHz, $CDCl_3$): δ 7.40-7.37 (2H, m, 2"-H), 7.36-7.29 (6H, m, 3'-H, 3"-H, 4'-H and 4"-H), 7.19-7.11 (2H, m, 2'-H), 6.98 (1H, d, *J* 3.8, 8-H), 6.76 (1H, dd, *J* 3.8, 0.5, 7-H), 5.16 (1H, d, *J* 15.0, 1-NCH_AH$_B$Ph), 4.84 (1H, s, 5-H), 4.77 (1H, d, *J* 14.5, 3-NCH_AH$_B$Ph), 4.67 (1H, d, *J* 14.5, 3-NCH$_A$$H_B$Ph), 3.88 (1H, d, *J* 15.0, 1-NCH$_A$$H_B$Ph).

^{13}C NMR (150 MHz, $CDCl_3$) δ 169.5 (4-C), 155.8 (2-C), 136.4 (6-C), 135.9 (1"-C), 135.1 (1'-C), 130.4 (7-C), 129.2 (8-C), 128.9 (3'-C or 3"-C), 128.8 (3'-C or 3"-C), 128.7 (2"-C), 128.5 (2'-C), 128.4 (4'-C) 128.2 (4"-C) 114.32 (9-C), 58.7 (5-C), 44.9 (1-N*C*H$_2$Ph), 43.1 (3-N*C*H$_2$Ph).

IR ν_{max}(film from $CDCl_3$)/cm^{-1} 3031, 1773 (C=O), 1707 (C=O), 1493, 1440, 1416, 1337.

HRMS (ESI): $C_{21}H_{18}{}^{79}BrN_2O_2$ $[M+H]^+$; calculated 441.0267, found 441.0266.

$[\alpha]_D^{21}$ = −87 (c. 0.2, $CHCl_3$, 91:9 e.r.).

HPLC (CHIRALPAK® AS-3R, 80:20 MeCN–H_2O, 0.5 mL/min flow rate, 280 nm); t_r(e_1, major) = 6.0 min; t_r(e_2, minor) = 6.4 min.

[*] Vendor: AK Scientific.

1,3-Dimethyl-5-phenylimidazolidine-2,4-dione 17u

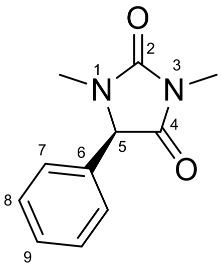

General procedure **A** was followed starting with phenylglyoxal monohydrate* **13a** (15 mg, 99 µmol, 1.0 eq.) and 1,3-dimethylurea **14b**† (9.0 mg, 101 µmol, 1.00 eq.) for 20 h. Flash chromatography eluting with 70:30 hexane–EtOAc gave the title compound **17u** (19 mg, 95 µmol, 95%, 85:15 e.r.) as a colourless amorphous solid.

R*f* 0.32 (60:40 hexane–EtOAc).

^{1}H NMR (600 MHz, $CDCl_3$): δ 7.46-7.36 (3H, m, 8 and 9-H), 7.26-7.23 (2H, m, 7-H), 4.86 (1H, s, 5-H), 2.94 (3H, s, 3-NCH_3), 2.78 (3H, s, 1-NCH_3).

^{13}C NMR (150 MHz, $CDCl_3$ 1×Ar-C not observed): δ 172.5 (4-C), 157.9 (2-C), 137.7 (6-C), 129.5 (Ar-C), 127.4 (7-C), 66.1 (5-C), 28.2 (1-NCH_3), 25.3 (3-NCH_3).

IR ν_{max}(neat)/cm^{-1} 2943, 1761 (C=O), 1707 (C=O), 1464, 1386, 1272, 1222.

HRMS (ESI): $C_{11}H_{13}O_2N_2$ $[M+H]^+$; calculated: 205.0971, found 205.0972.

$[\alpha]_D^{21}$ = −62 (c. 0.1, $CHCl_3$ for 85:15 e.r.).

HPLC (CHIRALPAK® AS-3R, 30:70 MeCN–H_2O, 0.5 mL/min flow rate, 220 nm); t_r(e_1, major) = 8.2 min; t_r(e_2, minor) = 9.5 min.

The spectral data are consistent with the literature values.[10]

* Vendor: Alfa Aesar.
† Vendor: Sigma-Aldrich.

1,3-Bis[(2,4-dimethoxyphenyl)methyl]-5-phenylimidazolidine-2,4-dione 17v-a

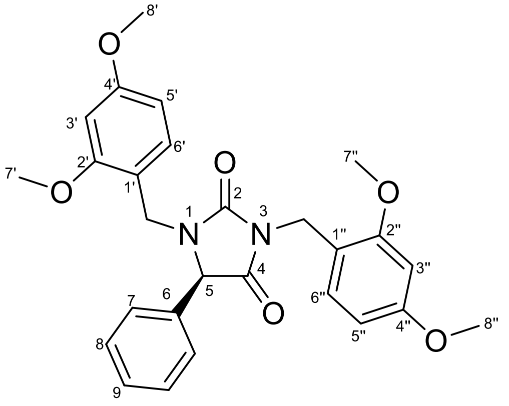

General procedure **A** was followed using phenylglyoxal monohydrate **13a**[*] (15 mg, 99 µmol, 1.0 eq.) and 1,3-bis[(2,4-dimethoxyphenyl)methyl]urea **14c** (36 mg, 101 µmol, 1.0 eq.) for 20 h. Flash chromatography eluting with 70:30 petrol–EtOAc gave the *title compound* **17v-a** (46 mg, 97 µmol, 97%, 89:11 e.r.) as a colourless amorphous solid.

General procedure **B** was followed using phenylglyoxal monohydrate **13a** (76 mg, 0.50 mmol, 1.0 eq.) 1,3-bis[(2,4-dimethoxyphenyl)methyl]urea **14c** and (180 mg 0.50 mmol, 1.0 eq.) for 20 h. Flash chromatography eluting with 90:10 petrol–EtOAc gave the *title compound* **17v-a** (231 mg, 0.48 mmol, 97%, 89:11 e.r.) as a colourless amorphous solid.

$\mathbf{R}_f$ 0.28 (7:3 petrol–EtOAc).

^{1}H NMR (600 MHz, $CDCl_3$): δ 7.41-7.34 (3H, m, 8-H and 9-H), 7.19 (2H, dd, *J* 7.7, 1.7, 7-H), 7.15 (1H, d, *J* 8.7, 6″-H), 7.06 (1H, d, *J* 8.9, 6′-H), 6.41-6-37 (4H, m, 3′-H, 3″-H, 5″-H and 5″-H), 4.90 (1H, d, *J* 14.8, 1-NC$H_A$$H_B$Ar), 4.75 (1H, d, *J* 15.0, 3-NC$H_A$$H_B$Ar), 4.72 (1H, s, 5-C), 4.58 (1H, d, *J* 15.0, 3-NC$H_A$$H_B$Ar), 3.90 (1H, d, *J* 14.8, 1-NC$H_A$$H_B$Ar), 3.79 (3H, s 8′-$CH_3$), 3.78 (3H, s, 8″-$CH_3$), 3.70 (3H, s, 7′-$CH_3$), 3.66 (3H, s, 7″-$CH_3$).

^{13}C NMR (150 MHz, $CDCl_3$): δ 171.5 (4-C), 161.1 (4′-C), 160.6 (4″-C) 158.7 (2′-C), 158.3 (2″-C) 156.7 (2-C), 133.9 (6-C), 131.9 (6″-C), 130.2 (6′-C), 129.1 (8-C), 129.0 (9-C), 127.5 (7-C), 116.7 (1″-C), 116.4 (1′-C), 104.2 (5′-C), 104.0 (5″-C), 98.5 (2 peaks 3′-C and 3″-C), 63.6 (5-C), 55.5 (2 peaks, OCH_3), 55.4 (OCH_3), 55.3 (OCH_3), 39.8 (1-NCH_2Ar), 37.9 (3-NCH_2Ar).

IR ν_{max}(neat)/cm^{-1} 2937, 1771 (C=O), 1708 (C=O), 1612, 1588, 1507, 1443.

HRMS (ESI): $C_{27}H_{29}N_2O_6$ $[M+H]^+$; calculated: 477.2020, found 477.2031.

$[\alpha]_D^{23}$ = −51 (c. 0.2, $CHCl_3$ for 89:11 e.r.).

[*] Vendor: Alfa Aesar.

HPLC (Astec® Cellulose DMP column, 70:30 hexane–EtOH, 1.0 mL/min flow rate, 254 nm); t_r(e_1, major) = 14.7 min; t_r(e_2, minor) = 20.2 min.

1,3-Bis[(2,4-dimethoxyphenyl)methyl]-5-(4-methylphenyl)imidazolidine-2,4-dione 17v-b

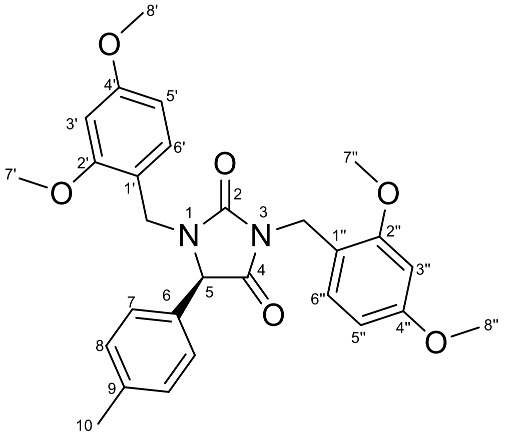

General procedure **A** was followed using 4-methylphenylglyoxal monohydrate **13b** (17 mg, 101 µmol,* 1.0 eq.) and 1,3-bis[(2,4-dimethoxyphenyl)methyl]urea **14c** (36 mg, 101 µmol, 1.0 eq.) for 20 h. Flash chromatography eluting with 70:30 petrol–EtOAc gave the *title compound* **17v-b** (48 mg, 98 µmol, 98%, 89:11 e.r.) as a colourless amorphous solid.

R*f* 0.29 (7:3 petrol–EtOAc).

^{1}H NMR (600 MHz, $CDCl_3$): δ 7.18 (2H, d, *J* 8.1, 8-H), 7.14 (1H, d, *J* 8.9, 6″-H), 7.07 (2H, d, *J* 8.1, 7-H), 7.06 (1H, d, *J* 8.9, 6′-H) 6.44-6.37 (4H, m, 3′-H, 3″-H, 5′-H and 5″-H), 4.90 (1H, d, *J* 14.7, 1-NC$H_A$$H_B$Ar), 4.74 (1H, d, *J* 15.0, 3-NC$H_A$$H_B$Ar), 4.69 (1H, s, 5-H), 4.59 (1H, d, *J* 15.0, 3-NC$H_A$$H_B$Ar), 3.87 (1H, d, *J* 14.7, 1-NC$H_A$$H_B$Ar), 3.79 (3H, s, 8′-$CH_3$), 3.78 (3H, s, 8″-$CH_3$), 3.72 (4H, s, 7′-$CH_3$), 3.67 (3H, s, 7″-$CH_3$), 2.36 (3H, s, 10-$CH_3$).

^{13}C NMR (150 MHz, $CDCl_3$, 1×OCH_3 not observed): δ 171.7 (4-C), 161.1 (4′-C), 160.6 (4″-C), 158.7 (2′-C), 158.3 (2″-C) 156.6 (2-C), 138.8 (9-C) 131.8 (6″-C), 130.8 (6-C), 130.0 (6′-C), 129.8 (8-C), 127.4 (7-C), 116.7 (1″-C), 116.5 (1′-C), 104.3 (5′-C), 104.1 (5″-C), 98.5 (2 peaks 3′-C and 3″-C), 63.4 (5-C), 55.5 (OCH_3), 55.4 (OCH_3), 55.2 (OCH_3), 39.7 (1-NCH_2Ar), 37.8 (3-NCH_2Ar), 21.3 (10-C).

IR ν_{max}(neat)/cm^{-1} 2939, 1772 (C=O), 1705 (C=O), 1613, 1592, 1502, 1445.

HRMS (ESI): $C_{28}H_{31}N_2O_7$ $[M+H]^+$; calculated: 491.2182, found 491.2193.

$[\alpha]_D^{23}$ = −56 (c. 0.3, $CHCl_3$ for 89:10 e.r.).

HPLC (Astec® Cellulose DMP column, 70:30 hexane–EtOH, 1.0 mL/min flow rate, 254 nm); t_r(e_1, major) = 15.7 min; t_r(e_2, minor) = 18.3 min.

* mmol estimated based on the molecular weight of the monohydrate form of the glyoxal.

1,3-Bis[(2,4-dimethoxyphenyl)methyl]-5-(4-methoxyphenyl)imidazolidine-2,4-dione 17v-c

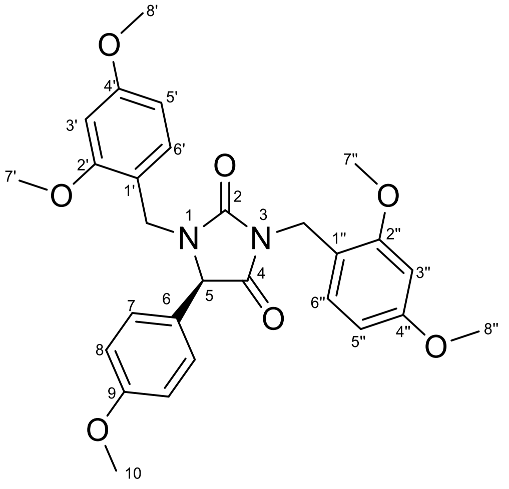

General procedure **A** was followed using 4-methoxyphenylglyoxal monohydrate **13d*** (18 mg, 99 μmol, 1.0 eq.) and 1,3-bis[(2,4-dimethoxyphenyl)methyl]urea **14c** (36 mg, 101 μmol, 1.0 eq.) for 20 h. Flash chromatography eluting with 70:30 petrol–EtOAc gave the *title compound* **17v-c** (49 mg, 97 μmol, 97%, 90:10 e.r.) as a colourless amorphous solid.

$\mathbf{R}_f$ 0.26 (7:3 petrol–EtOAc).

^{1}H NMR (600 MHz, $CDCl_3$): δ 7.15 (1H, d, *J* 8.9, 6″-H), 7.10 (2H, d, *J* 8.8, 7-H), 7.06 (1H, d, *J* 8.9, 6′-H), 6.90 (2H, d, *J* 8.8, 8-H), 6.46-6.36 (4H, m, 3′-H, 3″-H, 5′-H and 5″-H), 4.89 (1H, d, *J* 14.7, 1-NCH_AH$_B$Ar), 4.75 (1H, d, *J* 15.0, 3-NCH_AH$_B$Ar), 4.67 (1H, s, 5-H), 4.60 (1H, d, *J* 15.0, 3-NCH$_A$$H_B$Ar), 3.87 (1H, d, *J* 14.7, 1-NCH$_A$$H_B$Ar), 3.81 (3H, s, 10-$CH_3$), 3.79 (3H, s, 8′-$CH_3$), 3.78 (3H, s, 8″-$CH_3$), 3.73 (3H, s, 7′-$CH_3$), 3.68 (3H, s, 7″-$CH_3$).

^{13}C NMR (150 MHz, $CDCl_3$, 3×OCH$_3$ not observed): δ 171.8 (4-C), 161.1 (4′-C), 160.6 (4″-C) 160.2 (9-C), 158.7 (2′-C), 158.3 (2″-C) 156.6 (2-C), 131.8 (6″-C), 130.1 (6′-C),128.8 (7-C), 125.8 (6-C) 116.8 (1″-C), 116.5 (1′-C), 114.5 (8-C), 104.3 (5′-C), 104.1 (5″-C), 98.6 (3′-C or 3″-C), 98.5 (3′-C or 3″-C) 63.1 (5-C), 55.5 (2 peaks, OCH$_3$), 55.3 (OCH$_3$), 39.7 (1-NCH$_2$Ar), 37.8 (3-NCH$_2$Ar).

IR ν_{max}(neat)/cm^{-1} 2937, 1771 (C=O), 1708 (C=O), 1612, 1588, 1507, 1443.

HRMS (ESI): $C_{28}H_{31}N_2O_7$ $[M+H]^+$; calculated: 507.2131, found 507.2148.

$[\alpha]_D^{23}$ = −49 (c. 0.4, $CHCl_3$ for 90:10 e.r.).

HPLC (Astec® Cellulose DMP column, 70:30 hexane–EtOH, 1.0 mL/min flow rate, 254 nm); t_r(e_1, major) = 20.1 min; t_r(e_2, minor) = 27.1 min.

* Vendor: Alfa Aesar.

1,3-Bis[(2,4-dimethoxyphenyl)methyl]-5-(4-fluorophenyl)imidazolidine-2,4-dione 4v-d

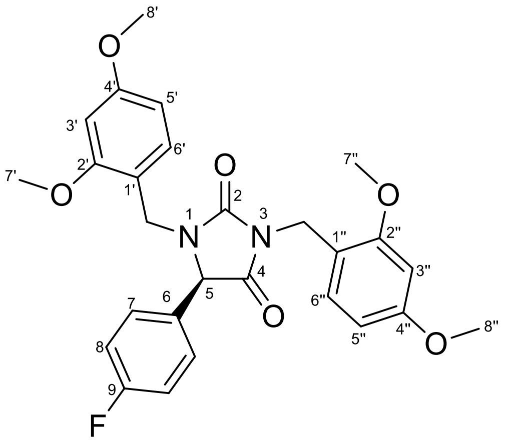

General procedure **A** was followed using 4-fluorophenylglyoxal monohydrate **13f*** (18 mg, 99 µmol, 1.0 eq.) and 1,3-bis[(2,4-dimethoxyphenyl)methyl]urea **14c** (36 mg, 101 µmol, 1.0 eq.) for 20 h. Flash chromatography eluting with 70:30 petrol–EtOAc gave the *title compound* **17v-d** (45 mg, 92 µmol, 92%, 81:19 e.r.) as a colourless amorphous solid.

^{1}H NMR (600 MHz, $CDCl_3$): δ 7.19-7.13 (3H, m, 7-H and 6″-H), 7.10-7.04 (3H, m, 8-H and 6′-H), 6.44-6.37 (4H, m, 3′-H, 3″-H, 5′-H and 5″-H), 4.90 (1H, d, *J* 14.7, 1-NCH_AH$_B$Ar), 4.74 (1H, d, *J* 14.9, 3-NCH_AH$_B$Ar), 4.69 (1H, s, 5-H), 4.59 (1H, d, *J* 14.9, 3-NCH$_A$$H_B$Ar), 3.90 (1H, d, *J* 14.7, 1-NCH$_A$$H_B$Ar), 3.79 (3H, s, 8′-$CH_3$), 3.78 (3H, s, 8″-$CH_3$), 3.71(3H, s, 7′-$CH_3$), 3.65 (3H, s, 7″-$CH_3$).

^{13}C NMR (150 MHz, $CDCl_3$): δ 171.3 (4-C), 163.1 (d, $J_{C\text{-}F}$ 247.8, 9-C) 161.1 (4′-C), 160.6 (4″-C), 158.7 (2′-C), 158.3 (2″-C) 156.6 (2-C), 131.9 (6″-C), 130.2 (6′-C), 129.7 (d, $^4J_{C\text{-}F}$ 3.6, 6-C), 129.5 (d, $^3J_{C\text{-}F}$ 8.7, 7-C), 116.5 (1″-C), 116.2 (1′-C), 116.1 (d, $^2J_{C\text{-}F}$ 21.7, 8-C), 104.3 (5′-C), 104.1 (5″-C), 98.5 (2 peaks, 3′-C and 3″-C), 62.8 (5-C), 55.5 (O*C*H_3), 55.4 (2 peaks, O*C*H_3), 55.2 (O*C*H_3), 39.8 (1-N*C*H_2Ar), 37.9 (3-N*C*H_2Ar).

IR ν_{max}(neat)/cm^{-1} 2941, 1770 (C=O), 1707 (C=O), 1613, 1587, 1511, 1442.

HRMS (ESI): $C_{27}H_{28}FN_2O_6$ [M+H]$^+$; calculated: 495.1926, found 495.1931.

$[\alpha]_D^{23}$ = −39 (c. 0.2, $CHCl_3$ for 81:19 e.r.).

HPLC (Astec® Cellulose DMP column, 70:30 hexane–EtOH, 1.0 mL/min flow rate, 254 nm); t_r(e_1, major) = 16.0 min; t_r(e_2, minor) = 20.0 min.

* Vendor: AK Scientific.

1,3-Bis[(2,4-dimethoxyphenyl)methyl]-5-(4-iodophenyl)imidazolidine-2,4-dione 4v-e

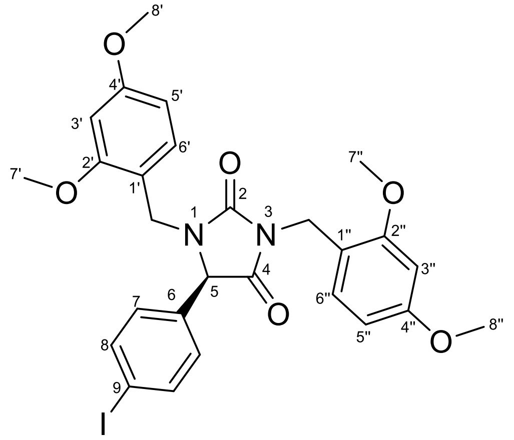

General procedure **A** was followed using 4-iodophenylglyoxal monohydrate **13i** (27 mg, 99 µmol,* 1.0 eq.) and 1,3-bis[(2,4-dimethoxyphenyl)methyl]urea **14c** (36 mg, 101 µmol, 1.0 eq.) for 20 h. Flash chromatography eluting with 70:30 petrol–EtOAc gave the *title compound* **17v-e** (58 mg, 96 µmol, 96%, 88:12 e.r.) as a colourless amorphous solid.

^{1}H NMR (600 MHz, $CDCl_3$): δ 7.71 (2H, d, *J* 8.4, 8-H), 7.15-7.10 (1H, m, 6″-H), 7.05 (1H, m, 6′-H), 6.93 (2H, d, *J* 8.4, 7-H), 6.44-6.37 (4H, m, 3′-H, 3″-H, 5′-H and 5″-H), 4.89 (1H, d, *J* 14.7, 1-NCH_AH_BAr), 4.73 (1H, d, *J* 14.9, 3-NCH_AH_BAr), 4.64 (1H, s, 5-H), 4.58 (1H, d, *J* 14.9, 3-NCH_AH_BAr), 3.90 (1H, d, *J* 14.7, 1-NCH_AH_BAr), 3.79 (3H, s, 8′-CH_3), 3.78 (3H, s, 8″-CH_3), 3.71 (3H, s, 7′-CH_3), 3.64 (3H, s, 7″-CH_3).

^{13}C NMR (150 MHz, $CDCl_3$ 1×OCH_3 not observed): δ 170.9 (4-C), 161.2 (4′-C), 160.7 (4″-C), 158.7 (2′-C), 158.4 (2″-C) 156.6 (2-C), 138.2 (8-C), 133.7 (6-C), 132.0 (6″-C), 130.3 (6′-C), 129.3 (7-C), 116.5 (1″-C), 116.1 (1′-C), 104.4 (5′-C), 104.2 (5″-C), 98.6 (3′-C or 3″-C), 98.5 (3′-C or 3″-C), 94.7 (9-C), 62.6 (5-C), 55.6 (OCH_3), 55.5 (OCH_3), 55.3 (OCH_3), 39.9 (1-NCH_2Ar), 38.0 (3-NCH_2Ar).

IR ν_{max}(neat)/cm^{-1} 2941, 1770 (C=O), 1707 (C=O), 1613, 1587, 1511, 1442.

HRMS (ESI): $C_{27}H_{28}FN_2O_6$ $[M+H]^+$; calculated: 603.0987, found 603.0995.

$[\alpha]_D^{23}$ = −108 (c. 0.3, $CHCl_3$ for 88:12 e.r.).

HPLC (Astec® Cellulose DMP column, 70:30 hexane–EtOH, 1.0 mL/min flow rate, 254 nm); t_r(e_1, major) = 19.6 min; t_r(e_2, minor) = 27.0 min.

* mmol estimated based on the molecular weight of the monohydrate form of the glyoxal.

1,3-[(2,4-Dimethoxyphenyl)methyl]-5-phenylimidazolidine-2,4-dione 17w

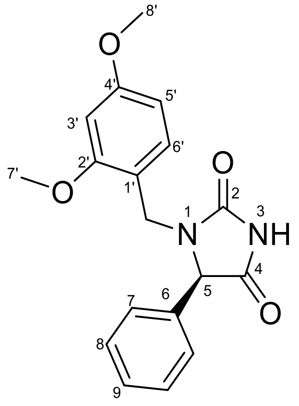

General procedure **B** was followed using phenylglyoxal monohydrate **13a*** (15 mg, 99 μmol, 1.0 eq.) and 1-[(2,4-dimethoxyphenyl)methyl]urea **14d** (22 mg, 101 μmol, 1.0 eq.) for 72 h. Flash chromatography eluting with 75:25 petrol–EtOAc gave the *title compound* **17w** (11 mg, 35 μmol, 35%, 69:31 e.r.) as a colourless amorphous solid.

R*f* 0.16 (80:20 petrol–EtOAc).

^{1}H NMR (600 MHz, $CDCl_3$): δ 9.08 (1H, br. s, NH), 7.44-7.36 (3H, m, 8-H and 9-H), 7.23 (2H, d, *J* 7.9, 7-H), 7.06 (1H, d, *J* 8.9, 6′-H) 6.43-6.38 (2H, m, 3′-H, and 5′-H), 4.89 (1H, d, *J* 14.7, NCH_AH_BAr), 4.77 (1H, s, 5-H), 3.89 (1H, d, *J* 14.7, NCH_AH_BAr), 3.79 (3H, s, 8′-CH_3), 3.68 (3H, s, 7′-CH_3).

^{13}C NMR (150 MHz, $CDCl_3$, 1×Ar not observed): δ 172.4 (4-C), 161.2 (4′-C), 158.7 (2′-C), 156.6 (2-C), 133.1 (6-C), 131.9 (6′-C), 129.2 (8-C), 127.5 (7-C), 116.1 (1′-C), 104.3 (5′-C), 98.6 (3′-C), 65.0 (5-C), 55.5 (OCH_3), 55.3 (OCH_3), 39.5 (1-NCH_2Ar).

IR ν_{max}(neat)/cm^{-1} 3279 (NH), 1770 (C=O), 1701 (C=O), 1612, 1586, 1438, 1178.

HRMS (ESI): $C_{18}H_{19}N_2O_4$ $[M+H]^+$; calculated: 327.1345, found 327.1337

$[\alpha]_D^{21}$ = −87 (c. 0.2, $CHCl_3$ for 69:32 e.r.)

HPLC (CHIRALPAK® AS-3R, 50:50 MeCN–H_2O, 0.5 mL/min flow rate, 280 nm); t_r(e_1, major) = 7.5 min; t_r(e_2, minor) = 8.2 min.

* Vendor: Alfa Aesar.

1,3-Dimethyl-5-phenyl-2-sulfanylideneimidazolidin-4-one 17x

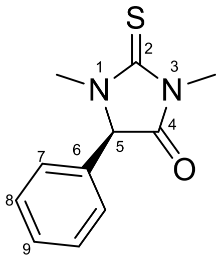

General procedure **A** was followed starting with phenylglyoxal monohydrate **13a*** (15 mg, 99 μmol, 1.0 eq.) and 1,3-dimethylthiourea **14e†** (10 mg, 99 μmol, 1.0 eq.) at 60 ºC for 0.5 h. Flash chromatography eluting with 90:10 petrol–EtOAc gave the *title compound* **17x** (21 mg, 95 μmol, 95%, 71:29 e.r.) as a colourless amorphous solid.

R*f* 0.32 (90:10 petrol–EtOAc).

^{1}H NMR (600 MHz, $CDCl_3$): δ 7.49-7.35 (3H, m, 8 and 9-H), 7.23-6.96 (2H, m, 7-H), 4.89 (1H, s, 5-H), 3.31 (3H, s, 3-NC*H*$_3$), 3.20 (3H, s, 1-NC*H*$_3$).

^{13}C NMR (100 MHz, $CDCl_3$): δ 183.6 (2-C), 172.1 (4-C), 131.9 (6-C), 129.8 (9-C), 129.7 (8-C), 127.4 (7-C), 68.2 (5-C), 32.8(1-N*C*H$_3$), 28.8 (3-N*C*H$_3$).

IR ν_{max}(neat)/cm^{-1} 2927, 1741 (C=O), 1495, 1371, 1322, 1136, 985.

HRMS (ESI): $C_{11}H_{13}N_2OS$ $[M+H]^+$; calculated: 221.0743, found 221.0755.

$[\alpha]_D^{23}$ = −93 (c. 0.1, $CHCl_3$ for 91:9 e.r.).

HPLC (CHIRALPAK® AS-3R, 50:50 MeCN–H_2O, 0.5 mL/min flow rate, 254 nm); t_r(e_1, major) = 8.7 min; t_r(e_2, minor) = 10.5 min.

* Vendor: Alfa Aesar.

† Vendor: AK Scientific.

11. Alkylglyoxal substrate scope for asymmetric hydantoin synthesis

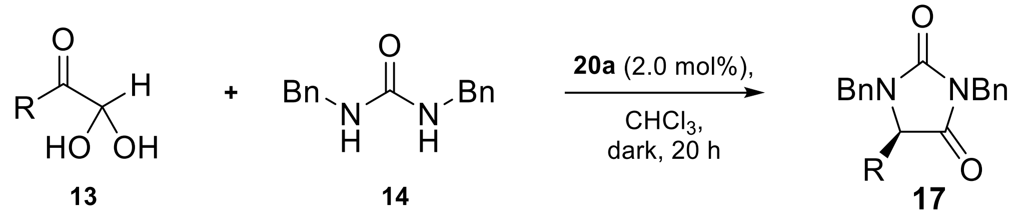

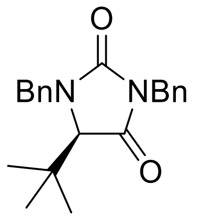

S17y, 89%, 68:32 e.r.[a,b]
(89%, 65:35 e.r.)[a,b,c]
(89%, 64:36 e.r.)[a,d]

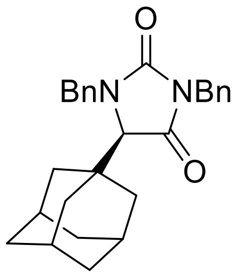

S17z, 97%, 72:28 e.r.
(95%, 51:49 e.r.)[c]

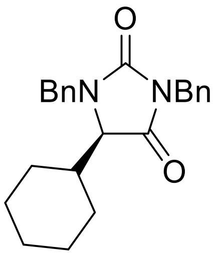

S17aa, 96%, 56:44 e.r.
(95%, 51:49 e.r.)[c]

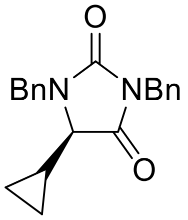

S17ab, 92% 63:37 e.r.[a]
(97%, 65:35 e.r.)[c]
(93%, 65:35 e.r.)[a,d]

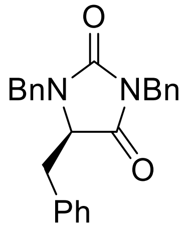

S17ac, 95% 62:38 e.r[a]

Scheme S1: Phosphoric acid-catalysed condensation of alkylglyoxals with 1,3 dibenzylurea to give enantioenriched hydantoins. *Standard reaction conditions:* **13** (0.1 mmol), **14** (0.1 mmol), catalyst **20a** (2.0 mol%), $CHCl_3$ (0.1 M), dark, Ar, rt, no stirring‡, 20 h, (see General Procedure A). *a:* **13** (0.15 mmol) used; *b:* 72 h; *c:* catalyst **19b** (2.0 mol%); *d:* 60 °C for 2 h. ‡Reaction performed inside a Wilmad® Z271993 5 mL borosilicate NMR tube from Sigma Aldrich.

1,3-Dibenzyl-5-tert-butylimidazolidine-2,4-dione S17y

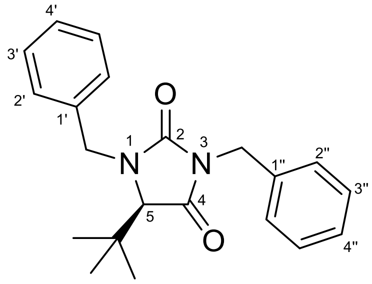

General procedure **A** was followed starting with 3,3-dimethyl-2-oxobutanal **S13y** (18 mg, 150 μmol,* 1.5 eq.) and 1,3-dibenzylurea **14a** (24 mg, 100 μmol, 1.0 eq.) for 72 h. Flash chromatography eluting with 90:10 hexane–EtOAc gave the *title compound* **S17y** (30 mg, 89 μmol, 89%, 68:32 e.r.) as a colourless amorphous solid.

$\mathbf{R}_f$ 0.22 (90:10 hexane–EtOAc).

^{1}H NMR (600 MHz, $CDCl_3$): δ 7.40-7.37 (2H, m, 2''-H), 7.36-7.28 (6H, m, 3'-H, 3''-H, 4'-H and 4''-H), 7.13-7.10 (2H, m, 2'-H), 5.29 (1H, d, *J* 15.4, 1-NCH_AH$_B$Ph), 4.71 (1H, d, *J* 14.4, 3-NCH_AH$_B$Ph), 4.66 (1H, d, *J* 14.4, 3-NCH$_A$$H_B$Ph), 4.19 (1H, d, *J* 15.4, 1-NCH$_A$$H_B$Ph), 3.39 (1H, s, 5-H), 1.00 (9H, s, C(CH_3)$_3$).

^{13}C NMR (150 MHz, $CDCl_3$, 1×Ar-C not observed) δ 172.5 (4-C), 159.1 (2-C), 136.2 (1''-C), 136.0 (1'-C), 129.1 (Ar-C), 128.7 (Ar-C), 128.6 (Ar-C), 128.1 (Ar-C), 128.0 (Ar-C), 66.8 (5-C), 48.8 (1-N*C*H$_2$Ph), 42.7 (3-N*C*H$_2$Ph), 36.7 (*C*$_q$(CH$_3$)$_3$), 26.6 (C$_q$(*C*H$_3$)$_3$).

IR ν_{max}(film from $CDCl_3$)/cm^{-1} 2925, 2853, 1764 (C=O), 1703 (C=O), 1495, 1443, 1419

HRMS (ESI): $C_{21}H_{25}N_2O_2$ [M+H]$^+$; calculated 337.1911, found 337.1921.

$[\alpha]_D^{21}$ = −12 (c. 0.1, $CHCl_3$, 68:32 e.r.).

HPLC (Astec® Cellulose DMP column, 94:4 hexane–EtOH, 1.0 mL/min flow rate, 220 nm), t_r(e_1, major) = 8.0 min; t_r(e_2, minor) = 9.2 min.

* mmol estimated based on the molecular weight of the monohydrate form of the glyoxal.

5-(Adamantan-1-yl)-1,3-dibenzylimidazolidine-2,4-dione S17z

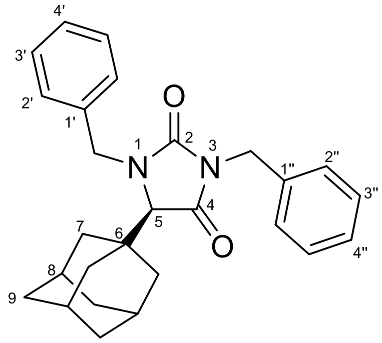

General procedure **A** was followed starting with 2-(adamantan-1-yl)-2-oxoacetaldehyde **S13z** (22 mg, 102 μmol,* 1.0 eq,) and 1,3-dibenzylurea **14a** (24 mg, 100 μmol, 1.0 eq.) for 20 h. Flash chromatography eluting with 90:10 hexane–EtOAc gave the *title compound* **S17z** (40 mg, 98 μmol, 98%, 74:26 e.r.) as a colourless amorphous solid.

$\mathbf{R}_f$ 0.42 (90:10 hexane–EtOAc).

^{1}H NMR (600 MHz, $CDCl_3$): δ 7.37 (2H, dd, *J* 6.8, 1.7, 2"-H), 7.33-7.29 (2H, m, 4'-H and 4"-H), 7.29-7.26 (4H, m, 3'-H and 3"-H), 7.10 (2H, dd, *J* 7.4, 2.2, 2'-H), 5.29 (1H, d, *J* 15.3, 1-NCH_AH$_B$Ph), 4.69 (1H, d, *J* 14.5, 3-NCH_AH$_B$Ph), 4.64 (1H, d, *J* 14.5, 3-NCH$_A$$H_B$Ph), 4.16 (1H, d, *J* 15.3, 1-NCH$_A$$H_B$Ph), 3.24 (1H, s, 5-H), 1.99-1.93 (3H, m, 8-H), 1.84 (3H, dq, *J* 12.1, 2.7, 7-H$_A$), 1.68 (3H, d, *J* 12.5, 9-H$_A$), 1.59 (3H, d, *J* 12.5, 9-H$_B$), 1.49 (3H, dq, *J* 12.1, 2.7, 7-H$_B$).

^{13}C NMR (150 MHz, $CDCl_3$) δ 172.3 (4-C), 159.4 (2-C), 136.3 (1"-C), 136.0 (1'-C), 129.1 (3'-C or 3"-C), 128.7 (3'-C or 3"-C), 128.6 (2"-C), 128.3 (2'-C), 128.2 (4'-C), 127.9 (4"-C), 67.4 (5-C), 49.6 (1-N*C*H$_2$Ph), 42.7 (3-N*C*H$_2$Ph), 39.02 (6-C), 38.4 (7-C), 36.6 (9-C), 28.2 (8-C).

IR ν_{max}(film from $CDCl_3$)/cm^{-1} 2925, 2853, 1764 (C=O), 1703 (C=O), 1495, 1443, 1420.

HRMS (ESI): $C_{27}H_{31}N_2O_2$ $[M+H]^+$; calculated 415.2380, found 415.2384.

$[\boldsymbol{\alpha}]_{\mathbf{D}}^{\mathbf{21}}$ = −13 (c. 0.2, $CHCl_3$, 72:28 e.r.).

HPLC (Astec® Cellulose DMP column, 80:20 hexane–EtOH, 1.0 mL/min flow rate, 254 nm), t_r(e_1, major) = 6.4 min; t_r(e_2, minor) = 7.1 min.

* mmol estimated based on the molecular weight of glyoxal form.

1,3-Dibenzyl-5-cyclohexylimidazolidine-2,4-dione S17aa

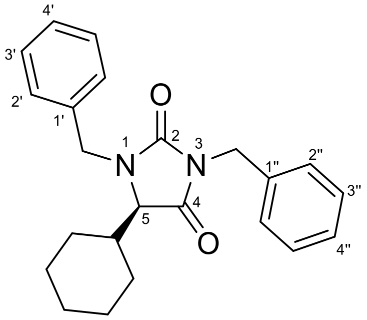

General procedure **A** was followed starting with 2-cyclohexyl-2-oxoacetaldehyde hemihydrate **S13aa** (15 mg, 101 µmol,* 1.0 eq.) and 1,3-dibenzylurea **14a** (24 mg, 100 µmol, 1.0 eq). Flash chromatography eluting with 90:10 hexane–EtOAc gave the *title compound* **S17aa** (35 mg, 96 µmol, 96%, 56:44 e.r.) as a colourless amorphous solid.

R*f* 0.24 (90:10 hexane–EtOAc).

^{1}H NMR (600 MHz, $CDCl_3$): δ 7.38 (2H, dd, *J* 8.2, 1.4, 2''-H), 7.35-7.26 (6H, m, 3'-H, 3''-H 4' and 4''-H), 7.27-7.18 (2H, dd, *J* 8.2, 1.4, 2'-H), 5.04 (1H, d, *J* 15.3, 1-NCH_AH$_B$Ph), 4.70 (1H, d, *J* 14.5, 3-NCH_AH$_B$Ph), 4.64 (1H, d, *J* 14.5, 3-NCH$_A$$H_B$Ph), 4.11 (1H, d, *J* 15.3, 1-NCH$_A$$H_B$Ph), 3.61 (1H, d, *J* 3.0, 5-H), 1.81-1.77 (1H, m, Cy-CH), 1.77-1.71 (1H, m, Cy-CH_2), 1.71-1.65 (1H, m, Cy-CH_2), 1.65-1.56 (2H, m, Cy-CH_2), 1.53-1.46 (1H, m, Cy-CH_2), 1.45-1.40 (1H, m, Cy-CH_2), 1.22-1.01 (3H, m, Cy-CH_2), 0.96-0.80 (1H, m, Cy-CH_2).

^{13}C NMR (150 MHz, $CDCl_3$) δ 172.2 (4-C), 157.1 (2-C), 136.4 (1''-C), 135.9 (1'-C), 129.1 (3'-C or 3''-C), 128.7 (3'-C or 3''-C), 128.6 (2''-C), 128.3 (2'-C), 128.2 (4'-C) 127.9 (4''-C), 63.3 (5-C), 45.0 (1-N*C*H_2Ph), 42.6 (3-N*C*H_2Ph), 38.5 (Cy-*C*H), 28.0 (Cy-*C*H_2), 27.0 (Cy-*C*H_2), 26.5 (Cy-*C*H_2), 26.2 (Cy-*C*H_2), 26.1 (Cy-*C*H_2).

IR ν_{max}(film from $CDCl_3$)/cm^{-1} 2925, 2853, 1764 (C=O), 1701 (C=O), 1495, 1443, 1420.

HRMS (ESI): $C_{23}H_{27}N_2O_2$ [M+H]$^+$; calculated 363.2067, found 363.2079.

$[\alpha]_D^{21}$ = −7.2 (c. 0.2, $CHCl_3$, 56:44 e.r.).

HPLC (Astec® Cellulose DMP column, 25 cm, 80:20 hexane–EtOH, 1.0 mL/min flow rate, 254 nm), t_r(e_1, major) = 7.3 min; t_r(e_2, minor) = 7.8 min.

* mmol estimated based on the molecular weight of the hemihydrate form of the glyoxal.

1,3-Dibenzyl-5-cyclopropylimidazolidine-2,4-dione S17ab

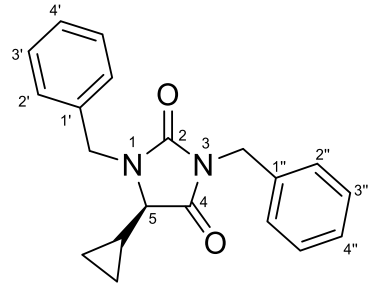

General procedure **A** was followed starting with 2-cyclopropyl-2-oxoacetaldehyde **S13ab** (18 mg, 150 µmol,* 1.5 eq.) and 1,3-dibenzylurea **14a** (24 mg, 100 µmol, 1.0 eq) for 20 h. Flash chromatography eluting with 90:10 hexane–EtOAc gave the *title compound* **S17ab** (30 mg, 92 µmol, 92%, 62:38 e.r.) as a colourless amorphous solid.

R*f* 0.19 (90:10 hexane–EtOAc).

^{1}H NMR (600 MHz, $CDCl_3$): δ 7.41 (2H, dd, *J* 8.3, 1.4, 2"-H), 7.35- 7.32 (2H, m, 4'-H and 4"-H) 7.31-7.25 (4H, m, 3'-H and 3"-H), 7.21 (2H, dd, *J* 7.0, 2.1, 2"-H), 5.07 (1 H, d, *J* 15.4, 1-NCH_AH$_B$Ph), 4.73 (1H, d, *J* 14.5, 3-NCH_AH$_B$Ph), 4.66 (1H, d, *J* 14.5, 3-NCH$_A$$H_B$Ph), 4.30 (1H, d, *J* 15.4, 1-NCH$_A$$H_B$Ph), 3.02 (1H, d, *J* 9.4, 5-H), 0.85-0.77 (1H, m, cyclopropyl C*H*), 0.67-0.57 (2H, m, cyclopropyl CH_2), 0.52-0.45 (1H, m, cyclopropyl CH_2), 0.28-0.21 (1H, m, cyclopropyl CH_2).

^{13}C NMR (150 MHz, $CDCl_3$) δ 172.1 (4-C), 156.2 (2-C), 136.4 (1"-C), 136.3 (1'-C), 129.0 (3'-C or 3"-C), 128.8 (3'-C or 3"-C), 128.7 (2"-C), 128.0 (3 peaks, 4', 4" and 2'-C), 63.1 (5-C), 44.6 (1-N*C*H_2Ph), 42.7 (3-N*C*H_2Ph), 11.2 (cyclopropyl *C*H), 3.7 (cyclopropyl *C*H_2), 1.0 (cyclopropyl *C*H_2).

IR ν_{max}(film from $CDCl_3$)/cm^{-1} 2962, 1764 (C=O), 1702 (C=O), 1495, 1442, 1385, 1336.

HRMS (ESI): $C_{20}H_{21}N_2O_2$ $[M+H]^+$; calculated 322.1676 found 322.1679.

$[\alpha]_D^{21}$ = −5.0 (c. 0.1, $CHCl_3$, 63:37 e.r.).

HPLC (CHIRALPAK® AS-3R, 60:40 MeCN–H_2O, 0.5 mL/min flow rate, 220 nm); t_r(e_1, major) = 8.9 min; t_r(e_2, minor) = 9.8 min.

* mmol estimated based on the molecular weight of the monohydrate form of the glyoxal.

1,3,5-Tribenzylimidazolidine-2,4-dione S17ac

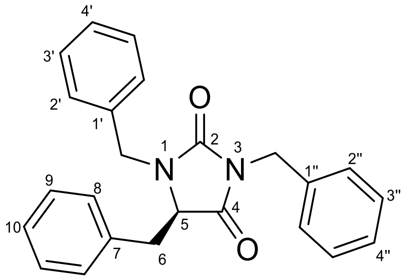

General procedure **A** was followed starting with 2-oxo-3-phenylpropanal **S13ac** (22.3 mg, 150 μmol,[*] 1.5 eq.) and 1,3-dibenzylurea **14a** (24 mg, 100 μmol, 1.0 eq.) for 20 h. Flash chromatography eluting with DCM gave the title compound **S17ac** (36 mg, 95 μmol, 95%, 62:38 e.r.) as a colourless amorphous solid.

R*f* 0.45 (DCM).

^{1}H NMR (600 MHz, $CDCl_3$): δ 7.35-7.29 (3H, m, 10-H, 4'-H and 4''-H), 7.25-7.19 (4H, m, 3'-H and 3''-H), 7.19-7.15 (2H, m, 9-H), 7.15-7.11 (2H, m, 2'-H), 7.09-7.05 (2H, m, 2''-H), 7.05-7.01 (2H, m, 8-H), 5.15 (1H, d, *J* 15.1, 1-NC$\mathit{H_A}$$H_B$Ph), 4.62 (1H, d, *J* 14.6, 3-NC$\mathit{H_A}$$H_B$Ph), 4.51 (1H, d, *J* 14.6, 3-NC$H_A$$\mathit{H_B}$Ph), 4.02 (1H, t, *J* 4.7, 5-H), 3.97 (1H, d, *J* 15.1, 1-NC$H_A$$\mathit{H_B}$Ph), 3.15 (1H, dd, *J* 14.5, 4.7, 5-CC$\mathit{H_A}$$H_B$Ph), 3.11 (1H, dd, *J* 14.5, 4.7, 5-CC$H_A$$\mathit{H_B}$Ph).

^{13}C NMR (150 MHz, $CDCl_3$) δ 172.1 (4-C), 156.6 (2-C), 135.8 (1''-C), 135.5 (1'-C), 134.4 (7-C), 129.6 (8-C), 129.1 (9-C), 129.8 (3''-C or 3'-C), 128.7 (3''-C or 3'-C), 128.4 (2'-C), 128.3 (4'-C), 128.1 (2''-C), 127.7 (Ar-C), 127.5 (Ar-C), 59.3 (5-C), 45.1 (1-N*C*H_2Ph), 42.5 (3-N*C*H_2Ph), 34.9 (5-C*C*H_2Ph).

IR ν_{max}(film from $CDCl_3$)/cm^{-1} 2924, 1764 (C=O), 1704 (C=O), 1494, 1445, 1418, 1143.

HRMS (ESI): $C_{24}H_{23}N_2O_2$ [M+H]$^+$; calculated 371.1754, found 371.1759.

$[\alpha]_D^{21}$ = −7.1 (c. 0.1, $CHCl_3$, 62:38 e.r.).

HPLC (Astec® Cellulose DMP column, 80:20 hexane–EtOH, 1.0 mL/min flow rate, 254 nm), t_r(e_1, minor) = 11.5 min; t_r(e_2, major) = 12.2 min.

The spectral data are consistent with the literature values.[9]

[*] mmol estimated based on the molecular weight of the dioxo form of the glyoxal.

12. Reactions of enantioenriched hydantoins

12.1 Attempted deprotection of 1,3-*di*DMB hydantoin

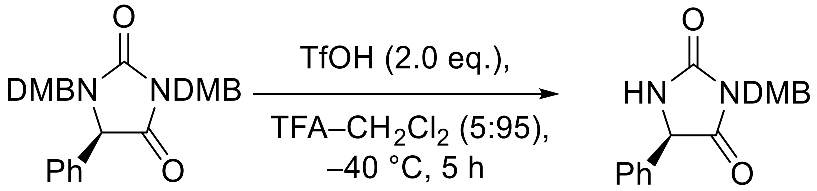

17v-a, 89:11 e.r. **22v-a**, 87%, 85:15 e.r.

3-[(2,4-Dimethoxyphenyl)methyl]-5-phenylimidazolidine-2,4-dione 22aa

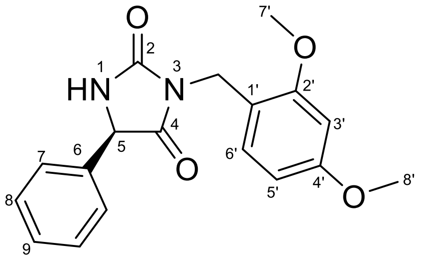

To a stirred solution of 1,3-bis[(2,4-dimethoxyphenyl)methyl]-5-phenylimidazolidine-2,4-dione **17v-a** (47 mg, 99 µmol, 1.0 eq.) in 5 v/v% TFA in CH_2Cl_2 (1.0 mL) at – 40 °C (MeCN/dry ice bath) was added dropwise a solution of TfOH (37 µl, 201 µmol, 2.0 eq.) in CH_2Cl_2 (1.0 mL). The reaction mixture was stirred for 5 h. After the disappearance of the starting material spot by TLC, sat. aq. $NaHCO_3$ solution (5 mL) was added at 0 °C and the resulting solution was extracted with CH_2Cl_2 (3 × 15 mL). The combined organic layer was washed with brine (10 mL), dried over $MgSO_4$, filtered, and concentrated. Flash chromatography eluting with 99:1 CH_2Cl_2–MeOH gave the *title compound* **22v-a** (28 g, 87 µmol, 87%, 85:15 e.r.) as a colourless solid.

R*f* 0.21 (99:1 CH_2Cl_2–MeOH).

^{1}H NMR (600 MHz, $CDCl_3$): δ 7.44-7.32 (5H, m, 7-H, 8-H and 9-H), 7.15-7.05 (1H, d *J* 9.0, 6′-H), 6.45-6.36 (2H, m, 3′-H and 5′-H), 6.16 (1H, br. s, NH), 5.03 (1H, s 5-H), 4.71 (1H, d, *J* 15.1, 3-NC$H_A$$H_B$Ar), 4.60 (1H, d, *J* 15.1, 3-NC$H_A$$H_B$Ar), 3.77 (3H, s, 7′-$CH_3$), 3.71 (3H, s, 8′-$CH_3$).

^{13}C NMR (150 MHz, $CDCl_3$): δ 171.8 (4-C), 160.7 (4′-C), 158.4 (2′-C) 157.7 (2-C), 134.7 (6-C), 130.2 (6′-C), 129.2 (8-C), 129.1 (9-C), 126.6 (7-C), 116.2 (1′-C), 104.1 (5′-C), 98.6 (3′-C), 60.8 (5-C), 55.5 (2 peaks, O*C*H_3), 37.8 (3-N*C*H_2Ar).

IR ν_{max} (film from $CDCl_3$)/cm^{-1} 3301 (NH), 1775 (C=O), 1706 (C=O), 1508, 1442, 1208, 1034.

HRMS (ESI): $C_{18}H_{18}N_2NaO_4$ $[M+Na]^+$; calculated: 349.1159, found 349.1174

$[\alpha]_D^{21}$ = −109 (c. 0.1, $CHCl_3$ for 85:15 e.r.)

HPLC (CHIRALPAK® AS-3R, 50:50 MeCN–H_2O, 0.5 mL/min flow rate, 280 nm); t_r(e_1, major) = 9.0 min; t_r(e_2, minor) = 10.6 min.

12.2 Synthesis of an enantioenriched vicinal diamine

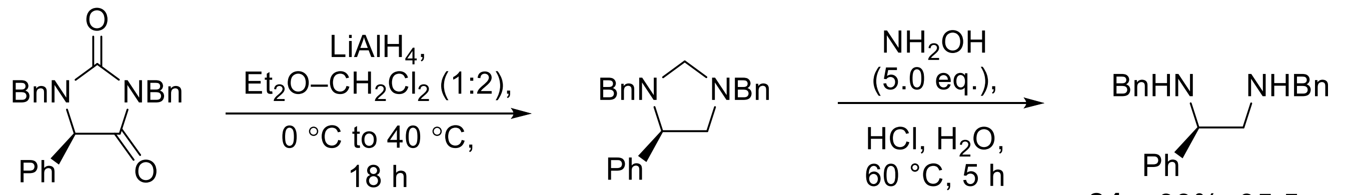

17a, 95:5 e.r. **23a**, 95%, 95:5 e.r. **24a**, 93%, 95:5 e.r.

1,3-Dibenzyl-4-phenylimidazolidine 23a

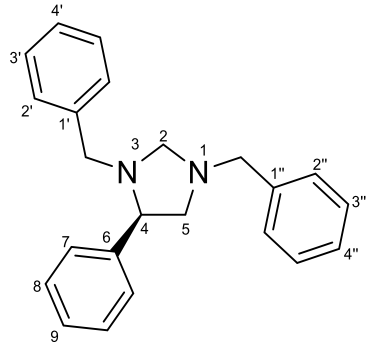

To a suspension of $LiAlH_4$ (72 mg, 20 mmol, 20 eq.) in Et_2O (1.0 mL) at 0 °C, was added dropwise a solution of 1,3-dibenzyl-5-phenylimidazolidine-2,4-dione **17a** (36 mg, 99 μmol, 1.0 eq.) in DCM (2.0 mL). The reaction mixture was heated to 40 °C for 18 h. After the disappearance of the starting material and hemiaminal spots by TLC, unreacted $LiAlH_4$ was decomposed by carefully adding aq. NaOH (1.0 M, 5.0 mL) at 0 °C. The residue was diluted with H_2O (10 mL) and extracted DCM (3 × 15 mL). The combined organic layer was washed with brine (10 mL), dried over $MgSO_4$, filtered, and concentrated. Flash chromatography eluting with 90:10 petrol–EtOAc gave the *title compound* **23a** (31 mg, 95 μmol, 95%, e.r. 95:5) as a colourless oil.

R*f* 0.51 (8:2 petrol–EtOAc).

^{1}H NMR (600 MHz, $CDCl_3$): δ 7.58-7.52 (2H, m, 7-H), 7.43-7.28 (12H, m, Ar-H), 7.28-7.23 (1H, m, Ar-H) 3.94 (1H, d, *J* 13.5 3-NCH_AH$_B$Ph), 3.92 (1H, dd, *J* 8.6, 7.2, 4-H), 3.85 (1H, d, *J* 5.6, 2-H$_A$), 3.80 (1H, d, *J* 13.0, 1-NCH_AH$_B$Ph), 3.77 (1H, d, *J* 13.0, 1-NCH$_A$$H_B$Ph), 3.51 (1H, d, *J* 5.6, 2-H$_B$), 3.42 (1H, d, *J* 13.5, 3-NCH$_A$$H_B$Ph), 3.34 (1H, dd, *J* 9.6, 7.2, 5-H$_A$), 2.71 (1H, dd, *J* 9.6, 8.6, 5-H$_B$).

^{13}C NMR (150 MHz, $CDCl_3$): δ 141.7 (6-C), 139.5 (1'-C), 139.0 (1''-C), 128.8 (Ar-C), 128.6 (Ar-C), 128.5 (Ar-C), 128.4 (Ar-C), 128.3 (Ar-C), 127.6 (Ar-C), 127.5 (Ar-C), 127.2 (Ar-C), 127.0 (Ar-C), 76.5 (2-C), 68.2 (4-C), 62.2 (5-C), 60.1 (1-N*C*H_2Ph), 57.2 (3-N*C*H_2Ph).

IR ν_{max}(film from $CDCl_3$)/cm^{-1} 2796, 1492, 1452, 1354, 1026, 734, 696.

HRMS (ESI): $C_{23}H_{25}N_2$ $[M+H]^+$; calculated 329.2012, found 329.2022.

$[\alpha]_D^{21}$ = −89 (c. 0.2, $CHCl_3$ for 95:5 e.r.).

HPLC (Astec® Cellulose DMP column, 80:20 hexane–*i*PrOH, 0.5 mL/min flow rate, 254 nm); $t_r(e_1$, major) = 9.1 min; $t_r(e_2$, minor) = 9.4 min.

Benzyl[2-(benzylamino)-1-phenylethyl]amine 24a

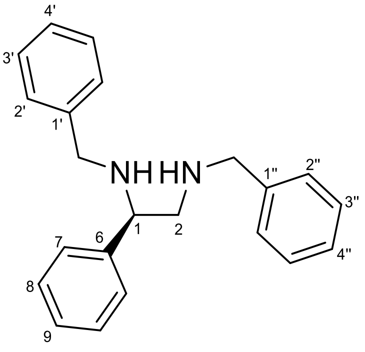

To a stirred solution of 1,3-dibenzyl-4-phenylimidazolidine **23a** (31 mg, 95 µmol, 1.0 eq.) in aq. HCl (0.01 w/w%, 2.8 mL) was added hydroxylamine hydrochloride (32 mg, 475 µmol, 5.0 eq.). The reaction mixture was heated to 60 °C for 5 h. The mixture was diluted with aq. HCl solution (1.0 N, 7.0 mL) and washed with CH_2Cl_2 (3 ×15 mL). The aqueous layer was basified with aq. NaOH (5.0 N) until the pH reached 11-13 (litmus paper), then extracted with CH_2Cl_2 (5 ×15 mL). The combined organic layer was washed with brine (10 mL), dried over $MgSO_4$, filtered, and concentrated. Flash chromatography eluting with 60:40 petrol–EtOAc gave the title compound **24a** (28 mg, 88 µmol, 93%, e.r. 95:5) as a colourless oil.

R*f* 0.21 (60:40 petrol–EtOAc).

^{1}H NMR (600 MHz, $CDCl_3$): δ 7.39-7.22 (15H, m, Ar-H), 3.80-3.77 (1H, m, 1-H), 3.77 (2H, s, NC*H$_2$*Ph), 3.72 (1H, d, *J* 13.3, NC*H$_A$*H$_B$Ph), 3.53 (1H, d, *J* 13.3, NCH$_A$*H$_B$*Ph), 2.89-2.74 (2H, m, 2-H), 2.31 (2H, br. s, 2 × N*H*).

^{13}C NMR (150 MHz, $CDCl_3$): δ 142.5 (Ar-C$_q$),140.7 (Ar-C$_q$), 140.3 (Ar-C$_q$), 128.7 (Ar-C), 128.5 (Ar-C), 128.5 (Ar-C), 128.4 (Ar-C), 128.2 (Ar-C), 127.5 (Ar-C), 127.5 (Ar-C), 127.1 (Ar-C), 127.0 (Ar-C), 61.8 (1-C), 56.1 (2-C), 53.8 (N*C*H$_2$Ph), 51.4 (N*C*H$_2$Ph).

IR ν_{max}(film from $CDCl_3$)/cm^{-1} 3304 (NH), 2801, 1492, 1451, 1118, 735, 697.

HRMS (ESI): $C_{22}H_{25}N_2$ $[M+H]^+$; calculated 317.2012, found 317.2019.

$[\alpha]_D^{21}$ = −46 (c. 0.1, $CHCl_3$ for 95:5 e.r.).

HPLC (Astec® Cellulose DMP column, 94:6 hexane–EtOH, 0.5 mL/min flow rate, 254 nm); t_r(e_1, minor) = 12.7 min; t_r(e_2, major) = 13.1 min.

The spectral data are consistent with the literature values.[11]

13. Synthesis of *cis*-diol 15a and 5-hydroxyhydantoin 21a

1,3-Dibenzyl-4,5-dihydroxy-4-phenylimidazolidin-2-one 15a

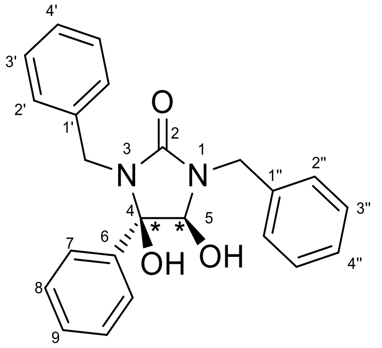

*Synthesis and isolation of racemic **15a***: A mixture of phenylglyoxal monohydrate 1a (152 mg, 1.00 mmol, 1.00 eq.), and 1,3-dibenzylurea 2a (240 mg, 1.00 mmol, 1.00 eq.) was stirred in CH_3CN (10 mL) for 18 h. An insoluble precipitate formed, and the reaction mixture was concentrated. The crude precipitate was recrystallised from EtOH (5.0 mL) to give *the title compound* **15a** (209 mg, 0.60 mmol, 60%) as colourless flakes.

Synthesis of enantioenriched **15a**: General procedure **B** was followed using phenylglyoxal monohydrate **13a** (85 mg, 0.50 mmol, 1.0 eq.) 1,3-dibenzyl urea **14a** (120 mg 0.50 mmol, 1.0 eq.) for 1 h. Flash chromatography eluting from 90:5 petrol–EtOAc to 70:30 petrol–EtOAc gave the *title compound* **15a** (112 mg, 0.30 mmol, 62%, 66:34 e.r.) as a colourless amorphous solid. *Relative stereochemistry as shown, but the exact identity of the major enantiomer was not determined.*

R_f 0.18 (60:40 hexane–EtOAc).

^{1}H NMR (600 MHz, DMSO-d_6):δ 7.32-7.28 (2H, m, Ar-H), 7.28-7.21 (8H, m, Ar-H), 7.20 (4H, d, *J* 4.5, Ar-H), 7.15 (1H, m, Ar-H), 6.66 (1H, d, *J* 6.5, C(O*H*)H), 6.10 (1H, s, C(O*H*)Ph), 4.70 (1H, d, *J* 15.3, 1-NCH_AH$_B$Ph), 4.52 (1H, d, *J* 6.5, C(OH)*H*), 4.22 (1H, d, *J* 15.7, 3-NCH_AH$_B$Ph), 4.09 (1H, d, *J* 15.3, 1-NCH_AH$_B$Ph), 4.00 (1H, d, *J* 15.7, 3-NCH$_A$$H_B$Ph).

^{13}C NMR (150 MHz, DMSO-d_6): δ 157.9 (2-C), 141.8 (6-C), 139.3 (1'-C), 137.8 (1"-C), 128.4 (Ar-C), 128.2 (Ar-C), 128.0 (Ar-C), 127.8 (Ar-C), 127.7 (2×Ar-C), 127.1 (Ar-C), 126.4 (Ar-C), 125.8 (7-C), 87.9 (4-C), 84.0 (5-C), 43.4 (3-NCH$_2$Ph), 43.2 (1-NCH$_2$Ph).

IR ν_{max}(neat)/cm^{-1} 3449 (OH), 3229 (OH), 3030, 1653 (C=O), 1476, 1449, 1419 1344.

HRMS (ESI): $C_{23}H_{23}N_2O_3$ $[M+H]^+$; calculated 375.1703, found 375.1703

$[\alpha]^{D}_{21}$ = +19 (c. 0.1, MeOH for 66:34 e.r.).

HPLC (CHIRALPAK® AS-3R, 50:50 MeCN–H_2O, 0.5 mL/min flow rate, 210 nm); t_r(e_1, minor) = 16.0 min; t_r(e_2, major) = 23.7 min.

1,3-Dibenzyl-5-hydroxy-5-phenylimidazolidine-2,4-dione 21a

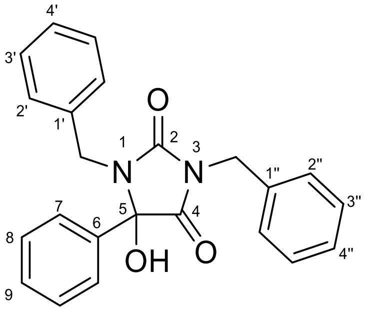

A mixture of phenylglyoxal monohydrate (15 mg, 99 µmol, 1.0 eq.), 1,3-dibenzylurea **14a** (24 mg, 99 µmol, 1.0 eq.) and catalyst **20a** were added to a 7.0 mL reaction vial equipped with a screw-cap lid equipped with a PTFE/silicone septum.* The reactants were stirred in CH_3CN† (0.1 M, 1.0 mL) under an air atmosphere and normal fumehood lighting (see General Experimental section). The reaction progress was monitored every 20 h by TLC. After 48 h, the reaction mixture was concentrated *in vacuo*. Flash chromatography from 95:5 hexane–EtOAc to 80:20 hexane–EtOAc gave the *title compound* **21a** (12 mg, 32 µmol, 32%, 50:50 e.r.) as a colourless amorphous solid.

R*f* 0.20 (80:20 hexane–EtOAc).

^{1}H NMR (400 MHz, $CDCl_3$) δ 7.40-7.28 (10H, m, Ar-H), 7.18 (5H, m, Ar-H), 4.63 (1H, d, *J* 14.6, 3-NCH_AH$_B$Ph), 4.58 (1H, d, *J* 14.6, 3-NCH$_A$$H_B$Ph), 4.56 (1H, s, O*H*), 4.55 (1H, d, *J* 14.9, 1-NCH_AH$_B$Ph), 4.10 (1H, d, *J* 14.9, 1-NCH$_A$$H_B$Ph).

^{13}C NMR (100 MHz, $CDCl_3$) δ 172.8 (4-C), 155.6 (2-C), 137.0 (1'-C), 135.7 (1''-C), 135.1 (6-C), 129.6 (Ar-C), 128.9 (Ar-C), 128.8 (Ar-C), 128.7 (Ar-C), 128.5 (Ar-C), 128.4 (Ar-C), 128.3 (Ar-C), 128.1 (Ar-C), 127.5 (Ar-C), 126.1 (Ar-C), 88.0 (5-C), 43.8 (1-N*C*H$_2$Ph), 42.6 (3-N*C*H$_2$Ph).

IR ν_{max}(film from $CDCl_3$)/cm^{-1} 3300 (OH), 1775 (C=O), 1704 (C=O), 1495, 1457, 1442, 1303.

HRMS (ESI) $C_{23}H_{21}N_2O_3$ [M+H]$^+$ calculated 373.1547, found: 373.1556.

HPLC (Astec® Cellulose DMP column, 80:20 hexane–EtOH, 1.0 mL/min flow rate, 210 nm), $t_r(e_1)$ = 7.3 min; $t_r(e_2,)$ = 8.0 min.

* Supelco-27151 vials and Supelco-27019 lids purchased from Sigma-Aldrich
† The solvent was not degassed with Ar.

14. Limitations of the phosphoric acid-catalysed hydantoin synthesis

14.1 Phenylglyoxal diethyl acetal

Table S16: Attempted condensation of phenylglyoxal diethyl acetal with 1,3-dibenzylurea **14a** in the presence of diphenylphosphoric acid.

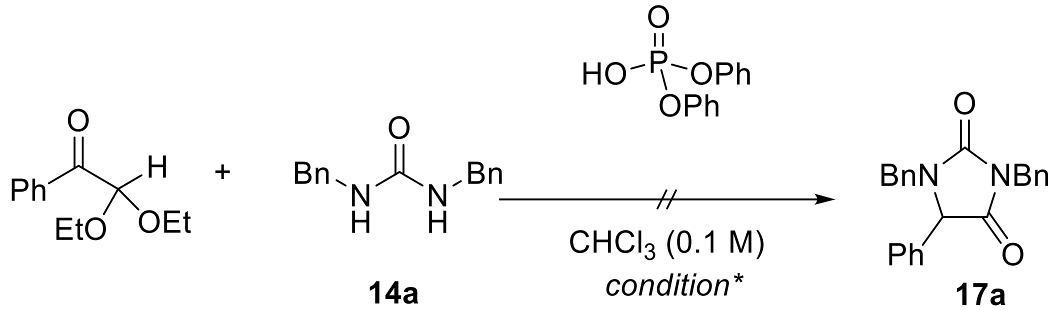

Entry[a]	H_2O (eq.)	Condition	Time /h	Observation
**1**	0	rt	24	no reaction[c]
**2**	1	rt	24	no reaction[c]
**3**	10	rt	24	no reaction[c]
**4**	1	50 ºC	24	no reaction[c]
**5**	10	50 ºC	24	no reaction[c]
**6**[b]	10	reflux	96	no reaction[c]

Reaction conditions: [a] Phenylglyoxal diethyl acetal (0.1 mmol), **14a** (0.1 mmol), diphenylphosphoric acid (5.0 mol%), $CHCl_3$ (1.0 mL). [b] Phenylglyoxal diethyl acetal (0.5 mmol), **14a** (0.5 mmol), diphenylphosphoric acid (5.0 mol%), $CHCl_3$ (5.0 mL). [c] Starting materials recovered. The reaction gave no detectable quantities of product **17a** as judged by analysis of crude reaction mixture by ^1H NMR (600 MHz).

14.2 Unsubstituted urea

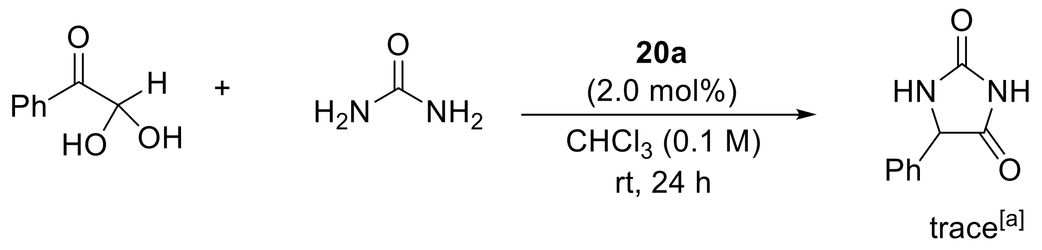

Scheme S2: Attempted condensation of phenylglyoxal monohydrate with unsubstituted urea (H_2NCONH_2) in the presence of catalyst **20a**. [a] Phenylglyoxal mostly polymerised; only a trace (~1% conversion) of the target hydantoin product was observed, as judged by analysis of the crude ^{1}H NMR spectrum (600 MHz). The % conversion was measured by integrating known product peaks versus mesitylene (internal standard, 3.5 μL, 0.33 eq.) in DMSO-d_6. Unsubstituted urea and the targeted unsubstituted hydantoin product were practically insoluble in $CHCl_3$.

14.3 Attempted glycouril synthesis

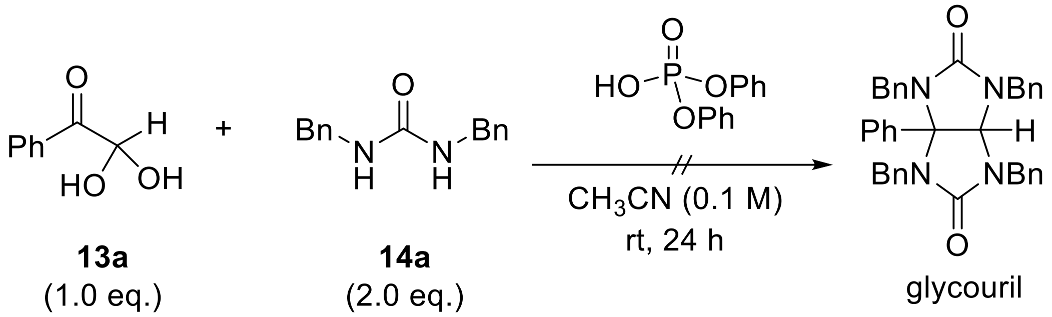

Scheme S3: Attempted glycouril synthesis *via* condensation of phenylglyoxal monohydrate (1.0 eq.) and 1,3-dibenzylurea (2.0 eq.) in the presence of diphenylphosphoric acid. [a] Phenylglyoxal monohydrate **13a** (0.1 mmol), **14a** (0.2 mmol), diphenylphosphoric acid (5.0 mol%), CH_3CN (1.0 mL). This reaction gave no detectable quantities of the glycouril product; the hydantoin **17a**, and unreacted **14a**, were observed in 1:1 ratio when the crude reaction mixture was analysed by ^{1}H NMR (600 MHz).

15. Attempted *N*-benzyl deprotection

15.1 Catalytic hydrogenation

Table S17: Catalytic hydrogenation.

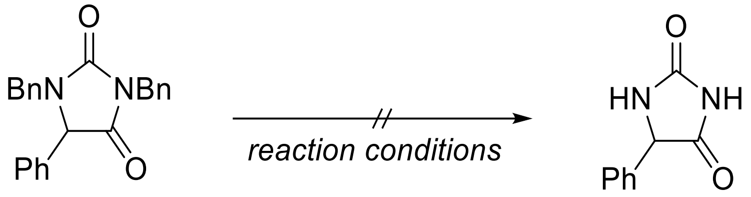

Entry[a]	Catalyst	H_2 source	Solvent	Conditions	Observation
1	10% Pd/C (1.0 mol% Pd)	H_2 (balloon)	MeOH	rt	no reaction[b]
2	10% Pd/C (7.0 mol% Pd)	HCO_2NH_4 (20.0 eq.)	MeOH	reflux	no reaction[b]
3	10% $Pd(OH)_2$/C (20% w/w %)	H_2 (balloon)	MeOH	rt	no reaction[b]
4	10% Pd/C (20 w/w %)	H_2 (40 psi)	MeOH	rt	no reaction[b]
5[c]	10% Pd/C (20 w/w %)	H_2 (40 psi)	MeOH	rt	no reaction[b]
6[c]	PtO_2 (20 mol%)	H_2 (40 psi)	MeOH	rt	aryl hydrogenation[d]

[a] The reaction was performed for 18 h. [b] The starting material was recovered after the reaction. [c] The reaction was performed with conc. HCl (10 eq.). [d] New aliphatic peaks were observed when the crude reaction mixture was analysed by ^{1}H NMR (600 MHz) and ^{13}C NMR (150 MHz) spectroscopy. These appear to be consistent with reduction of some of the Ph rings to Cy.

15.2 NBS-facilitated hydrolysis

Table S18: Attempted NBS-facilitated hydrolysis.

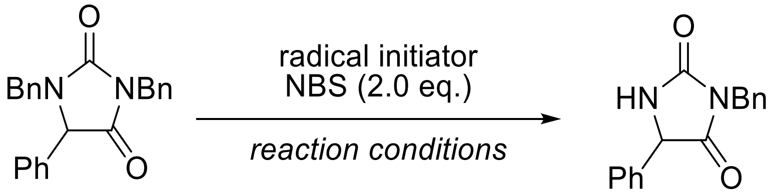

Entry	**Radical initiator** (mol%)	Solvent	T / °C	t /h	Comments
1	AIBN (1.0 M in PhMe, 10 mol%)	PhCl	130 °C	18	~10% conversion[a]
2	ACHN (10 mol%)	PhCl	130 °C	18	complex mixture
3	NMA (20 mol%)	$CHCl_3$	rt	6	complex mixture

[a] After 18 h, integration and analysis of the crude ^{1}H NMR spectra (600 MHz) showed a 90:10 ratio of starting material to product.

16. Synthesis of substituted ureas

General procedure C: Following modification of a procedure reported by Xu,[12] to a stirred mixture of urea (1.0 eq.) and aldehyde (2.0 eq.) in AcOH (1.0 M) was added TMSCl (1.0 eq.). The reaction mixture was stirred for 18 h, then $NaBH_4$ (2.00 eq.) was added portion wise over 0.5 h. After stirring for 1 h, the reaction mixture was concentrated (with addition of H_2O; 2 × 20 mL)* to provide a solid precipitate, which was purified as described.

1,3-Dibenzylurea 14a

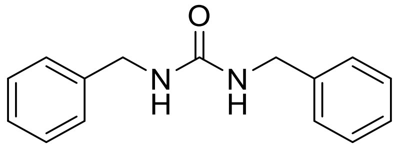

General procedure **C** was followed using urea (1.2 g, 20 mmol, 1.0 eq.), benzaldehyde (4.1 mL, 40 mmol, 2.0 eq.) and TMSCl (2.5 mL, 20 mmol, 1.0 eq.) in AcOH (20 mL) followed by the addition of $NaBH_4$ (1.51 g, 40.0 mmol, 2.00 eq.). The precipitate after AcOH evaporation was suspended in H_2O (20 mL) and basified with aq. NaOH (5 N) until the pH reached 9-10 (litmus paper). The crude residue was washed with ice-cold H_2O (20 mL) using Büchner funnel fitted with filter paper and recrystallised from EtOH (20 mL) to afford the title compound **14a** (2.60 g, 10.8 mmol, 54%) as colourless needles.

^{1}H NMR (400 MHz, DMSO-d_6): δ 7.36-7.28 (4H, m, Ar-H), 7.27-7.17 (6H, m, Ar-H), 6.45 (2H, t, *J* 6.0, N*H*CH_2), 4.23 (4H, d, *J* 6.0, NC*H*$_2$Ph).

^{13}C NMR (100 MHz, DMSO-d_6) δ 158.1 (C=O), 141.0 (Ar-C_q), 128.3 (Ar-C), 127.0 (Ar-C), 126.6 (Ar-C), 43.0 (N*C*H_2Ph).

IR ν_{max} (neat)/cm^{-1} 3319 (NH), 1570 (C=O), 1485, 1236, 1122, 1070, 1030.

M.p. 167-168 °C (colourless needles).

HRMS (ESI) $C_{15}H_{17}N_2O$ $[M+H]^+$; calculated 241.1335, found 241.1337.

The spectral data are consistent with the literature values.[13]

* Rotatory evaporator used inside a fumehood.

1,3-Bis[(2,4-dimethoxyphenyl)methyl]urea 14c

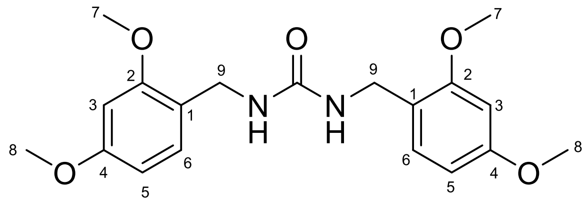

General procedure **C** was followed using urea (120 mg, 2.00 mmol, 1.0 eq.) 2,4-dimethoxybenzaldehyde (665 mg, 4.00 mmol, 2.00 eq.) and TMSCl (0.25 mL, 2.0 mmol, 1.0 eq.) in AcOH (2.0 mL) followed by the addition of $NaBH_4$ (151 mg, 4.00 mmol, 2.00 eq.). The precipitate after AcOH evaporation was suspended in H_2O (5.0 mL) and basified with aq. NaOH (5 N) until the pH reached 9-10 (litmus paper). The basified aq. crude reside was extracted with CH_2Cl_2 (3 × 15 mL). The combined organic phase was washed with brine (10 mL), dried over $MgSO_4$, and concentrated. Flash chromatography eluting from 100:0 DCM–MeOH to 98:2 DCM–MeOH gave the title compound **14c** (432 mg, 1.2 mmol, 60%) as a colourless amorphous solid.

$\mathbf{R}_f$ 0.12 (98:2 DCM–MeOH).

^{1}H NMR (600 MHz, DMSO-d_6): δ 7.05 (2H, d, *J* 8.3, 6-H), 6.52 (2H, d, *J* 2.4, 3-H), 6.46 (2H, dd, *J* 8.3, 2.4, 5-H), 6.12 (2H, t, *J* 5.9, N*H*), 4.07 (4H, d, *J* 5.9, 9-H), 3.77 (6H, s, 7-CH_3), 3.73 (6H, s, 8-CH_3).

^{13}C NMR (150 MHz, DMSO-d_6) δ 159.6 (4-C), 158.0 (C=O), 157.7 (2-C), 128.9 (6-C), 120.4 (1-C), 104.2 (5-C), 98.2 (3-C), 55.4 (7-CH_3), 55.2 (8-CH_3), 37.7 (9-C).

IR ν_{max} (neat)/cm^{-1} 3309 (NH), 1620 (C=O), 1587, 1503, 1254, 1210, 1031.

HRMS (ESI) $C_{19}H_{25}N_2O_5$ [M+H]$^+$; calculated 361.1758, found 361.1767.

The spectral data are consistent with the literature values.[14]

1-[(2,4-Dimethoxyphenyl)methyl]urea 14d.

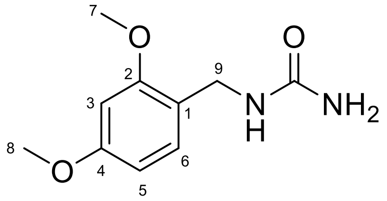

General procedure **C** was followed using urea (1.2 g, 20 mmol, 20.0 eq.) 2,4-dimethoxybenzaldehyde (167 mg, 1.00 mmol, 1.00 eq.) and TMSCl (0.25 mL, 2.0 mmol, 1.0 eq.) in AcOH (5.0 mL) followed by the addition of $NaBH_4$ (151 mg, 4.00 mmol, 2.00 eq.). The precipitate after AcOH evaporation was suspended in H_2O (10.0 mL) and basified with aq. NaOH (5 N) until the pH reached 9-10 (litmus paper). The basified aq. crude reside was extracted with CH_2Cl_2 (3 × 15 mL). The combined organic phase was washed with brine (10 mL), dried over $MgSO_4$, and concentrated. Flash chromatography eluting from 100:0 DCM–MeOH to 98:5 DCM–MeOH gave the title compound **14d** (112 mg, 0.53 mmol, 53%) as a colourless amorphous solid.

R*f* 0.15 (98:5 DCM–MeOH).

^{1}H NMR (400 MHz, DMSO-d$_6$): δ 7.07 (1H, d, *J* 8.3, 6-H), 6.53 (1H, d, *J* 2.5, 3-H), 6.46 (1H, dd, *J* 8.3, 2.5, 5-H), 6.10 (1H, t, *J* 6.0, N*H*), 5.46 (2 H, br. s, N*H$_2$*) 4.05 (2H, d, *J* 6.0, 9-H), 3.78 (3H, s, 7-CH_3), 3.73 (3H, s, 8-CH_3).

^{13}C NMR (150 MHz, DMSO-d$_6$) δ 159.6 (4-C), 158.7 (C=O), 157.7 (2-C), 128.7 (6-C), 120.4 (1-C), 104.2 (5-C), 98.2 (3-C), 55.4 (7-C), 55.2 (8-C), 37.7 (9-C).

IR ν_{max} (neat)/cm^{-1} 3409 (NH), 1609 (C=O), 1543, 1504, 1207, 1128, 1029.

HRMS (ESI) $C_{10}H_{14}N_2O_3Na$ $[M+Na]^+$; calculated 233.0902, found 233.0899.

The spectral data are consistent with the literature values.[15]

17. Synthesis of aryl / alkyl glyoxals

General procedure D: To synthesis aryl / alkylglyoxals, procedure **Part I** and/or **Part II** was followed:

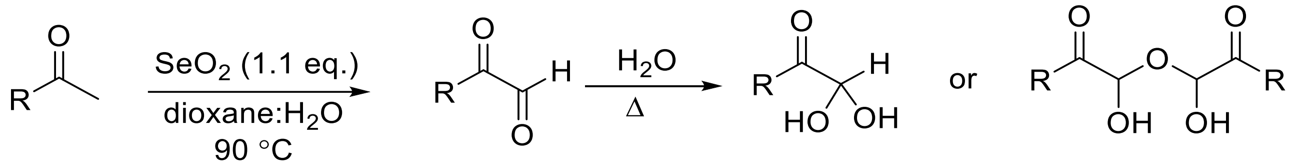

Part I: Glyoxal synthesis.

SeO_2 (665 mg, 6.0 mmol, 1.1 eq.) was stirred in 1,4-dioxane:H_2O (7:1, 4.0 mL, 1.5 M) at 55 °C until it dissolved. Aryl or alkyl ketone (5.0 mmol, 1.0 eq.) was added, and the reaction mixture was heated to 90 °C. The reaction progress was monitored by TLC until complete consumption of aryl or alkyl ketone was observed (typically 18 h). The reaction mixture was cooled to rt, then the selenium residues were removed by filtration through a celite pad, washed through with CH_2Cl_2 (20 mL). The reaction mixture was concentrated *in vacuo* to provide the crude glyoxal, typically as a yellow oil.

Part II: Glyoxal hydration

In all cases, hydration of the crude glyoxal was attempted by adding boiling H_2O (10 mL). In instances where a precipitate formed, the resulting solid was washed with cold H_2O (10 mL). The solid was re-crystallised from $CHCl_3$ (3.0 mL) to give the hydrated glyoxal product, as one or a mixture of the following forms: dioxo (anhydrous glyoxal), aldehyde hydrate (glyoxal•H_2O), aldehyde and ketone hydrate (glyoxal•$2H_2O$), hemihydrate (glyoxal•½H_2O), oligomers, and polymers – as can be anticipated from previous studies in the literature.[16] Wherever possible we have clearly stated the form(s) of the glyoxal products prepared. In instances where attempted hydration did not result in a precipitate, the glyoxal was purified as stated.

4-Methylphenylglyoxal hydrate 13b

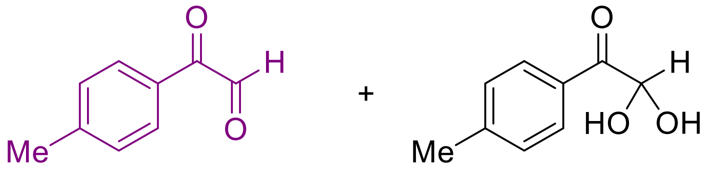

General procedure **D** parts **I** and **II** starting with 4-methylacetophenone (0.7 mL, 5.0 mmol, 1.0 eq) gave the title compound **13b** (231 mg, 1.4 mmol,* 28%) as a colourless foam.

^{1}H NMR (400 MHz, CD_3CN, mixture of dioxo [33%] and monohydrate [67%] forms): 9.59 (1H, s, C(O)H), 8.02 (2H, d, *J* 8.2, dioxo Ar-H), 7.98 (2H, d, *J* 8.7, hydrate Ar-H), 7.38 (2H, d, *J* 8.2, dioxo Ar-H), 7.35 (2H, d, *J* 8.7, hydrate Ar-H), 5.85 (1H, t, *J* 7.3, $C(OH)_2H$), 4.63 (2H, d, *J* 8.6, $C(OH)_2H$), 2.43 (3H, s, dioxo $ArCH_3$), 2.41 (3H, s, hydrate $ArCH_3$).

HRMS (ESI) $C_9H_9O_2$ $[M(dioxo)+H]^+$; calculated 149.0597 found: 149.0598; $C_{18}H_{18}NaO_5$ [M'M"(dimer[dioxo+glyoxal•H_2O])+Na]$^+$; calculated 337.1046, found 337.1045.

IR ν_{max}(neat)/cm^{-1} 3449 (OH), 2915, 1686 (C=O), 1604, 1222, 1092, 1025.

The reported ^{1}H NMR data agree with the literature values recorded in DMSO d_6[17]/MeOH-d_4 [18]

* Yield calculated based on the molecular weight of the glyoxal•H_2O (monohydrate) form.

4-Hydroxyphenylglyoxal hydrate 13c

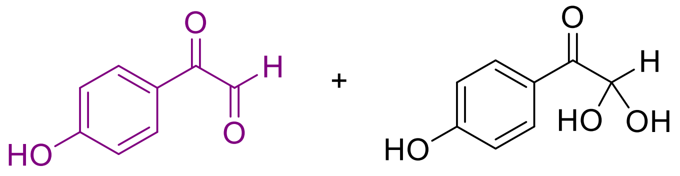

General procedure **D** parts **I** and **II** starting with 4-hydroxyacetophenone (680 mg, 5.0 mmol, 1.0 eq.) gave the title compound **13c** (178 mg, 1.1 mmol,* 21%) as a colourless foam.

^{1}H NMR (400 MHz, CD_3CN, mixture of dioxo [30%] and monohydrate [70%] forms): 9.57 (1H, s, C(O)H), 8.06 (2H, dd, *J* 8.9, 2.5, dioxo Ar-H), 8.00 (2H, dd, *J* 8.9, 2.7, hydrate Ar-H), 7.00-6.84 (m, includes: 2H, dioxo; and 2H, hydrate Ar-H), 5.83 (1H, s, $C(OH)_2$*H*), 4.63 (br. s, includes: 1H, dioxo ArO*H*; and 3H, C(O*H*)$_2$H and monohydrate ArO*H*).

HRMS (ESI) $C_{16}H_{14}NaO_7$ [M'M''(dimer[dioxo+glyoxal•H_2O])+Na]$^+$; calculated 341.0632, found 341.0630, $C_8H_7O_3$ [M(dioxo)+H]$^+$; calculated 151.0390 found: 151.0393.

IR ν_{max}(neat)/cm^{-1} 3388 (OH), 3288 (OH), 3114, 1669 (C=O), 1591, 1391, 1251.

* Yield calculated based on the molecular weight of the glyoxal•H_2O (monohydrate) form.

4-Chlorophenylglyoxal hydrate 13g

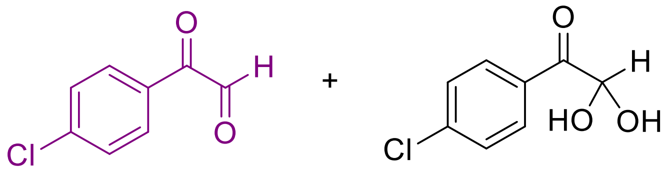

General procedure **D** parts **I** and **II** starting with 4-chloroacetophenone (0.65 mL, 5.0 mmol, 1.0 eq.) gave the title compound **13g** (158 mg, 0.83 mmol,* 17%) as a colourless foam.

^{1}H NMR (600 MHz, CD_3CN, mixture of dioxo [2%] and monohydrate [98%] forms): δ 9.54 (1H, s, C(O)*H*), 8.13-8.10 (2H, m, dioxo Ar-H), 8.09-8.04 (2H, m, hydrate Ar-H), 7.62-7.55 (2H, m, dioxo Ar-H), 7.57-7.53 (2H, m, hydrate Ar-H), 5.82 (1H, app. d, *J* 6.8, C(OH)*H*), 4.87 (2H, d, *J* 7.9, C(O*H*)$_2$H).

HRMS (ESI) $C_{16}H_{12}{}^{35}Cl_2NaO_5$ [M'M"(dimer[dioxo+glyoxal•H_2O])+Na]$^+$; calculated 376.9954, found 376.9958, $C_8H_6{}^{35}ClO_2$ [M(dioxo)+H]$^+$; calculated 169.0051 found: 169.0055.

IR ν_{max}(neat)/cm^{-1} 3373 (OH), 1694 (C=O), 1587, 1221, 1093, 1024, 810.

The reported ^{1}H NMR data agree with the literature values recorded in DMSO d_6.[17]

* Yield calculated based on the molecular weight of the glyoxal•H_2O (monohydrate) form.

4-Iodophenylglyoxal monohydrate 13i

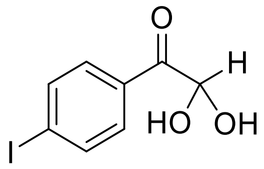

General procedure **D** parts **I** and **II** starting with 4-iodoacetophenone (1.2 g, 5.0 mmol, 1.0 eq.) gave title compound **13i** (589 mg, 2.11 mmol,* 42%) as a colourless foam.

^{1}H NMR (600 MHz, CD_3CN, monohydrate form): δ 7.94 (2H, dd, *J* 8.3, 3.5, Ar-H), 7.81 (2H, dd, *J* 8.3, 3.5, Ar-H), 5.81 (1H, dd, *J* 8.5, 2.6, C(OH)*H*), 4.85 (2H, app. dd, *J* 8.5, 2.6, C(O*H*)$_2$H).

HRMS (ESI) $C_{16}H_{12}I_2NaO_5$ [M'M"(dimer[dioxo+glyoxal•H_2O])+Na]$^+$; calculated 560.8666, found 560.8645, $C_8H_6IO_2$ [M(dioxo)+H]$^+$; calculated 260.9407 found: 260.9410

IR ν_{max}(neat)/cm^{-1} 3389 (OH), 1693 (C=O), 1578, 1219, 1122, 1024, 949.

* Yield calculated based on the molecular weight of the glyoxal•H_2O (monohydrate) form.

4-(Trifluoromethyl)phenylglyoxal hydrate 13j

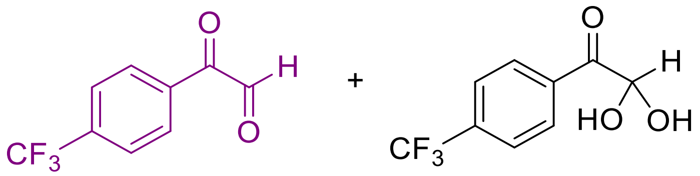

General procedure **D** parts **I** and **II** starting with 4-(trifluoromethyl)acetophenone (941 mg, 5.00 mmol, 1.00 eq.) gave the title compound **13j** (235 mg, 1.07 mmol,* 21%) as a colourless foam.

^{1}H NMR (400 MHz, CD_3CN, a mixture of dioxo [27%] and monohydrate [73%] forms): δ 9.55 (1H, s, C(O)*H*), 8.26 (2H, d, *J* 8.2, dioxo Ar-H), 8.23 (2H, d, *J* 8.1, hydrate Ar-H), 7.87 (2H, d, *J* 8.2, dioxo Ar-H), 7.84 (2H, d, *J* 8.1, hydrate Ar-H), 5.87 (1H, app. s, $C(OH)_2$*H*), 4.96 (2H, app. d, *J* 5.3, $C(O$*H*$)_2H$).

HRMS (ESI) $C_{18}H_{12}F_6NaO_5$ [M'M"(dimer[dioxo+glyoxal•H_2O])+Na]$^+$; calculated 445.0481, found 445.0477, $C_9H_6F_3O_2$ [M(dioxo)+H]$^+$; calculated 203.0314 found: 203.0308

IR ν_{max}(neat)/cm^{-1} 3427 (OH), 1695 (C=O), 1324, 1123, 1012, 954, 815.

The reported ^{1}H NMR data agree with the literature values recorded in DMSO d_6.[17]

* Yield calculated based on the molecular weight of the glyoxal•H_2O (monohydrate) form.

4-Nitrophenylglyoxal hydrate 13k

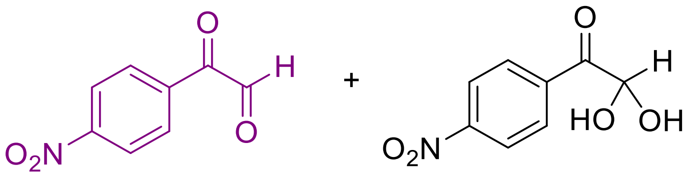

General procedure **D** parts **I** and **II** starting with 4-nitroacetophenone (941 mg, 5.00 mmol, 1.00 eq.) gave the title compound **13k** (385 mg, 1.07 mmol,* 20%) as a yellow amorphous solid.

^{1}H NMR (400 MHz, CD_3CN, a mixture of dioxo [10%] and monohydrate [90%] forms): δ 9.54 (1H, s, C(O)*H*), 8.37-8.29 (2.2H, d, m, dioxo Ar-H and hydrate Ar-H), 8.29-8.22 (2.2H, d, m, dioxo Ar-H and hydrate Ar-H), 5.86 (1H, app. s, $C(OH)_2$*H*), 5.01 (2H, app. br. s, C(O*H*)$_2$H).

HRMS (ESI) $C_{16}H_{12}N_2NaO_{10}$ [M'M''(dimer[dioxo+glyoxal•H_2O])+Na]$^+$; calculated 415.0384, found 415.0376, $C_8H_6NO_4$ [M(dioxo)+H]$^+$; calculated 180.0291 found: 180.0286

IR ν_{max}(neat)/cm^{-1} 3282 (OH), 1690 (C=O), 1606, 1598, 1531, 1346, 1212, 1100.

The reported ^{1}H NMR data agree with the literature values recorded in DMSO d_6.

* Yield calculated based on the molecular weight of the glyoxal•H_2O (monohydrate) form.

3-Methoxyphenylglyoxal hemihydrate 13l

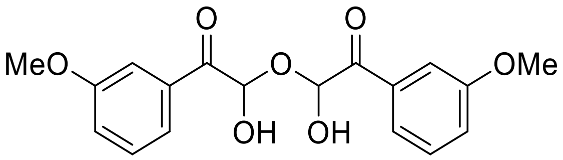

General procedure **D** parts **I** and **II** starting with 3-methoxyacetophenone (0.67 mL, 5.0 mmol, 1.0 eq.) gave the title compound **13l** (341 mg, 0.98 mmol,* 20%) as a yellow amorphous solid.

^{1}H NMR (600 MHz, CD_3CN, hemihydrate): δ 7.68 (2H, d, *J* 7.7, Ar-H), 7.58 (2H, s, Ar-H), 7.43 (2H, td, *J* 8.0, Ar-H), 7.23-7.19 (2H, m, Ar-H), 6.25 (2H, dd, *J* 10.9, 3.0, C(OH)*H*), 5.29 (2H, dd, *J* 10.9, 3.0, C(O*H*)$_2$H), 3.82 (6H, s, OC*H$_3$*).

HRMS (ESI) $C_{18}H_{18}NaO7$ [M'M"(dimer[dioxo+glyoxal•H_2O])+Na]$^+$ calculated 369.0945, found 369.0947, $C_9H_9O_3$ [M(dioxo)+H]$^+$; calculated 165.0546 found: 165.0548

IR ν_{max}(neat)/cm^{-1} 3362 (OH), 1679 (C=O), 1579, 1414, 1257, 1103, 975.

The reported ^{1}H NMR data agree with the literature values recorded in DMSO d_6[17]/MeOH-d_4 [18]

---

* Yield calculated based on the molecular weight of the glyoxal•½H_2O (hemihydrate) form.

3-Chlorophenylglyoxal hemihydrate 13m

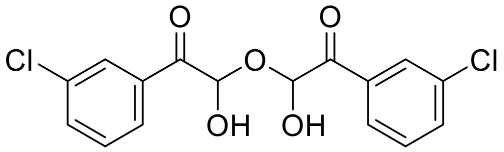

General procedure **D** parts **I** and **II** starting with 3-chloroacetophenone (0.65 mL, 5.00 mmol, 1.0 eq,) gave compound **13m** (251 mg, 0.71 mmol,* 14%) as a yellow solid.

^{1}H NMR (600 MHz, CD_3CN, hemihydrate form): δ 8.04 (2H, m, Ar-H), 8.01 (2H, d, *J* 7.8, Ar-H), 7.65 (2H, dd, *J* 8.0, 2.2, Ar-H), 7.51 (2H, td, *J* 8.0, 7.8, 2.2, Ar-H), 6.19 (2H, d, *J* 10.8, C(OH)*H*), 5.39 (2H, d, *J* 10.8, C(O*H*)$_2$H).

HRMS (ESI) $C_{16}H_{12}{}^{35}Cl_2NaO_5$ [M'M''(dimer[dioxo+glyoxal•H_2O])+Na]$^+$; calculated 376.9954, found 376.9953, $C_8H_6{}^{35}ClO_2$ [M(dioxo)+H]$^+$; calculated 169.0051 found: 169.0053.

IR ν_{max}(neat)/cm^{-1} 3374 (OH), 1694 (C=O), 1569, 1407, 1293, 1216, 1002.

* Yield calculated based on the molecular weight of the glyoxal•½H_2O (hemihydrate) form.

2-Methoxyphenylglyoxal 13o

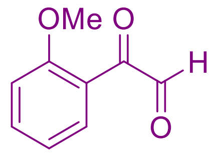

General procedure **D** parts **I** was followed starting with 2′-methoxyacetophenone (0.80 mL, 5.0 mmol, 1.0 eq.). [***Attempted***: hydration with hot H_2O did not result in a precipitate]. The residue was cooled to rt, and partitioned with CH_2Cl_2 (15 mL). The organic layer was washed with sat. aq. $NaHCO_3$ (10 mL) and brine (10 mL), dried over $MgSO_4$, filtered, and concentrated. Flash chromatography eluting with 80:20 hexane–EtOAc gave the title compound **13o** (756 mg, 4.15 mmol,* 83%) as a viscous yellow oil, comprising several different forms,† including the dioxo (anhydrous) and glyoxal monohydrate forms. The product was carried forward to the next reaction without further purification. Characteristic data are as follows:

R*f* 0.42 (80:20 hexane–EtOAc).

^{1}H NMR (600 MHz, $CDCl_3$, mixture of glyoxal forms, characteristic peaks only): δ 9.72 (1H, s, CHO).

HRMS (ESI) $C_{18}H_{18}NaO_7$ [M'M"(dimer[dioxo+glyoxal•H_2O])+Na]$^+$; calculated 369.0945, found 369.0946, $C_9H_9O_3$ [M(dioxo)+H]$^+$; calculated 165.0546 found: 165.0549.

IR ν_{max}(neat)/cm^{-1} 3454 (OH), 1679 (C=O), 1596, 1487, 1463, 1246, 1014.

* Yield calculated based on the molecular weight of the glyoxal form.

† See General Procedure B for more details.

2-Chlorophenylglyoxal 13p

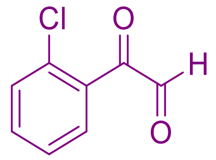

General procedure **D** part **I** was followed starting with 2-chloroacetophenone (0.64 mL, 5.0 mmol, 1.0 eq.). [***Attempted***: hydration with hot H_2O did not result in a precipitate]. The residue was cooled to rt, and partitioned with CH_2Cl_2 (15 mL). The organic layer was washed with sat. aq. $NaHCO_3$ (10 mL) and brine (10 mL), dried over $MgSO_4$, filtered, and concentrated. Flash chromatography eluting with 80:20 hexane–EtOAc gave the title compound **13p** (742 mg, 3.97 mmol,* 79%) as a viscous yellow oil, comprising several different forms.† The product was carried forward to the next reaction without further purification. Characteristic data are as follows:

R*f* 0.46 (80:20 hexane–EtOAc).

^{1}H NMR (400 MHz, $CDCl_3$, mixture of glyoxal forms, characteristic peaks only): δ 9.69 (1H, s, C(O)*H*).

IR ν_{max}(neat)/cm^{-1} 3397 (OH), 1703 (C=O), 1589, 1434, 1114, 1062, 967.

HRMS (ESI): $C_{16}H_{12}{}^{35}Cl_2NaO_5$ [M'M"(dimer[dioxo+glyoxal•H_2O])+Na]$^+$; calculated 376.9954, found 376.9953, $C_8H_6{}^{35}ClO_2$ [M(dioxo)+H]$^+$; calculated 169.0051 found: 169.0051

* Yield calculated based on the molecular weight of the glyoxal form.
† See General Procedure D for more details.

2-(Trifluoromethyl)phenylglyoxal hydrate 13q

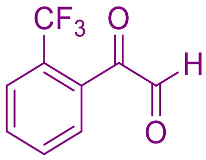

General procedure **B** parts **I** was followed starting with 2′-(trifluoromethyl)acetophenone (0.76 mL, 5.0 mmol, 1.0 eq.). [***Attempted***: hydration with hot H_2O did not result in a precipitate]. The residue was cooled at rt was diluted in CH_2Cl_2 (15 mL). The organic layer was washed with sat. aq. $NaHCO_3$ (10 mL) and brine (10 mL), dried over $MgSO_4$, filtered, and concentrated. Flash chromatography eluting with 80:20 hexane–EtOAc gave the title compound **13q** (680 mg, 3.37 mmol,* 67%) as a viscous yellow oil, comprising several different forms.† The product was carried forward to the next reaction without further purification. Characteristic data are as follows:

R*f* 0.45 (80:20 hexane–EtOAc).

^{1}H NMR (600 MHz, $CDCl_3$, mixture of glyoxal forms, characteristic peaks only): δ 9.56 (1H, s, C(O)*H*).

IR ν_{max}(neat)/cm^{-1} 3412 (OH), 1718 (C=O), 1311, 1117, 1034, 972, 765.

HRMS (ESI): $C_{18}H_{12}F_6NaO_5$ [M'M''(dimer[dioxo+glyoxal•H_2O])+Na]$^+$; calculated 445.0481, found 445.0477, $C_9H_6F_3O_2$ [M(dioxo)+H]$^+$; calculated 203.0314 found: 203.0308

* Yield calculated based on the molecular weight of the glyoxal form.
† See General Procedure B for more details.

1-Naphthylglyoxal hemihydrate 13r

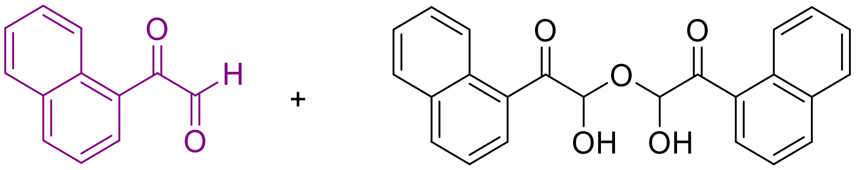

General procedure **D** parts **I** and **II** starting with 1-acetonaphthone (0.76 mL, 5.0 mmol, 1.0 eq.) gave the title compound **13r** (245 mg, 0.63 mmol,* 13%) as a colourless foam.

^{1}H NMR (400 MHz, CD_3CN, a mixture of dioxo [10%] and hemihydrate [90%] forms): δ 9.69 (1H, s, C(O)*H*), 8.76 (1H, d, *J* 8.60, dioxo Ar-H), 8.70 (2H, dd, *J* 7.82, 2.21, hemihydrate Ar-H), 8.36-8.27 (2H, m, hemihydrate Ar-H), 8.25-8.17 (2H, m, dioxo Ar-H), 8.14 (2H, dd, *J* 8.53, 2.65, hemihydrate Ar-H), 8.03 (1H, d, *J* 8.58, dioxo Ar-H), 7.95 (2H, ddd, *J* 6.22, 3.67, 1.81, hemihydrate Ar-H), 7.77-7.63 (3H, m, dioxo Ar-H), 7.58 (6H, m, hemihydrate Ar-H), 6.33 (2H, d, *J* 10.7, C(OH)*H*), 5.31 (2H, d, *J* 10.7, C(O*H*)$_2$H).

HRMS (ESI): $C_{24}H_{18}NaO_5$ [M'M"(dimer[dioxo+glyoxal•H_2O])+Na]$^+$; calculated 409.1046, found 409.1043, $C_{12}H_9O_2$ [M(dioxo)+H]$^+$; calculated 185.0597 found: 185.0597

IR ν_{max}(neat)/cm^{-1} 3433 (OH), 1671 (C=O), 1505, 1368, 1065, 989, 931

The reported ^{1}H NMR data agree with the literature values recorded in $CDCl_3$[17].

* Yield calculated based on the molecular weight of the glyoxal•½H_2O (hemihydrate) form.

2-Naphthylglyoxal hydrate 13s

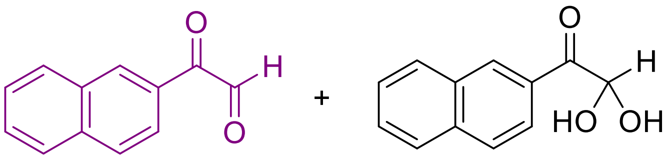

General procedure **D** parts **I** and **II** starting with 2-acetonaphthone (851 mg, 5.0 mmol, 1.0 eq.) gave the title compound **13s** (385 mg, 1.90 mmol,* 38%) as colourless foam. Characteristic data are as follows

^{1}H NMR (600 MHz, CD_3CN, mixture of dioxo [53%] and hydrate [47%] forms): δ 9.70 (1H, s, C(O)*H*), 8.83 (1H, s, dioxo Ar-H), 8.73 (1H, s, hydrate Ar-H), 8.16-7.91 (4H + 4H, m, Ar-H and Ar-H), 7.77-7.56 (2H,+ 2H m, Ar-H and Ar-H), 6.03 (1H, br. s, C(OH)*H*), 4.93 (2H, app. br. s, C(O*H*)H).

HRMS (ESI): $C_{24}H_{18}NaO_5$ [M'M"(dimer[dioxo+glyoxal•H_2O])+Na]$^+$; calculated 409.1046, found 409.1043, $C_{12}H_9O_2$ [M(dioxo)+H]$^+$; calculated 185.0597 found: 185.0597

IR ν_{max}(neat)/cm^{-1} 3362 (OH), 1690 (C=O), 1183, 1097, 991, 797, 745.

The reported ^{1}H NMR data agree with the literature values recorded in DMSO d_6[17]/MeOH-d_4 [18]

* Yield calculated based on the molecular weight of the glyoxal•H_2O (monohydrate) form.

3,3-Dimethyl-2-oxobutanal S13y

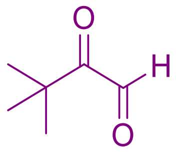

General procedure **D** part **I** was followed starting with 3,3-dimethylbutyraldehyde (0.63 mL, 5.0 mmol, 1.0 eq.). [***Attempted***: hydration with hot H_2O did not result in a precipitate]. The residue was purified by short path vacuum distillation under reduced pressure at 100 °C to give the title compound **S13y** (250 mg, 1.89 mmol,* 38%) as a yellow oil, comprising different glyoxal forms.† The product was carried forward to the next reaction without further purification. Characteristic data are as follows:

^{1}H NMR (600 MHz, $CDCl_3$, mixture of glyoxal forms, characteristic peak only): δ 9.30 (1H, s, C(O)*H*).

IR ν_{max}(neat)/cm^{-1} 3375 (OH), 2965, 1717 (C=O), 1255, 1118, 994, 870.

HRMS (ESI): $C_{12}H_{22}NaO_5$ [M'M"(dimer[dioxo+glyoxal•H_2O])+Na]$^+$; calculated 269.13594, found 269.13596, $C_6H_{11}O_2$ [M(dioxo)+H]$^+$; calculated 115.07536 found: 115.07536.

* Yield calculated based on the molecular weight of the glyoxal•H_2O (monohydrate) form.
† See General Procedure B for more details.

2-(Adamantan-1-yl)-2-oxoacetaldehyde S13z

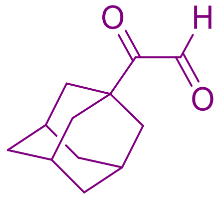

General procedure **D** part **I** was followed starting with 1-acetyladamantane (891 mg, 5.0 mmol, 1.0 eq.). [***Attempted***: hydration with hot H_2O did not result in a precipitate]. The residue was diluted in CH_2Cl_2 (15 mL), washed with sat. aq. $NaHCO_3$ (10 mL) and brine (10 mL), then dried over $MgSO_4$, filtered, and concentrated. Flash chromatography eluting with 80:20 hexane–EtOAc gave the title compound **S13z** (768 mg, 3.98 mmol,* 80%) as an amorphous colourless solid, comprising different glyoxal forms.† The product was carried forward to the next reaction without further purification. Characteristic data are as follows:

^{1}H NMR (600 MHz, $CDCl_3$, mixture of glyoxal forms, characteristic peak only): δ 9.31 (1H, s C(O)*H*).

IR ν_{max}(neat)/cm^{-1} 3510-3294 (OH), 2902, 2849, 1711 (C=O), 1450, 1343, 1073.

HRMS (ESI): $C_{24}H_{35}NaO_5$ [M'M''(dimer[dioxo+glyoxal•H_2O])+Na]$^+$; calculated 425.2298, found 425.2319, $C_{12}H_{17}O_2$ [M(dioxo)+H]$^+$; calculated 193.1223 found: 193.1223

---

* Yield calculated based on the molecular weight of the glyoxal•H_2O (monohydrate) form.

† See General Procedure B for more details.

2-Cyclohexyl-2-oxoacetaldehyde hemihydrate S13aa

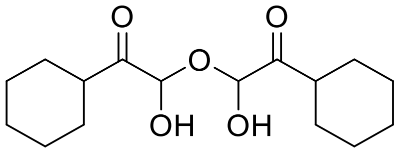

General procedure **B** part **I** and part **II** were followed starting with 1-cyclohexylethan-1-one (0.69 mL, 5.0 mmol, 1.0 eq.) gave the title compound **S13aa** (155 mg, 0.52 mmol,* 10%) as a colourless solid.

^{1}H NMR (600 MHz, $CDCl_3$, hemihydrate form): δ 4.89 (2H, d, *J* 10.7, C(O*H*)$_2$H), 2.14 (2H, d, *J* 11.8, C(OH)*H*), 2.05-1.97 (2H, m, CyH), 1.87-1.75 (6H, m, CyH), 1.70 (2H, d, *J* 12.3, CyH), 1.42-1.14 (12H, m, CyH).

IR ν_{max}(neat)/cm^{-1} 3299 (OH), 2922, 2853, 1711 (C=O), 1435, 1080, 1042.

HRMS (ESI): $C_{16}H_{26}NaO_5$ [M'M"(dimer[dioxo+glyoxal•H_2O])+Na]$^+$; calculated 321.1672, found 321.1686, $C_8H_{13}O_2$ [M(dioxo)+H]$^+$; calculated 141.0910 found: 141.0912**.**

The reported ^{1}H NMR data agree with the literature values recorded in DMSO d$_6$.[17]

* Yield calculated based on the molecular weight of the glyoxal•½H_2O (hemihydrate) form.

2-Cyclopropyl-2-oxoacetaldehyde S13ab

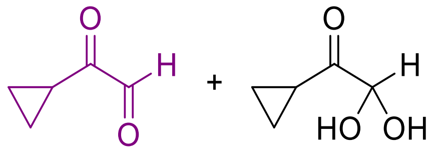

General procedure **B** part I was followed starting with cyclopropyl methyl ketone (0.49 mL, 5.0 mmol, 1.0 eq.). [***Attempted***: hydration with hot H_2O did not result in a precipitate]. The residue was purified by short path vacuum distillation under reduced pressure at 100 °C to give the title compound **S13ab** (180 mg, 1.83 mmol,* 37%) as a yellow oil, mainly comprising the dioxo and monohydrate forms of the glyoxal, but other forms are also present in smaller amounts.† The product was carried forward to the next reaction without further purification. Characteristic data are as follows:

^{1}H NMR (400 MHz, $CDCl_3$, 2H for C(O*H*)$_2$ in the hydrate form not observed): δ 9.22 (1H, s, C(O)*H*), 5.56 (1H, d, *J* 10.7, C(OH)*H*), 2.66-2.59 (1H, m, hydrate cyclopropyl (CH), 2.31-2.09 (1H, m, dioxo cyclopropyl (CH), 1.40-0.95 (4H + 4H, m, dioxo and hydrate (CH_2)$_2$).

IR ν_{max}(neat)/cm^{-1} 3401 (OH), 3012, 1702 (C=O), 1382, 1324, 1057, 1018.

HRMS (ESI): $C_{10}H_{14}NaO_5$ [M'M"(dimer[dioxo+glyoxal•H_2O])+Na]$^+$; calculated 237.0733, found 237.0731, $C_5H_7O_2$ [M(dioxo)+H]$^+$; calculated 99.0441 found: 99.0439

* Yield calculated based on the molecular weight of the glyoxal•H_2O (monohydrate) form.
† See General Procedure B for more details.

***N*-methoxy-*N*-methyl-2-phenylacetamide 25**

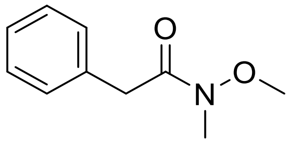

Following modification of a procedure by Iwasa,[19] to a stirred solution of phenyl acetylchloride (0.33 mL, 2.50 mmol, 1.0 eq.) and *N*,*O*-dimethyl hydroxylamine (293 mg, 3.00 mmol, 1.20 eq.) in DCM (10 mL) at 0 °C was added dropwise pyridine (0.89 mL, 11.0 mmol, 2.2 eq.). The reaction mixture was stirred for 2 h, warming from 0 °C to rt. After disappearance of the starting material spot by TLC, the reaction mixture was washed with aq. HCl (0.1 N, 2 x 10 mL), H_2O (2 x 10 mL), sat. aq. $NaHCO_3$ solution (2 x 10 mL), and brine (1 x 10 mL). The organic layer was dried over $MgSO_4$ and concentrated to provide the title compound **25** (493 mg, 2.45 mmol, 98%) as colourless oil, which was carried forward to the next step without purification.

R*f* 0.40 (80:20 hexane–EtOAc).

^{1}H NMR (600 MHz, $CDCl_3$): δ 7.33-7.28 (4H, m, Ar-H), 7.26-7.22 (1H, m, Ar-H), 3.78 (2H, s, PhCH_2), 3.60 (3H, s NCH_3), 3.19 (3H, s, OCH_3).

^{13}C NMR (150 MHz, $CDCl_3$): δ 172.5 (C=O), 135.0 (Ar-C_q), 129.4 (Ar-C), 128.6 (Ar-C), 126.9 (Ar-C), 61.4 (O*C*H_3), 39.5 (Ph*C*H_2), 32.3 (N*C*H_3).

HRMS (ESI) HRMS (ESI): $C_{10}H_{15}NO_2$ $[M+H]^+$; calculated 180.1019, found 180.1021

The spectral data are consistent with the literature values.[19]

Benzyl vinyl ketone 26

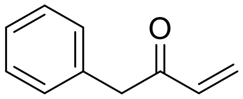

To a stirred solution of *N*-methoxy-*N*-methyl-2-phenylacetamide **25** (179.2 mg, 1.0 mmol, 1.0 eq.) in dry THF (7.0 mL) at 0 °C was added dropwise a solution of vinyl magnesium bromide (1.2 mL, 1.2 mmol, 1.0 M in THF, 1.2 eq.). The reaction mixture was stirred for 10 min at 0 °C. After the disappearance of the starting material spot by TLC, the reaction mixture was quenched with pre-cooled sat. aq. NH_4Cl solution (10 mL) at 0 °C, then concentrated. The residue was partitioned between H_2O (10 mL) and separated with CH_2Cl_2 (3 × 15 mL). The combined organic phase was washed with brine (10 mL), dried over $MgSO_4$, and concentrated. Flash chromatography eluting with 99:1 DCM–MeOH gave the title compound **26** (130 mg, 0.89 mmol, 89%) as a colourless oil.

$\mathbf{R}_f$ 0.63 (80:20 hexane–EtOAc).

^{1}**H NMR** (600 MHz, $CDCl_3$): δ 7.38-7.31 (2H, m, Ar-H), 7.29-7.26 (1H, m, Ar-H), 7.23-7.19 (2H, m, Ar-H), 6.41 (1H, dd, *J* 17.6, 10.4, C*H*=CH$_2$), 6.31 (1H, dd, *J* 17.6, 1.2, CH=CH_AH$_B$), 5.83 (1H, dd, *J* 10.4, 1.2, CH=CH$_A$$H_B$), 3.88 (2H, s, PhC$H_2$).

^{13}**C NMR** (150 MHz, $CDCl_3$): δ 197.9 (C=O), 135.7 (CO*C*H=CH$_2$), 134.2 (Ar-C_q), 129.6 (Ar-C), 129.2 (CH=*C*H$_2$), 128.9 (Ar-C), 127.2 (Ar-C), 47.3 (CO*C*H$_2$Ph).

IR ν_{max} (neat)/cm^{-1} 2930, 1679 (C=O) 1615, 1526, 1483, 1233, 1082, 1030.

HRMS (ESI): $C_{10}H_{11}O$ $[M+H]^+$; calculated: 147.0804, found 147.0806.

The spectral data are consistent with the literature values.[19]

2-Oxo-3-phenylpropanal S13ac

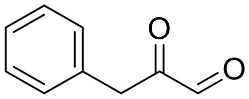

***Caution!:** Ozonides and peroxides are potentially explosive. The presence of ozonides / peroxides can be detected using potassium iodide-starch paper (e.g Supelco®109512). Test strips are dipped in the relevant reaction mixture, then dipped in H_2O, then dried with a heatgun. A dark blue colour indicates ozonides / peroxides are present, while no colour change indicates ozonides / peroxides are absent.*

A solution of benzyl vinylketone **26** (130 mg, 0.89 mmol) in MeOH:CH_2Cl_2 (0.5:2.0 mL) at −78 °C was purged with O_2. After ~2 min, the ozone generator was initiated, and O_3 was bubbled through the solution for ~5 min. The reaction mixture turned yellowish and TLC analysis confirmed the disappearance of the starting material spot. The reaction mixture was purged with O_2 to remove the dissolved O_3. After >5 min, the O_2 stream was switched off, and DMS (0.26 mL, 3.56, 4.00 eq.) was added. The reaction mixture was warmed to rt. After 6 h, it was established that no peroxides were present (potassium iodide-starch paper, *see note above*) and the reaction mixture was concentrated. Flash chromatography eluting with 99:1 DCM–MeOH gave the title product S**13ac** (80 mg, 0.53 mmol,[*] 61%) as a yellow oil, mainly comprising the dioxo form, but other glyoxal forms were present in smaller amounts. The product was carried forward to the next reaction without further purification. Characteristic data are as follows:

R_f 0.72 (90:10 DCM–MeOH).

^{1}H NMR (600 MHz, $CDCl_3$): δ 9.27 (1H, s, C(O)*H*), 3.64 (2H, s, PhCH_2CO).

HRMS (ESI): $C_9H_9O_2$ [M(dioxo)+H]$^+$; calculated 149.0597 found: 149.0598.

IR ν_{max} (neat)/cm^{-1} 3395 (OH), 2830, 1809, 1620 (C=O), 1556, 1435, 1244, 1082, 972.

[*] Yield calculated based on the molecular weight of the dioxo (anhydrous) glyoxal form.

18. Crystal data

18.1 Crystal data for 17a

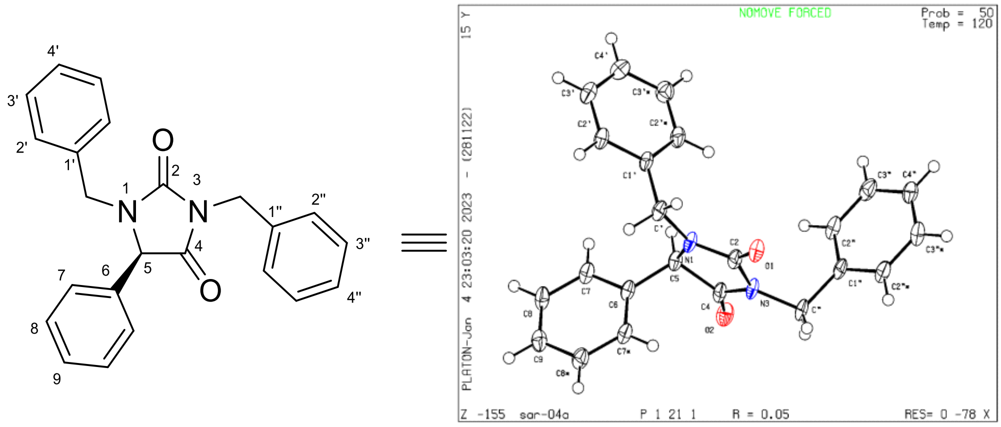

**Identification code**	**SAR-04A**
Empirical formula	$C_{23}H_{20}N_2O_2$
Formula weight	356.41
Temperature/K	120.0(2)
Crystal system	monoclinic
Space group	$P2_1$
a/Å	12.0926(2)
b/Å	5.4361(2)
c/Å	13.6728(3)
α/°	90
β/°	95.641(2)
γ/°	90
Volume/Å^3	894.45(4)
Z	2
ρ_{calc}g/cm^3	1.323
μ/mm^{-1}	0.679
F(000)	376.0
Crystal size/mm^3	0.291 × 0.195 × 0.085
Radiation	Cu Kα (λ = 1.54184)
2Θ range for data collection/°	7.346 to 148.93
Index ranges	-15 ≤ h ≤ 14, -6 ≤ k ≤ 6, -17 ≤ l ≤ 17
Reflections collected	15907
Independent reflections	3500 [R_{int} = 0.0890, R_{sigma} = 0.0566]
Data/restraints/parameters	3500/1/244
Goodness-of-fit on F^2	1.063
Final R indexes [I>=2σ (I)]	R_1 = 0.0550, wR_2 = 0.1485
Final R indexes [all data]	R_1 = 0.0574, wR_2 = 0.1526
Largest diff. peak/hole / e Å^{-3}	0.21/-0.39
Hooft parameter	0.15(13)
Flack parameter	0.1(3)
CCDC	2235706

18.2 Crystal data for 17f

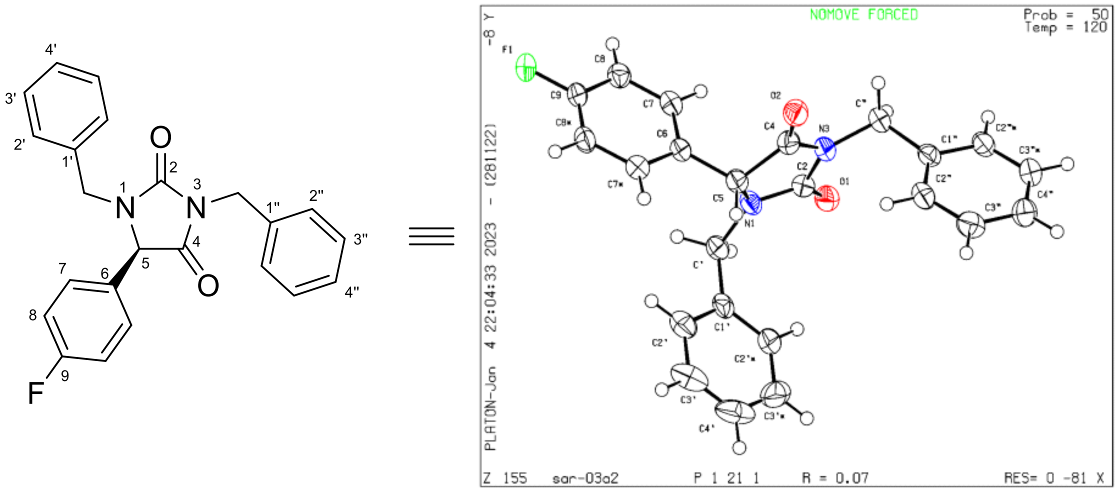

**Identification code**	**SAR-03a2**
Empirical formula	$C_{23}H_{19}N_2O_2F$
Formula weight	374.40
Temperature/K	119.9(3)
Crystal system	monoclinic
Space group	$P2_1$
a/Å	12.3101(2)
b/Å	5.45170(10)
c/Å	14.3867(2)
α/°	90
β/°	106.621(2)
γ/°	90
Volume/Å^3	925.16(3)
Z	2
ρ_{calc}g/cm^3	1.344
μ/mm^{-1}	0.764
F(000)	392.0
Crystal size/mm^3	0.663 × 0.137 × 0.089
Radiation	Cu Kα (λ = 1.54184)
2Θ range for data collection/°	7.494 to 134.126
Index ranges	$-14 \le h \le 14$, $-6 \le k \le 6$, $-15 \le l \le 17$
Reflections collected	11231
Independent reflections	3114 [R_{int} = 0.0762, R_{sigma} = 0.0570]
Data/restraints/parameters	3114/1/253
Goodness-of-fit on F^2	1.034
Final R indexes [I>=2σ (I)]	R_1 = 0.0731, wR_2 = 0.1820
Final R indexes [all data]	R_1 = 0.0738, wR_2 = 0.1834
Largest diff. peak/hole / e Å^{-3}	0.49/-0.38
Hooft parameter	0.20(8)
Flack parameter	0.3(2)
CCDC	2235705

18.3 Crystal data for 17h

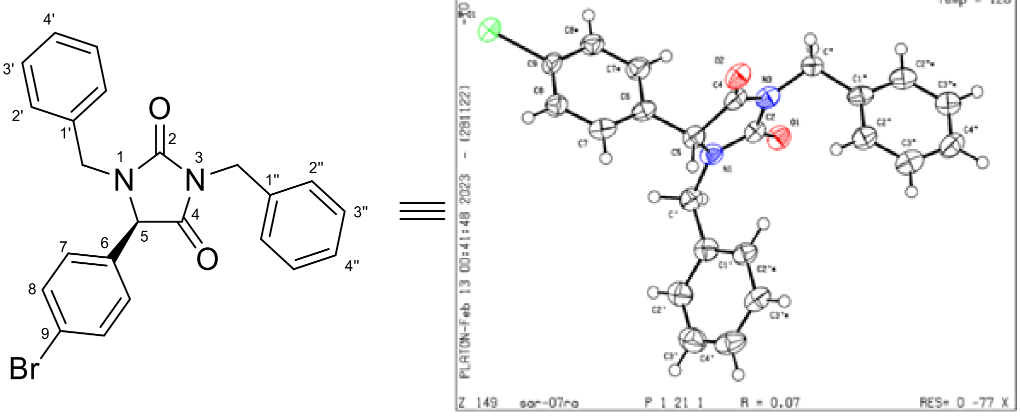

**Identification code**	**SAR-07RA**
Empirical formula	$C_{23}H_{19}N_2O_2Br$
Formula weight	435.31
Temperature/K	120.0(2)
Crystal system	monoclinic
Space group	$P2_1$
a/Å	12.5962(6)
b/Å	5.2216(2)
c/Å	15.4025(6)
α/°	90
β/°	109.764(5)
γ/°	90
Volume/Å^3	953.38(7)
Z	2
ρ_{calc}g/cm^3	1.516
μ/mm^{-1}	3.112
F(000)	444.0
Crystal size/mm^3	0.148 × 0.07 × 0.037
Radiation	Cu Kα (λ = 1.54184)
2Θ range for data collection/°	6.098 to 133.94
Index ranges	$-14 \le h \le 15$, $-4 \le k \le 6$, $-18 \le l \le 18$
Reflections collected	8019
Independent reflections	3014 [R_{int} = 0.0824, R_{sigma} = 0.0773]
Data/restraints/parameters	3014/1/253
Goodness-of-fit on F^2	0.959
Final R indexes [I>=2σ (I)]	R_1 = 0.0616, wR_2 = 0.1549
Final R indexes [all data]	R_1 = 0.0702, wR_2 = 0.1612
Largest diff. peak/hole / e Å^{-3}	0.78/-0.66
Flack parameter	0.01(5)
CCDC	2241398

19. References

[1] L. Caruana, M. Fochi, S. Ranieri, A. Mazzanti, L. Bernardi, *Chem. Commun.* **2013**, *49*, 880–882.

[2] O. V Dolomanov, L. J. Bourhis, R. J. Gildea, J. A. K. Howard, H. Puschmann, *J. Appl. Cryst* **2009**, *42*, 339–341.

[3] G. M. Sheldrick, *Acta Cryst A71* **2015**, 3–8.

[4] G. M. Sheldrick, *Acta Cryst. C71* **2015**, 3–8.

[5] A. F. Olea, F. Wilkinson, *J. Phys. Chem* **1995**, *99*, 4518–4524.

[6] H. H. Wasserman, S. Terao, *Tetrahedron Lett.* **1975**, *16*, 1735–1738.

[7] J. Burés, *Angew. Chemie Int. Ed.* **2016**, *55*, 16084–16087.

[8] T. Keenan, A. Jean, S. Arseniyadis, *ACS Org. Inorg. Au* **2022**, *2*, 312–317.

[9] Y. Nie, J. Li, Q. Yuan, W. Zhang, *Chinese J. Chem.* **2022**, *40*, 819–824.

[10] R. C. Atkinson, D. J. Leonard, J. Maury, D. Castagnolo, N. Volz, J. Clayden, *Chem. Commun.* **2013**, *49*, 9734–9736.

[11] L. Bou-Iserte, A. Latorre, S. Rodríguez, F. V. González, *ACS Omega* **2019**, *4*, 2261–2267.

[12] D. Xu, L. Ciszewski, T. Li, O. Repič, T. J. Blacklock, *Tetrahedron Lett.* **1998**, *39*, 1107–1110.

[13] H. Song, Z. Han, C. Zhang, *Chem. – A Eur. J.* **2019**, *25*, 10907–10912.

[14] A. E. Owen, A. Preiss, A. Mcluskie, C. Gao, G. Peters, M. Bühl, A. Kumar, *ACS Catal.* **2022**, *12*, 6923–6933.

[15] M. H. Holschbach, D. Bier, W. Wutz, S. Willbold, R. A. Olsson, *Org. Lett.* **2009**, *11*, 4266–4269.

[16] Olpp T, *Sci. Synth.* **2007**, *25*, 423–439.

[17] P. Wang, W. J. Tao, X. L. Sun, S. Liao, Y. Tang, *J. Am. Chem. Soc.* **2013**, *135*, 16849–16852.

[18] S. Zheng, W. Smit, A. Spannenberg, S. Tin, J. G. de Vries, *Chem. Commun.* **2022**, *58*, 4639–4642.

[19] S. Chanthamath, S. Takaki, K. Shibatomi, S. Iwasa, *Angew. Chemie Int. Ed.* **2013**, *52*, 5818–5821.

20. Processed NMR spectra

1,3-Dibenzyl-5-phenylimidazolidine-2,4-dione 17a, ^{1}H NMR in CDCl$_3$

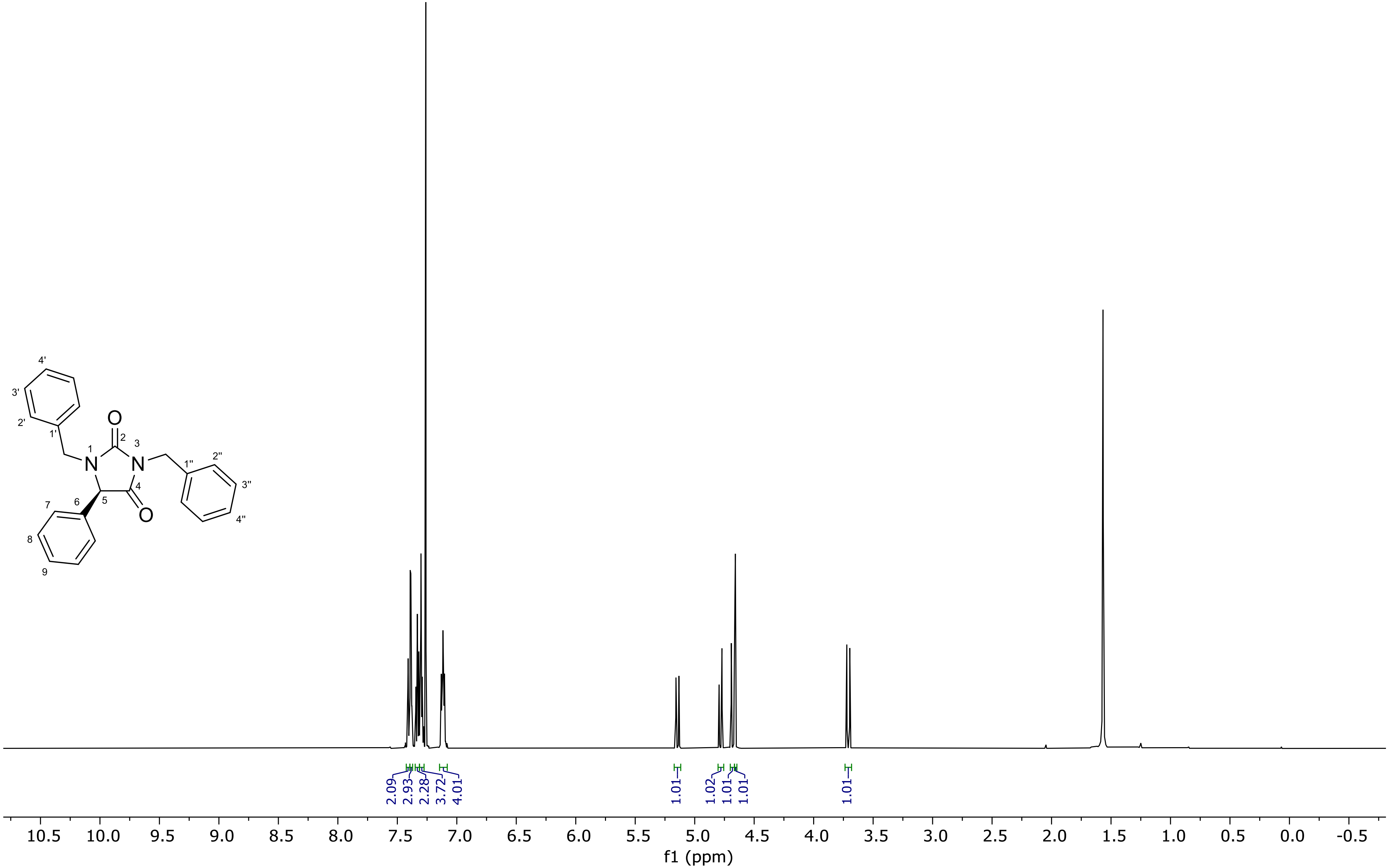

1,3-Dibenzyl-5-phenylimidazolidine-2,4-dione 17a, ^{13}C NMR in $CDCl_3$

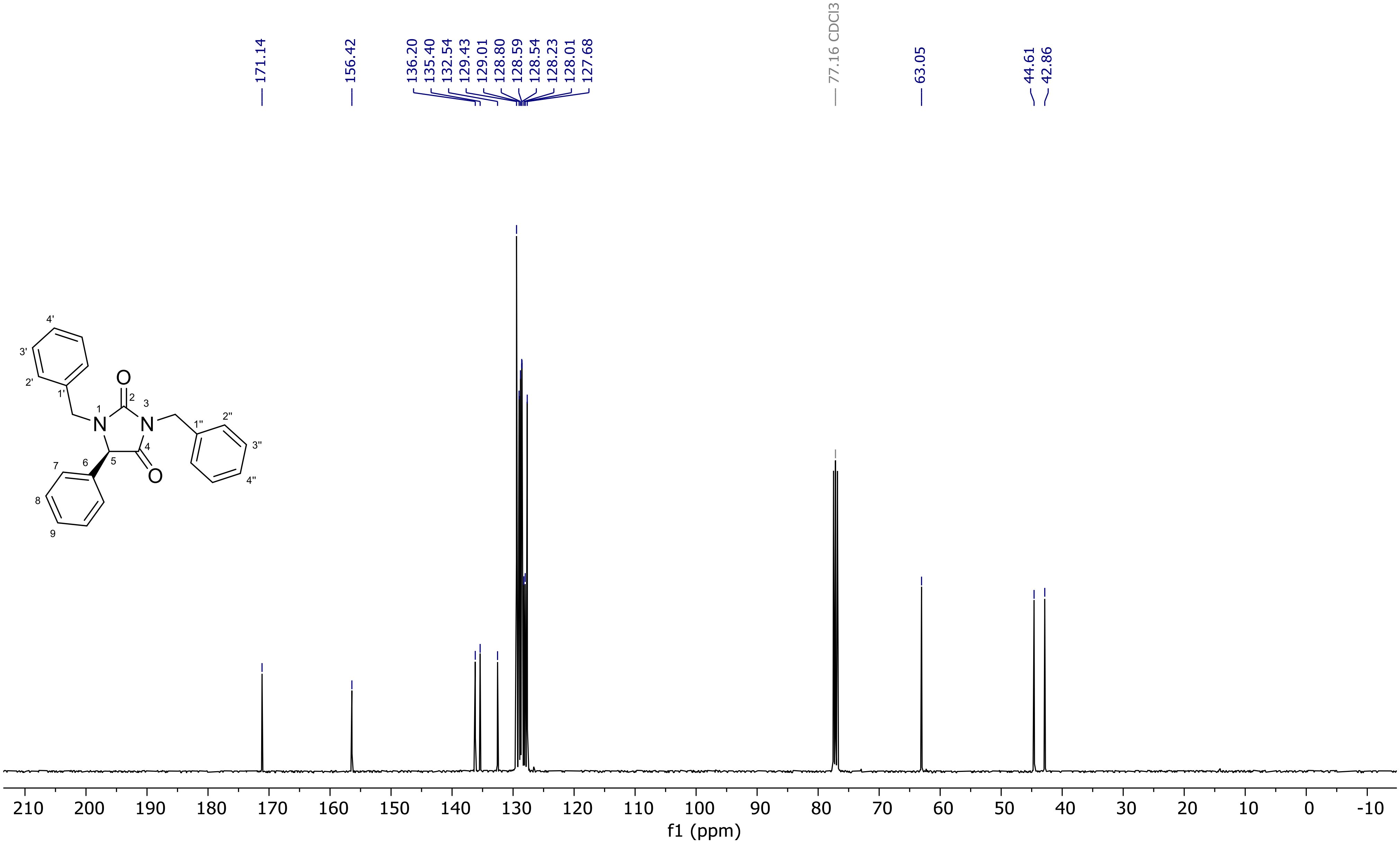

1,3-Dibenzyl-5-(4-methylphenyl)imidazolidine-2,4-dione 17b, ^{1}H NMR in CDCl₃

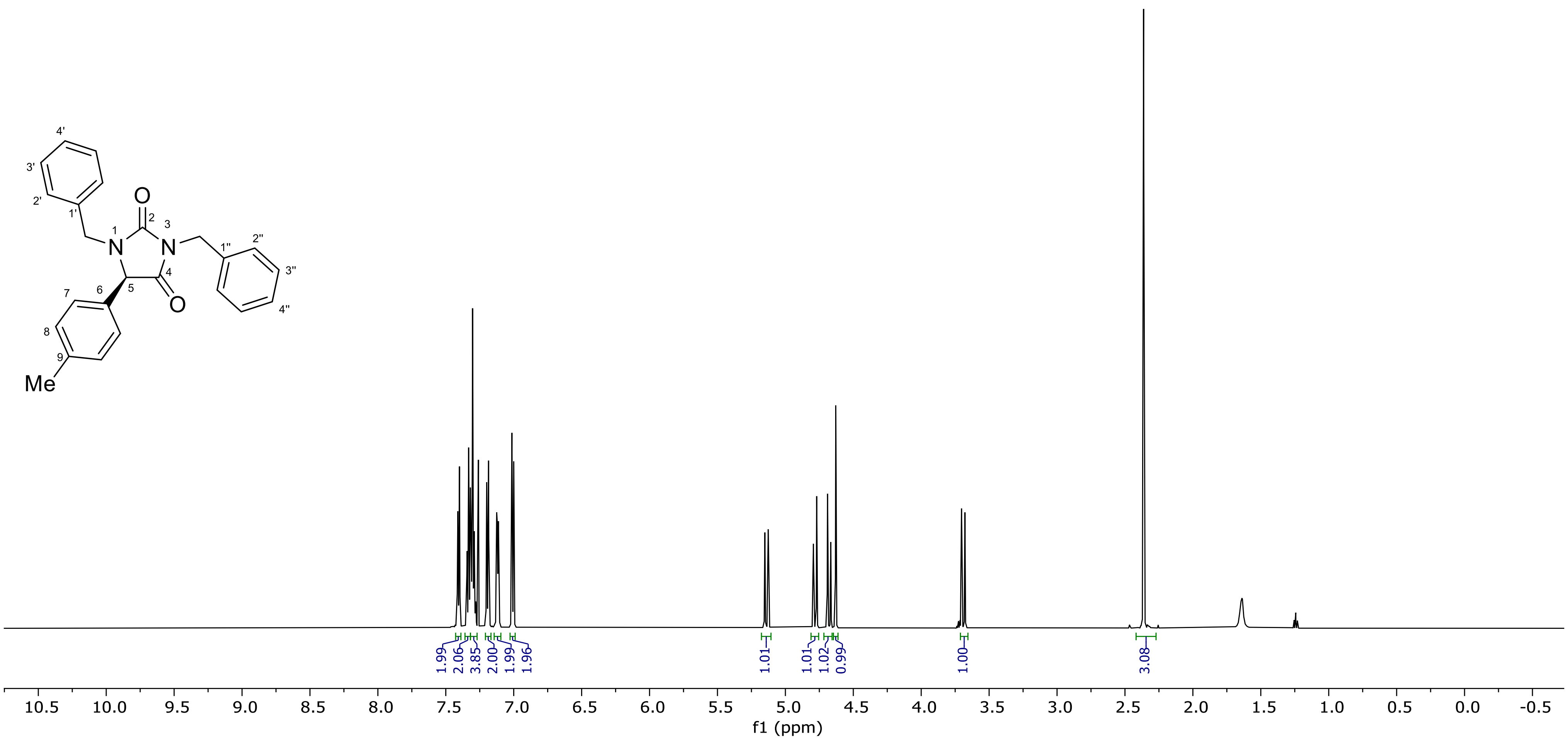

1,3-Dibenzyl-5-(4-methylphenyl)imidazolidine-2,4-dione 17b, ^{13}C NMR in $CDCl_3$

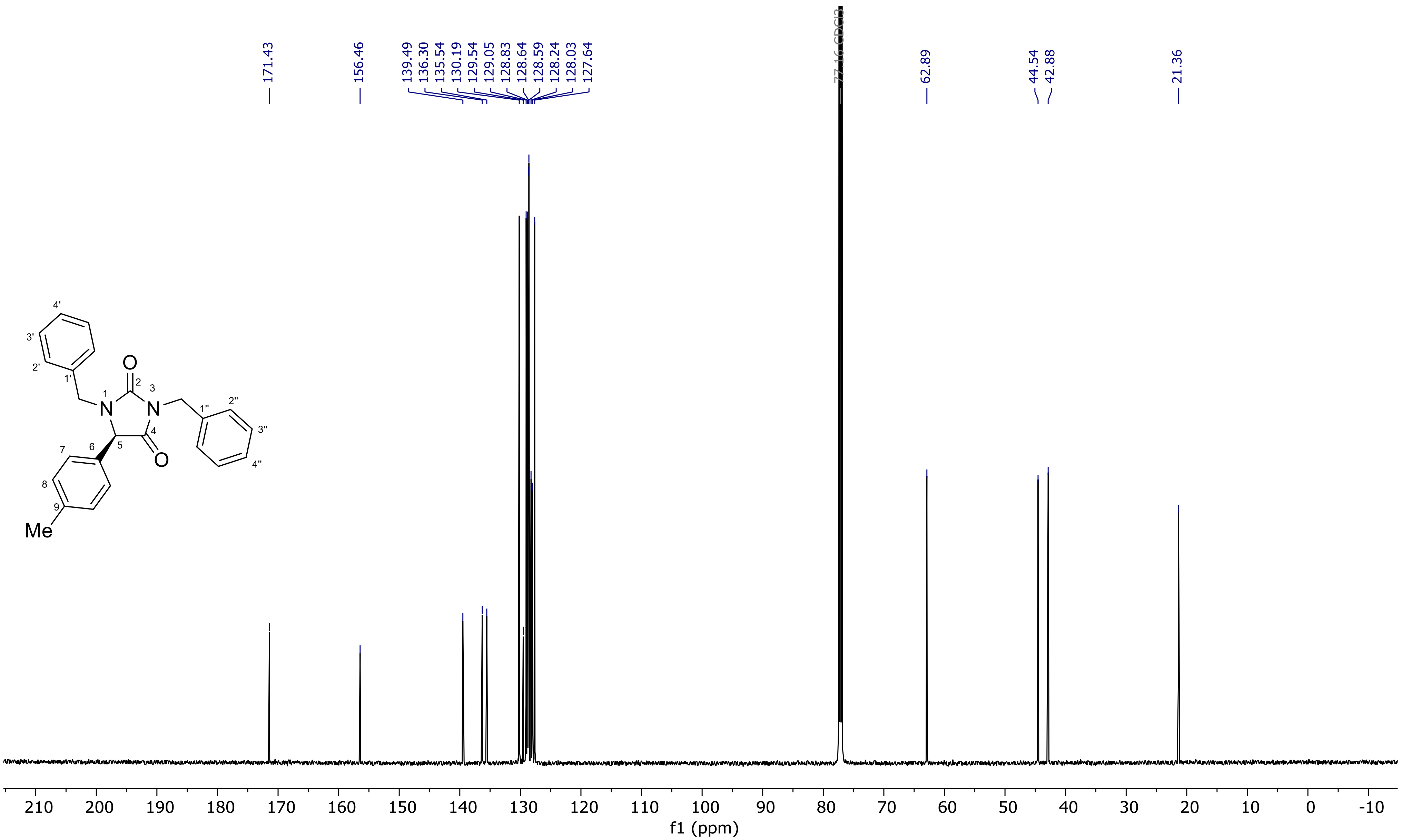

1,3-Dibenzyl-5-(4-hydroxyphenyl)imidazolidine-2,4-dione 17c, ^{1}H NMR in CDCl₃

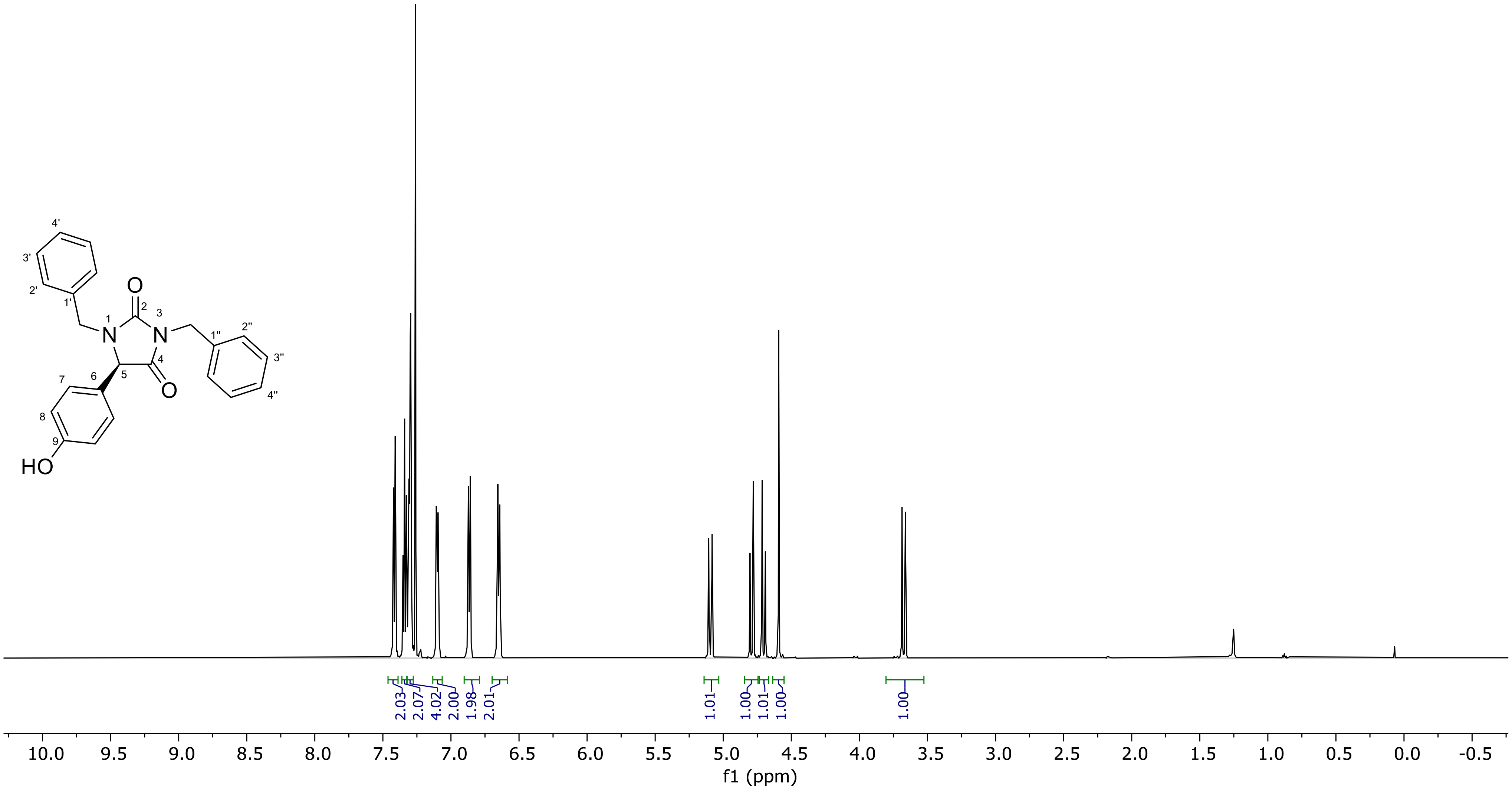

1,3-Dibenzyl-5-(4-hydroxyphenyl)imidazolidine-2,4-dione 17c, ^{13}C NMR in $CDCl_3$

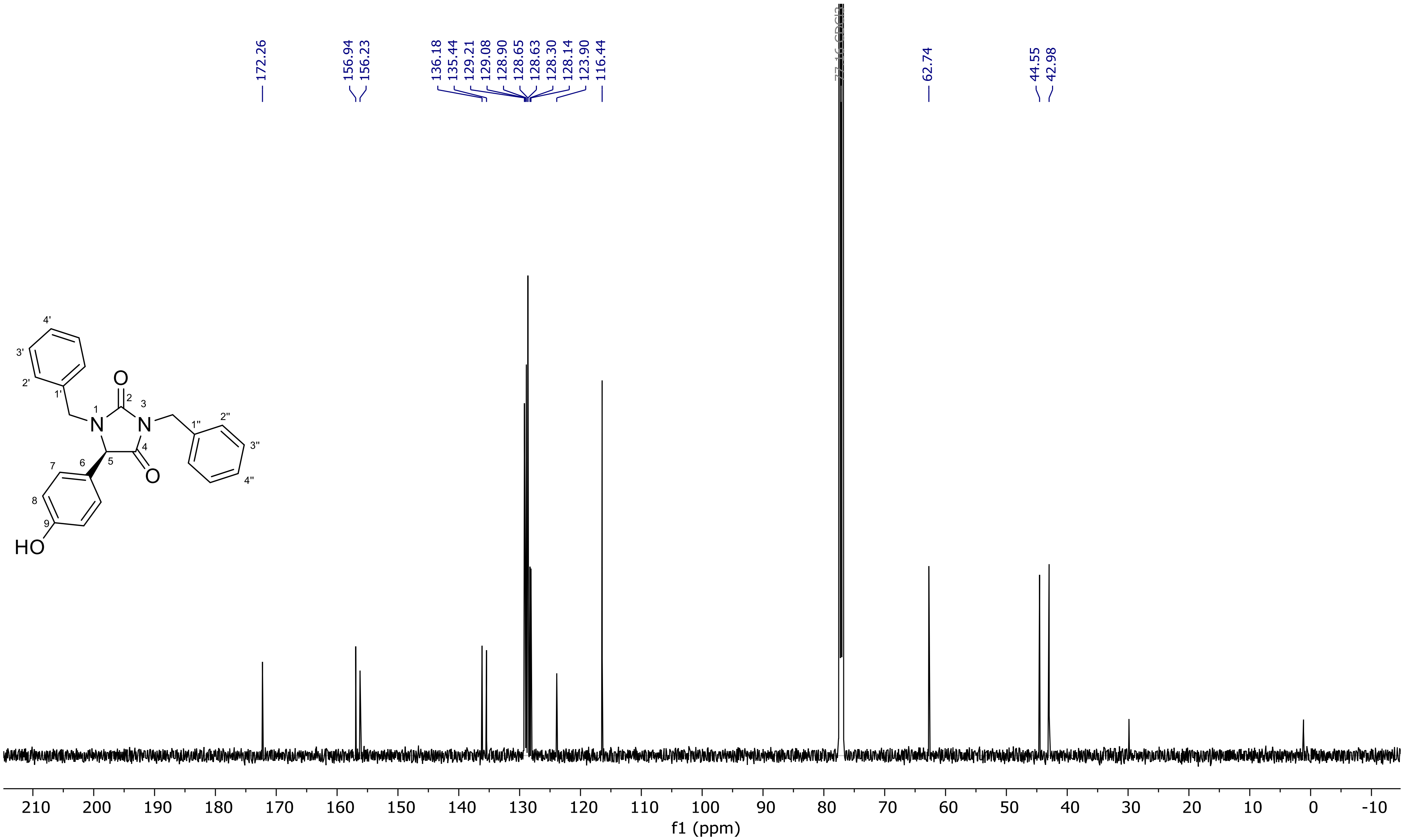

1,3-Dibenzyl-5-(4-methoxyphenyl)imidazolidine-2,4-dione 17d, ^{1}H NMR in $CDCl_3$

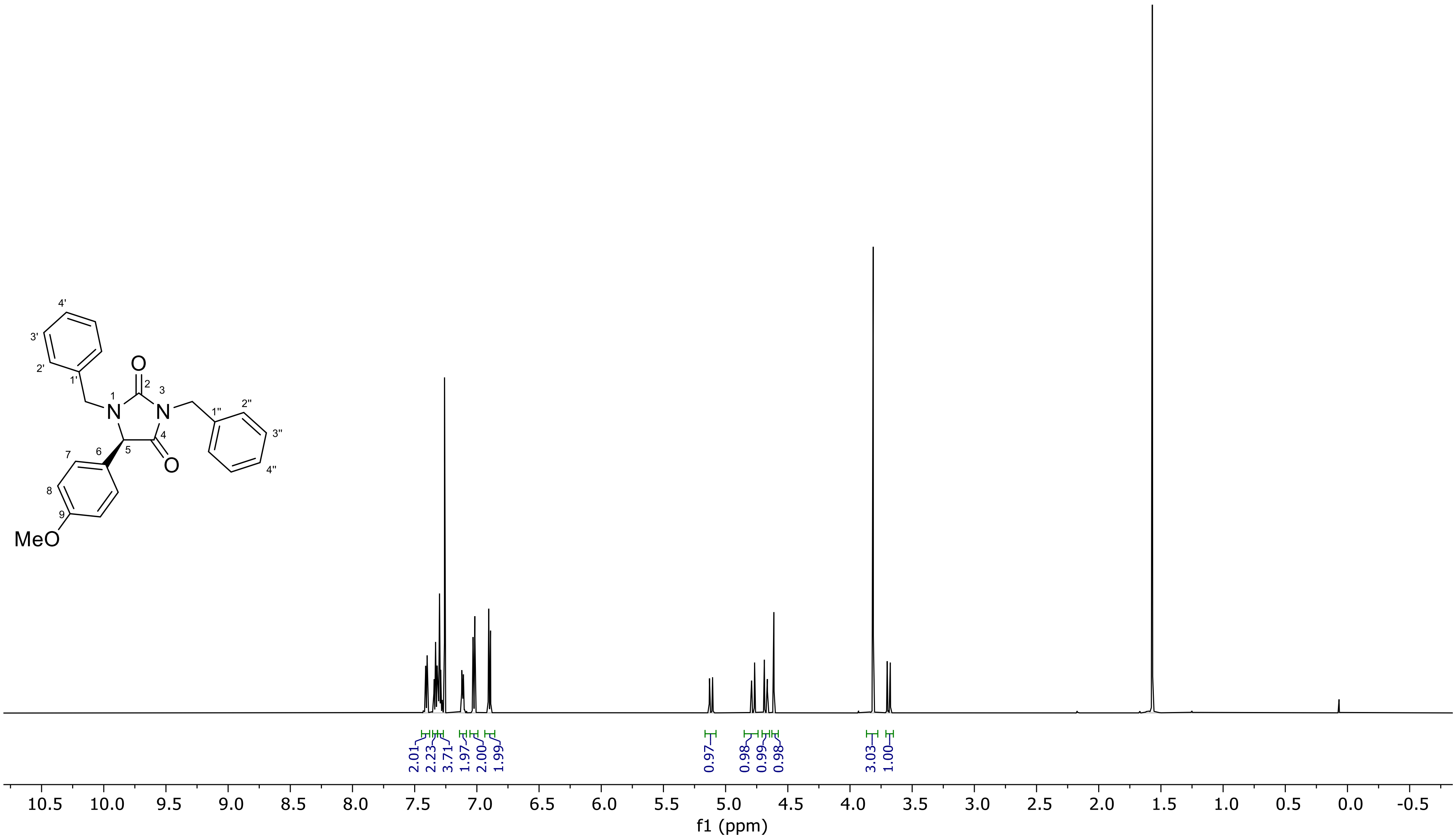

1,3-Dibenzyl-5-(4-methoxyphenyl)imidazolidine-2,4-dione 17d, ^{13}C NMR in $CDCl_3$

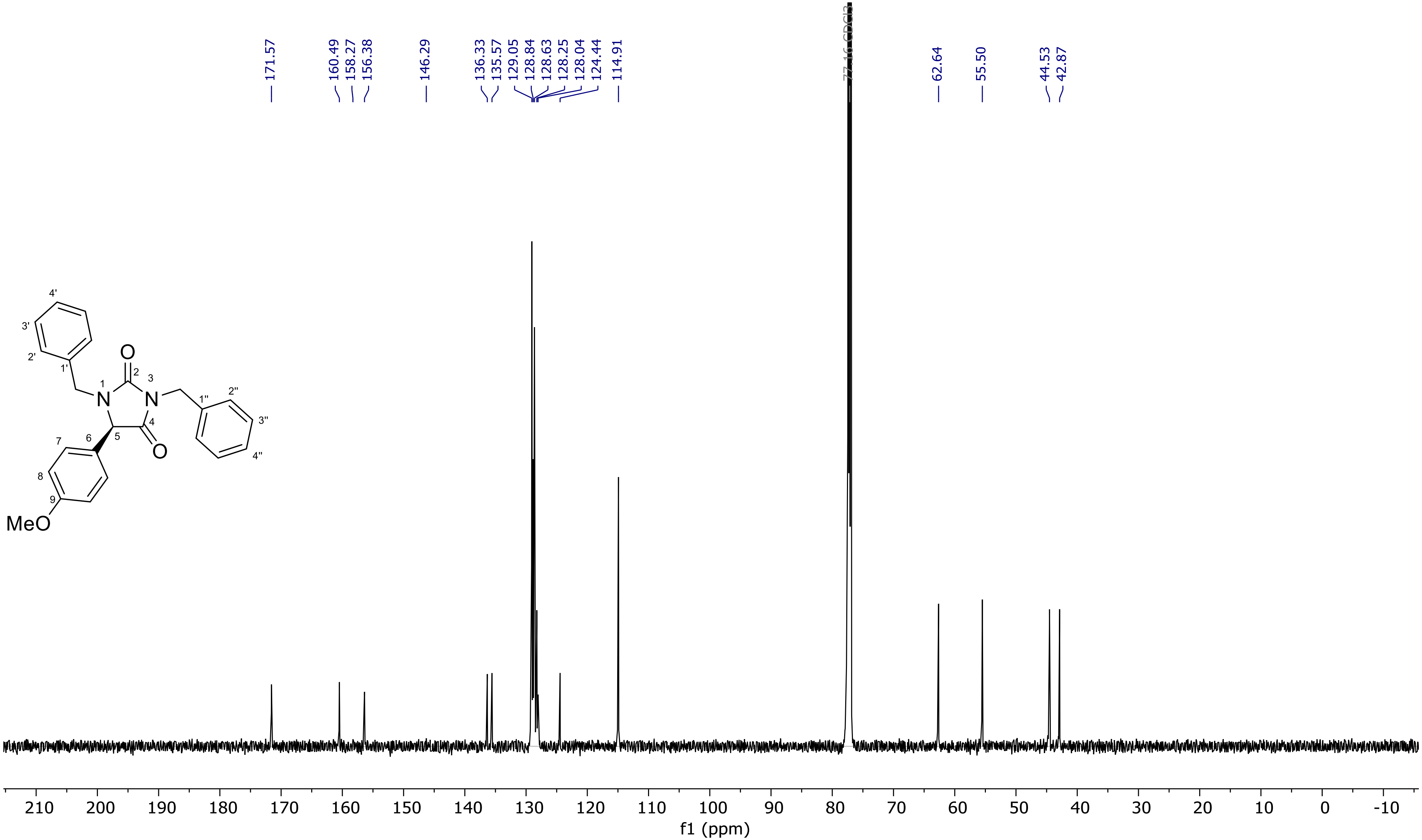

1,3-Dibenzyl-5-(4-phenoxyphenyl)imidazolidine-2,4-dione 17e, ¹H NMR in CDCl₃

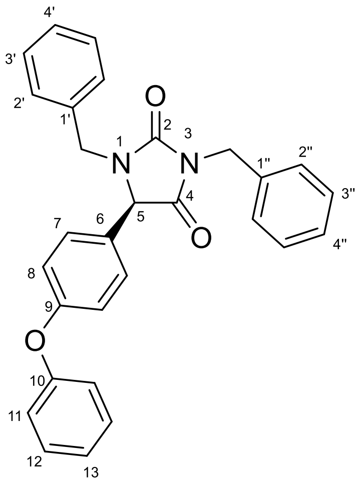

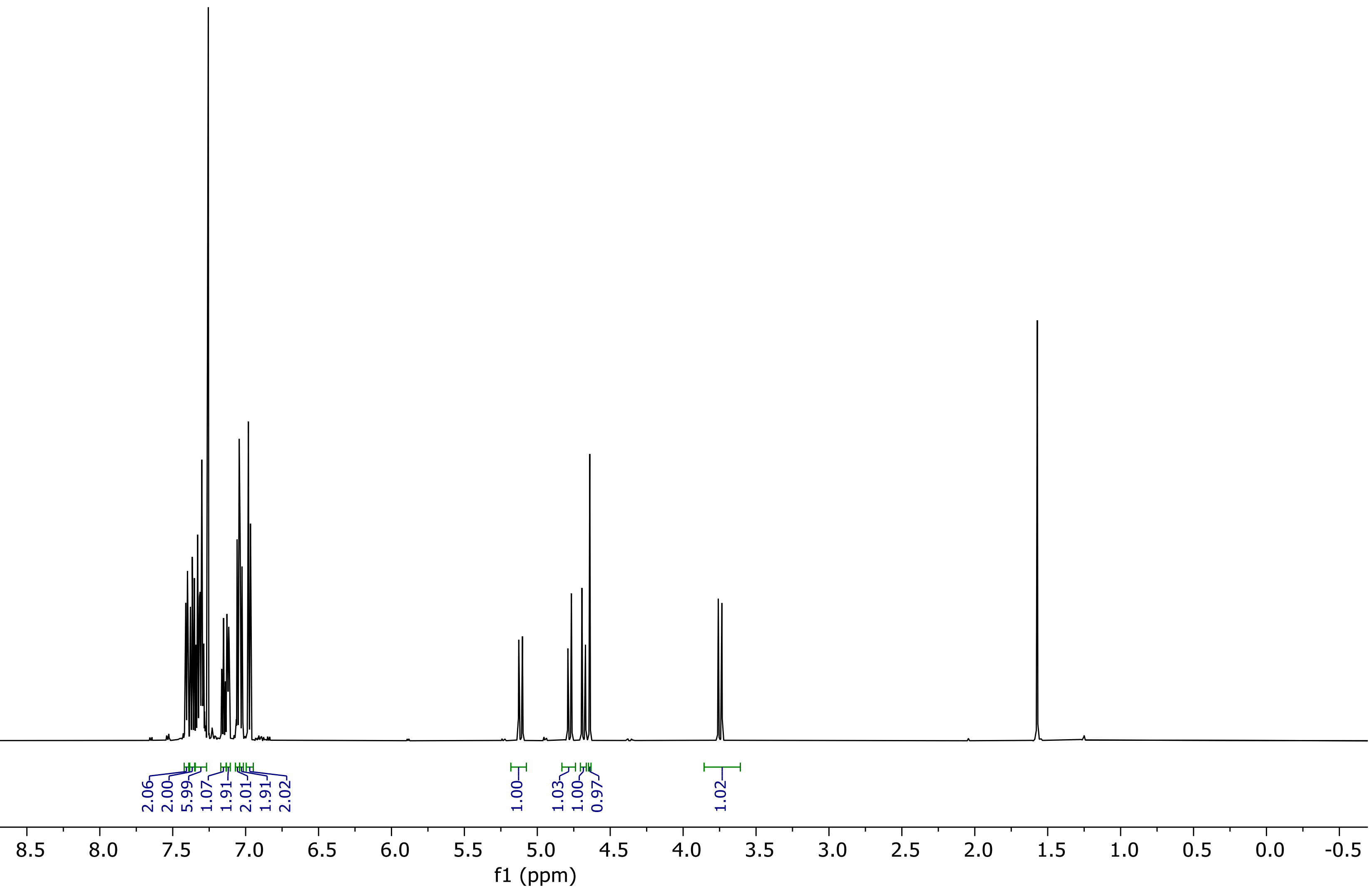

1,3-Dibenzyl-5-(4-phenoxyphenyl)imidazolidine-2,4-dione 17e, ^{13}C NMR in $CDCl_3$

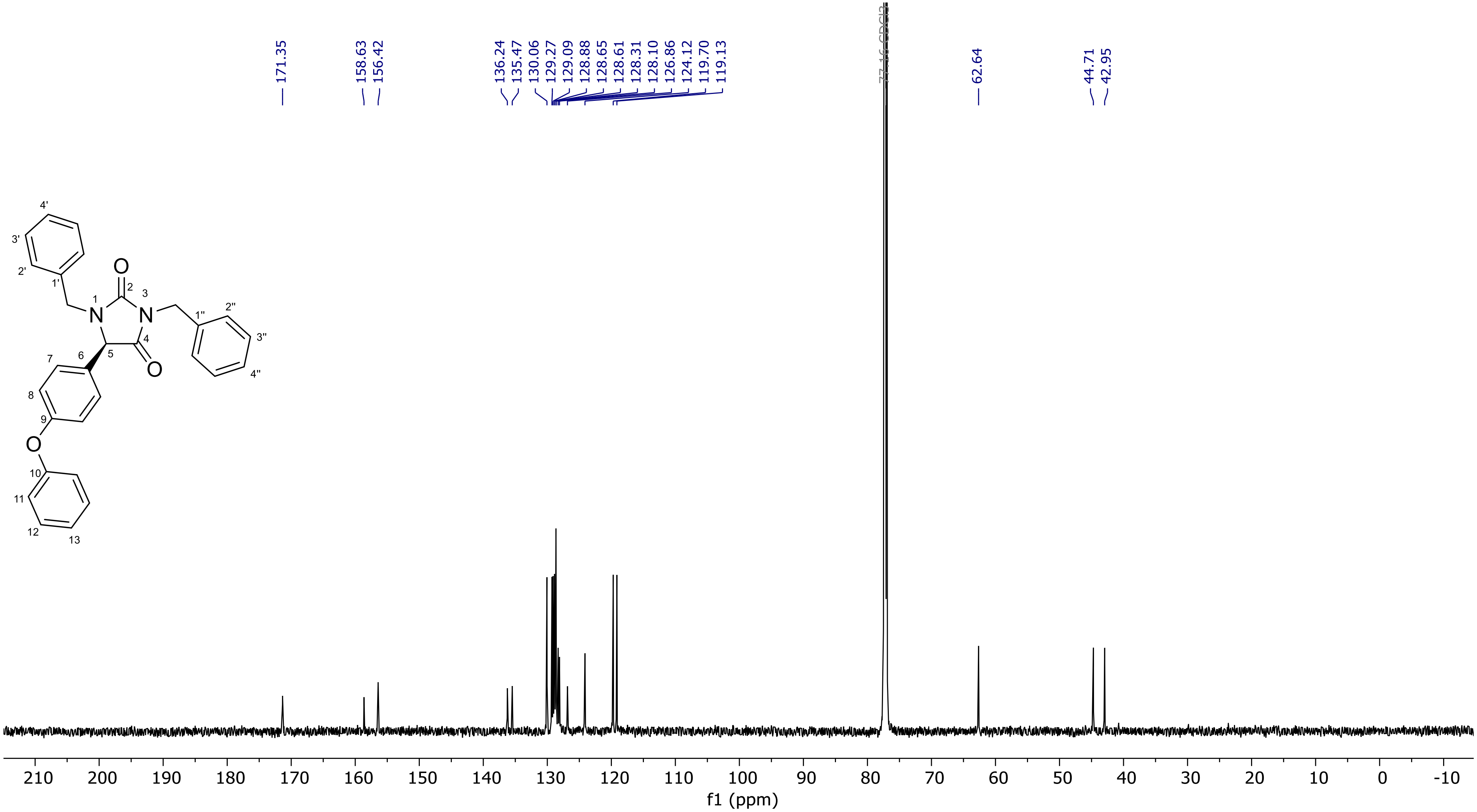

1,3-Dibenzyl-5-(4-fluorophenyl)imidazolidine-2,4-dione 17f, ^{1}H NMR in $CDCl_3$

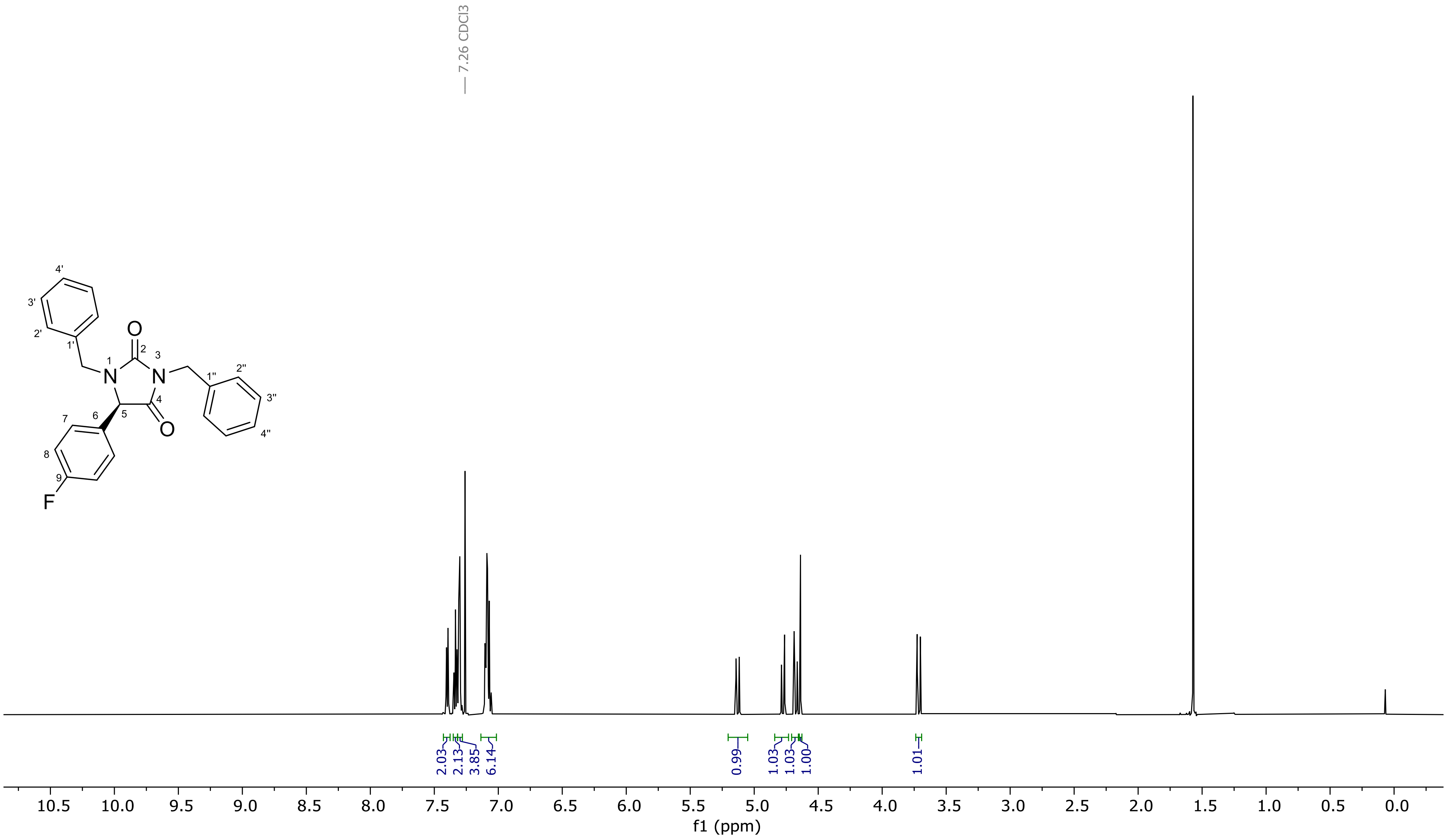

1,3-Dibenzyl-5-(4-fluorophenyl)imidazolidine-2,4-dione 17f, ^{13}C NMR in CDCl$_3$

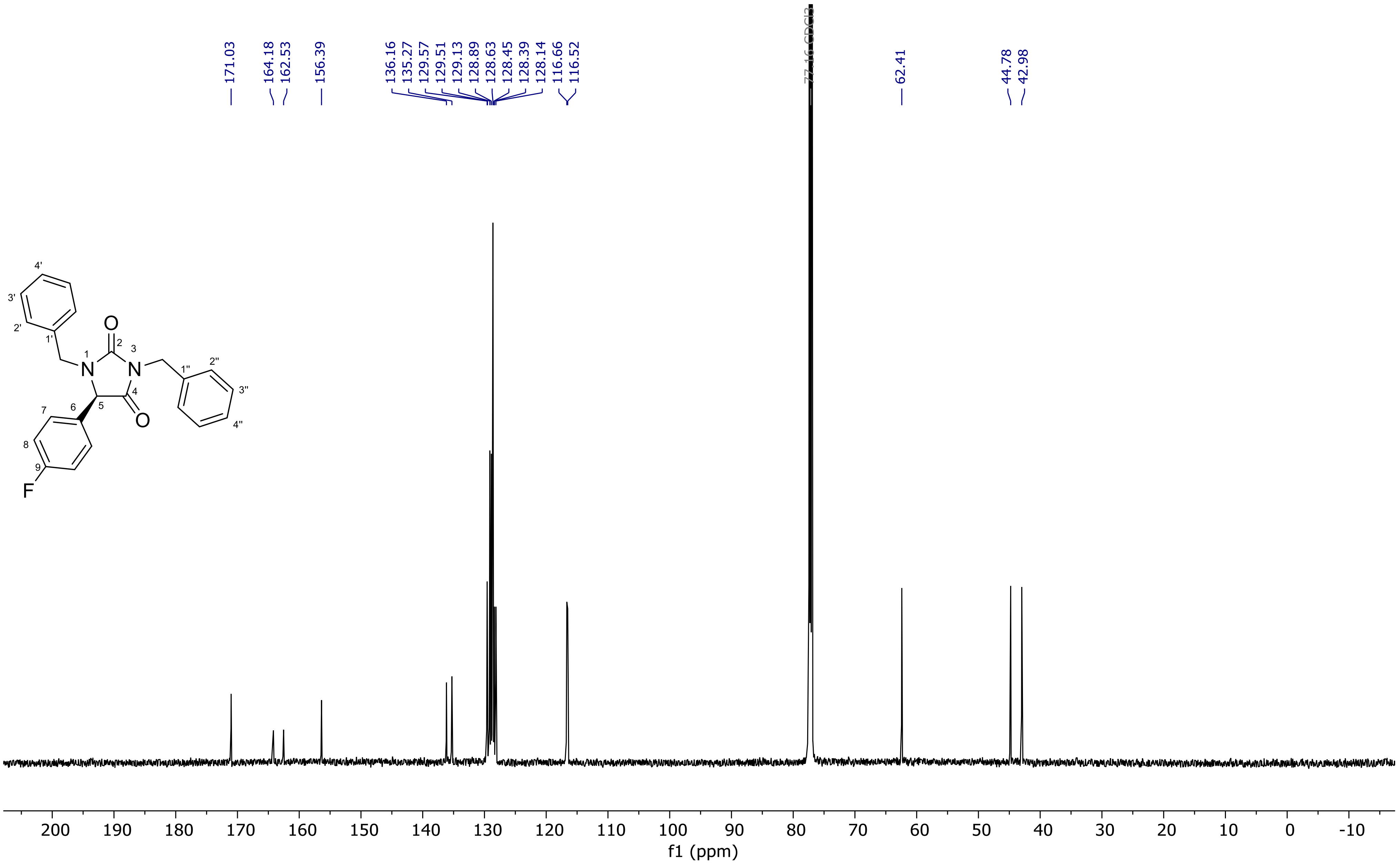

1,3-Dibenzyl-5-(4-fluorophenyl)imidazolidine-2,4-dione 17f, ^{19}F NMR in $CDCl_3$

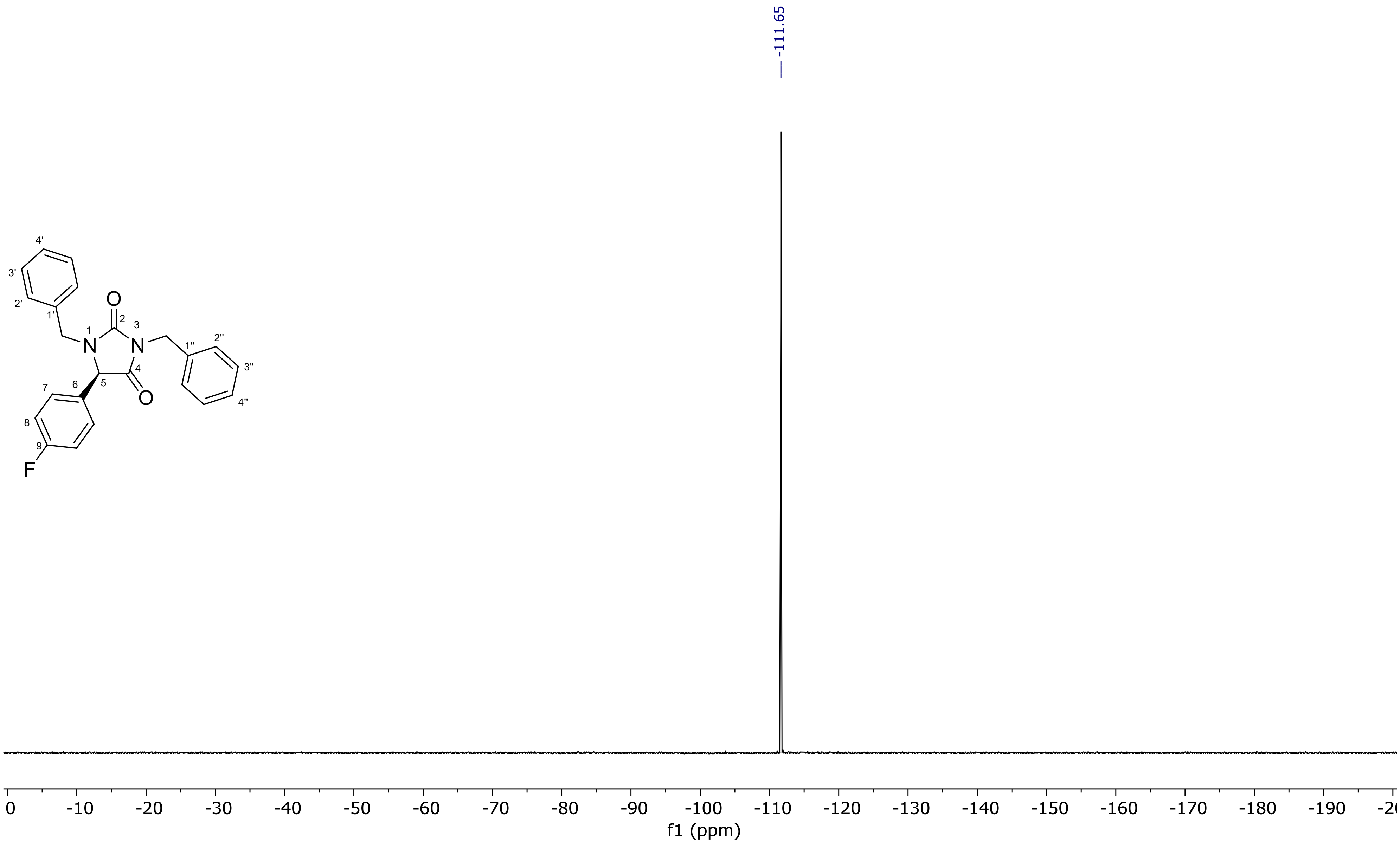

1,3-Dibenzyl-5-(4-chlorophenyl)imidazolidine-2,4-dione 17g, ^{1}H NMR in CDCl$_3$

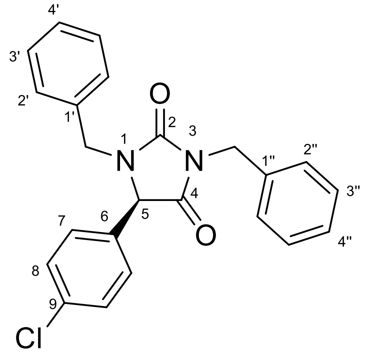

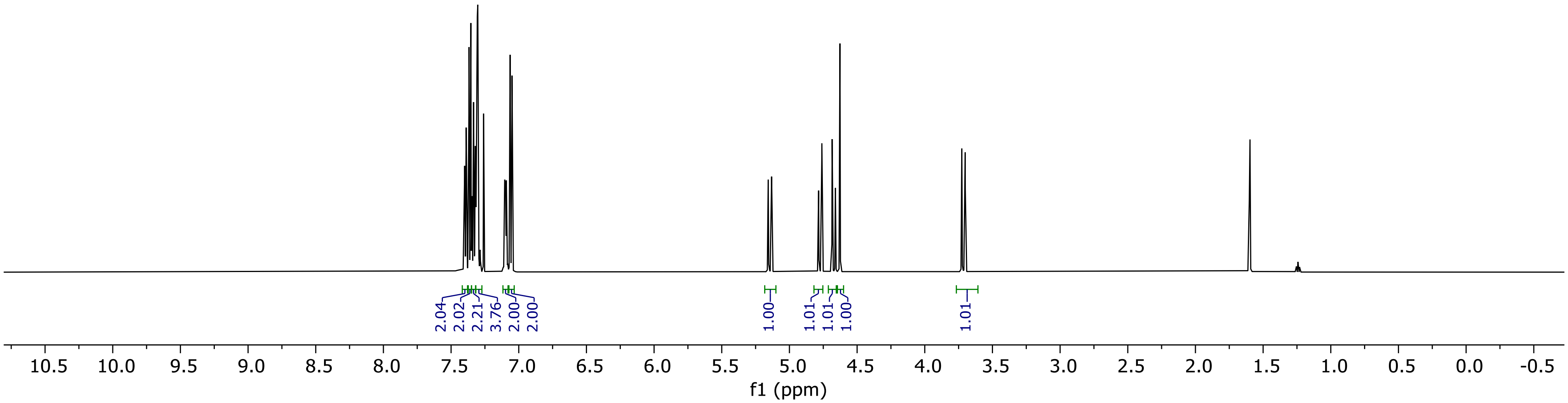

1,3-Dibenzyl-5-(4-chlorophenyl)imidazolidine-2,4-dione 17g, ^{13}C NMR in $CDCl_3$

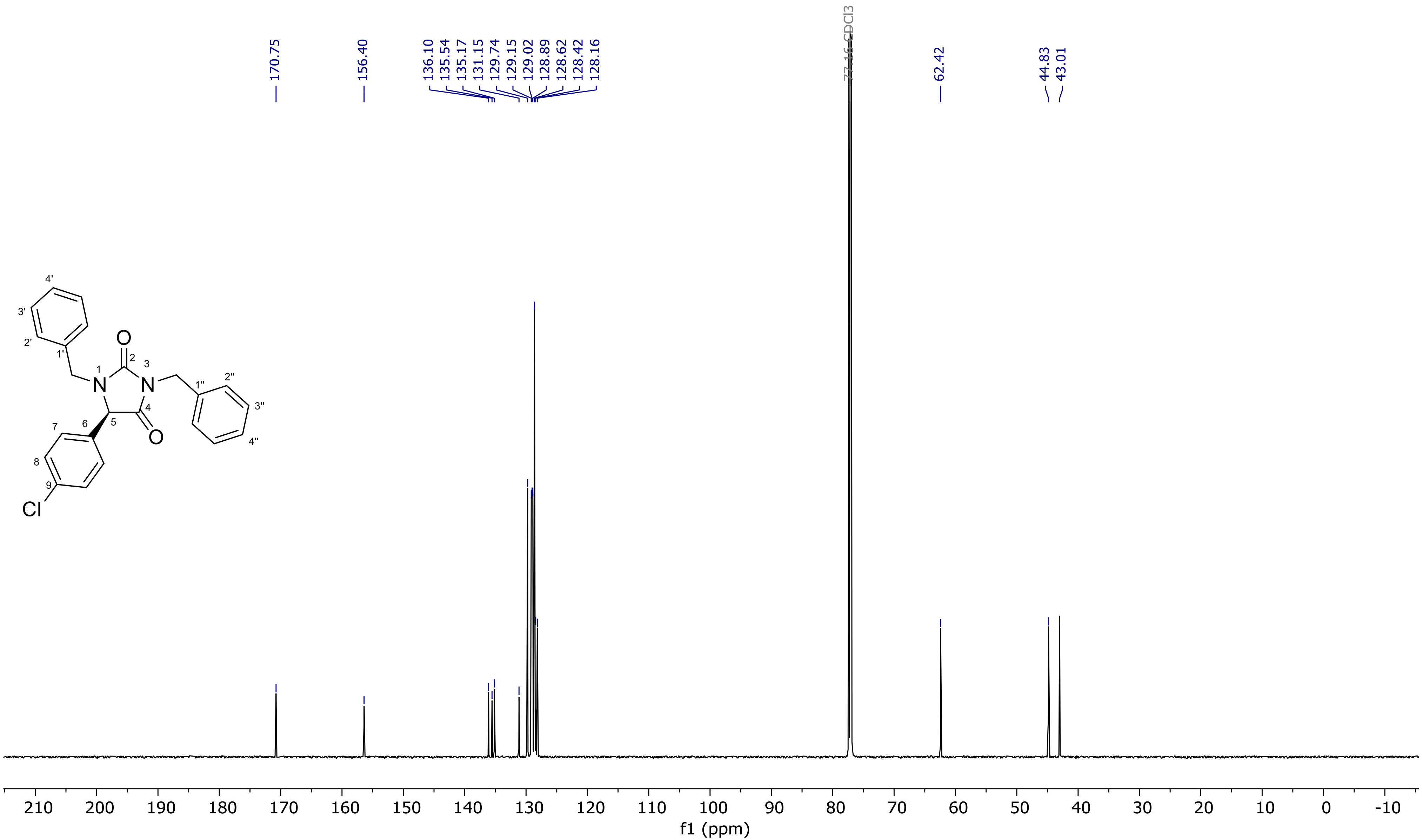

1,3-Dibenzyl-5-(4-bromophenyl)imidazolidine-2,4-dione 17h, ^{1}H NMR in $CDCl_3$

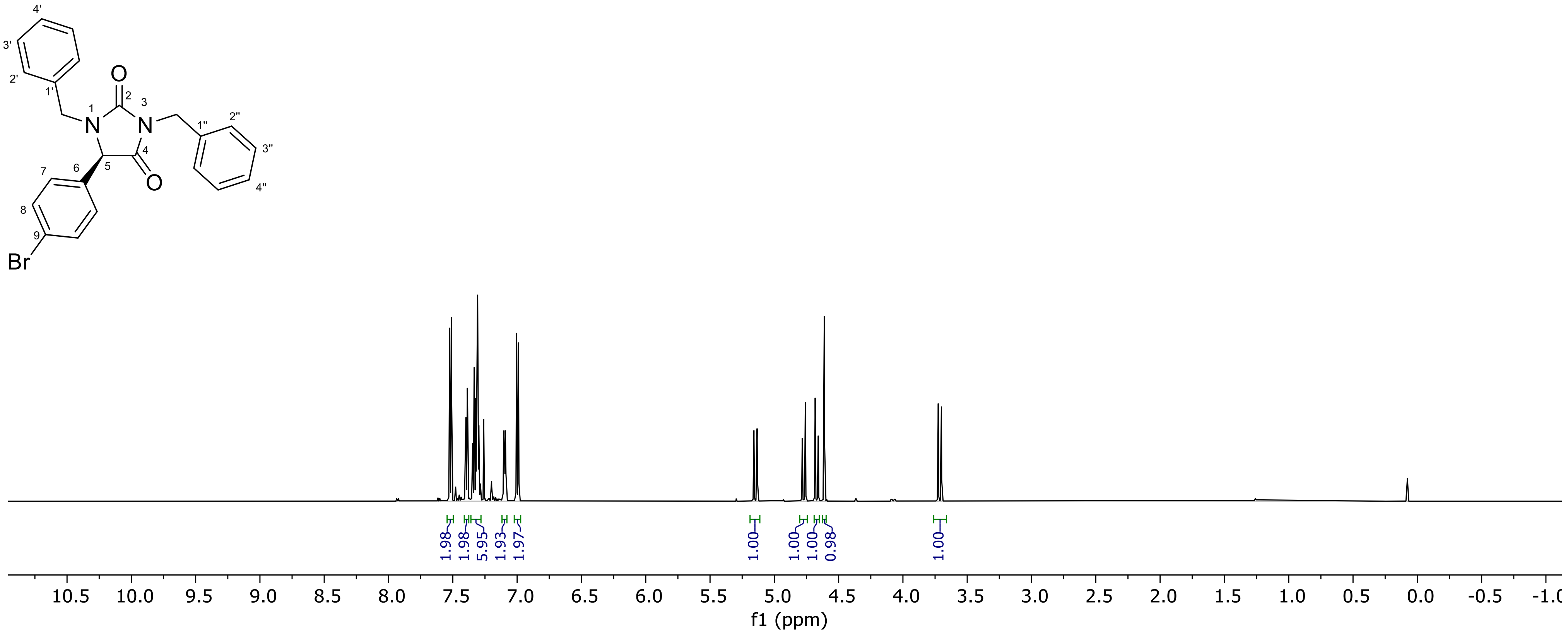

1,3-Dibenzyl-5-(4-bromophenyl)imidazolidine-2,4-dione 17h, ^{13}C NMR in $CDCl_3$

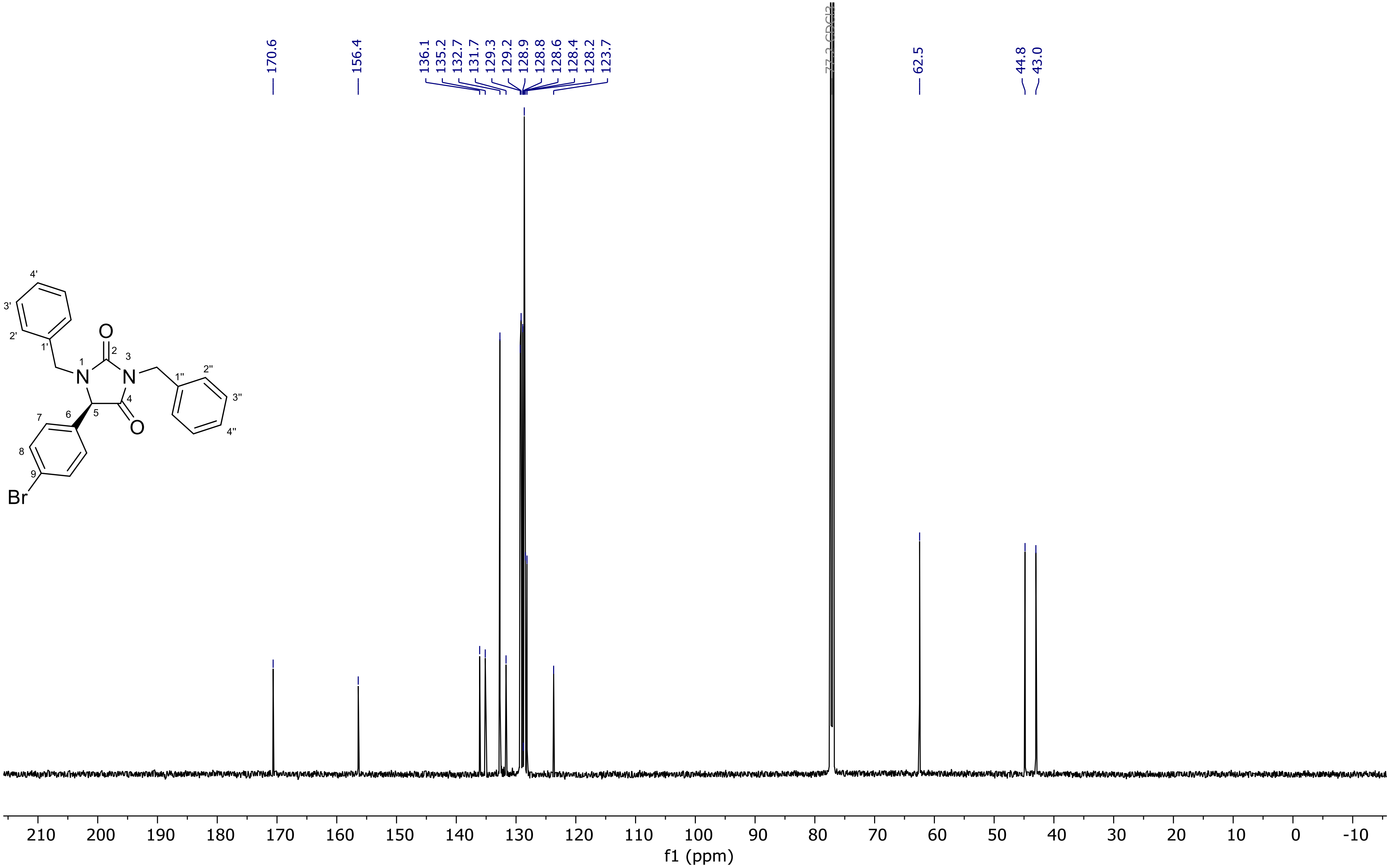

1,3-Dibenzyl-5-(4-iodophenyl)imidazolidine-2,4-dione 17i, ^{1}H NMR in CDCl$_3$

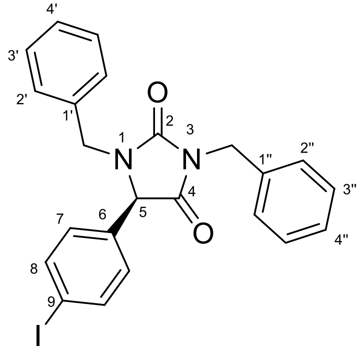

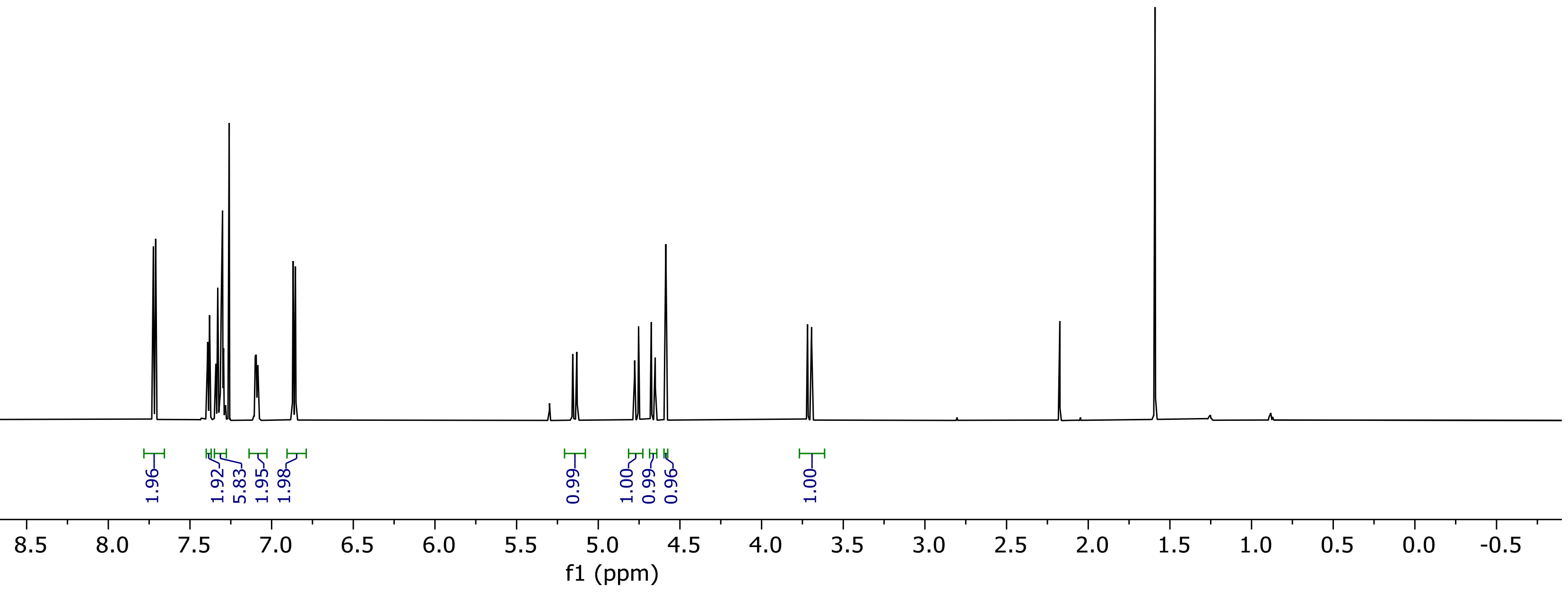

1,3-Dibenzyl-5-(4-iodophenyl)imidazolidine-2,4-dione 17i, ^{13}C NMR in $CDCl_3$

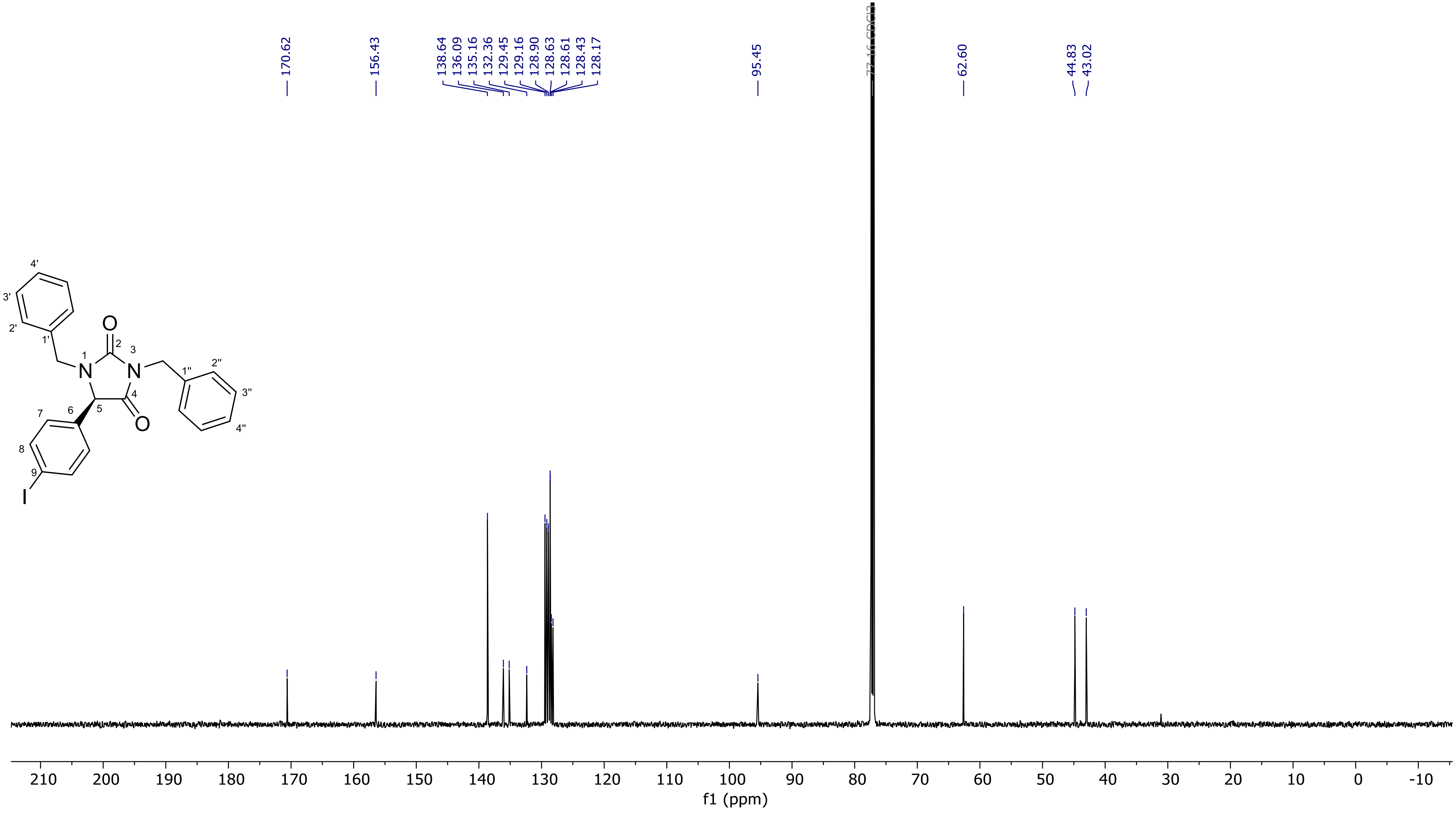

1,3-Dibenzyl-5-(4-(trifluoromethyl)phenyl)imidazolidine-2,4-dione 17j, ^{1}H NMR in $CDCl_3$

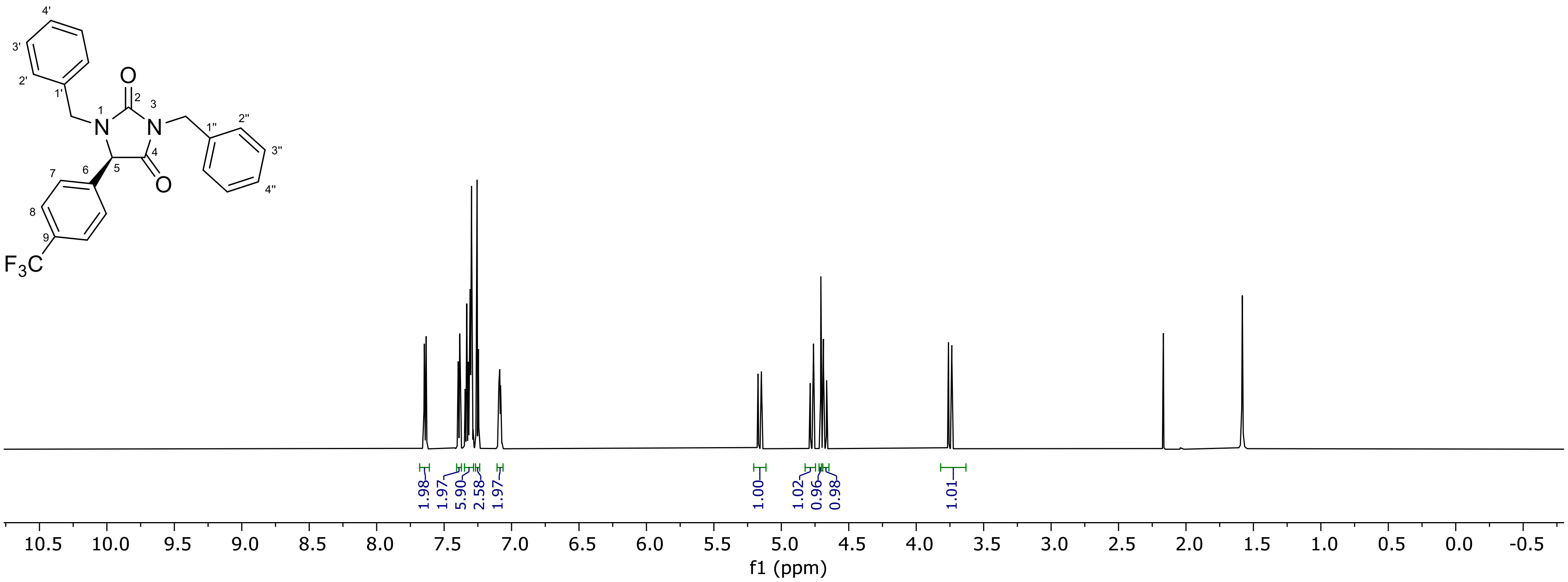

1,3-Dibenzyl-5-(4-(trifluoromethyl)phenyl)imidazolidine-2,4-dione 17j, ^{13}C NMR in $CDCl_3$

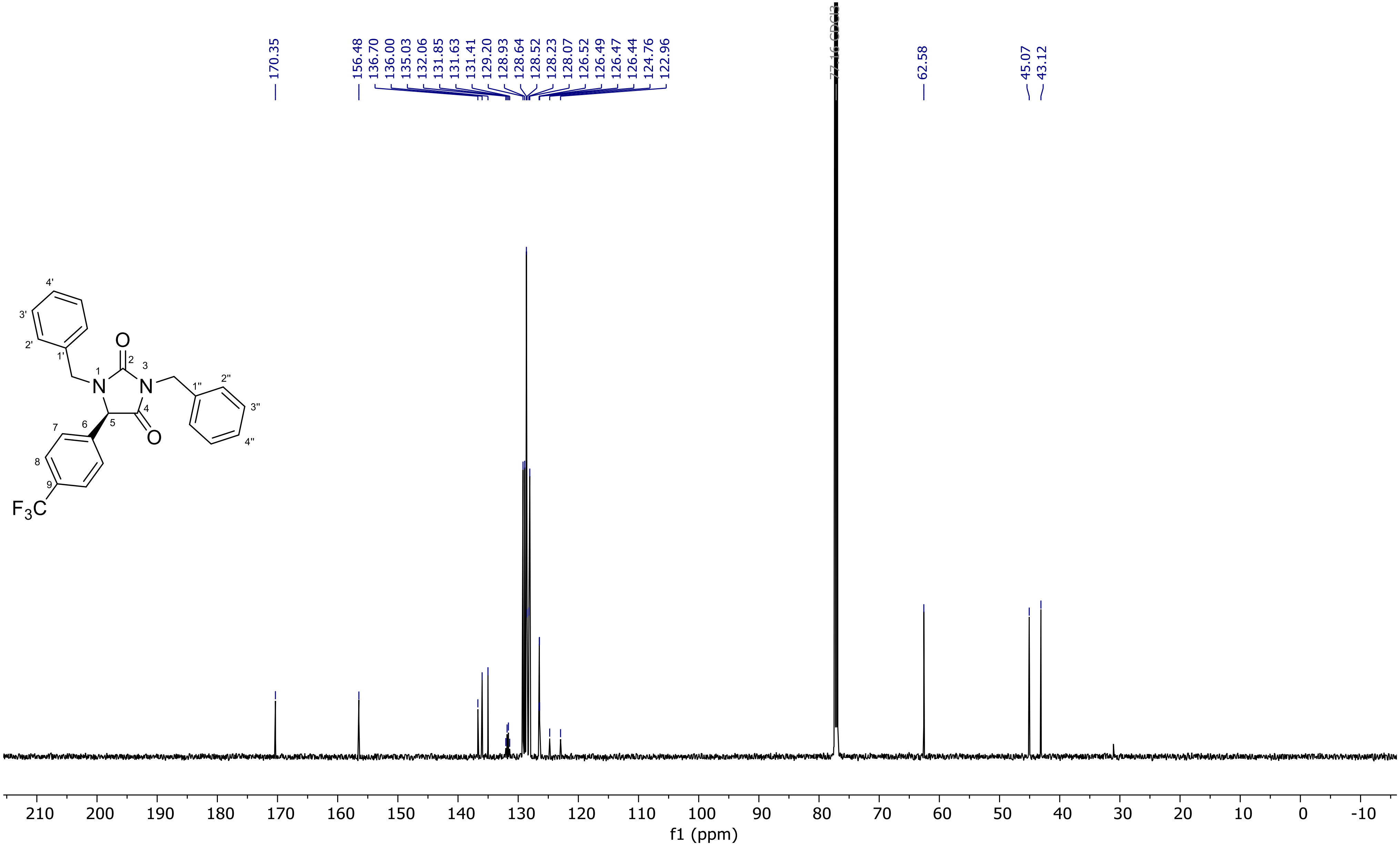

1,3-Dibenzyl-5-(4-(trifluoromethyl)phenyl)imidazolidine-2,4-dione 17j, ^{19}F NMR in $CDCl_3$

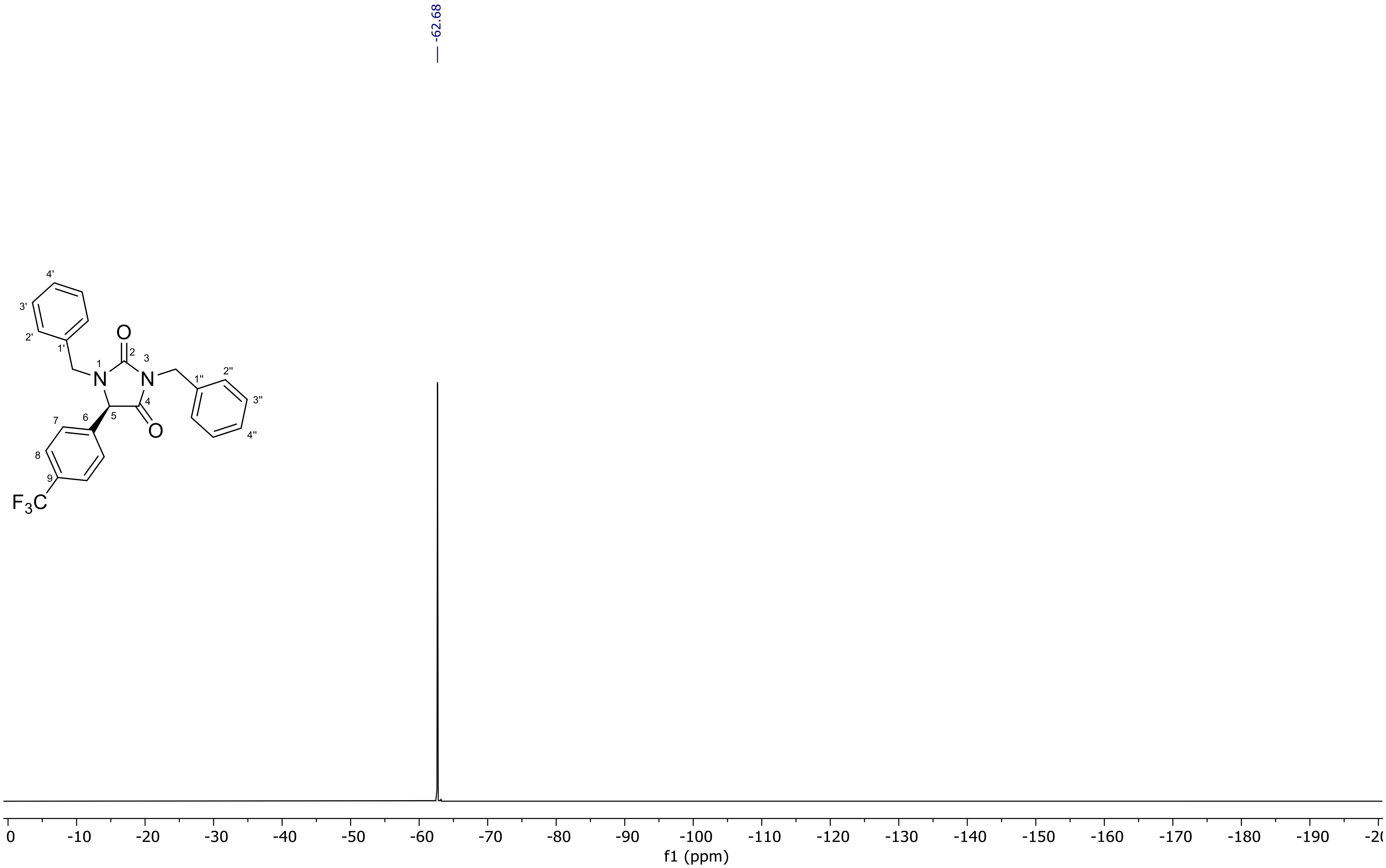

1,3-Dibenzyl-5-(4-nitrophenyl)imidazolidine-2,4-dione 17k, 1H NMR in $CDCl_3$

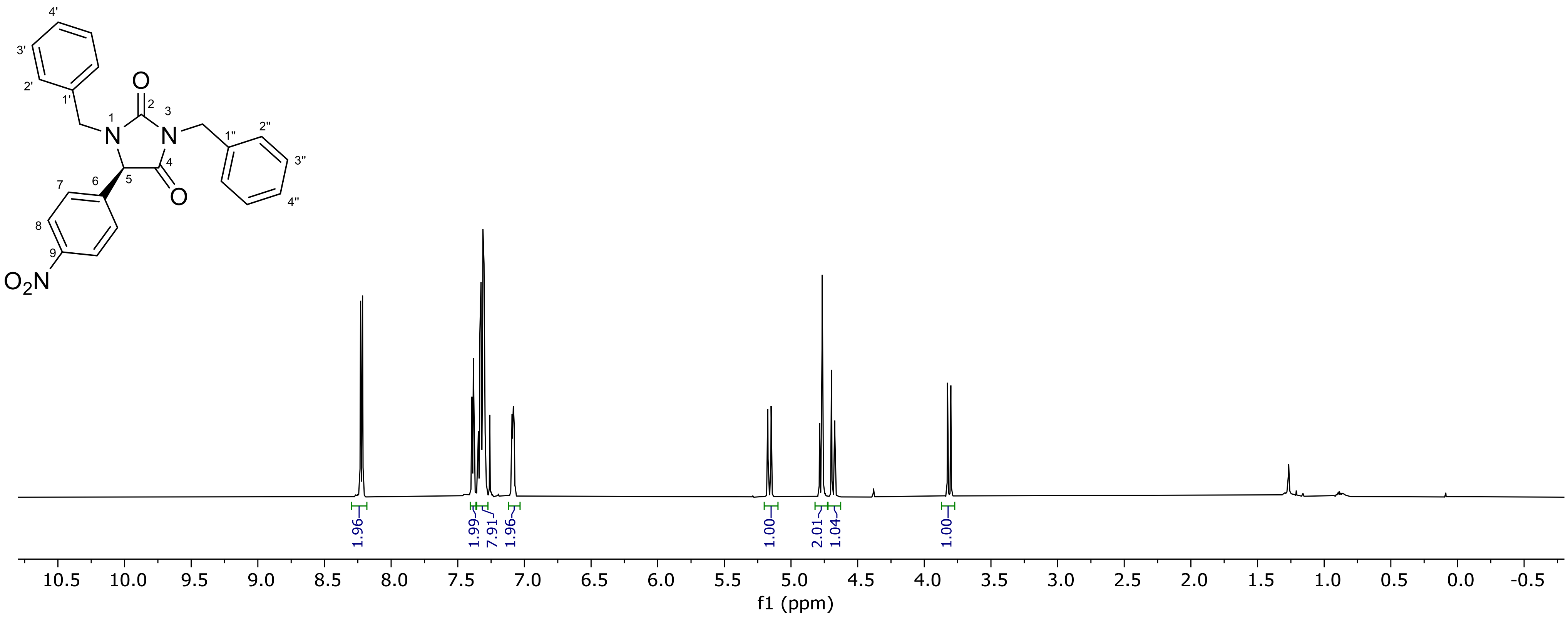

1,3-Dibenzyl-5-(4-nitrophenyl)imidazolidine-2,4-dione 17k, ^{13}C NMR in CDCl$_3$

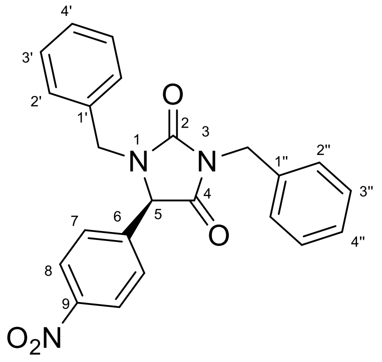

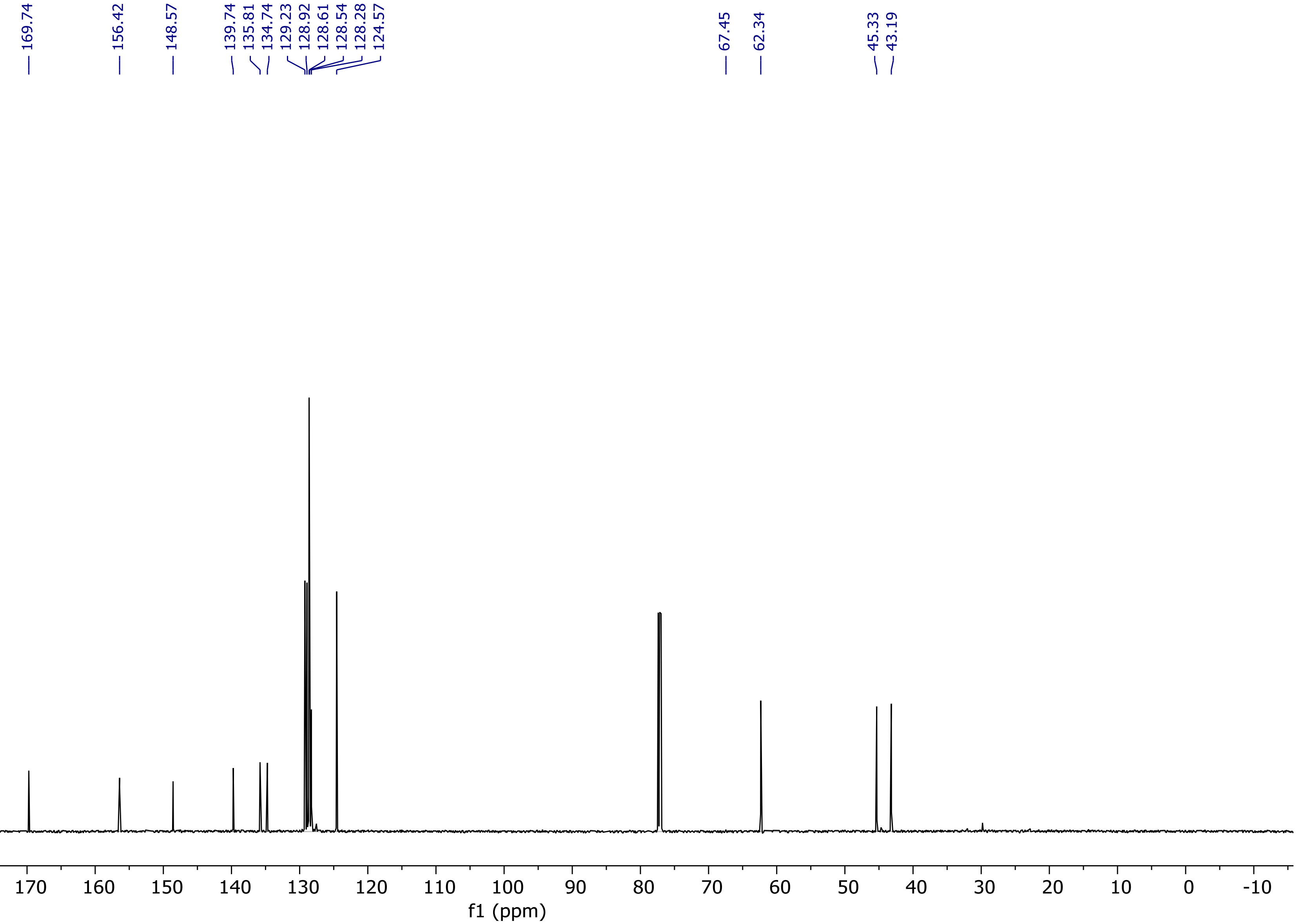

1,3-Dibenzyl-5-(3-methoxyphenyl)imidazolidine-2,4-dione 17l, ^{1}H NMR in $CDCl_3$

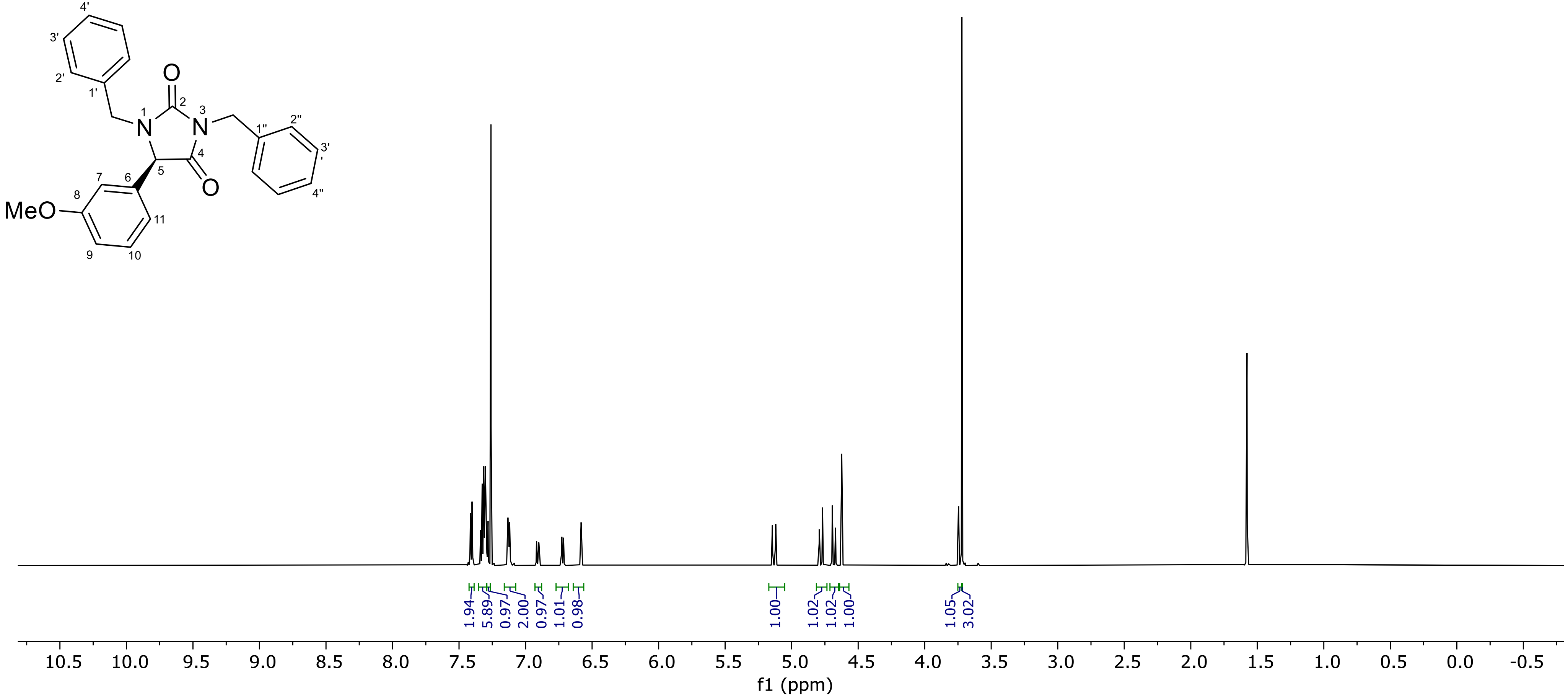

1,3-Dibenzyl-5-(3-methoxyphenyl)imidazolidine-2,4-dione 17l, ^{13}C NMR in CDCl$_3$

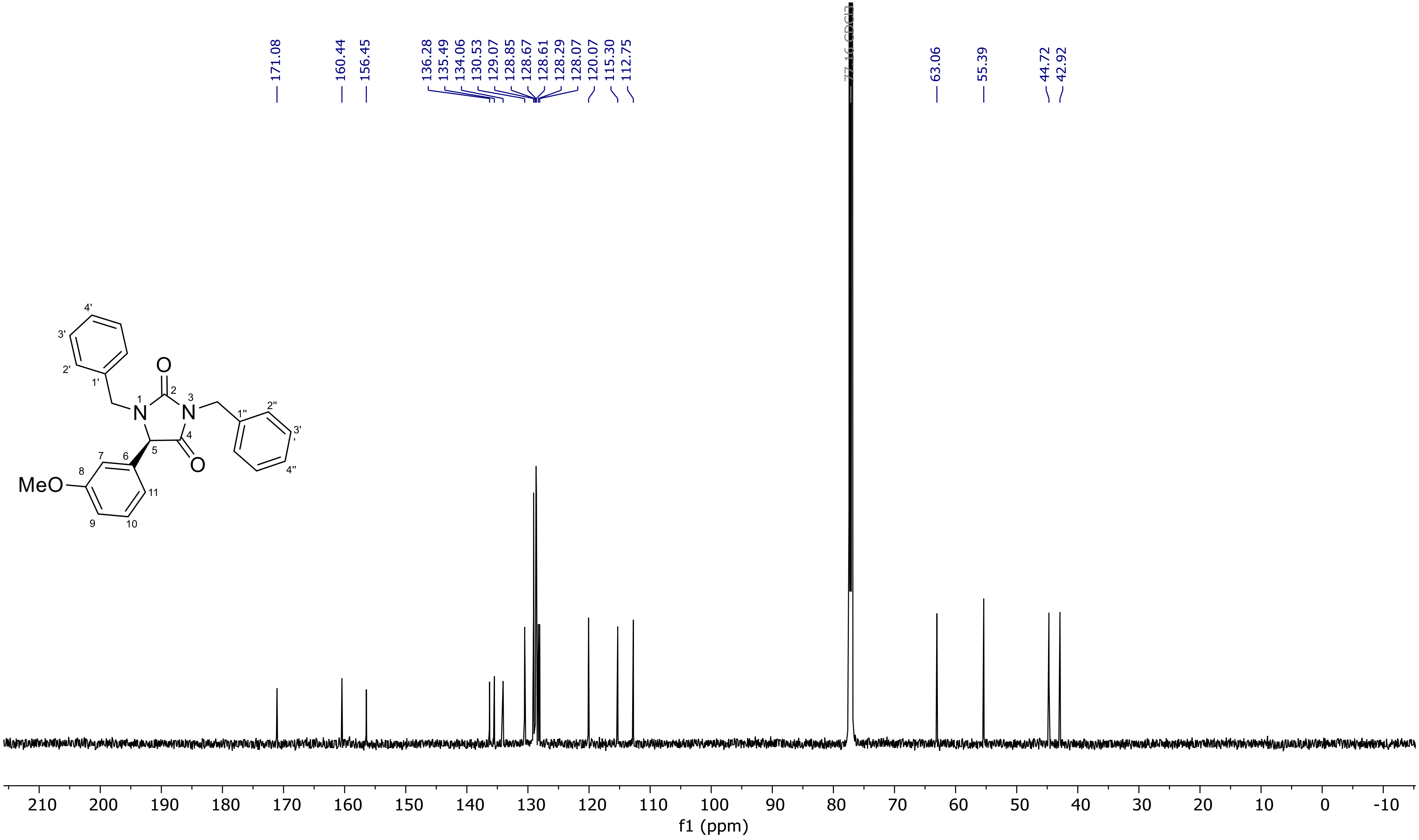

1,3-Dibenzyl-5-(3-chlorophenyl)imidazolidine-2,4-dione 17m, ^{1}H NMR in $CDCl_3$

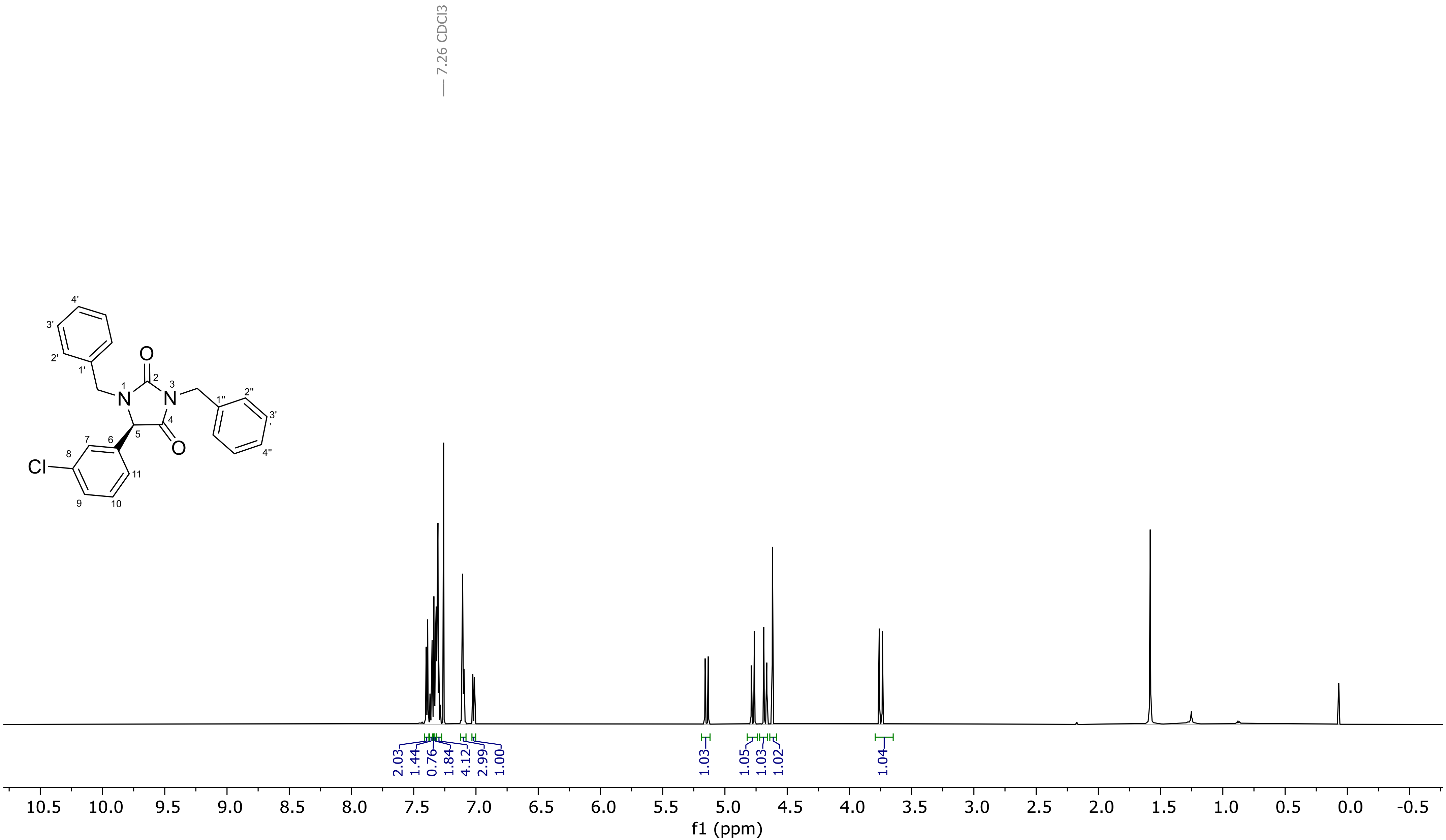

1,3-Dibenzyl-5-(3-chlorophenyl)imidazolidine-2,4-dione 17m, ^{13}C NMR in $CDCl_3$

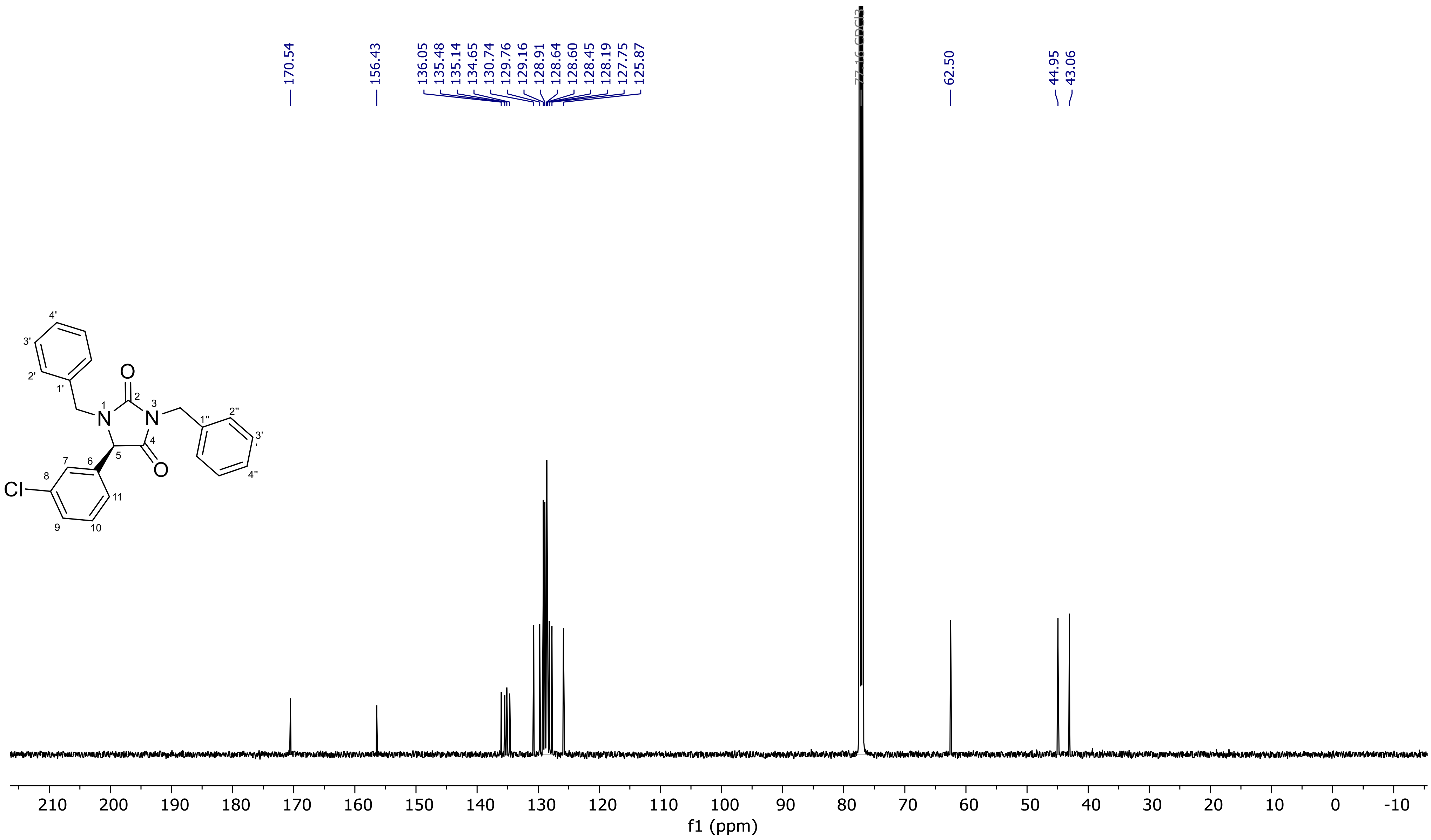

1,3-Dibenzyl-5-(3,4-difluorophenyl)imidazolidine-2,4-dione 17n, ^{1}H NMR in CDCl₃

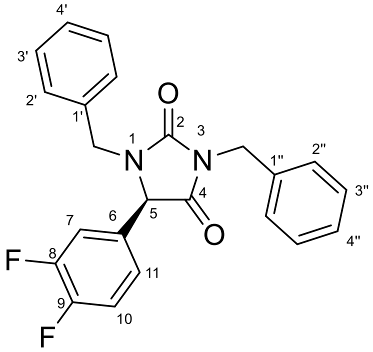

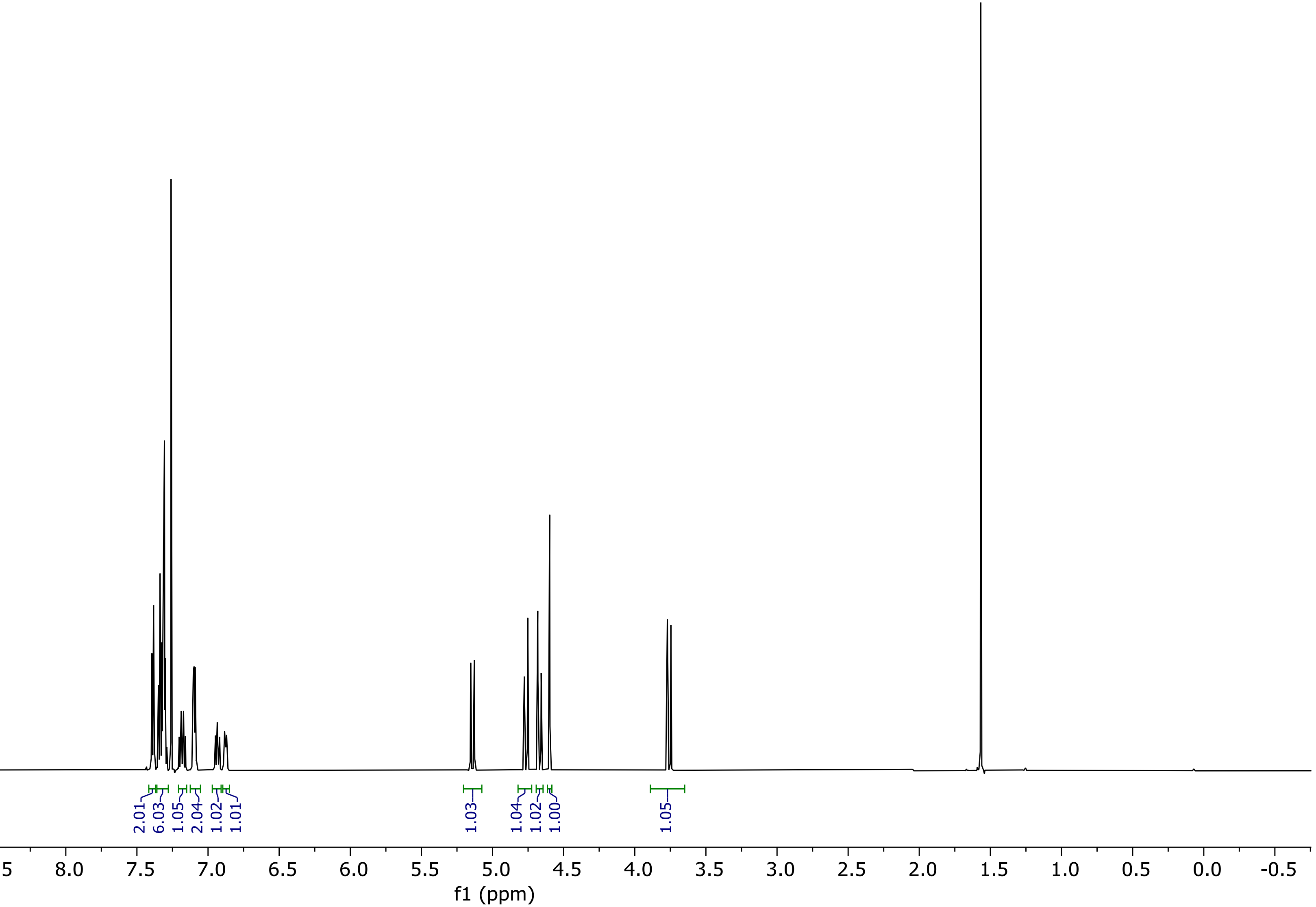

1,3-Dibenzyl-5-(3,4-difluorophenyl)imidazolidine-2,4-dione 17n, ^{13}C NMR in CDCl$_3$

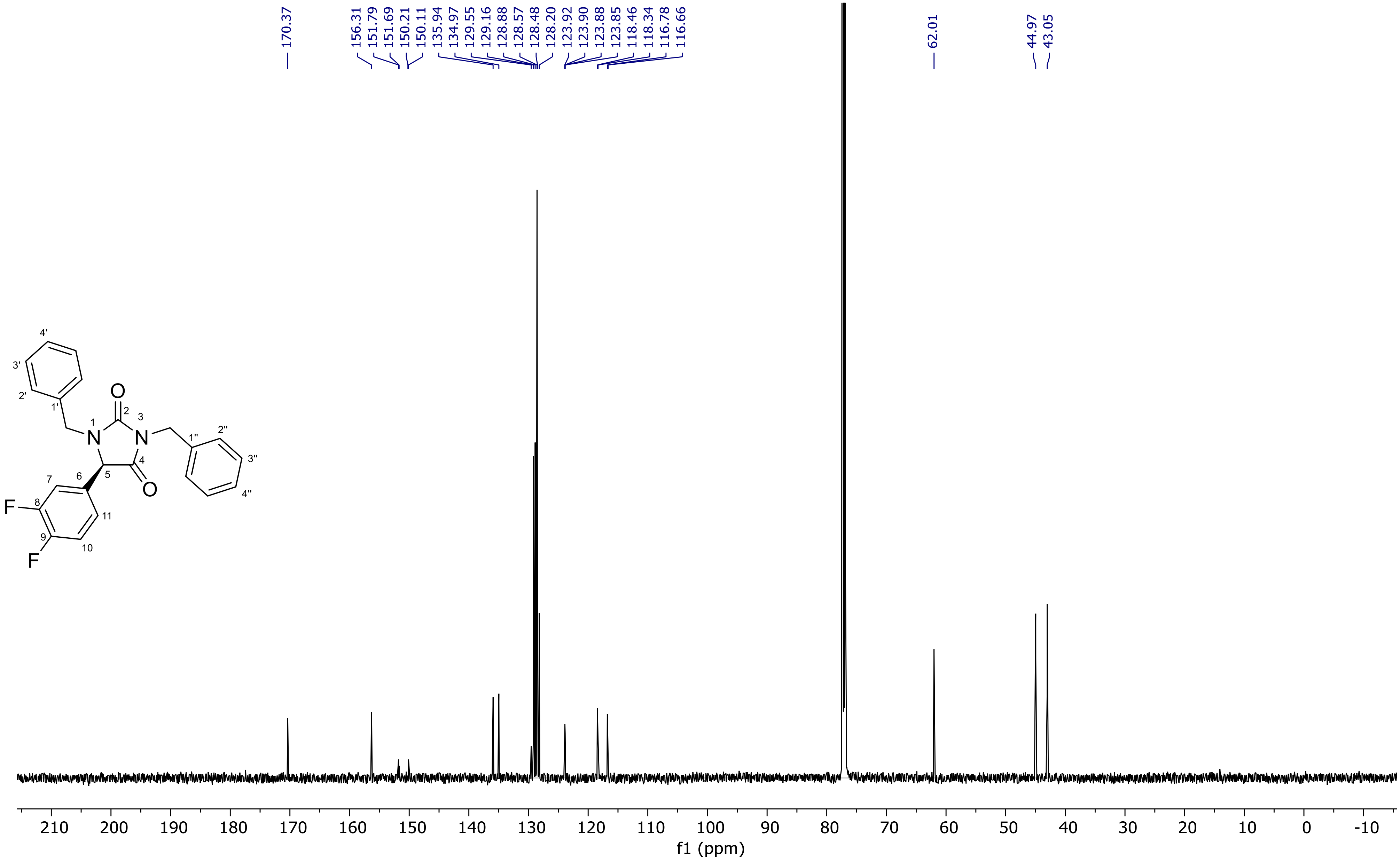

1,3-Dibenzyl-5-(3,4-difluorophenyl)imidazolidine-2,4-dione 17n, ^{19}F NMR in CDCl$_3$

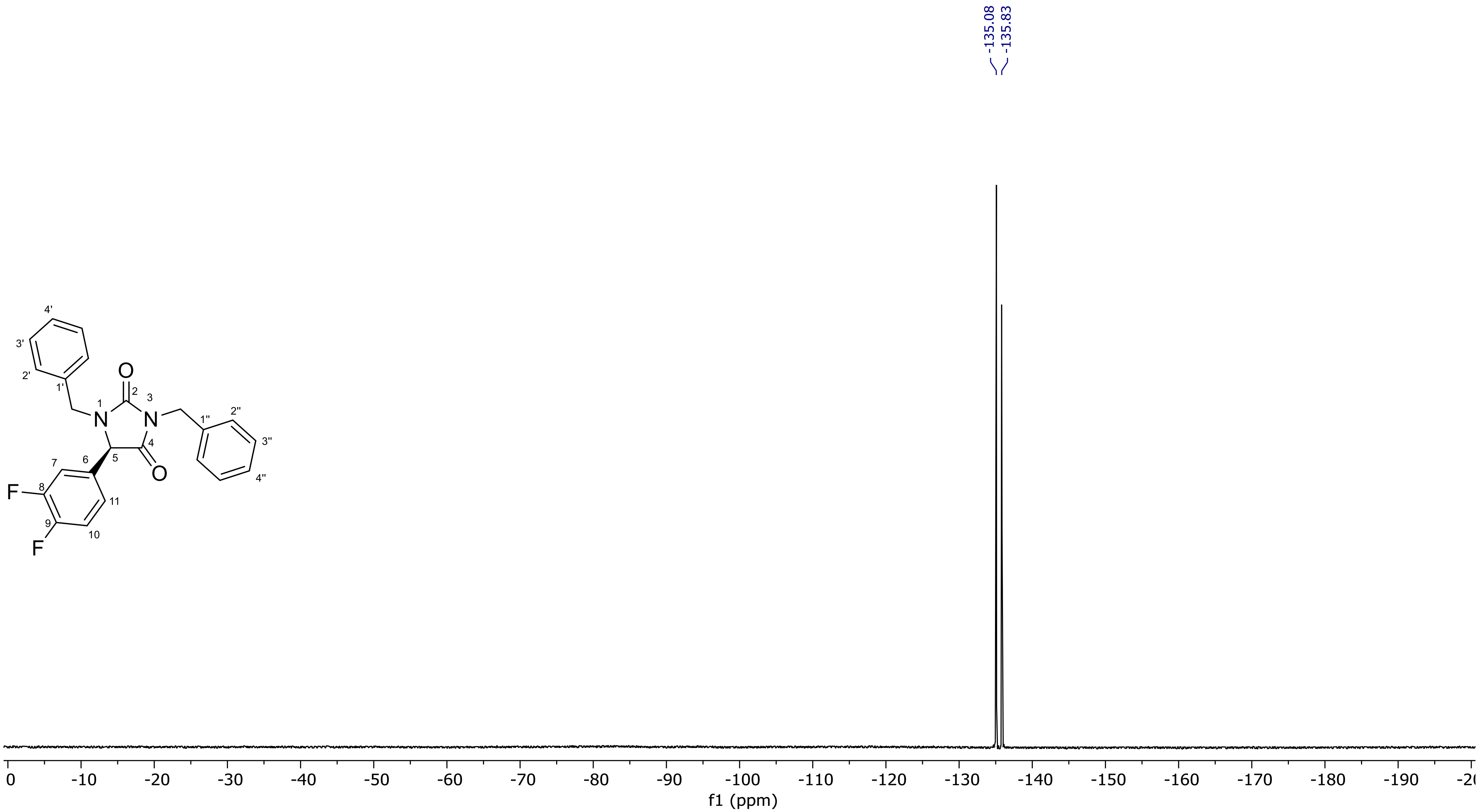

1,3-Dibenzyl-5-(2-methoxyphenyl)imidazolidine-2,4-dione 17o, ^{1}H NMR in CD_3CN (345 K)

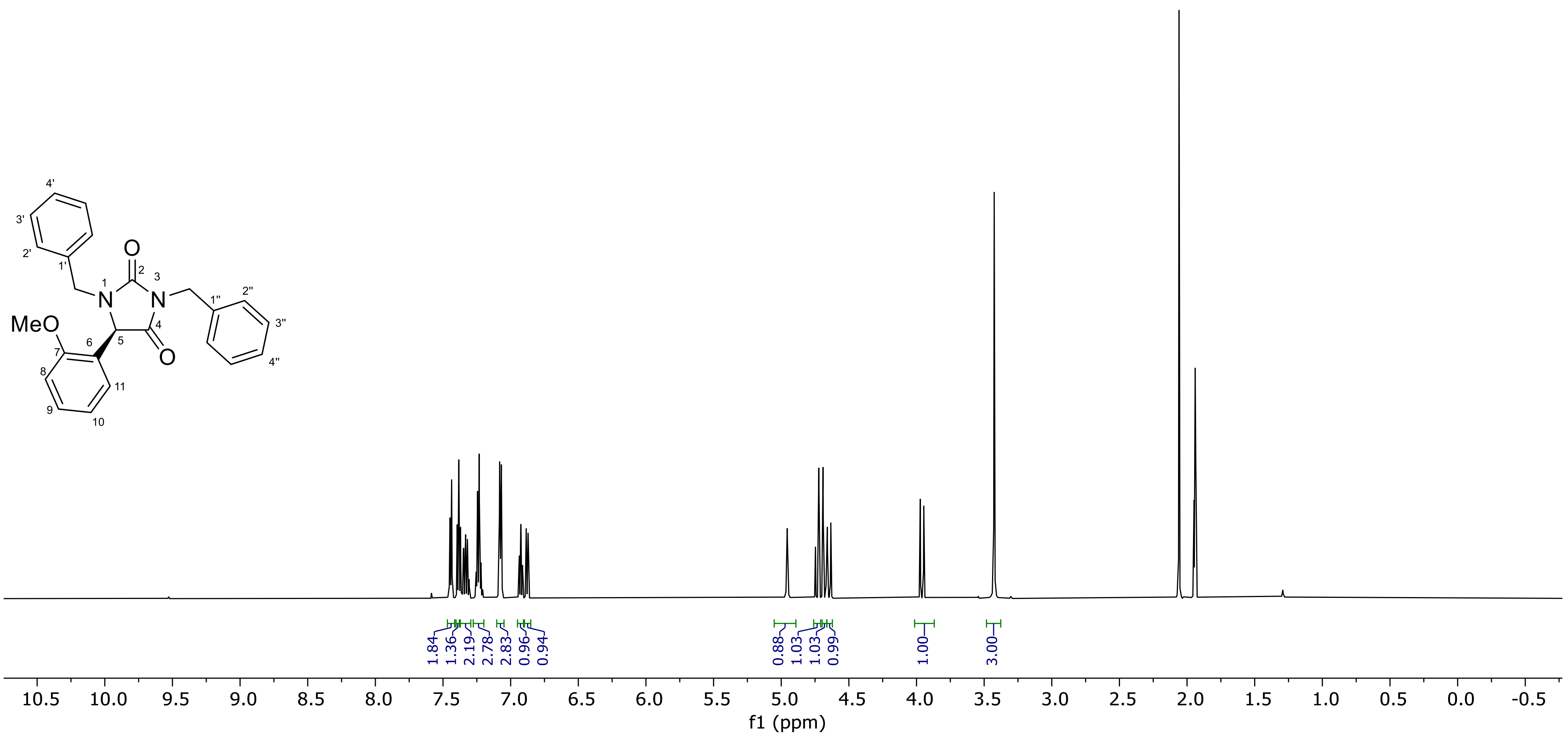

1,3-Dibenzyl-5-(2-methoxyphenyl)imidazolidine-2,4-dione 17o, ^{1}H NMR in CD_3CN (345 K)

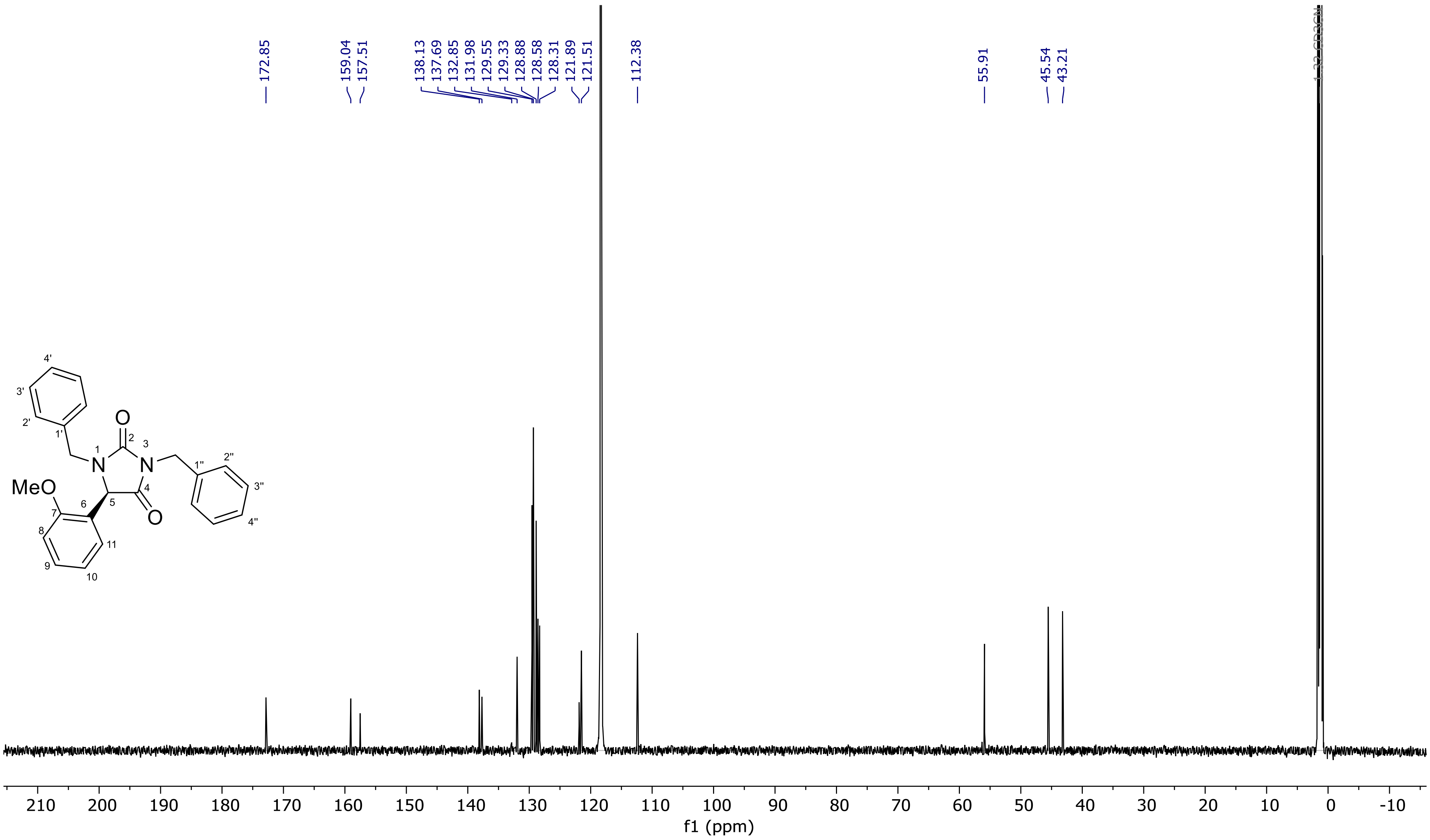

1,3-Dibenzyl-5-(2-methoxyphenyl)imidazolidine-2,4-dione 17p, ^{1}H NMR in CD_3CN (345 K)

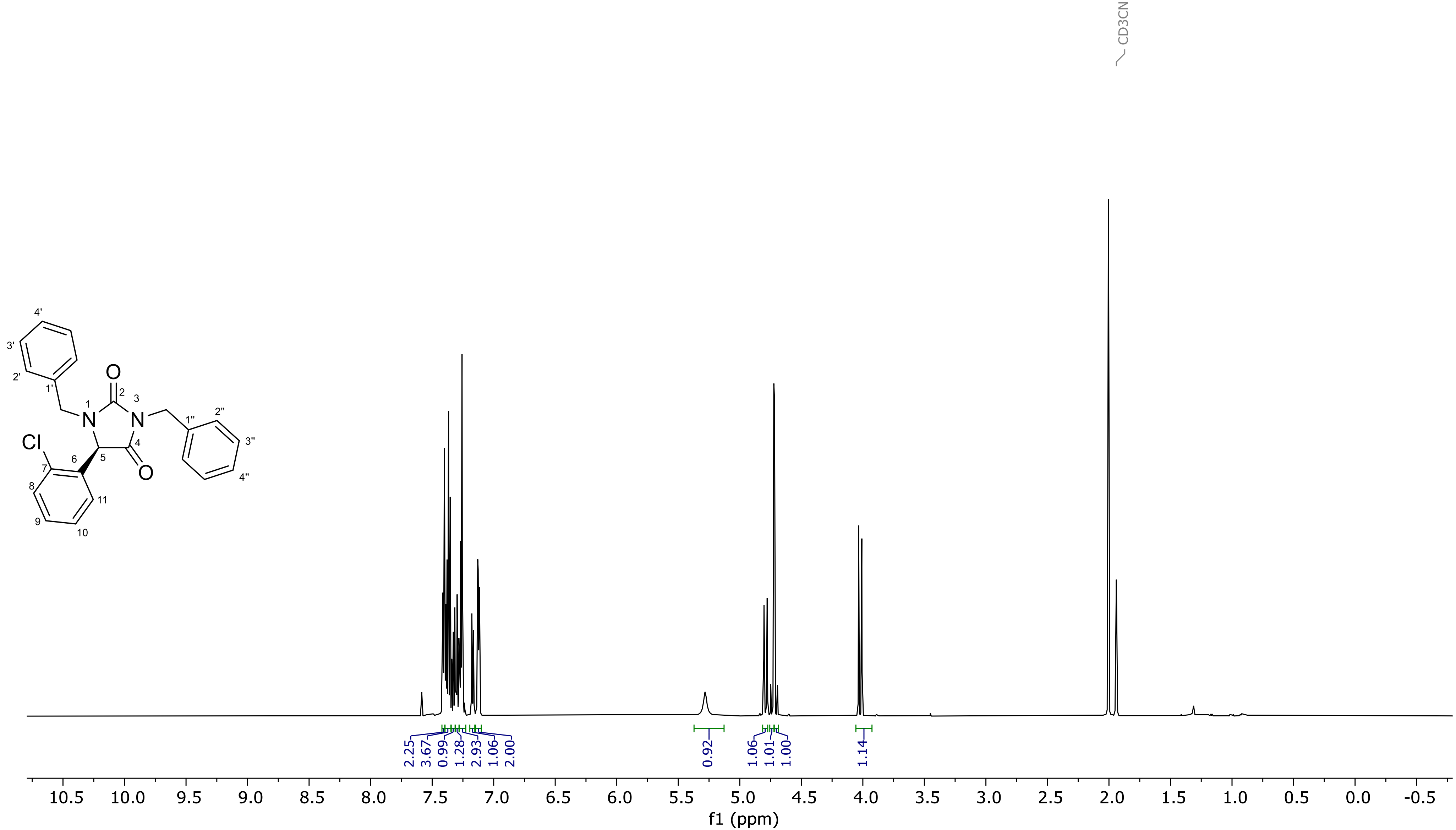

1,3-Dibenzyl-5-(2-chlorophenyl)imidazolidine-2,4-dione 17p, ^{13}C NMR in CD_3CN (345 K)

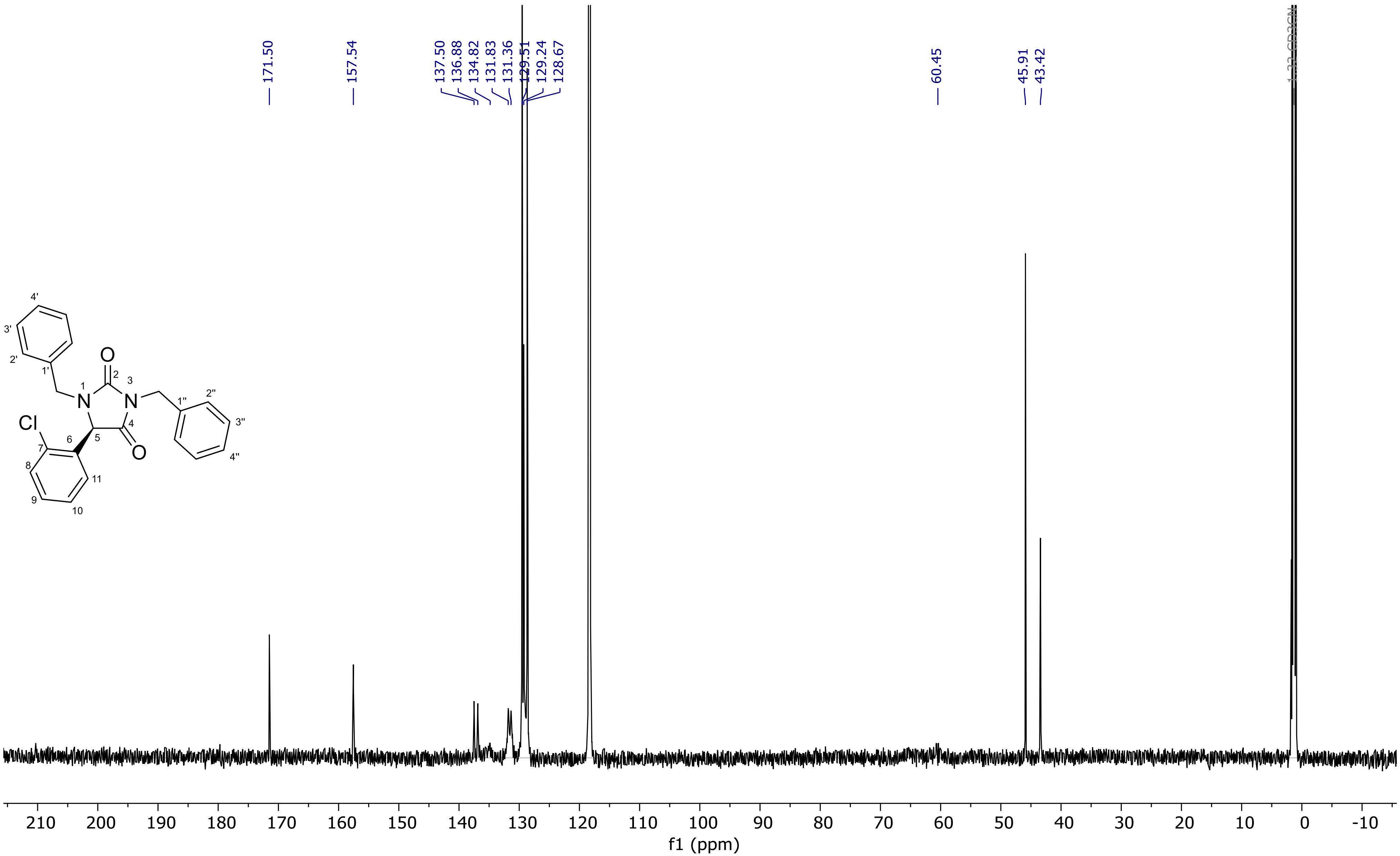

1,3-Dibenzyl-5-(2-(trifluoromethyl)phenyl)imidazolidine-2,4-dione 17q, ^{1}H NMR in CDCl₃

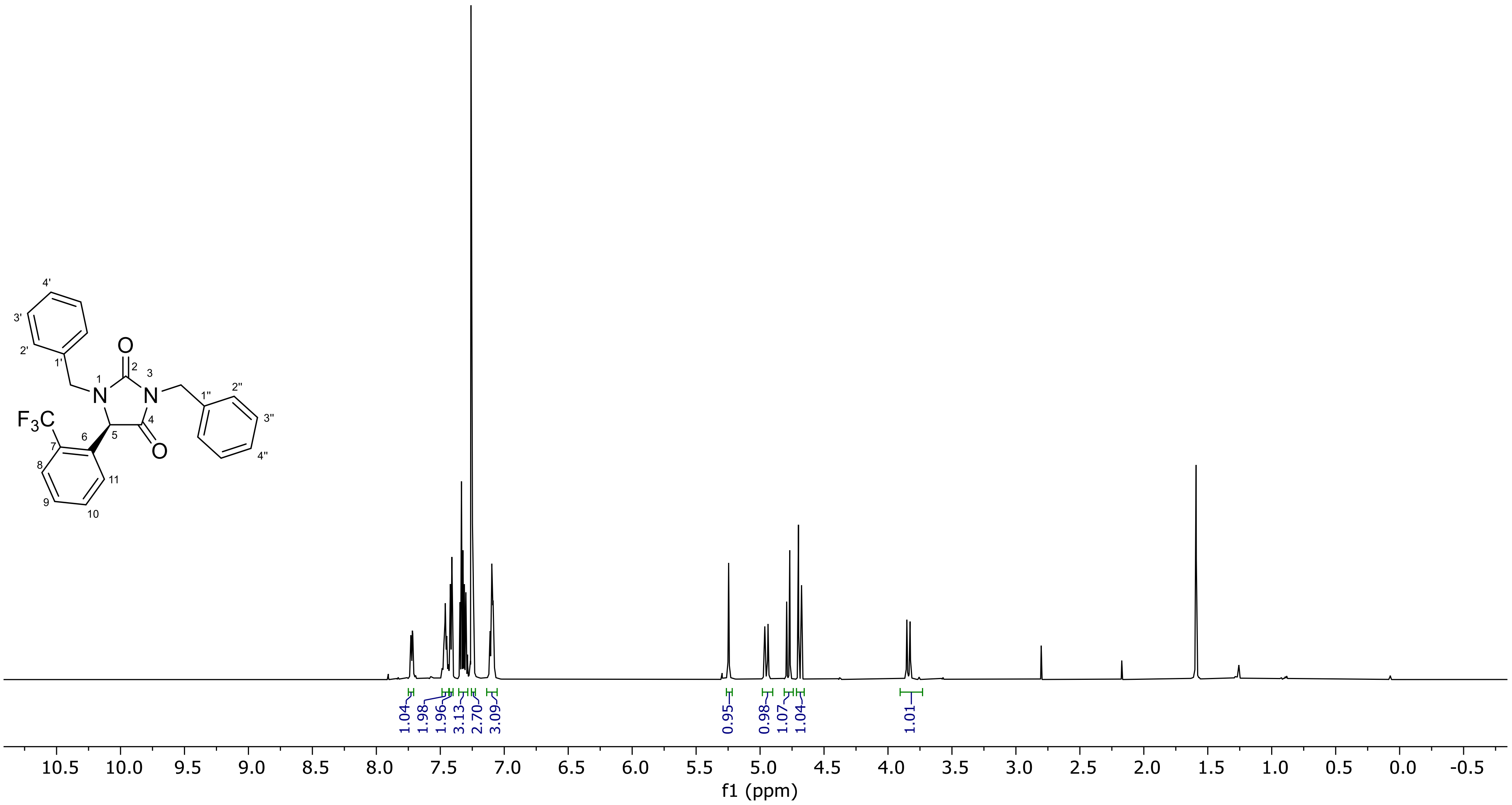

1,3-Dibenzyl-5-(2-(trifluoromethyl)phenyl)imidazolidine-2,4-dione 17q, ^{13}C NMR in $CDCl_3$

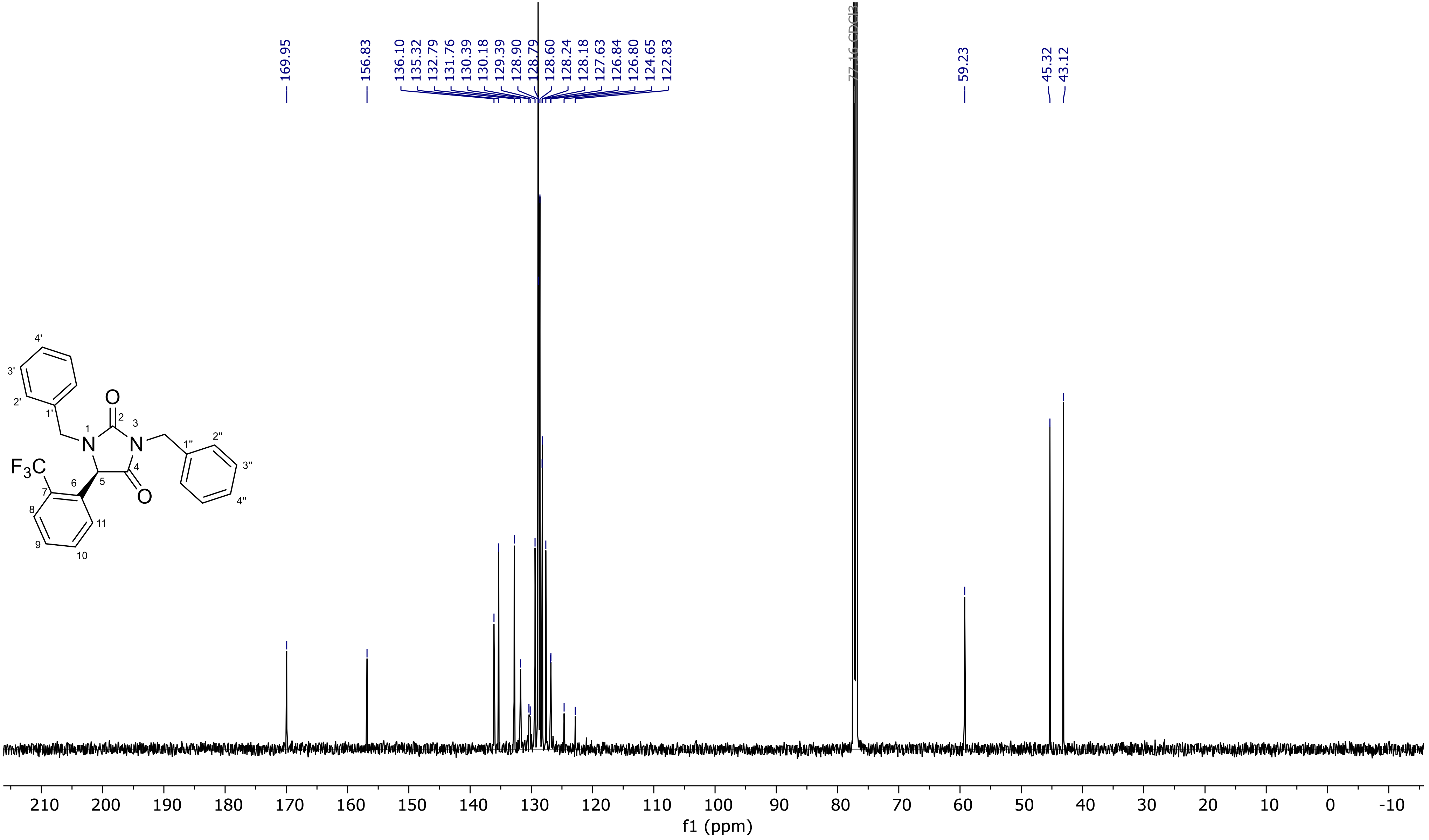

1,3-Dibenzyl-5-(2-(trifluoromethyl)phenyl)imidazolidine-2,4-dione 17q, ^{19}F NMR in $CDCl_3$

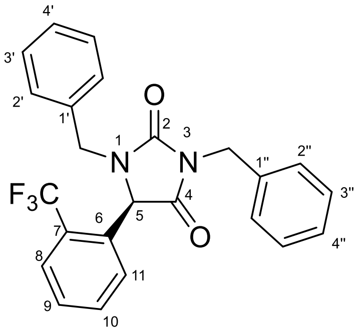

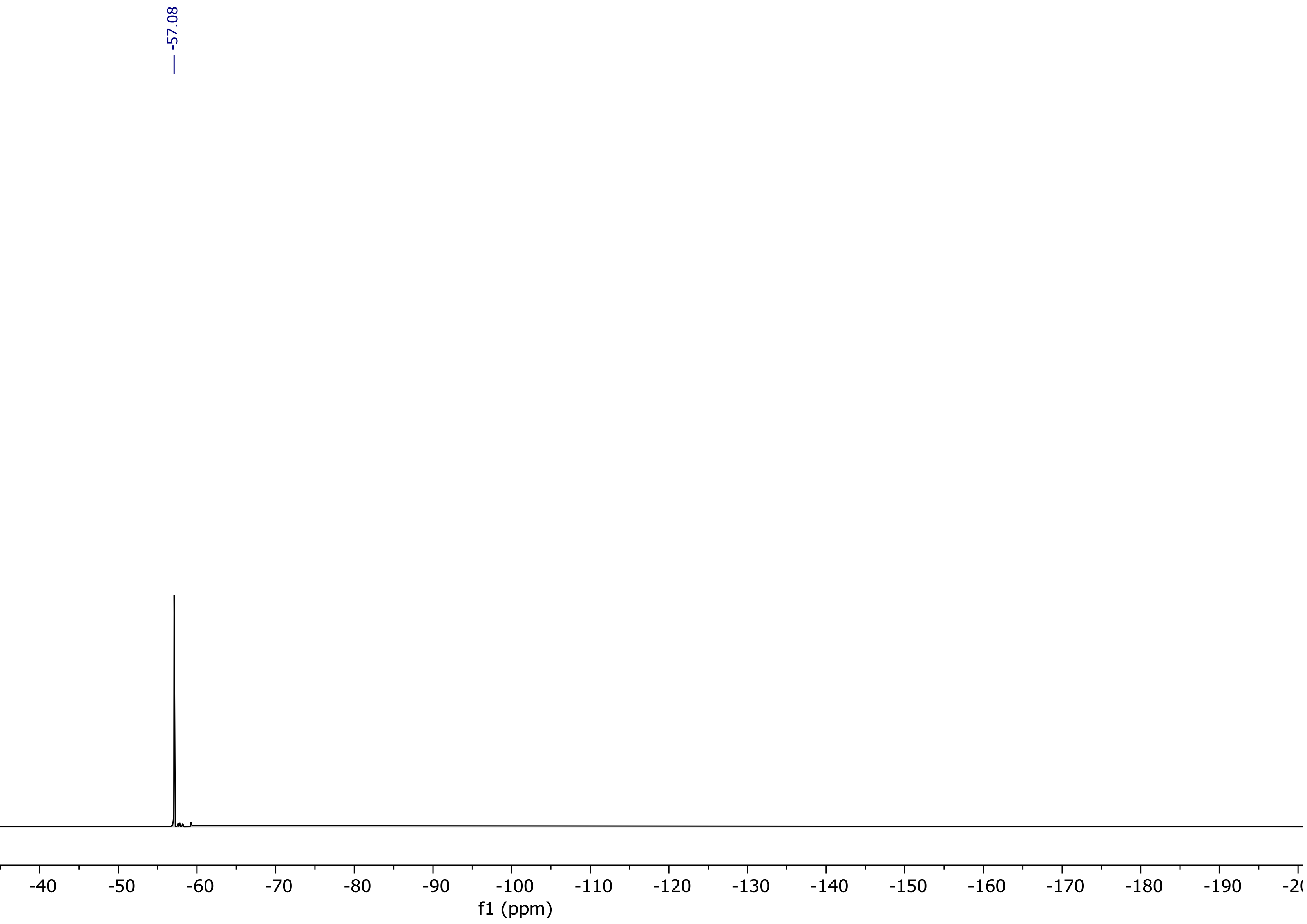

1,3-Dibenzyl-5-(naphthalen-1-yl)imidazolidine-2,4-dione 17r, ^{1}H NMR in $CDCl_3$ (223 K)

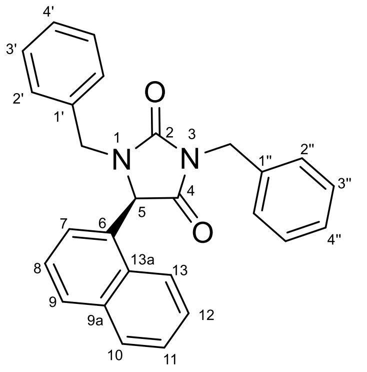

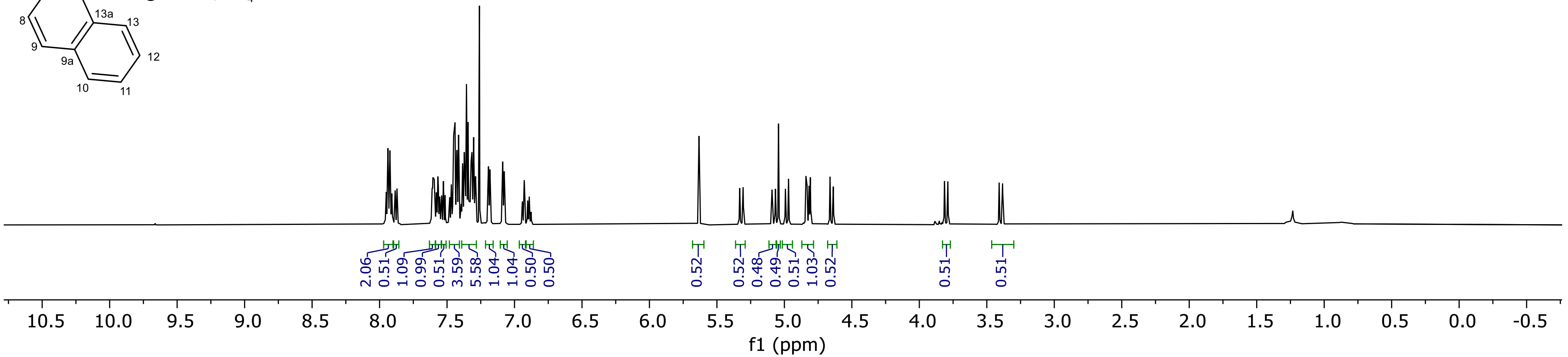

1,3-Dibenzyl-5-(naphthalen-1-yl)imidazolidine-2,4-dione 17r, ^{13}C NMR in CDCl$_3$

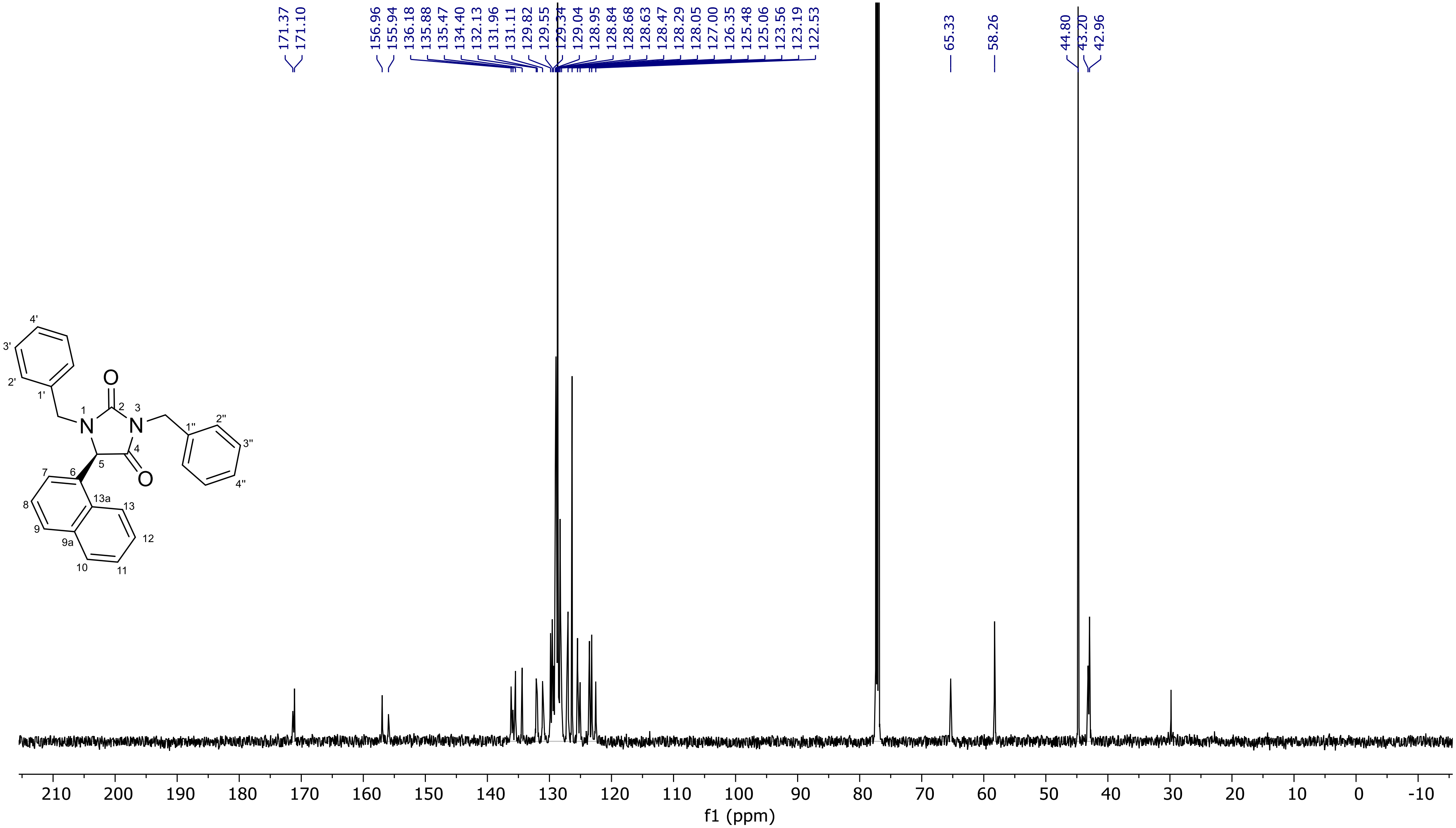

1,3-Dibenzyl-5-(naphthalen-2-yl)imidazolidine-2,4-dione 17s, ^{1}H NMR in CDCl₃

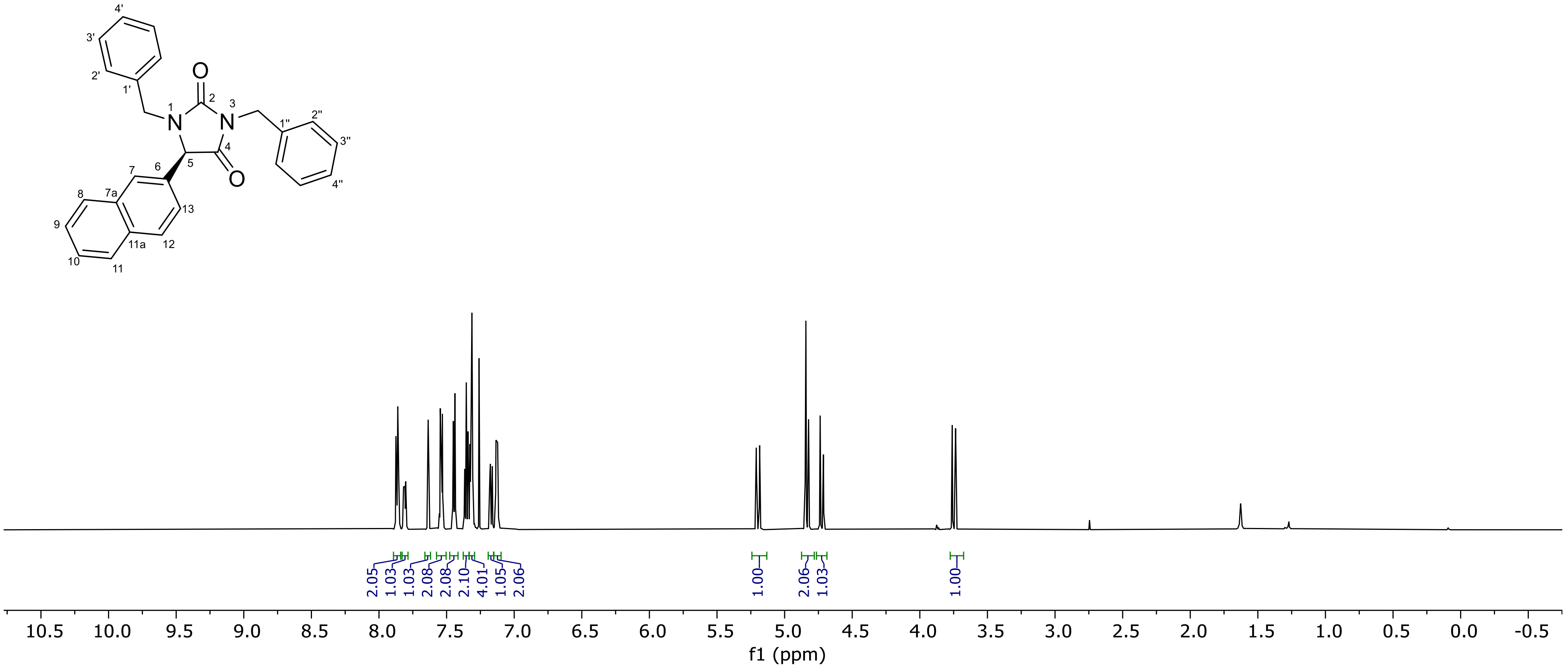

1,3-Dibenzyl-5-(naphthalen-2-yl)imidazolidine-2,4-dione 17s, ^{13}C NMR in $CDCl_3$

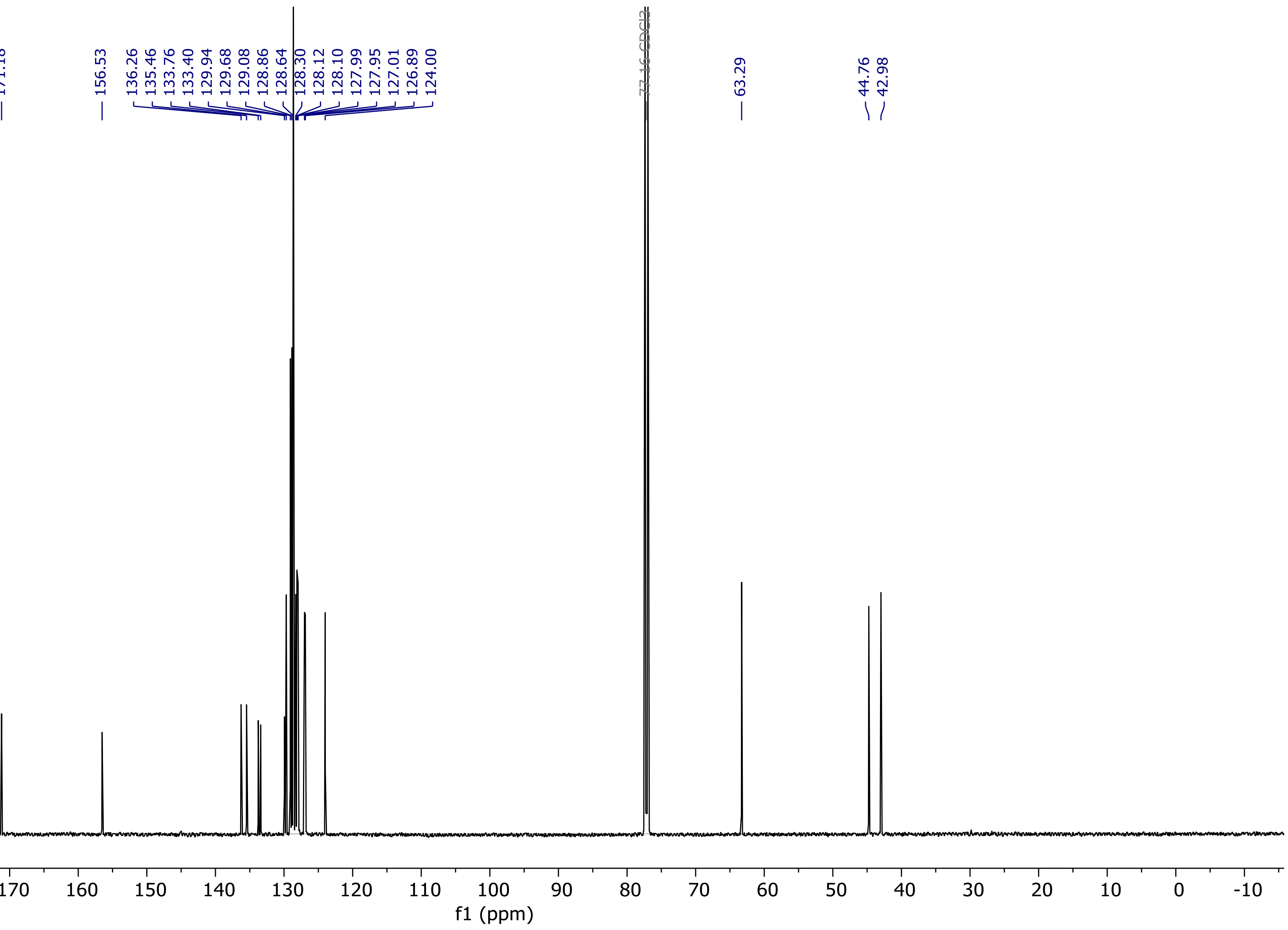

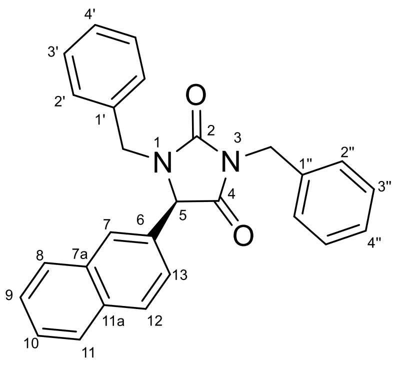

1,3-Dibenzyl-5-(5-bromothiophen-2-yl)imidazolidine-2,4-dione 17t, ^{1}H NMR in CDCl$_3$

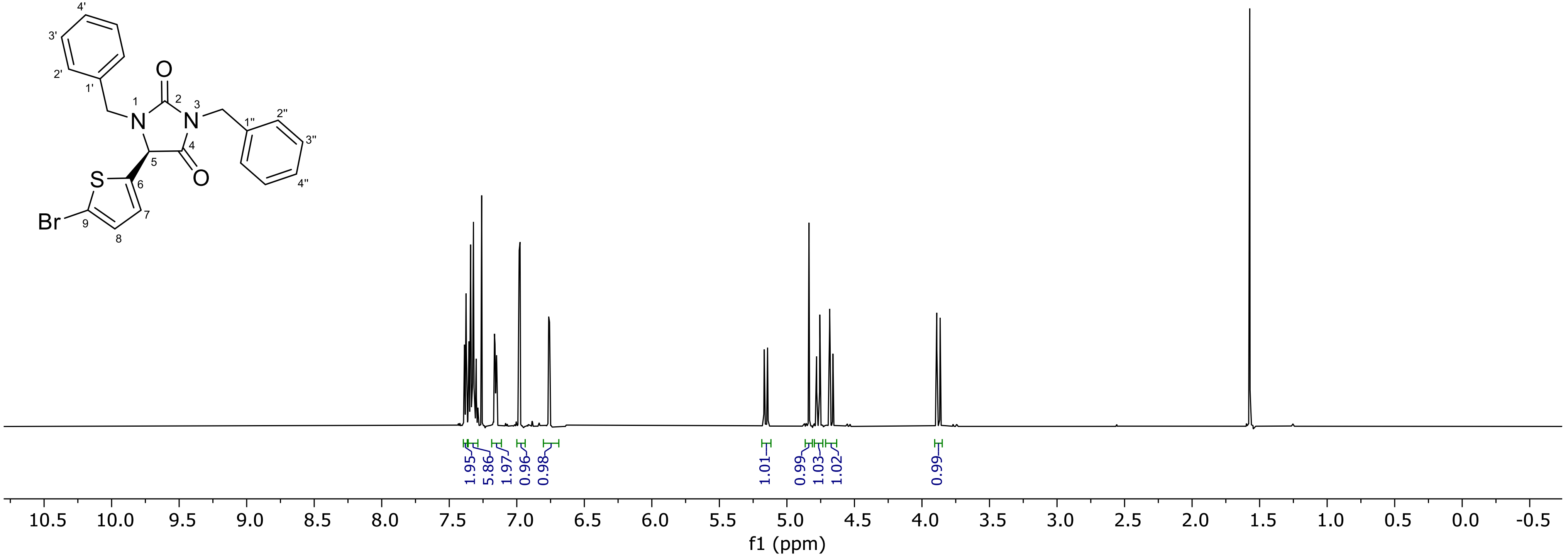

1,3-Dibenzyl-5-(5-bromothiophen-2-yl)imidazolidine-2,4-dione 17t, ^{13}C NMR in $CDCl_3$

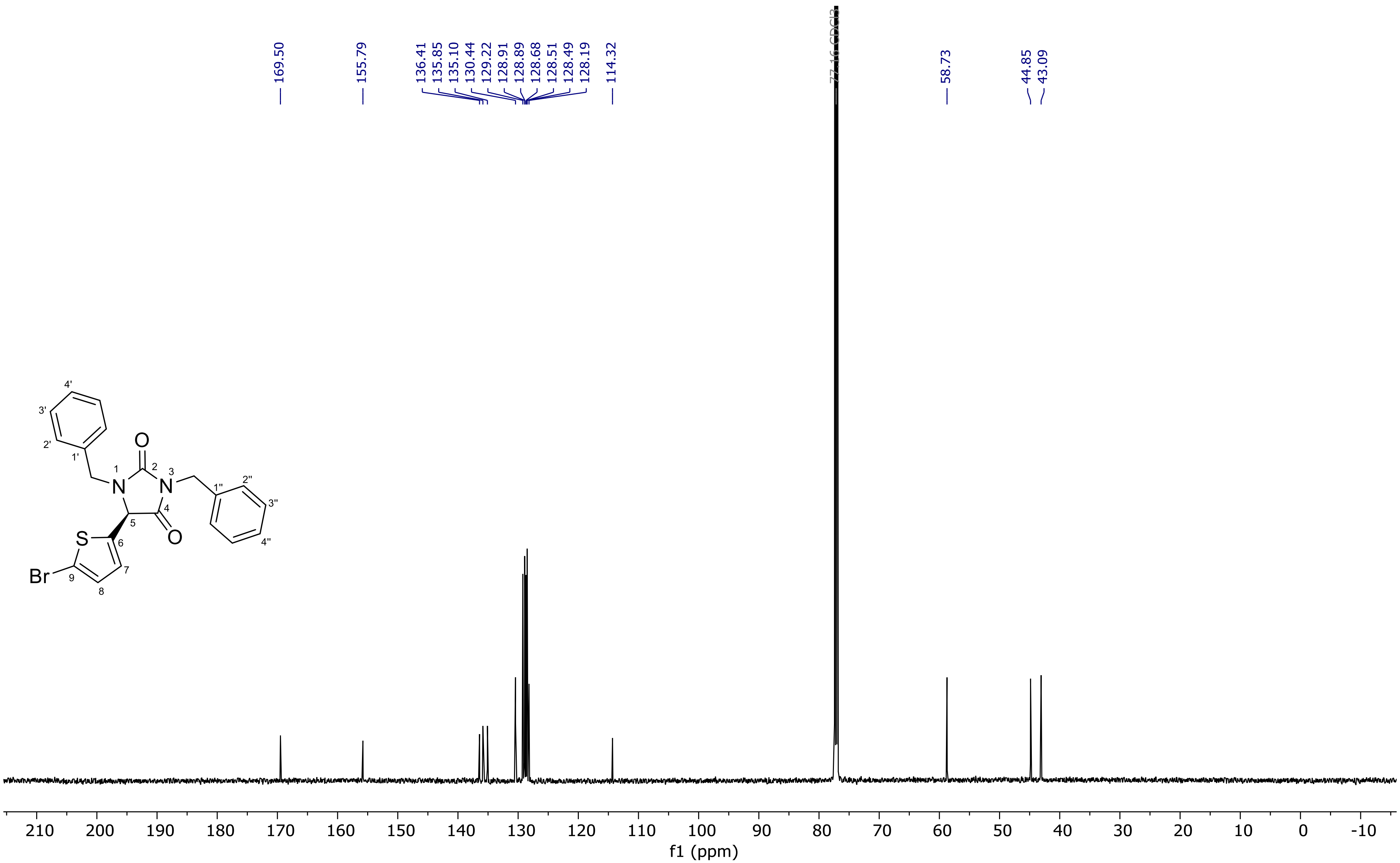

1,3-Dimethyl-5-phenylimidazolidine-2,4-dione 17z, ^{1}H NMR in $CDCl_3$

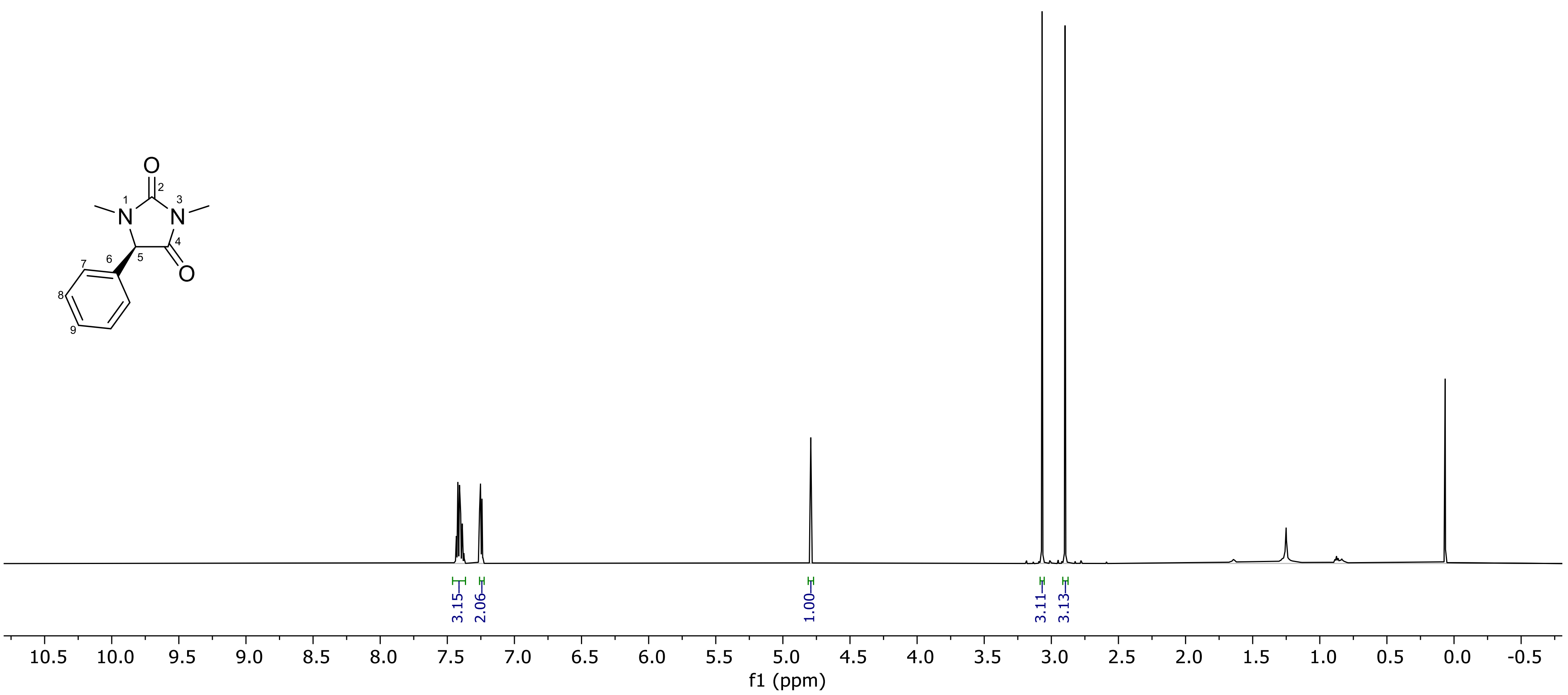

1,3-Dimethyl-5-phenylimidazolidine-2,4-dione 17z, ^{13}C NMR in CDCl$_3$

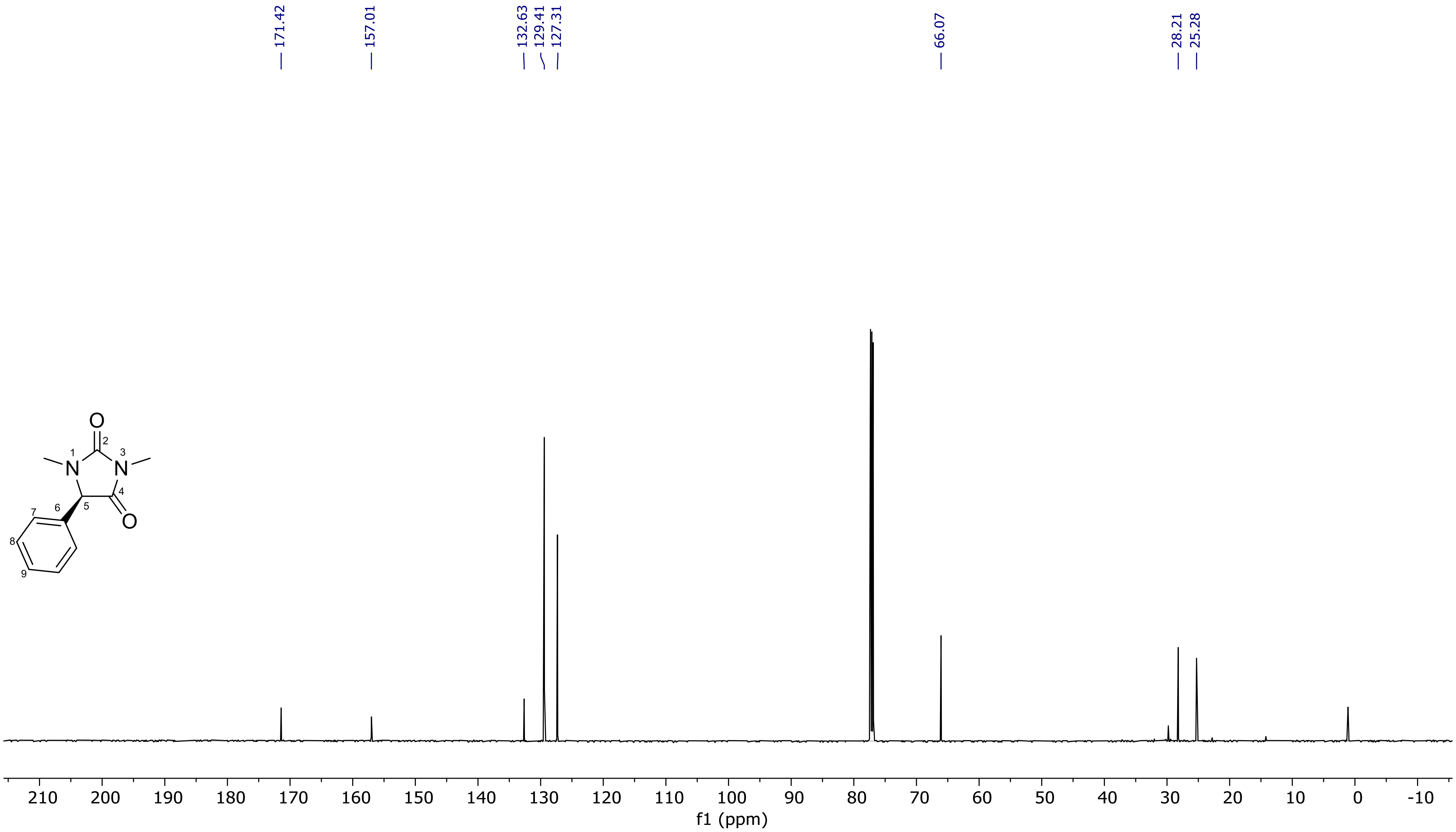

1,3-Bis[(2,4-dimethoxyphenyl)methyl]-5-phenylimidazolidine-2,4-dione 17v-a, ^{1}H NMR in CDCl₃

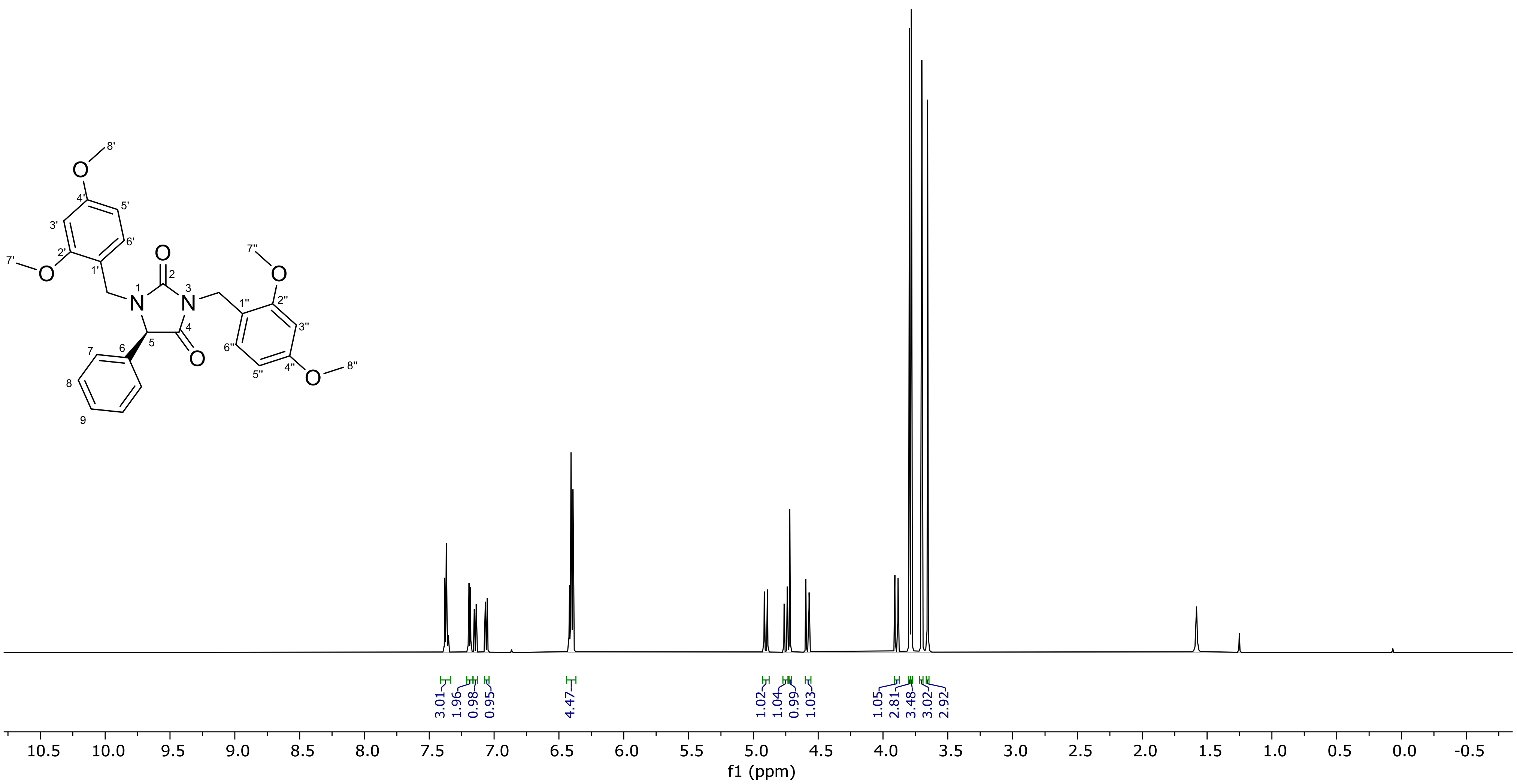

1,3-Bis[(2,4-dimethoxyphenyl)methyl]-5-phenylimidazolidine-2,4-dione 17v-a, ^{13}CNMR in CDCl$_3$

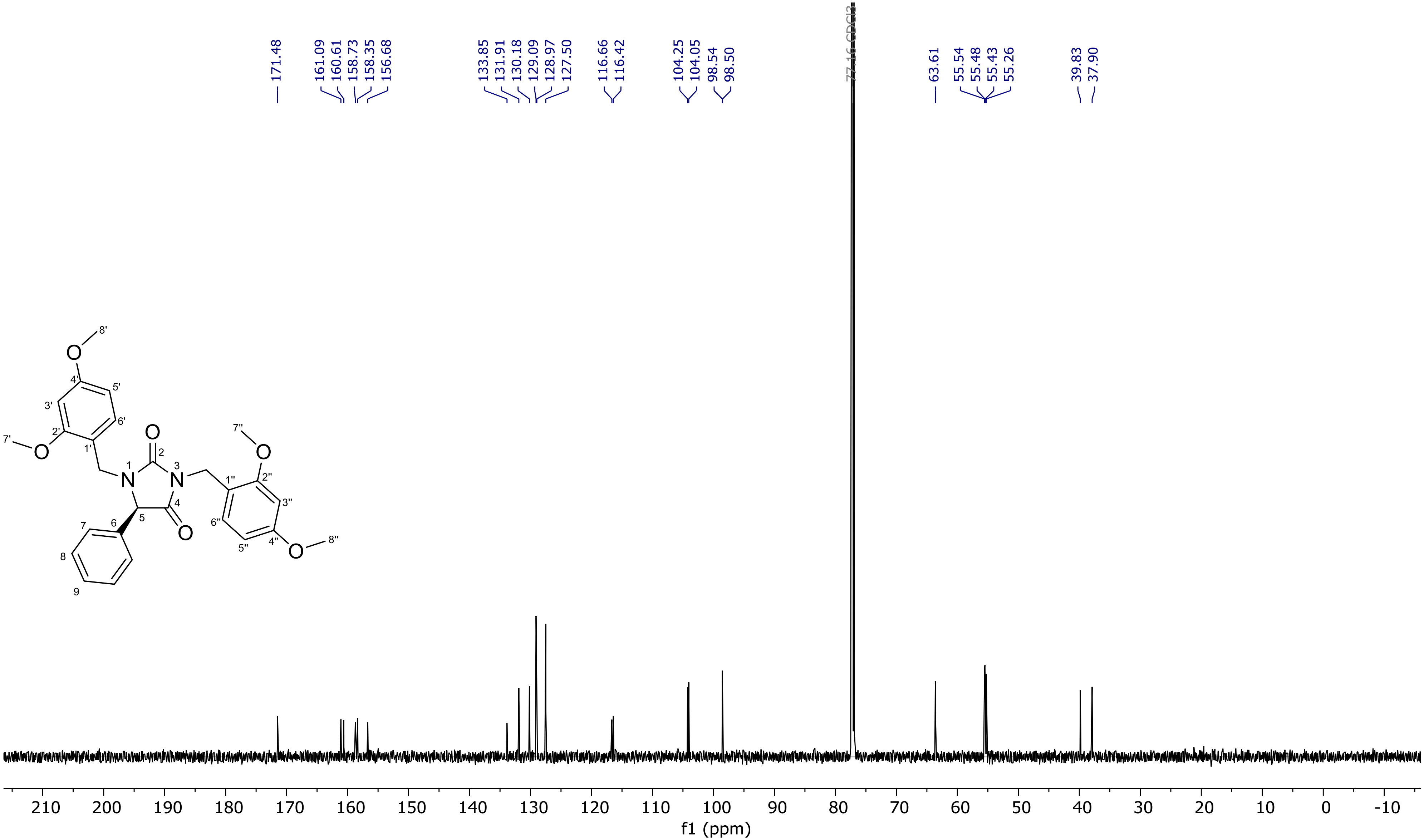

1,3-Bis[(2,4-dimethoxyphenyl)methyl]-5-(4-methylphenyl)imidazolidine-2,4-dione 17v-b, ^{1}H NMR in $CDCl_3$

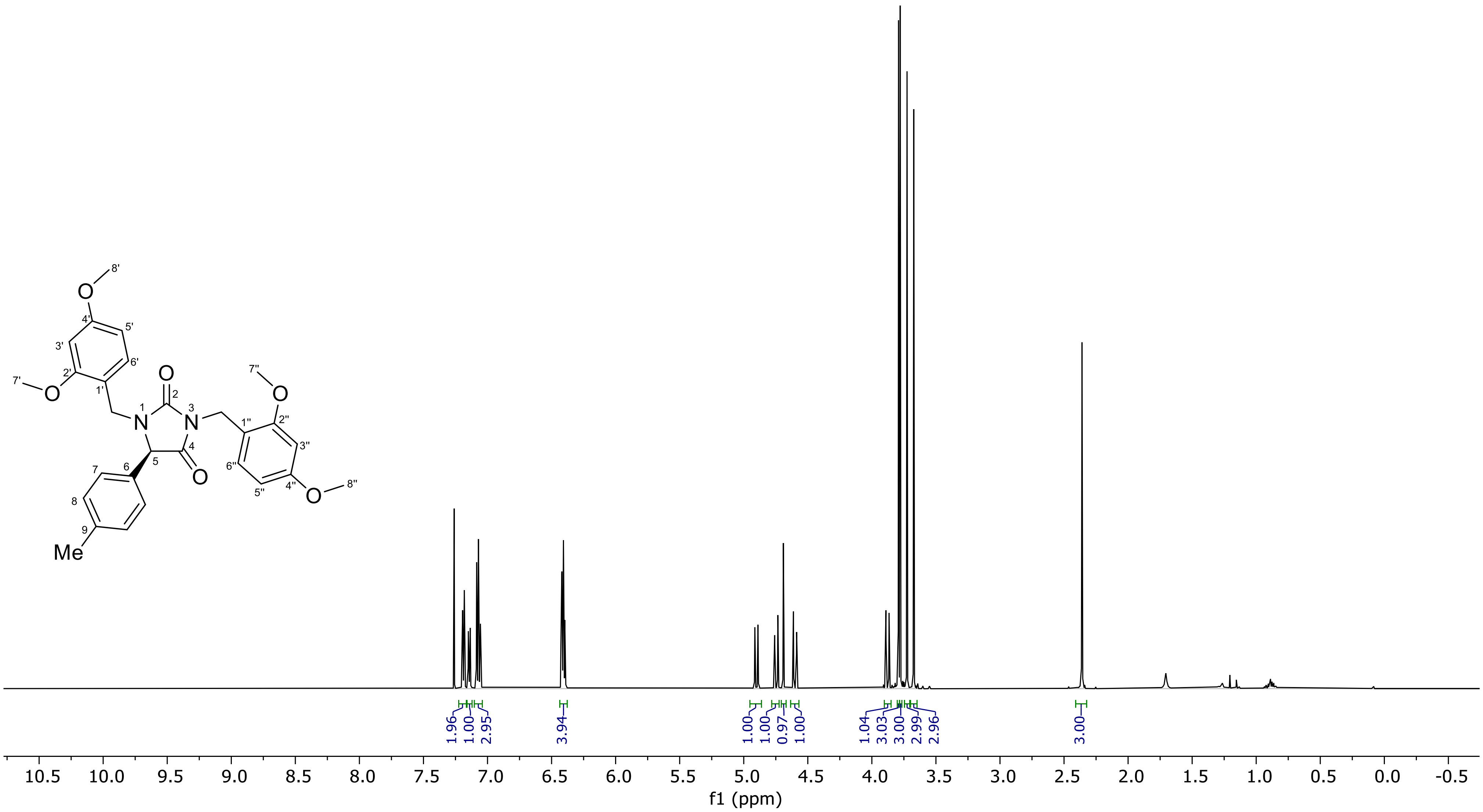

1,3-Bis[(2,4-dimethoxyphenyl)methyl]-5-(4-methylphenyl)imidazolidine-2,4-dione 17v-b, ^{13}C NMR in $CDCl_3$

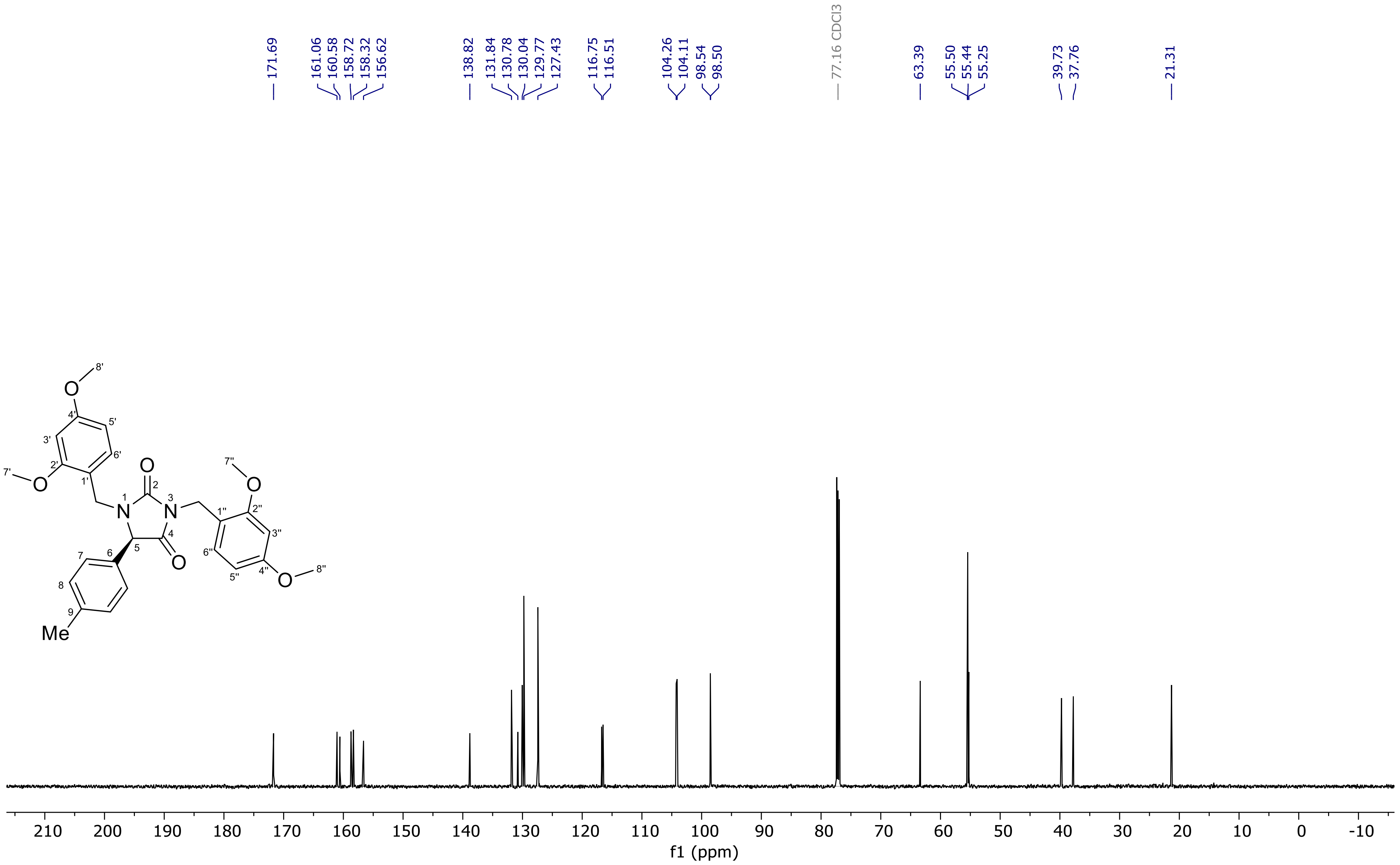

1,3-Bis[(2,4-dimethoxyphenyl)methyl]-5-(4-methoxyphenyl)imidazolidine-2,4-dione 17v-c, ^{1}H NMR in CDCl$_3$

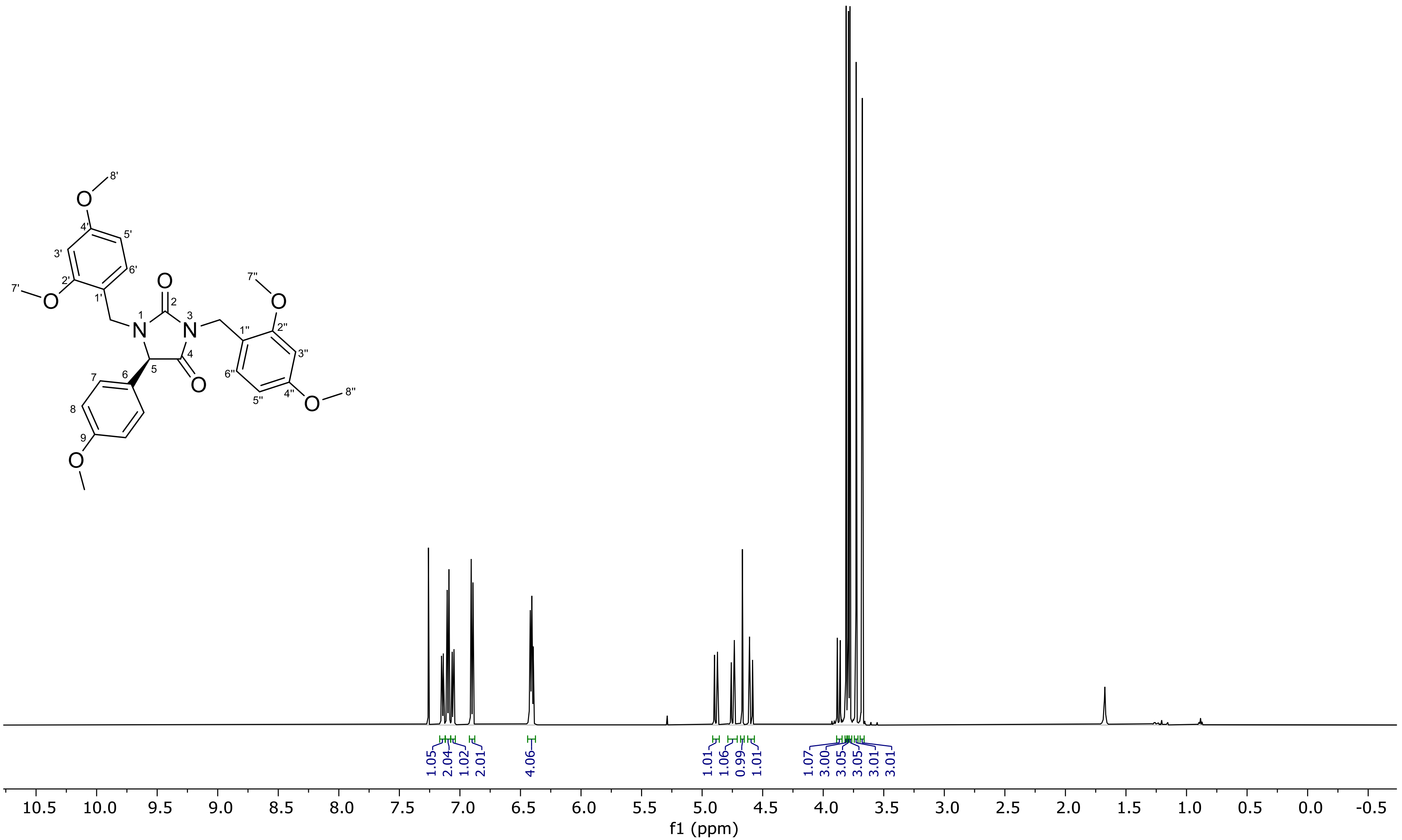

1,3-Bis[(2,4-dimethoxyphenyl)methyl]-5-(4-methoxyphenyl)imidazolidine-2,4-dione 17v-c, ^{13}CNMR in CDCl$_3$

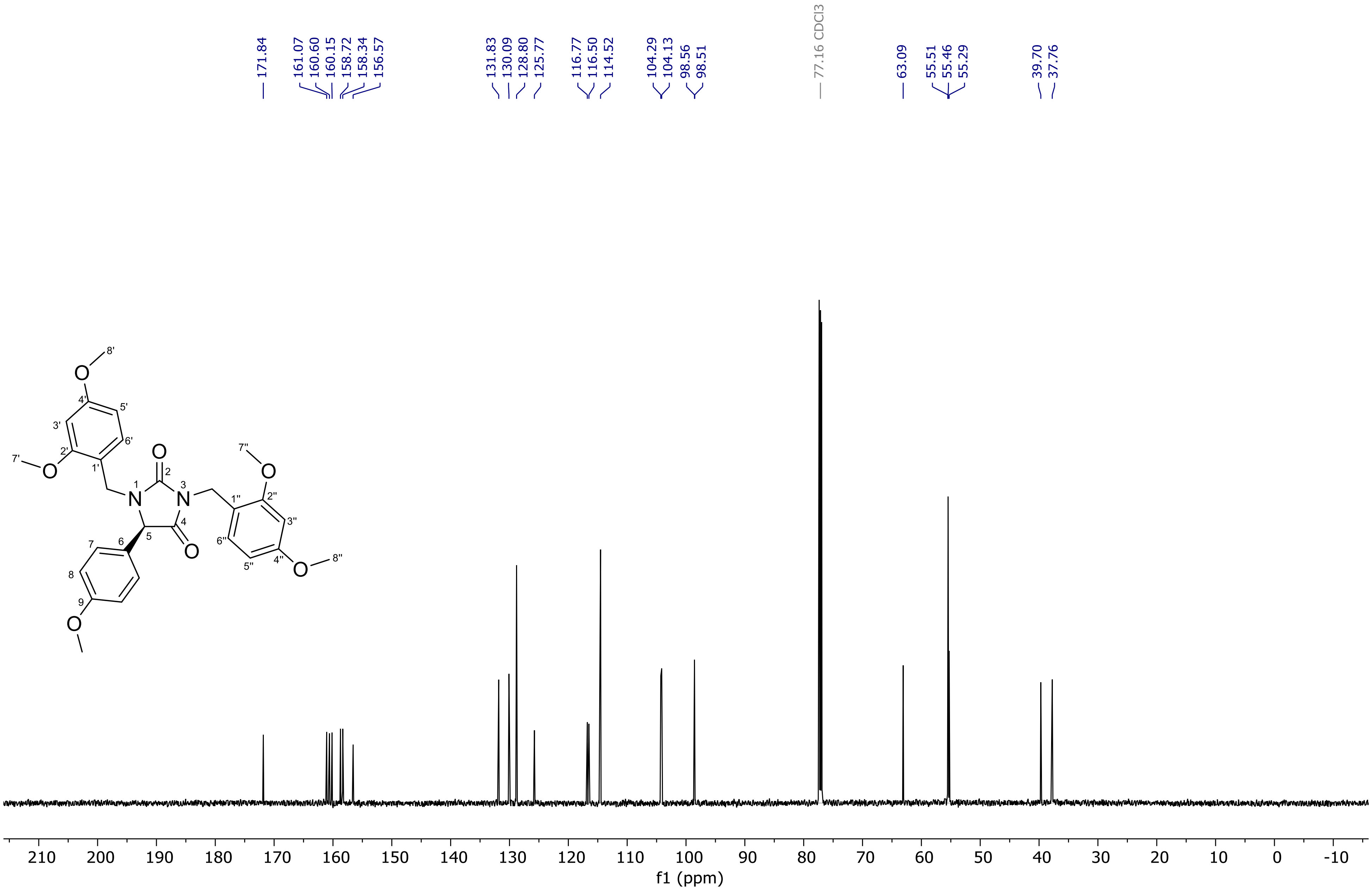

1,3-Bis[(2,4-dimethoxyphenyl)methyl]-5-(4-fluorophenyl)imidazolidine-2,4-dione, 17v-d, ^{1}H NMR in CDCl$_3$

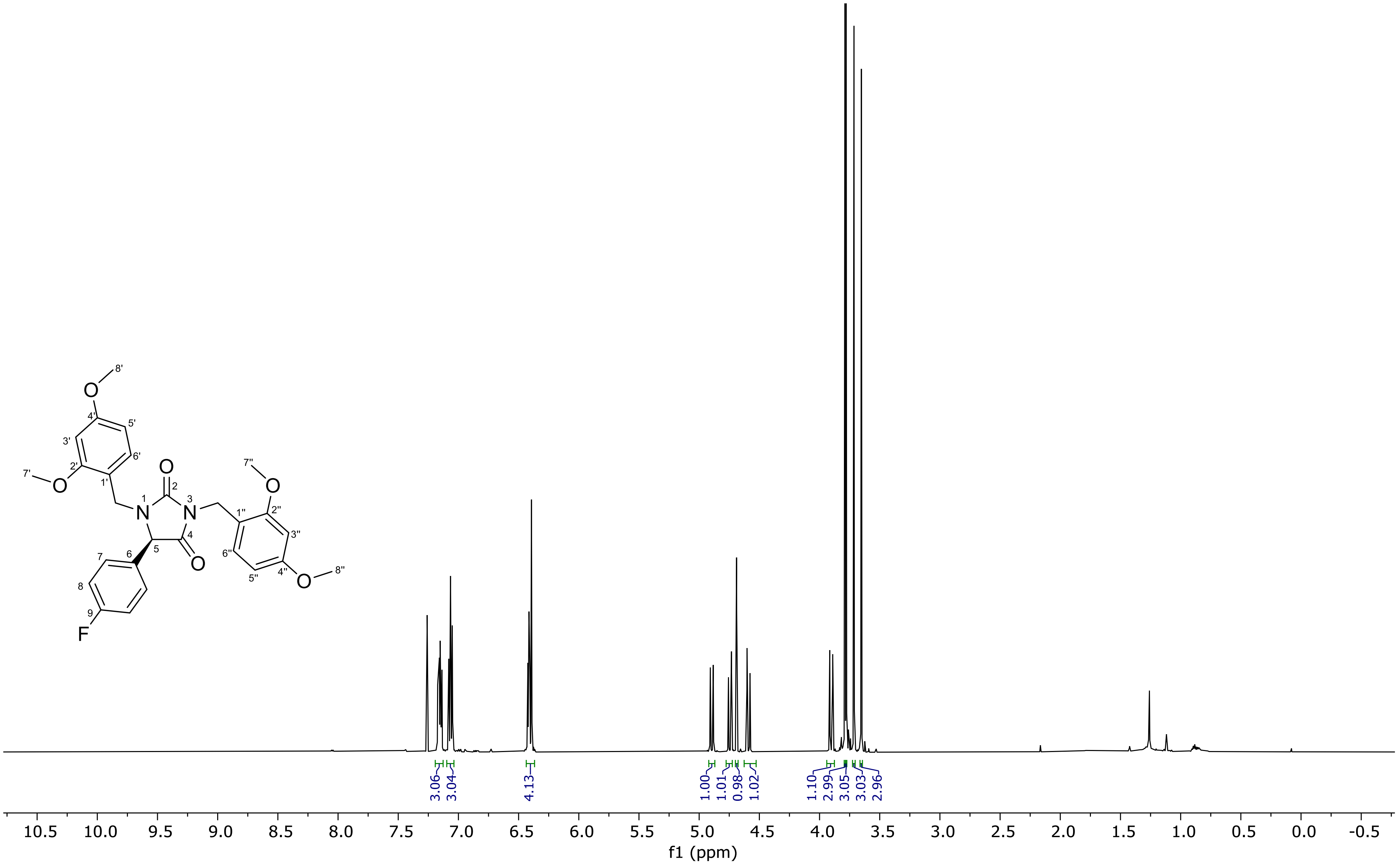

1,3-Bis[(2,4-dimethoxyphenyl)methyl]-5-(4-fluorophenyl)imidazolidine-2,4-dione, 17v-d, ^{13}H NMR in $CDCl_3$

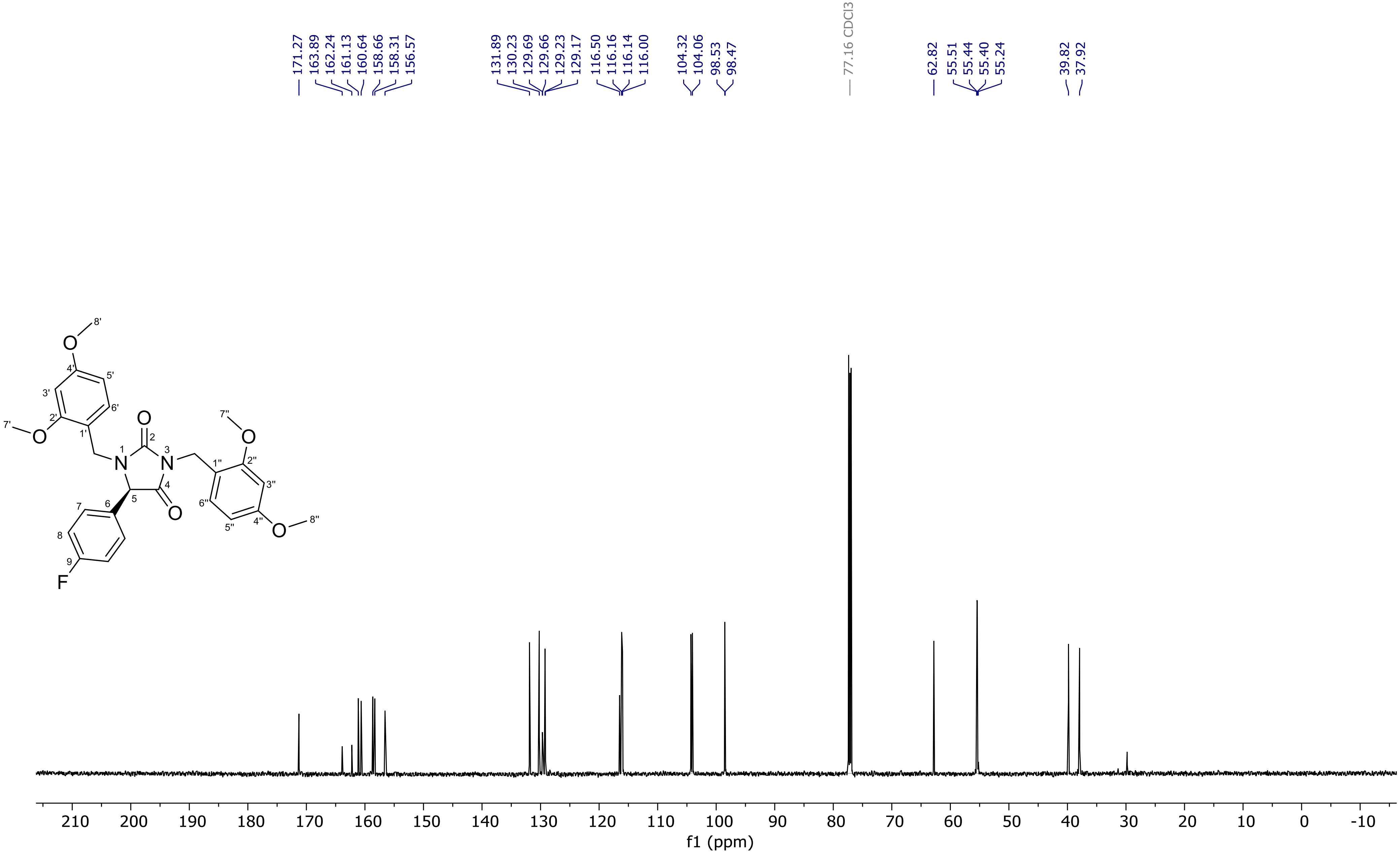

1,3-Bis[(2,4-dimethoxyphenyl)methyl]-5-(4-iodophenyl)imidazolidine-2,4-dione, 17v-e, ^{1}H NMR in $CDCl_3$

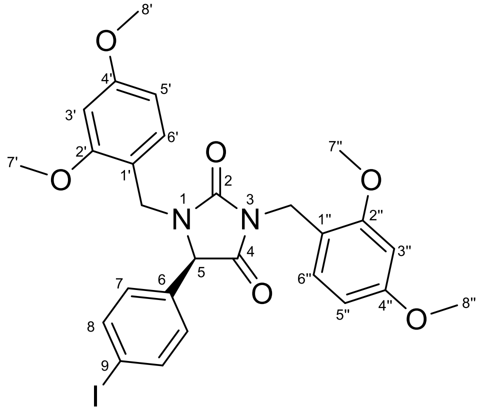

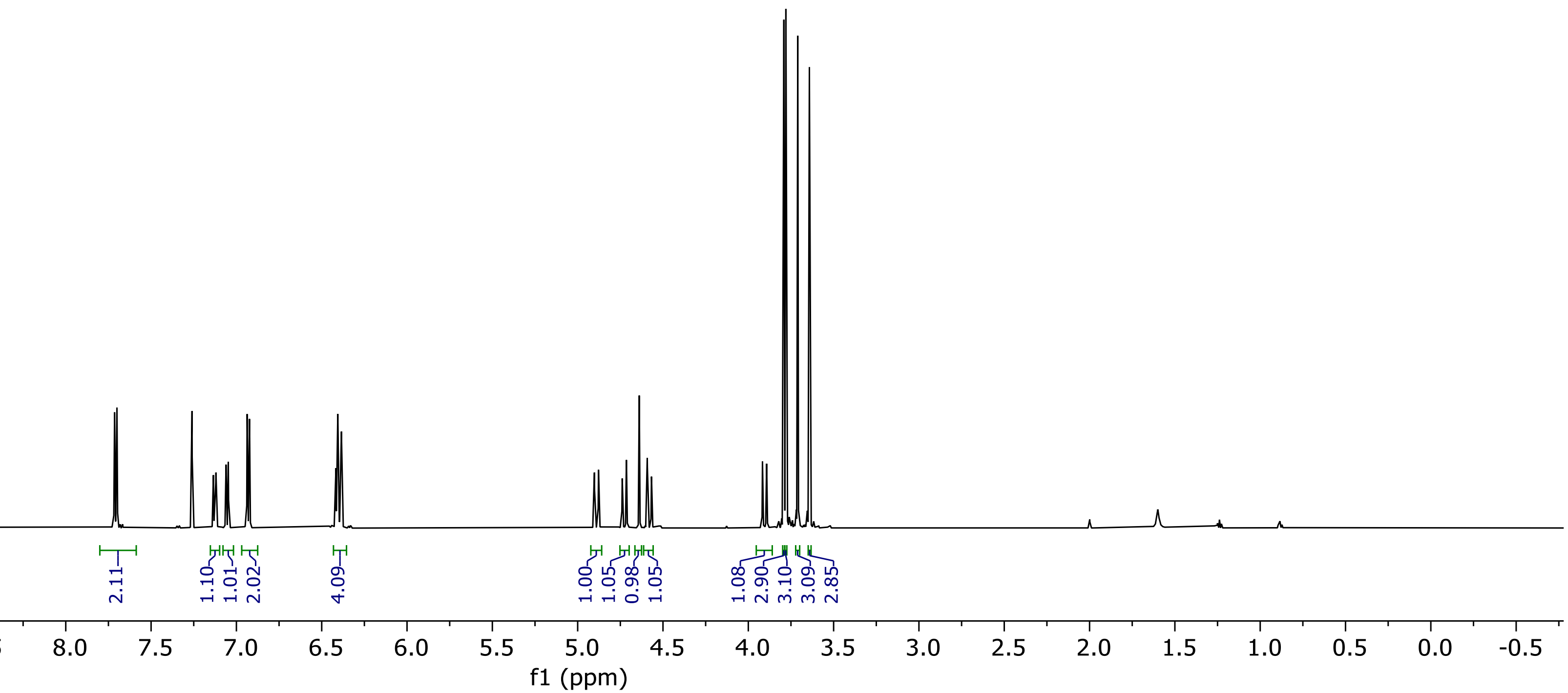

1,3-Bis[(2,4-dimethoxyphenyl)methyl]-5-(4-iodophenyl)imidazolidine-2,4-dione, 17v-e, ^{13}C NMR in $CDCl_3$

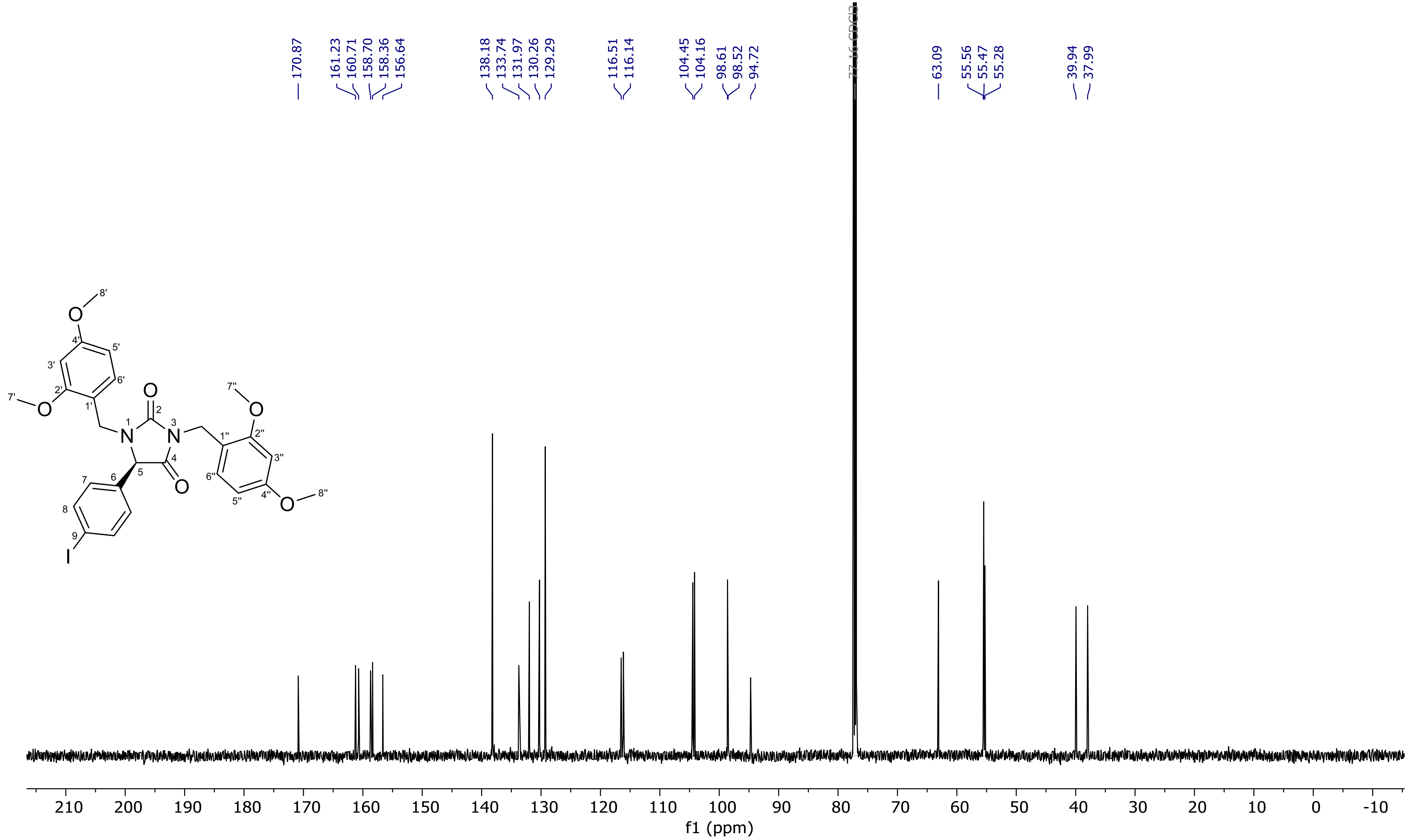

1-[(2,4-Dimethoxyphenyl)methyl]-5-phenylimidazolidine-2,4-dione 17w, ^{1}H NMR in CDCl$_3$

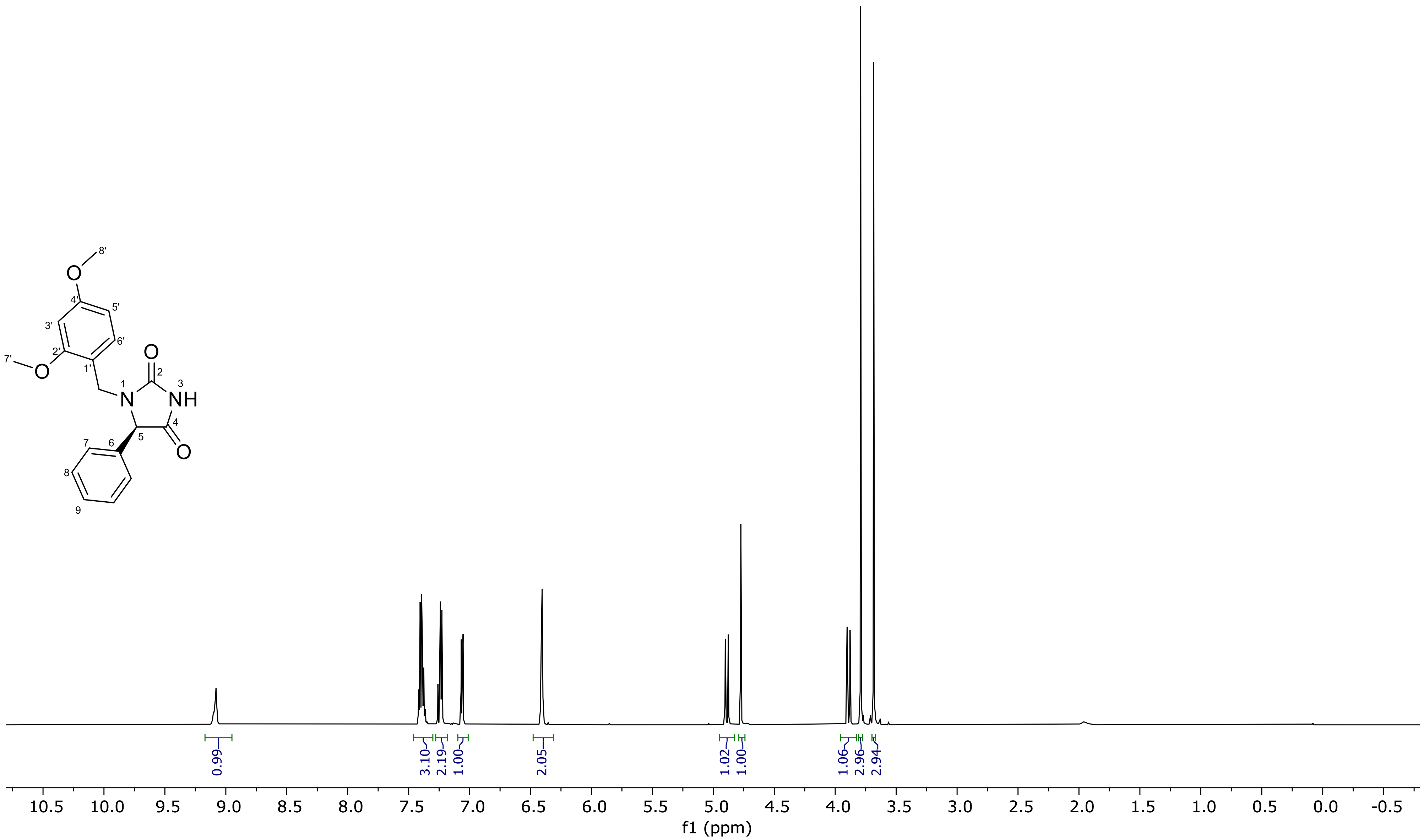

1-[(2,4-Dimethoxyphenyl)methyl]-5-phenylimidazolidine-2,4-dione 17w, ^{13}C NMR in $CDCl_3$

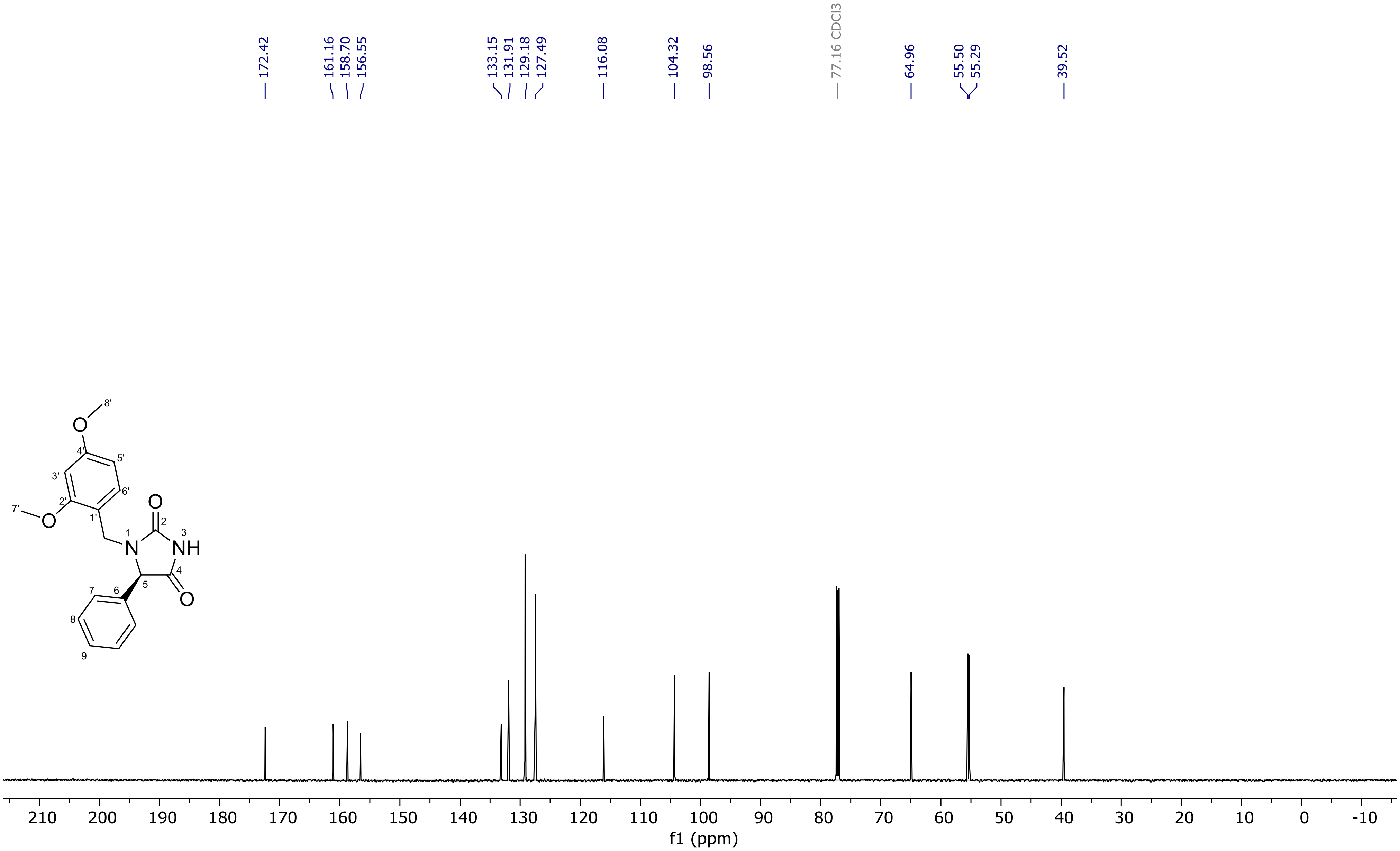

1-[(2,4-Dimethoxyphenyl)methyl]-5-phenylimidazolidine-2,4-dione 17w, HMBC correlations in $CDCl_3$

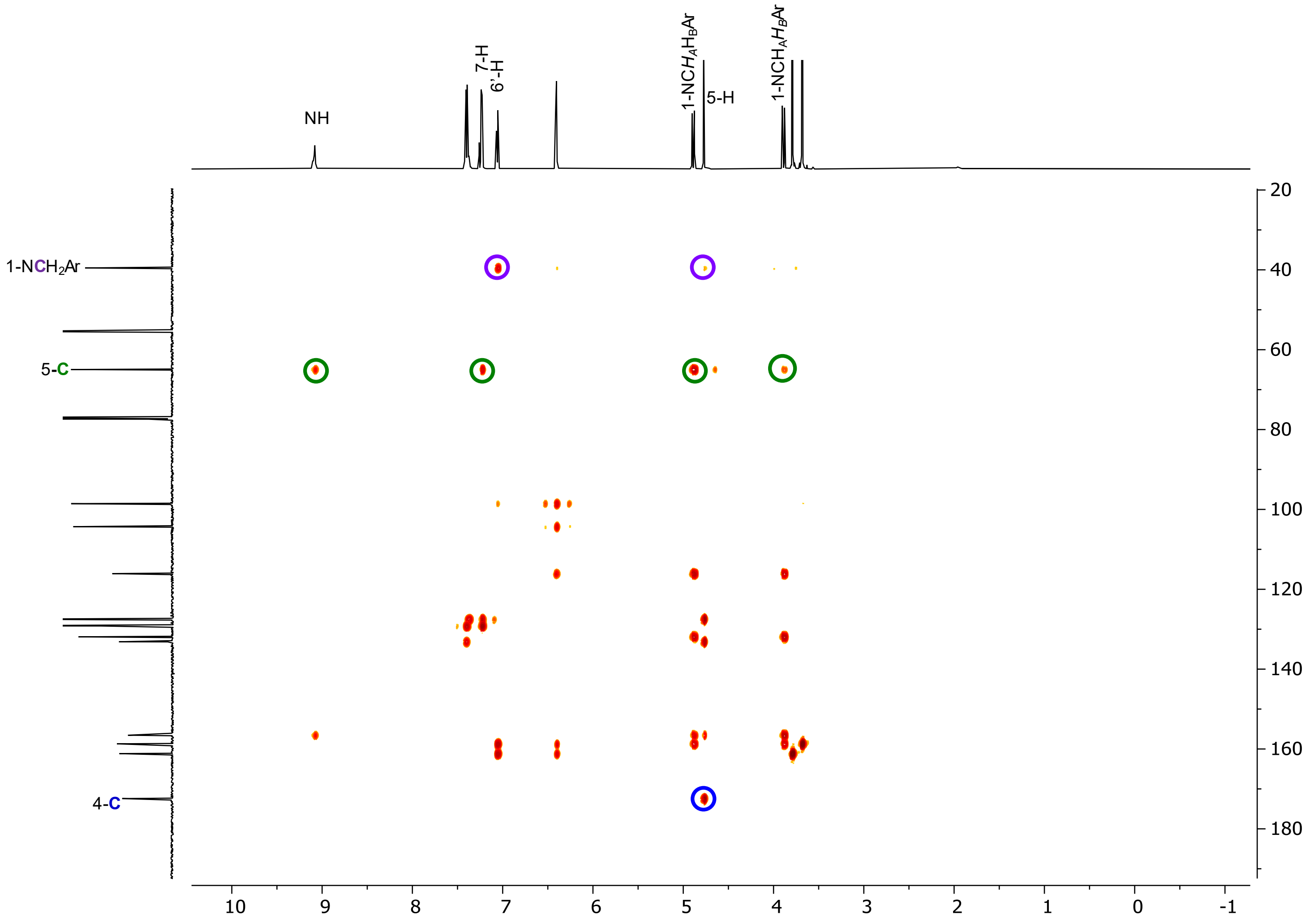

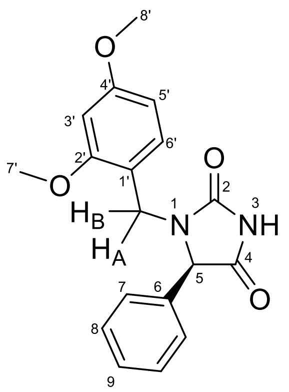

HMBC key correlations:

→ 1-N**C**H$_2$Ar: 5-H (^3J), 6'-H (^3J)

→ 5-**C**: 1-NCH$_A$H$_B$Ar (^3J), 7-H (^3J), NH (^3J)

→ 4-**C**: 5-H (^2J)

1,3-Dimethyl-5-phenyl-2-sulfanylideneimidazolidin-4-one 17x, ^{1}H NMR in $CDCl_3$

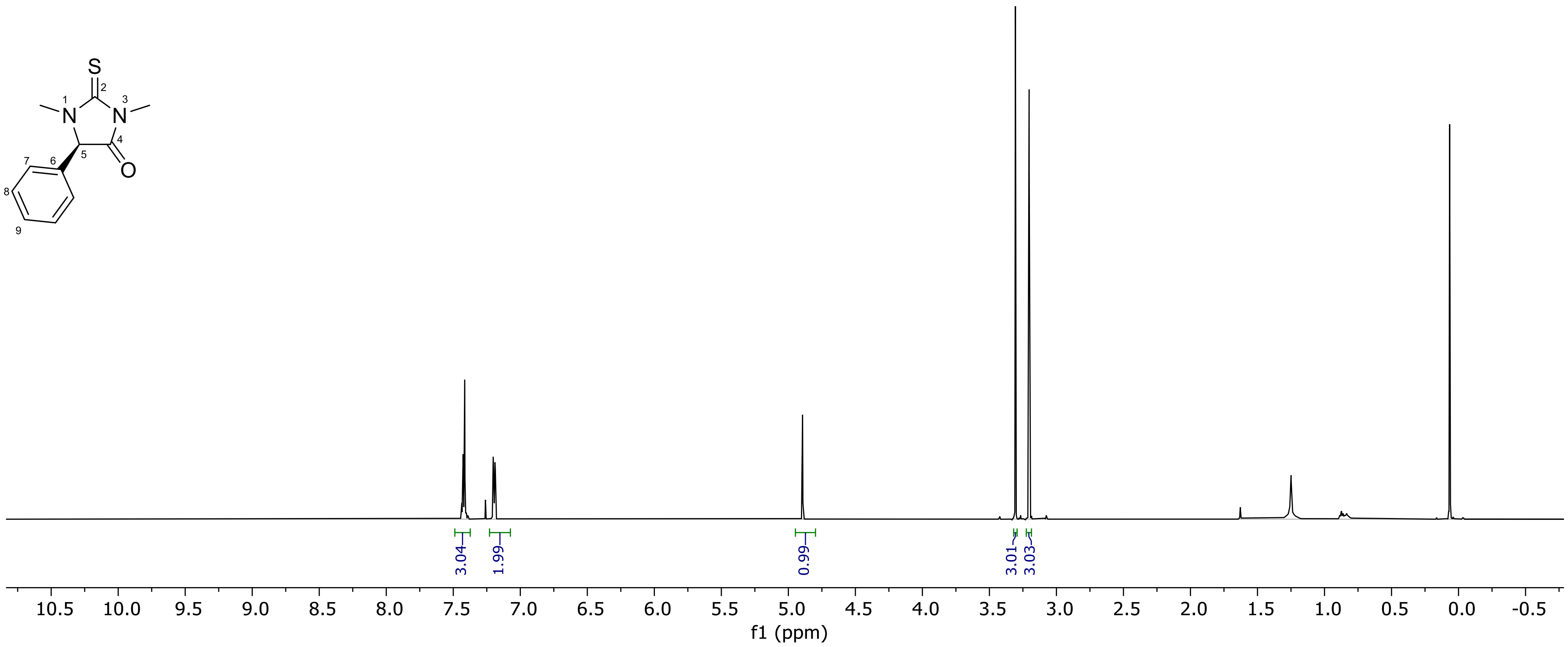

1,3-Dimethyl-5-phenyl-2-sulfanylideneimidazolidin-4-one 17x, ^{13}C NMR in $CDCl_3$

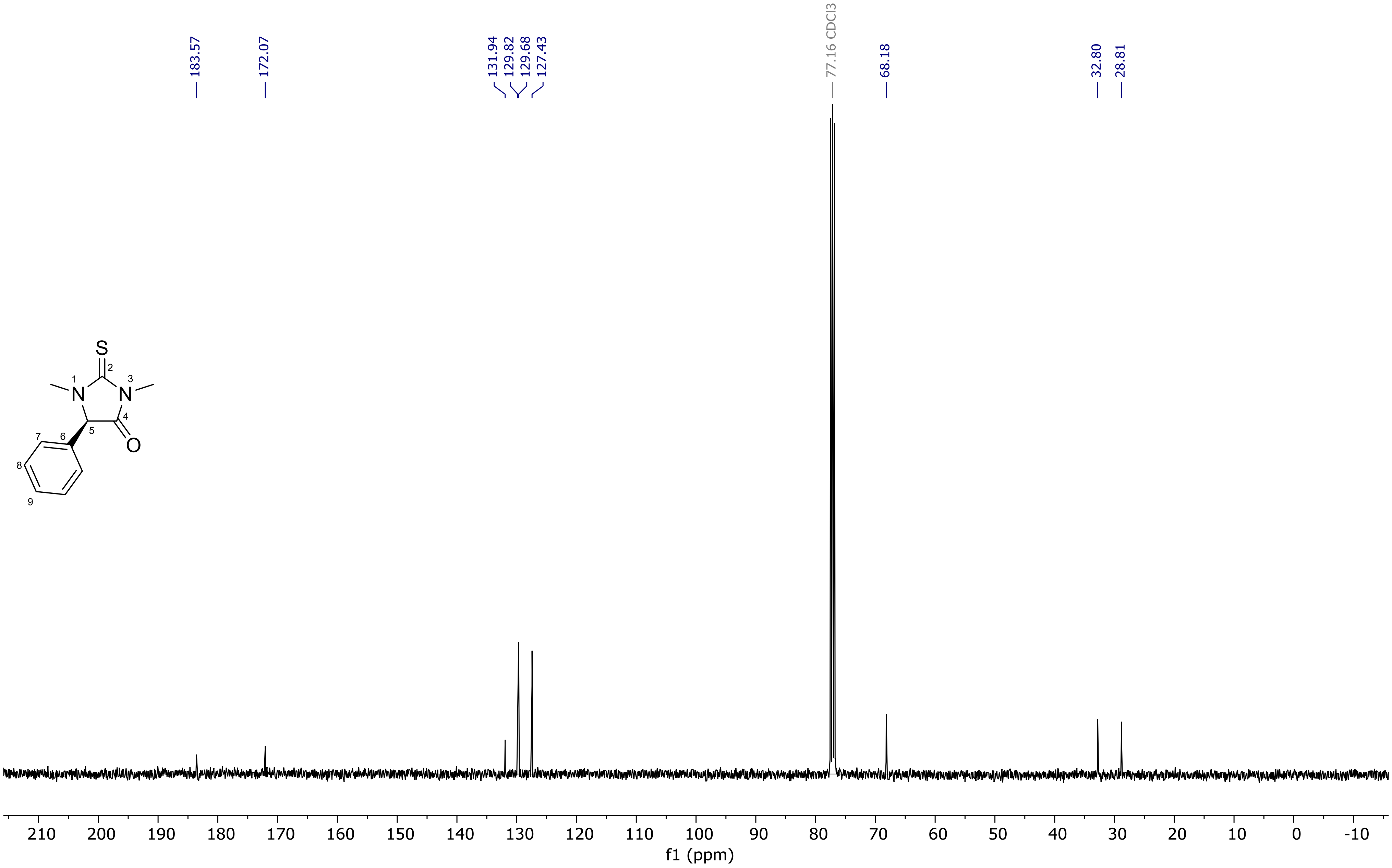

1,3-Dibenzyl-5-tert-butylimidazolidine-2,4-dione S17y, ^{1}H NMR in CDCl$_3$

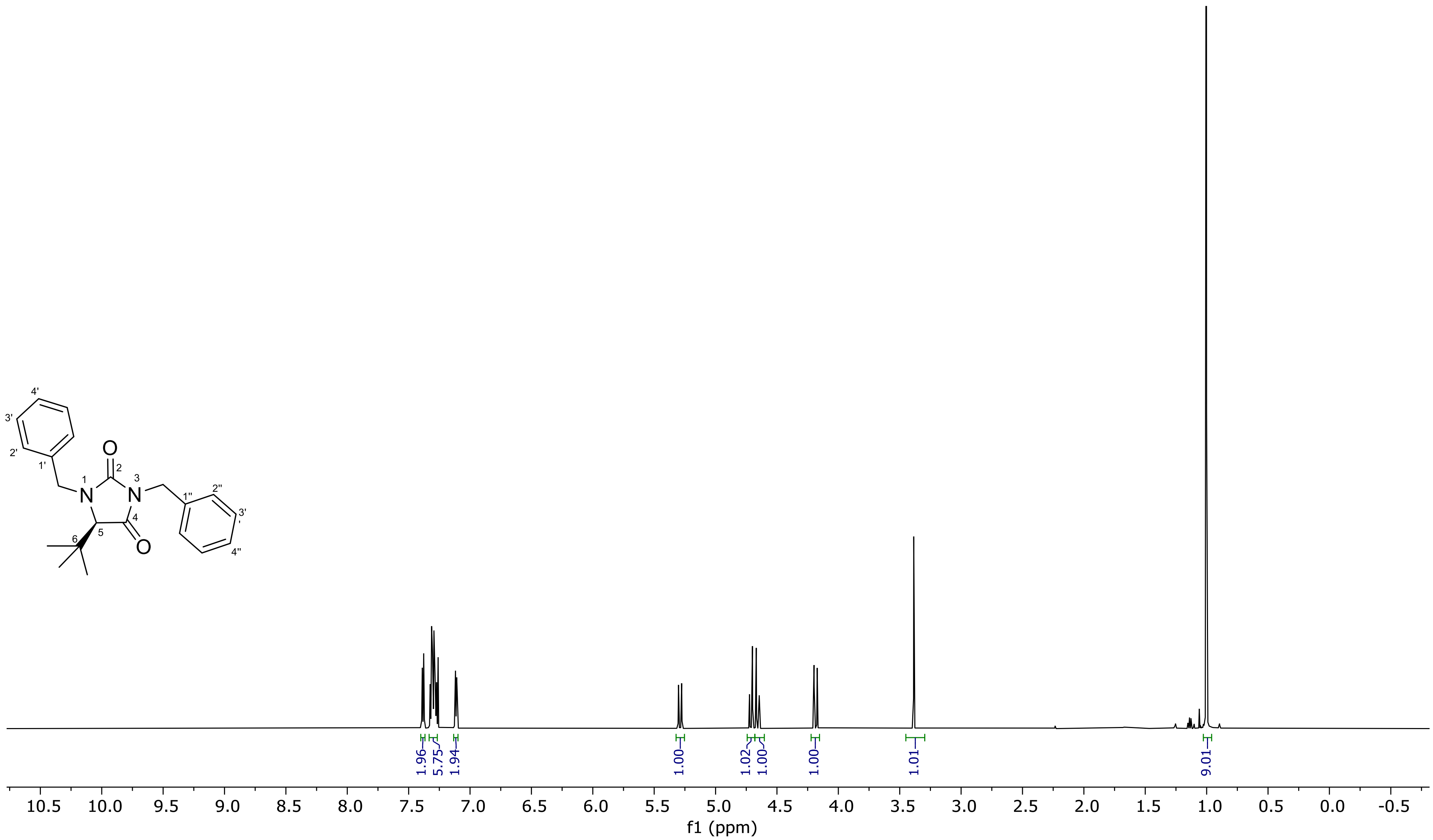

1,3-Dibenzyl-5-tert-butylimidazolidine-2,4-dione S17y, ^{13}C NMR in $CDCl_3$

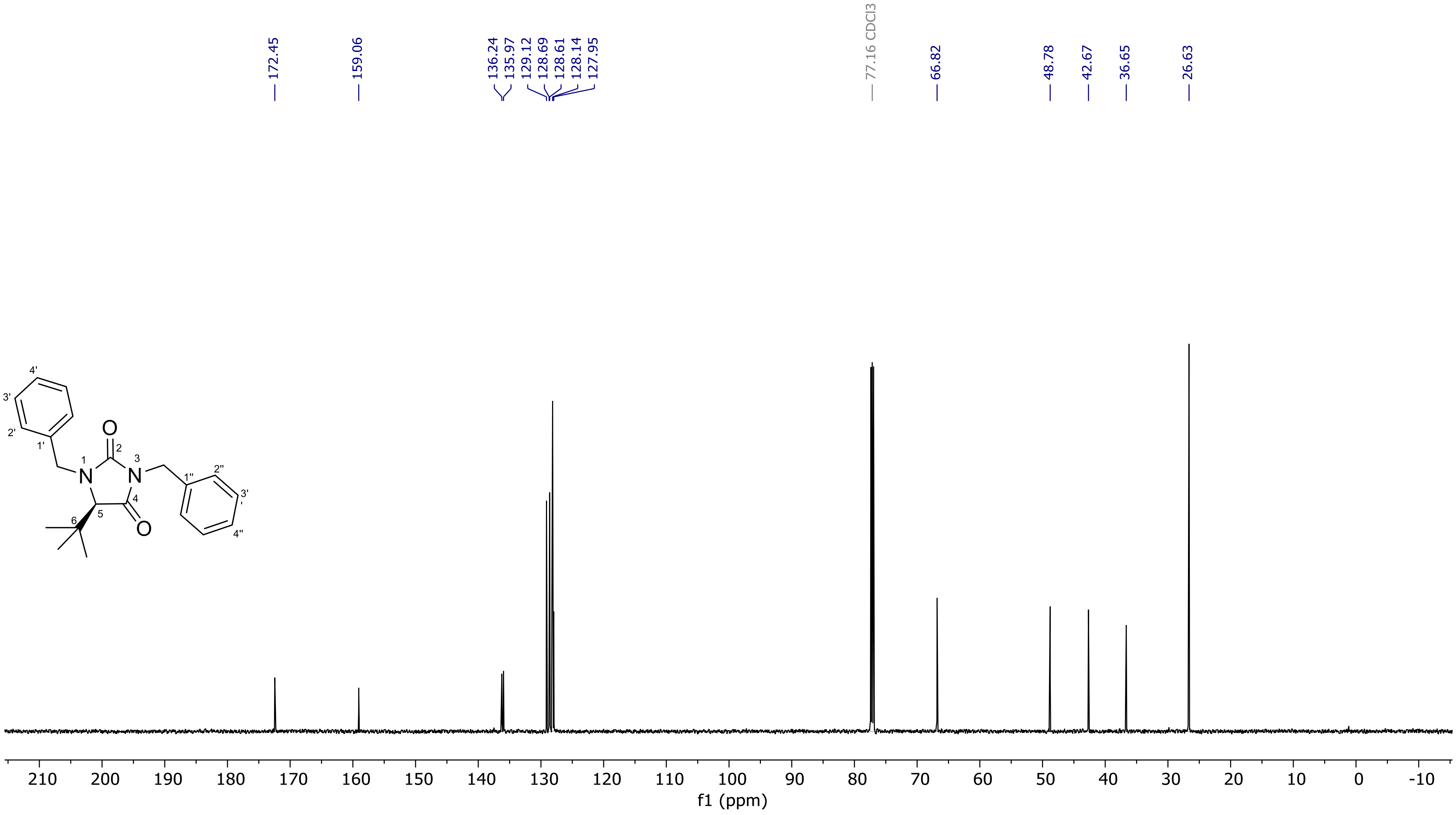

5-(Adamantan-1-yl)-1,3-dibenzylimidazolidine-2,4-dione S17z, ^{1}H NMR in CDCl$_3$

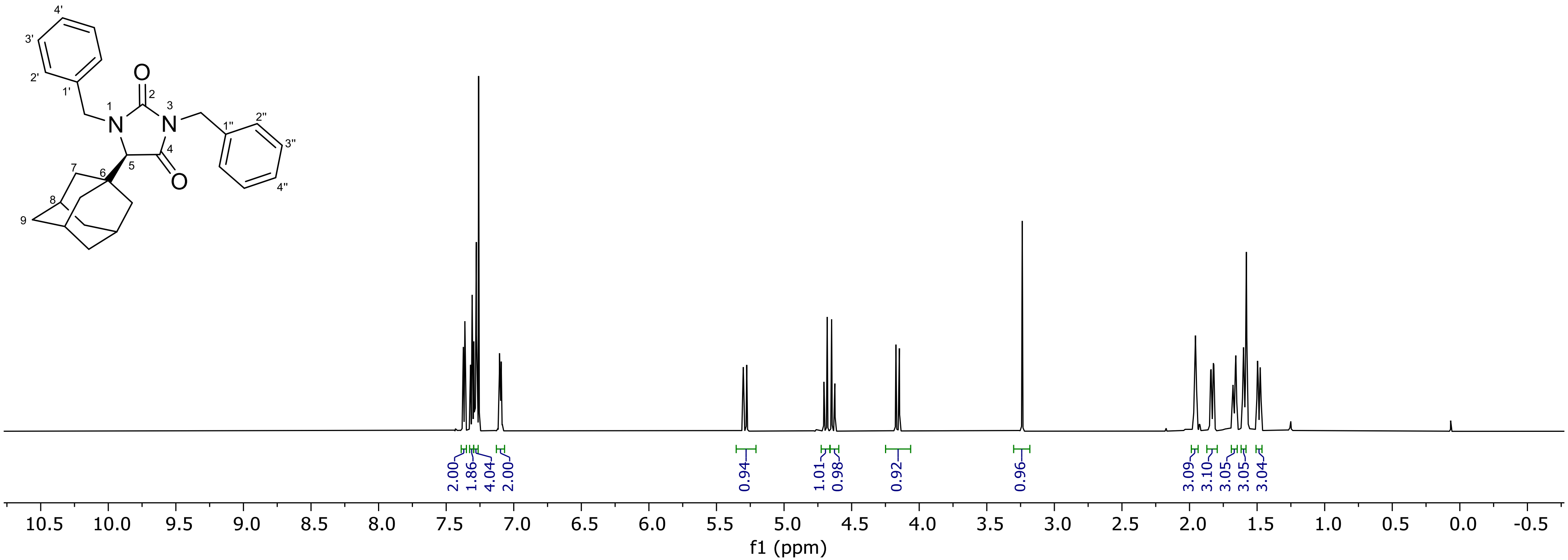

5-(Adamantan-1-yl)-1,3-dibenzylimidazolidine-2,4-dione S17z, ^{13}C NMR in CDCl$_3$

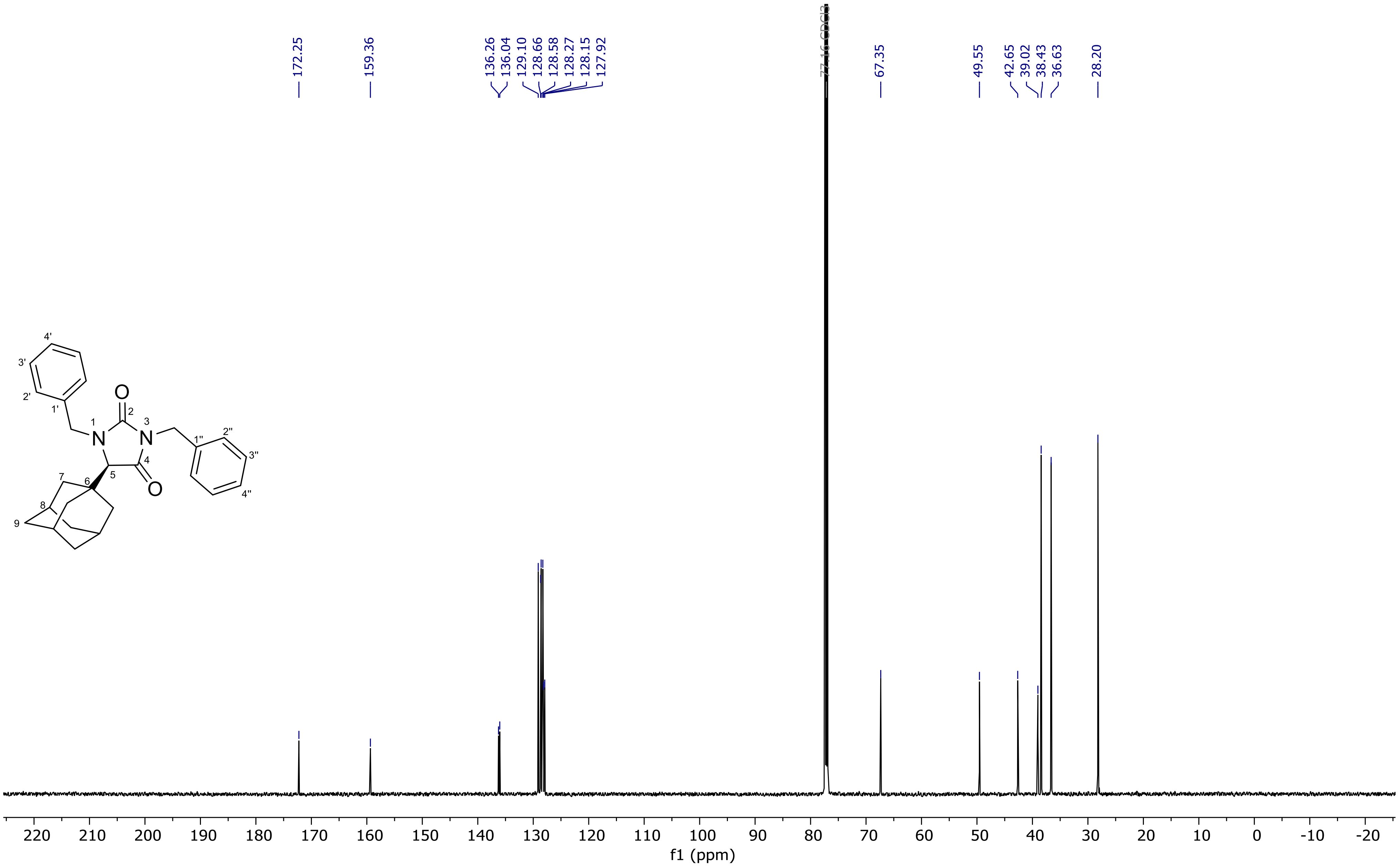

1,3-Dibenzyl-5-cyclohexylimidazolidine-2,4-dione S17aa, ^{1}H NMR in $CDCl_3$

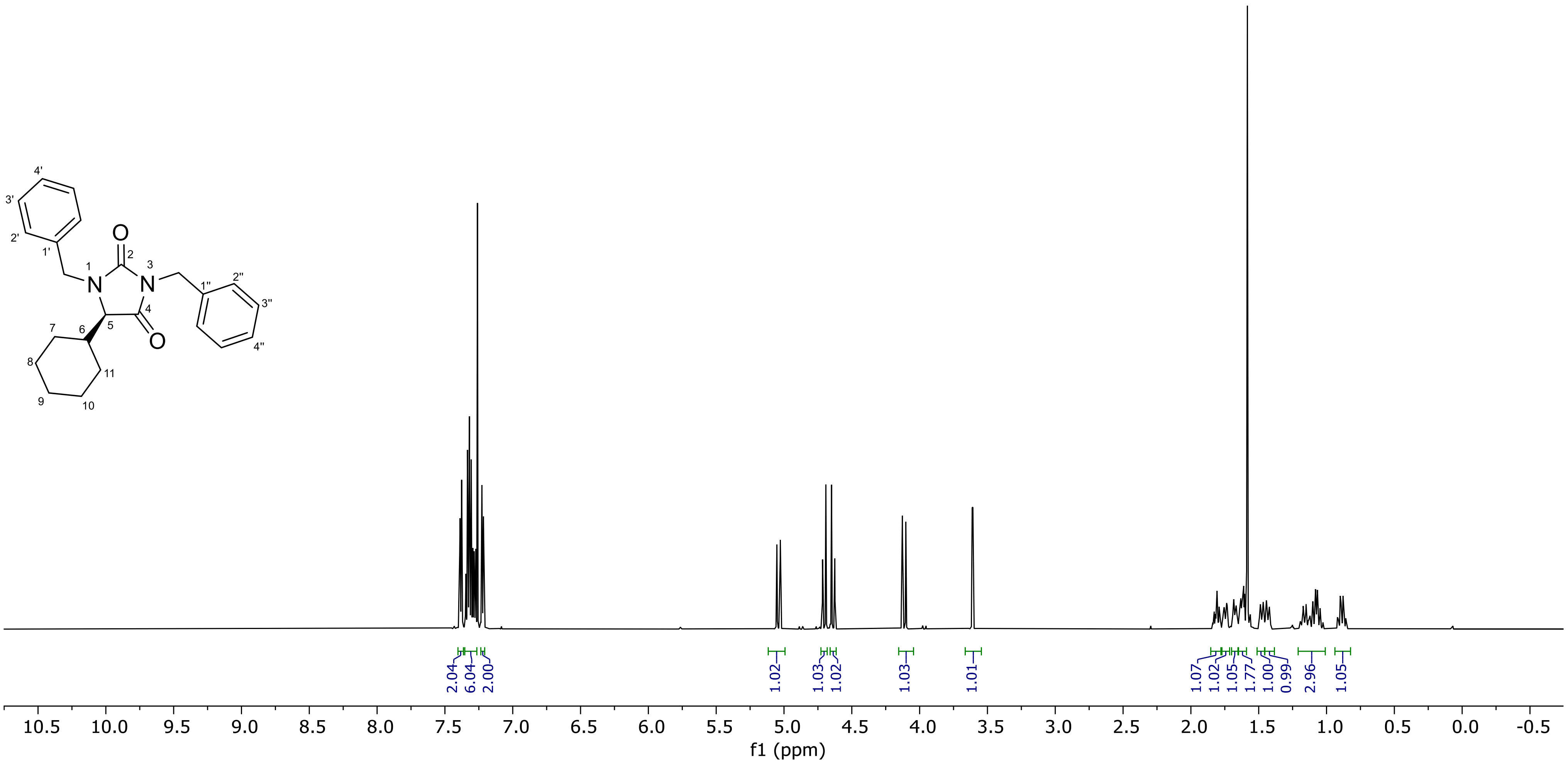

1,3-Dibenzyl-5-cyclohexylimidazolidine-2,4-dione S17aa, ^{13}C NMR in $CDCl_3$

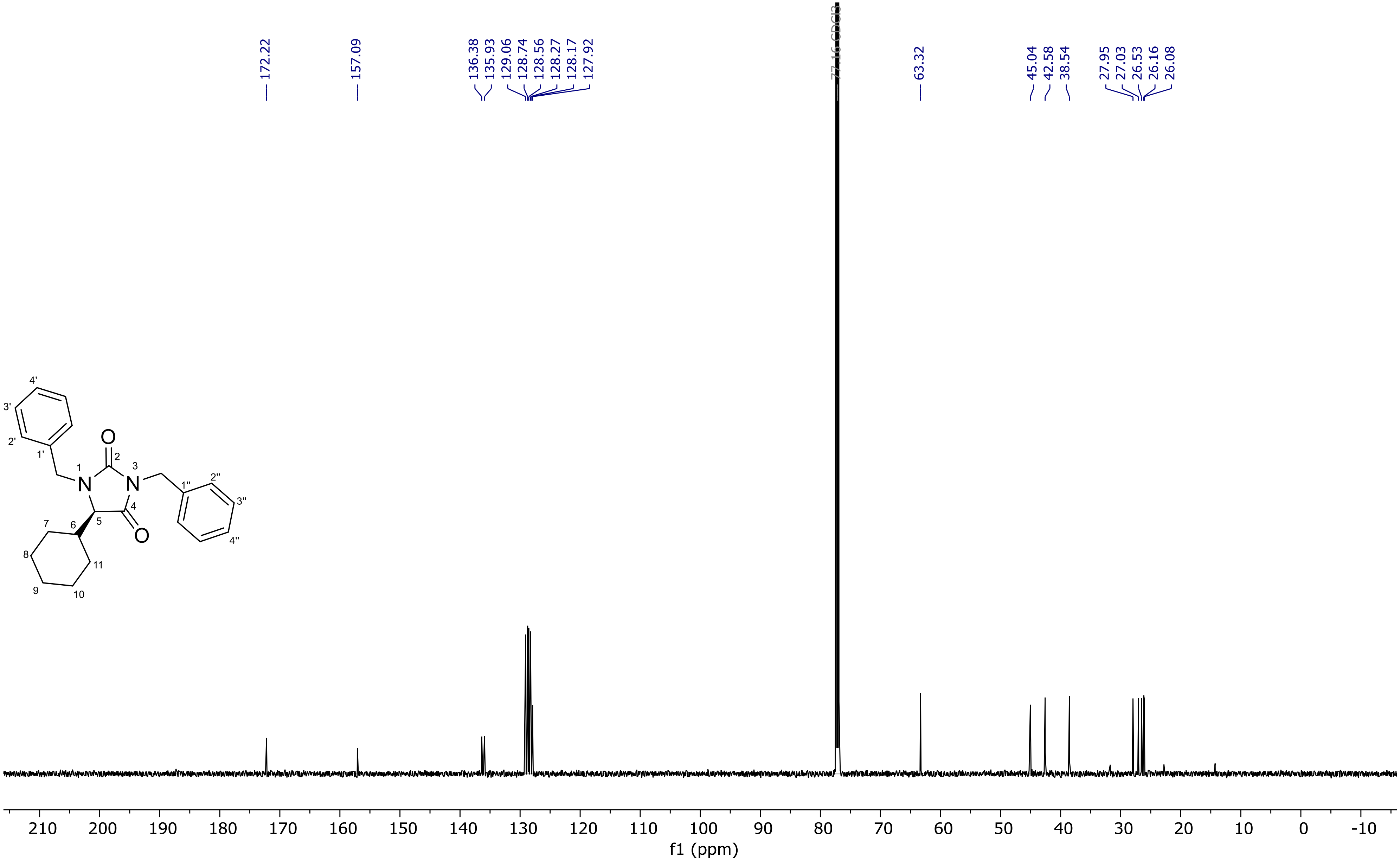

1,3-Dibenzyl-5-cyclopropylimidazolidine-2,4-dione S17ab, ^{1}H NMR in $CDCl_3$

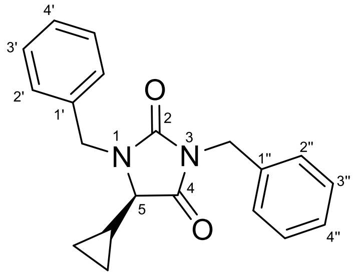

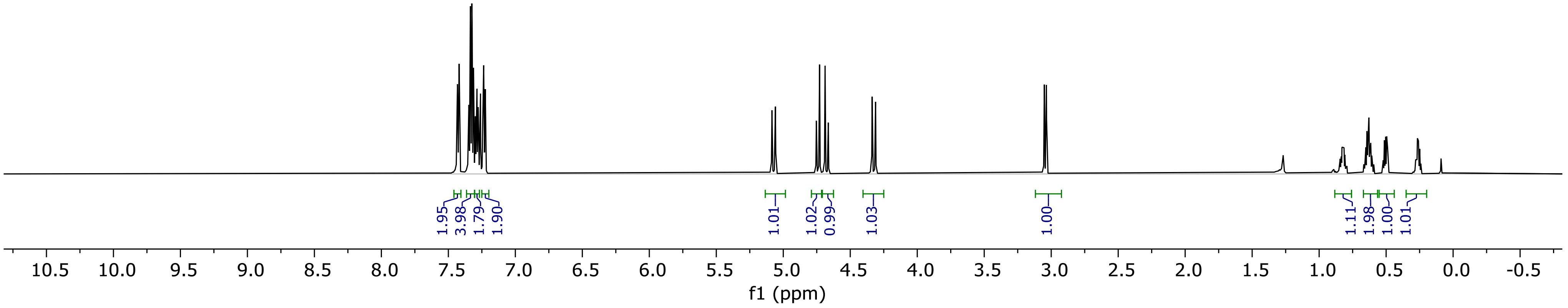

1,3-Dibenzyl-5-cyclopropylimidazolidine-2,4-dione S17ab, ^{13}C NMR in CDCl$_3$

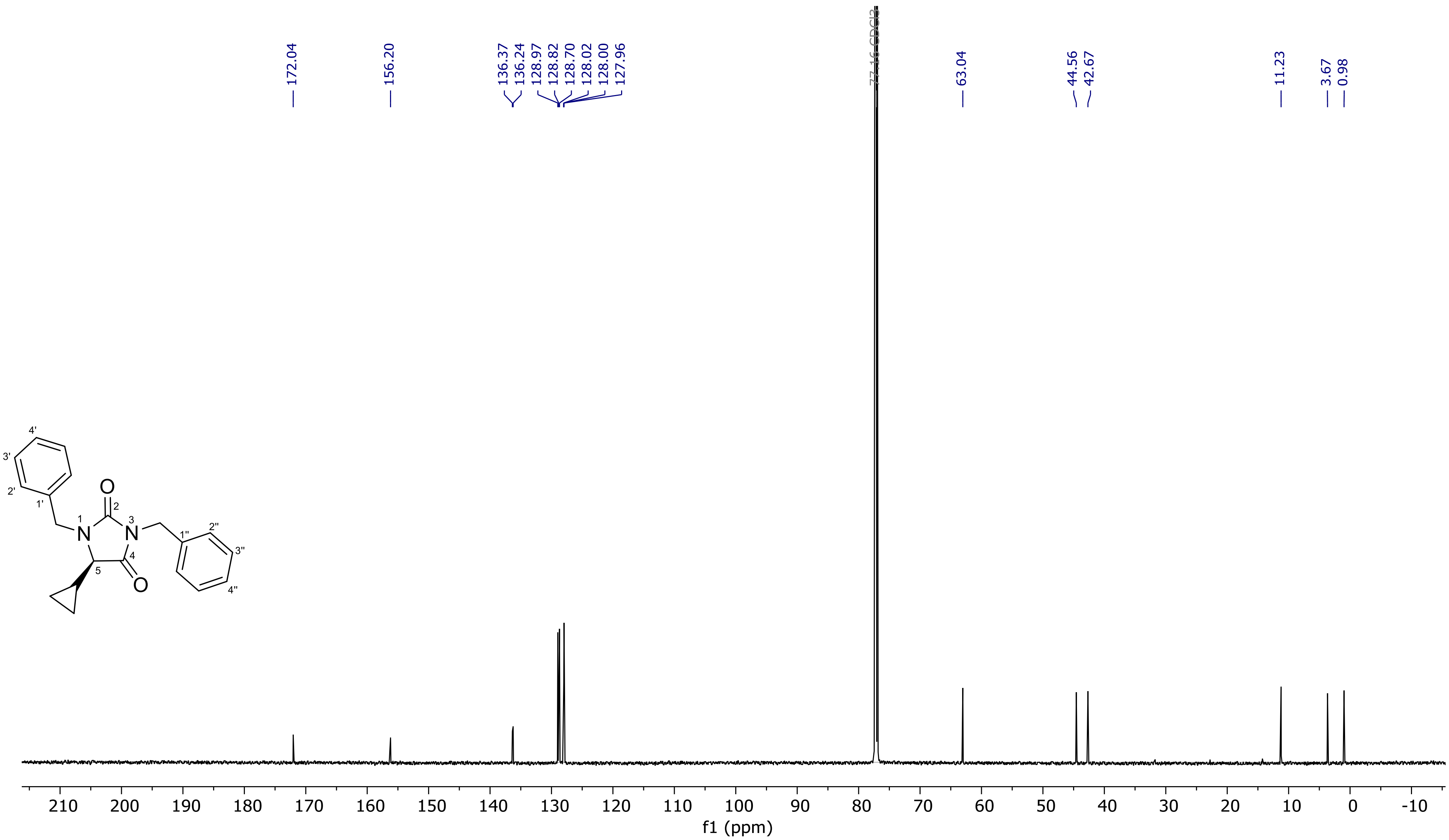

1,3,5-Tribenzylimidazolidine-2,4-dione S17ac, ^{1}H NMR in $CDCl_3$

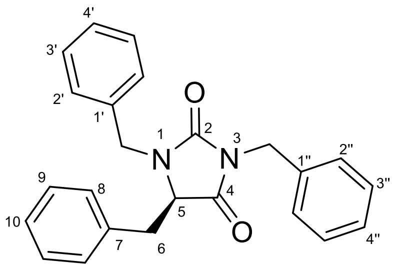

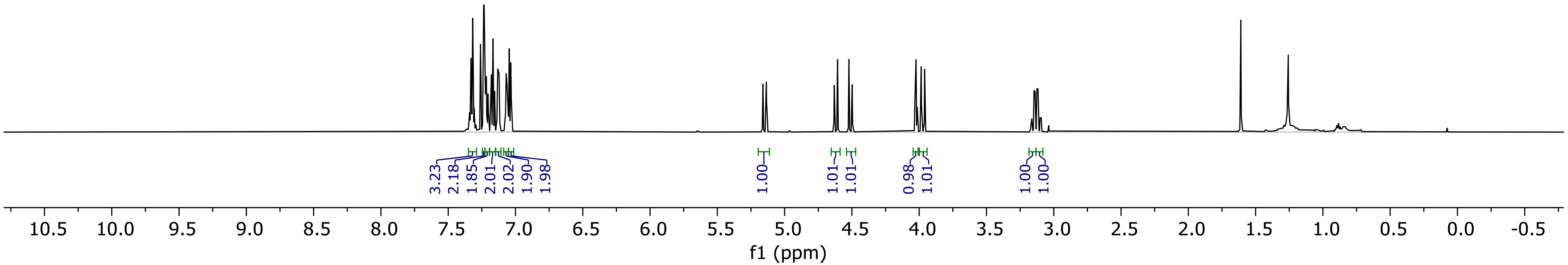

1,3,5-Tribenzylimidazolidine-2,4-dione S17ac, ^{13}C NMR in $CDCl_3$

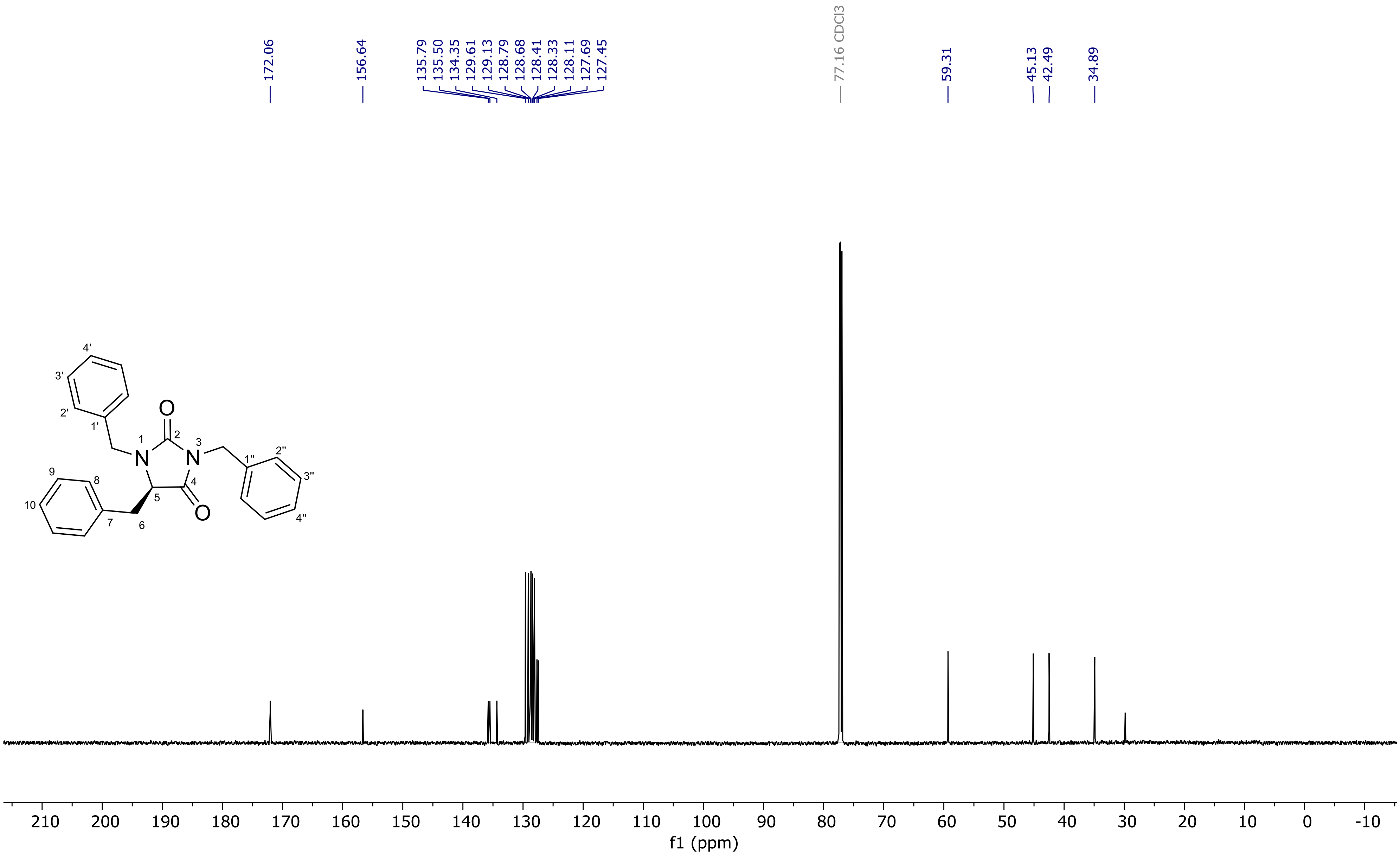

3-[(2,4-Dimethoxyphenyl)methyl]-5-phenylimidazolidine-2,4-dione 22v-a, ^{1}H NMR in $CDCl_3$

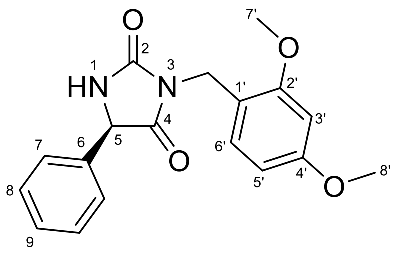

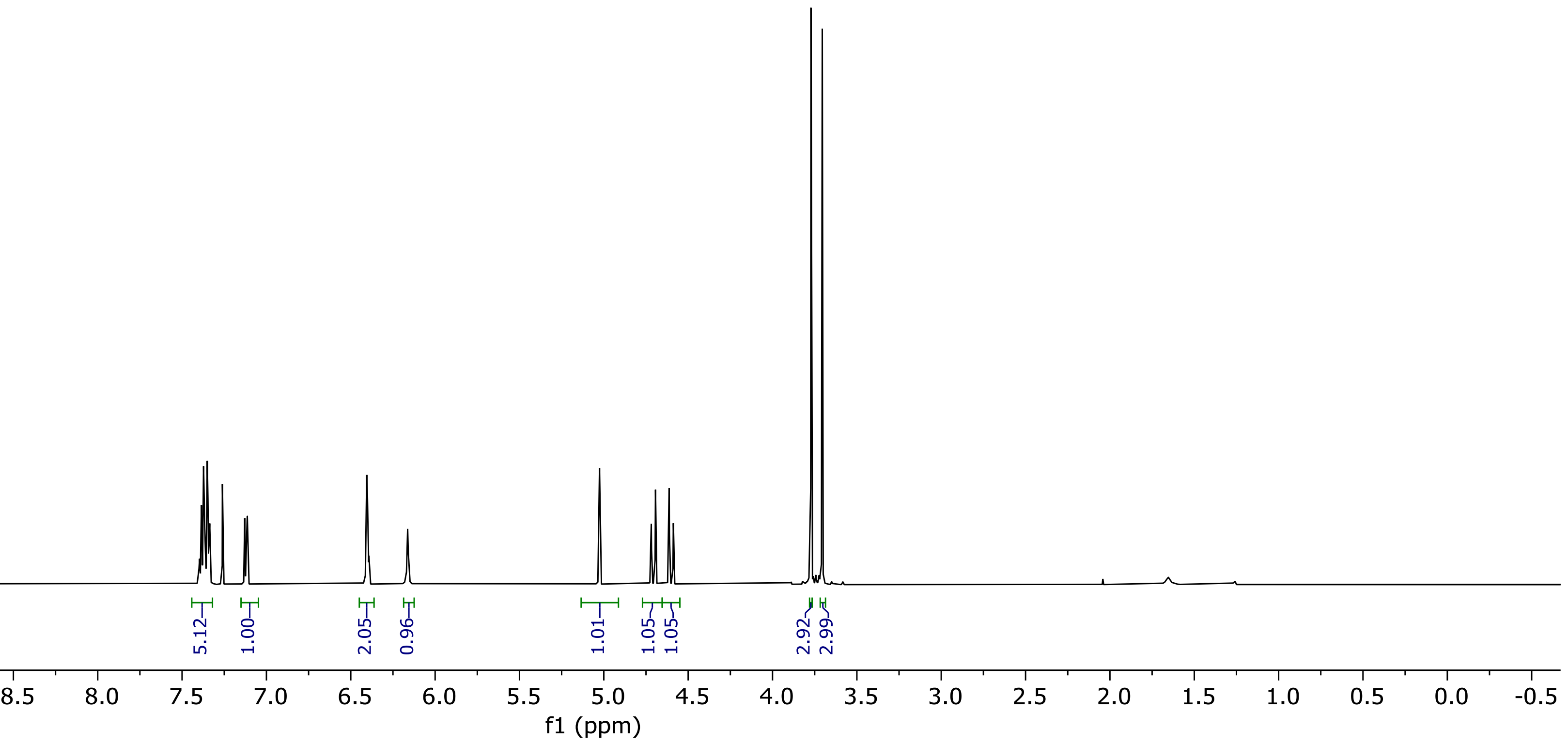

3-[(2,4-Dimethoxyphenyl)methyl]-5-phenylimidazolidine-2,4-dione 22v-a, ^{13}C NMR in $CDCl_3$

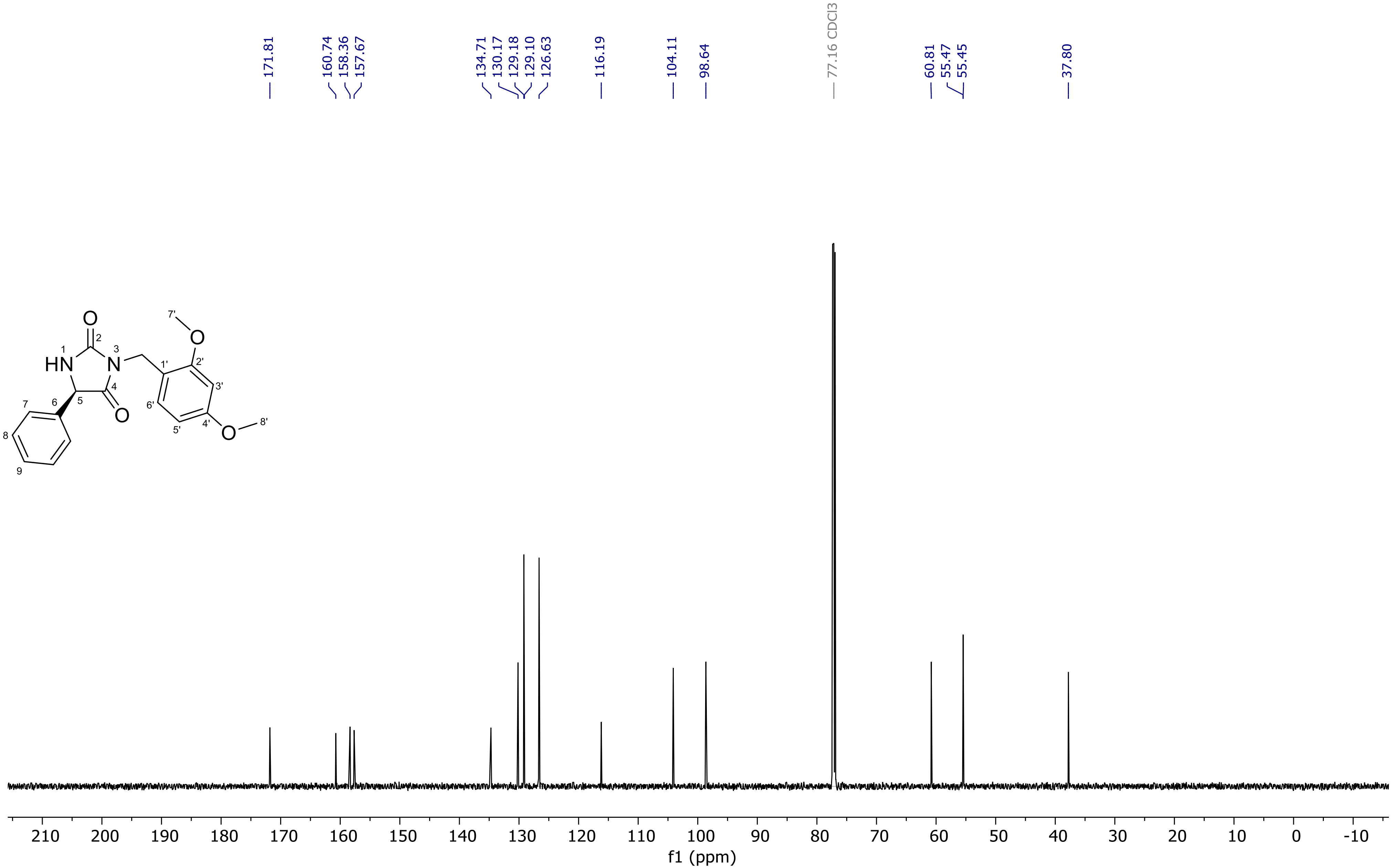

3-[(2,4-Dimethoxyphenyl)methyl]-5-phenylimidazolidine-2,4-dione 22v-a, HMBC correlations in $CDCl_3$

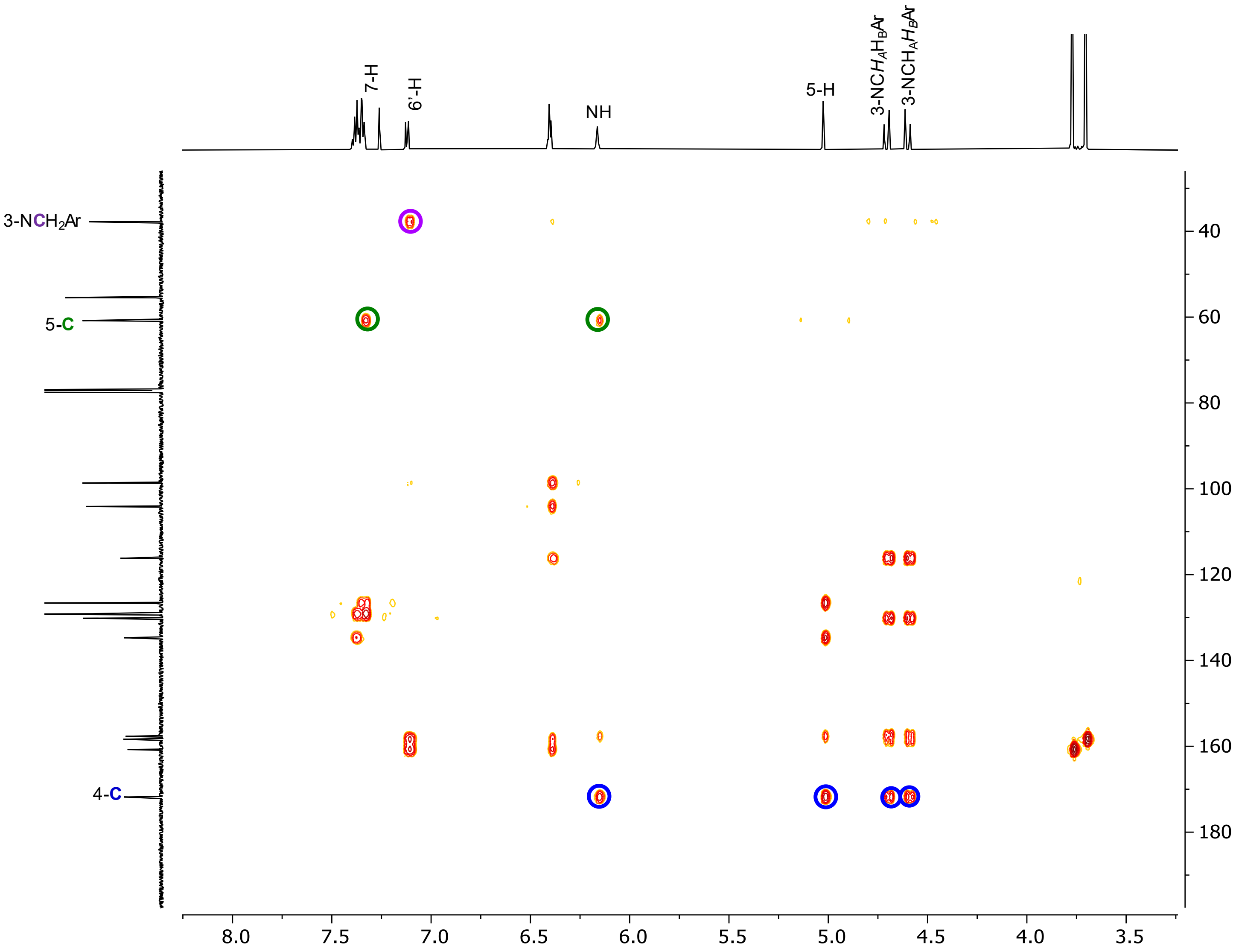

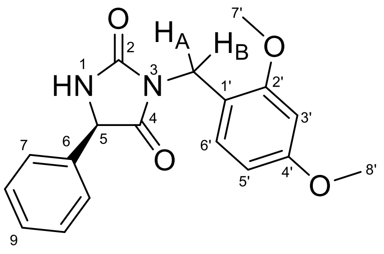

HMBC key correlations:

→ 3-NCH$_2$Ar: 6'-H (^3J)

→ 5-C: 7-H (^3J), NH (^2J)

→ 4-C: 5-H (^2J), 1-NCH$_2$Ar (^3J), NH (^3J)

1,3-Dibenzyl-4-phenylimidazolidine 23a, ^{1}H NMR in $CDCl_3$

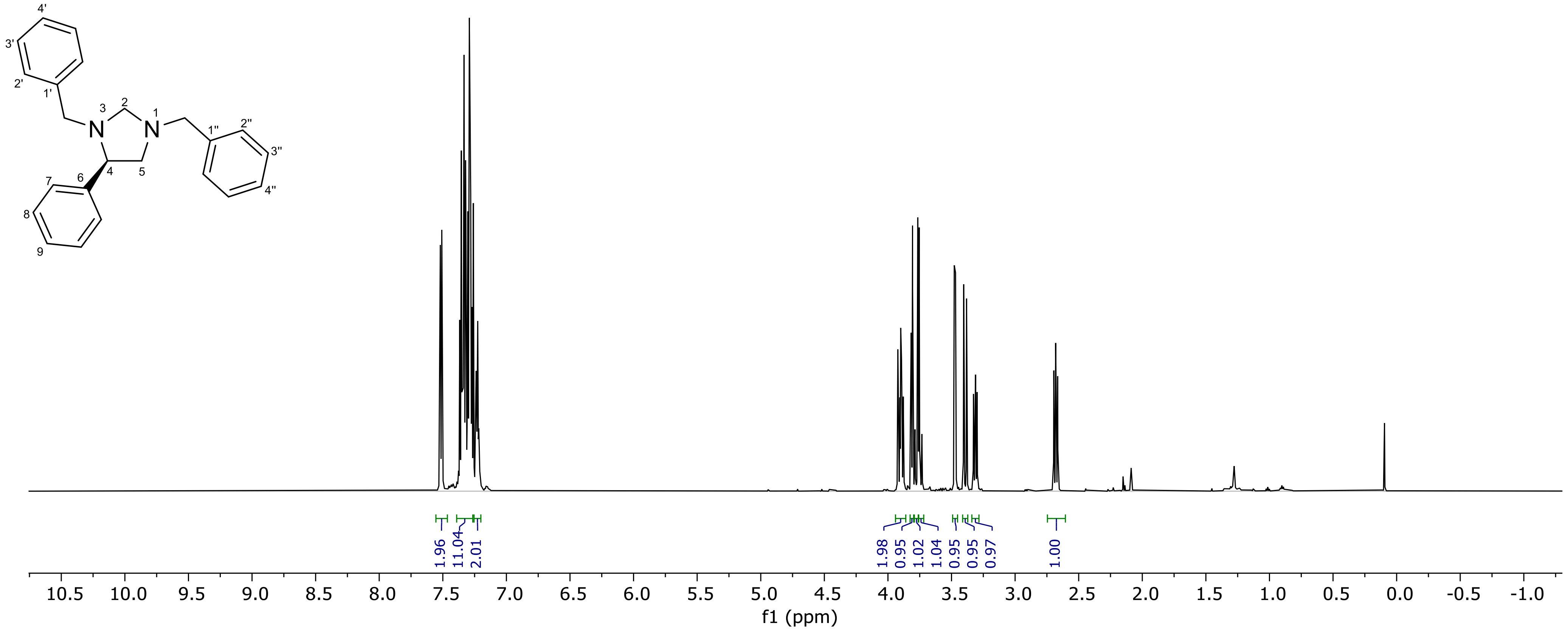

1,3-Dibenzyl-4-phenylimidazolidine 23a, ^{13}C NMR in $CDCl_3$

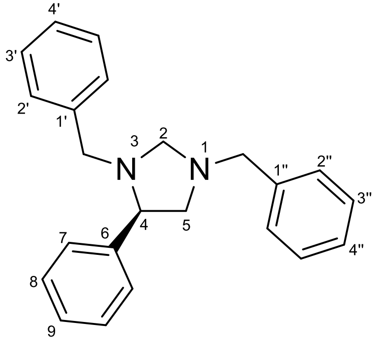

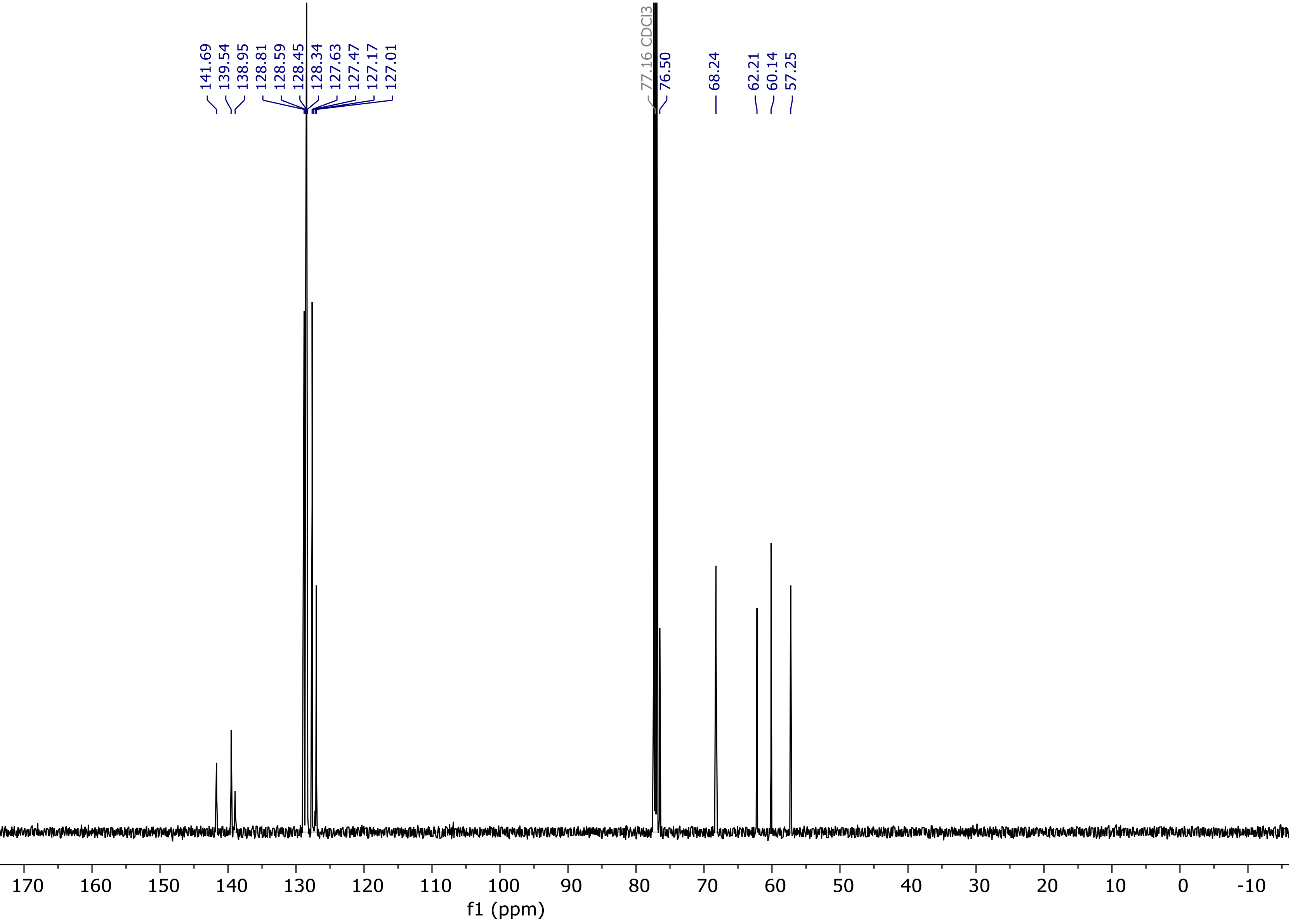

Benzyl[2-(benzylamino)-1-phenylethyl]amine 24a, ^{1}H NMR in $CDCl_3$

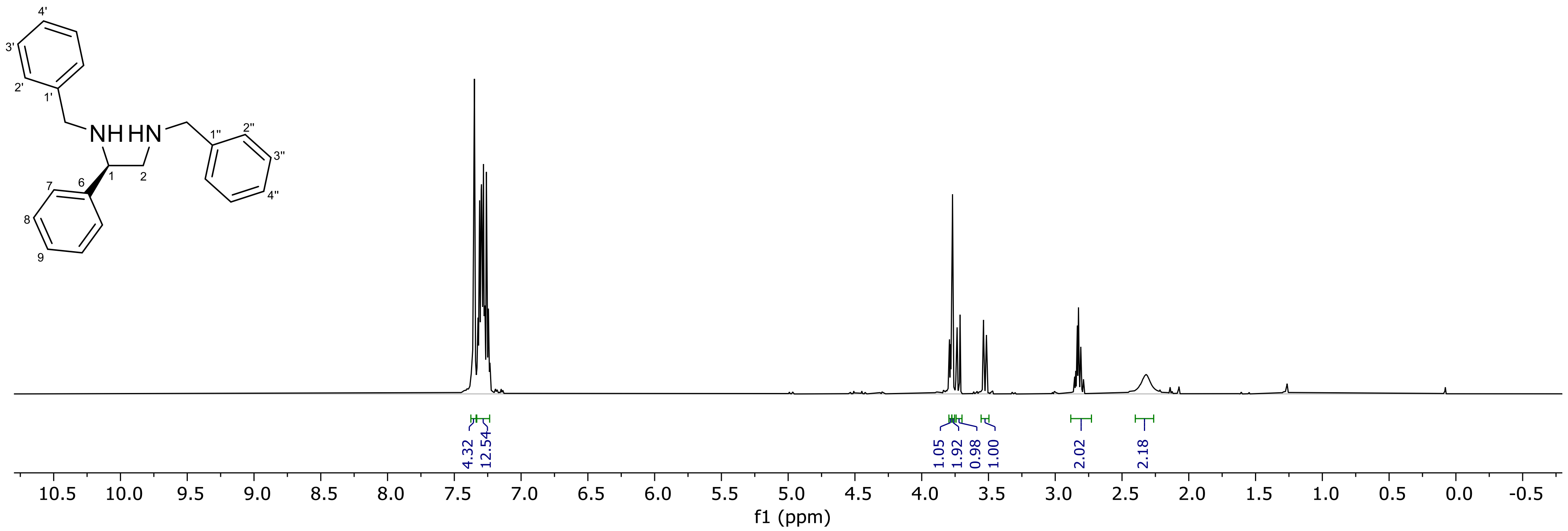

Benzyl[2-(benzylamino)-1-phenylethyl]amine 24a, ^{13}C NMR in $CDCl_3$

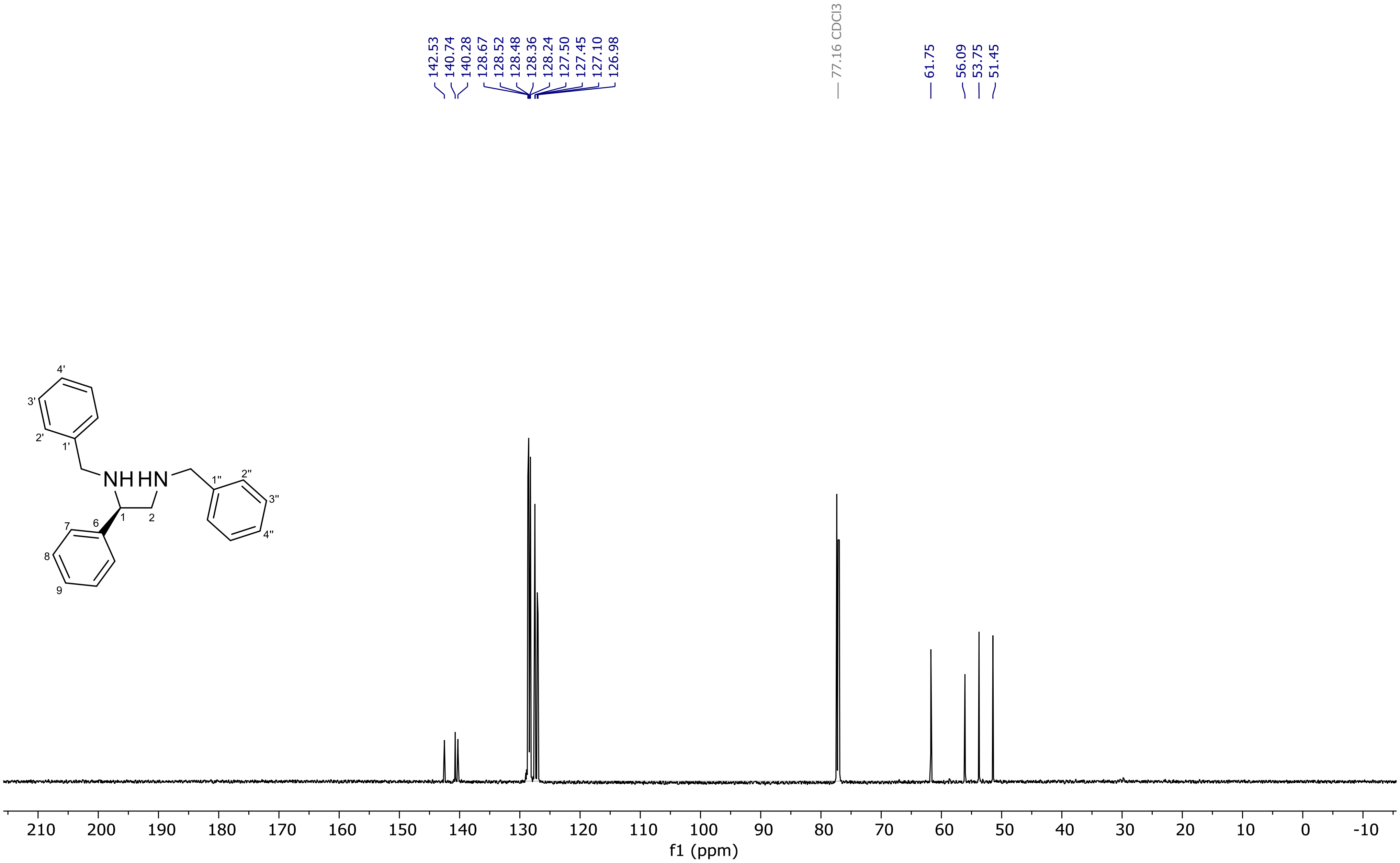

1,3-Dibenzyl-4,5-dihydroxy-4-phenylimidazolidin-2-one 15a, ¹H NMR in DMSO-d₆

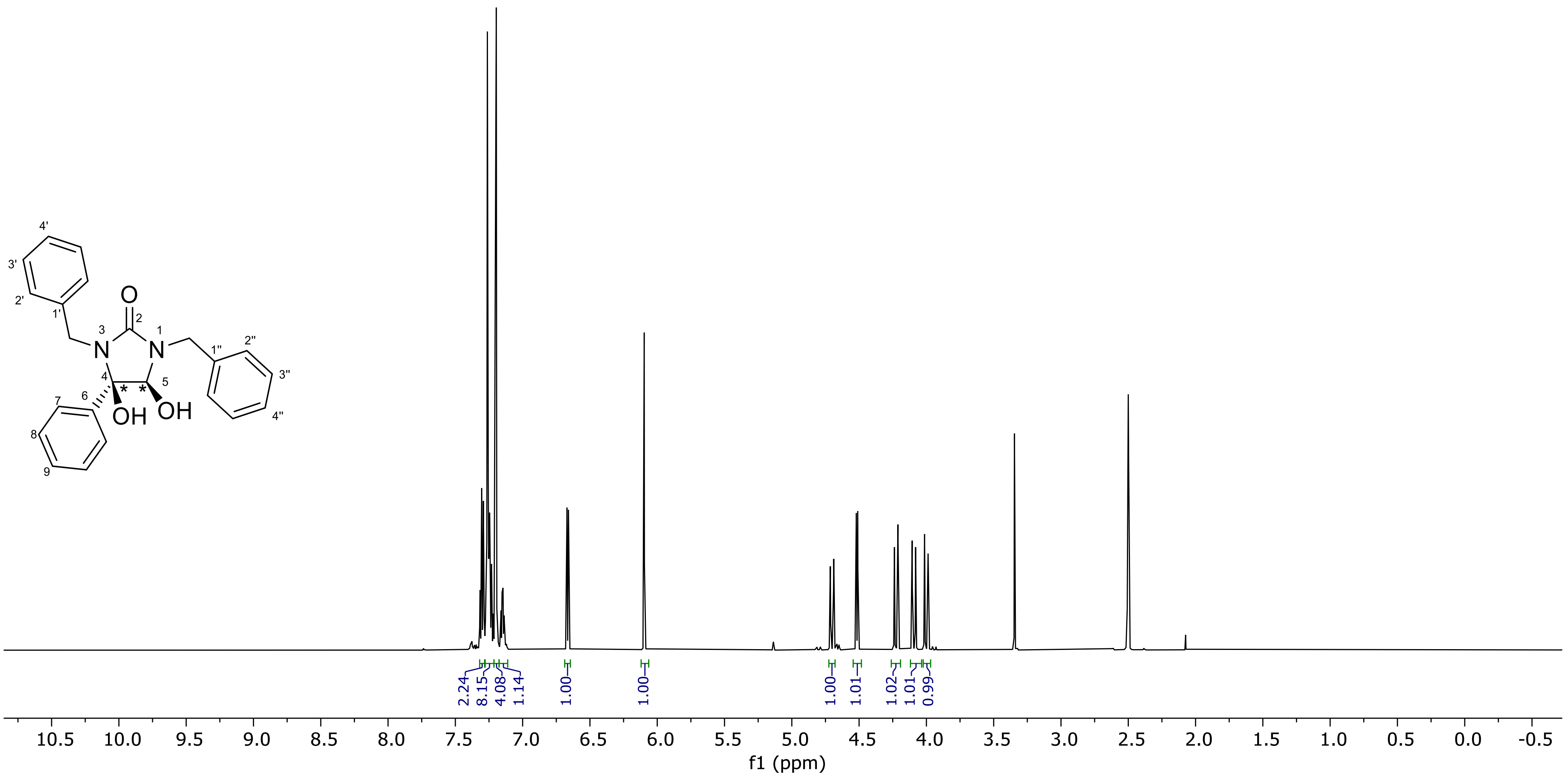

1,3-Dibenzyl-4,5-dihydroxy-4-phenylimidazolidin-2-one 15a, ^{13}C NMR in DMSO-d$_6$

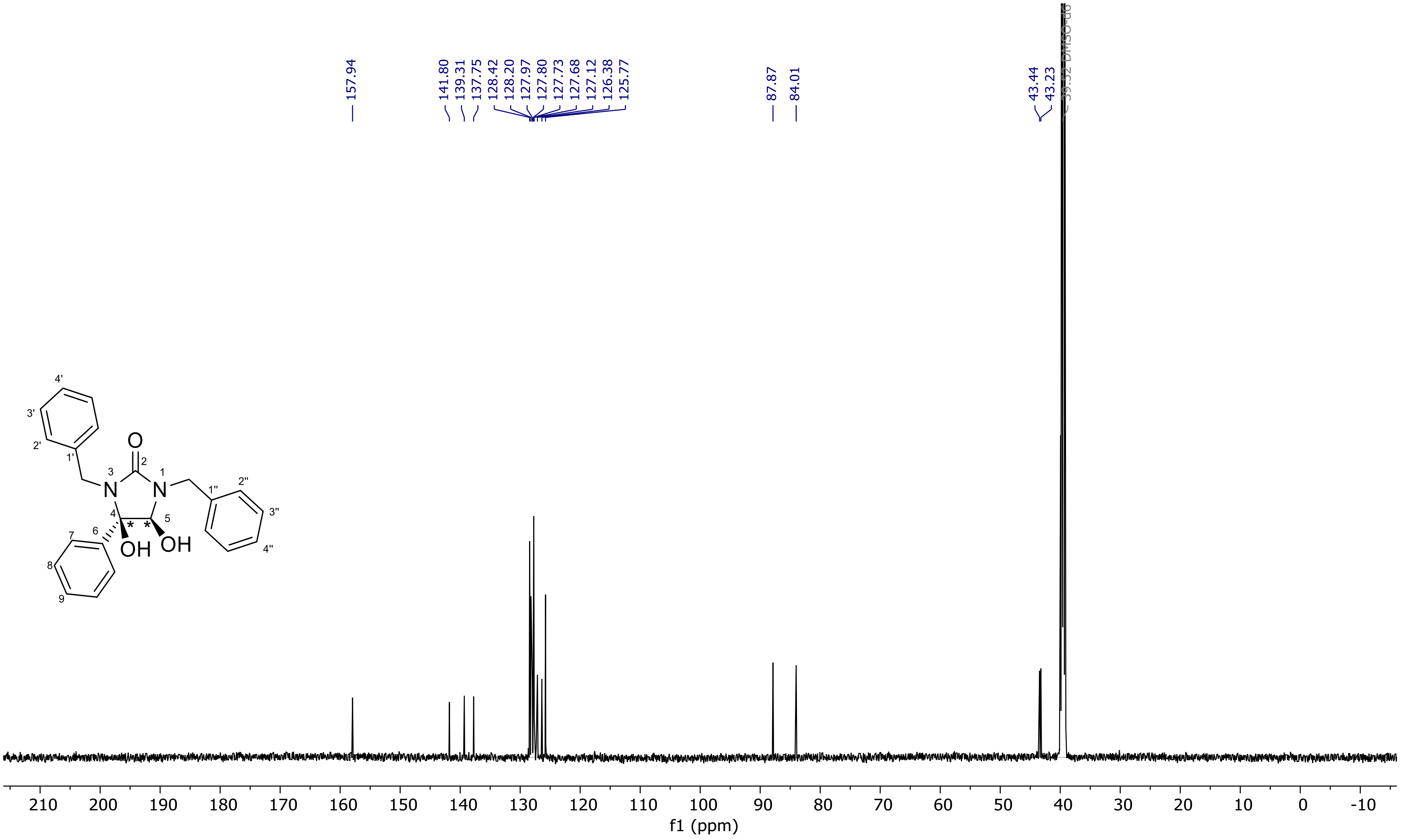

1,3-Dibenzyl-5-hydroxy-5-phenylimidazolidine-2,4-dione 21a, ^{1}H NMR in $CDCl_3$

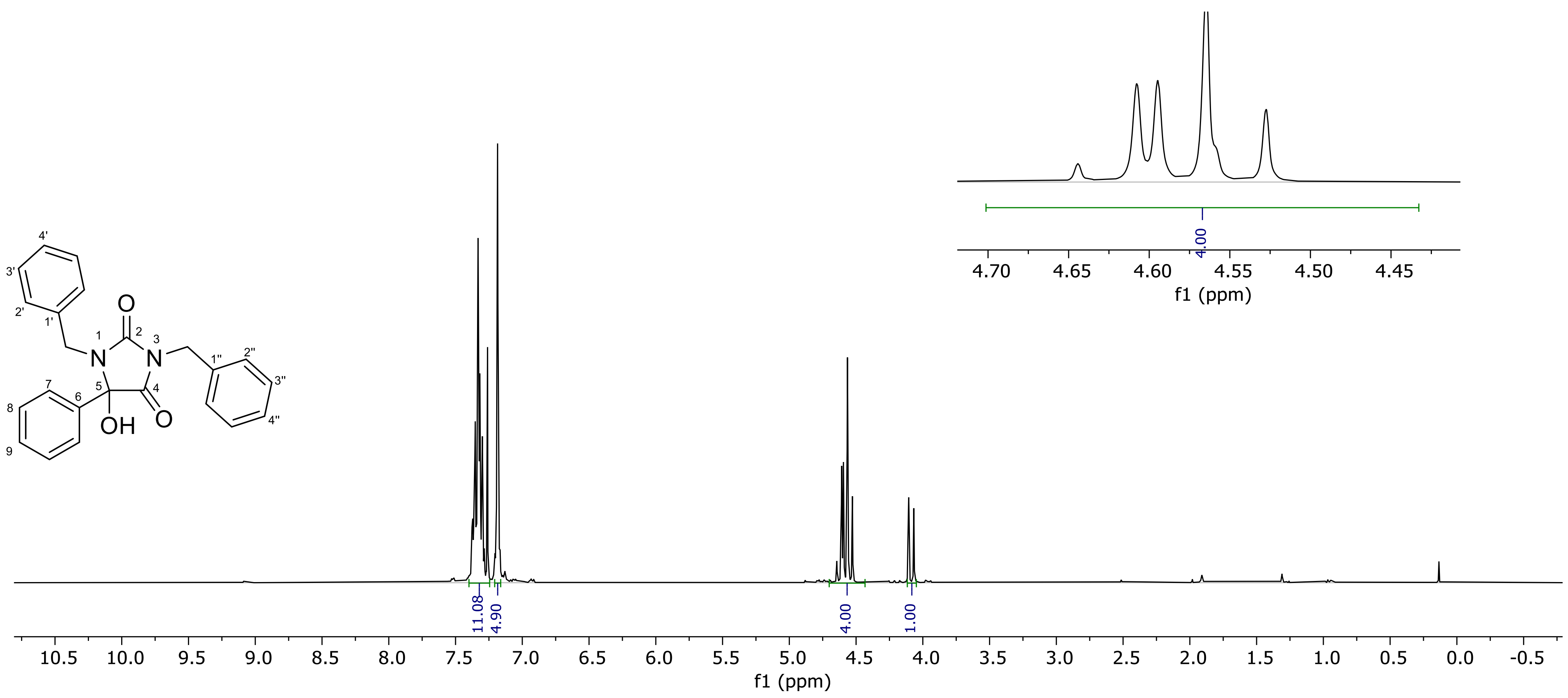

1,3-Dibenzyl-5-hydroxy-5-phenylimidazolidine-2,4-dione 21a, ^{13}C NMR in $CDCl_3$

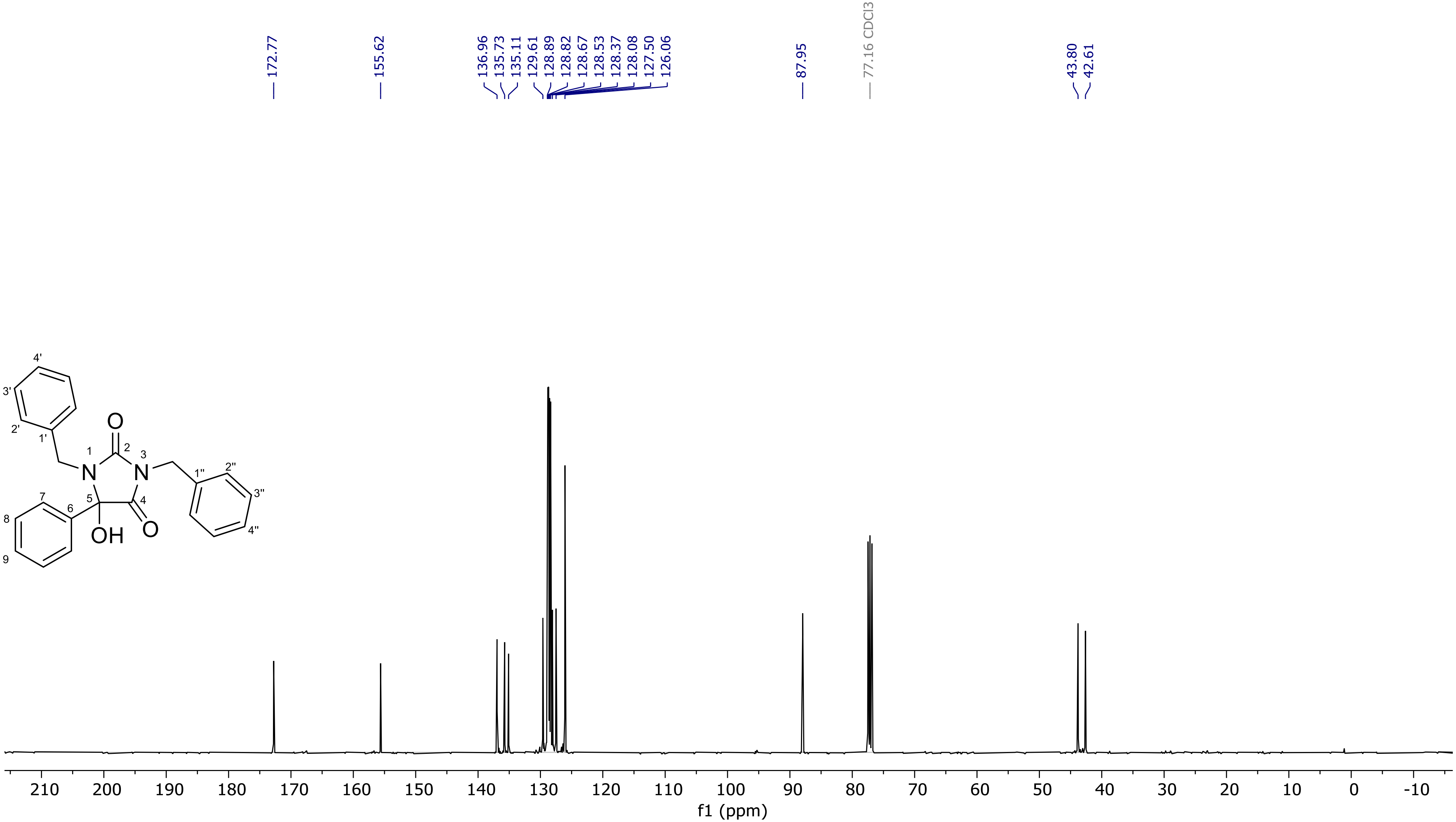

1,3-Dibenzylurea 14a, ^{1}H NMR in DMSO d_6

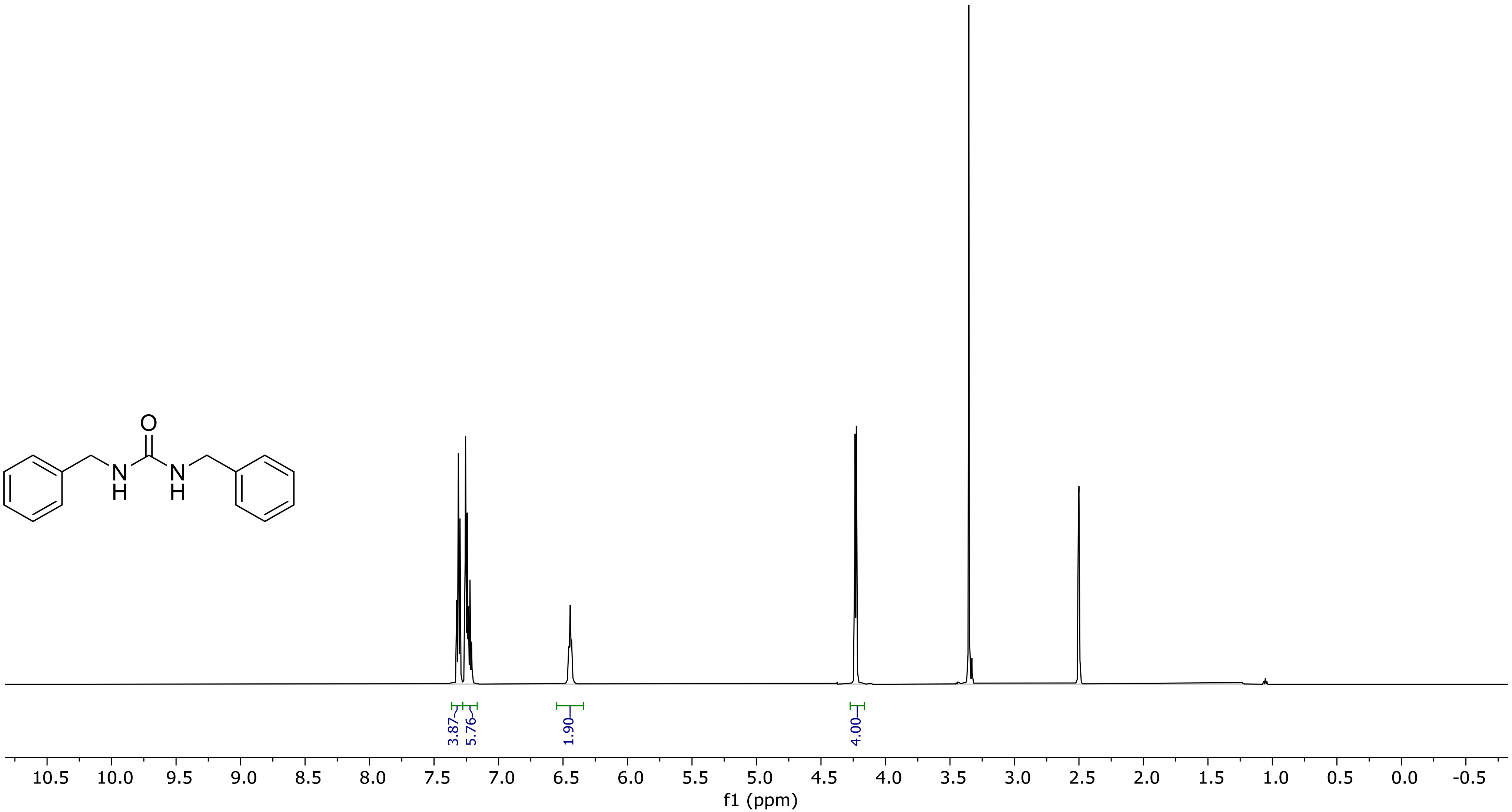

1,3-Dibenzylurea 14a, ^{13}C NMR in DMSO-d_6

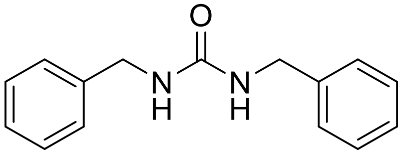

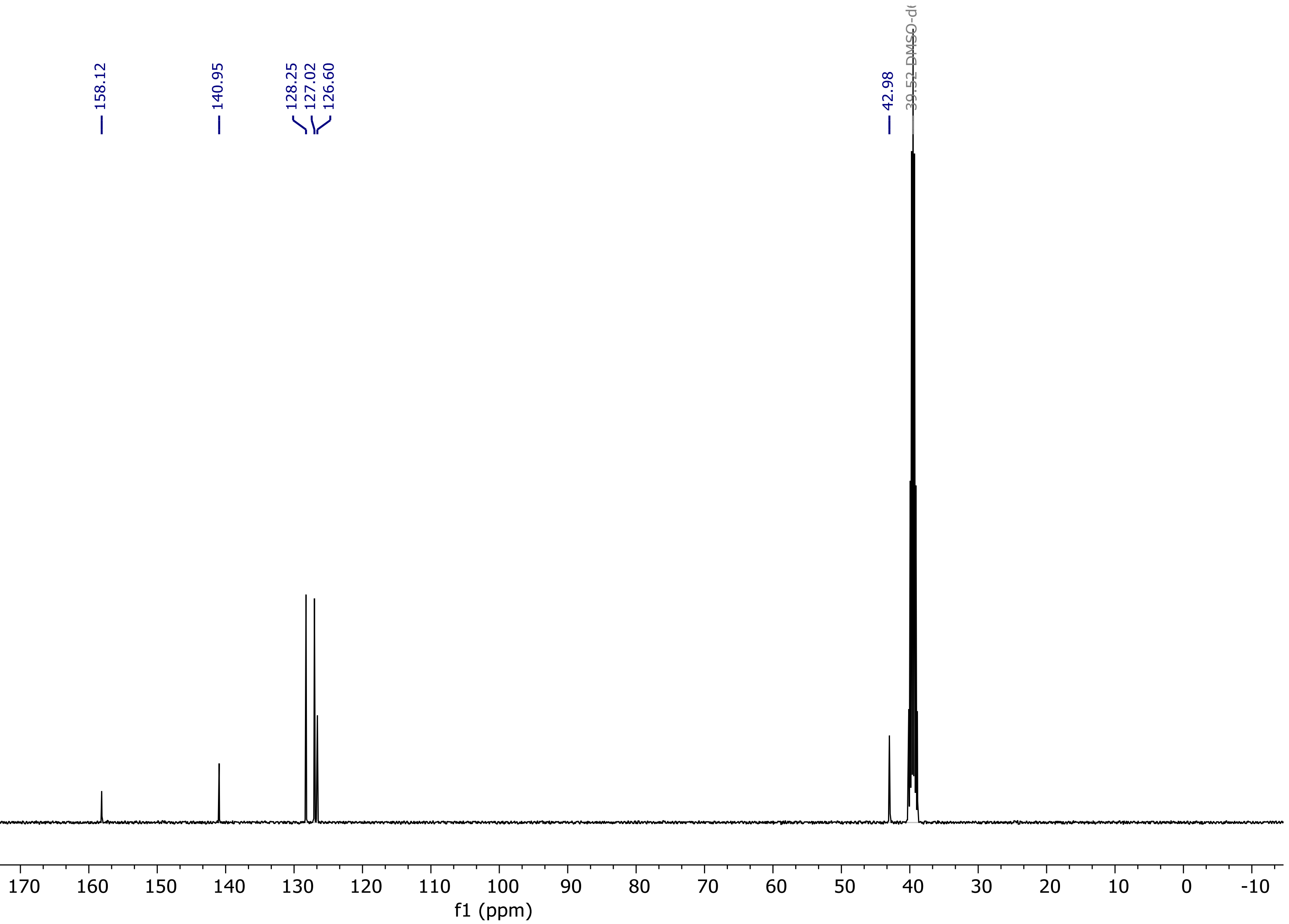

1,3-Bis[(2,4-dimethoxyphenyl)methyl]urea 14c, ^{1}H NMR in DMSO-d_6

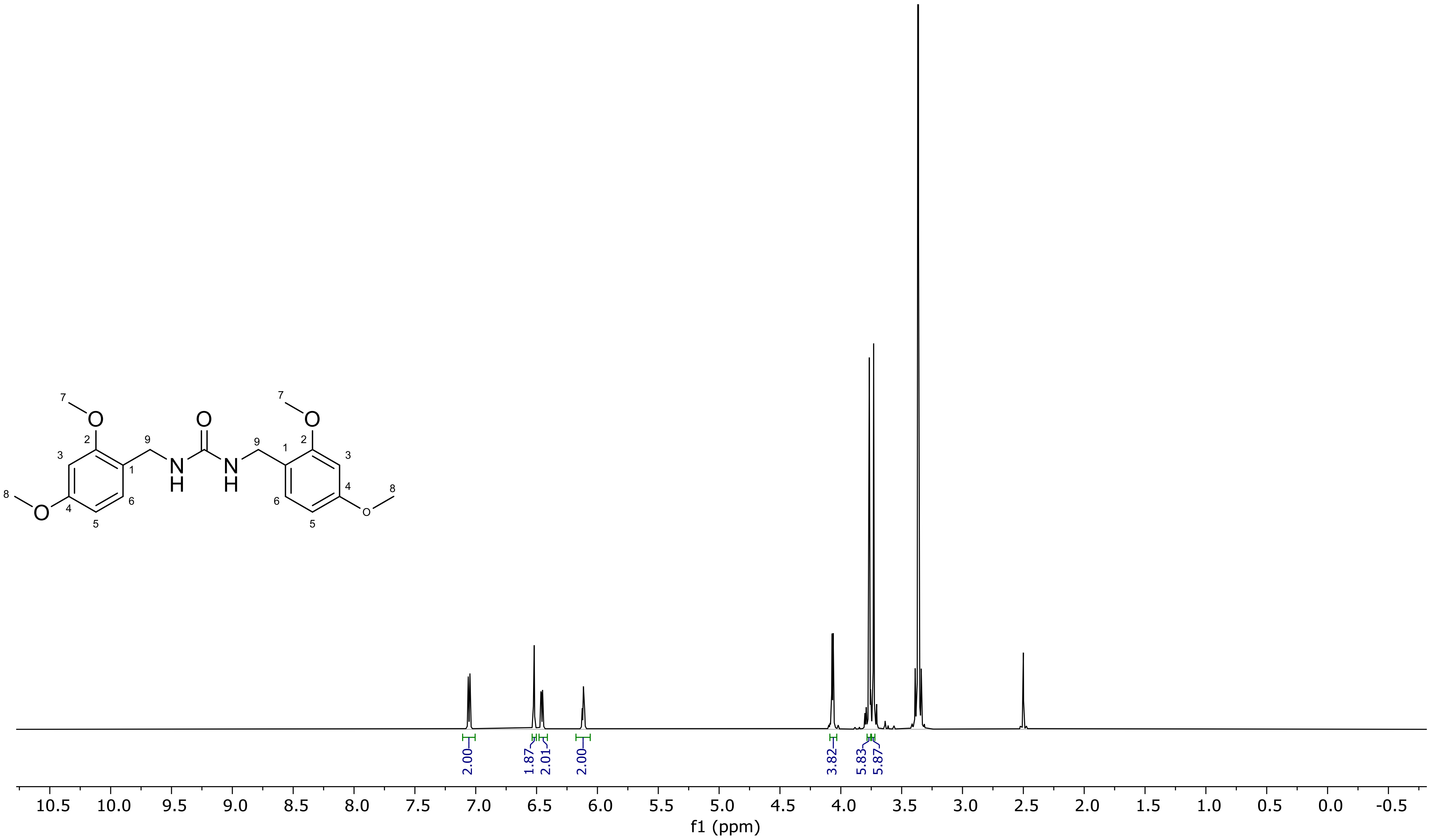

1,3-Bis[(2,4-dimethoxyphenyl)methyl]urea 14c, ^{13}C NMR in DMSO-d_6

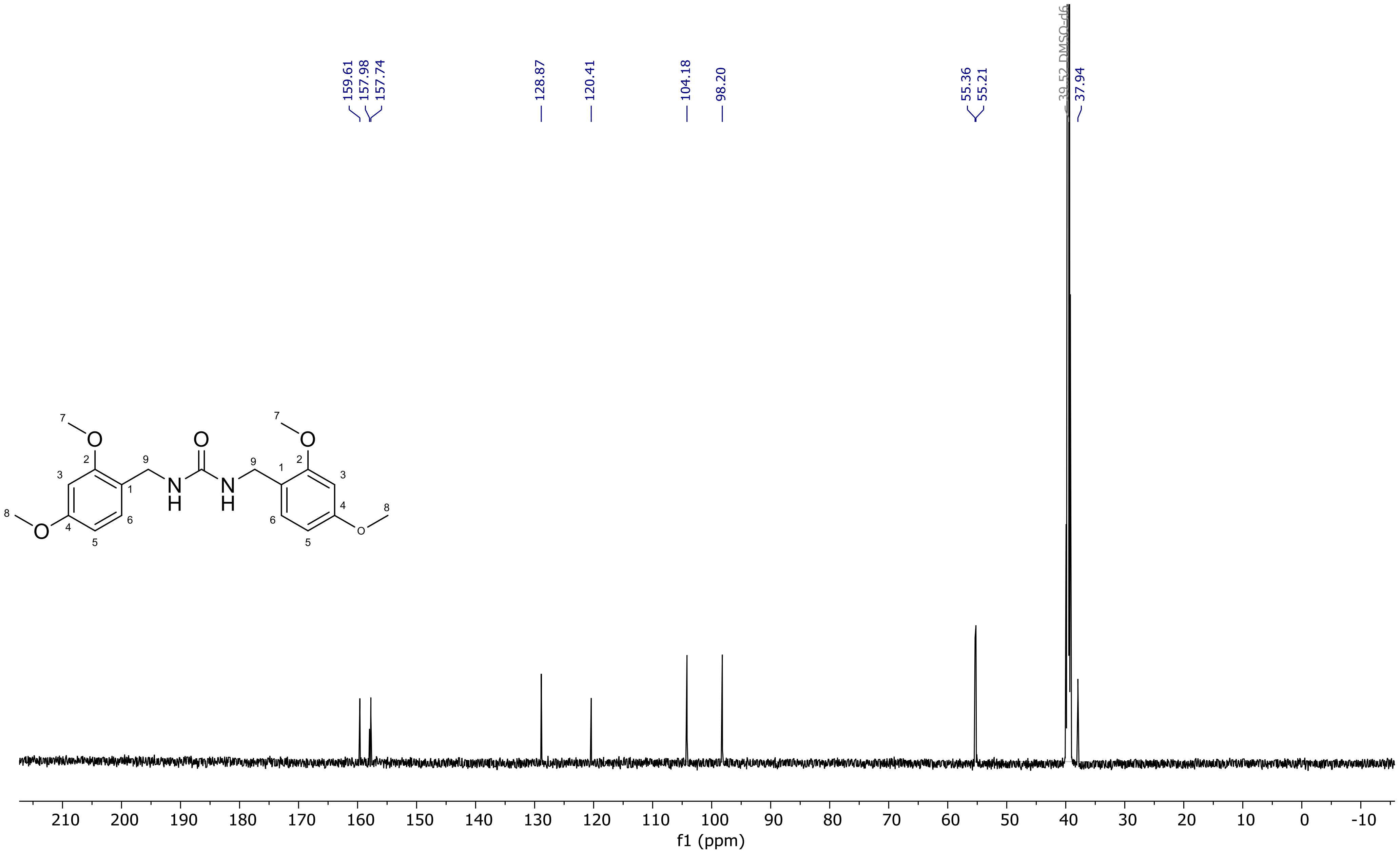

1-[(2,4-Dimethoxyphenyl)methyl]urea 14d, ^{1}H NMR in DMSO-d_6

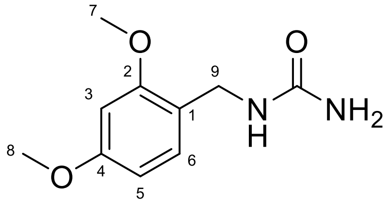

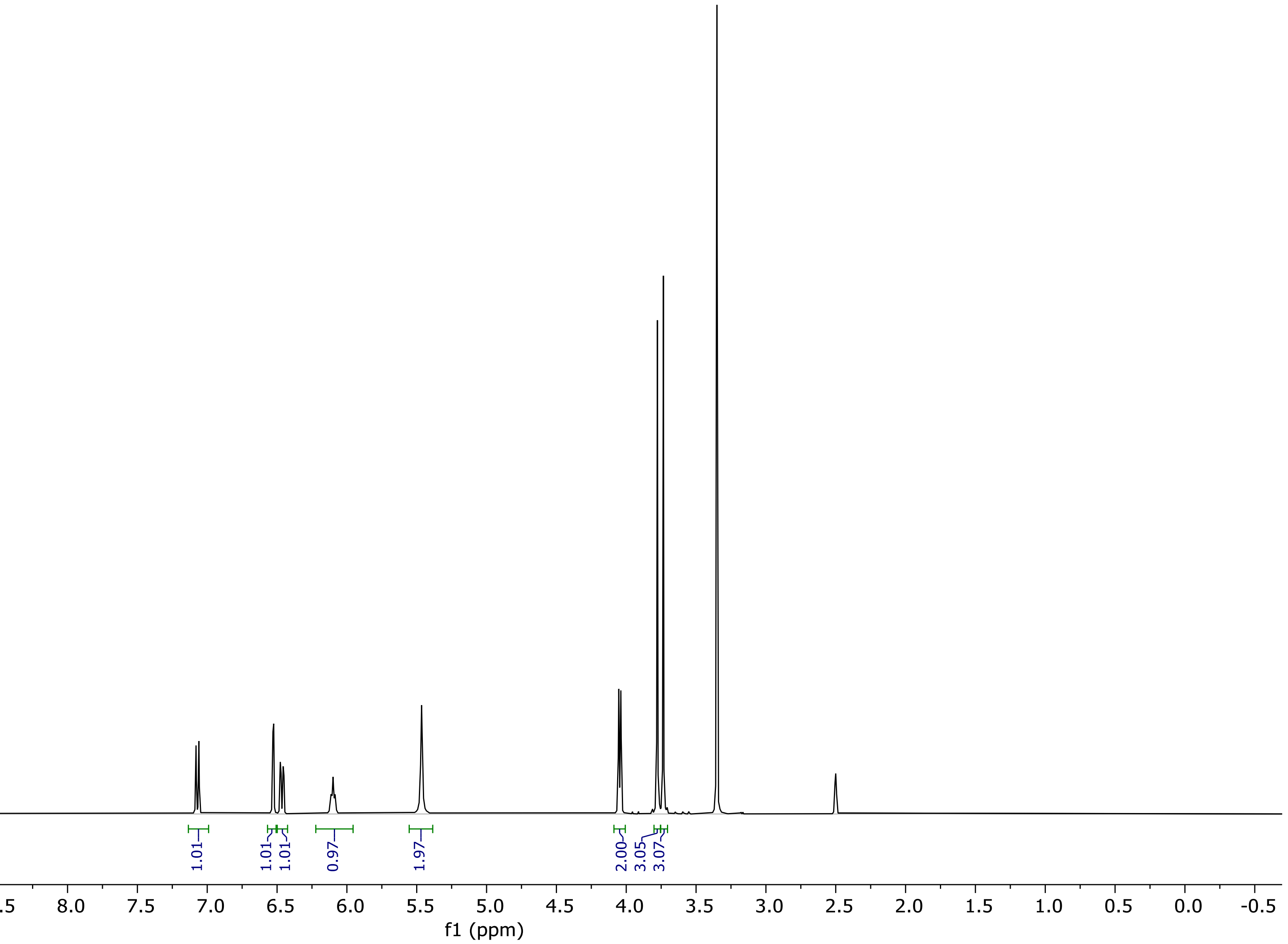

1-[(2,4-Dimethoxyphenyl)methyl]urea 14d, ^{13}C NMR in DMSO-d$_6$

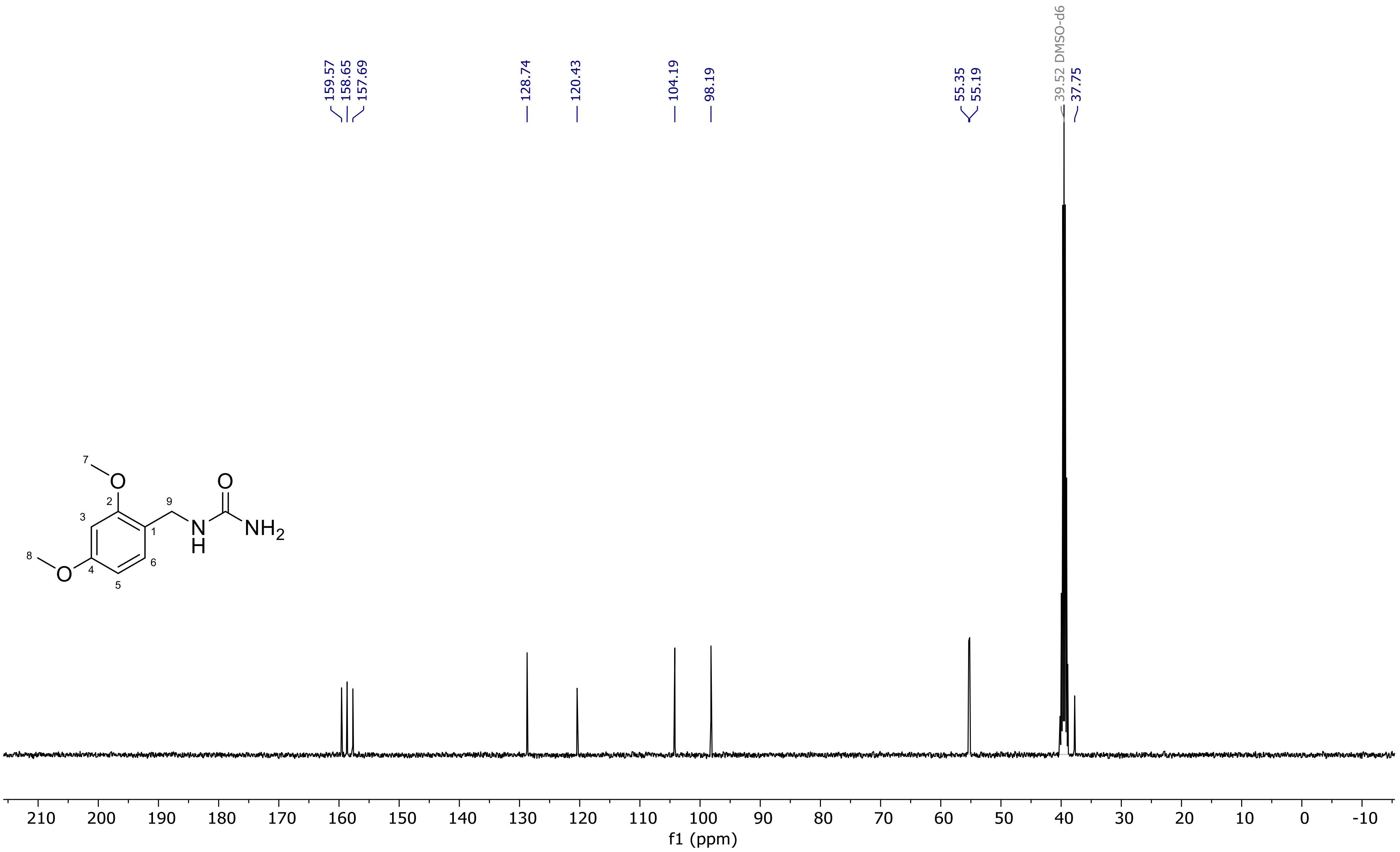

21. Aryl / alkyl glyoxal ^{1}H NMR and IR spectra

4-Methylphenylglyoxal 13b, ^{1}H NMR in CD_3CN

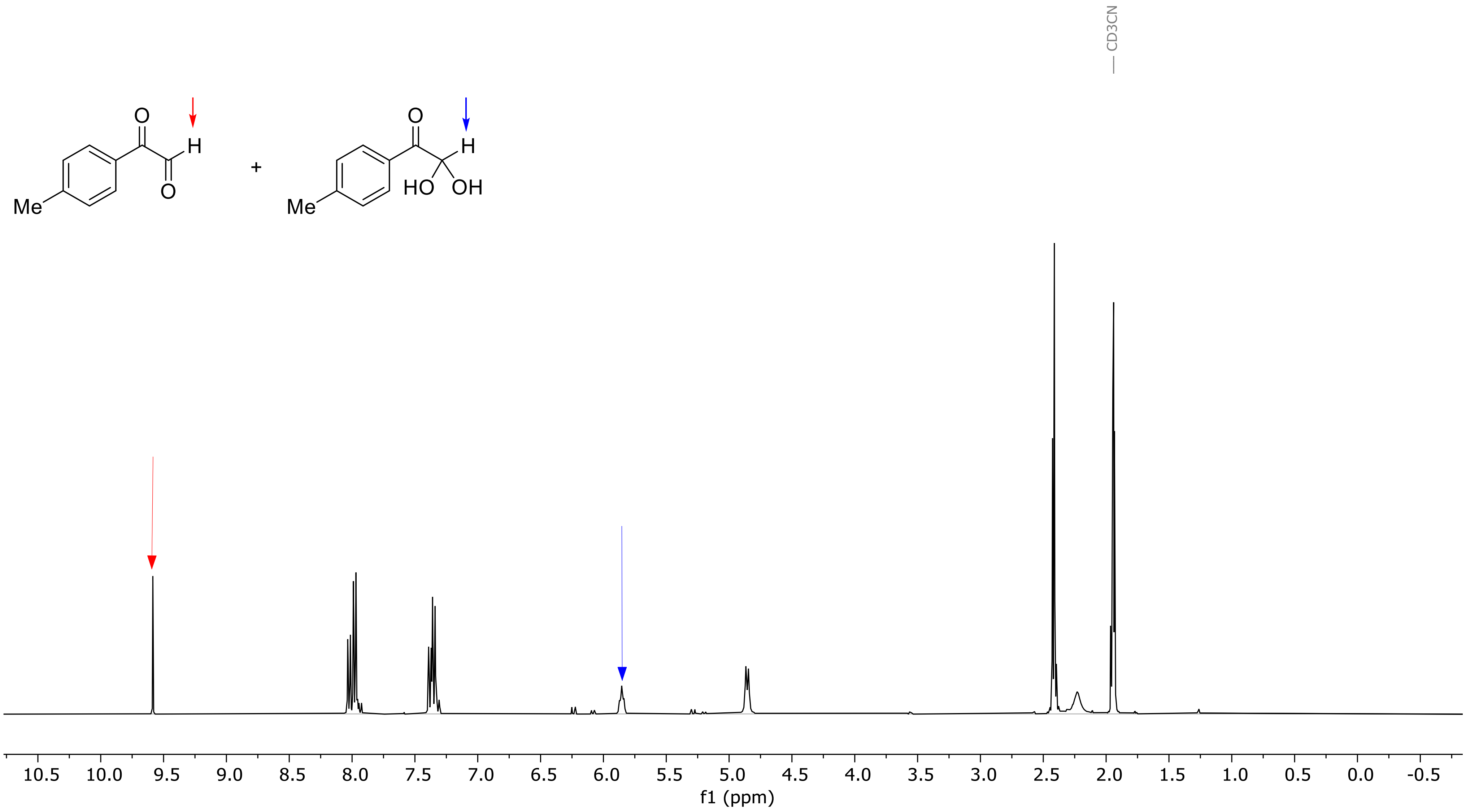

4-Methylphenylglyoxal 13b, IR Spectrum

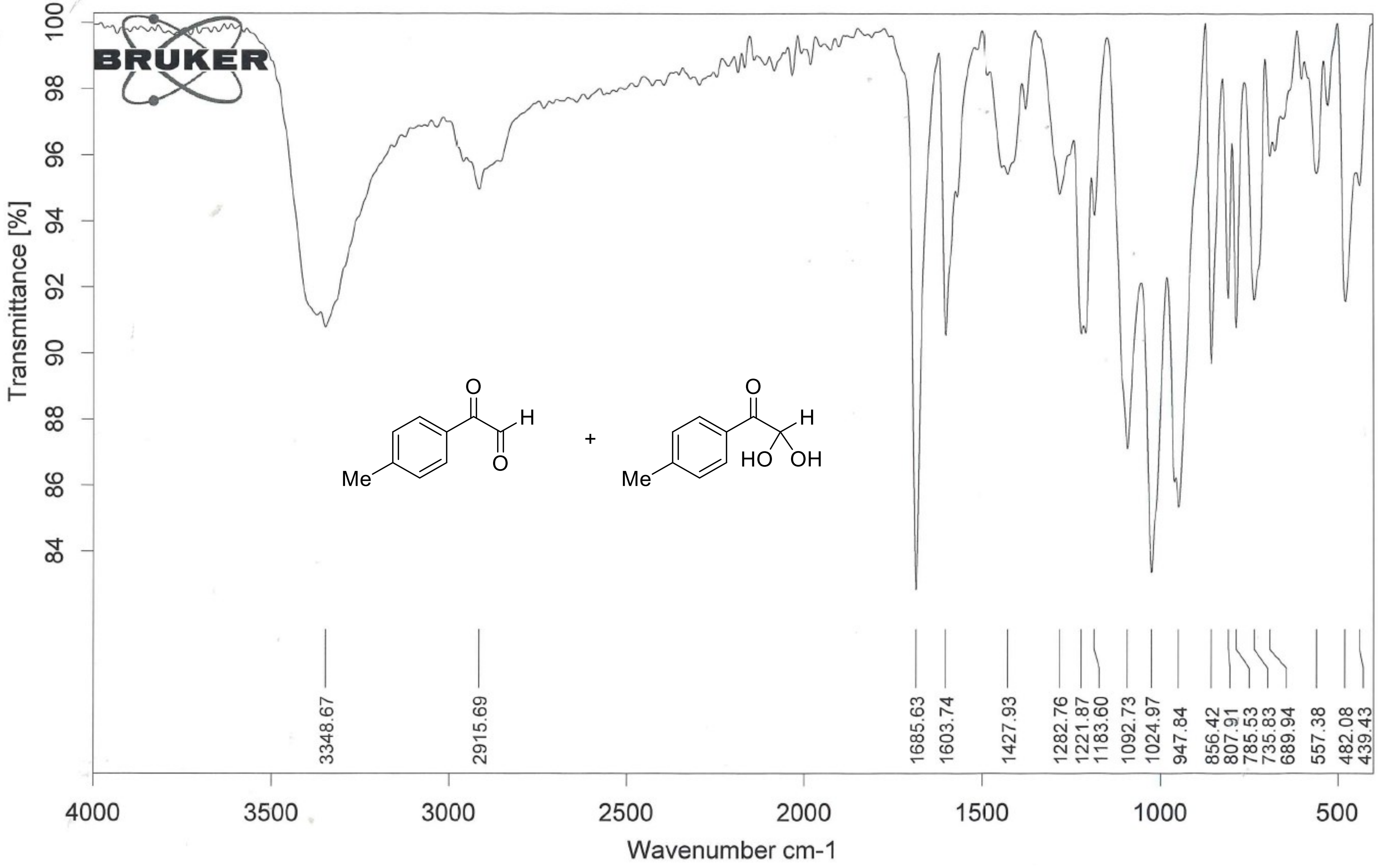

4-Hydroxyphenylglyoxal 13c, ^{1}H NMR in CD_3CN

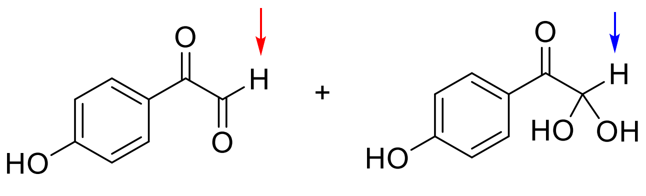

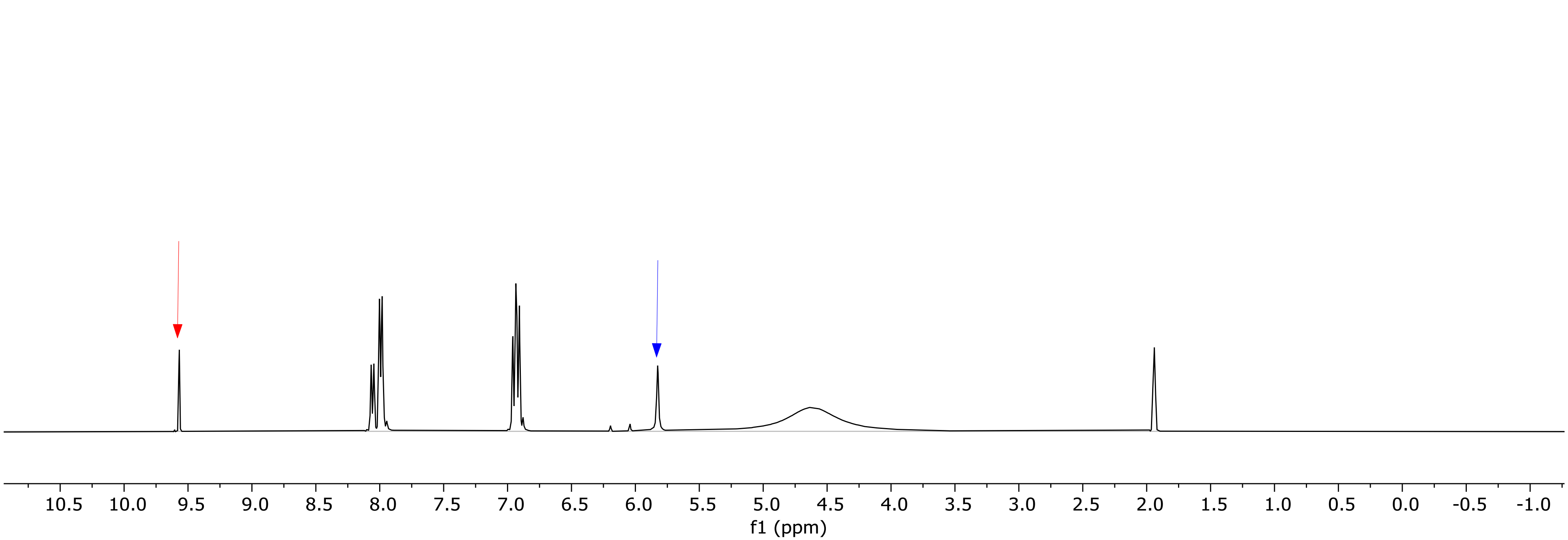

4-Hydroxyphenylglyoxal, 13c, IR Spectrum

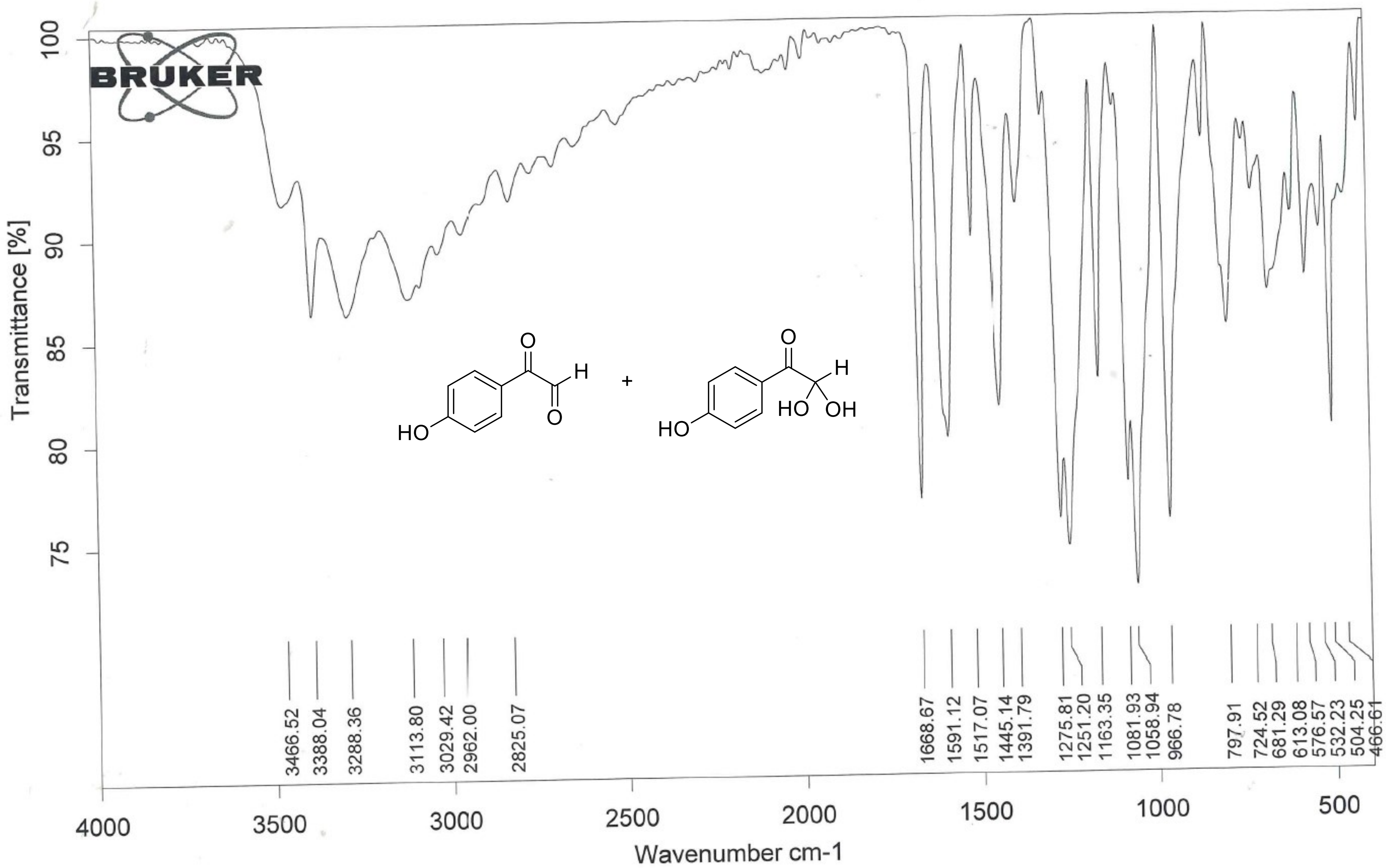

4-Chlorophenylglyoxal 13g, ^{1}H NMR in CD_3CN

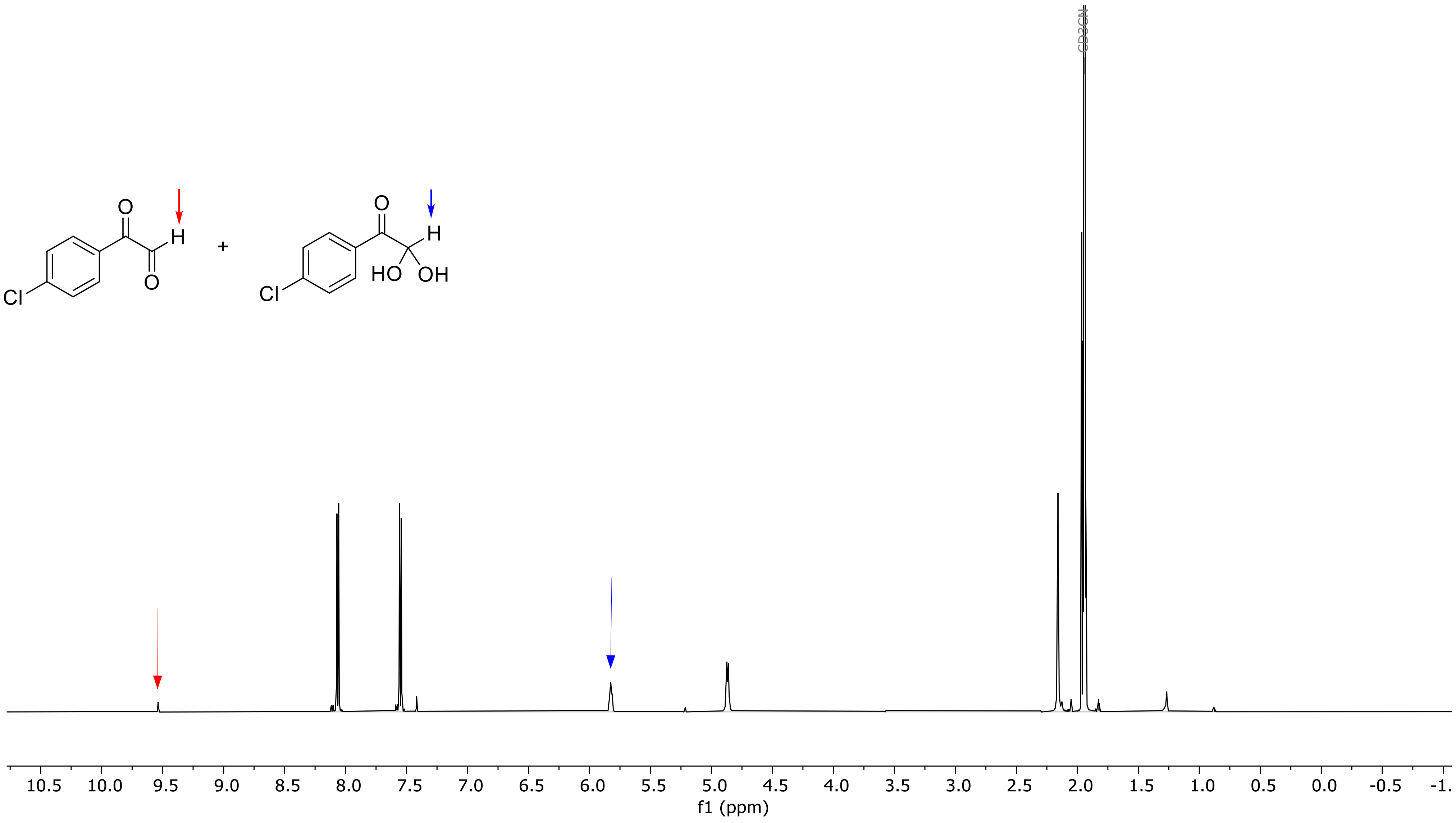

4-Chlorophenylglyoxal 13g, IR Spectrum

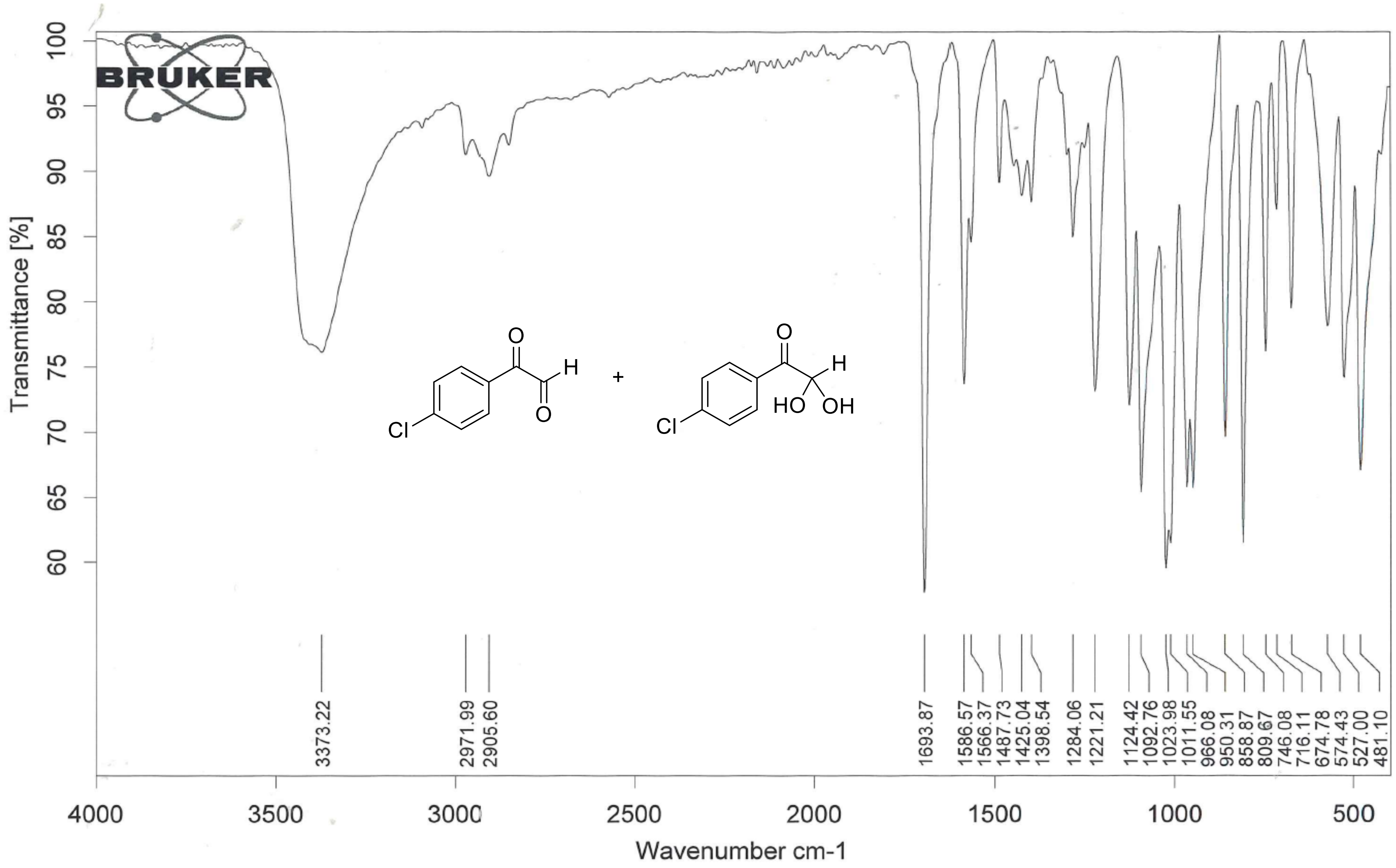

4-Iodophenylglyoxal 13i, ^{1}H NMR in CD_3CN

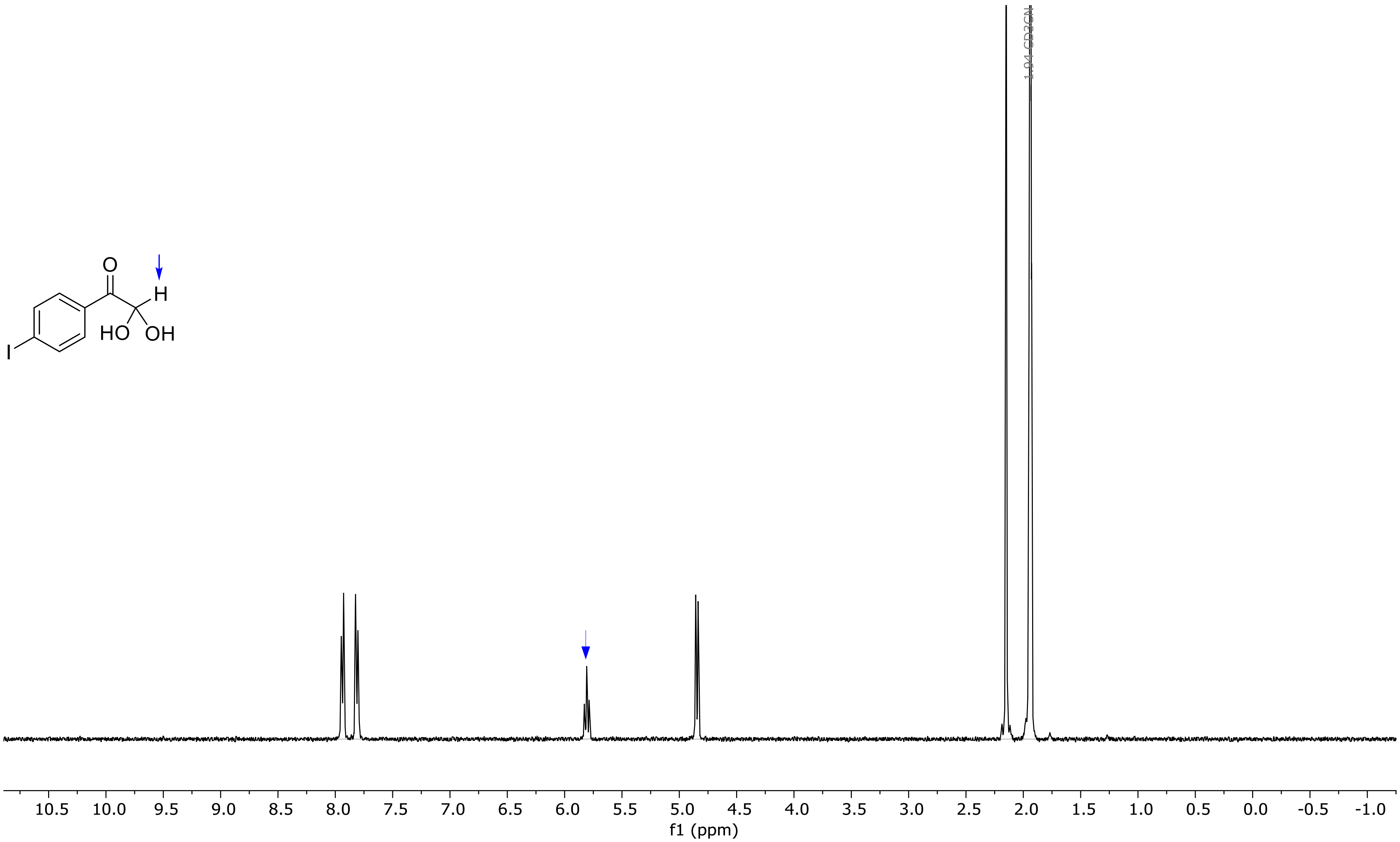

4-Iodophenylglyoxal 13i, IR Spectrum

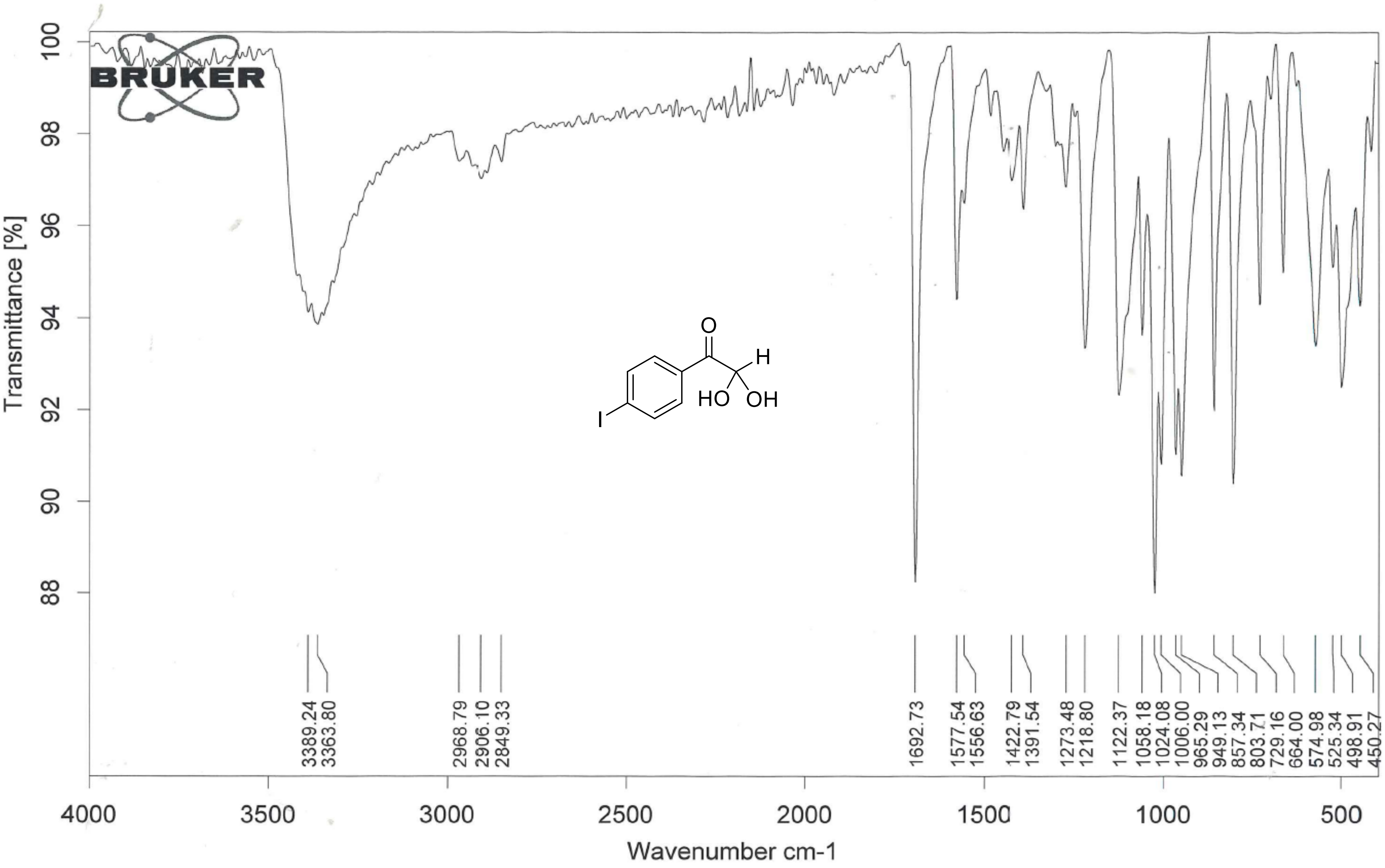

4-(Trifluoromethyl)phenylglyoxal 13j, ^{1}H NMR in CD_3CN

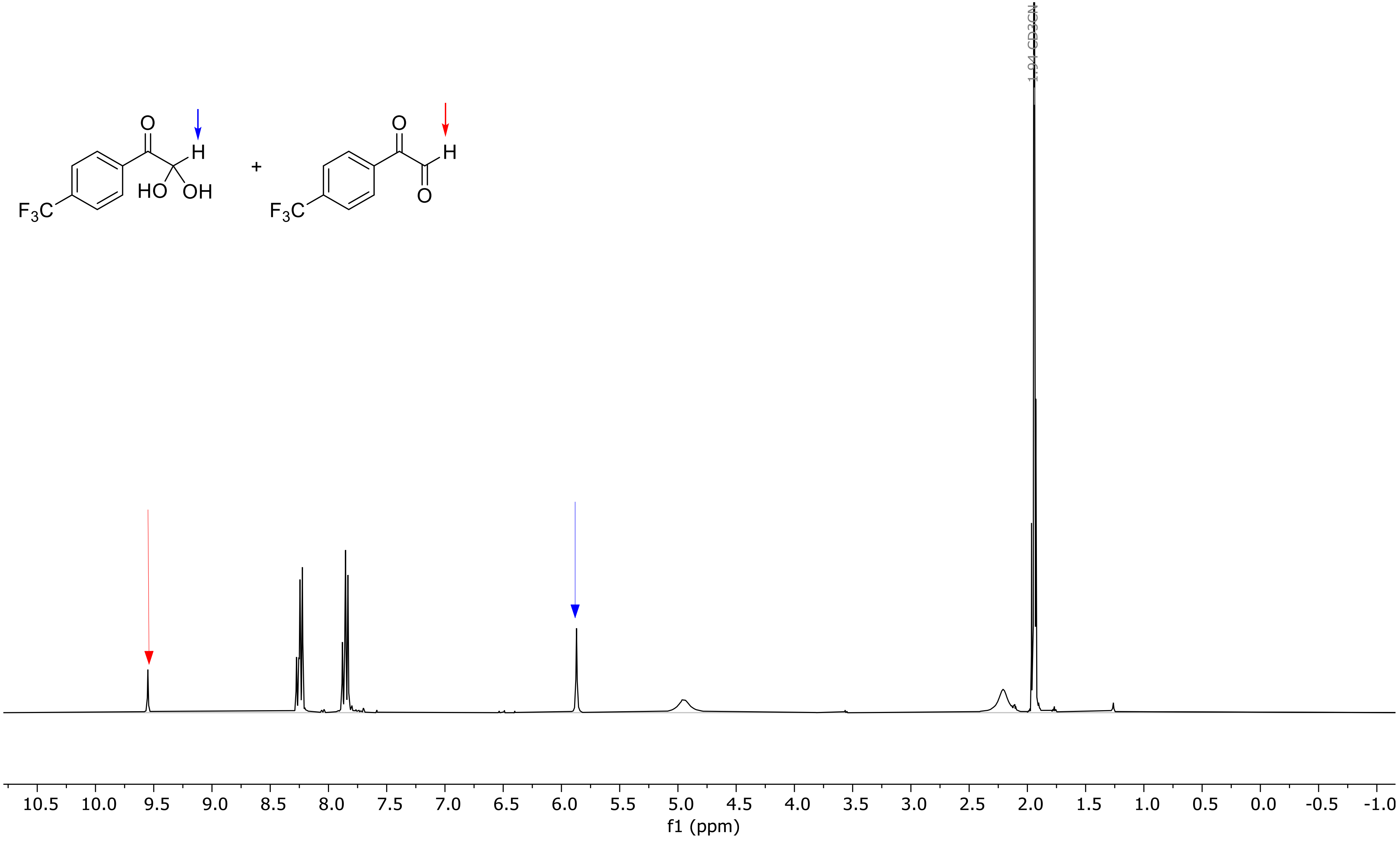

4-(Trifluoromethyl)phenylglyoxal 13j, IR Spectrum

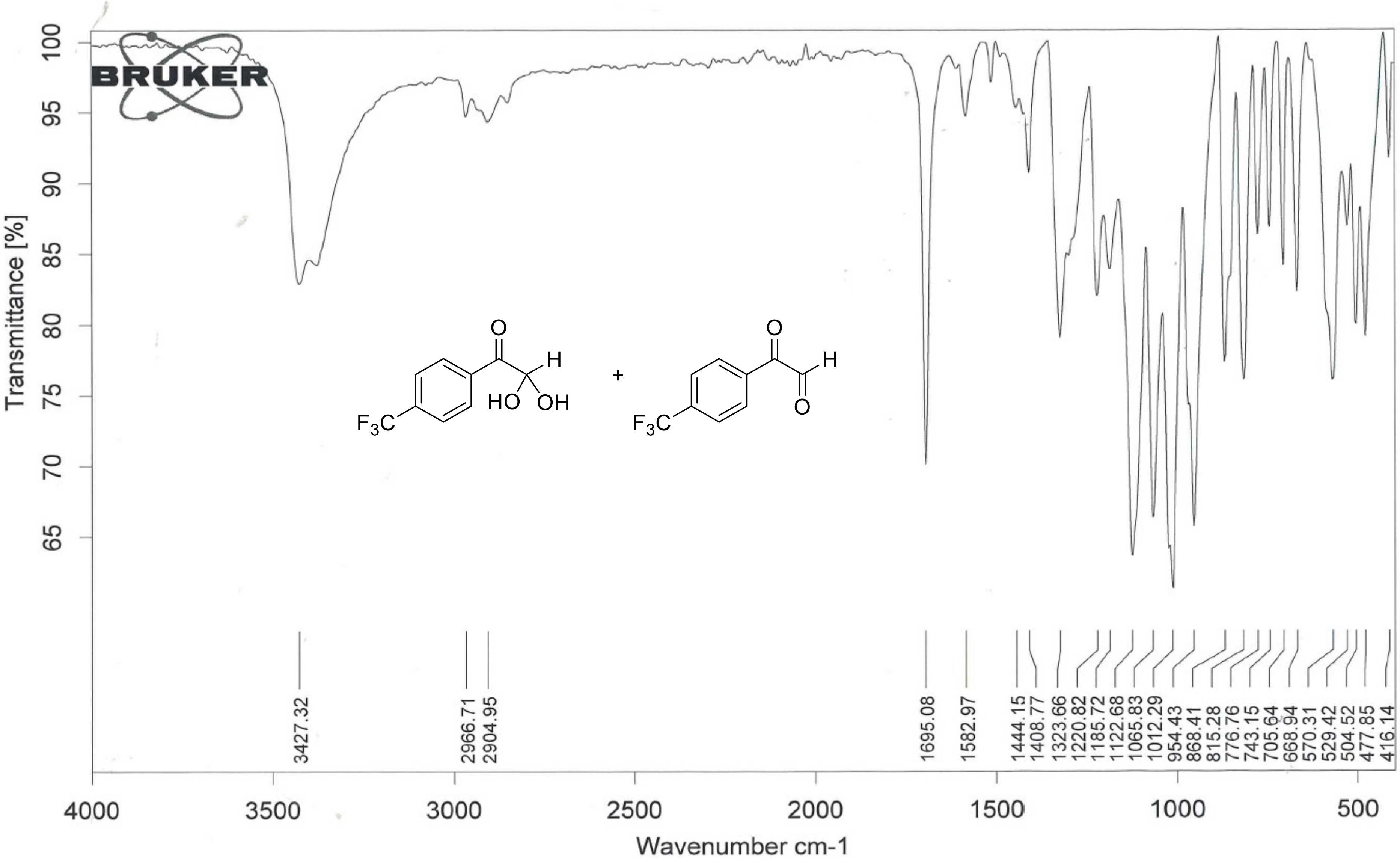

4-Nitrophenylglyoxal 13k, ^{1}H NMR in CD_3CN

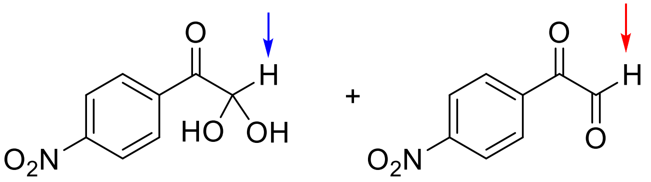

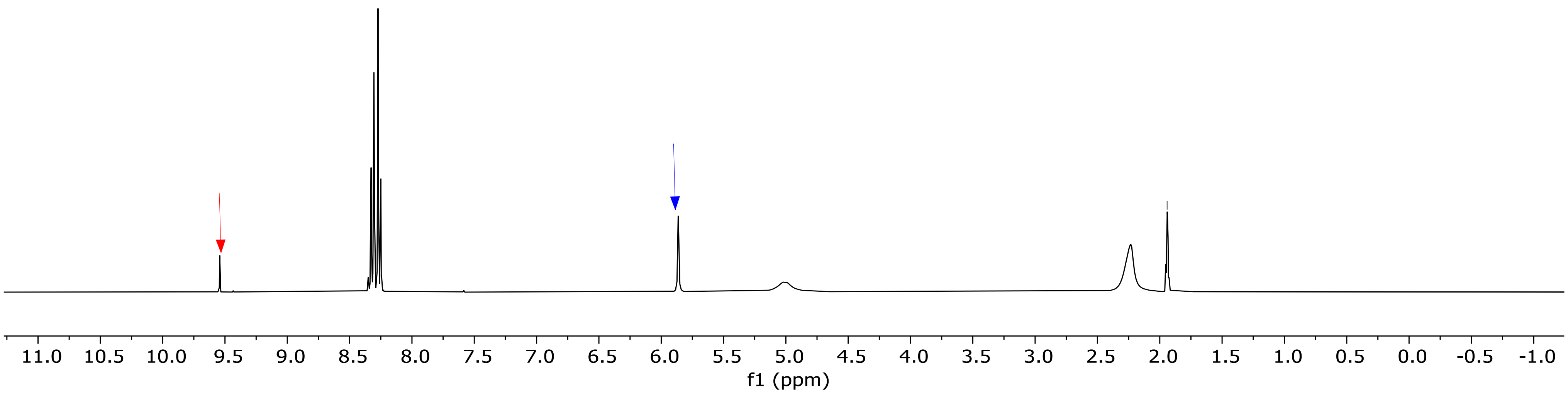

4-Nitrophenylglyoxal 13k, IR Spectrum

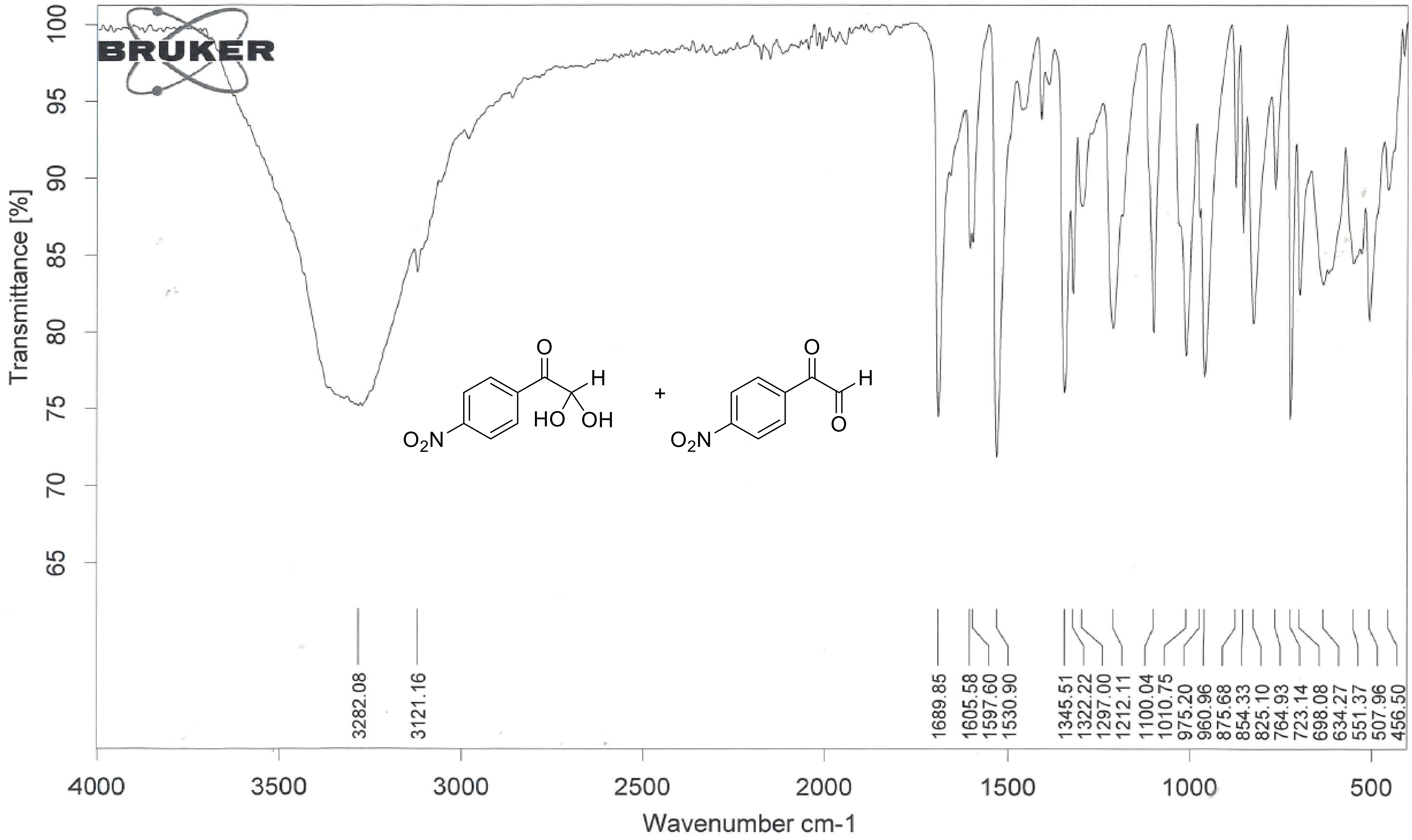

3-Methoxyphenylglyoxal hemihydrate 13l, ^1H NMR in CD_3CN

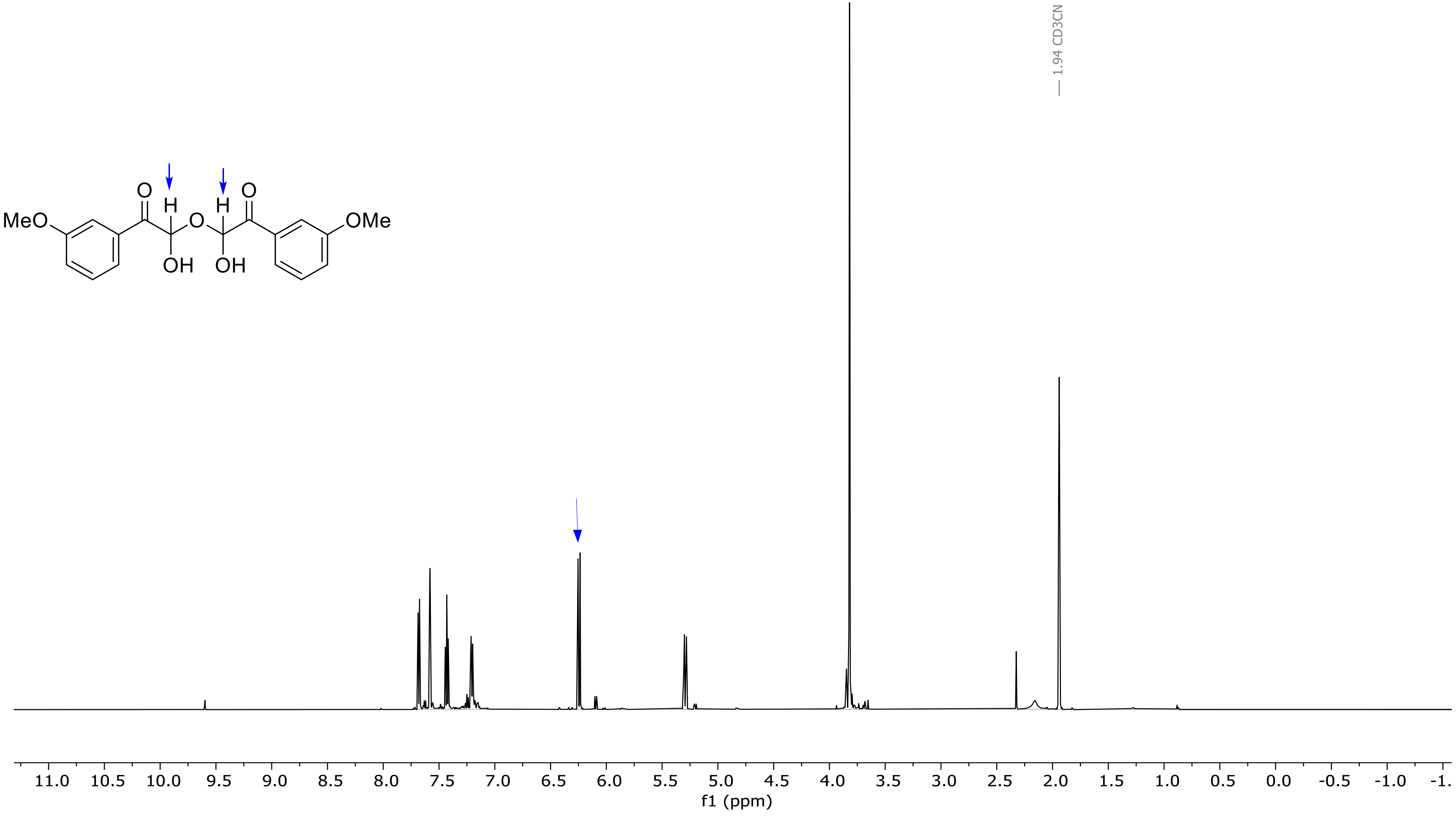

3-Methoxyphenylglyoxal hemihydrate 13l, IR Spectrum

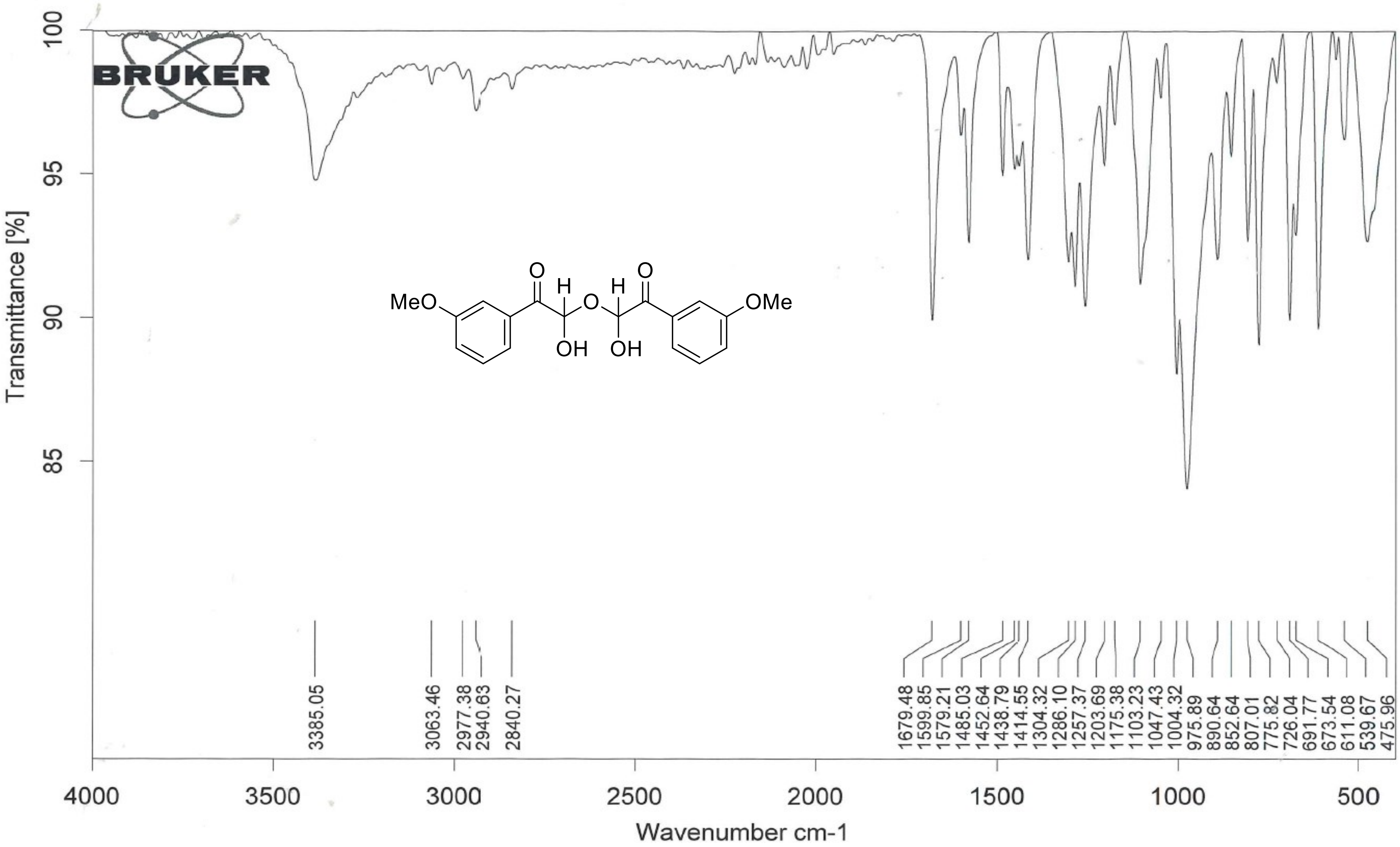

3-Chlorophenylglyoxal hemihydrate 13m, ^{1}H NMR in CD_3CN

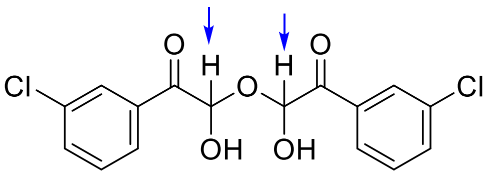

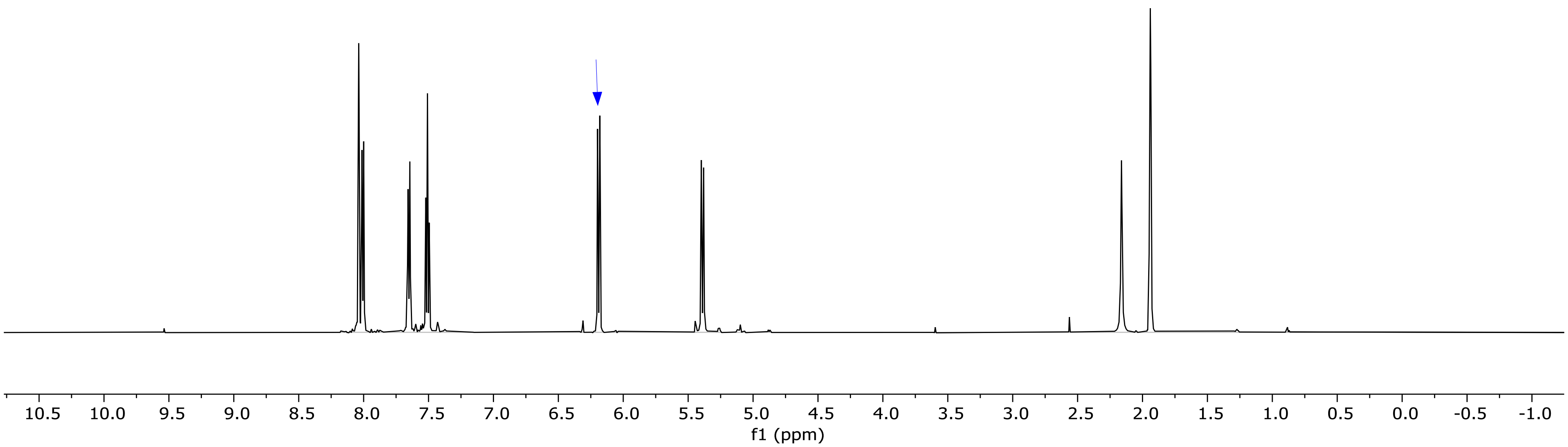

3-Chlorophenylglyoxal hemihydrate 13m, IR Spectrum

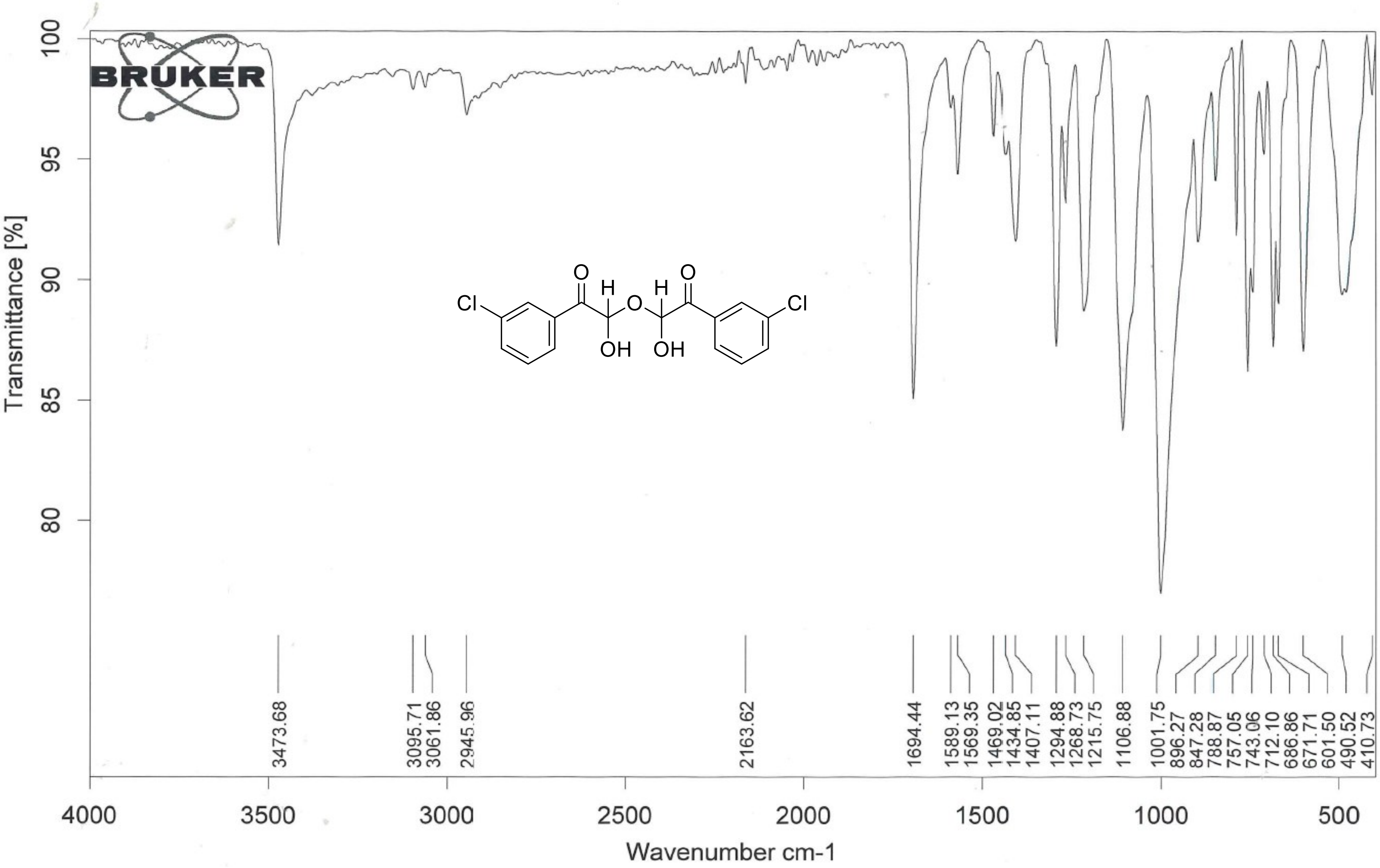

2-Methoxyphenylglyoxal 13o, IR Spectrum

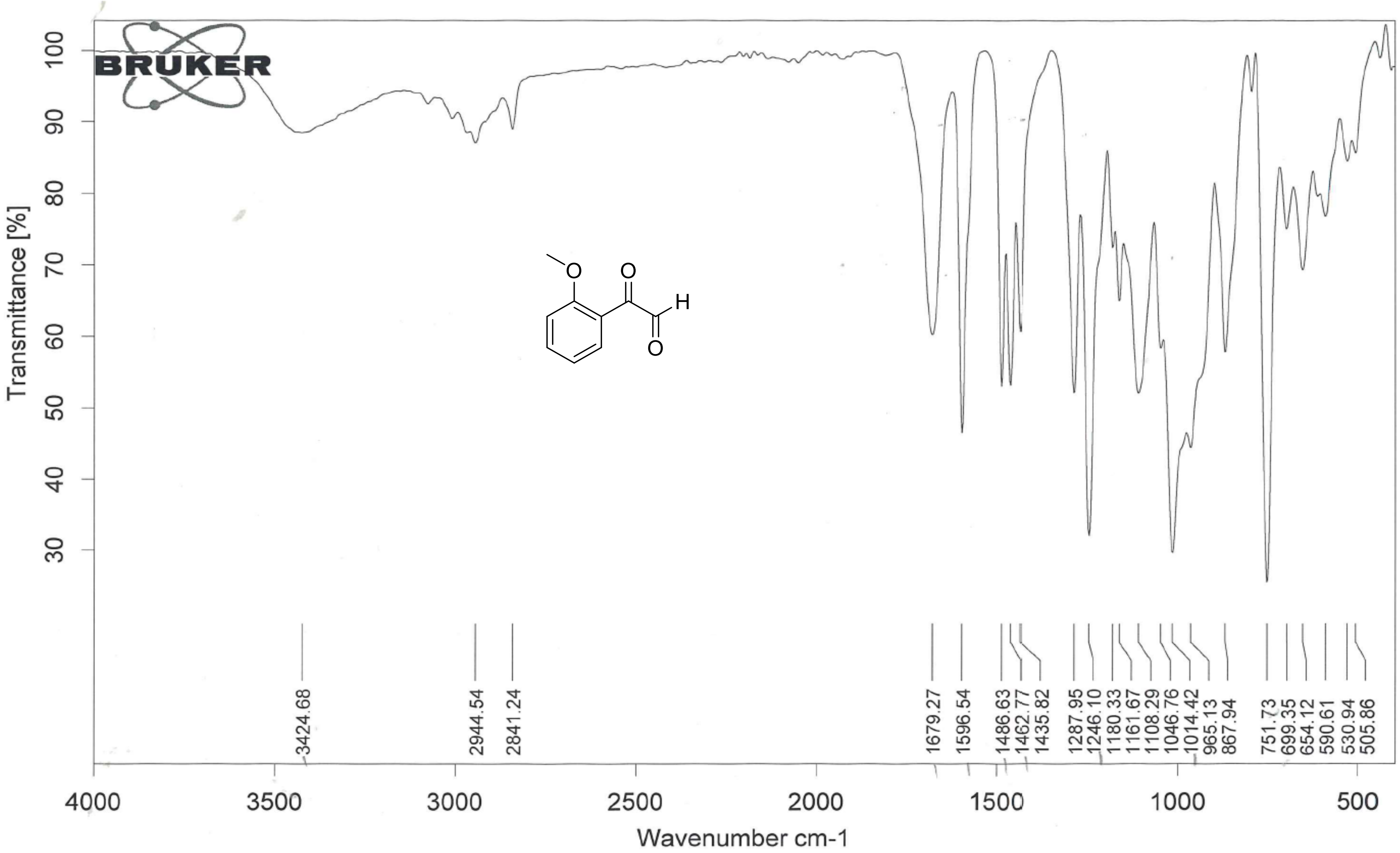

2-Chlorophenylglyoxal 13p, IR Spectrum

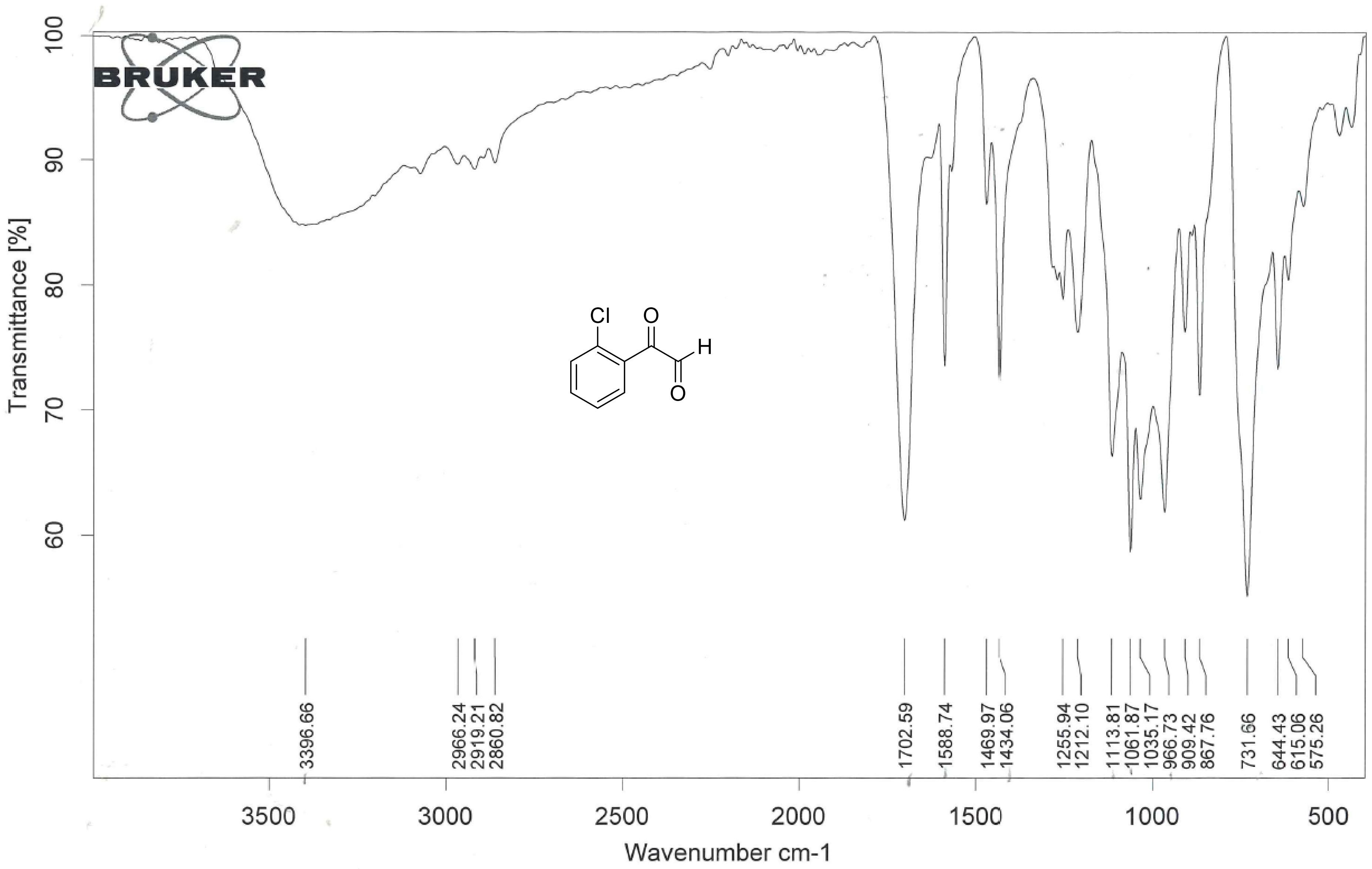

2-(Trifluoromethyl)phenylglyoxal 13q, IR Spectrum

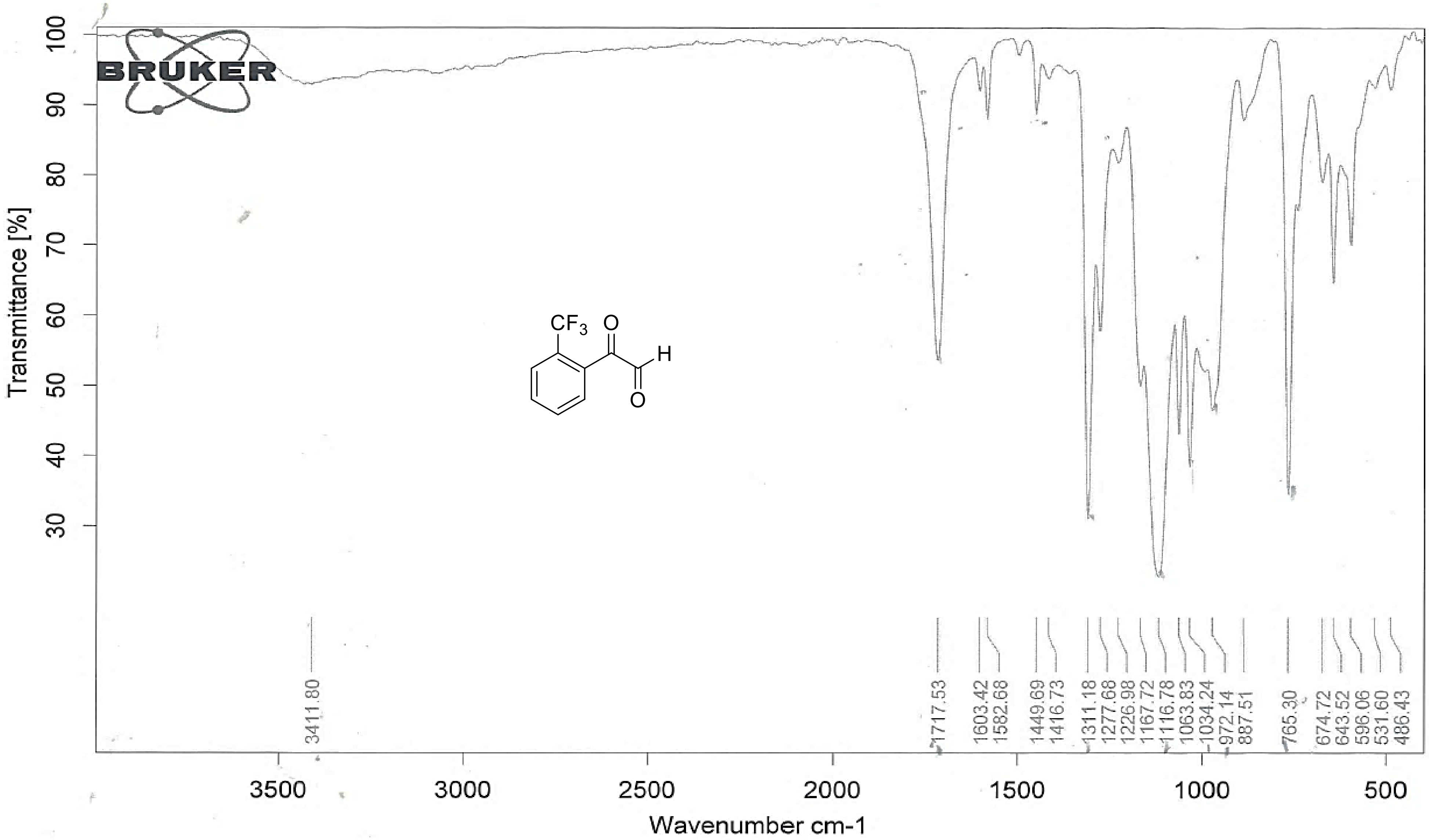

2-Naphthylglyoxal 13r, ^{1}H NMR in CD_3CN

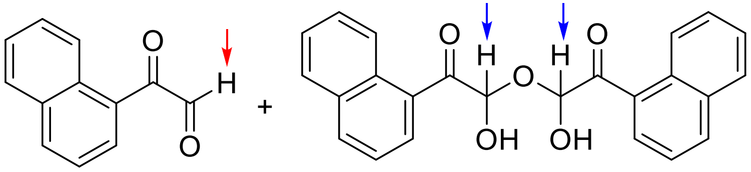

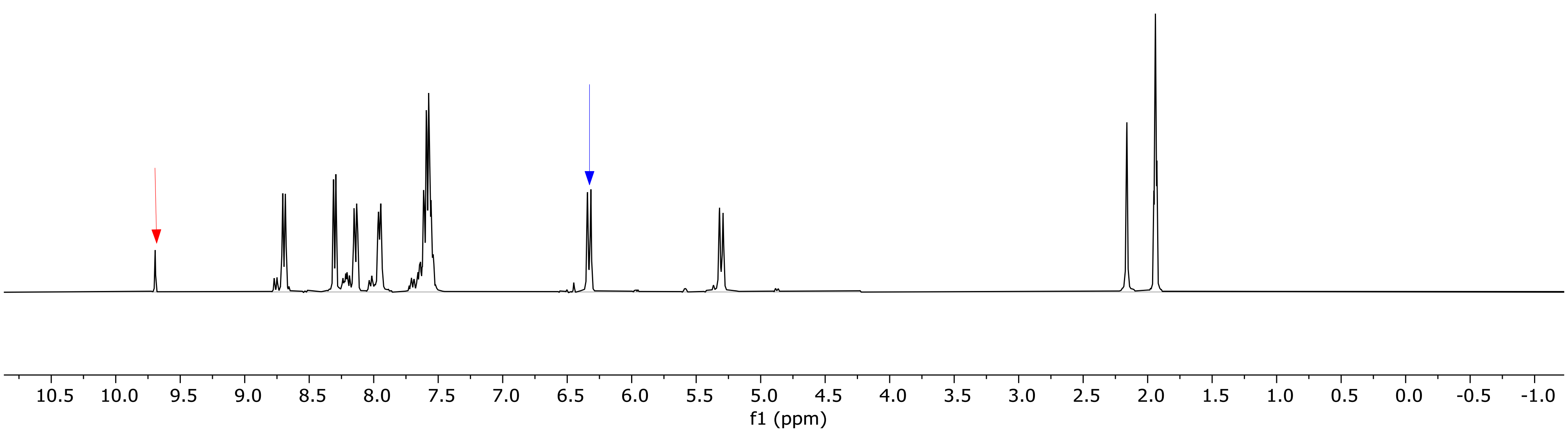

1- Naphthylglyoxal 13r, IR Spectrum

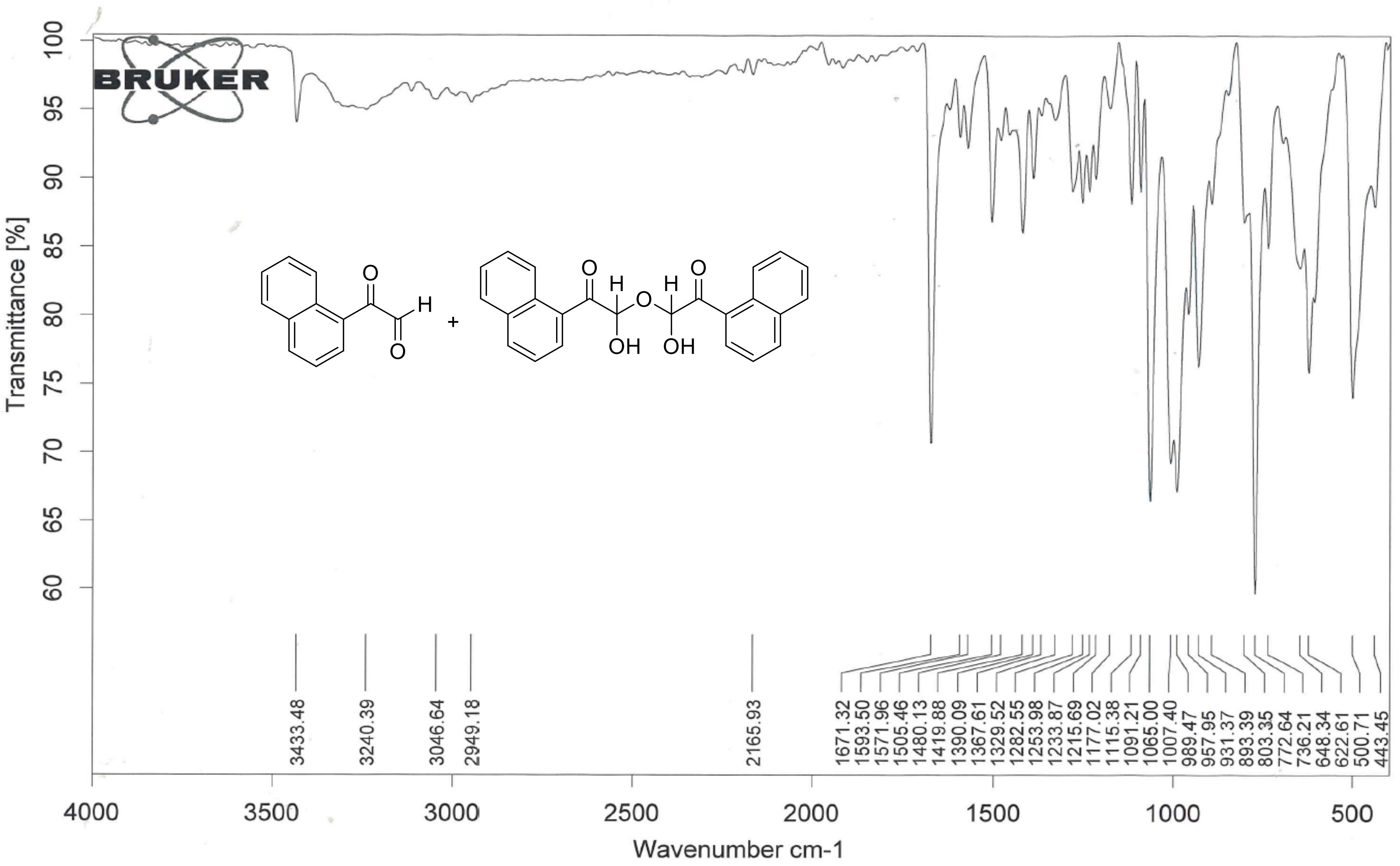

2- Naphthylglyoxal 13s, ^{1}H NMR in CD_3CN

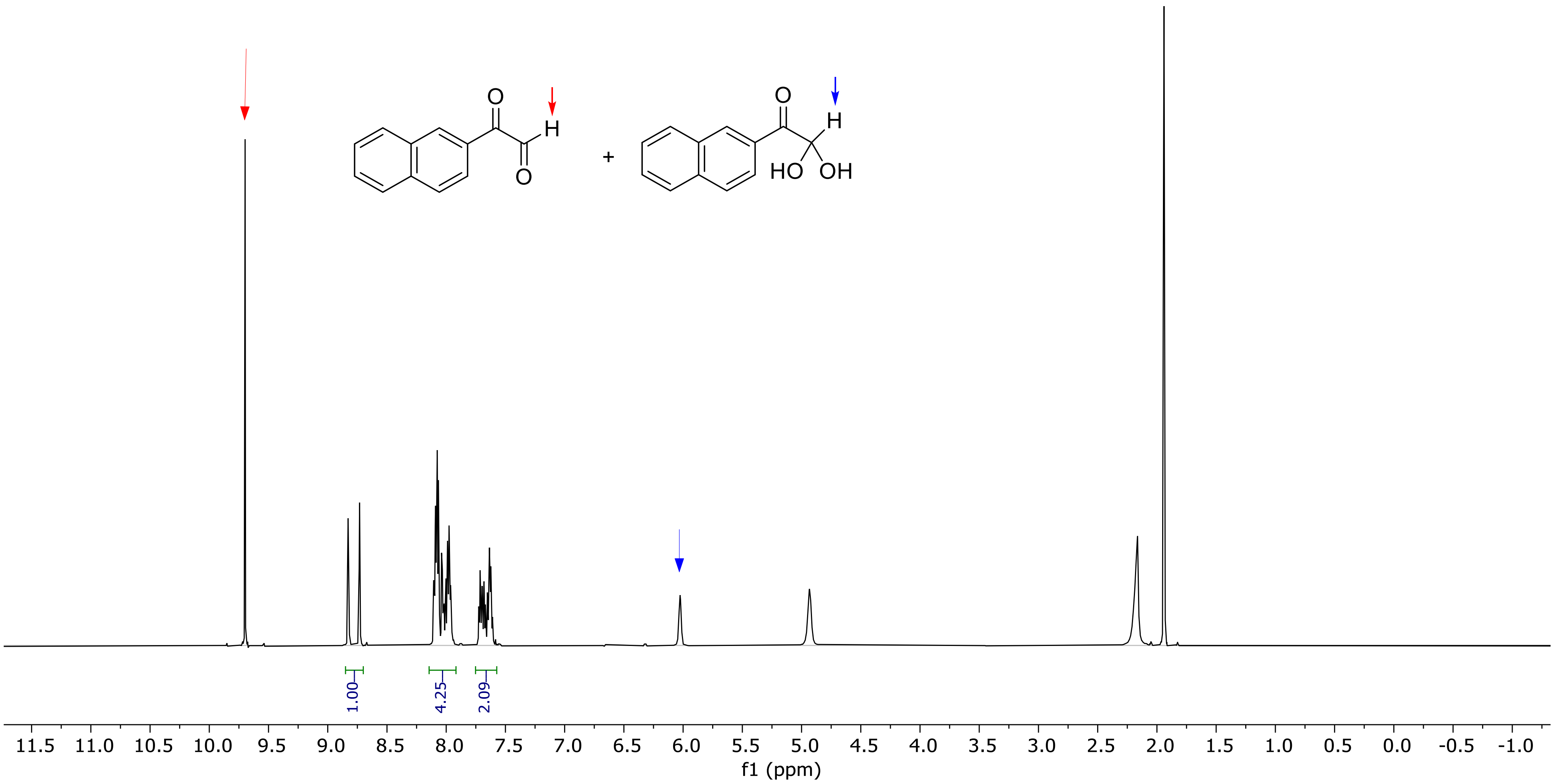

1-Naphthylglyoxal 13s, IR Spectrum

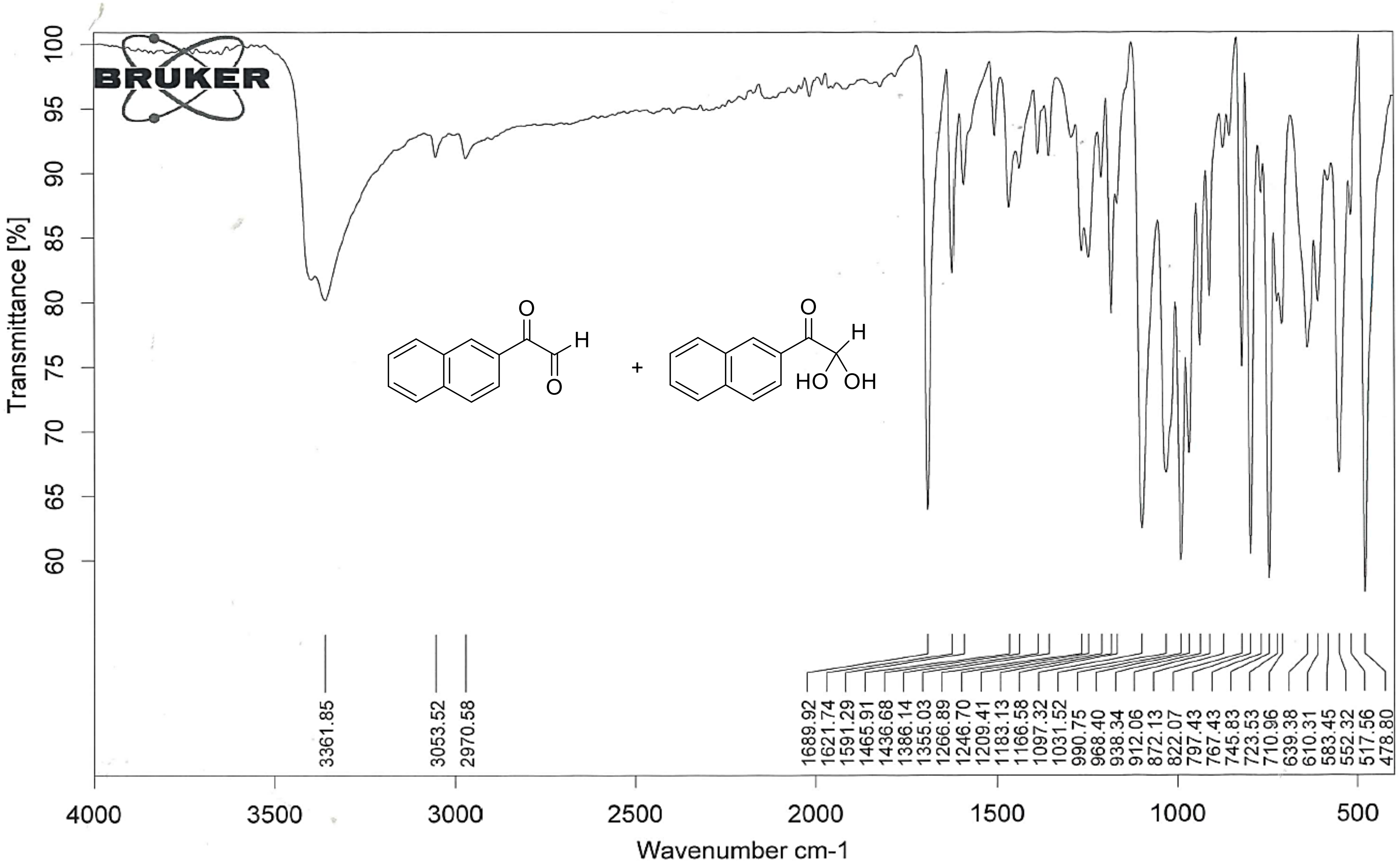

3,3-Dimethyl-2-oxobutanal S13y, IR Spectrum

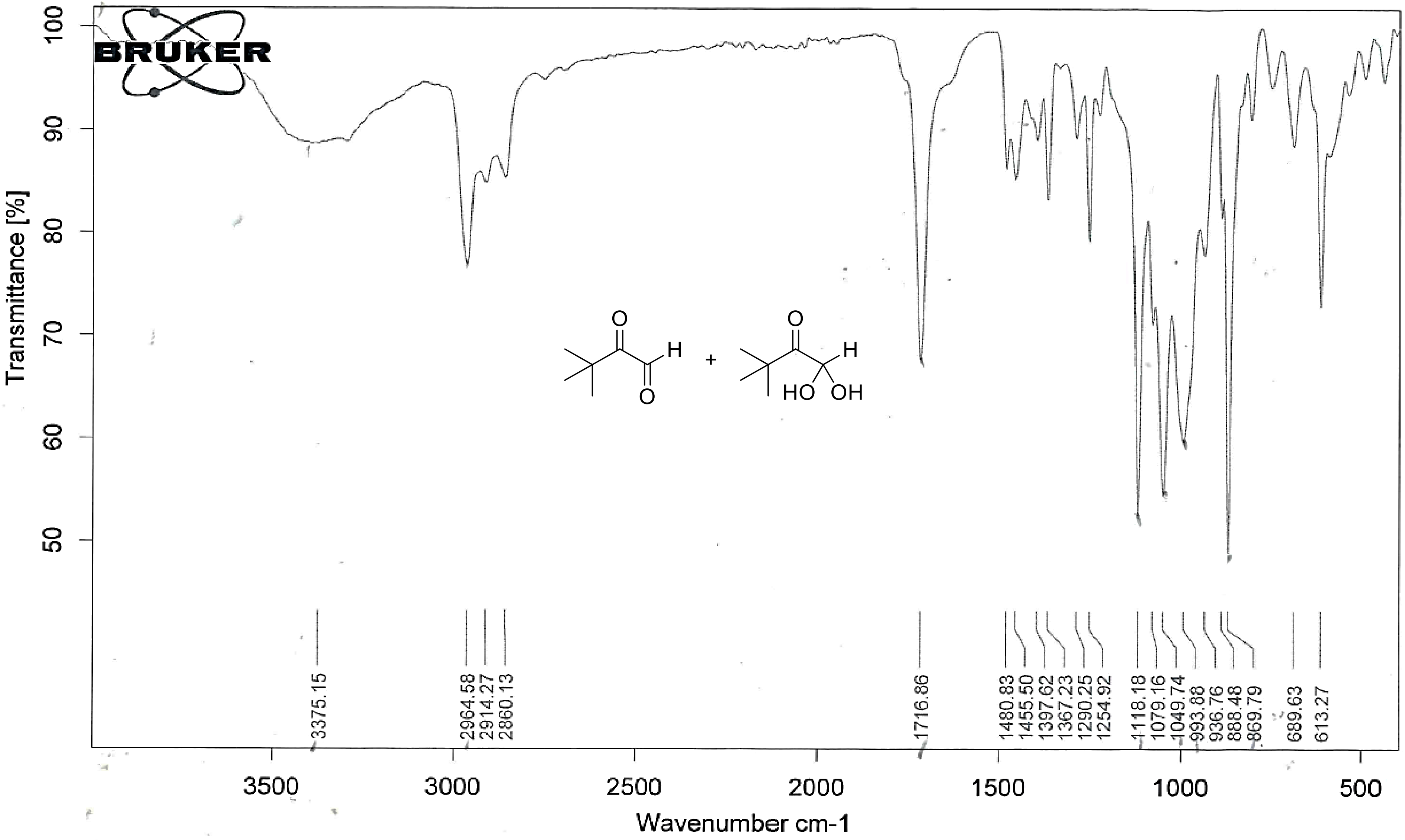

2-(Adamantan-1-yl)-2-oxoacetaldehyde S13z, IR Spectrum

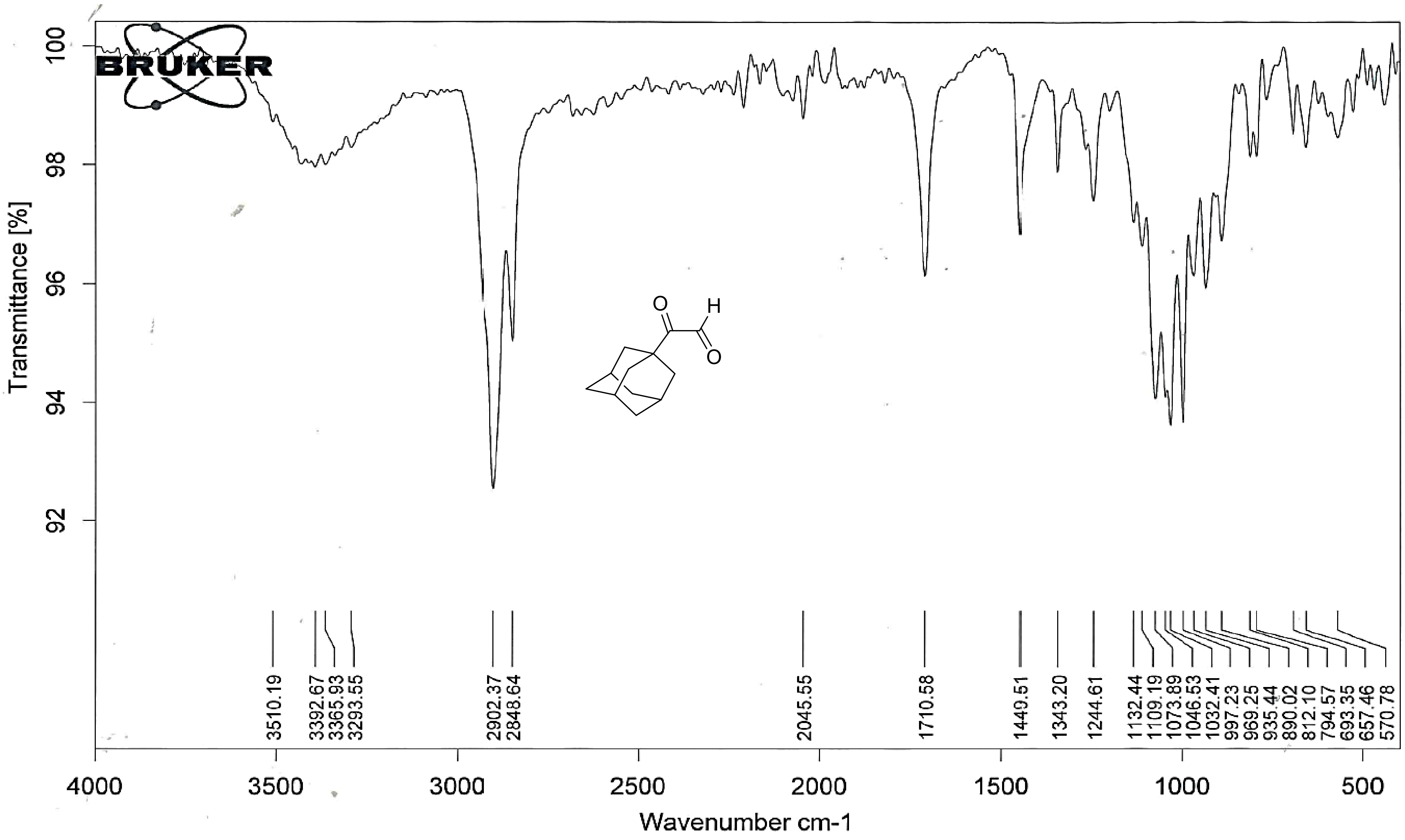

2-Cyclohexyl-2-oxoacetaldehyde. hemihydrate, S13aa ^{1}H NMR in $CDCl_3$ (*carried forward to the next step, without further purification*)

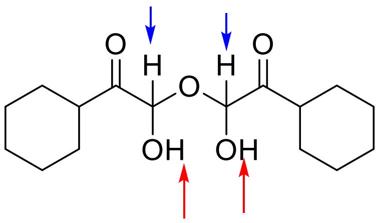

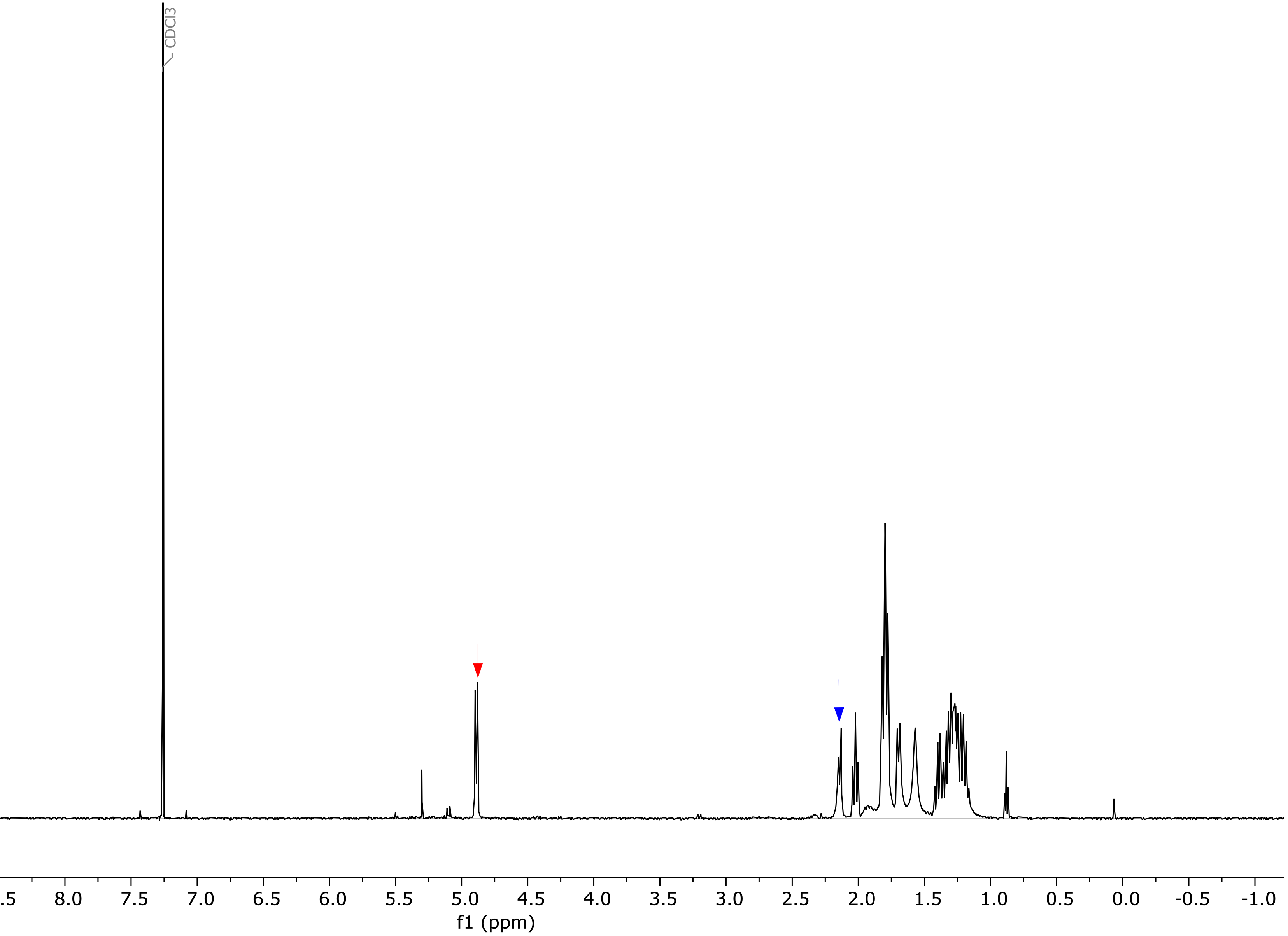

2-Cyclohexyl-2-oxoacetaldehyde hemihydrate S13aa, IR Spectrum

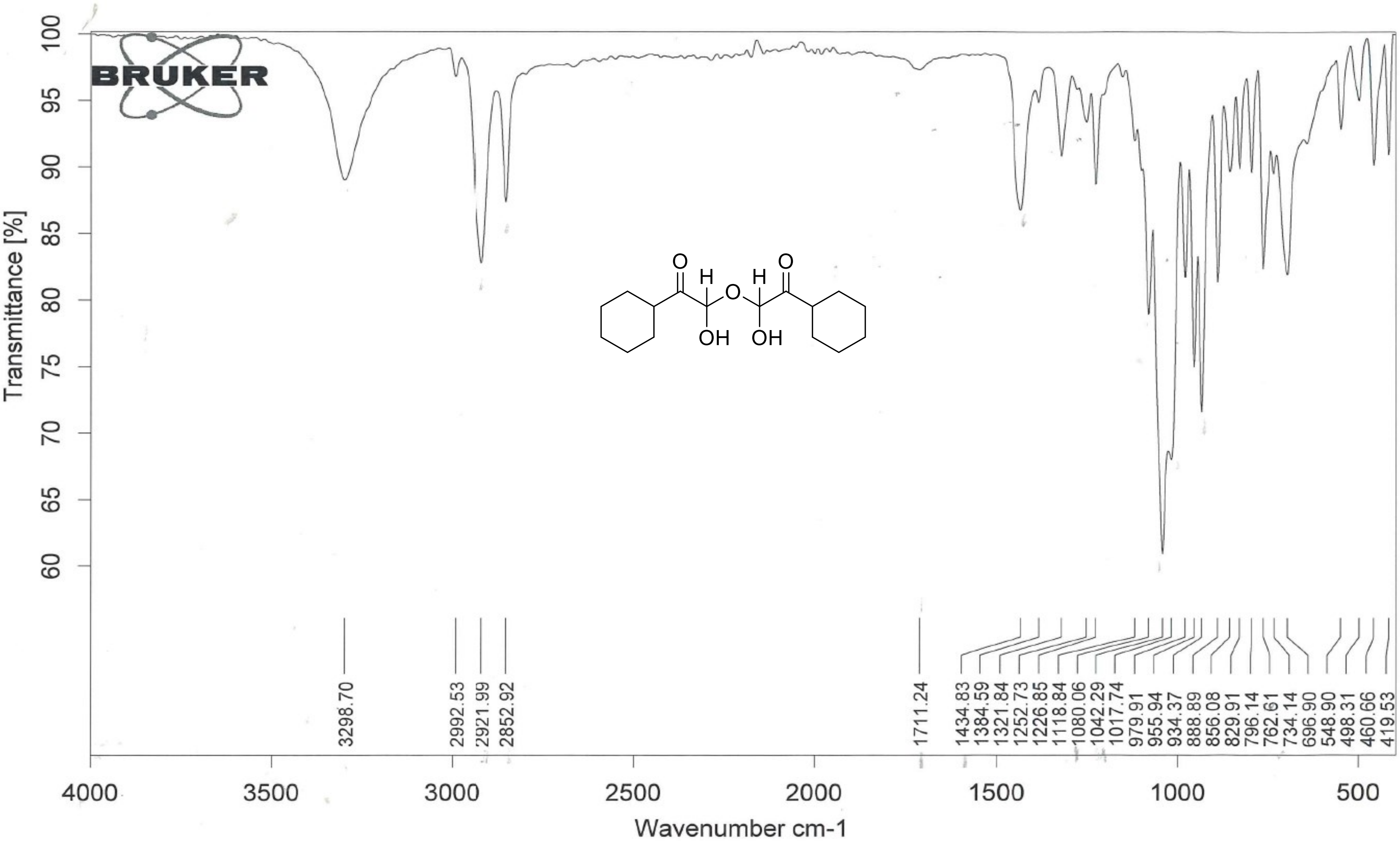

2-Cyclopropyl-2-oxoacetaldehyde 13ab, ^{1}H NMR in CDCl$_3$ (*carried forward to the next step, without further purification*)

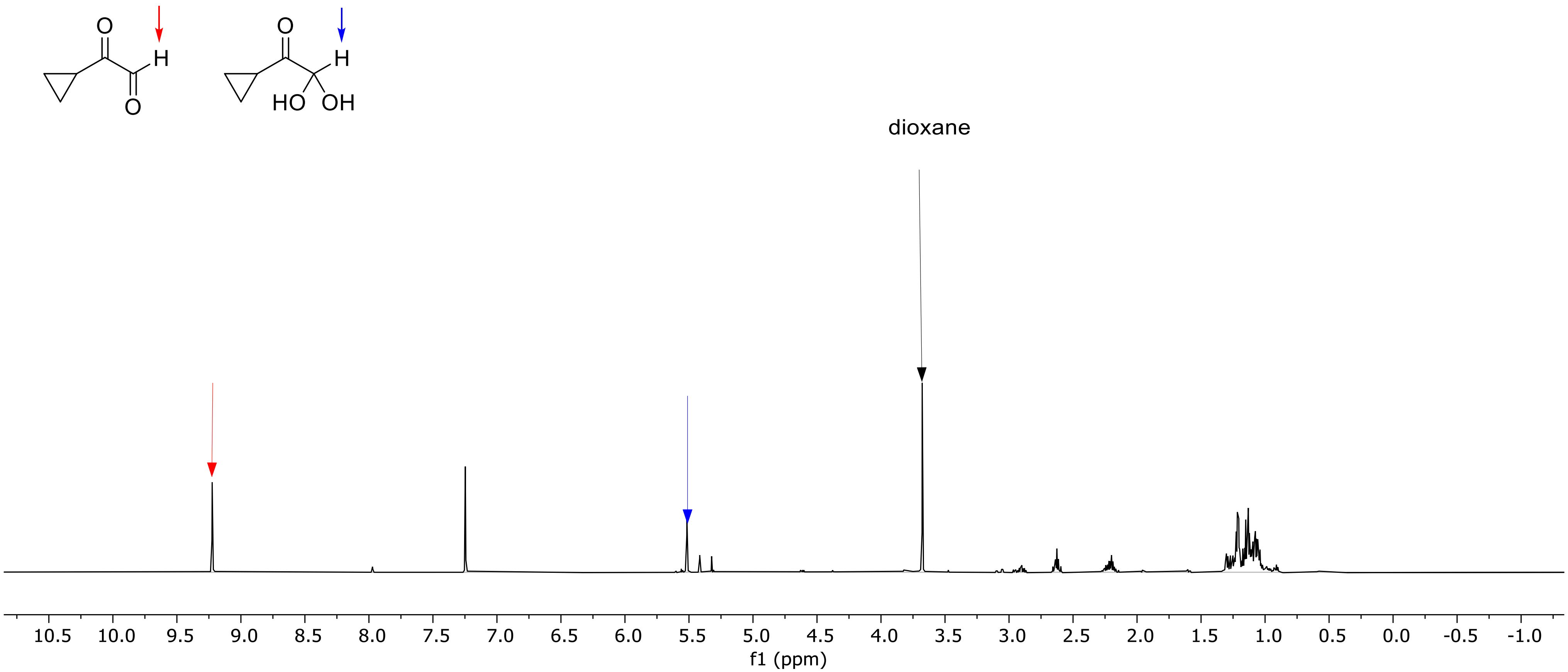

2-Cyclopropyl-2-oxoacetaldehyde 13ab, IR Spectrum

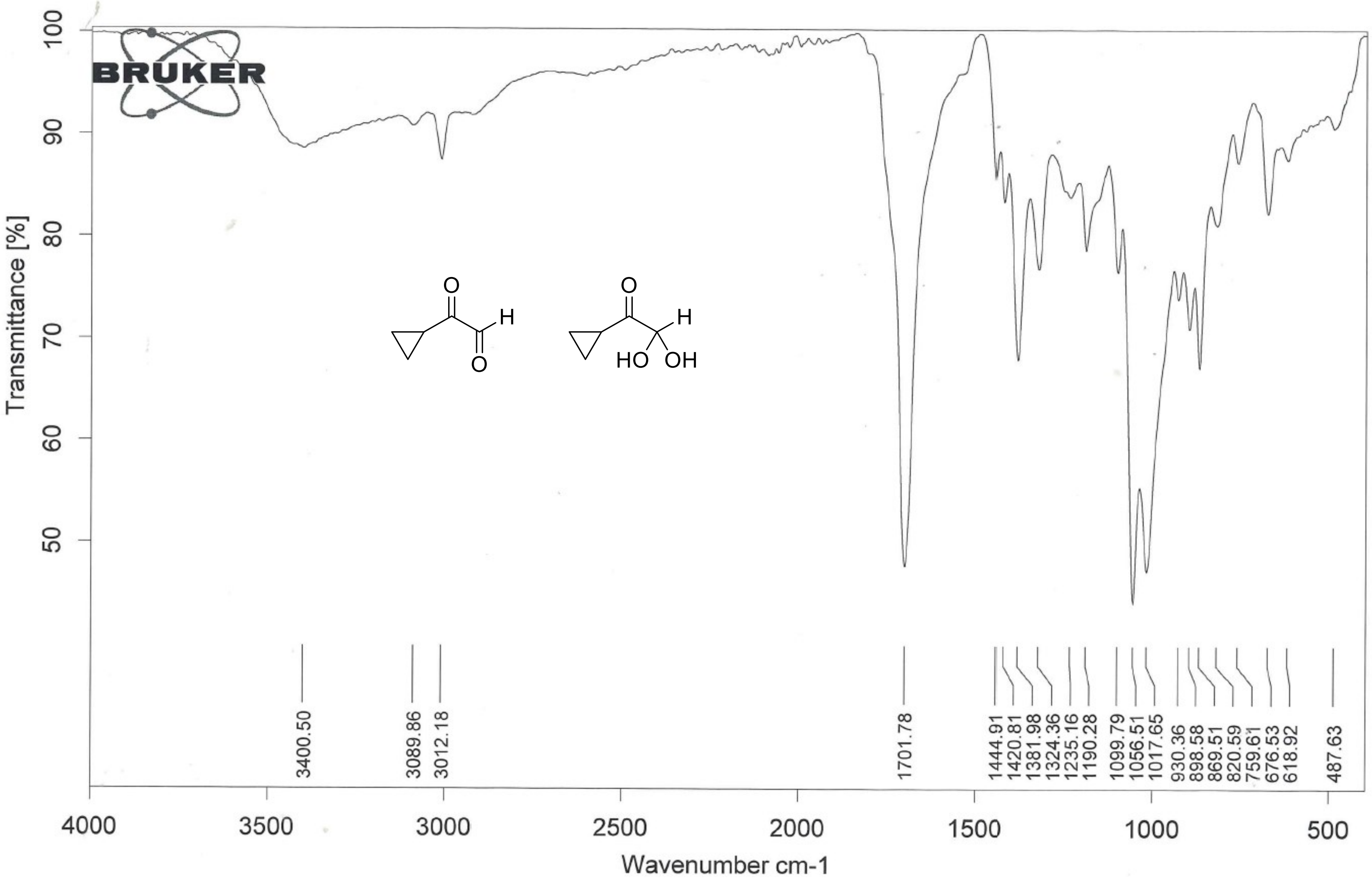

N-methoxy-*N*-methyl-2-phenylacetamide 25, ^{1}H NMR in CDCl3

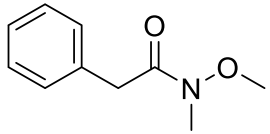

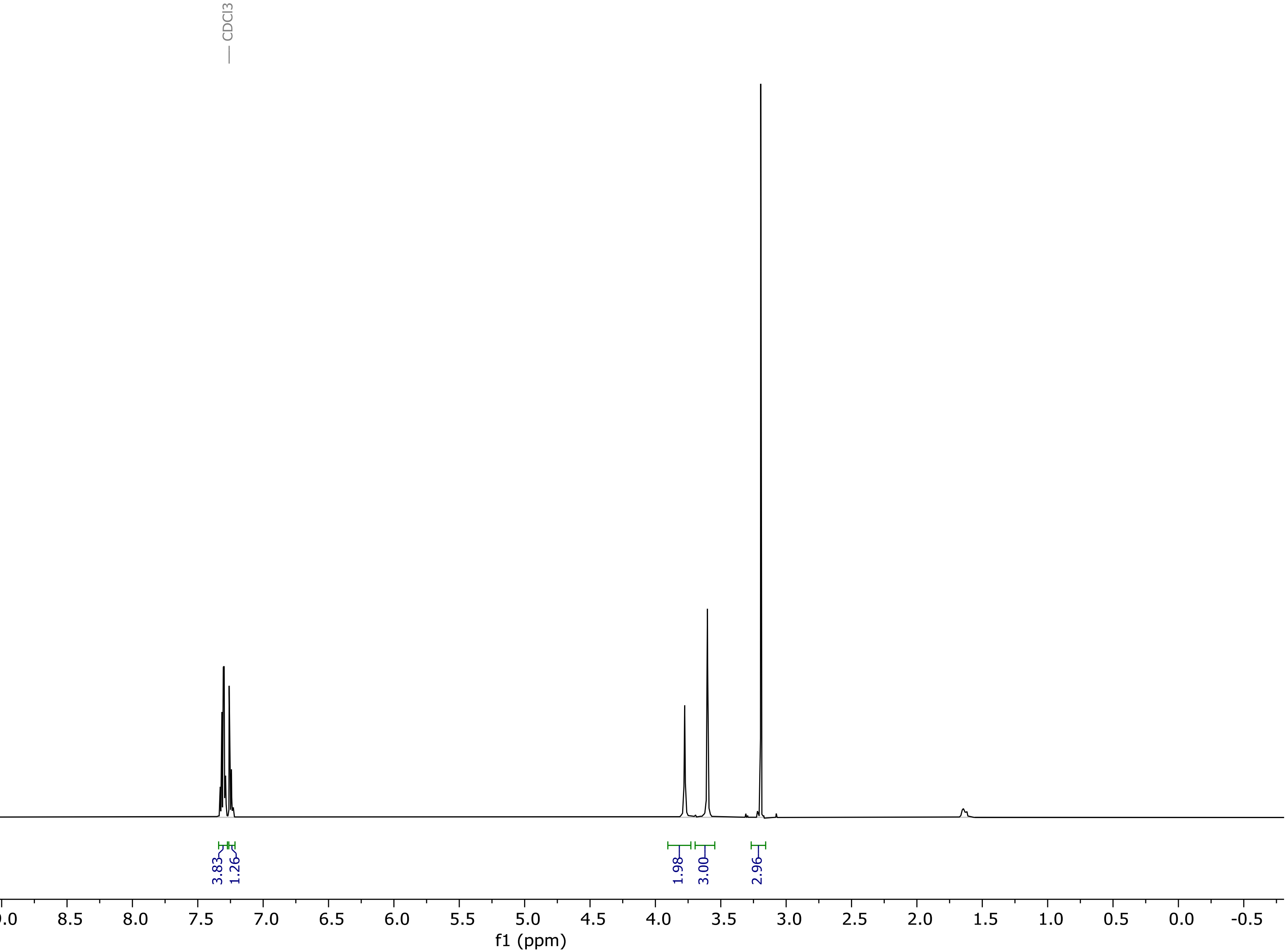

***N*-methoxy-*N*-methyl-2-phenylacetamide 25, ^{13}C NMR in $CDCl_3$**

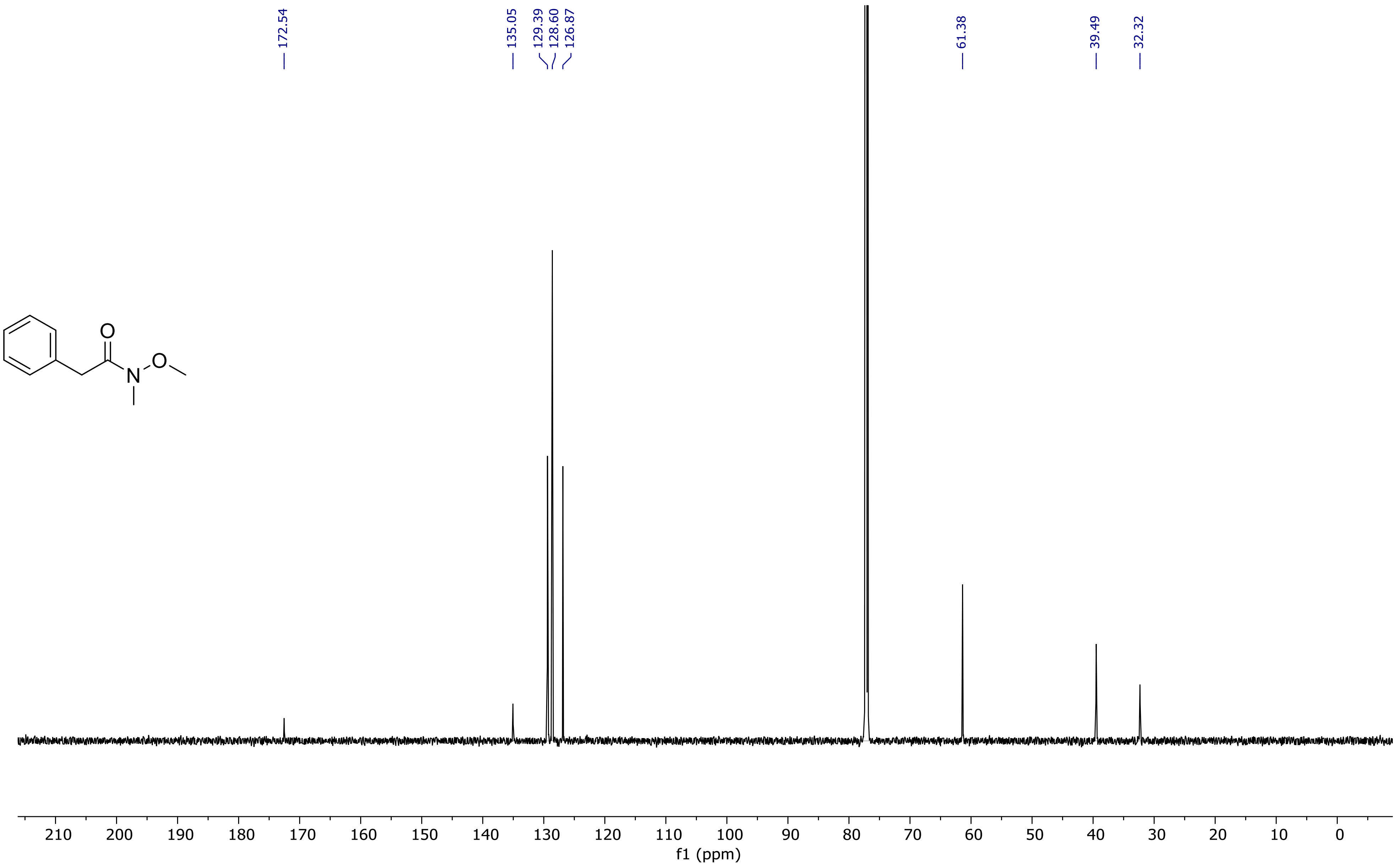

Benzyl vinyl ketone 26, ^{1}H NMR in $CDCl_3$

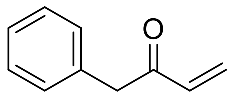

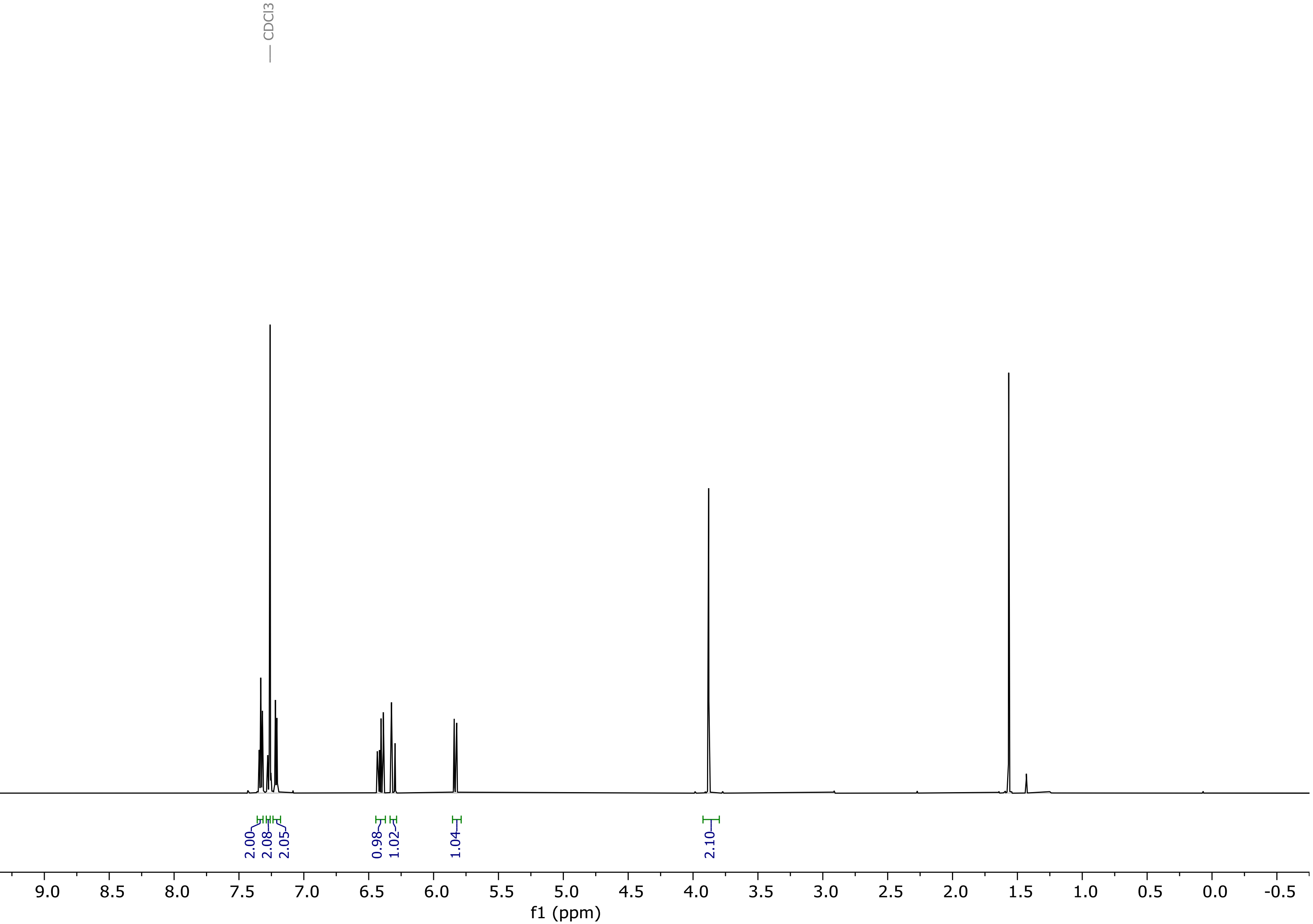

Benzyl vinyl ketone 26, ^{13}C NMR in $CDCl_3$

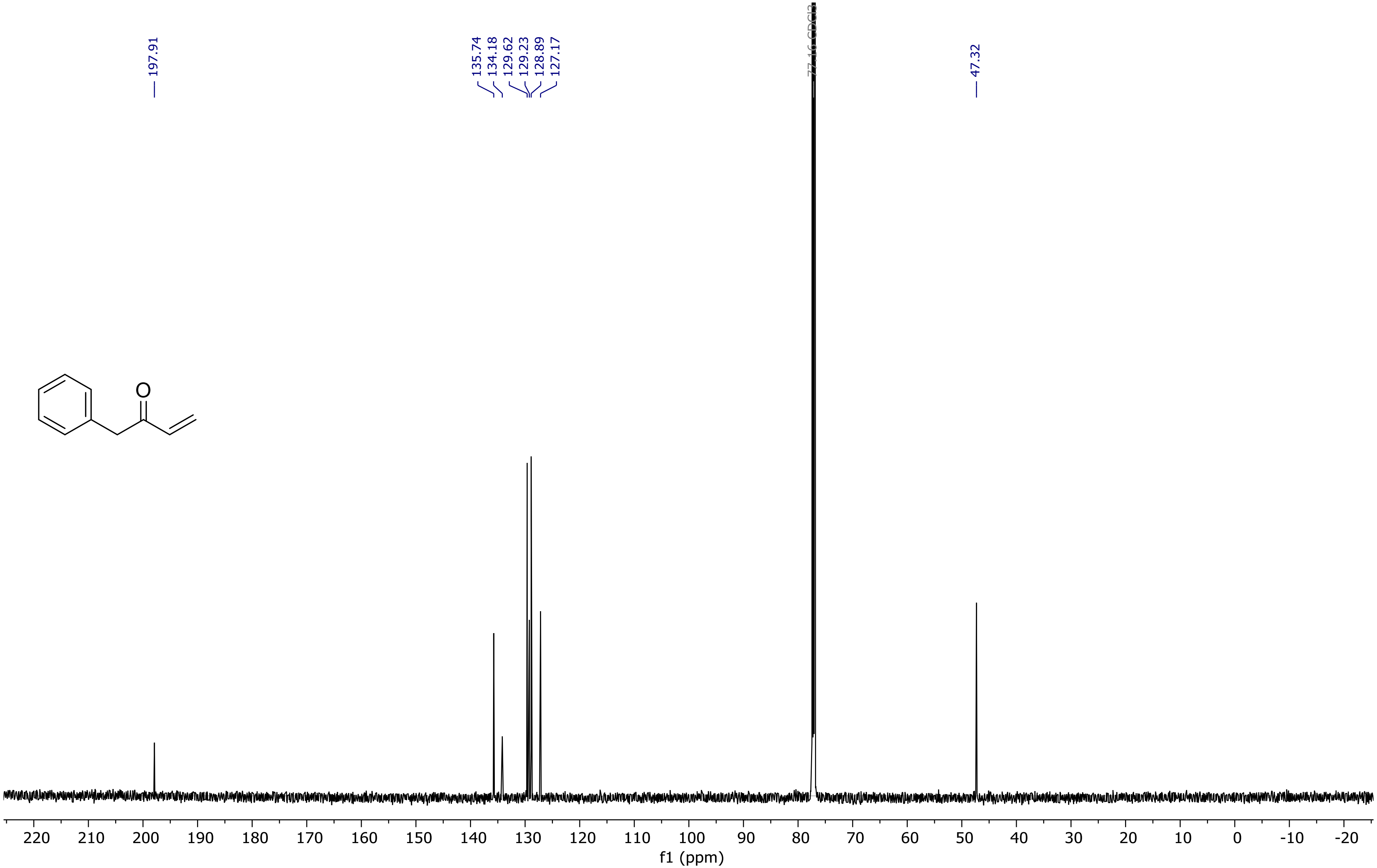

3- Oxo-3-phenylpropanal S13ac, ^{1}H NMR in CDCl₃ (*carried forward to the next step, without further purification*)

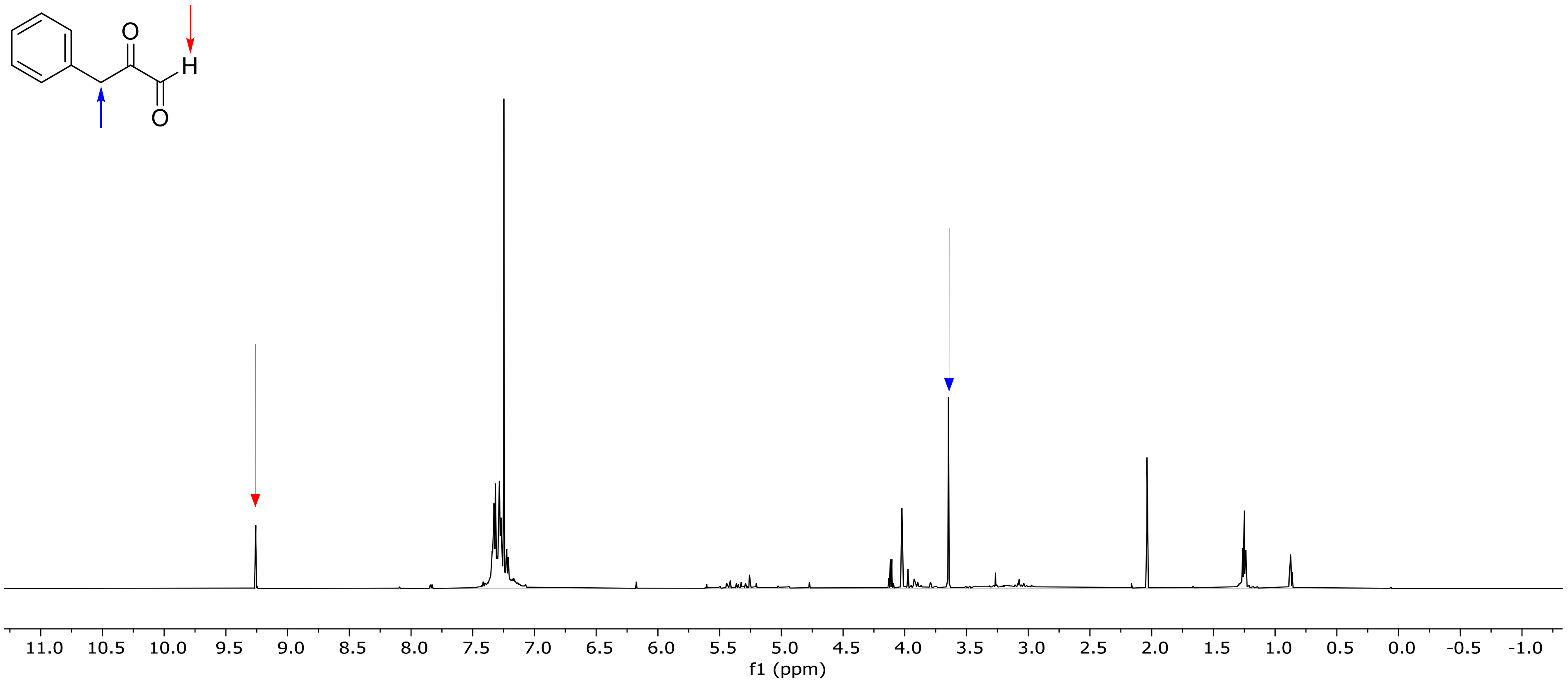

22. HPLC traces

1,3-Dibenzyl-5-phenylimidazolidine-2,4-dione, 17a

HPLC (Astec® Cellulose DMP, 94:6 hexane–EtOH, 1 mL/min, 254 nm)

Chromatogram

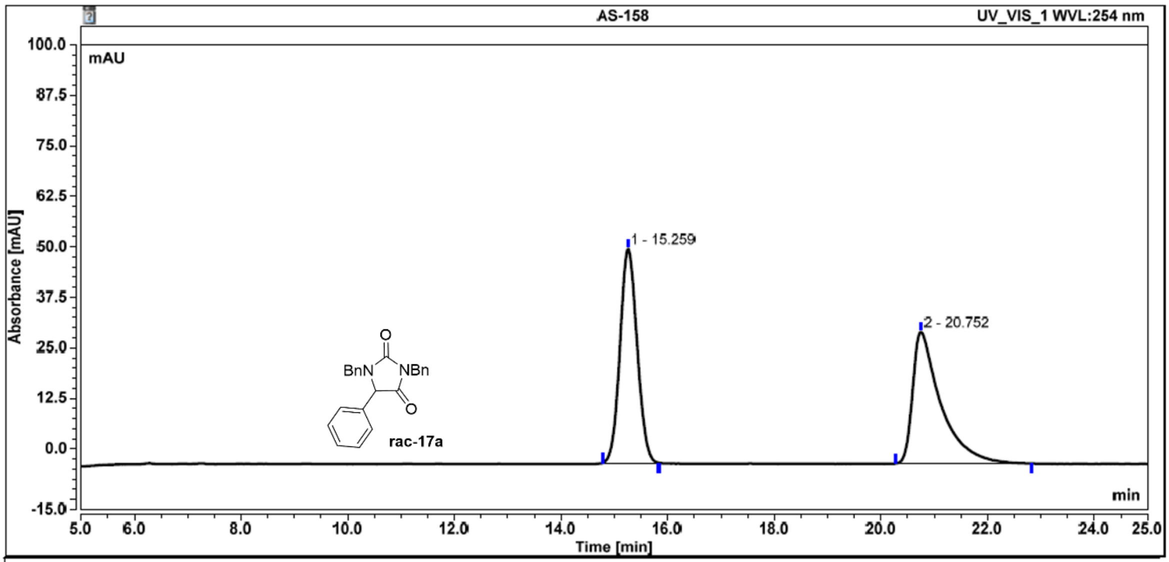

Integration Results

No.	Retention Time min	Area mAU*min	Height mAU	Relative Area %	Relative Height %
1	15.259	19.645	53.032	49.75	61.93
2	20.752	19.841	32.596	50.25	38.07
**Total:**		**39.486**	**85.627**	**100.00**	**100.00**

Chromatogram

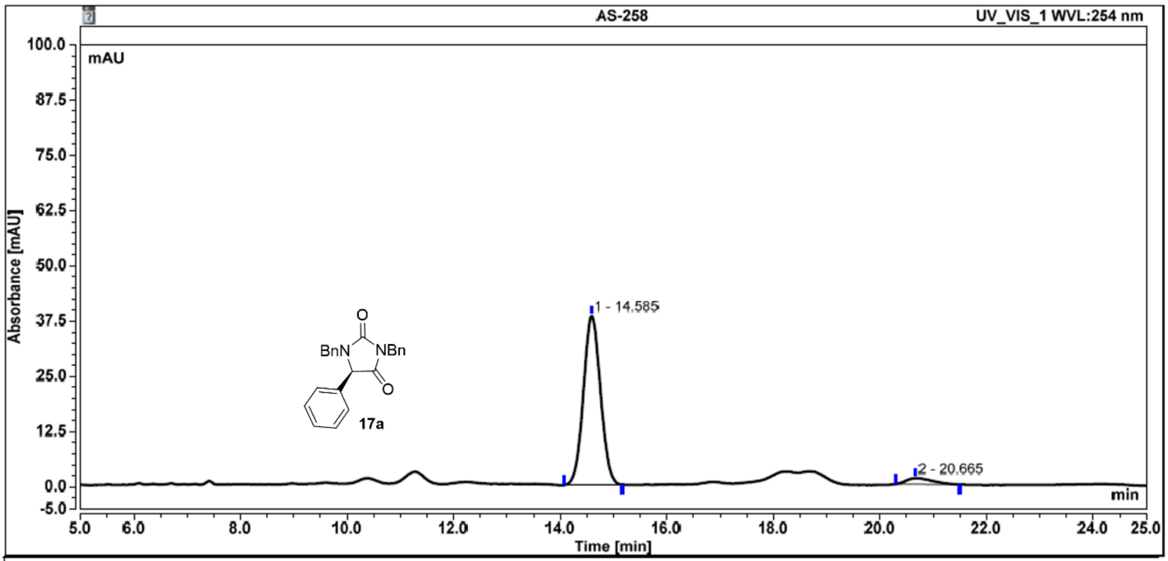

Integration Results

No.	Retention Time min	Area mAU*min	Height mAU	Relative Area %	Relative Height %
1	14.585	14.185	38.238	94.82	96.58
2	20.665	0.775	1.354	5.18	3.42
**Total:**		**14.960**	**39.592**	**100.00**	**100.00**

1,3-Dibenzyl-5-phenylimidazolidine-2,4-dione, 17a (*after recrystallisation)*

HPLC (Astec® Cellulose DMP, 94:6 hexane–EtOH, 1 mL/min, 254 nm)

Chromatogram

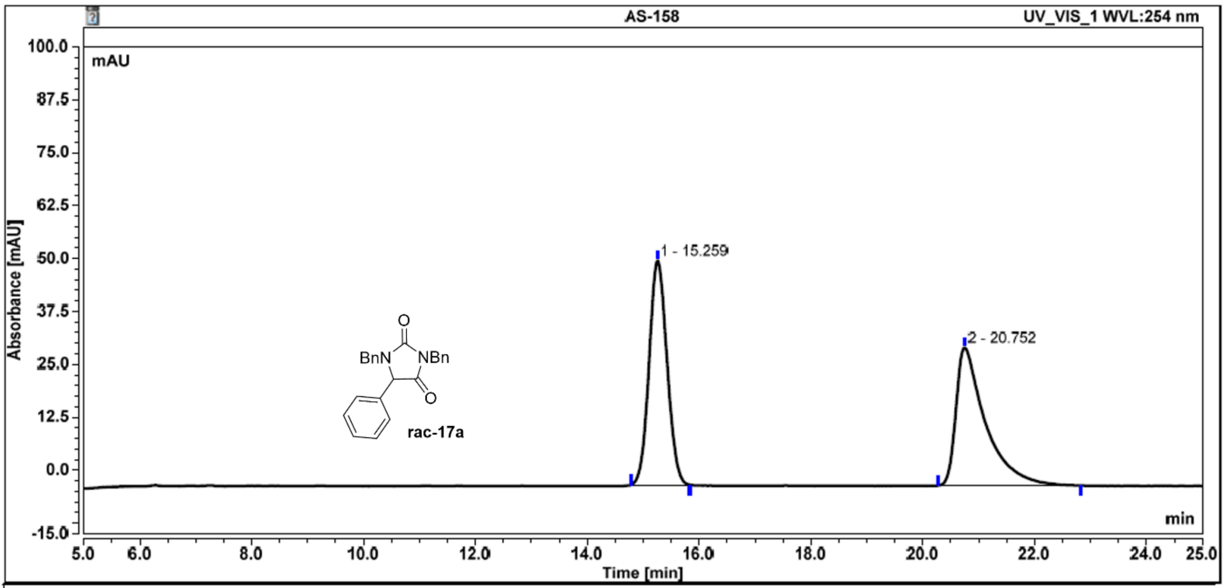

Integration Results

No.	Retention Time min	Area mAU*min	Height mAU	Relative Area %	Relative Height %
1	15.259	19.645	53.032	49.75	61.93
2	20.752	19.841	32.596	50.25	38.07
**Total:**		**39.486**	**85.627**	**100.00**	**100.00**

Chromatogram

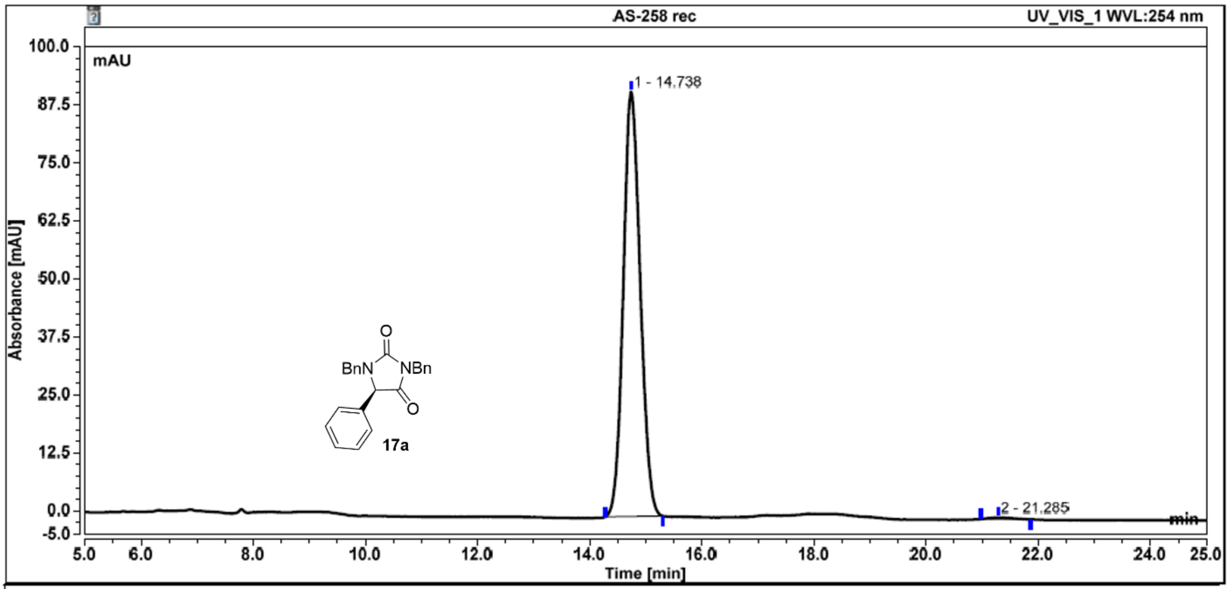

Integration Results

No.	Retention Time min	Area mAU*min	Height mAU	Relative Area %	Relative Height %
1	14.738	31.964	91.457	99.49	99.63
2	21.285	0.164	0.341	0.51	0.37
**Total:**		**32.127**	**91.798**	**100.00**	**100.00**

1,3-Dibenzyl-5-phenylimidazolidine-2,4-dione, 17a (*scale-up)*

HPLC (Astec® Cellulose DMP, 94:6 hexane–EtOH, 1 mL/min, 254 nm)

Chromatogram

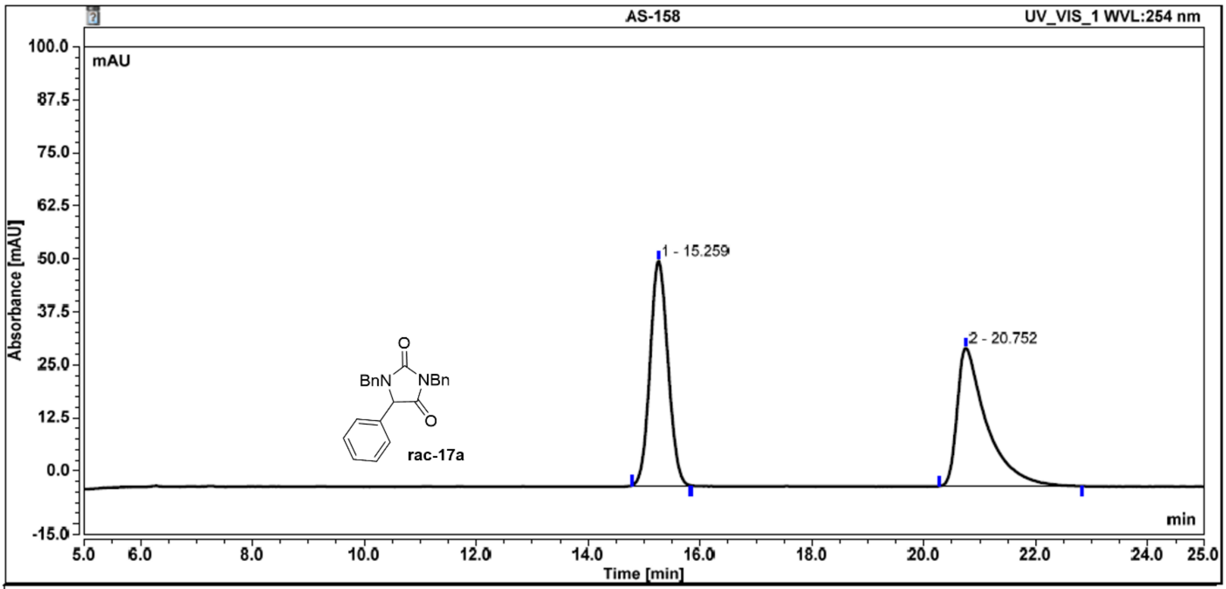

Integration Results

No.	Retention Time min	Area mAU*min	Height mAU	Relative Area %	Relative Height %
1	15.259	19.645	53.032	49.75	61.93
2	20.752	19.841	32.596	50.25	38.07
**Total:**		**39.486**	**85.627**	**100.00**	**100.00**

Chromatogram

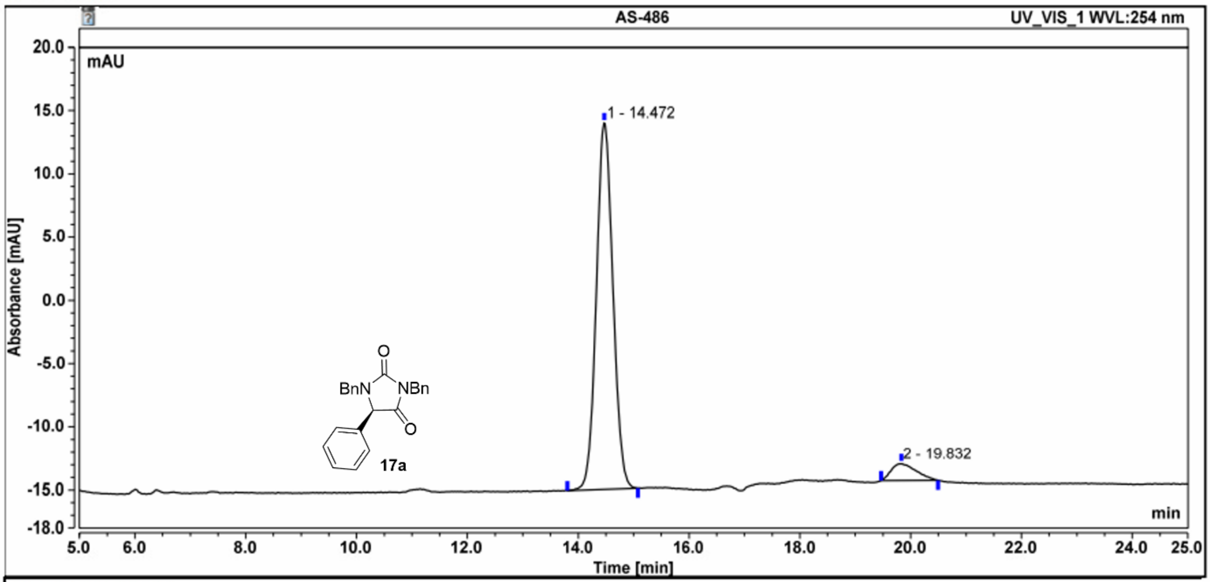

Integration Results

No.	Retention Time min	Area mAU*min	Height mAU	Relative Area %	Relative Height %
1	14.472	10.053	29.007	93.60	95.52
2	19.832	0.688	1.360	6.40	4.48
**Total:**		**10.741**	**30.367**	**100.00**	**100.00**

1,3-Dibenzyl-5-(4-methylphenyl)imidazolidine-2,4-dione, 17b

HPLC (CHIRALPAK® AS-3R, 60:40 MeCN–H_2O, 0.5 mL/min, 220 nm)

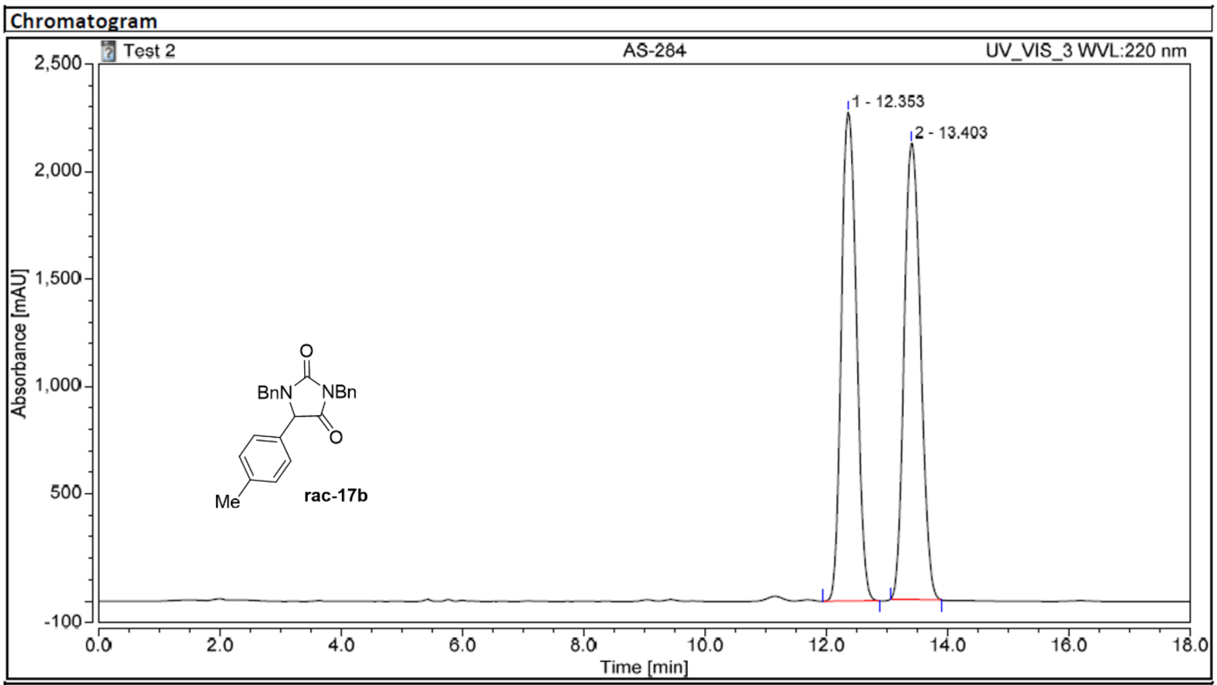

Integration Results

No.	Peak Name	Retention Time min	Area mAU*min	Height mAU	Relative Area %	Relative Height %	Amount n.a.
1		12.353	659.619	2275.408	49.66	51.71	n.a.
2		13.403	668.535	2124.527	50.34	48.29	n.a.
**Total:**			**1328.155**	**4399.936**	**100.00**	**100.00**	

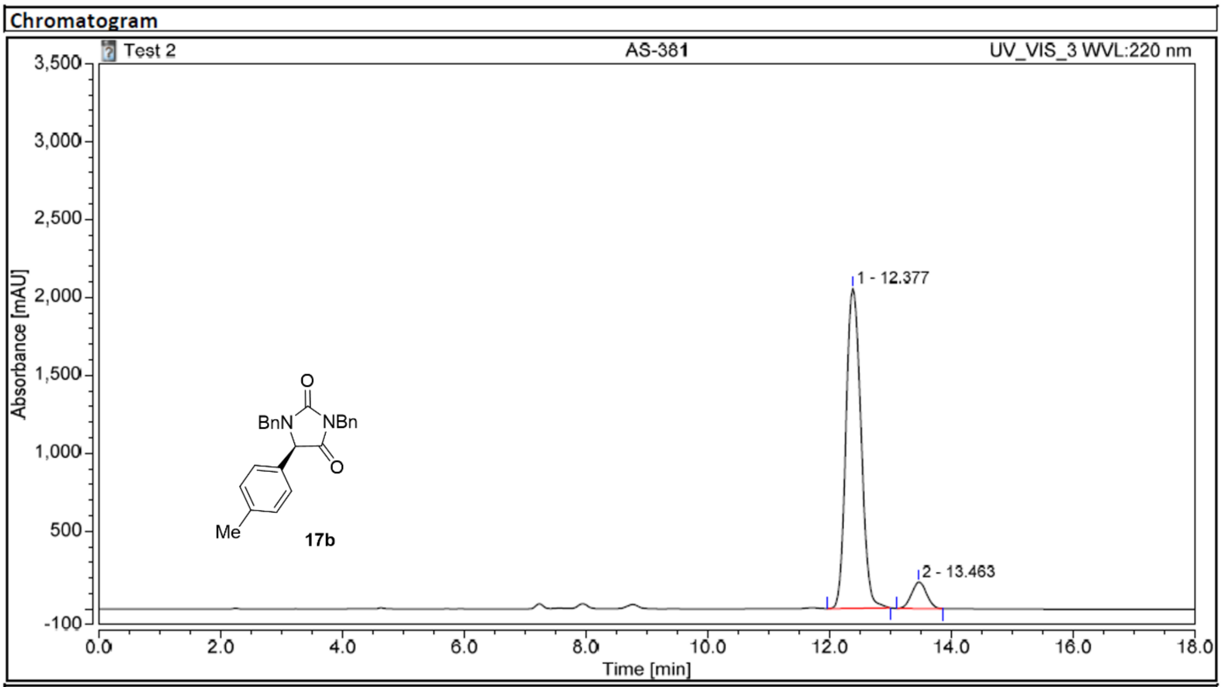

Integration Results

No.	Peak Name	Retention Time min	Area mAU*min	Height mAU	Relative Area %	Relative Height %	Amount n.a.
1		12.377	585.332	2055.719	92.35	92.35	n.a.
2		13.463	48.509	170.358	7.65	7.65	n.a.
**Total:**			**633.841**	**2226.077**	**100.00**	**100.00**	

1,3-Dibenzyl-5-(4-methylphenyl)imidazolidine-2,4-dione, 17b (*scale up*)

HPLC (CHIRALPAK® AS-3R, 60:40 MeCN–H_2O, 0.5 mL/min, 220 nm)

Chromatogram

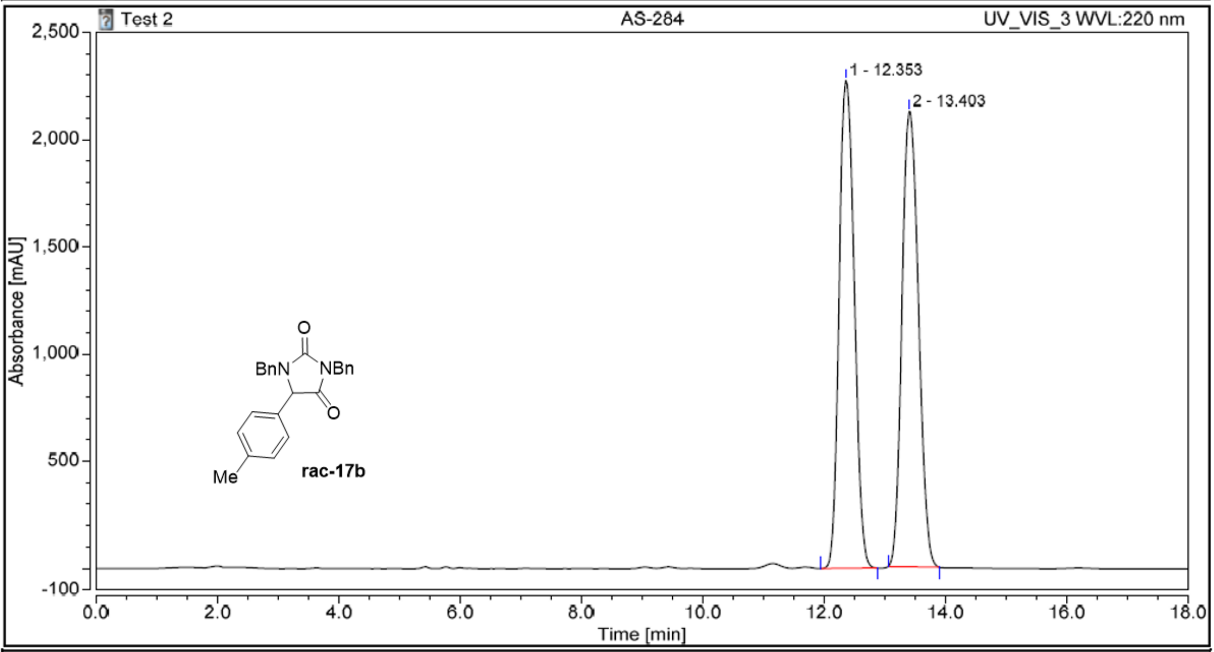

Integration Results

No.	Peak Name	Retention Time min	Area mAU*min	Height mAU	Relative Area %	Relative Height %	Amount n.a.
1		12.353	659.619	2275.408	49.66	51.71	n.a.
2		13.403	668.535	2124.527	50.34	48.29	n.a.
**Total:**			**1328.155**	**4399.936**	**100.00**	**100.00**	

Chromatogram

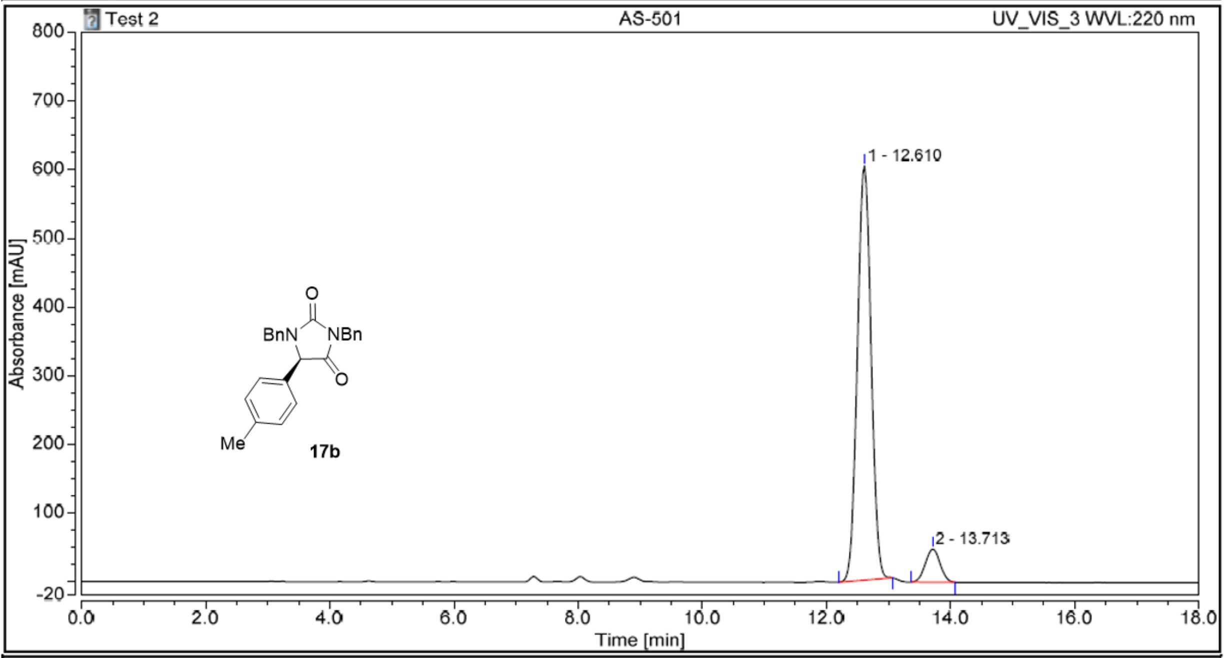

Integration Results

No.	Peak Name	Retention Time min	Area mAU*min	Height mAU	Relative Area %	Relative Height %	Amount n.a.
1		12.610	153.998	604.124	92.05	92.60	n.a.
2		13.713	13.299	48.261	7.95	7.40	n.a.
**Total:**			**167.297**	**652.385**	**100.00**	**100.00**	

1,3-Dibenzyl-5-(4-hydroxyphenyl)imidazolidine-2,4-dione, 17c

HPLC (Astec® Cellulose DMP, 80:20 hexane–EtOH, 1 mL/min, 254 nm)

Chromatogram

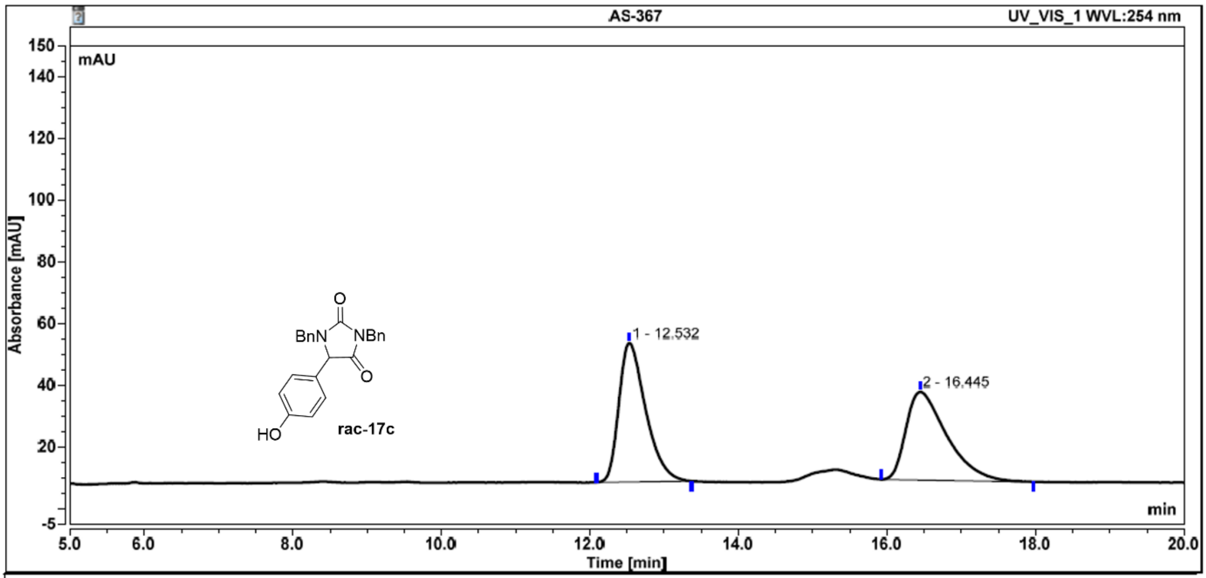

Integration Results

No.	Retention Time min	Area mAU*min	Height mAU	Relative Area %	Relative Height %
1	12.532	18.850	45.035	50.86	61.17
2	16.445	18.215	28.589	49.14	38.83
**Total:**		**37.065**	**73.624**	**100.00**	**100.00**

Chromatogram

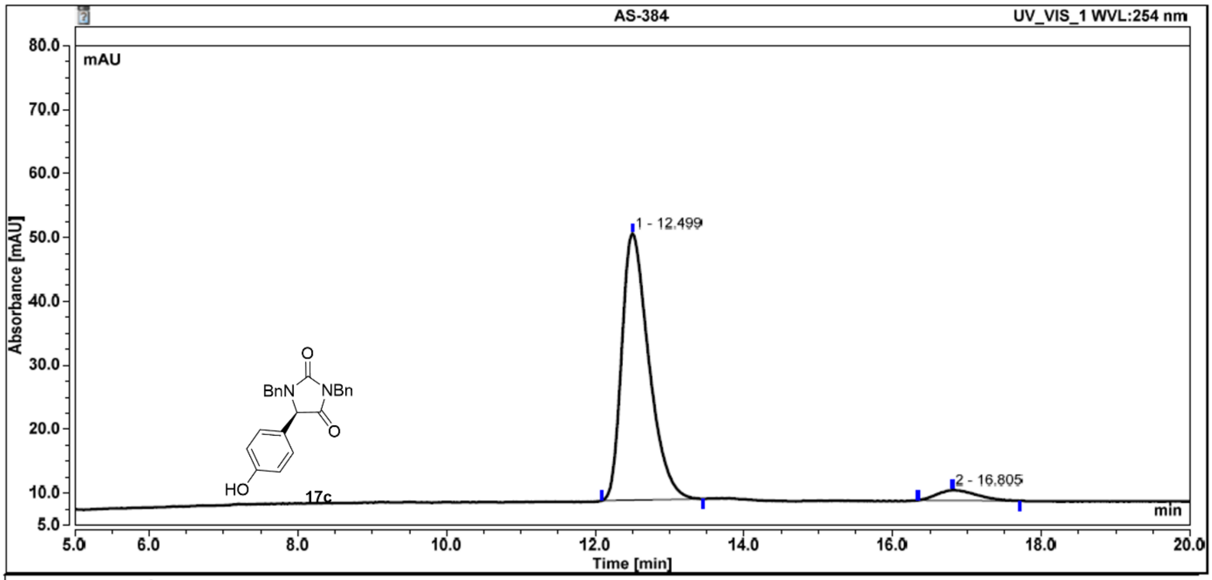

Integration Results

No.	Retention Time min	Area mAU*min	Height mAU	Relative Area %	Relative Height %
1	12.499	17.627	41.774	94.60	96.26
2	16.805	1.005	1.624	5.40	3.74
**Total:**		**18.633**	**43.398**	**100.00**	**100.00**

1,3-Dibenzyl-5-(4-hydroxyphenyl)imidazolidine-2,4-dione, 17c (*after recrystallisation)*

HPLC (Astec® Cellulose DMP, 80:20 hexane–EtOH, 1 mL/min, 254 nm)

Chromatogram

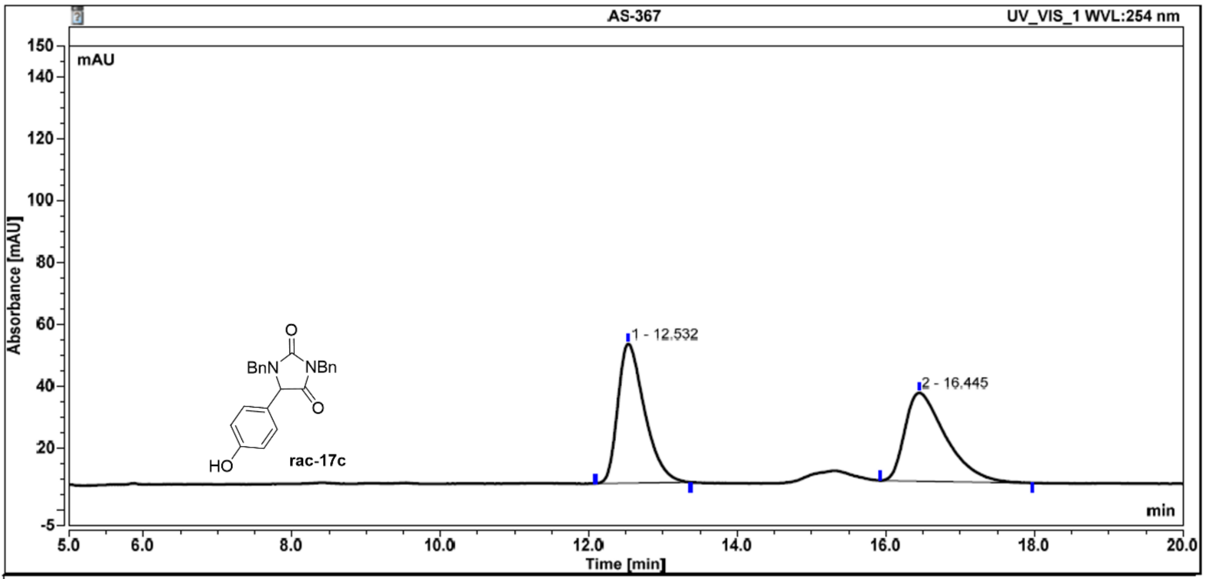

Integration Results

No.	Retention Time min	Area mAU*min	Height mAU	Relative Area %	Relative Height %
1	12.532	18.850	45.035	50.86	61.17
2	16.445	18.215	28.589	49.14	38.83
**Total:**		**37.065**	**73.624**	**100.00**	**100.00**

Chromatogram

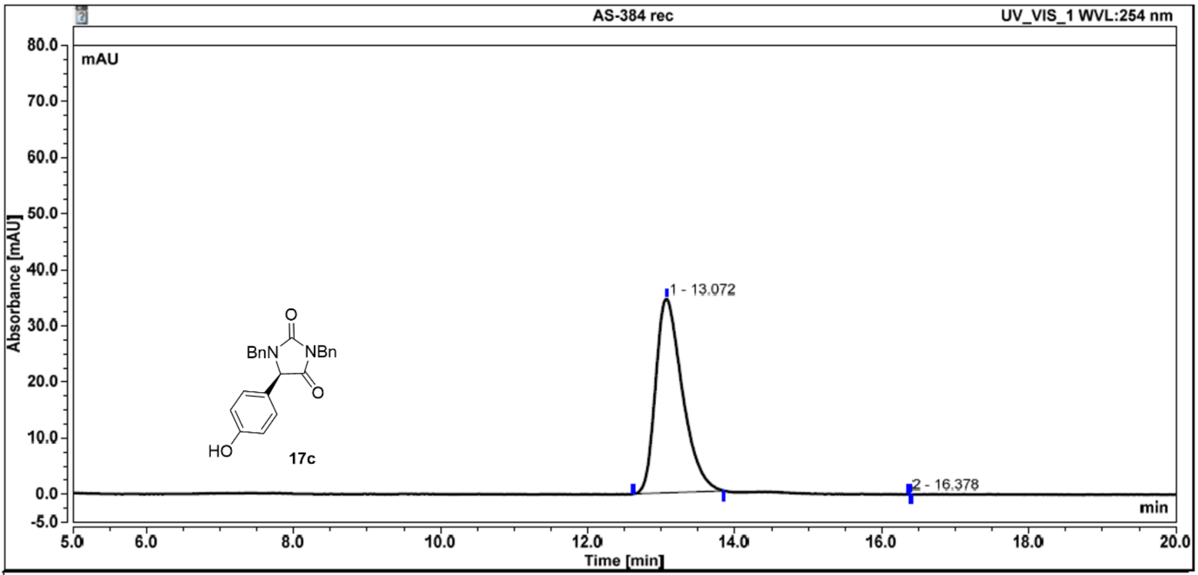

Integration Results

No.	Retention Time min	Area mAU*min	Height mAU	Relative Area %	Relative Height %
1	13.072	14.607	34.582	100.00	99.99
2	16.378	0.000	0.002	0.00	0.01
**Total:**		**14.607**	**34.584**	**100.00**	**100.00**

1,3-Dibenzyl-5-(4-methoxyphenyl)imidazolidine-2,4-dione, 17d

HPLC (Astec® Cellulose DMP, 80:20 hexane–*n*-PrOH, 1 mL/min, 220 nm)

Chromatogram

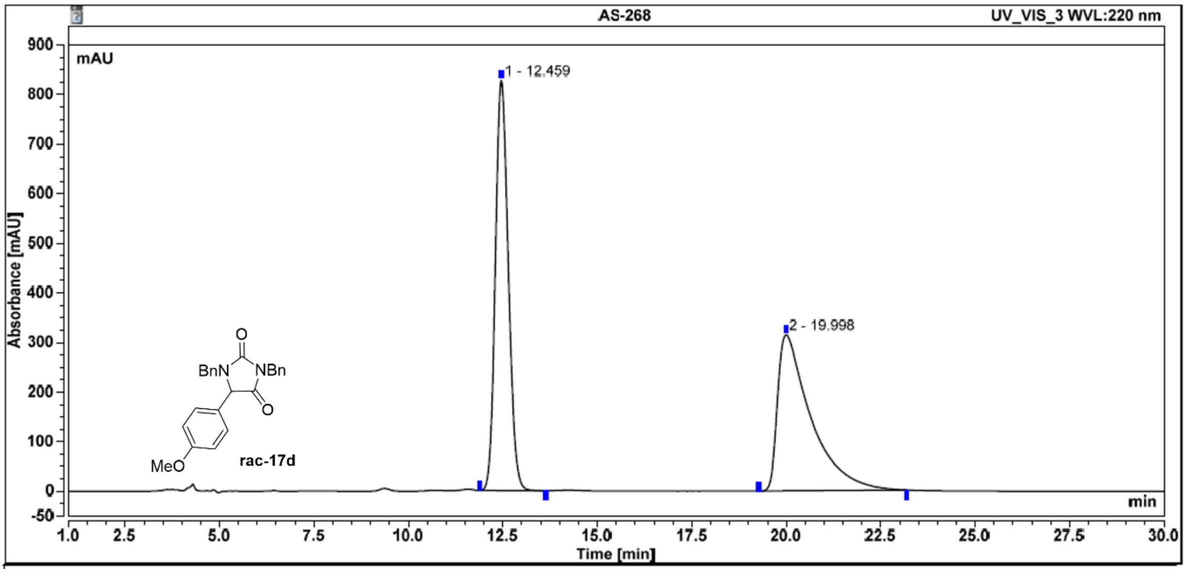

Integration Results

No.	Retention Time min	Area mAU*min	Height mAU	Relative Area %	Relative Height %
1	12.459	325.765	827.572	50.34	72.44
2	19.998	321.370	314.890	49.66	27.56
**Total:**		**647.136**	**1142.462**	**100.00**	**100.00**

Chromatogram

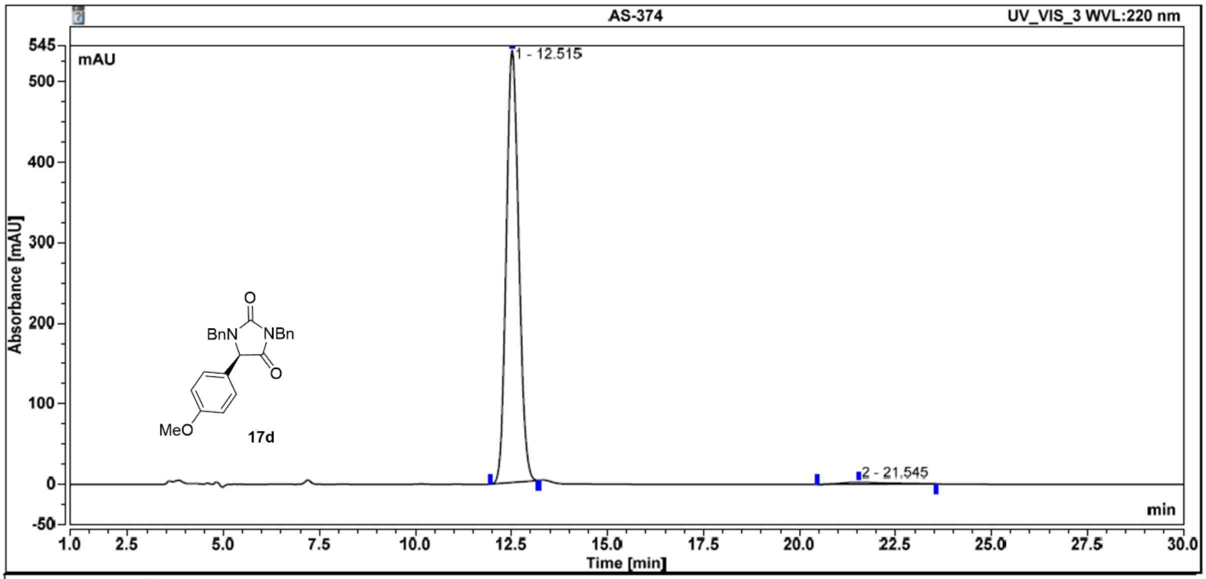

Integration Results

No.	Retention Time min	Area mAU*min	Height mAU	Relative Area %	Relative Height %
1	12.515	200.906	536.942	98.44	99.57
2	21.545	3.185	2.341	1.56	0.43
**Total:**		**204.091**	**539.284**	**100.00**	**100.00**

1,3-Dibenzyl-5-(4-methoxyphenyl)imidazolidine-2,4-dione, 17d (*scale-up*)

HPLC (Astec® Cellulose DMP, 80:20 hexane–*n*-PrOH, 1 mL/min, 220 nm)

Chromatogram

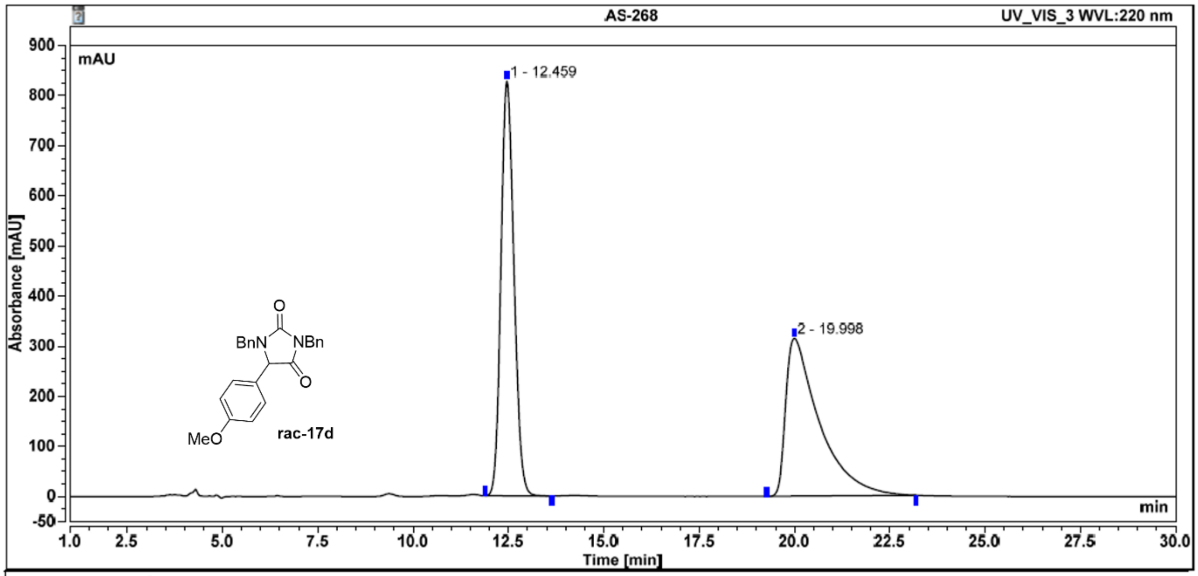

Integration Results

No.	Retention Time min	Area mAU*min	Height mAU	Relative Area %	Relative Height %
1	12.459	325.765	827.572	50.34	72.44
2	19.998	321.370	314.890	49.66	27.56
**Total:**		**647.136**	**1142.462**	**100.00**	**100.00**

Chromatogram

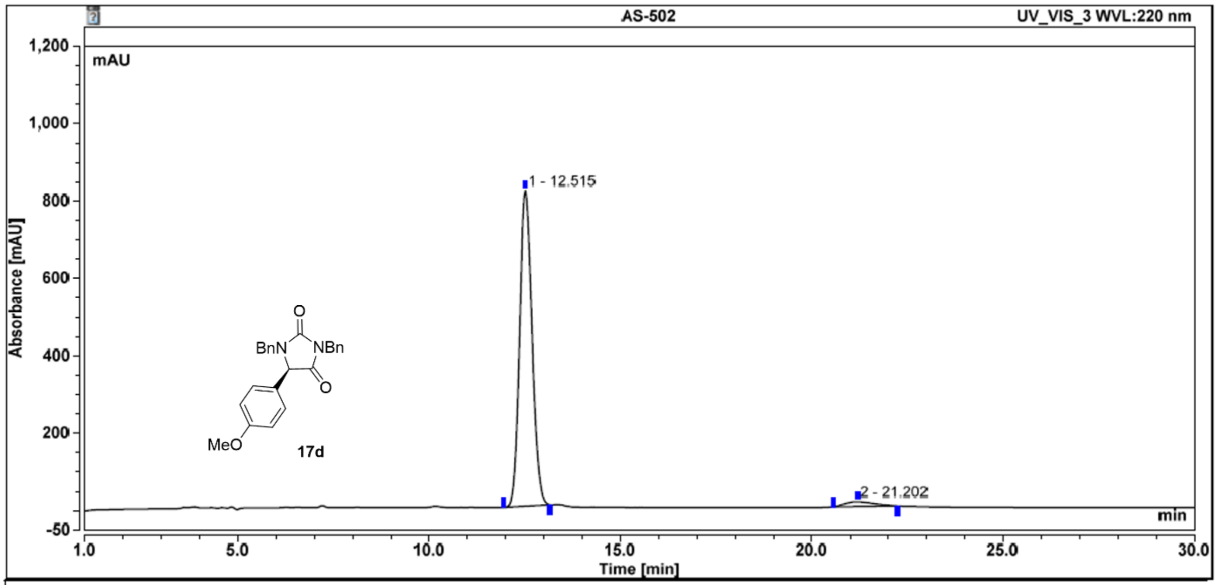

Integration Results

No.	Retention Time min	Area mAU*min	Height mAU	Relative Area %	Relative Height %
1	12.515	305.595	815.031	96.70	98.56
2	21.202	10.421	11.917	3.30	1.44
**Total:**		**316.016**	**826.948**	**100.00**	**100.00**

1,3-Dibenzyl-5-(4-phenoxyphenyl)imidazolidine-2,4-dione, 17e

HPLC (Astec® Cellulose DMP, 80:20 hexane–*n*-PrOH, 1 mL/min, 254 nm)

Chromatogram

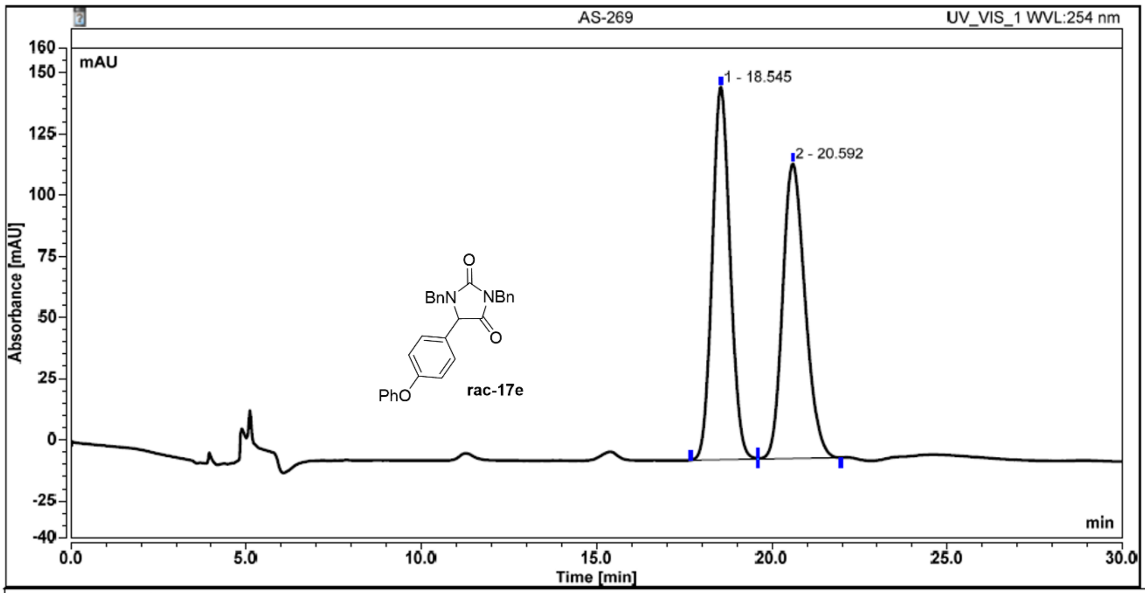

Integration Results

No.	Retention Time min	Area mAU*min	Height mAU	Relative Area %	Relative Height %
1	18.545	90.334	152.221	50.34	55.82
2	20.592	89.129	120.477	49.66	44.18
**Total:**		**179.463**	**272.698**	**100.00**	**100.00**

Chromatogram

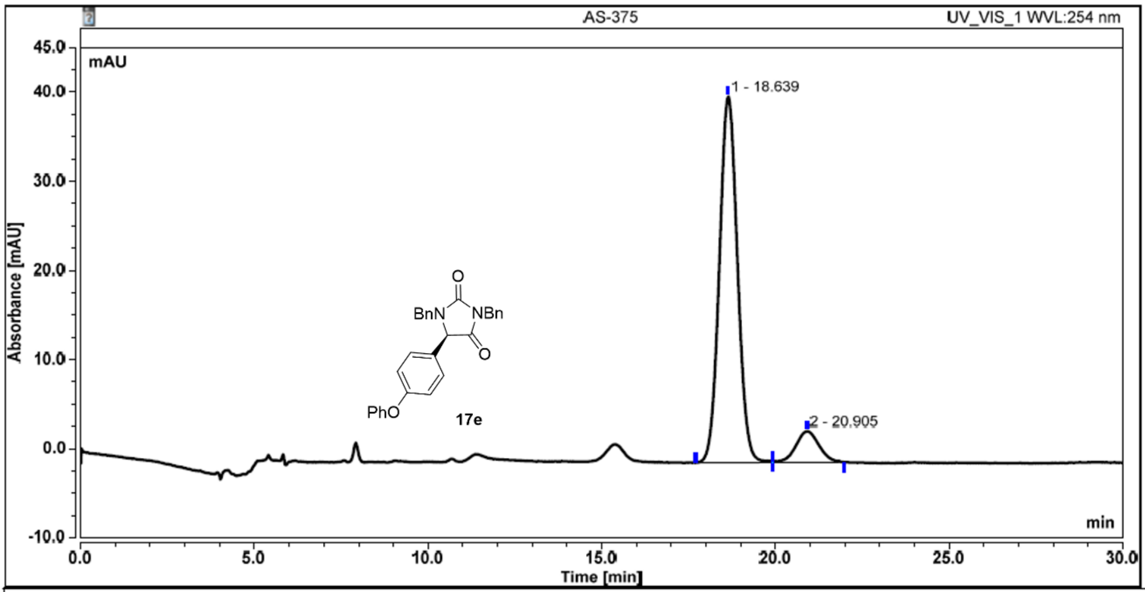

Integration Results

No.	Retention Time min	Area mAU*min	Height mAU	Relative Area %	Relative Height %
1	18.639	24.813	41.111	90.13	92.16
2	20.905	2.718	3.496	9.87	7.84
**Total:**		**27.532**	**44.607**	**100.00**	**100.00**

1,3-Dibenzyl-5-(4-fluorophenyl)imidazolidine-2,4-dione, 17f

HPLC (Astec® Cellulose DMP, 80:20 hexane–*n*-PrOH, 1 mL/min, 254 nm)

Chromatogram

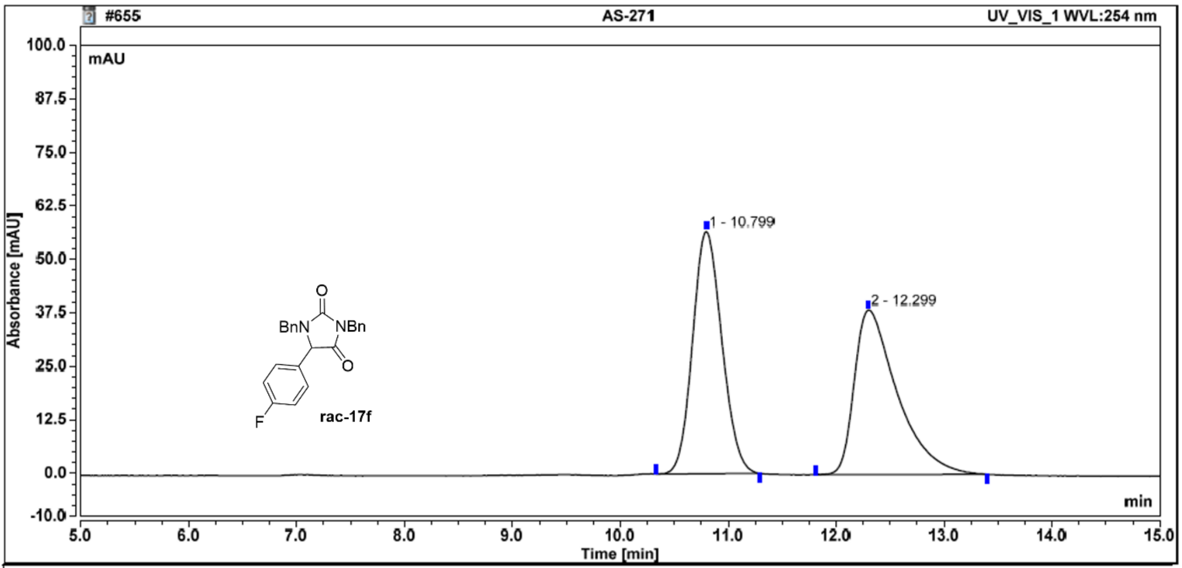

Integration Results

No.	Retention Time min	Area mAU*min	Height mAU	Relative Area %	Relative Height %
1	10.799	17.792	56.621	50.12	59.53
2	12.299	17.706	38.490	49.88	40.47
**Total:**		**35.499**	**95.112**	**100.00**	**100.00**

Chromatogram

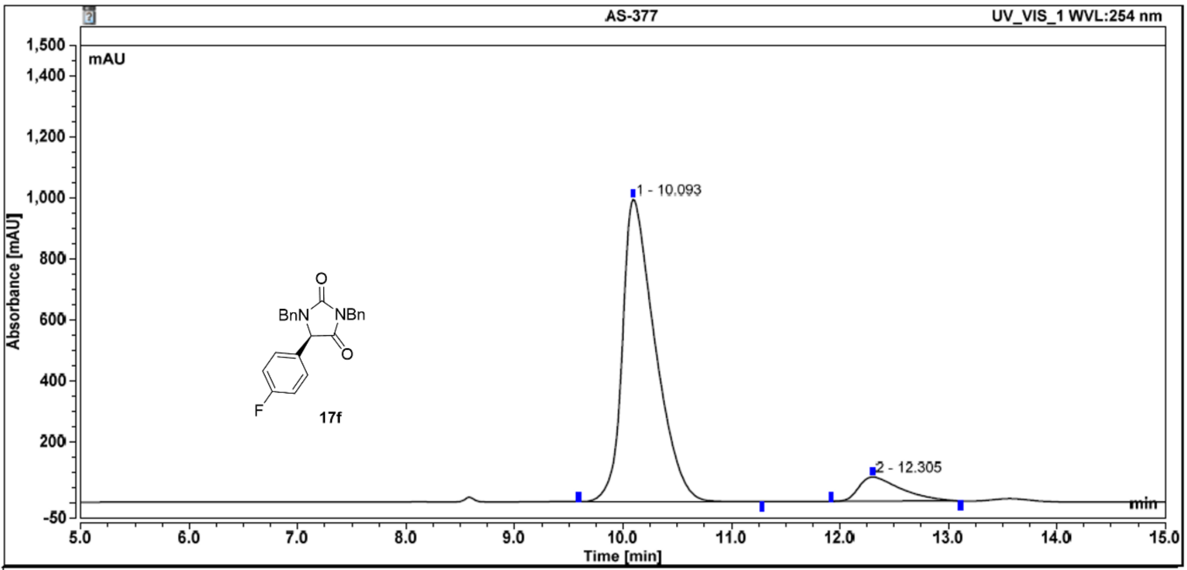

Integration Results

No.	Retention Time min	Area mAU*min	Height mAU	Relative Area %	Relative Height %
1	10.093	345.748	990.984	90.74	92.63
2	12.305	35.299	78.817	9.26	7.37
**Total:**		**381.048**	**1069.801**	**100.00**	**100.00**

1,3-Dibenzyl-5-(4-fluorophenyl)imidazolidine-2,4-dione, 17f (*scale up*)

HPLC (Astec® Cellulose DMP, 80:20 hexane–*n*-PrOH, 1 mL/min, 254 nm)

Chromatogram

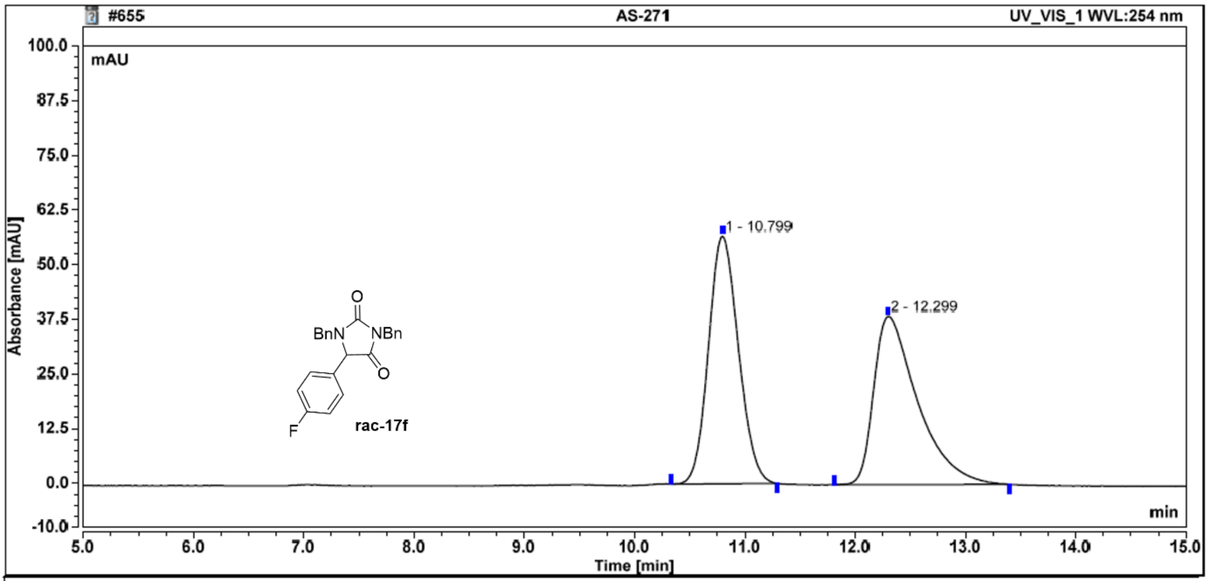

Integration Results

No.	Retention Time<br>min	Area<br>mAU*min	Height<br>mAU	Relative Area<br>%	Relative Height<br>%
1	10.799	17.792	56.621	50.12	59.53
2	12.299	17.706	38.490	49.88	40.47
**Total:**		**35.499**	**95.112**	**100.00**	**100.00**

Chromatogram

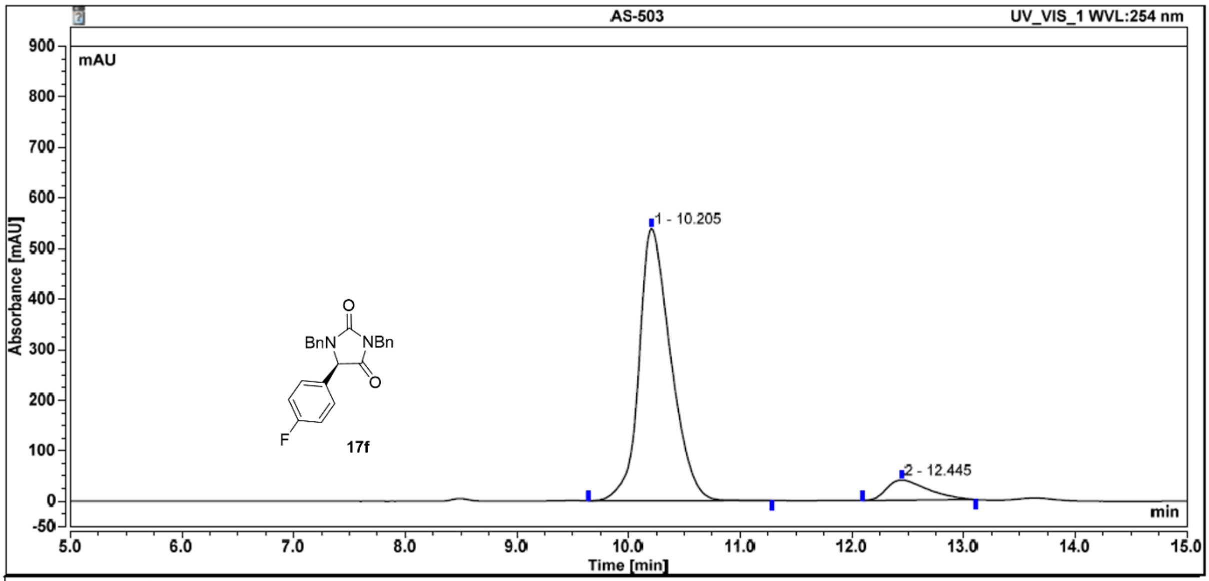

Integration Results

No.	Retention Time<br>min	Area<br>mAU*min	Height<br>mAU	Relative Area<br>%	Relative Height<br>%
1	10.205	177.215	538.317	91.29	93.12
2	12.445	16.918	39.794	8.71	6.88
**Total:**		**194.133**	**578.111**	**100.00**	**100.00**

1,3-Dibenzyl-5-(4-chlorophenyl)imidazolidine-2,4-dione 17g

HPLC (CHIRALPAK® AS-3R, 60:40 MeCN–H_2O, 0.5 mL/min, 238 nm)

Chromatogram

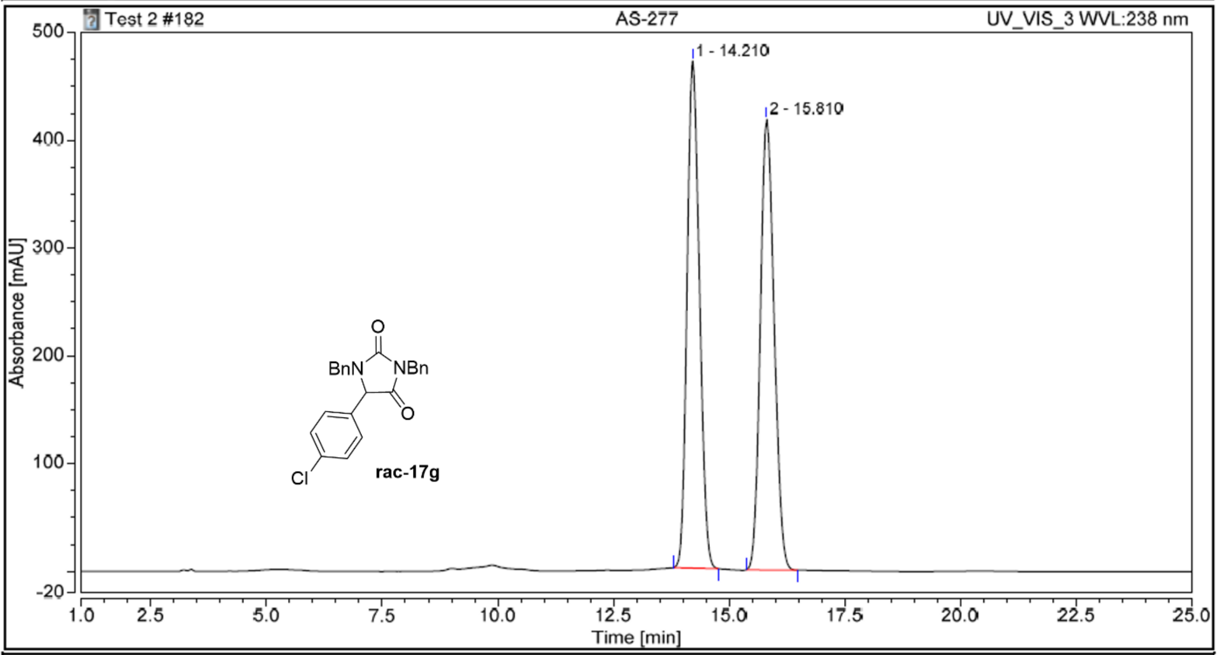

Integration Results

No.	Peak Name	Retention Time min	Area mAU*min	Height mAU	Relative Area %	Relative Height %	Amount n.a.
1		14.210	145.204	470.515	49.99	52.92	n.a.
2		15.810	145.276	418.558	50.01	47.08	n.a.
**Total:**			**290.480**	**889.073**	**100.00**	**100.00**	

Chromatogram

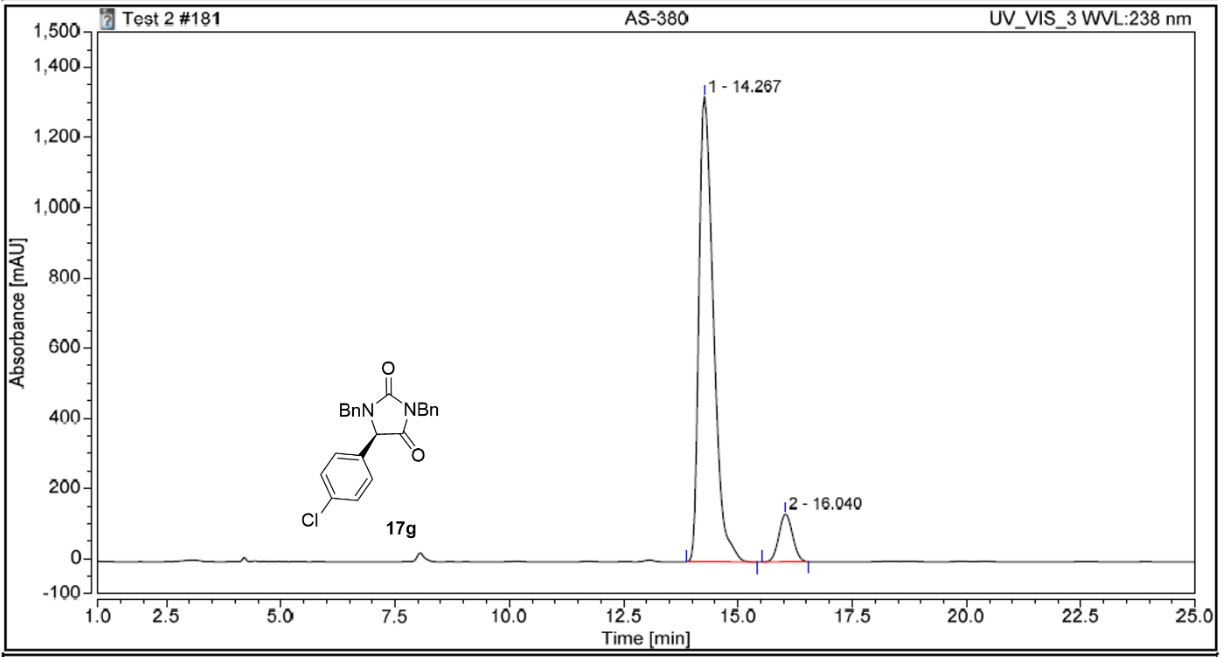

Integration Results

No.	Peak Name	Retention Time min	Area mAU*min	Height mAU	Relative Area %	Relative Height %	Amount n.a.
1		14.267	487.306	1325.087	91.18	90.68	n.a.
2		16.040	47.146	136.174	8.82	9.32	n.a.
**Total:**			**534.452**	**1461.261**	**100.00**	**100.00**	

1,3-Dibenzyl-5-(4-bromophenyl)imidazolidine-2,4-dione 17h

HPLC (CHIRALPAK® AS-3R, 50:50 MeCN–H_2O, 0.5 mL/min, 210 nm)

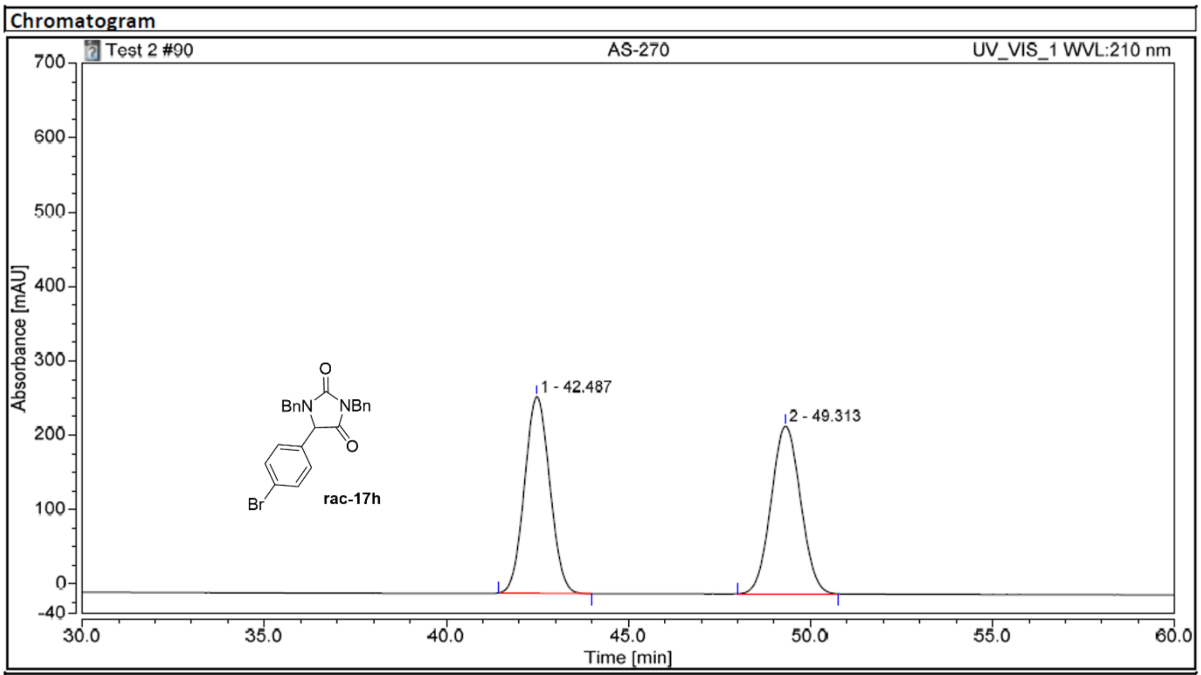

Integration Results

No.	Peak Name	Retention Time min	Area mAU*min	Height mAU	Relative Area %	Relative Height %	Amount n.a.
1		42.487	212.750	264.387	50.07	53.94	n.a.
2		49.313	212.131	225.740	49.93	46.06	n.a.
**Total:**			**424.881**	**490.128**	**100.00**	**100.00**	

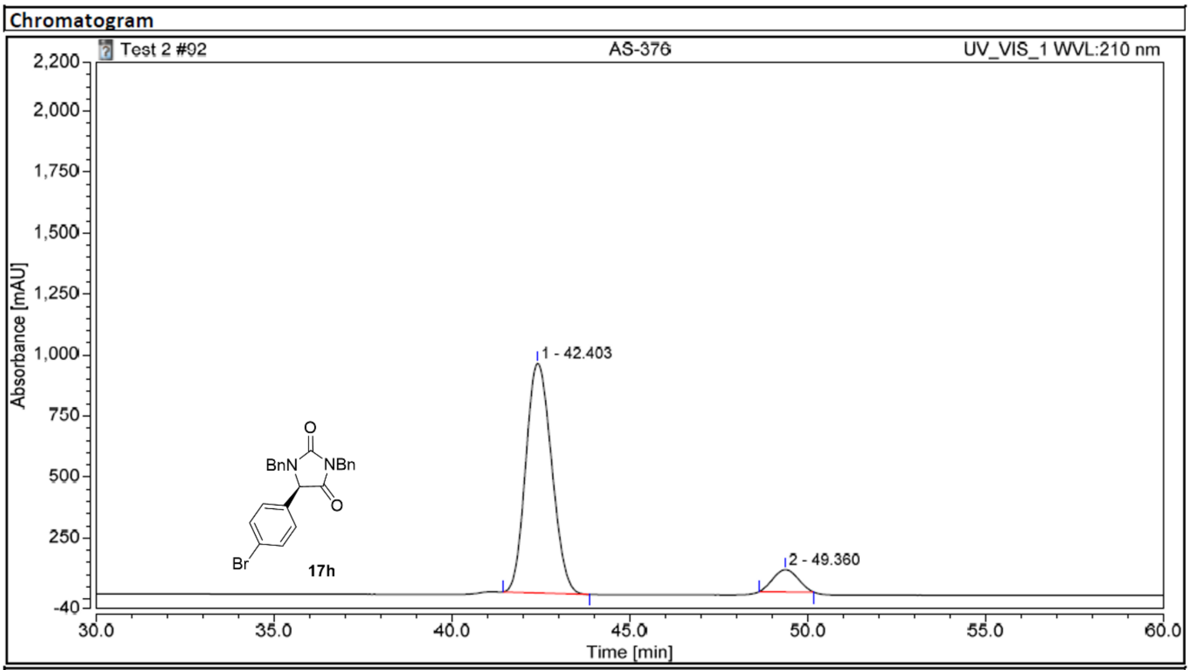

Integration Results

No.	Peak Name	Retention Time min	Area mAU*min	Height mAU	Relative Area %	Relative Height %	Amount n.a.
1		42.403	792.157	942.592	91.46	91.21	n.a.
2		49.360	73.996	90.884	8.54	8.79	n.a.
**Total:**			**866.153**	**1033.476**	**100.00**	**100.00**	

1,3-Dibenzyl-5-(4-bromophenyl)imidazolidine-2,4-dione 17h (*scale up*)

HPLC (CHIRALPAK® AS-3R, 50:50 MeCN–H_2O, 0.5 mL/min, 210 nm)

Chromatogram

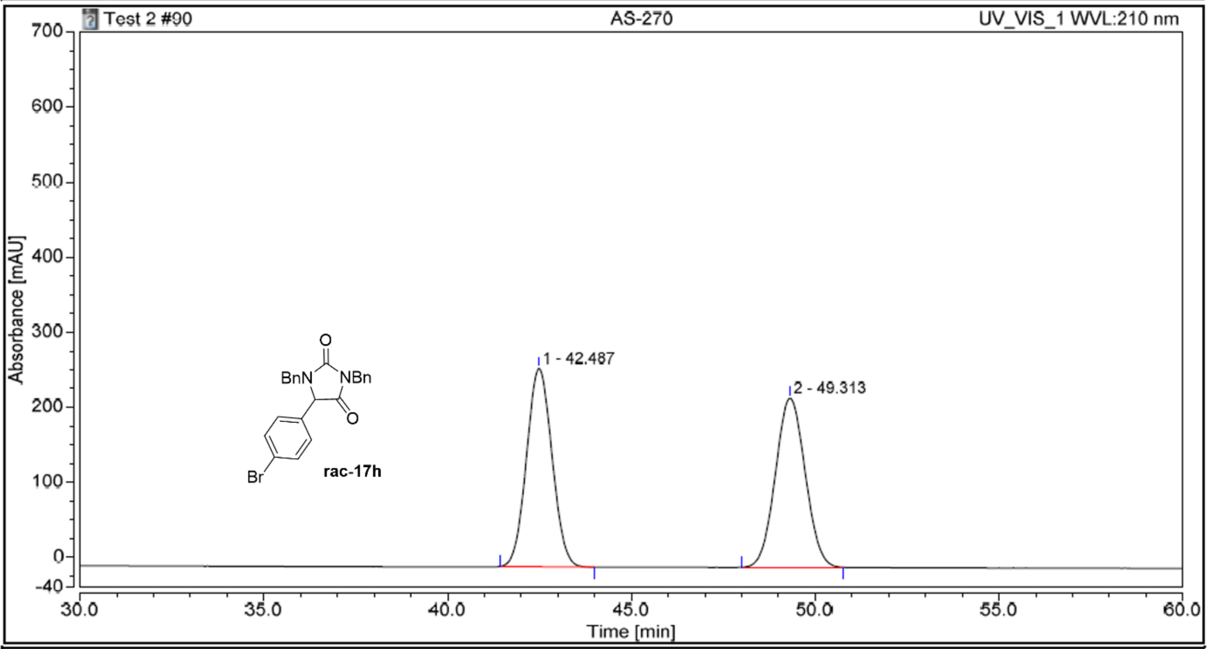

Integration Results

No.	Peak Name	Retention Time min	Area mAU*min	Height mAU	Relative Area %	Relative Height %	Amount n.a.
1		42.487	212.750	264.387	50.07	53.94	n.a.
2		49.313	212.131	225.740	49.93	46.06	n.a.
**Total:**			**424.881**	**490.128**	**100.00**	**100.00**	

Chromatogram

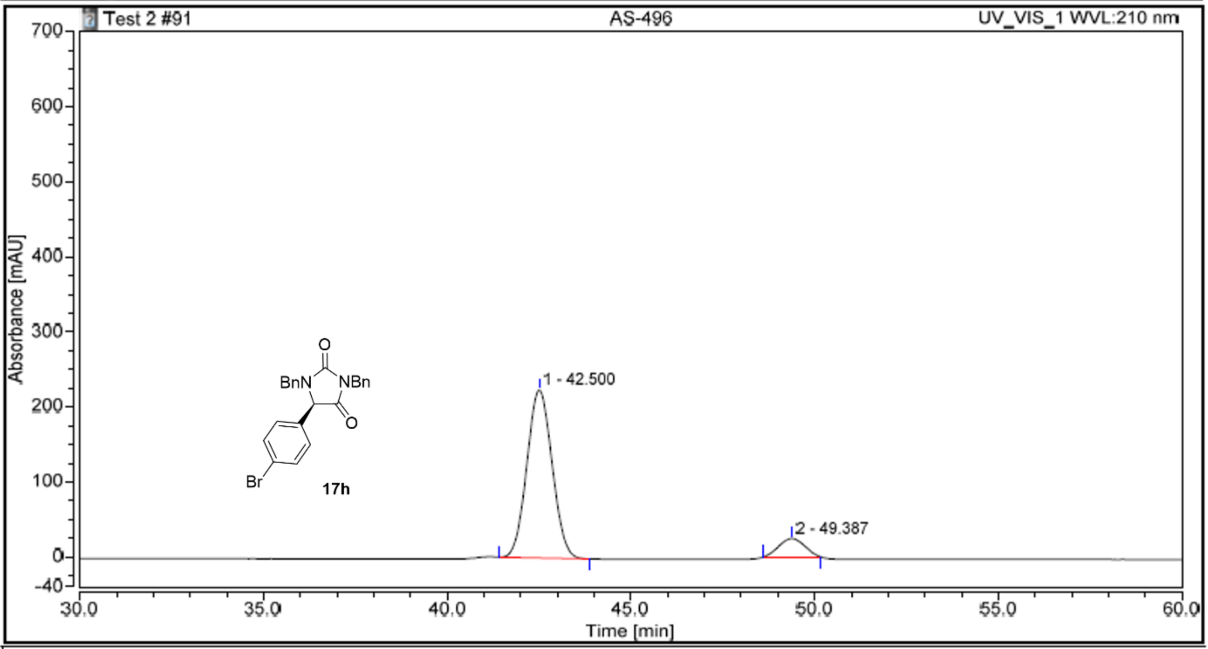

Integration Results

No.	Peak Name	Retention Time min	Area mAU*min	Height mAU	Relative Area %	Relative Height %	Amount n.a.
1		42.500	178.274	223.943	90.06	90.21	n.a.
2		49.387	19.672	24.306	9.94	9.79	n.a.
**Total:**			**197.946**	**248.250**	**100.00**	**100.00**	

1,3-Dibenzyl-5-(4-iodophenyl)imidazolidine-2,4-dione 17i

HPLC (CHIRALPAK® AS-3R, 70:30 MeCN–H_2O, 0.5 mL/min, 254 nm)

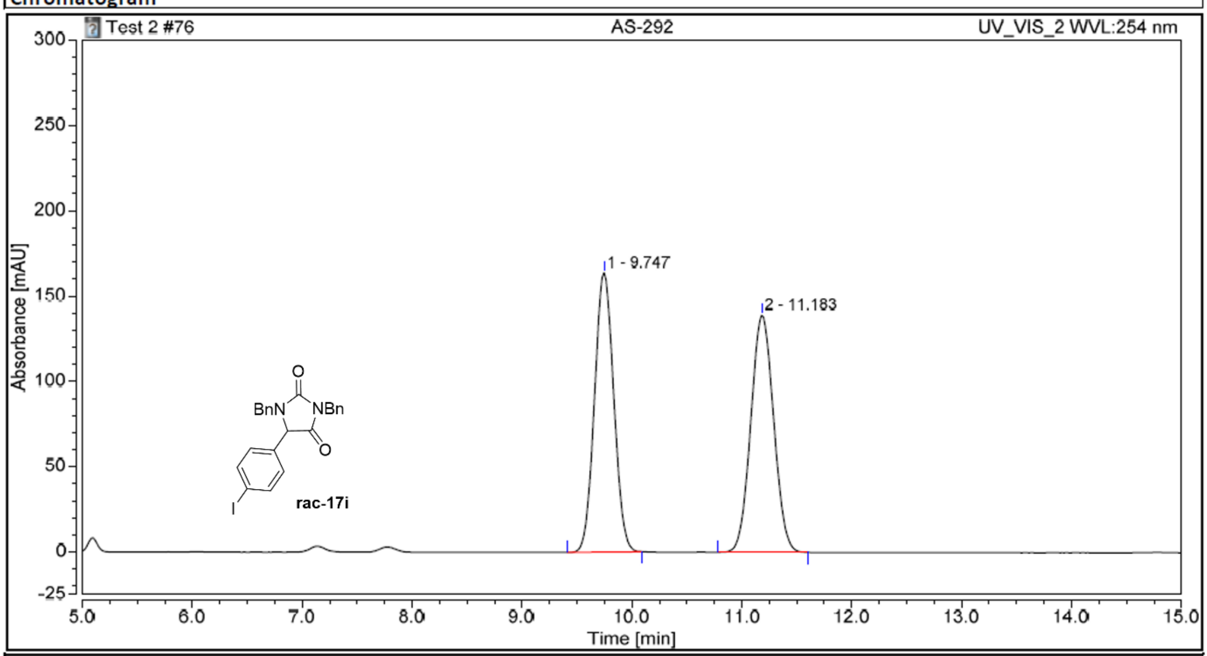

Integration Results

No.	Peak Name	Retention Time min	Area mAU*min	Height mAU	Relative Area %	Relative Height %	Amount n.a.
1		9.747	33.274	163.873	49.85	54.07	n.a.
2		11.183	33.478	139.179	50.15	45.93	n.a.
Total:			**66.752**	**303.053**	**100.00**	**100.00**	

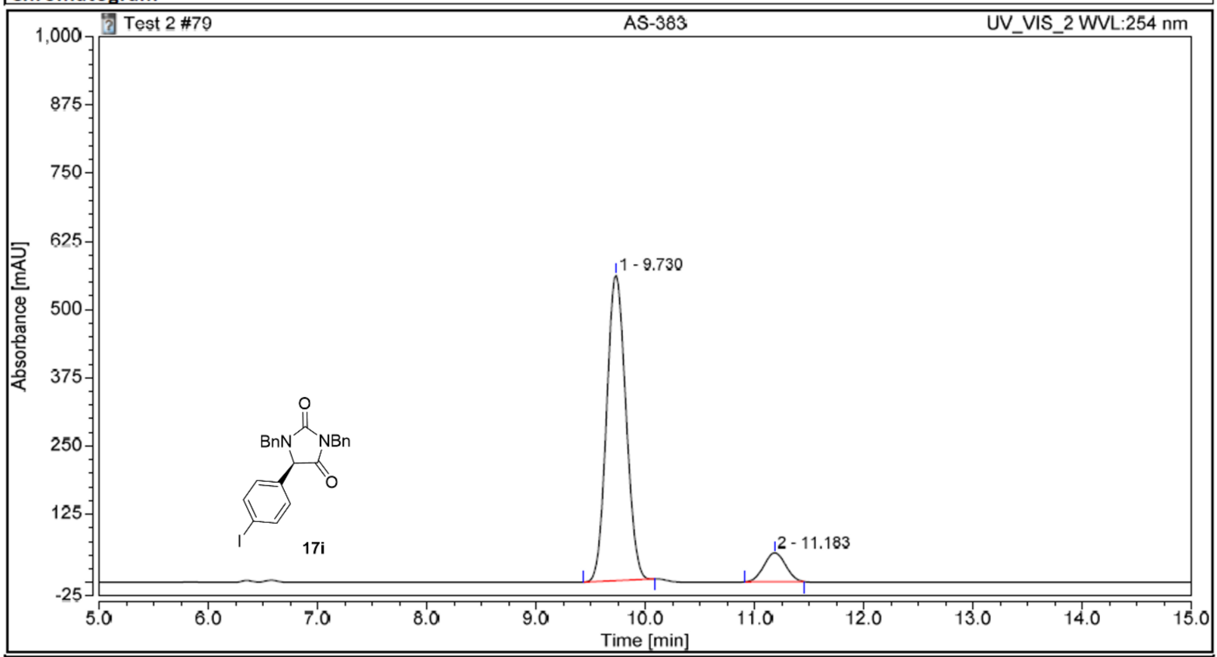

Integration Results

No.	Peak Name	Retention Time min	Area mAU*min	Height mAU	Relative Area %	Relative Height %	Amount n.a.
1		9.730	114.423	560.683	90.35	91.36	n.a.
2		11.183	12.226	53.047	9.65	8.64	n.a.
Total:			**126.649**	**613.730**	**100.00**	**100.00**	

1,3-Dibenzyl-5-(4-iodophenyl)imidazolidine-2,4-dione 17i (*scale up*)

HPLC (CHIRALPAK® AS-3R, 70:30 MeCN–H_2O, 0.5 mL/min, 254 nm)

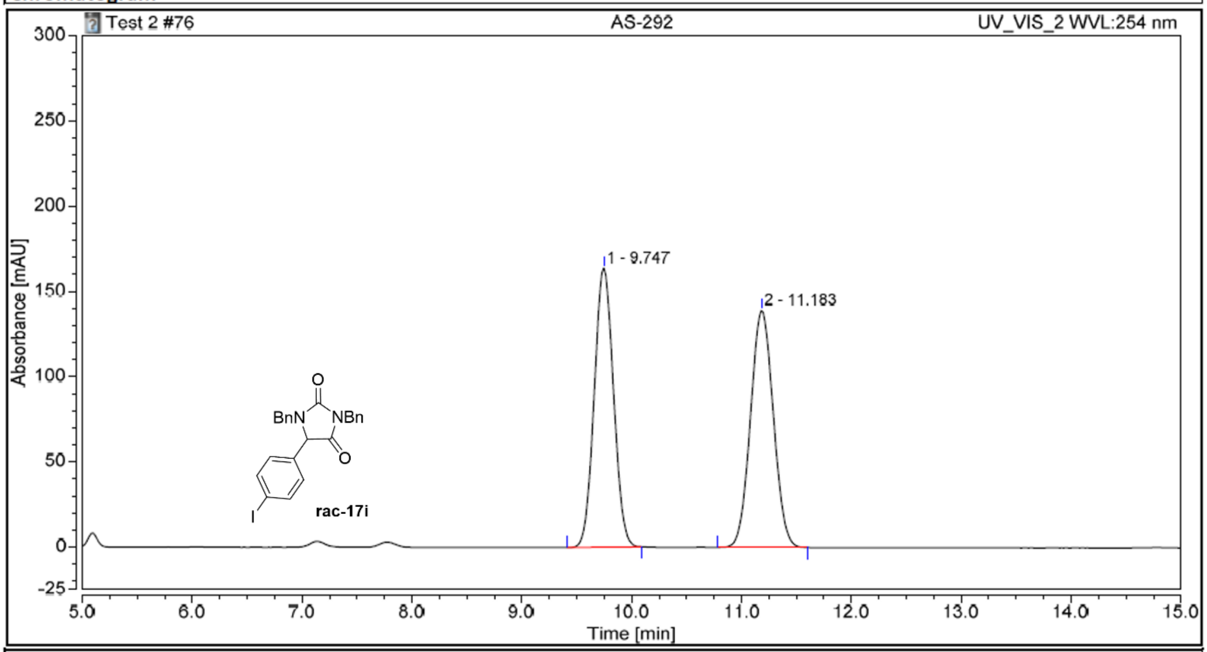

Integration Results

No.	Peak Name	Retention Time min	Area mAU*min	Height mAU	Relative Area %	Relative Height %	Amount n.a.
1		9.747	33.274	163.873	49.85	54.07	n.a.
2		11.183	33.478	139.179	50.15	45.93	n.a.
**Total:**			**66.752**	**303.053**	**100.00**	**100.00**	

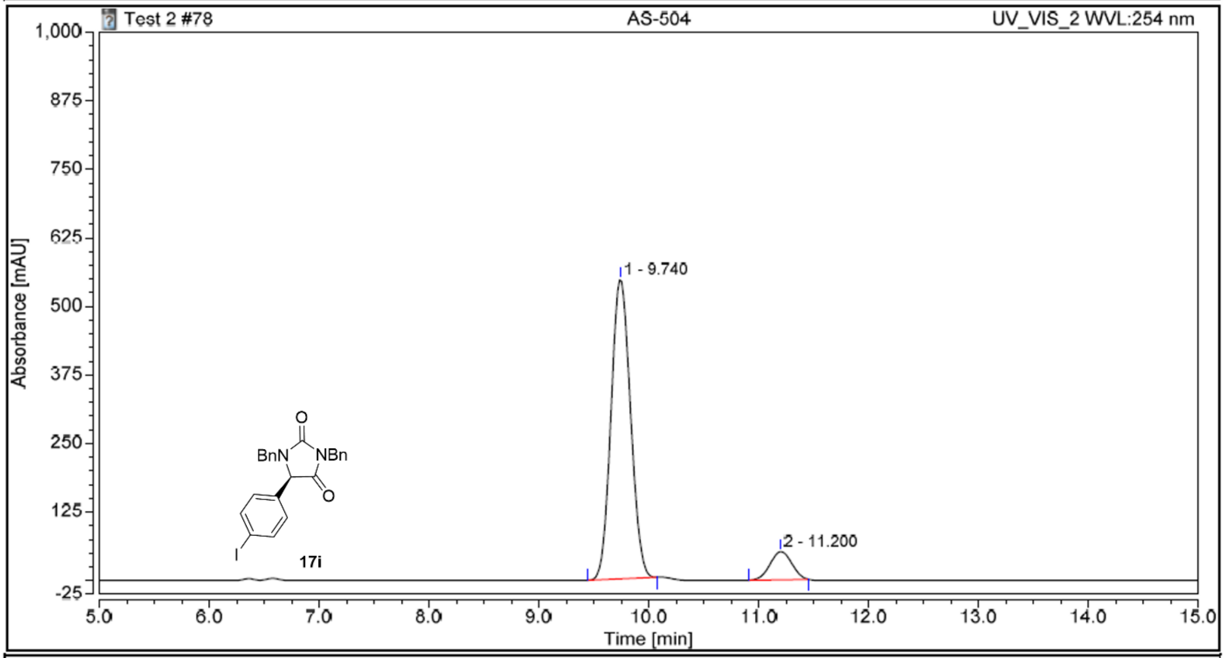

Integration Results

No.	Peak Name	Retention Time min	Area mAU*min	Height mAU	Relative Area %	Relative Height %	Amount n.a.
1		9.740	112.098	546.829	90.46	91.40	n.a.
2		11.200	11.821	51.424	9.54	8.60	n.a.
**Total:**			**123.919**	**598.253**	**100.00**	**100.00**	

1,3-Dibenzyl-5-(4-(trifluoromethyl)phenyl)imidazolidine-2,4-dione 17j (for 7 days at rt)

HPLC (Astec® Cellulose DMP, 80:20 hexane–*i*-PrOH, 0.5 mL/min, 254 nm)

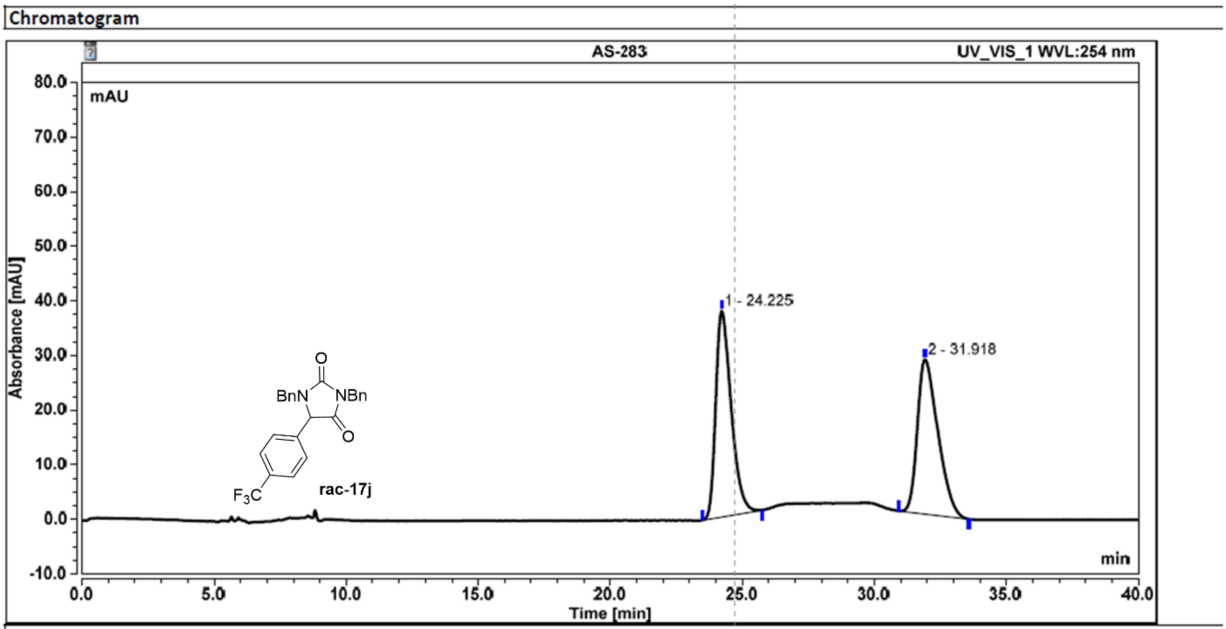

Integration Results

No.	Retention Time min	Area mAU*min	Height mAU	Relative Area %	Relative Height %
1	24.225	25.448	37.818	49.84	57.16
2	31.918	25.607	28.347	50.16	42.84
**Total:**		**51.055**	**66.165**	**100.00**	**100.00**

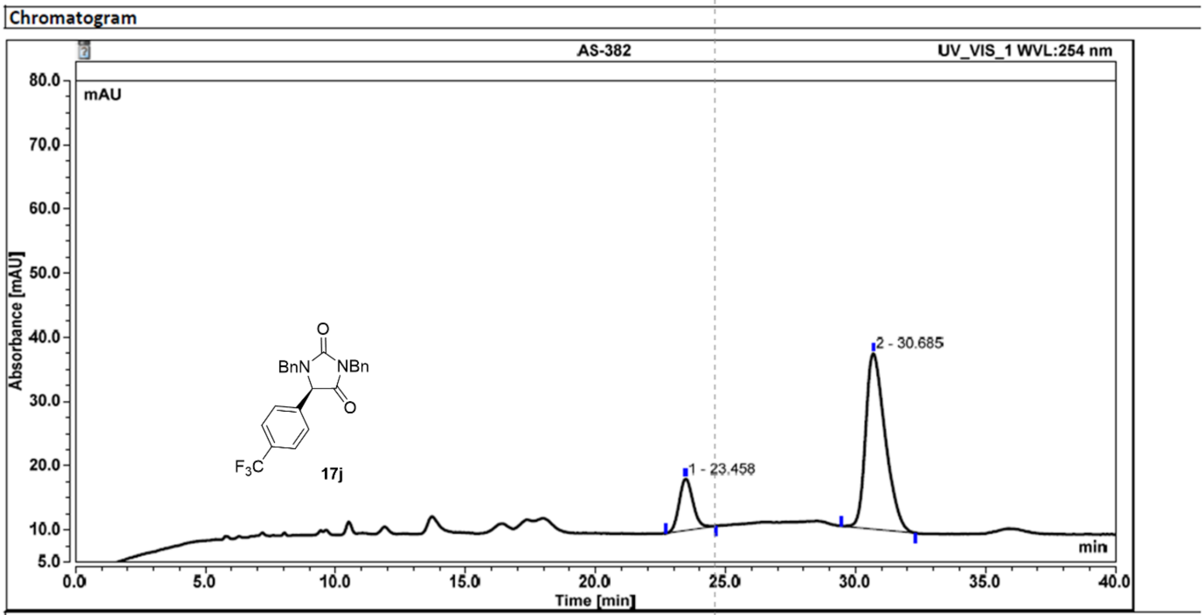

Integration Results

No.	Retention Time min	Area mAU*min	Height mAU	Relative Area %	Relative Height %
1	23.458	4.949	8.058	17.14	22.71
2	30.685	23.921	27.426	82.86	77.29
**Total:**		**28.870**	**35.485**	**100.00**	**100.00**

1,3-Dibenzyl-5-(4-(trifluoromethyl)phenyl)imidazolidine-2,4-dione 17j *(for 4.0 h at 60 ºC)*

HPLC (CHIRALPAK® AS-3R, 70:30 MeCN–H_2O, 0.5 mL/min, 254 nm)

Chromatogram

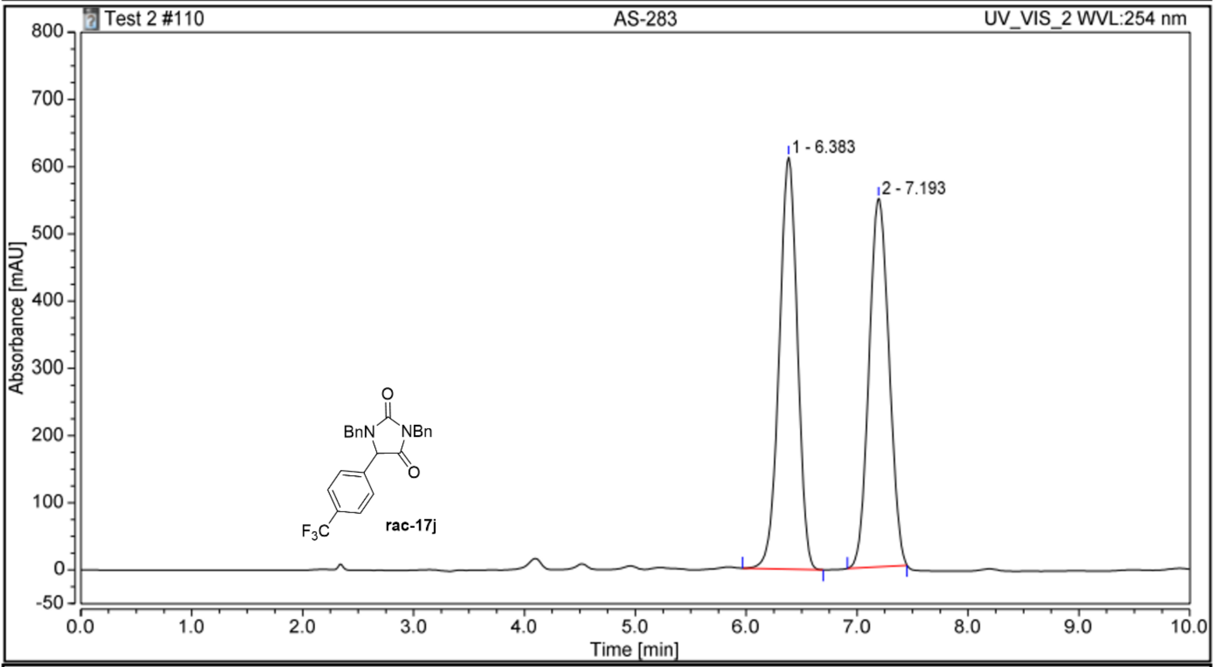

Integration Results

No.	Peak Name	Retention Time min	Area mAU*min	Height mAU	Relative Area %	Relative Height %	Amount n.a.
1		6.383	116.714	612.856	50.48	52.79	n.a.
2		7.193	114.510	548.147	49.52	47.21	n.a.
**Total:**			**231.224**	**1161.003**	**100.00**	**100.00**	

Chromatogram

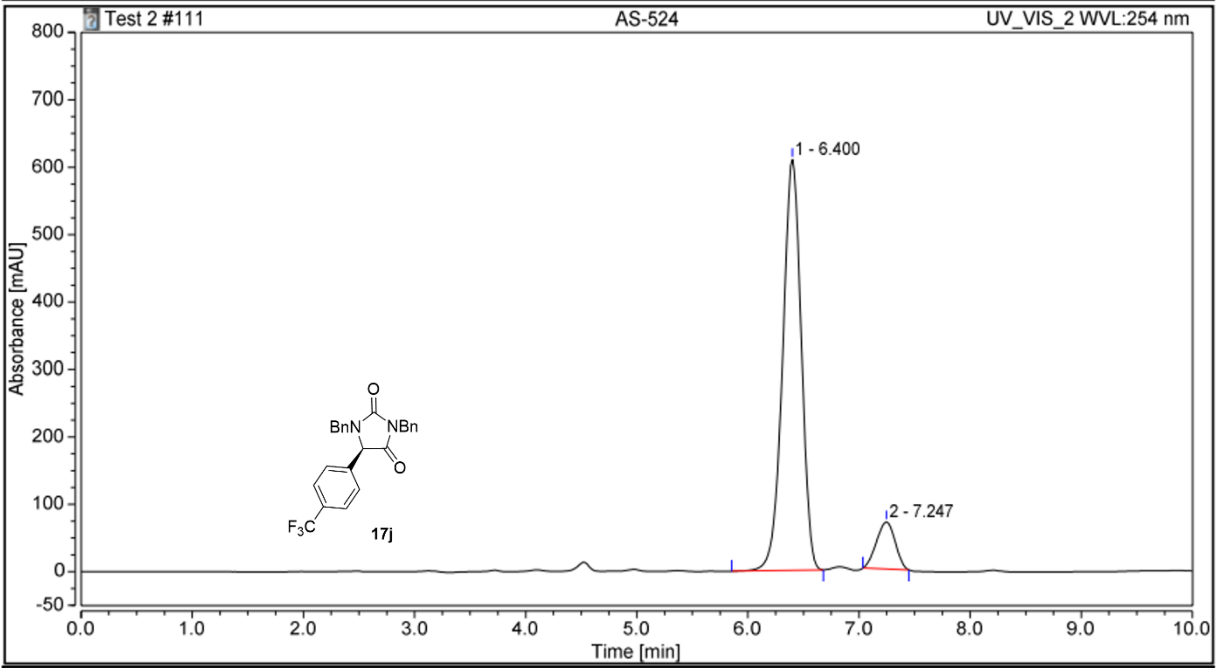

Integration Results

No.	Peak Name	Retention Time min	Area mAU*min	Height mAU	Relative Area %	Relative Height %	Amount n.a.
1		6.400	118.013	609.251	89.64	89.72	n.a.
2		7.247	13.643	69.831	10.36	10.28	n.a.
**Total:**			**131.656**	**679.083**	**100.00**	**100.00**	

1,3-Dibenzyl-5-(4-nitrophenyl)imidazolidine-2,4-dione 17k *(for 7 days at rt)*

HPLC (CHIRALPAK® AS-3R, 70:30 MeCN–H_2O, 0.5 mL/min, 254 nm)

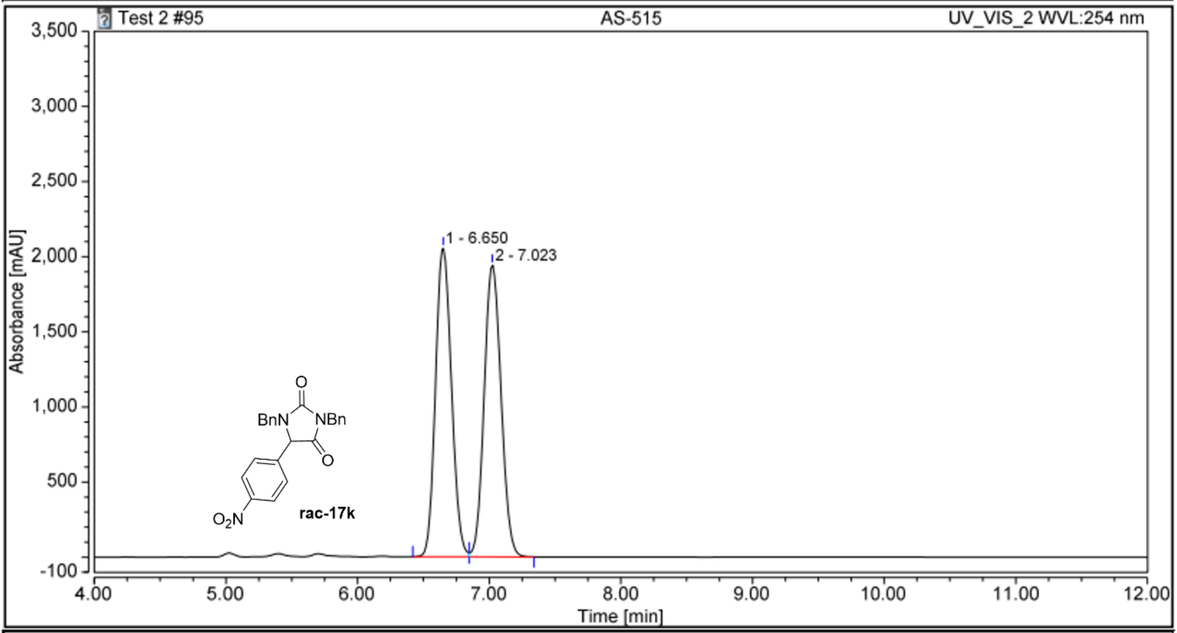

Integration Results

No.	Peak Name	Retention Time min	Area mAU*min	Height mAU	Relative Area %	Relative Height %	Amount n.a.
1		6.650	288.677	2056.778	49.91	51.43	n.a.
2		7.023	289.716	1942.729	50.09	48.57	n.a.
**Total:**			**578.393**	**3999.508**	**100.00**	**100.00**	

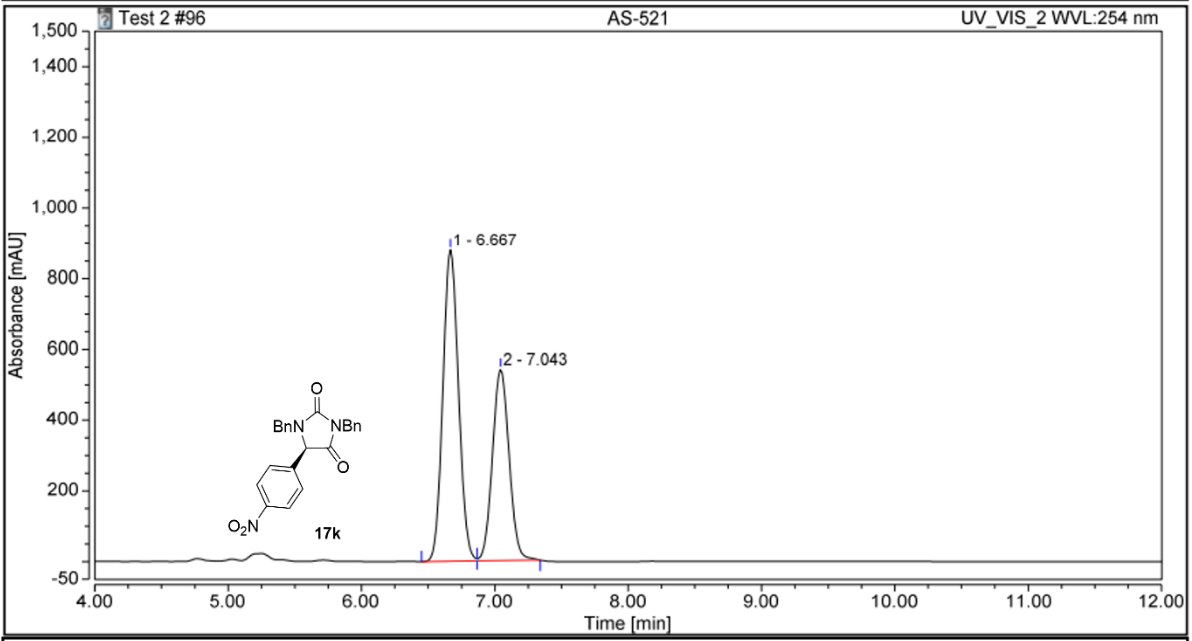

Integration Results

No.	Peak Name	Retention Time min	Area mAU*min	Height mAU	Relative Area %	Relative Height %	Amount n.a.
1		6.667	119.445	880.544	60.37	61.95	n.a.
2		7.043	78.413	540.880	39.63	38.05	n.a.
**Total:**			**197.857**	**1421.425**	**100.00**	**100.00**	

1,3-Dibenzyl-5-(4-nitrophenyl)imidazolidine-2,4-dione 17k (at 60 °C for 4 h)

HPLC (CHIRALPAK® AS-3R, 70:30 MeCN–H_2O, 0.5 mL/min, 254 nm)

Chromatogram

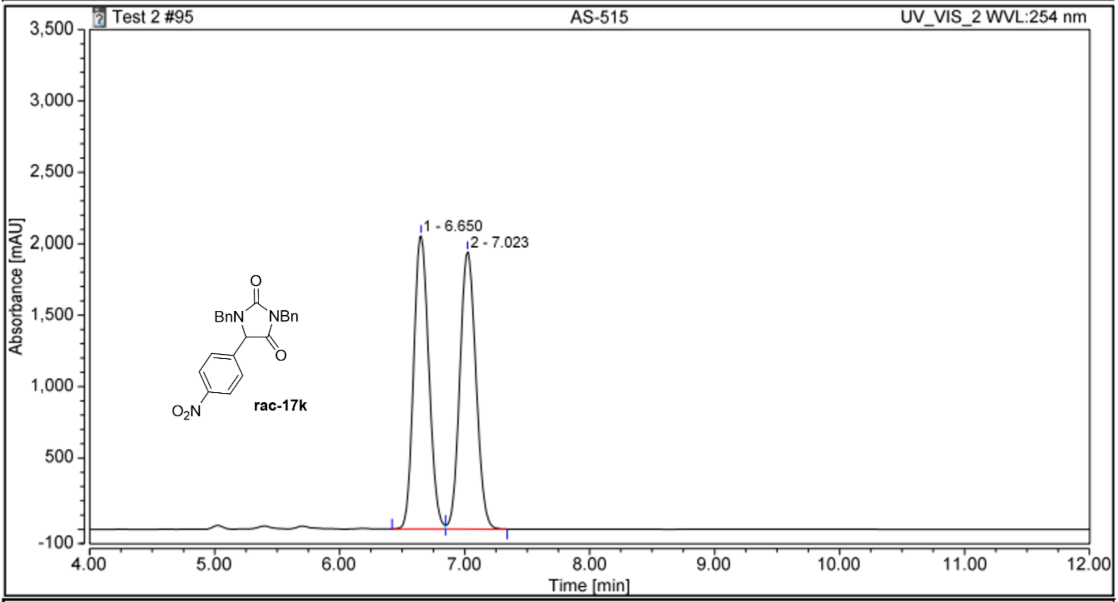

Integration Results

No.	Peak Name	Retention Time min	Area mAU*min	Height mAU	Relative Area %	Relative Height %	Amount n.a.
1		6.650	288.677	2056.778	49.91	51.43	n.a.
2		7.023	289.716	1942.729	50.09	48.57	n.a.
**Total:**			**578.393**	**3999.508**	**100.00**	**100.00**	

Chromatogram

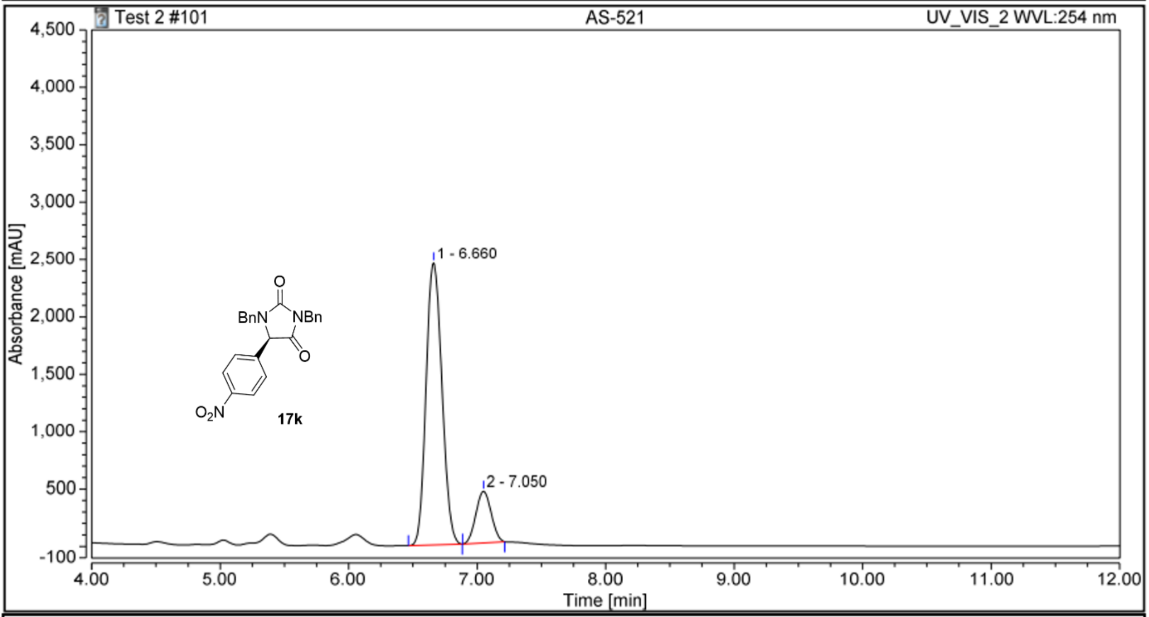

Integration Results

No.	Peak Name	Retention Time min	Area mAU*min	Height mAU	Relative Area %	Relative Height %	Amount n.a.
1		6.660	351.942	2456.530	85.00	84.52	n.a.
2		7.050	62.112	449.974	15.00	15.48	n.a.
**Total:**			**414.054**	**2906.505**	**100.00**	**100.00**	

1,3-Dibenzyl-5-(3-methoxyphenyl)imidazolidine-2,4-dione, 17l

HPLC (Astec® Cellulose DMP, 80:20 hexane–EtOH, 1 mL/min, 280 nm)

Chromatogram

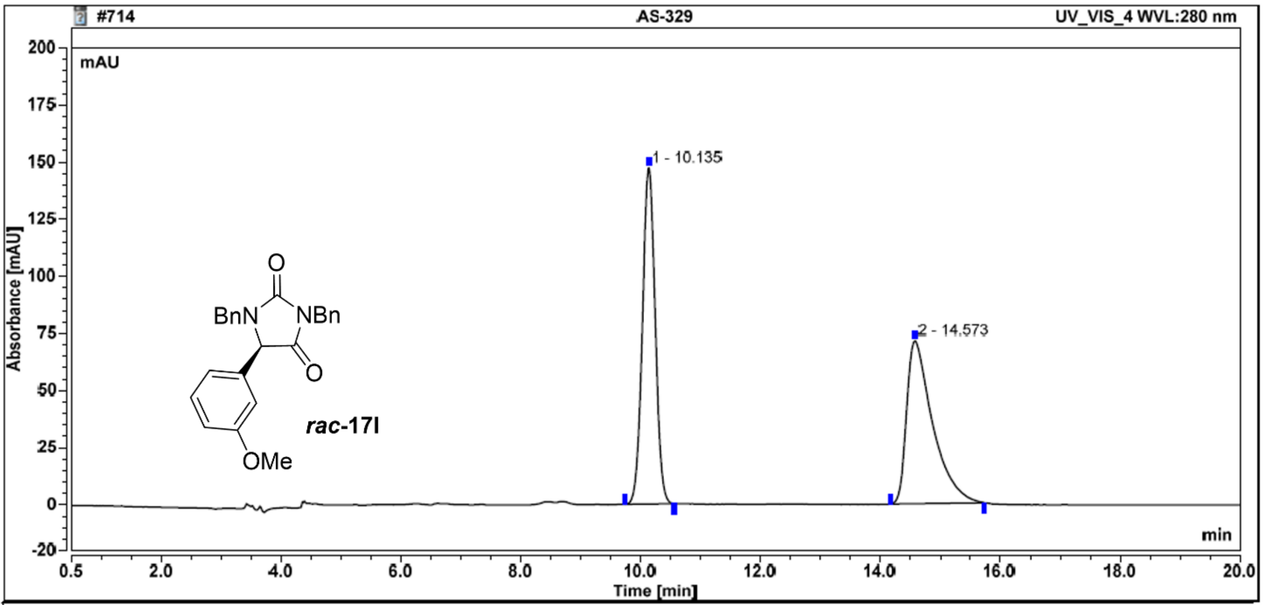

Integration Results

No.	Retention Time min	Area mAU*min	Height mAU	Relative Area %	Relative Height %
1	10.135	36.159	147.158	50.30	67.34
2	14.573	35.723	71.373	49.70	32.66
**Total:**		**71.882**	**218.531**	**100.00**	**100.00**

Chromatogram

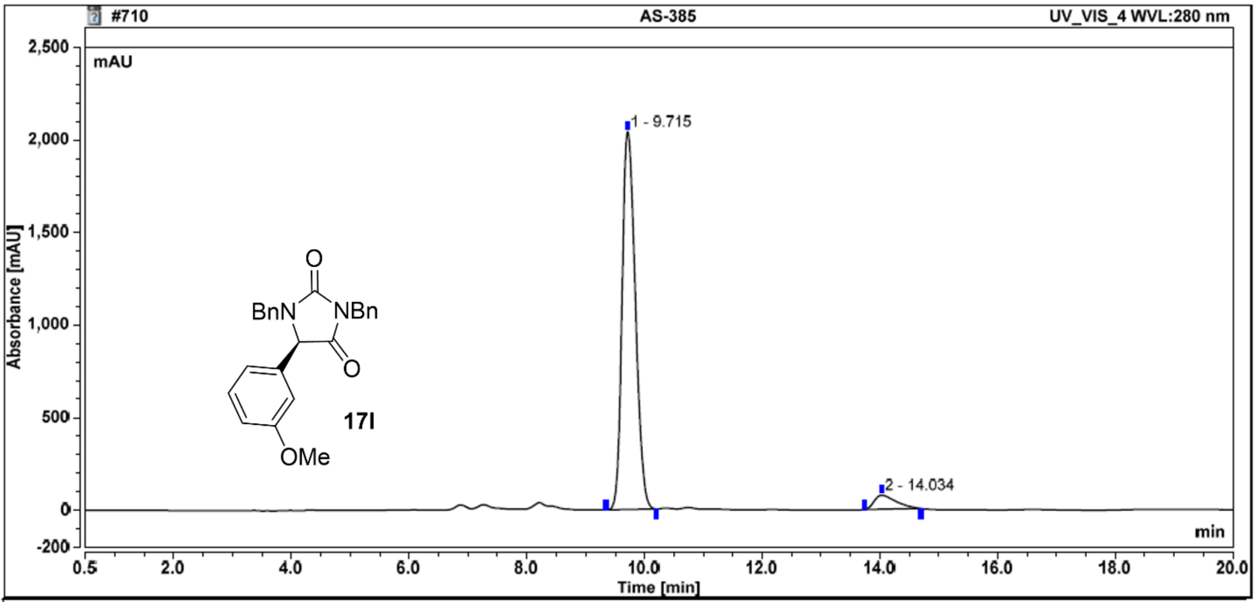

Integration Results

No.	Retention Time min	Area mAU*min	Height mAU	Relative Area %	Relative Height %
1	9.715	530.569	2041.149	94.21	96.42
2	14.034	32.627	75.810	5.79	3.58
**Total:**		**563.197**	**2116.959**	**100.00**	**100.00**

1,3-Dibenzyl-5-(3-chlorophenyl)imidazolidine-2,4-dione 17m

HPLC (Astec® Cellulose DMP, 80:20 hexane–EtOH, 1 mL/min, 254 nm)

Chromatogram

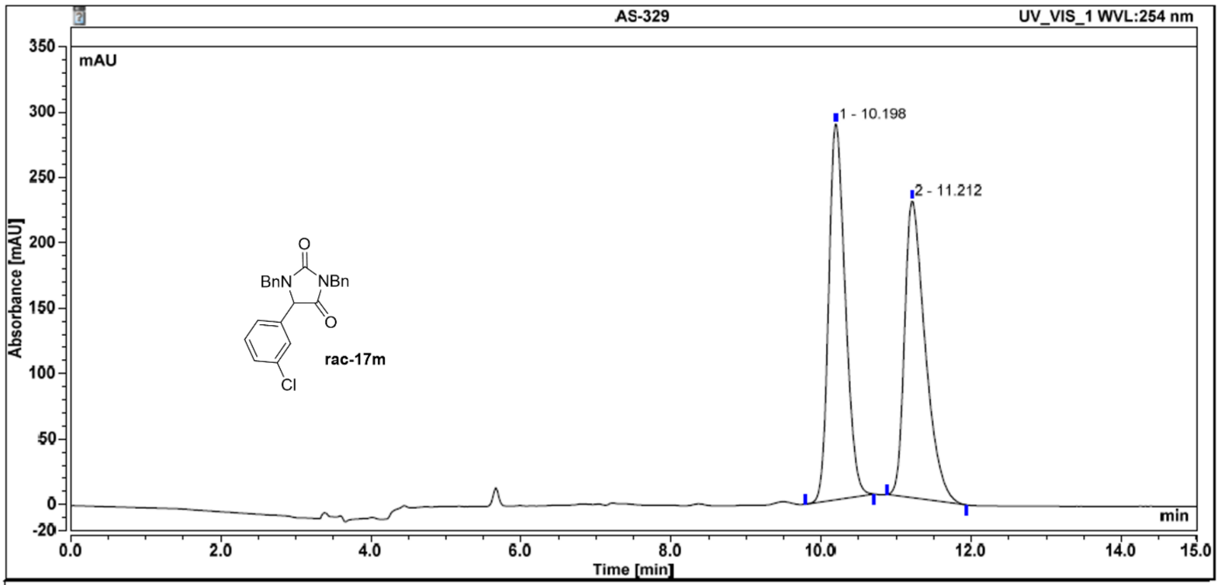

Integration Results

No.	Retention Time min	Area mAU*min	Height mAU	Relative Area %	Relative Height %
1	10.198	73.491	287.553	50.39	55.85
2	11.212	72.356	227.283	49.61	44.15
**Total:**		**145.847**	**514.836**	**100.00**	**100.00**

Chromatogram

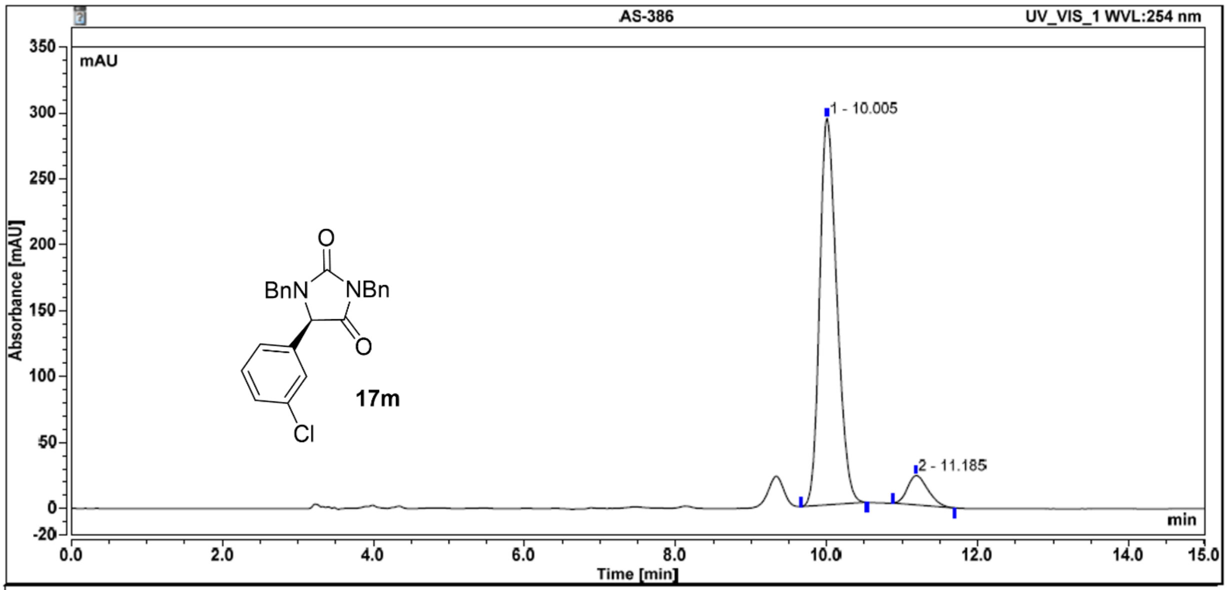

Integration Results

No.	Retention Time min	Area mAU*min	Height mAU	Relative Area %	Relative Height %
1	10.005	77.639	293.356	91.75	92.94
2	11.185	6.980	22.272	8.25	7.06
**Total:**		**84.620**	**315.628**	**100.00**	**100.00**

1,3-Dibenzyl-5-(3,4-difluorophenyl)imidazolidine-2,4-dione, 17n

HPLC (CHIRALPAK® AS-3R, 50:50 MeCN–H_2O, 0.5 mL/min, 254 nm)

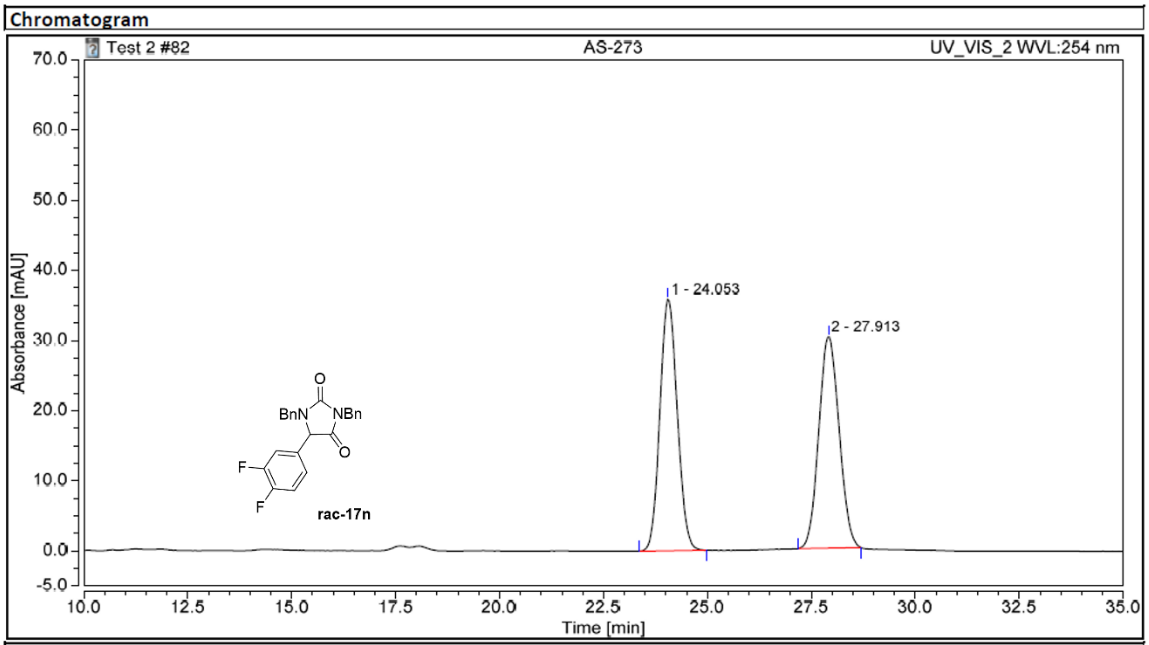

Integration Results

No.	Peak Name	Retention Time min	Area mAU*min	Height mAU	Relative Area %	Relative Height %	Amount n.a.
1		24.053	17.357	35.950	50.41	54.33	n.a.
2		27.913	17.073	30.226	49.59	45.67	n.a.
**Total:**			**34.430**	**66.176**	**100.00**	**100.00**	

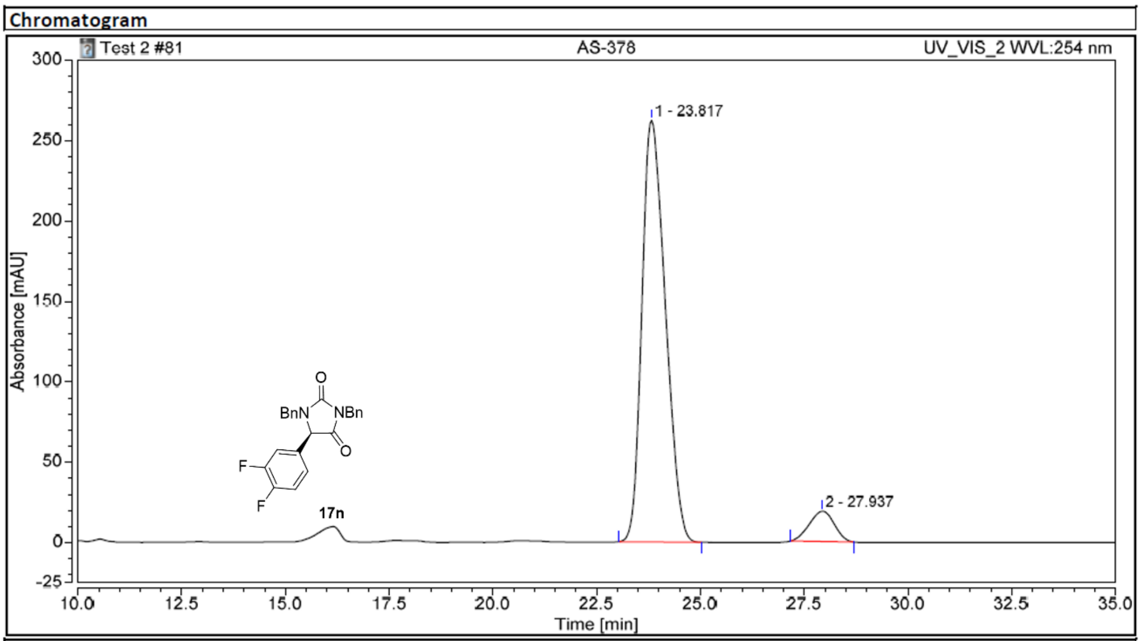

Integration Results

No.	Peak Name	Retention Time min	Area mAU*min	Height mAU	Relative Area %	Relative Height %	Amount n.a.
1		23.817	171.410	262.363	92.90	93.29	n.a.
2		27.937	13.106	18.873	7.10	6.71	n.a.
**Total:**			**184.516**	**281.236**	**100.00**	**100.00**	

1,3-Dibenzyl-5-(3,4-difluorophenyl)imidazolidine-2,4-dione, 17n (*scale up*)

HPLC (CHIRALPAK® AS-3R, 50:50 MeCN–H_2O, 0.5 mL/min, 254 nm)

Chromatogram

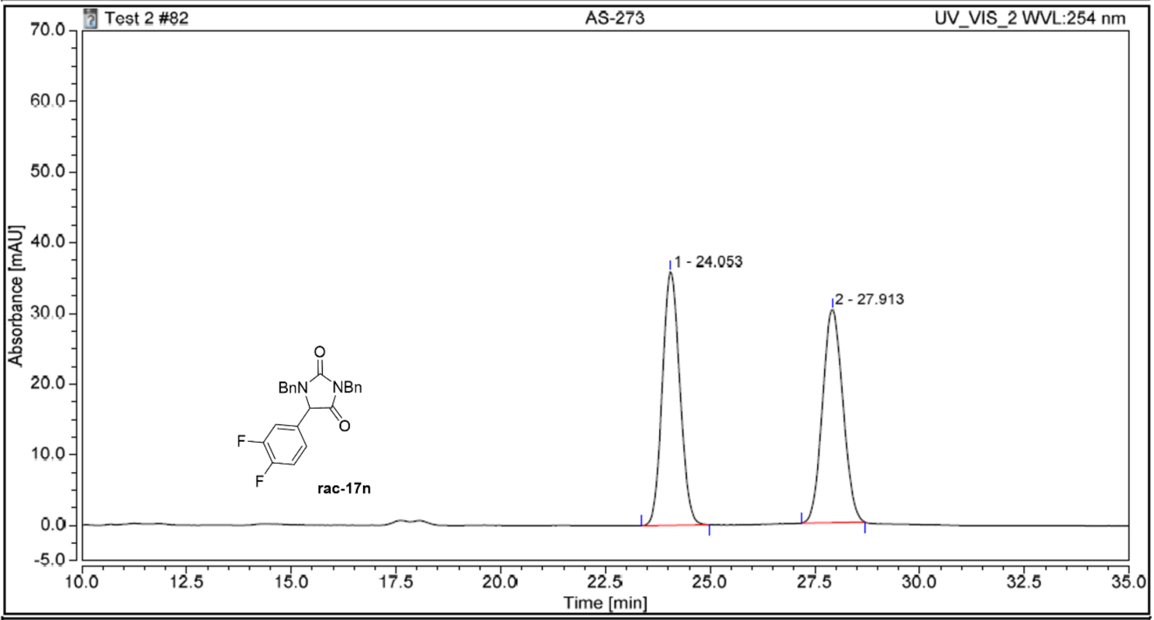

Integration Results

No.	Peak Name	Retention Time min	Area mAU*min	Height mAU	Relative Area %	Relative Height %	Amount n.a.
1		24.053	17.357	35.950	50.41	54.33	n.a.
2		27.913	17.073	30.226	49.59	45.67	n.a.
**Total:**			**34.430**	**66.176**	**100.00**	**100.00**	

Chromatogram

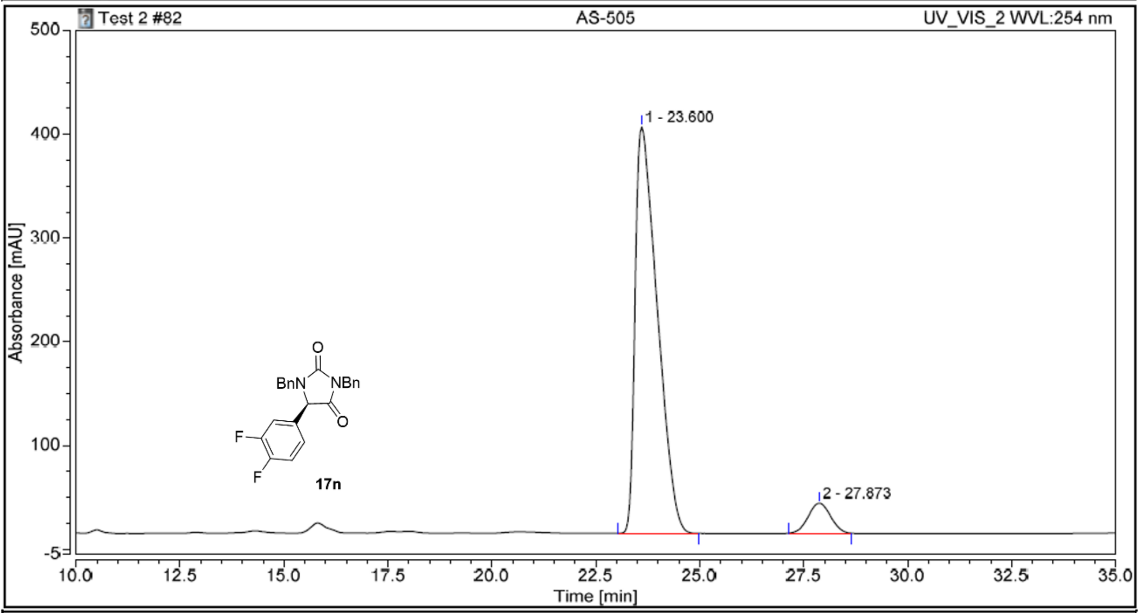

Integration Results

No.	Peak Name	Retention Time min	Area mAU*min	Height mAU	Relative Area %	Relative Height %	Amount n.a.
1		23.600	240.835	391.782	93.05	93.10	n.a.
2		27.873	17.995	29.057	6.95	6.90	n.a.
**Total:**			**258.829**	**420.839**	**100.00**	**100.00**	

1,3-Dibenzyl-5-(2-methoxy)phenyl)imidazolidine-2,4-dione 17o

HPLC (CHIRALPAK® AS-3R, 50:50 MeCN–H_2O, 0.5 mL/min, 280 nm)

Chromatogram

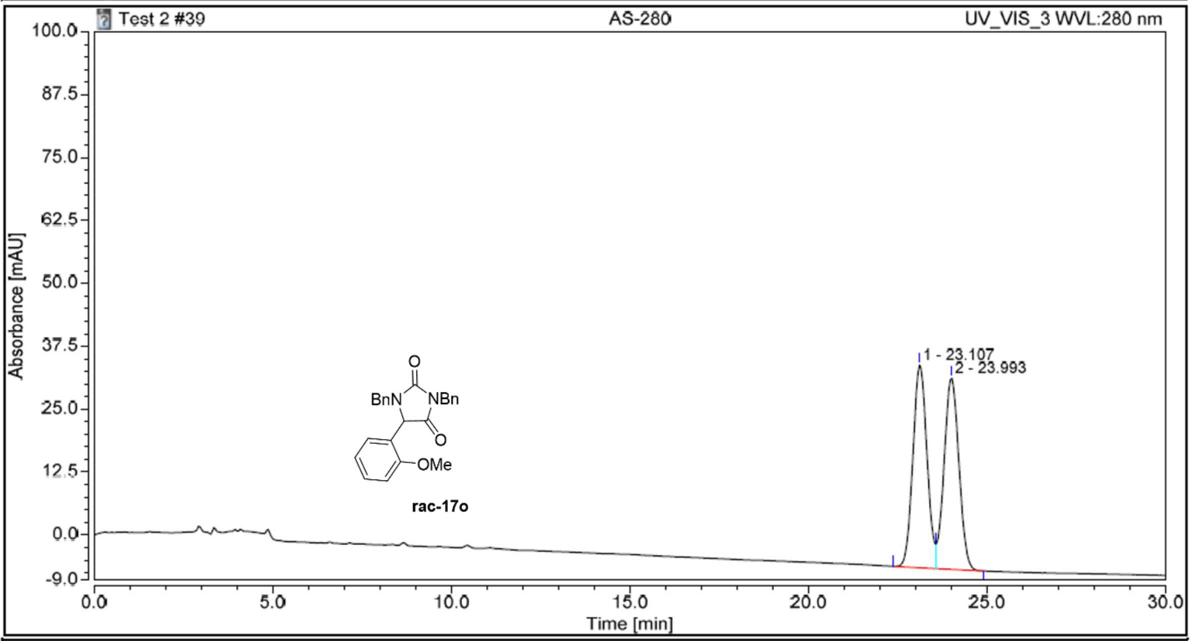

Integration Results

No.	Peak Name	Retention Time min	Area mAU*min	Height mAU	Relative Area %	Relative Height %	Amount n.a.
1		23.107	18.304	40.359	49.94	51.44	n.a.
2		23.993	18.351	38.097	50.06	48.56	n.a.
**Total:**			**36.654**	**78.456**	**100.00**	**100.00**	

Chromatogram

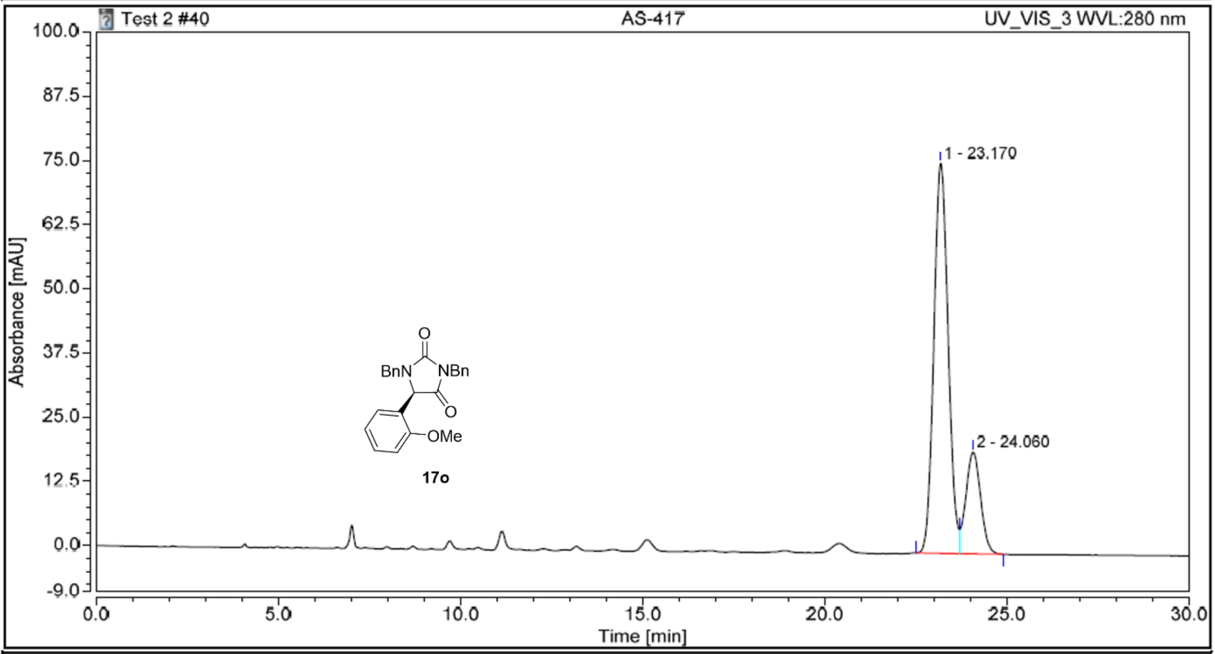

Integration Results

No.	Peak Name	Retention Time min	Area mAU*min	Height mAU	Relative Area %	Relative Height %	Amount n.a.
1		23.170	34.427	75.999	78.61	79.28	n.a.
2		24.060	9.368	19.867	21.39	20.72	n.a.
**Total:**			**43.795**	**95.866**	**100.00**	**100.00**	

1,3-Dibenzyl-5-(2-chlorophenyl)imidazolidine-2,4-dione 17p

HPLC (CHIRALPAK® AS-3R, 60:40 MeCN–H_2O, 0.5 mL/min, 220 nm)

Chromatogram

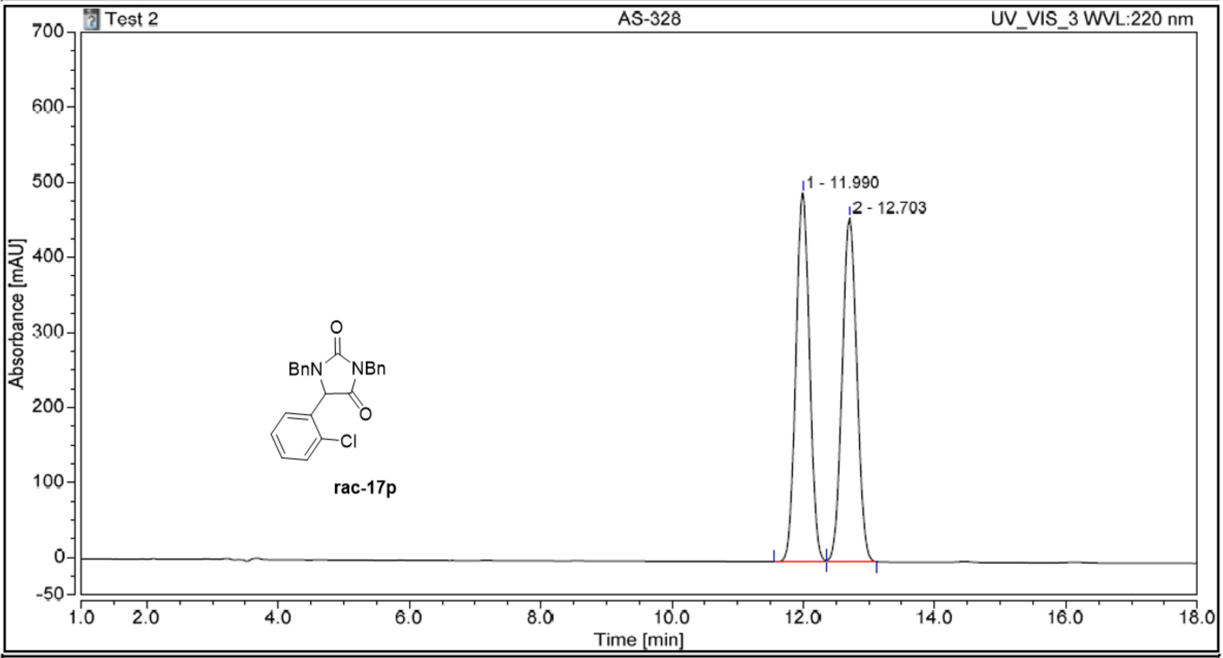

Integration Results

No.	Peak Name	Retention Time min	Area mAU*min	Height mAU	Relative Area %	Relative Height %	Amount n.a.
1		11.990	118.542	492.123	49.96	51.77	n.a.
2		12.703	118.748	458.508	50.04	48.23	n.a.
**Total:**			**237.289**	**950.631**	**100.00**	**100.00**	

Chromatogram

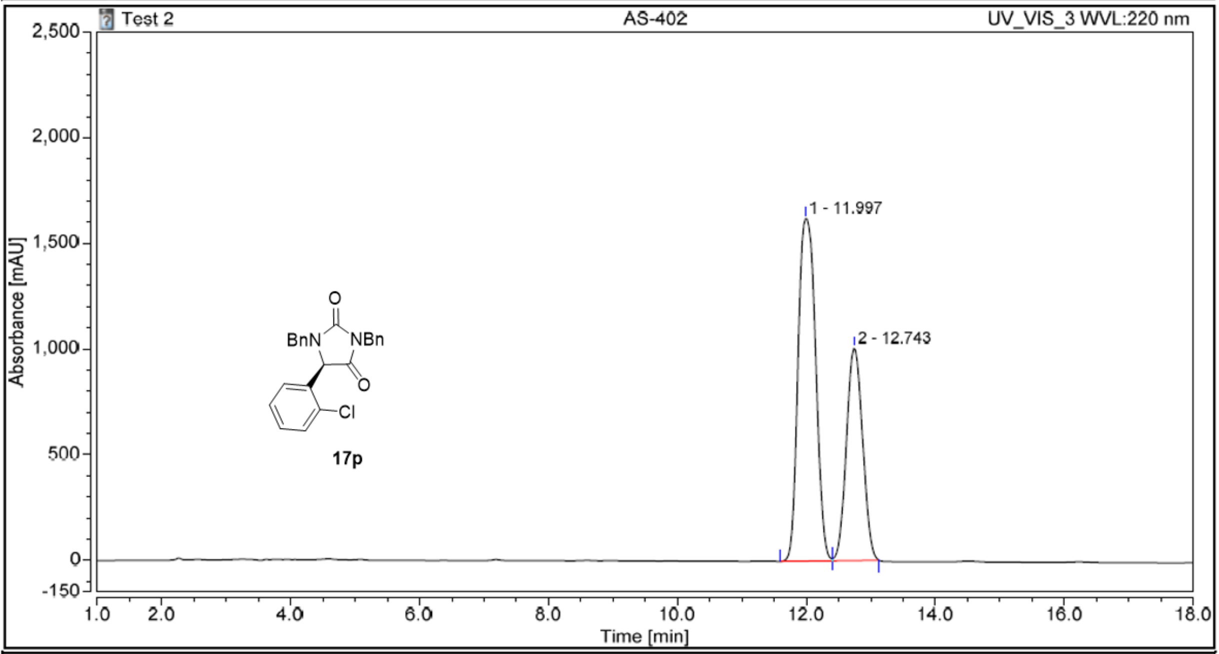

Integration Results

No.	Peak Name	Retention Time min	Area mAU*min	Height mAU	Relative Area %	Relative Height %	Amount n.a.
1		11.997	508.557	1623.151	63.52	61.73	n.a.
2		12.743	292.091	1006.169	36.48	38.27	n.a.
**Total:**			**800.648**	**2629.320**	**100.00**	**100.00**	

1,3-Dibenzyl-5-(2-chlorophenyl)imidazolidine-2,4-dione 17p (at 60 ºC for 4 h).

HPLC (CHIRALPAK® AS-3R, 60:40 MeCN–H_2O, 0.5 mL/min, 220 nm)

Chromatogram

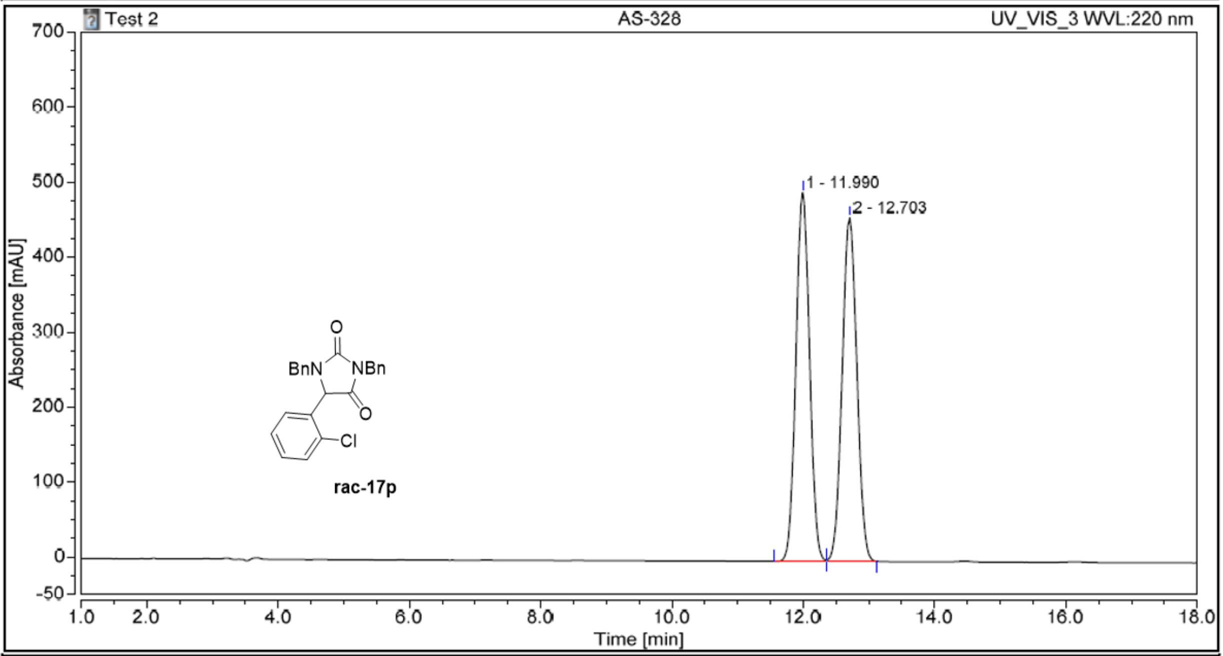

Integration Results

No.	Peak Name	Retention Time min	Area mAU*min	Height mAU	Relative Area %	Relative Height %	Amount n.a.
1		11.990	118.542	492.123	49.96	51.77	n.a.
2		12.703	118.748	458.508	50.04	48.23	n.a.
**Total:**			**237.289**	**950.631**	**100.00**	**100.00**	

Chromatogram

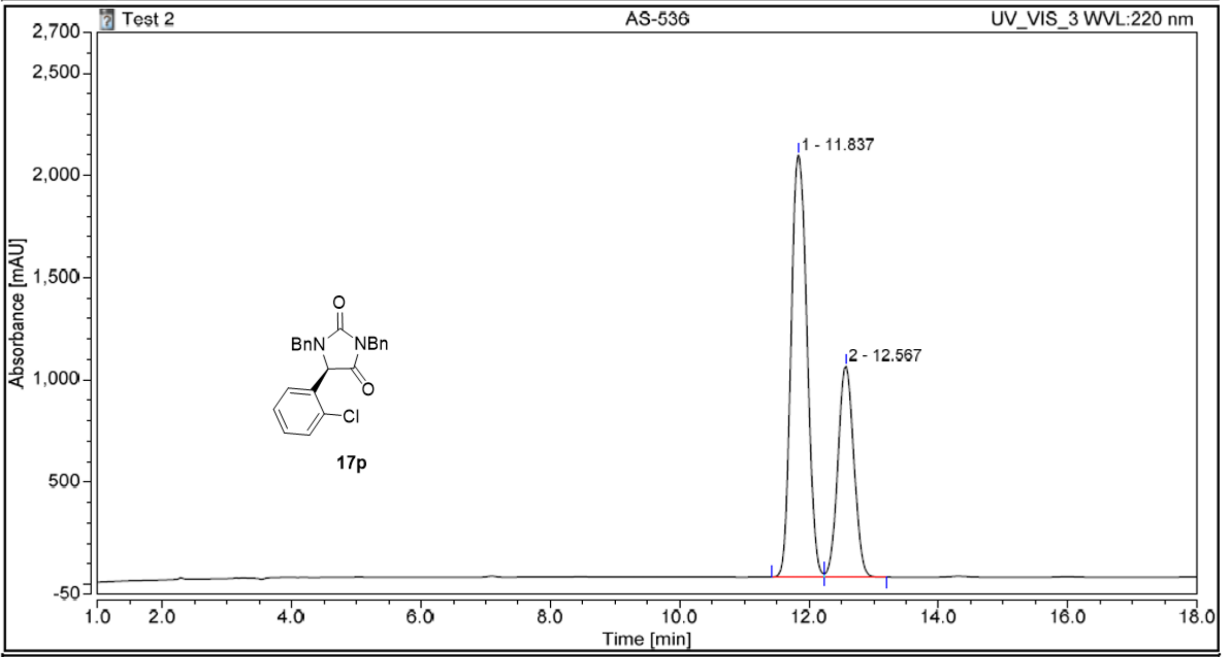

Integration Results

No.	Peak Name	Retention Time min	Area mAU*min	Height mAU	Relative Area %	Relative Height %	Amount n.a.
1		11.837	589.012	2064.689	66.67	66.63	n.a.
2		12.567	294.412	1033.940	33.33	33.37	n.a.
**Total:**			**883.425**	**3098.629**	**100.00**	**100.00**	

1,3-Dibenzyl-5-(2-(trifluoromethyl)phenyl)imidazolidine-2,4-dione 17q

HPLC (Astec® Cellulose DMP, 80:20 hexane–EtOH, 1 mL/min, 220 nm)

Chromatogram

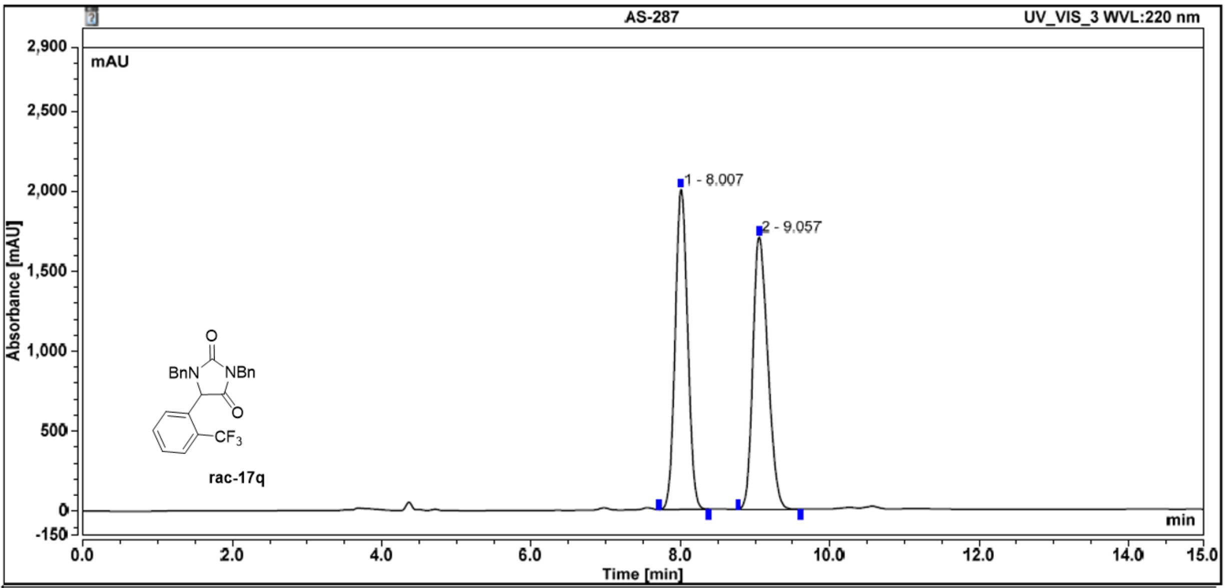

Integration Results

No.	Retention Time min	Area mAU*min	Height mAU	Relative Area %	Relative Height %
1	8.007	386.096	2001.096	49.48	54.02
2	9.057	394.237	1703.419	50.52	45.98
**Total:**		**780.333**	**3704.515**	**100.00**	**100.00**

Chromatogram

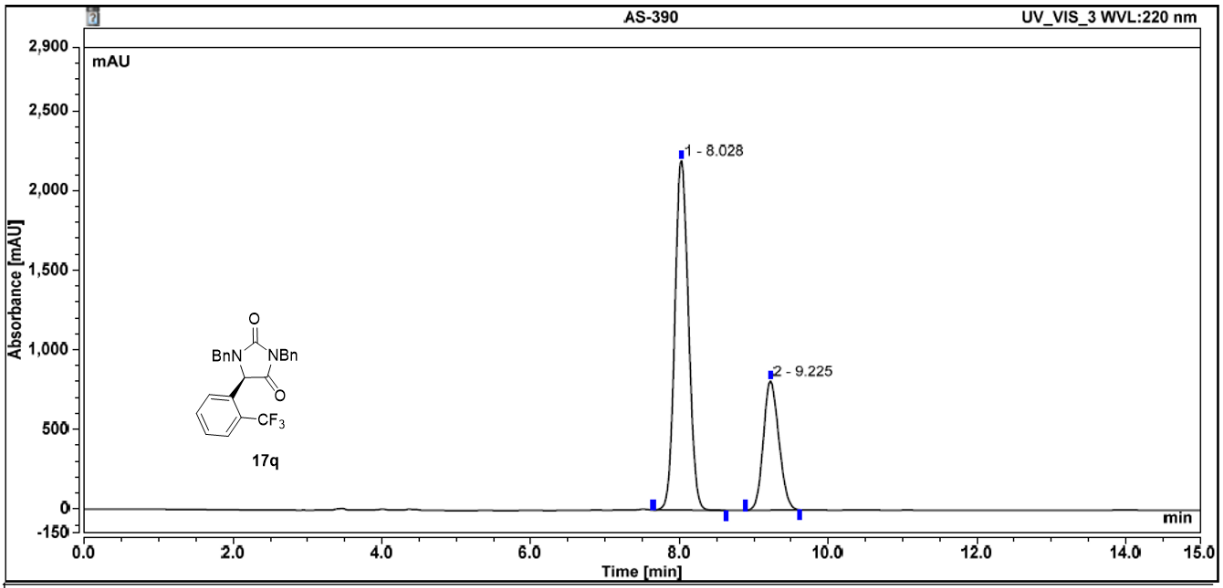

Integration Results

No.	Retention Time min	Area mAU*min	Height mAU	Relative Area %	Relative Height %
1	8.028	465.623	2197.415	70.32	73.03
2	9.225	196.530	811.681	29.68	26.97
**Total:**		**662.152**	**3009.096**	**100.00**	**100.00**

1,3-Dibenzyl-5-(naphthalen-1-yl)imidazolidine-2,4-dione 17r

HPLC (Astec® Cellulose DMP, 80:20 hexane–EtOH, 1 mL/min, 254 nm)

Chromatogram

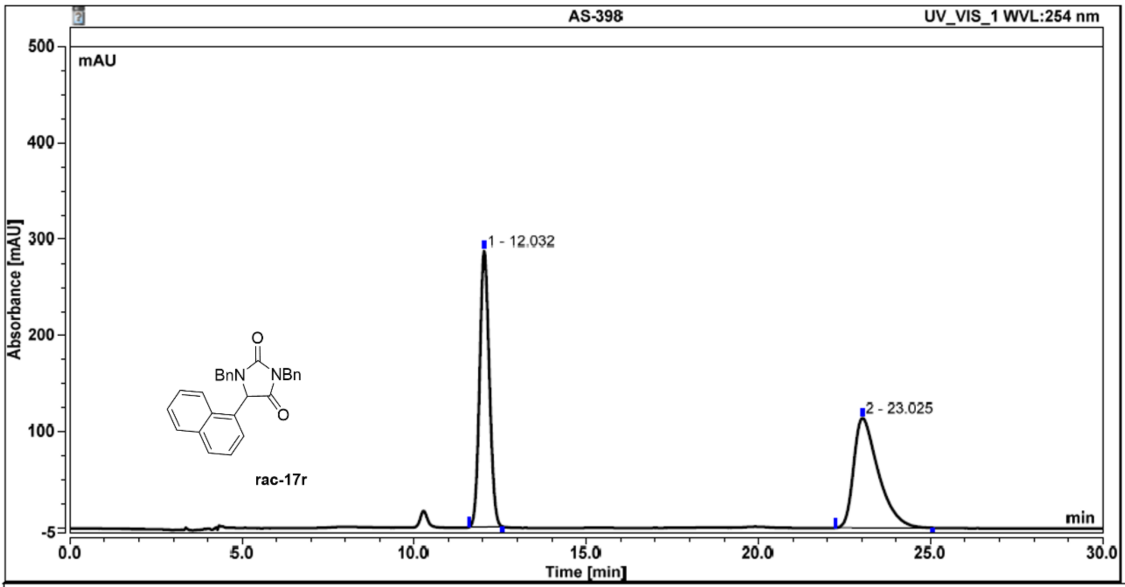

Integration Results

No.	Retention Time min	Area mAU*min	Height mAU	Relative Area %	Relative Height %
1	12.032	93.198	286.635	49.64	71.53
2	23.025	94.562	114.103	50.36	28.47
**Total:**		**187.759**	**400.739**	**100.00**	**100.00**

Chromatogram

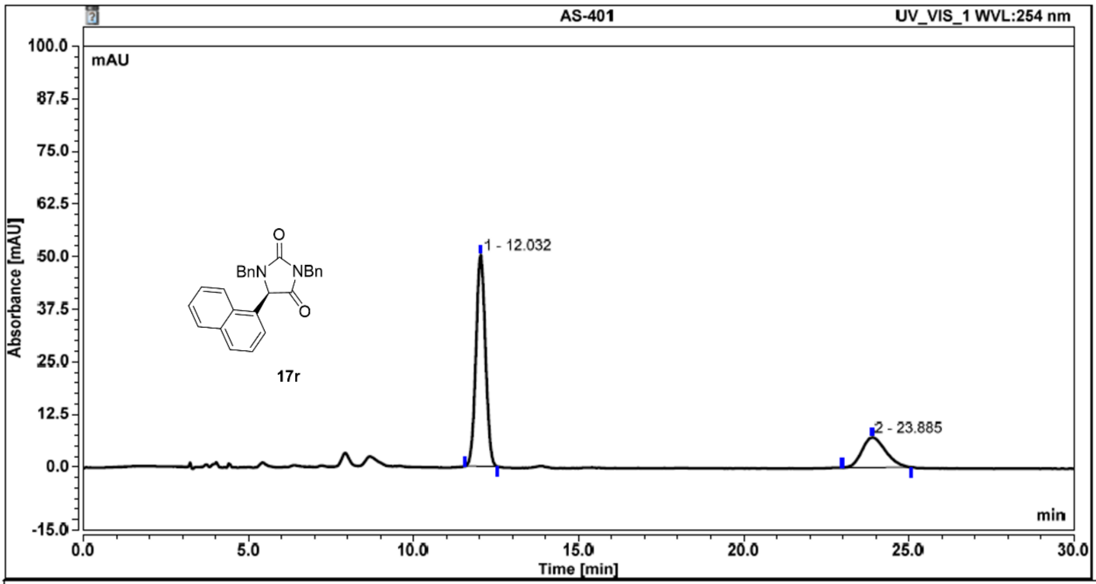

Integration Results

No.	Retention Time min	Area mAU*min	Height mAU	Relative Area %	Relative Height %
1	12.032	16.732	50.284	73.75	87.53
2	23.885	5.954	7.163	26.25	12.47
**Total:**		**22.686**	**57.447**	**100.00**	**100.00**

1,3-Dibenzyl-5-(naphthalen-2-yl)imidazolidine-2,4-dione 17s

HPLC (Astec® Cellulose DMP, 80:20 hexane–EtOH, 1 mL/min, 254 nm)

Chromatogram

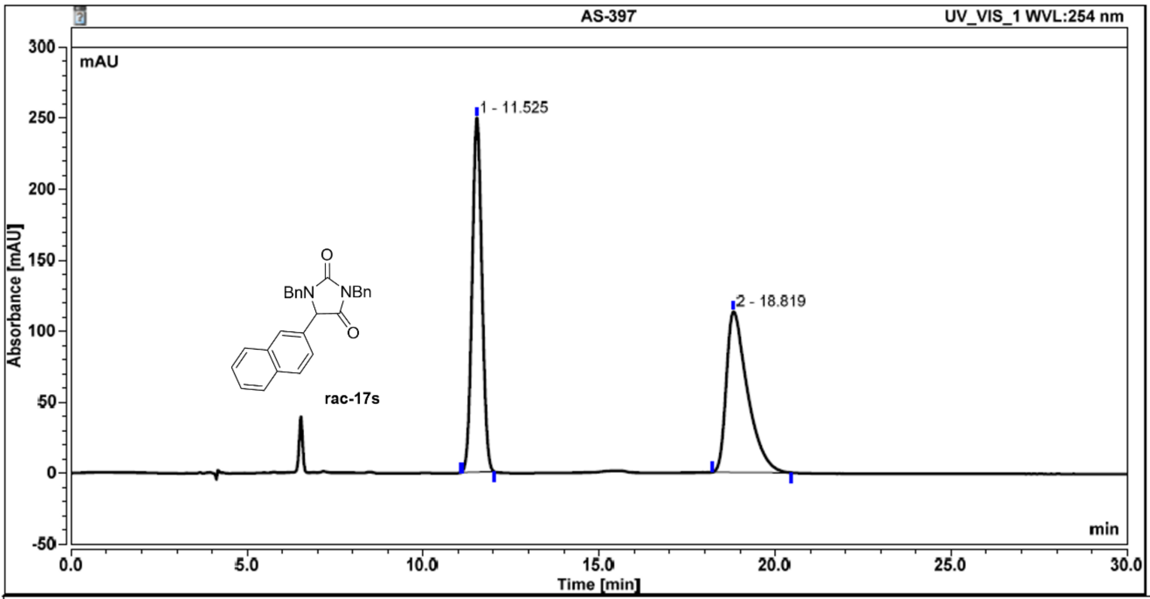

Integration Results

No.	Retention Time<br>min	Area<br>mAU*min	Height<br>mAU	Relative Area<br>%	Relative Height<br>%
1	11.525	79.830	249.522	50.03	68.77
2	18.819	79.748	113.288	49.97	31.23
**Total:**		**159.578**	**362.810**	**100.00**	**100.00**

Chromatogram

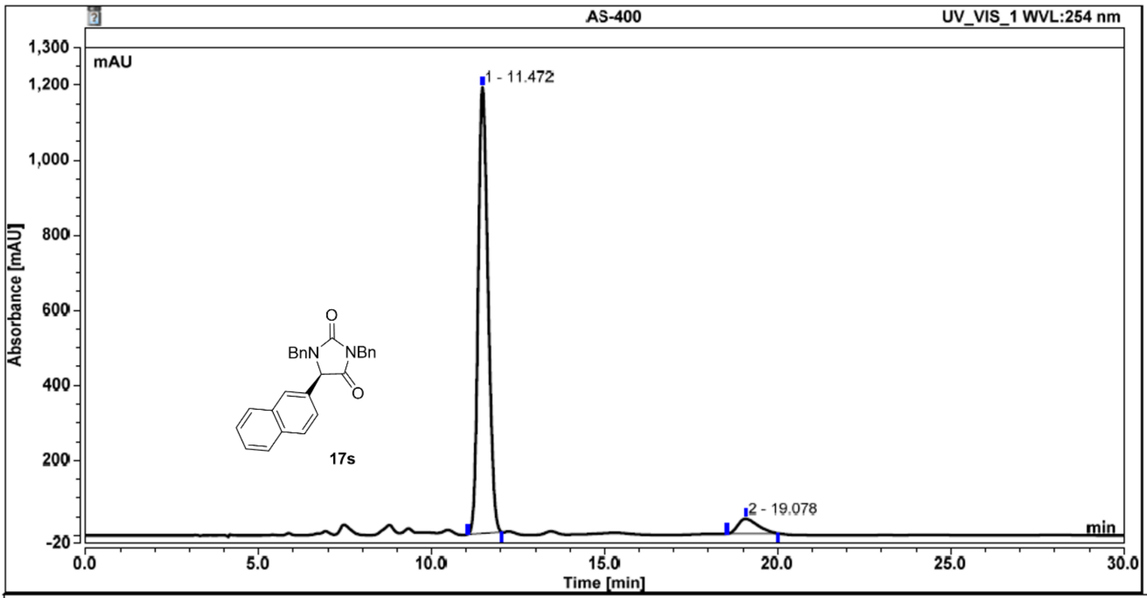

Integration Results

No.	Retention Time<br>min	Area<br>mAU*min	Height<br>mAU	Relative Area<br>%	Relative Height<br>%
1	11.472	391.131	1189.441	93.68	96.76
2	19.078	26.390	39.787	6.32	3.24
**Total:**		**417.521**	**1229.228**	**100.00**	**100.00**

1,3-Dibenzyl-5-(5-bromothiophen-2-yl)imidazolidine-2,4-dione 17t

HPLC (CHIRALPAK® AS-3R, 80:20 MeCN–H_2O, 0.5 mL/min, 254 nm)

Chromatogram

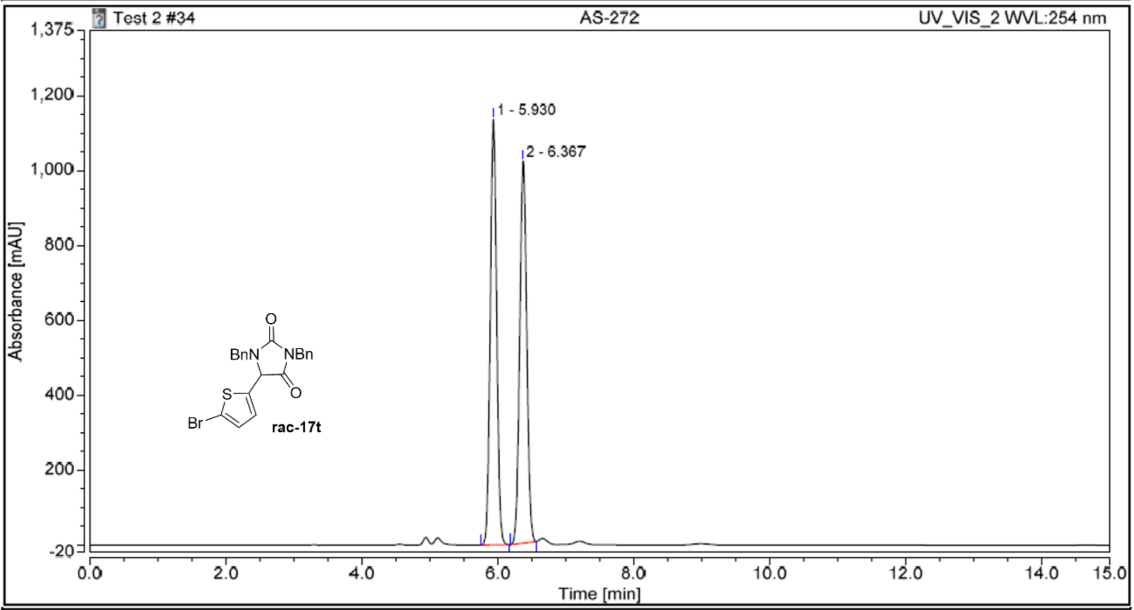

Integration Results

No.	Peak Name	Retention Time min	Area mAU*min	Height mAU	Relative Area %	Relative Height %	Amount n.a.
1		5.930	124.859	1137.405	50.50	52.69	n.a.
2		6.367	122.405	1021.389	49.50	47.31	n.a.
**Total:**			**247.263**	**2158.794**	**100.00**	**100.00**	

Chromatogram

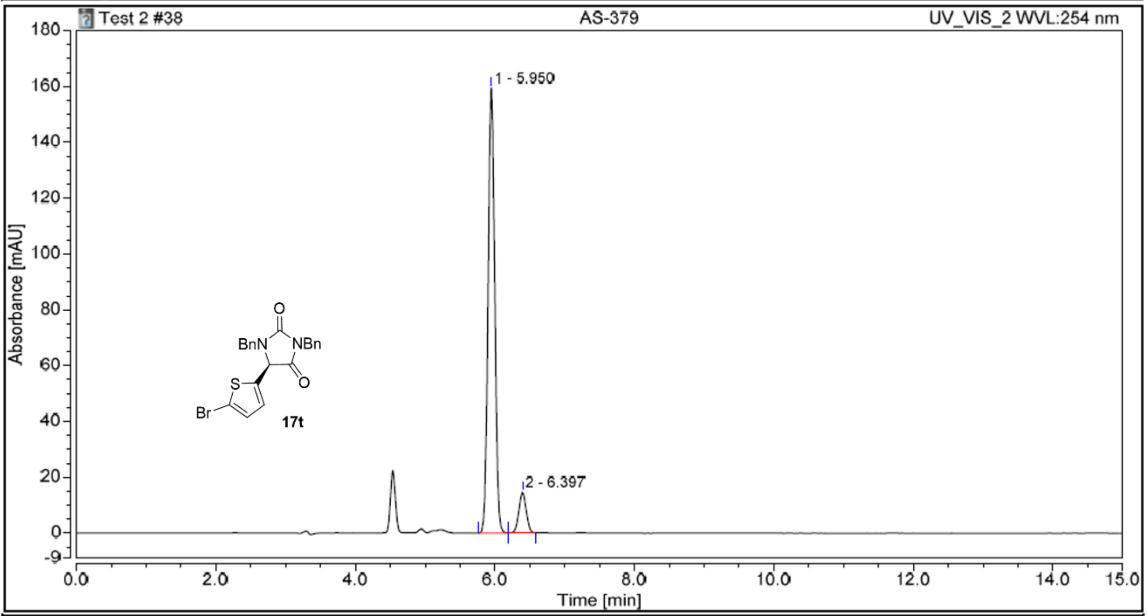

Integration Results

No.	Peak Name	Retention Time min	Area mAU*min	Height mAU	Relative Area %	Relative Height %	Amount n.a.
1		5.950	17.584	159.487	90.79	91.59	n.a.
2		6.397	1.784	14.645	9.21	8.41	n.a.
**Total:**			**19.368**	**174.132**	**100.00**	**100.00**	

1,3-Dimethyl-5-phenylimidazolidine-2,4-dione 17u

HPLC (CHIRALPAK® AS-3R, 30:70 MeCN–H_2O, 0.5 mL/min, 220 nm)

Chromatogram

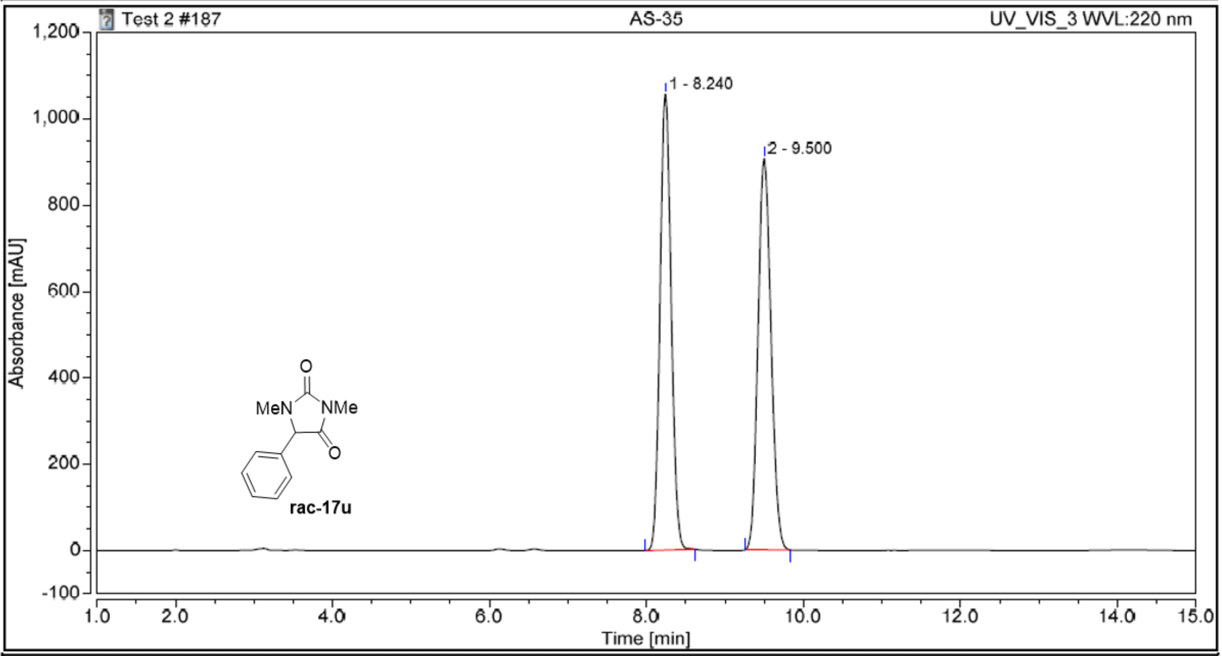

Integration Results

No.	Peak Name	Retention Time min	Area mAU*min	Height mAU	Relative Area %	Relative Height %	Amount n.a.
1		8.240	173.551	1055.402	49.96	53.81	n.a.
2		9.500	173.857	906.005	50.04	46.19	n.a.
**Total:**			**347.408**	**1961.407**	**100.00**	**100.00**	

Chromatogram

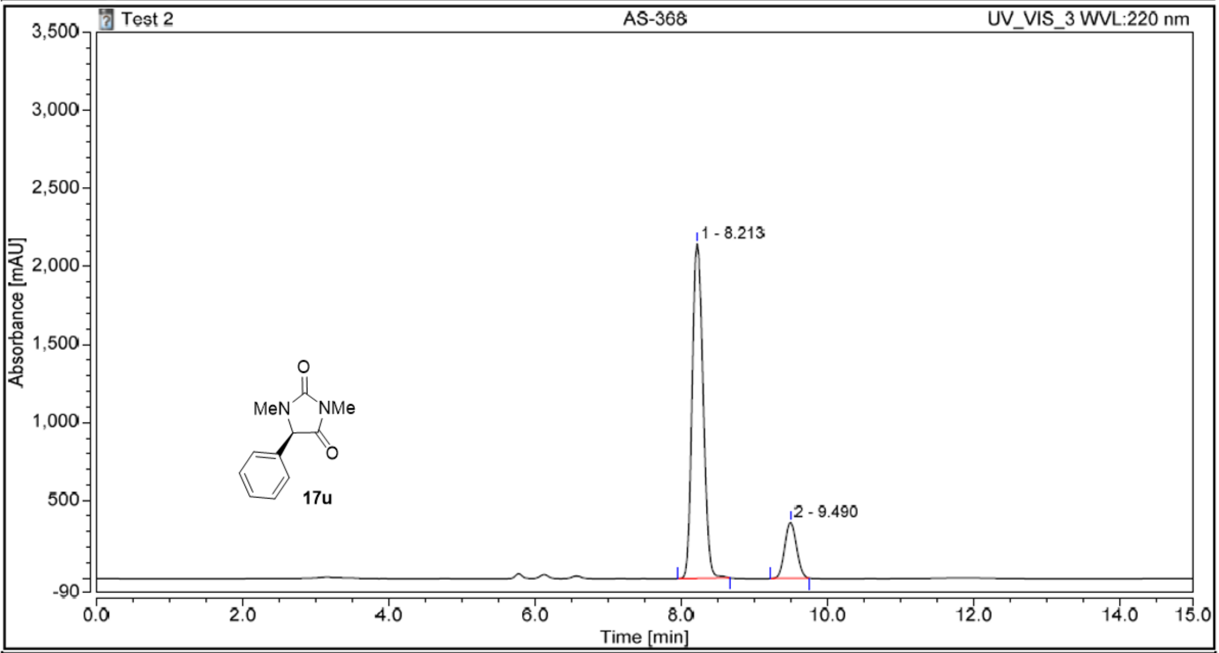

Integration Results

No.	Peak Name	Retention Time min	Area mAU*min	Height mAU	Relative Area %	Relative Height %	Amount n.a.
1		8.213	370.683	2144.838	84.65	85.67	n.a.
2		9.490	67.238	358.756	15.35	14.33	n.a.
**Total:**			**437.921**	**2503.594**	**100.00**	**100.00**	

1,3-Dimethyl-5-phenylimidazolidine-2,4-dione 17u (at 60 °C for 2 h)

HPLC (CHIRALPAK® AS-3R, 30:70 MeCN–H_2O, 0.5 mL/min, 220 nm)

Chromatogram

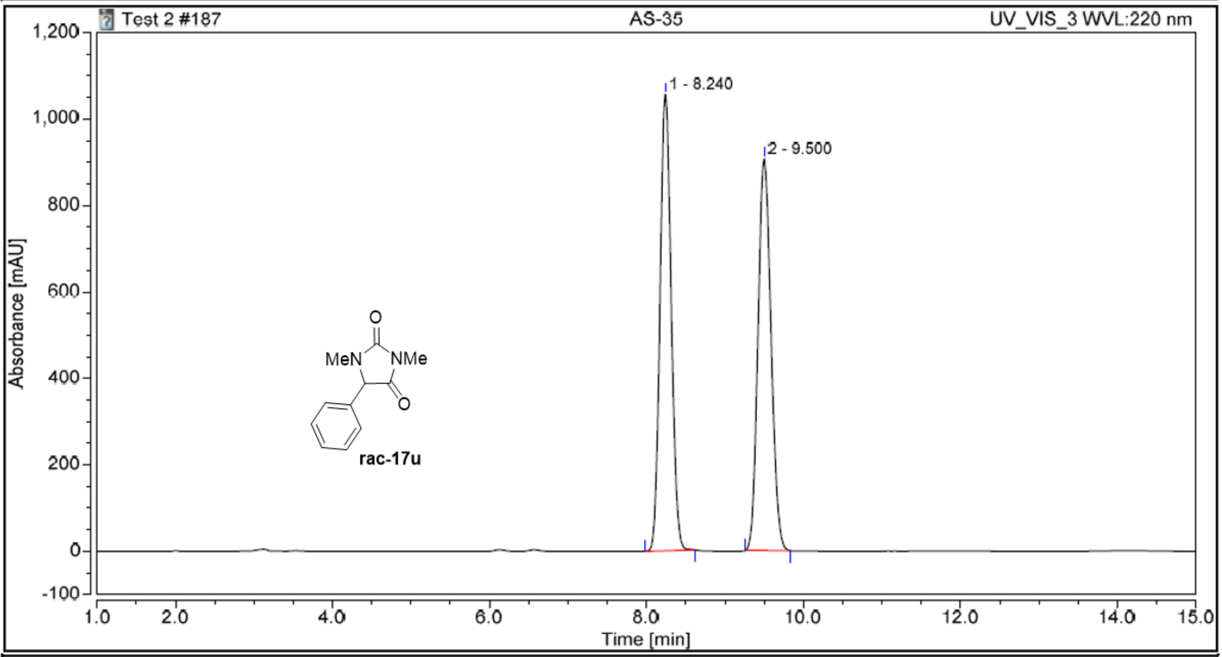

Integration Results

No.	Peak Name	Retention Time min	Area mAU*min	Height mAU	Relative Area %	Relative Height %	Amount n.a.
1		8.240	173.551	1055.402	49.96	53.81	n.a.
2		9.500	173.857	906.005	50.04	46.19	n.a.
**Total:**			**347.408**	**1961.407**	**100.00**	**100.00**	

Chromatogram

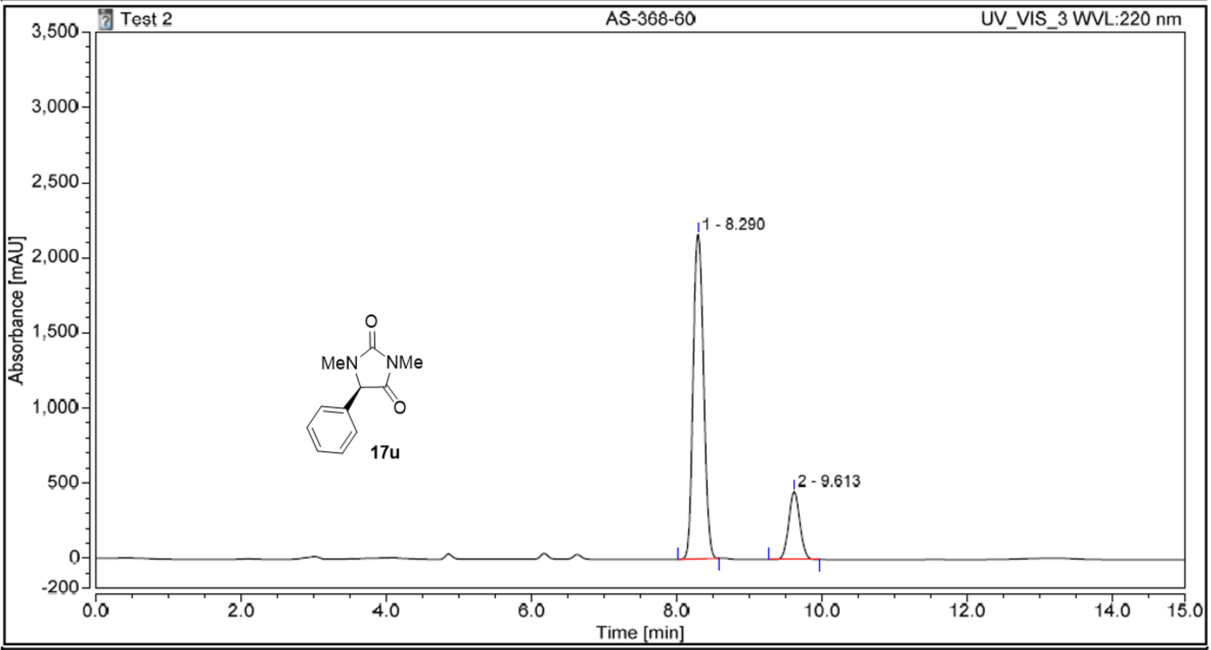

Integration Results

No.	Peak Name	Retention Time min	Area mAU*min	Height mAU	Relative Area %	Relative Height %	Amount n.a.
1		8.290	358.383	2159.582	81.59	82.65	n.a.
2		9.613	80.849	453.347	18.41	17.35	n.a.
**Total:**			**439.232**	**2612.929**	**100.00**	**100.00**	

1,3-Bis[(2,4-dimethoxyphenyl)methyl]-5-phenylimidazolidine-2,4-dione, 17v-a

HPLC (Astec® Cellulose DMP, 70:30 hexane–EtOH, 1 mL/min, 254 nm)

Chromatogram

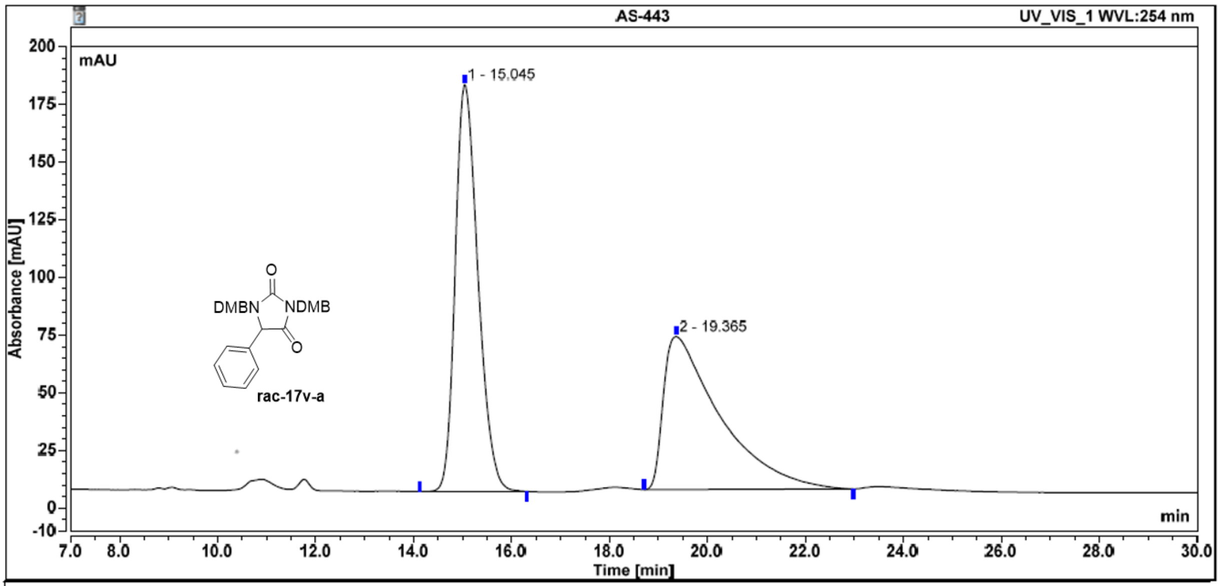

Integration Results

No.	Retention Time min	Area mAU*min	Height mAU	Relative Area %	Relative Height %
1	15.045	94.770	176.296	51.66	72.65
2	19.365	88.665	66.357	48.34	27.35
**Total:**		**183.435**	**242.653**	**100.00**	**100.00**

Chromatogram

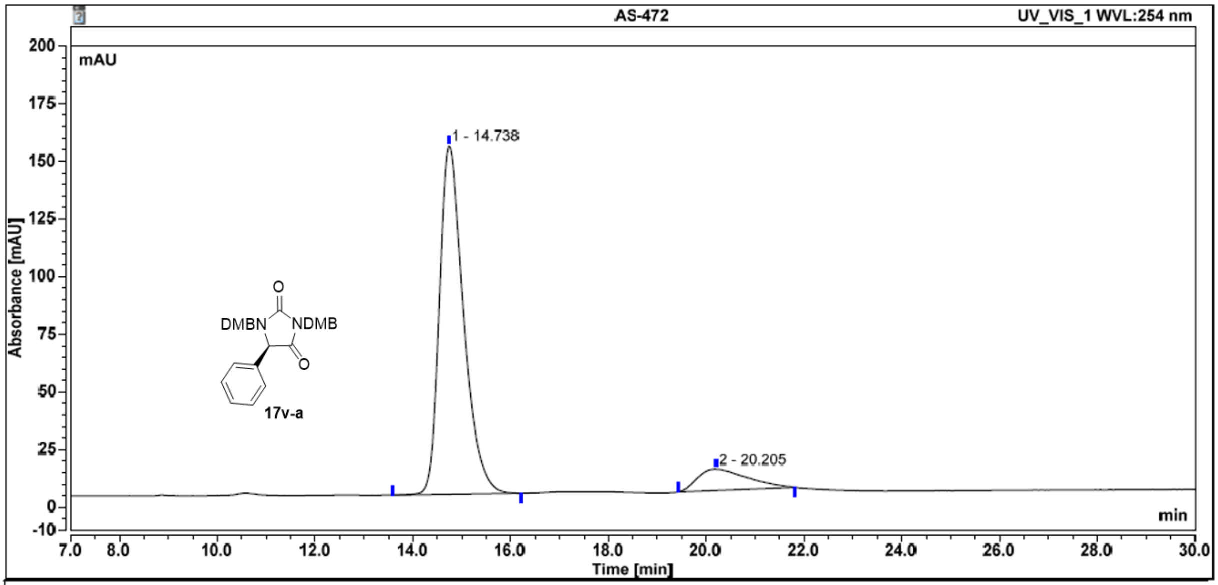

Integration Results

No.	Retention Time min	Area mAU*min	Height mAU	Relative Area %	Relative Height %
1	14.738	86.357	151.184	88.96	94.29
2	20.205	10.720	9.152	11.04	5.71
**Total:**		**97.077**	**160.335**	**100.00**	**100.00**

1,3-Bis[(2,4-dimethoxyphenyl)methyl]-5-phenylimidazolidine-2,4-dione, 17v-a (*scale-up*)

HPLC (Astec® Cellulose DMP, 70:30 hexane–EtOH, 1 mL/min, 254 nm)

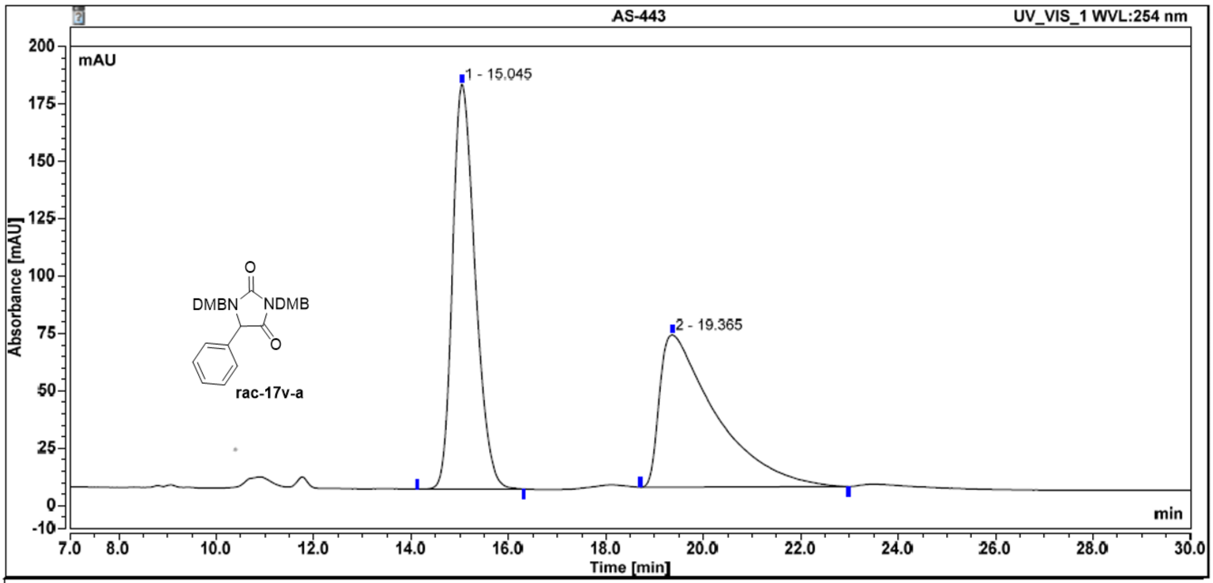

Integration Results

No.	Retention Time min	Area mAU*min	Height mAU	Relative Area %	Relative Height %
1	15.045	94.770	176.296	51.66	72.65
2	19.365	88.665	66.357	48.34	27.35
**Total:**		**183.435**	**242.653**	**100.00**	**100.00**

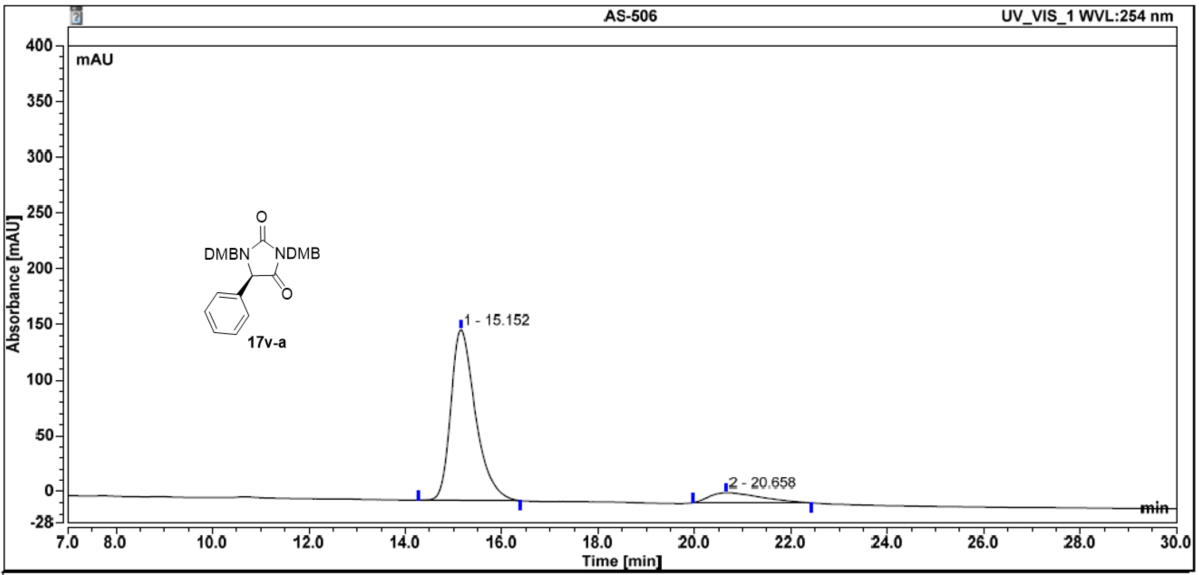

Integration Results

No.	Retention Time min	Area mAU*min	Height mAU	Relative Area %	Relative Height %
1	15.152	90.882	153.305	89.07	94.43
2	20.658	11.151	9.039	10.93	5.57
**Total:**		**102.034**	**162.344**	**100.00**	**100.00**

1,3-Bis[(2,4-dimethoxyphenyl)methyl]-5-(4-methylphenyl)imidazolidine-2,4-dione 17v-b

HPLC (Astec® Cellulose DMP, 70:30 hexane–EtOH, 1 mL/min, 254 nm)

Chromatogram

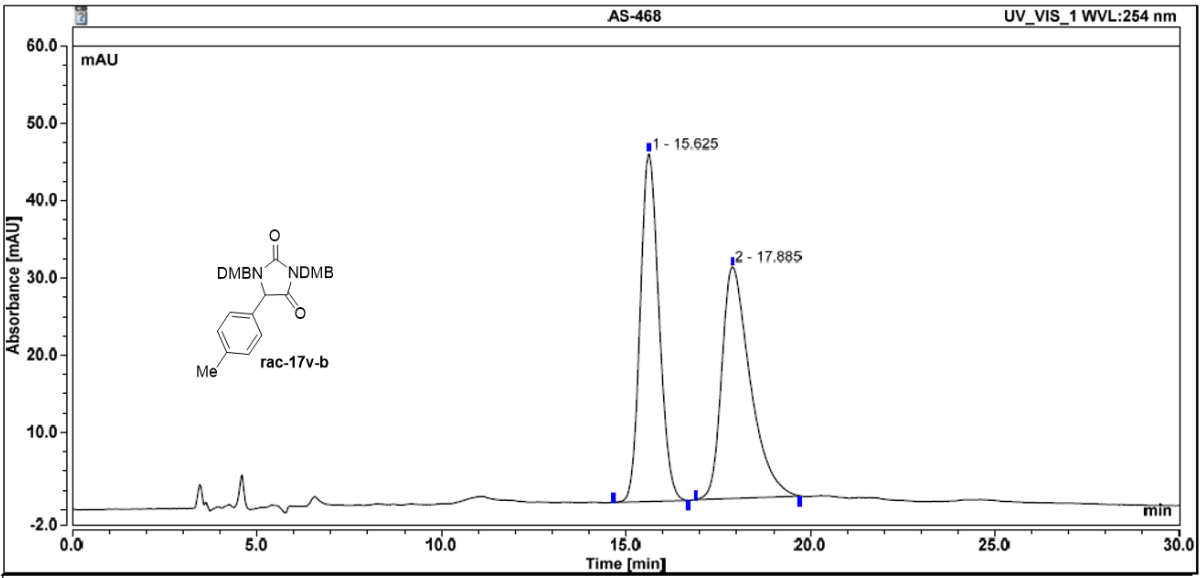

Integration Results

No.	Retention Time min	Area mAU*min	Height mAU	Relative Area %	Relative Height %
1	15.625	26.505	45.017	49.76	60.01
2	17.885	26.757	30.000	50.24	39.99
**Total:**		**53.262**	**75.017**	**100.00**	**100.00**

Chromatogram

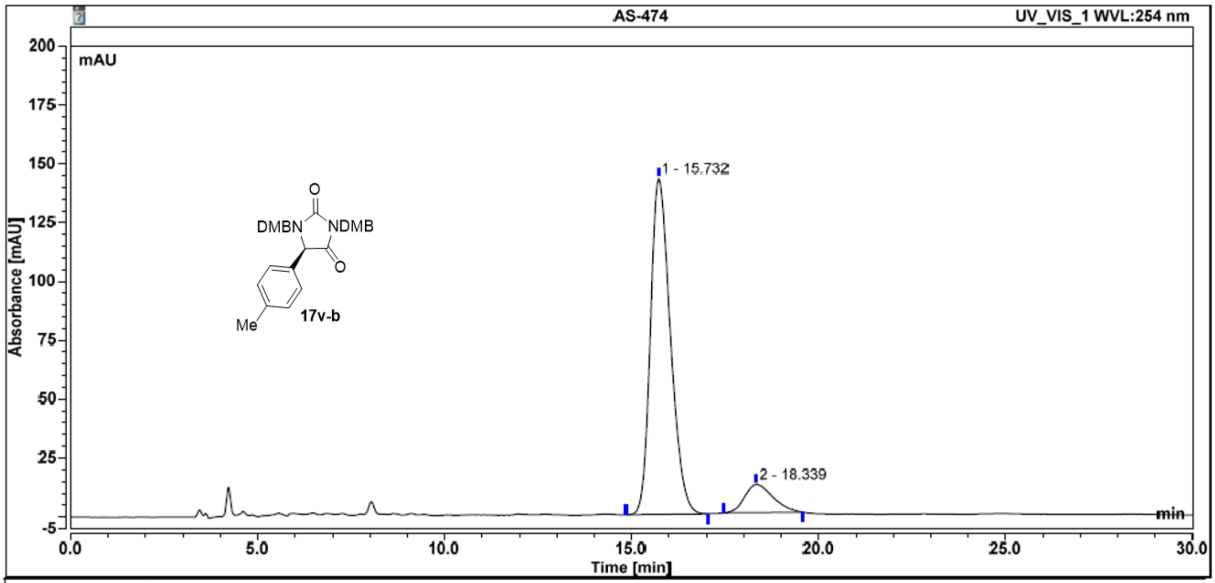

Integration Results

No.	Retention Time min	Area mAU*min	Height mAU	Relative Area %	Relative Height %
1	15.732	88.145	142.787	89.27	92.21
2	18.339	10.596	12.068	10.73	7.79
**Total:**		**98.741**	**154.855**	**100.00**	**100.00**

1,3-Bis[(2,4-dimethoxyphenyl)methyl]-5-(4-methoxyphenyl)imidazolidine-2,4-dione 17v-c

HPLC (Astec® Cellulose DMP, 70:30 hexane–EtOH, 1 mL/min, 254 nm)

Chromatogram

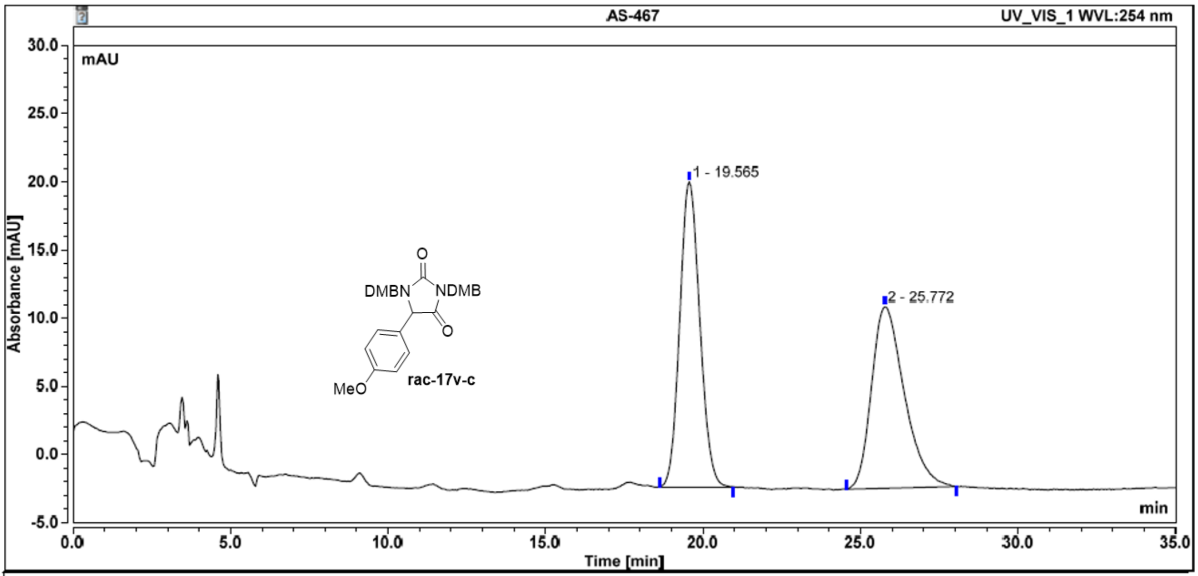

Integration Results

No.	Retention Time min	Area mAU*min	Height mAU	Relative Area %	Relative Height %
1	19.565	16.506	22.423	50.71	62.71
2	25.772	16.043	13.334	49.29	37.29
**Total:**		**32.550**	**35.757**	**100.00**	**100.00**

Chromatogram

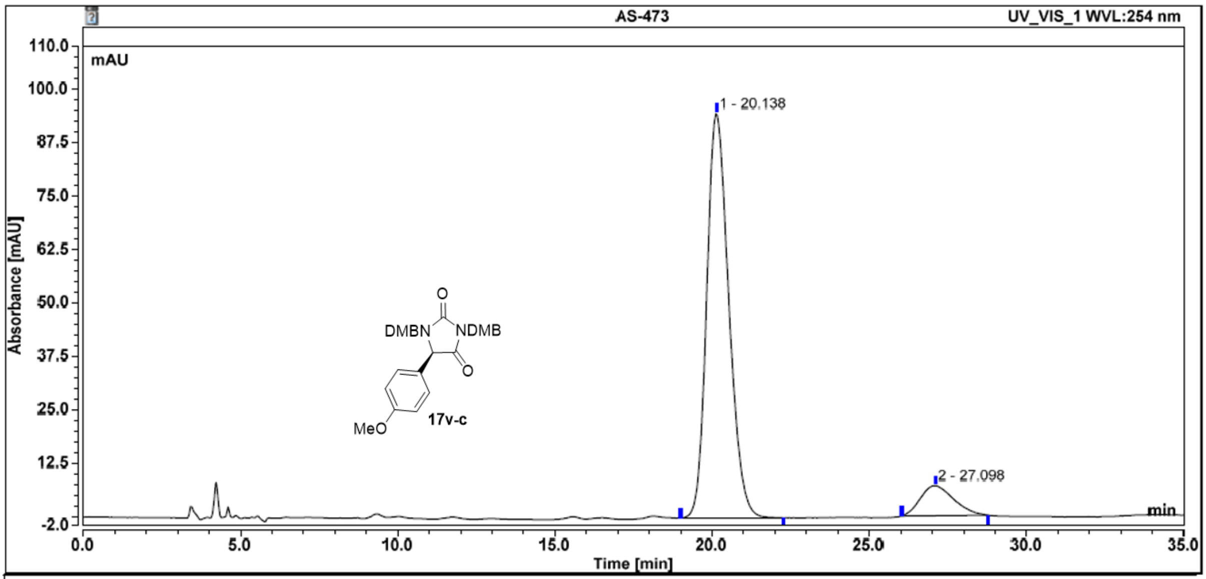

Integration Results

No.	Retention Time min	Area mAU*min	Height mAU	Relative Area %	Relative Height %
1	20.138	76.842	94.624	89.95	93.09
2	27.098	8.586	7.026	10.05	6.91
**Total:**		**85.428**	**101.650**	**100.00**	**100.00**

1,3-Bis[(2,4-dimethoxyphenyl)methyl]-5-(4-fluorophenyl)imidazolidine-2,4-dione 4v-d

HPLC (Astec® Cellulose DMP, 70:30 hexane–EtOH, 1 mL/min, 254 nm)

Chromatogram

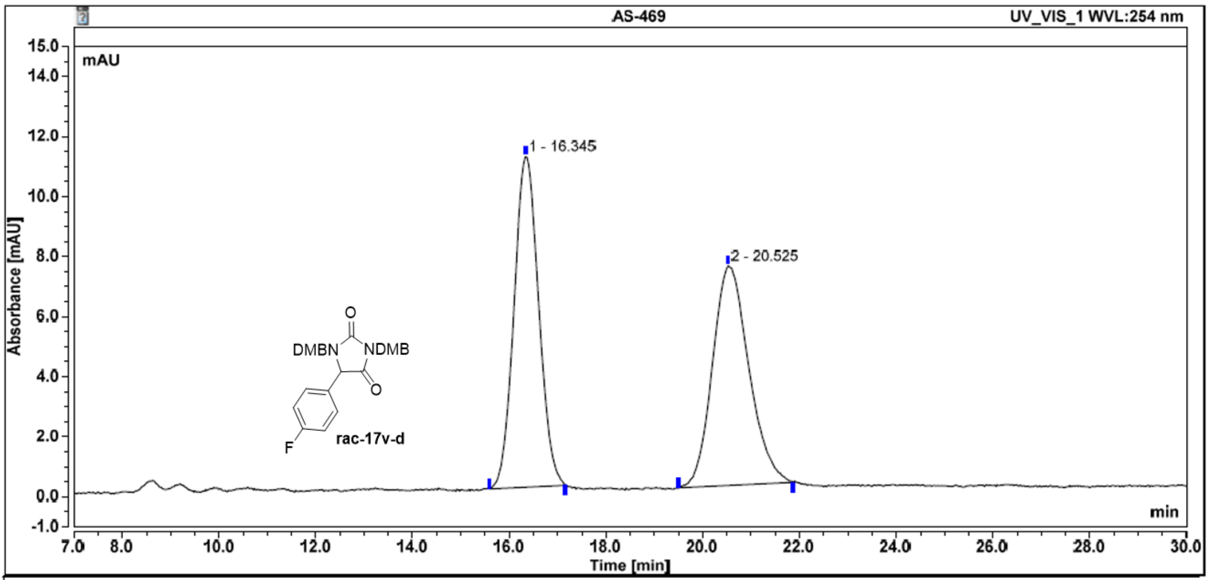

Integration Results

No.	Retention Time<br>min	Area<br>mAU*min	Height<br>mAU	Relative Area<br>%	Relative Height<br>%
1	16.345	6.506	11.041	50.57	60.15
2	20.525	6.360	7.315	49.43	39.85
**Total:**		**12.867**	**18.356**	**100.00**	**100.00**

Chromatogram

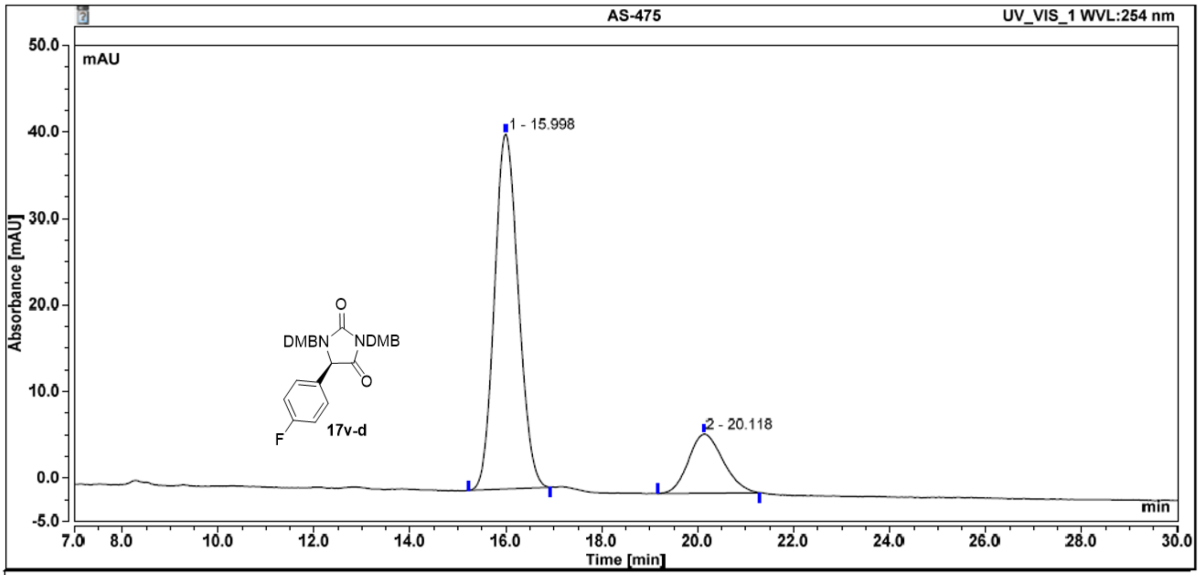

Integration Results

No.	Retention Time<br>min	Area<br>mAU*min	Height<br>mAU	Relative Area<br>%	Relative Height<br>%
1	15.998	23.678	41.049	80.54	85.68
2	20.118	5.722	6.860	19.46	14.32
**Total:**		**29.400**	**47.909**	**100.00**	**100.00**

1,3-Bis[(2,4-dimethoxyphenyl)methyl]-5-(4-iodophenyl)imidazolidine-2,4-dione 4v-e

HPLC (Astec® Cellulose DMP, 70:30 hexane–EtOH, 1 mL/min, 254 nm)

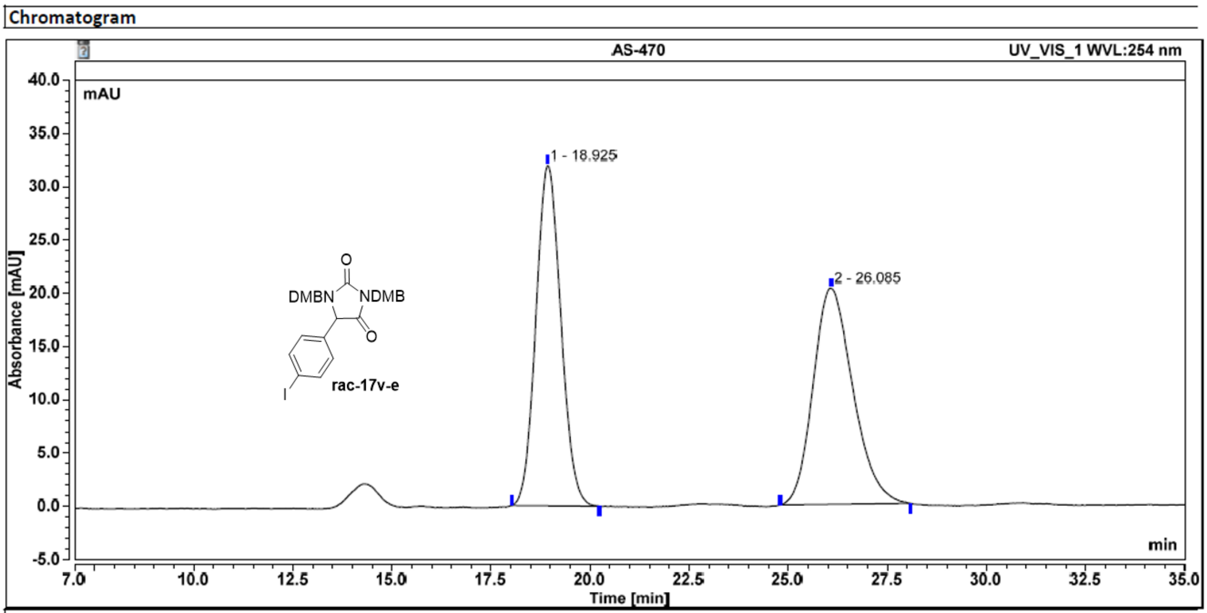

Integration Results

No.	Retention Time min	Area mAU*min	Height mAU	Relative Area %	Relative Height %
1	18.925	23.307	31.958	49.72	61.14
2	26.085	23.574	20.313	50.28	38.86
Total:		46.881	52.271	100.00	100.00

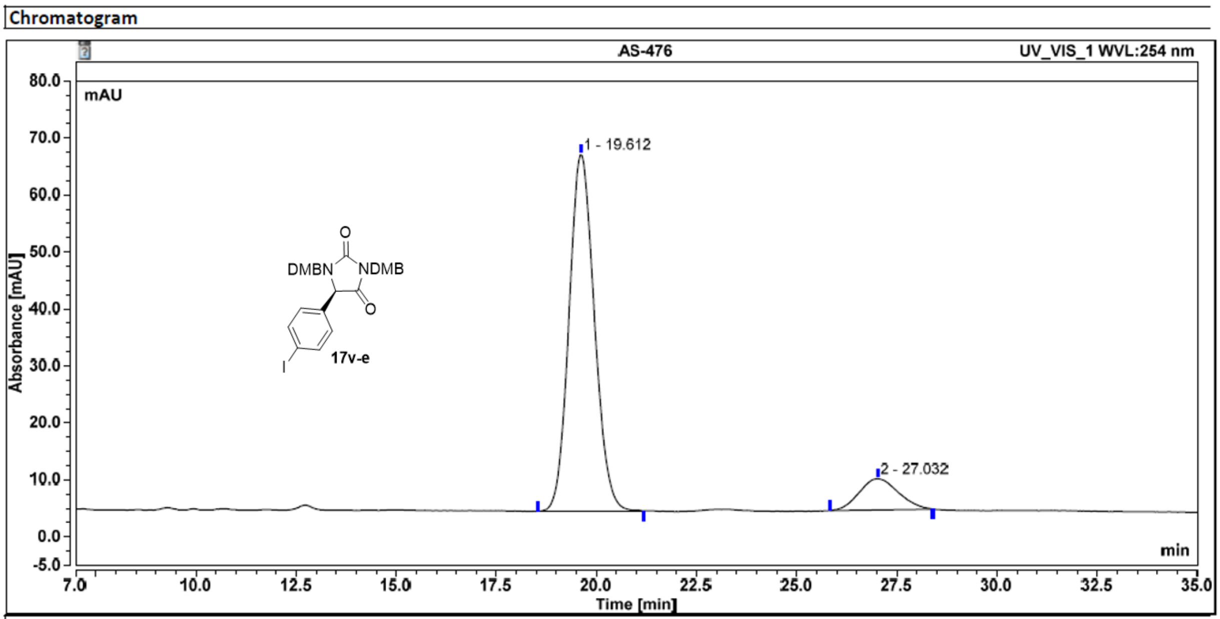

Integration Results

No.	Retention Time min	Area mAU*min	Height mAU	Relative Area %	Relative Height %
1	19.612	45.967	62.702	88.12	91.95
2	27.032	6.197	5.492	11.88	8.05
Total:		52.164	68.194	100.00	100.00

1,3-[(2,4-Dimethoxyphenyl)methyl]-5-phenylimidazolidine-2,4-dione 17w.

HPLC (CHIRALPAK® AS-3R, 50:50 MeCN–H_2O, 0.5 mL/min, 280 nm)

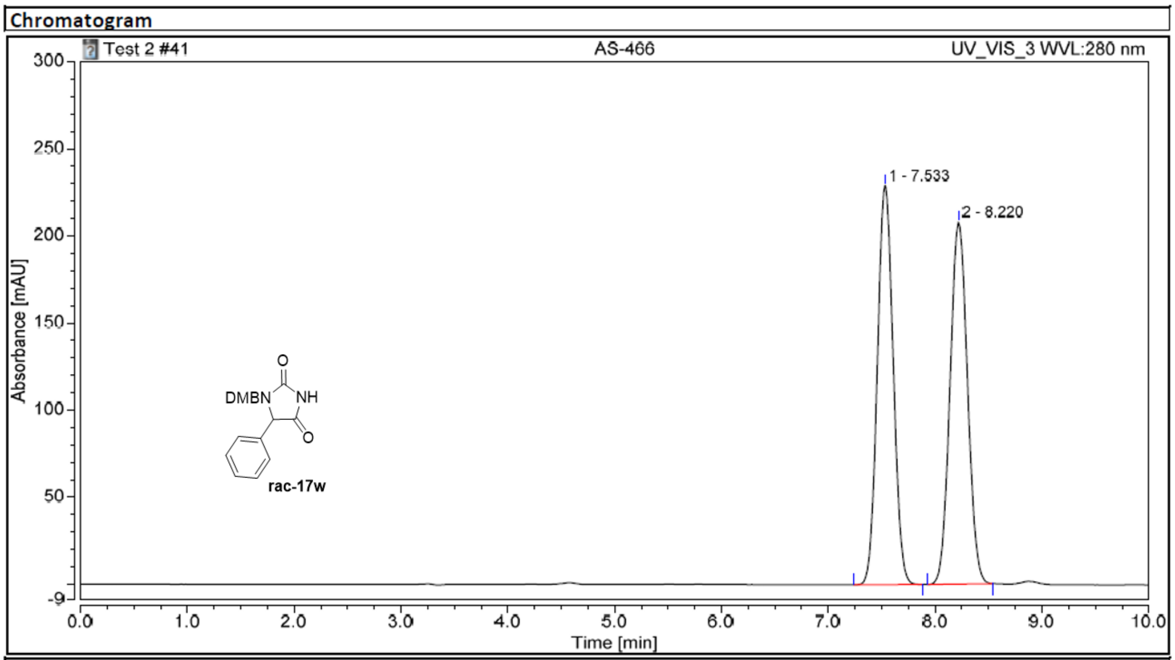

Integration Results

No.	Peak Name	Retention Time min	Area mAU*min	Height mAU	Relative Area %	Relative Height %	Amount n.a.
1		7.533	39.343	229.203	50.02	52.41	n.a.
2		8.220	39.312	208.152	49.98	47.59	n.a.
**Total:**			**78.655**	**437.355**	**100.00**	**100.00**	

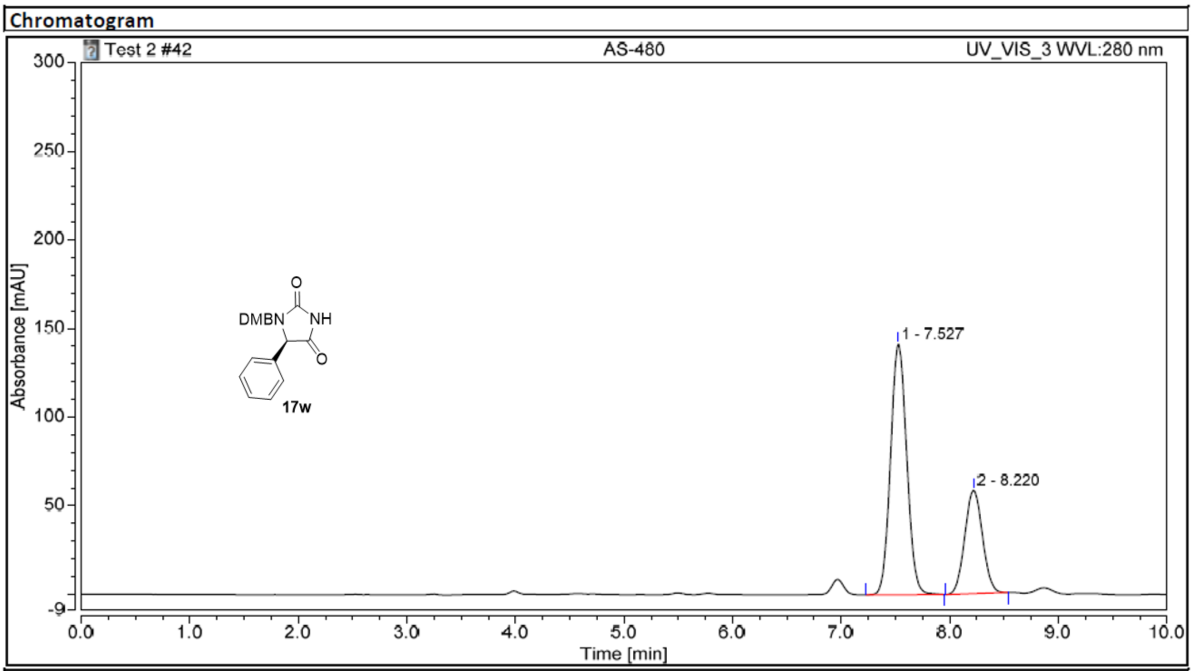

Integration Results

No.	Peak Name	Retention Time min	Area mAU*min	Height mAU	Relative Area %	Relative Height %	Amount n.a.
1		7.527	24.450	141.484	69.00	70.78	n.a.
2		8.220	10.984	58.414	31.00	29.22	n.a.
**Total:**			**35.434**	**199.899**	**100.00**	**100.00**	

1,3-Dimethyl-5-phenyl-2-sulfanylideneimidazolidin-4-one 17x (at rt for 20 h).

HPLC (CHIRALPAK® AS-3R, 50:50 MeCN–H_2O, 0.5 mL/min, 254 nm)

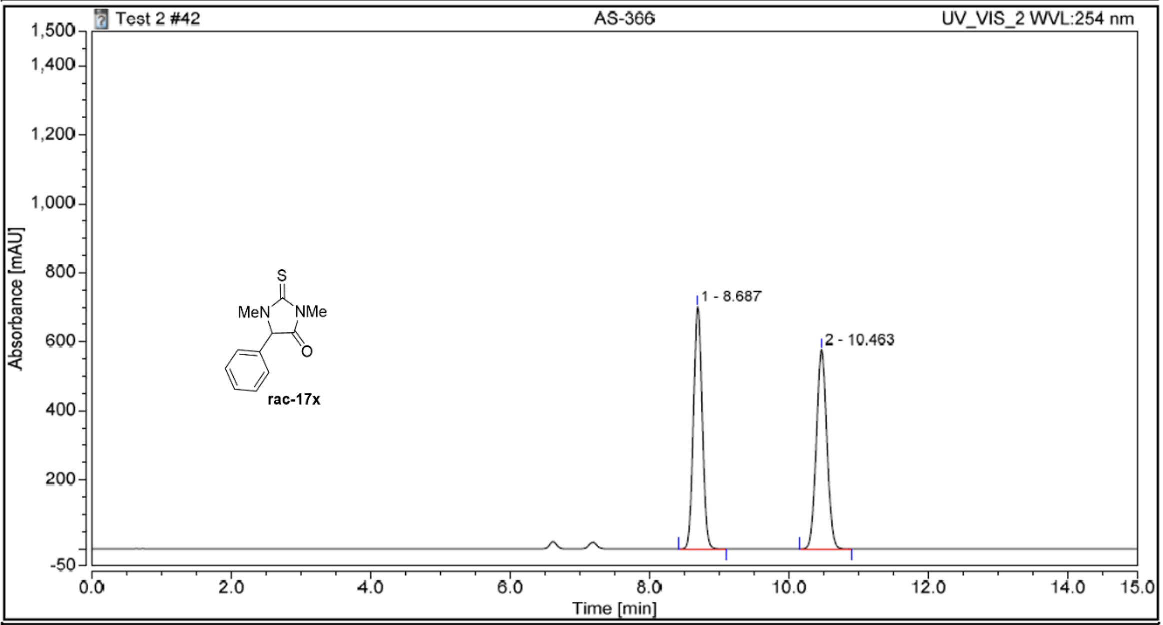

Integration Results

No.	Peak Name	Retention Time min	Area mAU*min	Height mAU	Relative Area %	Relative Height %	Amount n.a.
1		8.687	101.108	703.216	49.96	54.90	n.a.
2		10.463	101.257	577.630	50.04	45.10	n.a.
**Total:**			**202.365**	**1280.846**	**100.00**	**100.00**	

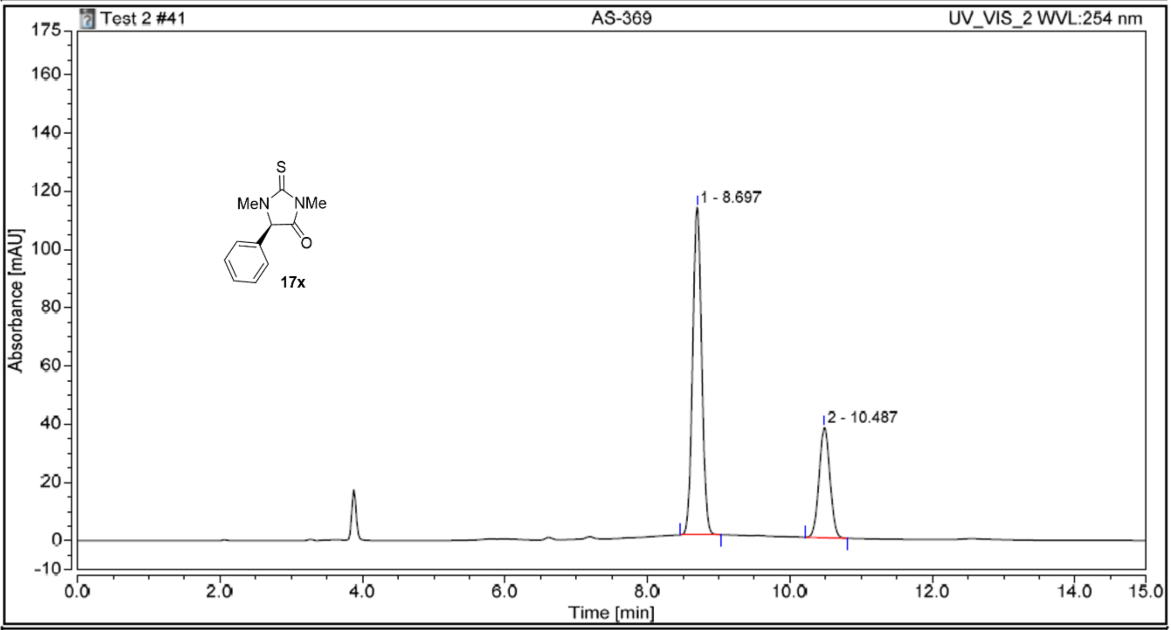

Integration Results

No.	Peak Name	Retention Time min	Area mAU*min	Height mAU	Relative Area %	Relative Height %	Amount n.a.
1		8.697	16.459	112.523	71.09	74.81	n.a.
2		10.487	6.692	37.879	28.91	25.19	n.a.
**Total:**			**23.151**	**150.402**	**100.00**	**100.00**	

1,3-Dimethyl-5-phenyl-2-sulfanylideneimidazolidin-4-one 17x (at 60 ºC for 0.5 h)

HPLC (CHIRALPAK® AS-3R, 50:50 MeCN–H_2O, 0.5 mL/min, 254 nm)

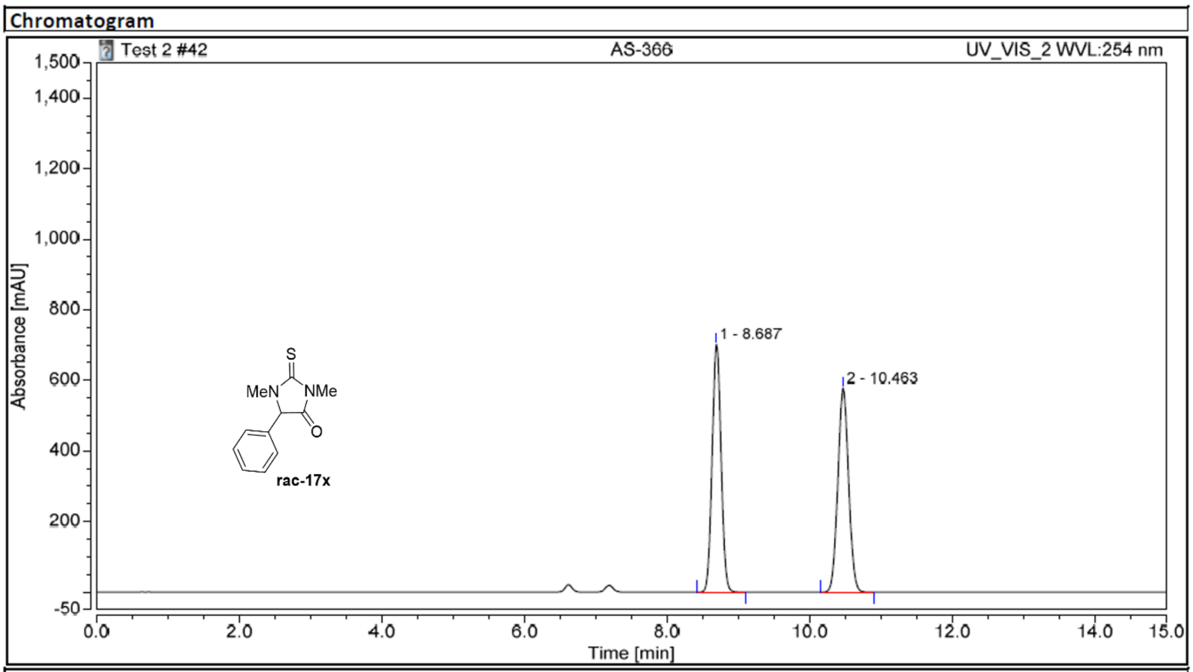

Integration Results

No.	Peak Name	Retention Time min	Area mAU*min	Height mAU	Relative Area %	Relative Height %	Amount n.a.
1		8.687	101.108	703.216	49.96	54.90	n.a.
2		10.463	101.257	577.630	50.04	45.10	n.a.
**Total:**			**202.365**	**1280.846**	**100.00**	**100.00**	

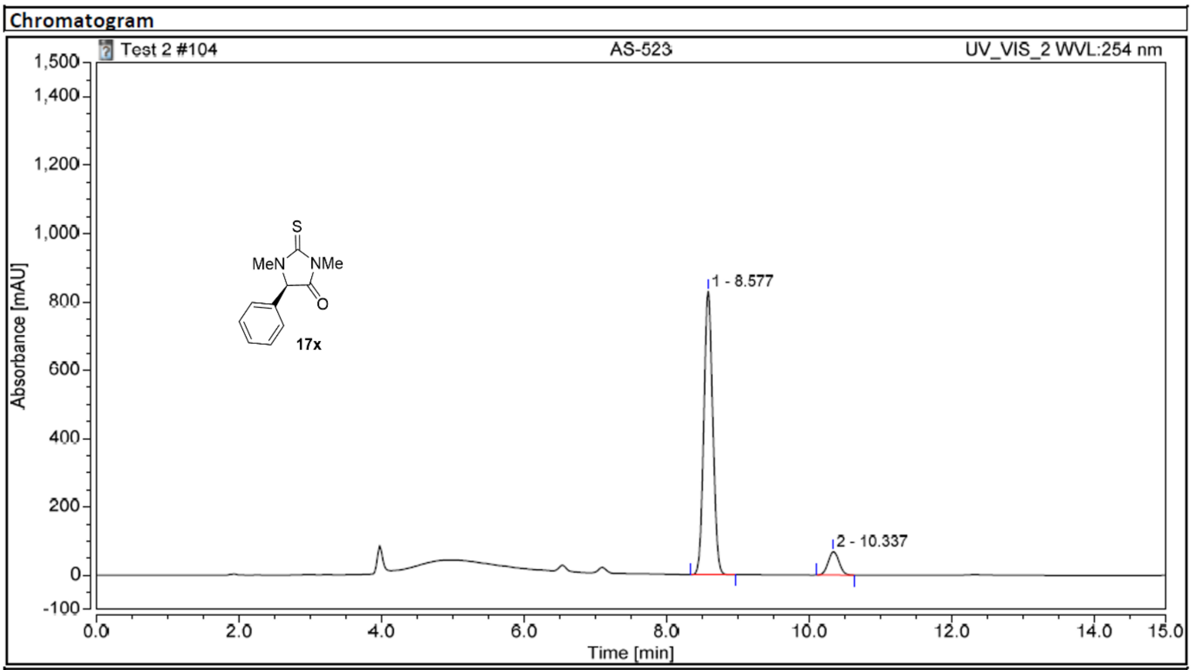

Integration Results

No.	Peak Name	Retention Time min	Area mAU*min	Height mAU	Relative Area %	Relative Height %	Amount n.a.
1		8.577	120.059	829.696	90.90	92.34	n.a.
2		10.337	12.019	68.829	9.10	7.66	n.a.
**Total:**			**132.078**	**898.526**	**100.00**	**100.00**	

1,3-Dibenzyl-5-tert-butylimidazolidine-2,4-dione S17y

HPLC (Astec® Cellulose DMP, 94:6 hexane–EtOH, 1 mL/min, 220 nm)

Chromatogram

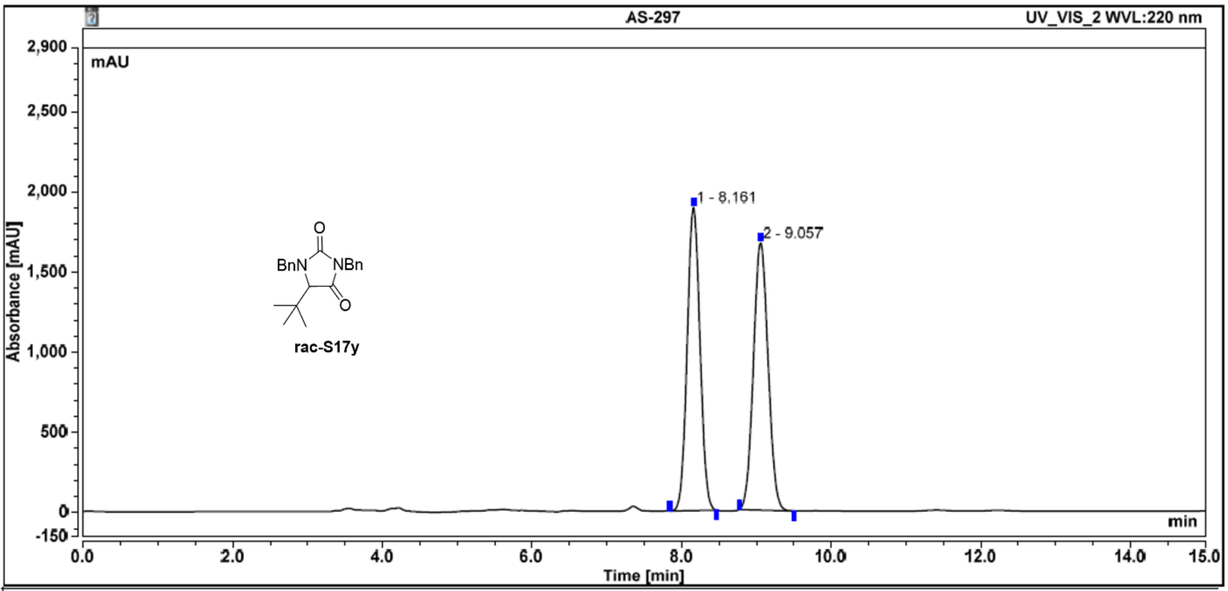

Integration Results

No.	Retention Time min	Area mAU*min	Height mAU	Relative Area %	Relative Height %
1	8.161	374.940	1893.065	49.69	53.15
2	9.057	379.640	1668.943	50.31	46.85
**Total:**		**754.580**	**3562.008**	**100.00**	**100.00**

Chromatogram

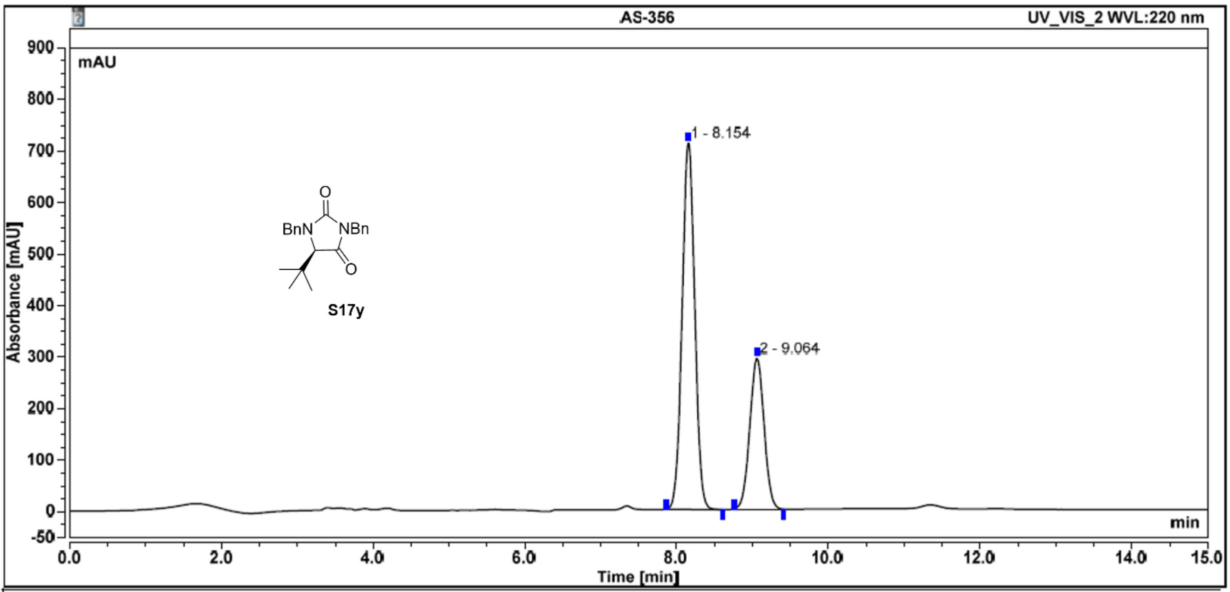

Integration Results

No.	Retention Time min	Area mAU*min	Height mAU	Relative Area %	Relative Height %
1	8.154	136.698	710.945	68.23	70.83
2	9.064	63.651	292.792	31.77	29.17
**Total:**		**200.349**	**1003.737**	**100.00**	**100.00**

5-(Adamantan-1-yl)-1,3-dibenzylimidazolidine-2,4-dione S17z

HPLC (Astec® Cellulose DMP, 80:20 hexane–EtOH, 1 mL/min, 254 nm)

Chromatogram

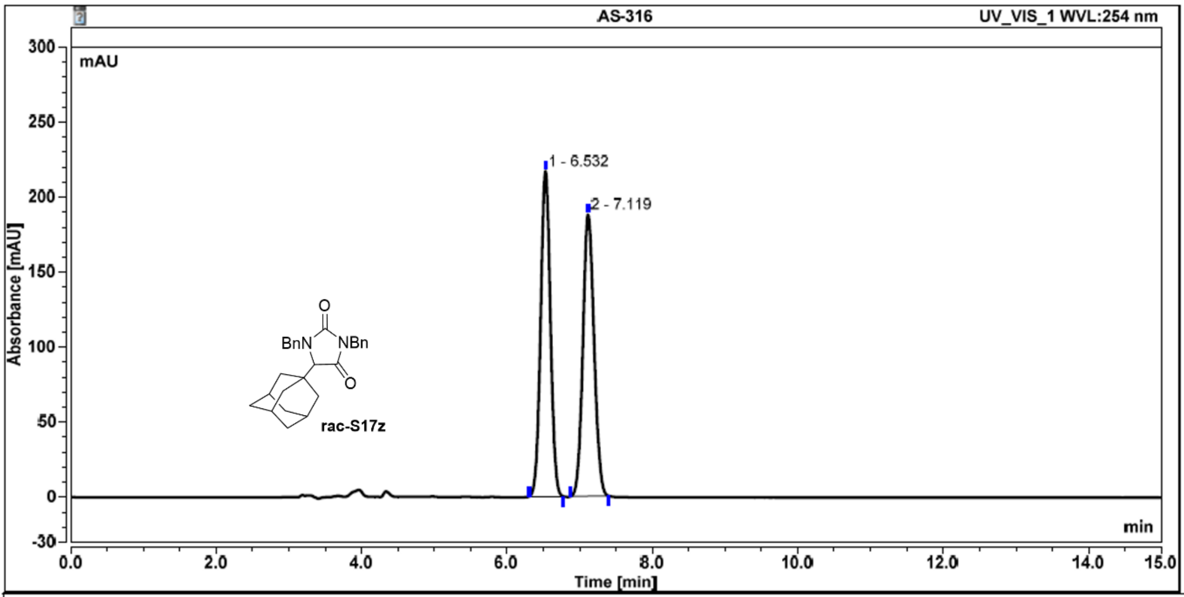

Integration Results

No.	Retention Time<br>min	Area<br>mAU*min	Height<br>mAU	Relative Area<br>%	Relative Height<br>%
1	6.532	33.554	216.924	50.12	53.57
2	7.119	33.391	188.000	49.88	46.43
**Total:**		**66.944**	**404.925**	**100.00**	**100.00**

Chromatogram

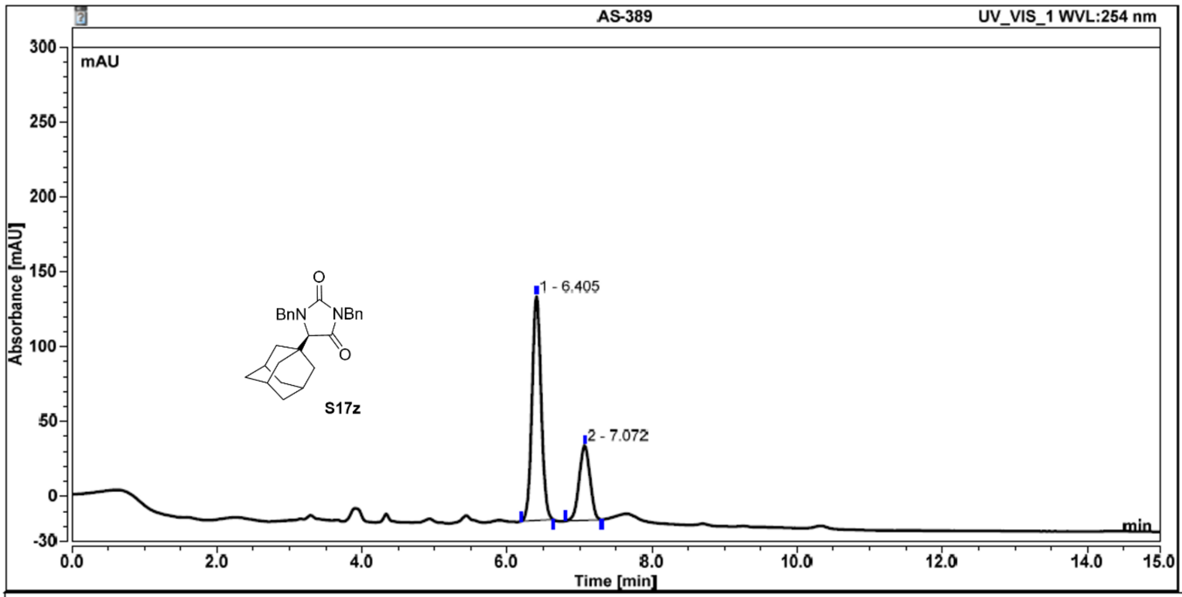

Integration Results

No.	Retention Time<br>min	Area<br>mAU*min	Height<br>mAU	Relative Area<br>%	Relative Height<br>%
1	6.405	21.793	149.695	71.58	74.94
2	7.072	8.653	50.049	28.42	25.06
**Total:**		**30.447**	**199.744**	**100.00**	**100.00**

1,3-Dibenzyl-5-cyclohexylimidazolidine-2,4-dione, S17aa

HPLC (Astec® Cellulose DMP, 80:20 hexane–EtOH, 1 mL/min, 254 nm)

Chromatogram

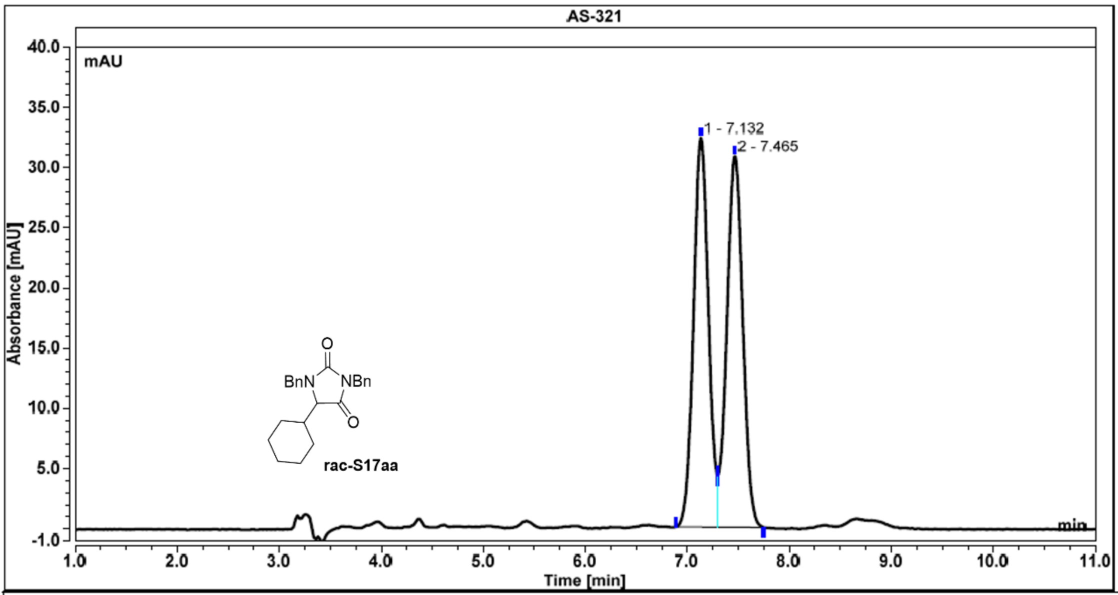

Integration Results

No.	Retention Time min	Area mAU*min	Height mAU	Relative Area %	Relative Height %
1	7.132	5.490	32.316	49.52	51.19
2	7.465	5.596	30.811	50.48	48.81
Total:		11.086	63.128	100.00	100.00

Chromatogram

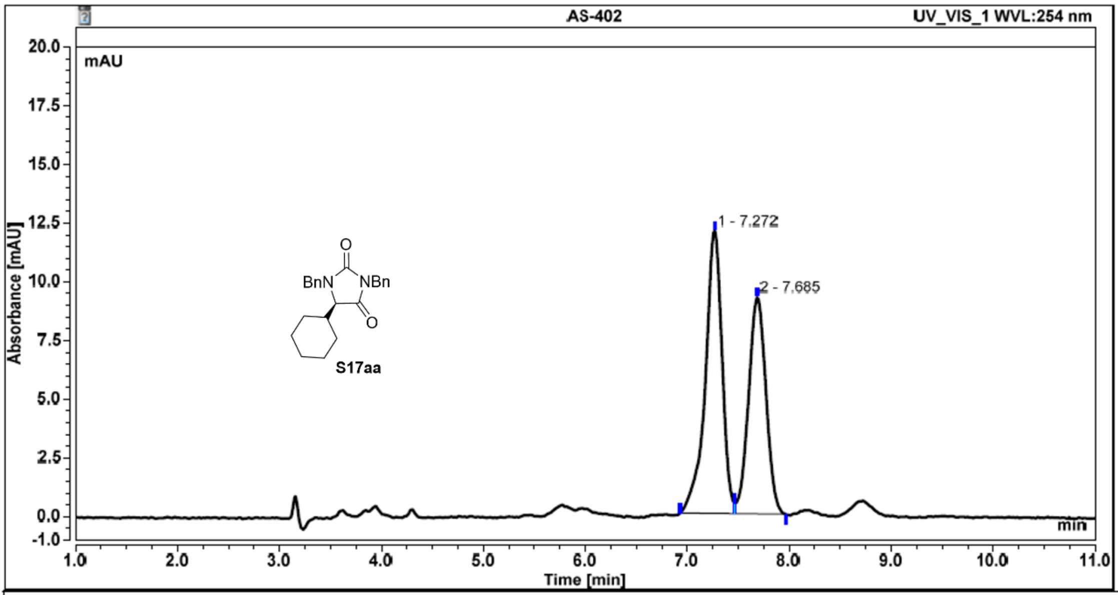

Integration Results

No.	Retention Time min	Area mAU*min	Height mAU	Relative Area %	Relative Height %
1	7.272	2.285	12.011	55.58	56.58
2	7.685	1.826	9.217	44.42	43.42
Total:		4.111	21.227	100.00	100.00

1,3-Dibenzyl-5-cyclopropylimidazolidine-2,4-dione S17ab

HPLC (CHIRALPAK® AS-3R, 60:40 MeCN–H_2O, 0.5 mL/min, 220 nm)

Chromatogram

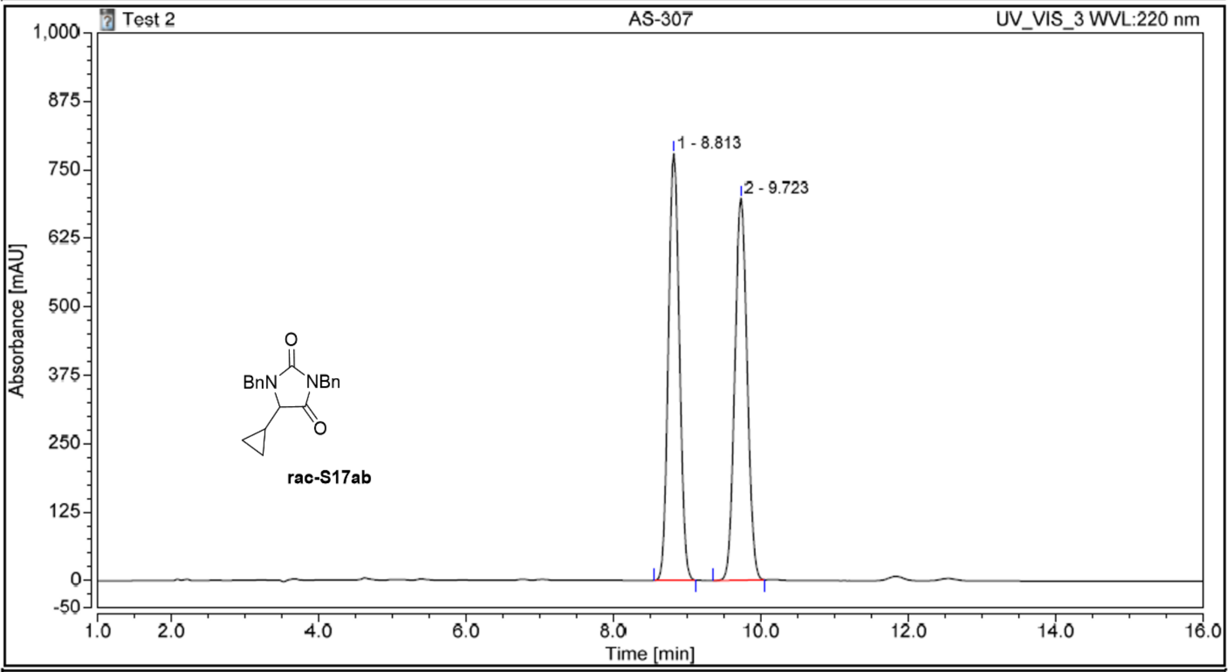

Integration Results

No.	Peak Name	Retention Time<br>min	Area<br>mAU*min	Height<br>mAU	Relative Area<br>%	Relative Height<br>%	Amount<br>n.a.
1		8.813	134.476	779.579	49.80	52.78	n.a.
2		9.723	135.558	697.344	50.20	47.22	n.a.
**Total:**			**270.034**	**1476.922**	**100.00**	**100.00**	

Chromatogram

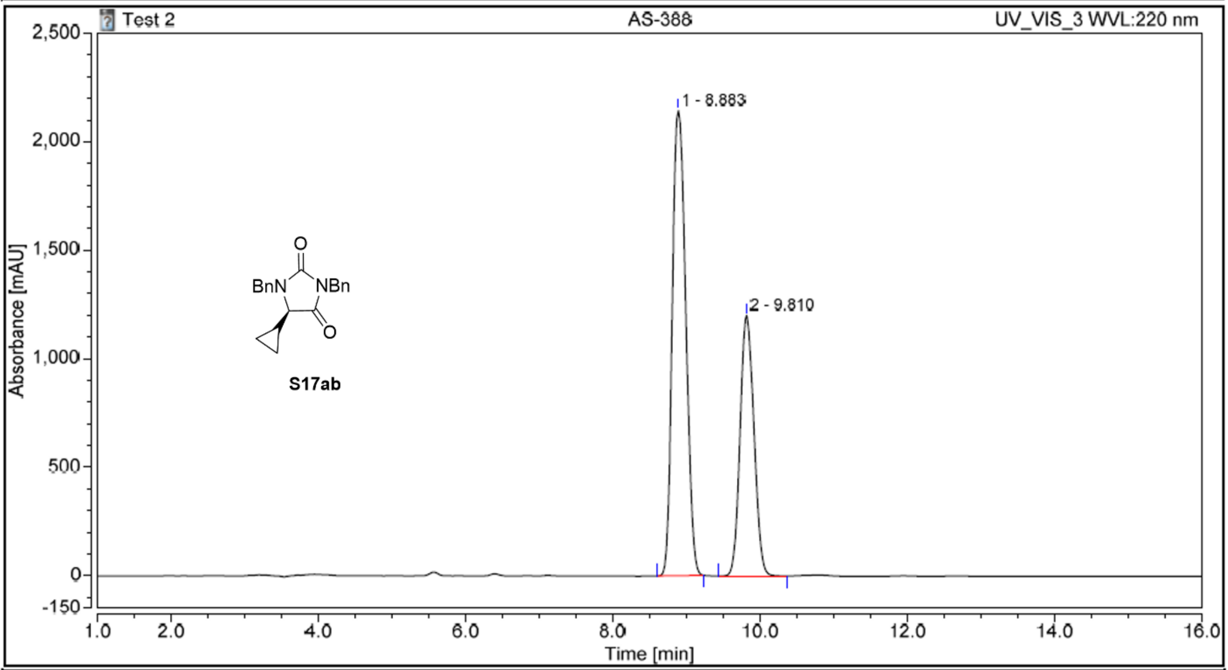

Integration Results

No.	Peak Name	Retention Time<br>min	Area<br>mAU*min	Height<br>mAU	Relative Area<br>%	Relative Height<br>%	Amount<br>n.a.
1		8.883	454.165	2145.498	63.44	64.13	n.a.
2		9.810	261.762	1199.968	36.56	35.87	n.a.
**Total:**			**715.928**	**3345.466**	**100.00**	**100.00**	

1,3-Dibenzyl-5-cyclopropylimidazolidine-2,4-dione S17ab (at 60 °C for 2 h)

HPLC (CHIRALPAK® AS-3R, 60:40 MeCN–H_2O, 0.5 mL/min, 220 nm)

Chromatogram

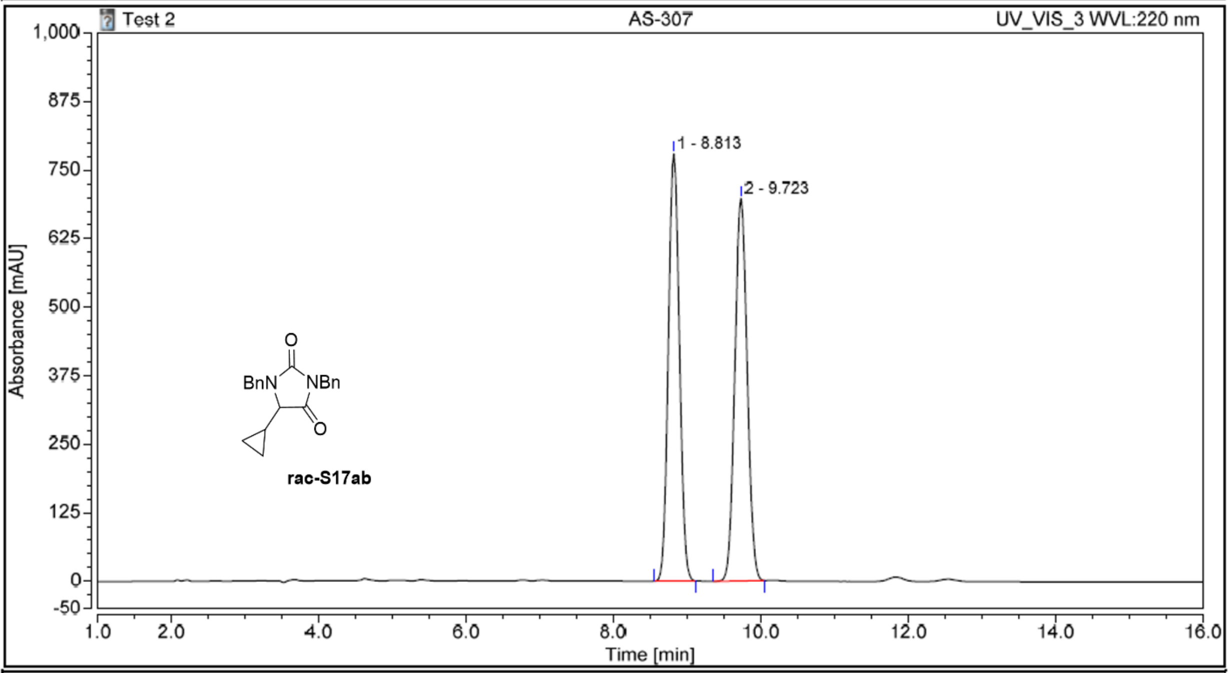

Integration Results

No.	Peak Name	Retention Time min	Area mAU*min	Height mAU	Relative Area %	Relative Height %	Amount n.a.
1		8.813	134.476	779.579	49.80	52.78	n.a.
2		9.723	135.558	697.344	50.20	47.22	n.a.
**Total:**			**270.034**	**1476.922**	**100.00**	**100.00**	

Chromatogram

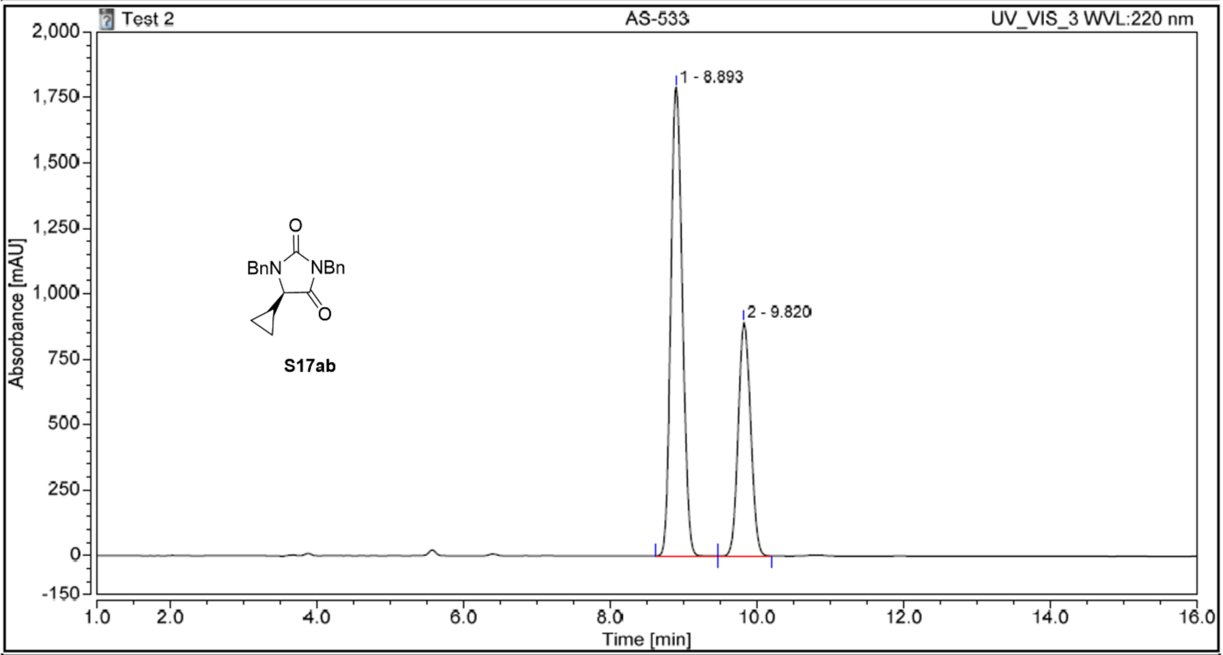

Integration Results

No.	Peak Name	Retention Time min	Area mAU*min	Height mAU	Relative Area %	Relative Height %	Amount n.a.
1		8.893	327.117	1792.733	65.12	66.73	n.a.
2		9.820	175.223	893.687	34.88	33.27	n.a.
**Total:**			**502.340**	**2686.419**	**100.00**	**100.00**	

1,3,5-Tribenzylimidazolidine-2,4-dione S17ac

HPLC (Astec® Cellulose DMP, 80:20 hexane–EtOH, 1 mL/min, 254 nm)

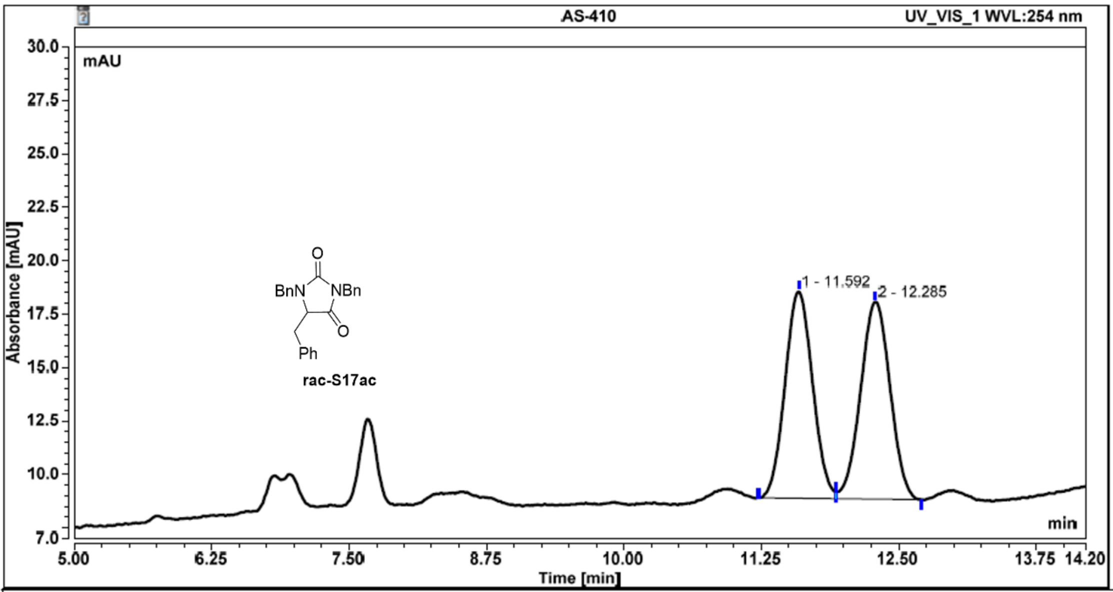

Integration Results

No.	Retention Time min	Area mAU*min	Height mAU	Relative Area %	Relative Height %
1	11.592	2.825	9.683	49.54	51.16
2	12.285	2.877	9.245	50.46	48.84
**Total:**		**5.702**	**18.928**	**100.00**	**100.00**

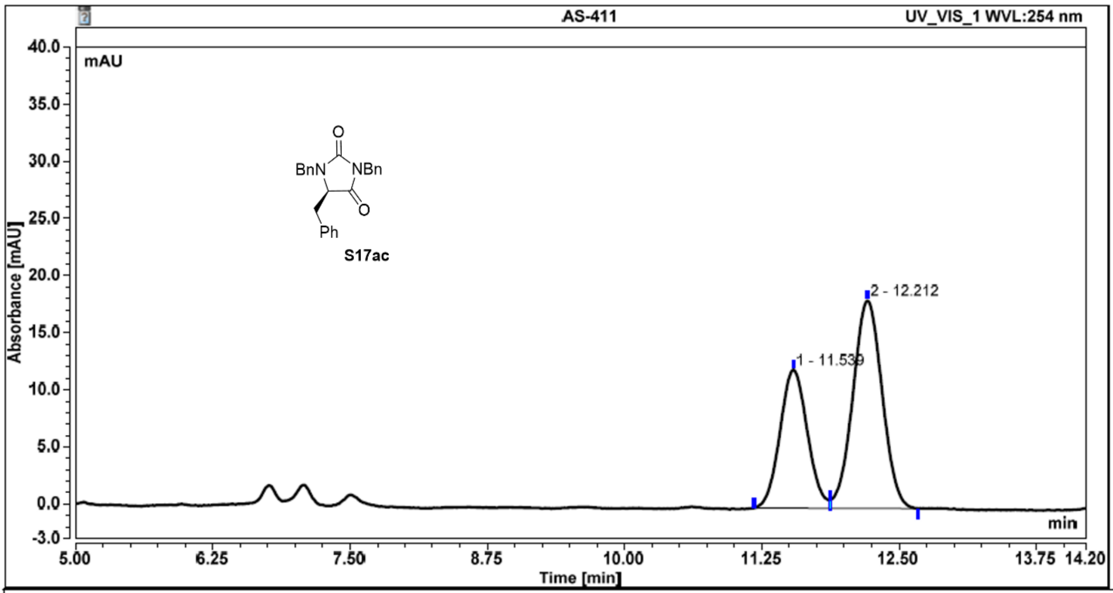

Integration Results

No.	Retention Time min	Area mAU*min	Height mAU	Relative Area %	Relative Height %
1	11.539	3.517	12.118	38.47	40.00
2	12.212	5.626	18.179	61.53	60.00
**Total:**		**9.143**	**30.297**	**100.00**	**100.00**

1,3-Dibenzyl-4,5-dihydroxy-4-phenylimidazolidin-2-one 15a

HPLC (CHIRALPAK® AS-3R, 50:50 MeCN–H_2O, 0.5 mL/min, 210 nm)

Chromatogram

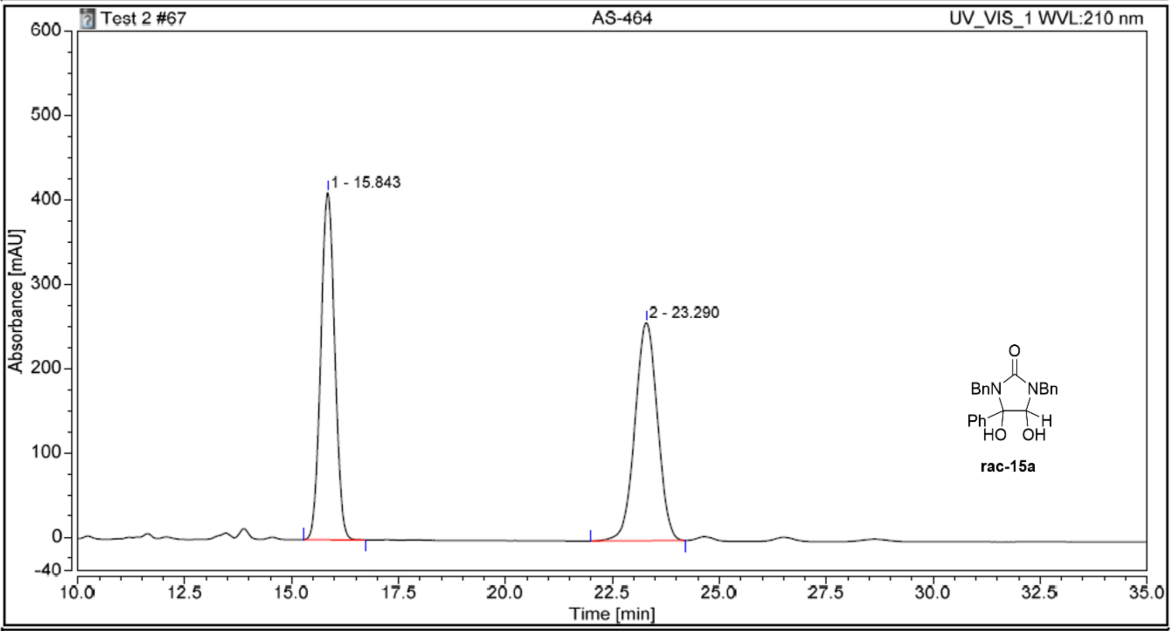

Integration Results

No.	Peak Name	Retention Time<br>min	Area<br>mAU*min	Height<br>mAU	Relative Area<br>%	Relative Height<br>%	Amount<br>n.a.
1		15.843	155.563	411.966	49.63	61.44	n.a.
2		23.290	157.861	258.585	50.37	38.56	n.a.
**Total:**			**313.423**	**670.552**	**100.00**	**100.00**	

Chromatogram

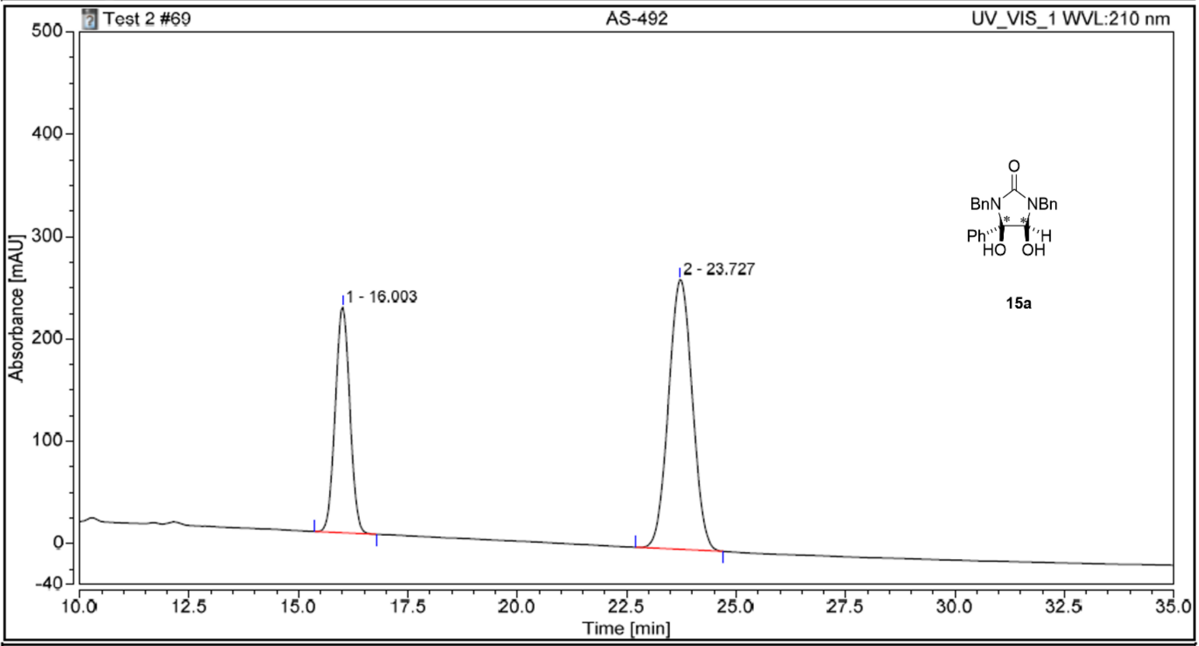

Integration Results

No.	Peak Name	Retention Time<br>min	Area<br>mAU*min	Height<br>mAU	Relative Area<br>%	Relative Height<br>%	Amount<br>n.a.
1		16.003	85.326	220.877	33.93	45.56	n.a.
2		23.727	166.117	263.912	66.07	54.44	n.a.
**Total:**			**251.443**	**484.789**	**100.00**	**100.00**	

3-[(2,4-Dimethoxyphenyl)methyl]-5-phenylimidazolidine-2,4-dione 22v-a

HPLC (CHIRALPAK® AS-3R, 50:50 MeCN–H_2O, 0.5 mL/min, 280 nm)

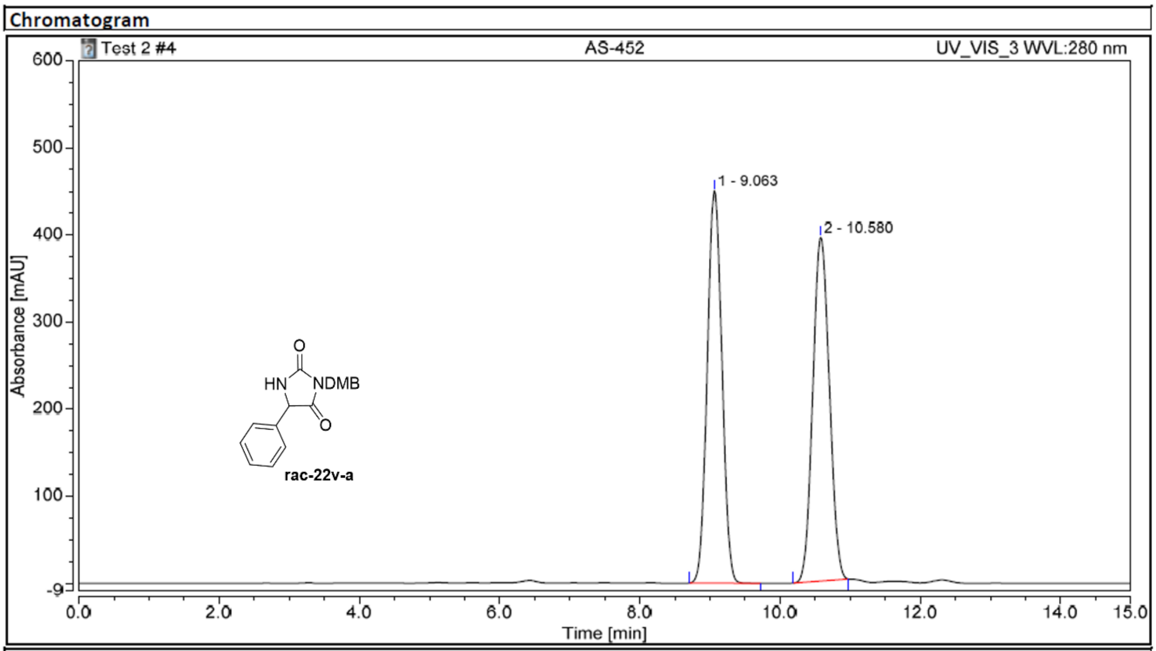

Integration Results

No.	Peak Name	Retention Time min	Area mAU*min	Height mAU	Relative Area %	Relative Height %	Amount n.a.
1		9.063	109.182	450.754	50.34	53.31	n.a.
2		10.580	107.709	394.755	49.66	46.69	n.a.
**Total:**			**216.891**	**845.509**	**100.00**	**100.00**	

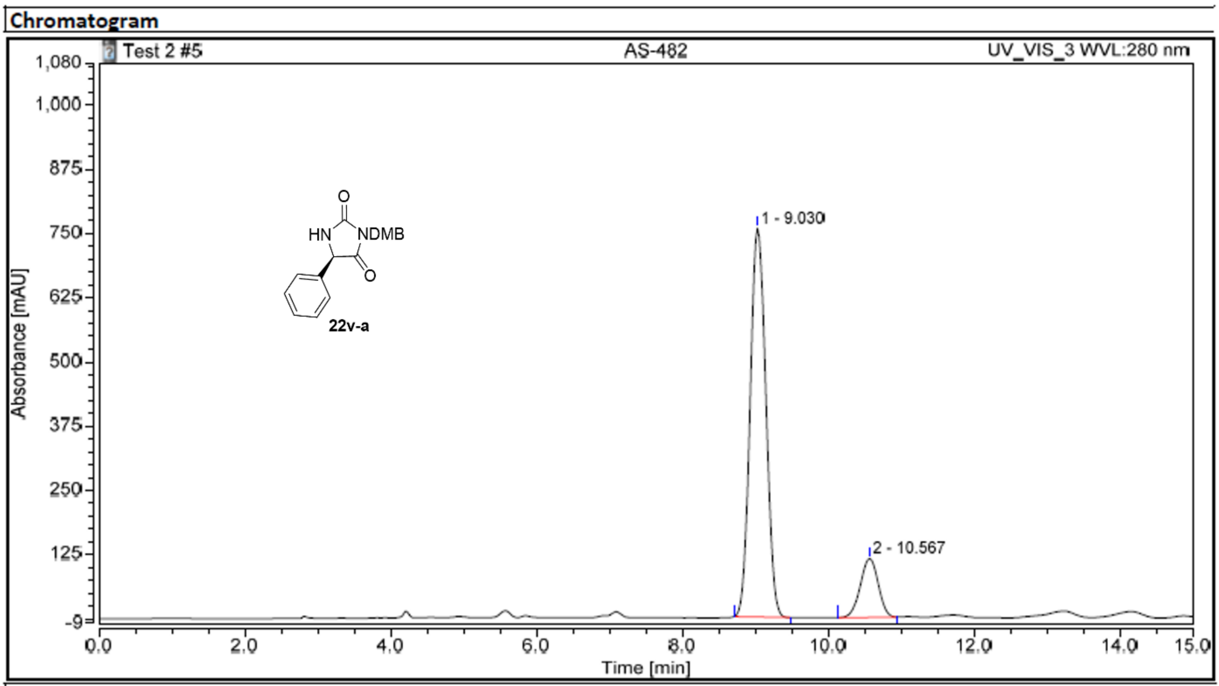

Integration Results

No.	Peak Name	Retention Time min	Area mAU*min	Height mAU	Relative Area %	Relative Height %	Amount n.a.
1		9.030	188.741	756.949	85.30	86.87	n.a.
2		10.567	32.514	114.432	14.70	13.13	n.a.
**Total:**			**221.255**	**871.381**	**100.00**	**100.00**	

1,3-Dibenzyl-4-phenylimidazolidine 23a

HPLC (Astec® Cellulose DMP, 80:20 hexane–*i*-PrOH, 0.5 mL/min, 254 nm)

Chromatogram

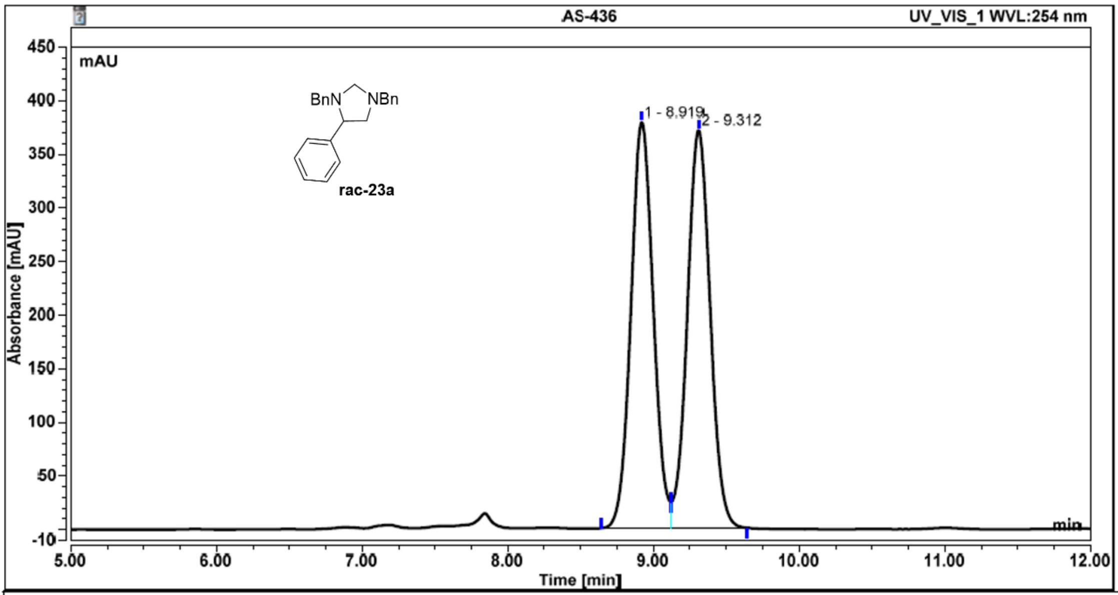

Integration Results

No.	Retention Time min	Area mAU*min	Height mAU	Relative Area %	Relative Height %
1	8.919	67.170	378.539	49.90	50.51
2	9.312	67.445	370.899	50.10	49.49
**Total:**		**134.615**	**749.438**	**100.00**	**100.00**

Chromatogram

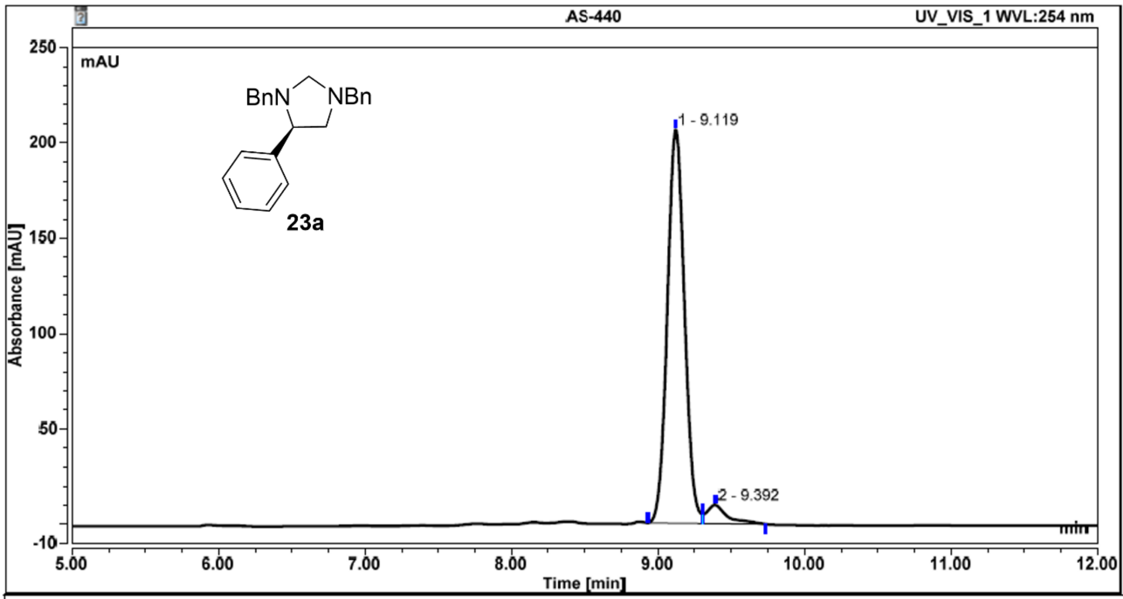

Integration Results

No.	Retention Time min	Area mAU*min	Height mAU	Relative Area %	Relative Height %
1	9.119	27.861	206.203	94.60	95.50
2	9.392	1.590	9.722	5.40	4.50
**Total:**		**29.451**	**215.925**	**100.00**	**100.00**

Benzyl[2-(benzylamino)-1-phenylethyl]amine 24a

HPLC (Astec® Cellulose DMP, 94:6 hexane–EtOH, 0.5 mL/min, 254 nm)

Chromatogram

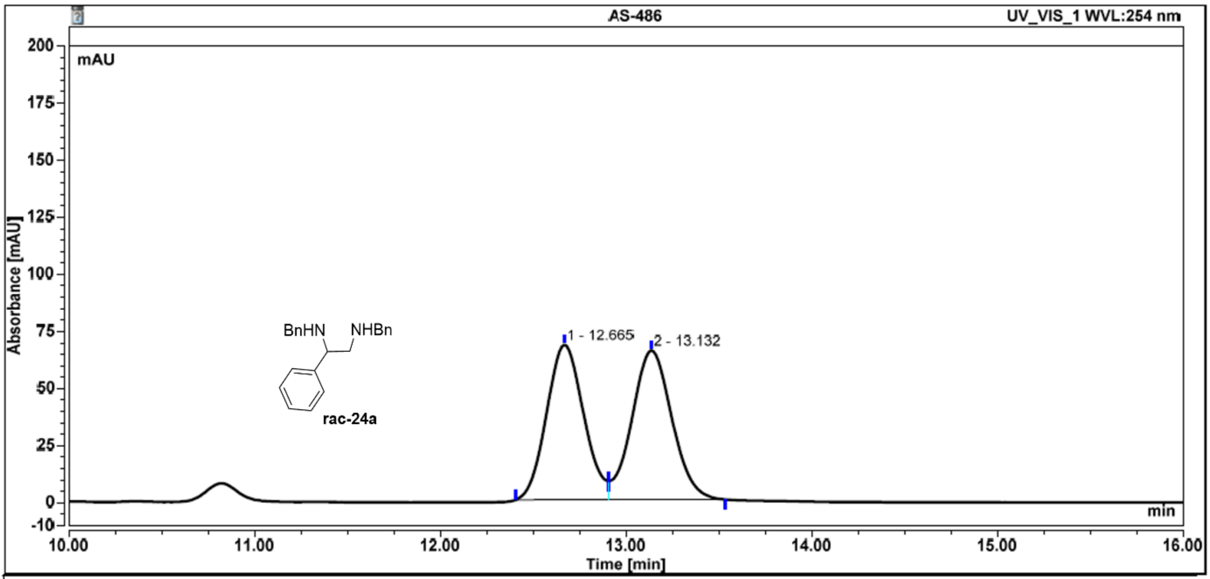

Integration Results

No.	Retention Time min	Area mAU*min	Height mAU	Relative Area %	Relative Height %
1	12.665	15.679	67.836	49.06	50.95
2	13.132	16.277	65.307	50.94	49.05
**Total:**		**31.957**	**133.143**	**100.00**	**100.00**

Chromatogram

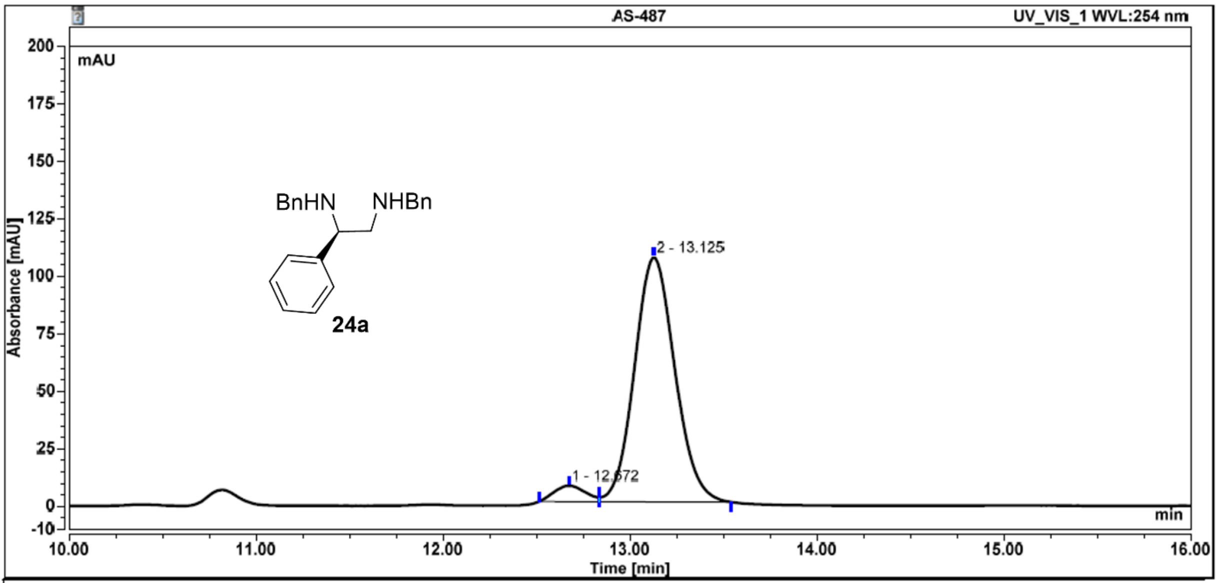

Integration Results

No.	Retention Time min	Area mAU*min	Height mAU	Relative Area %	Relative Height %
1	12.672	1.336	6.898	4.78	6.09
2	13.125	26.621	106.353	95.22	93.91
**Total:**		**27.956**	**113.251**	**100.00**	**100.00**

1,3-Dibenzyl-5-hydroxy-5-phenylimidazolidine-2,4-dione 21a

HPLC (Astec® Cellulose DMP, 80:20 hexane–EtOH, 1.0 mL/min, 210 nm)

Chromatogram

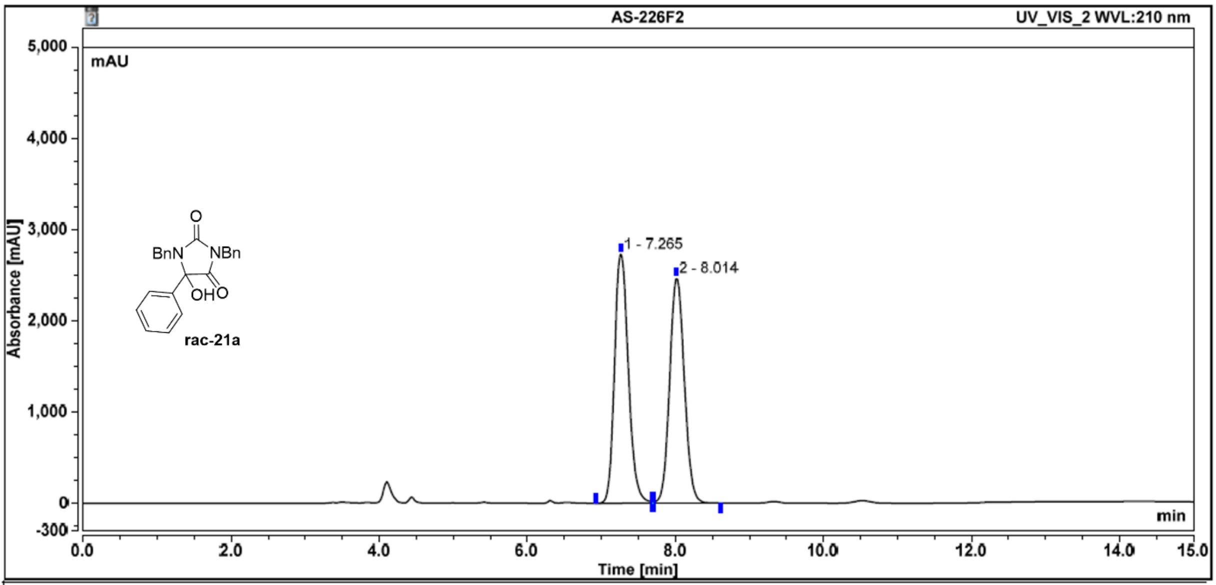

Integration Results

No.	Retention Time min	Area mAU*min	Height mAU	Relative Area %	Relative Height %
1	7.265	589.587	2734.906	50.93	52.56
2	8.014	568.144	2468.581	49.07	47.44
**Total:**		**1157.731**	**5203.487**	**100.00**	**100.00**
